# Structure–Activity Relationships Reveal Beneficial Selectivity Profiles of Inhibitors Targeting Acetylcholinesterase of Disease-Transmitting Mosquitoes

**DOI:** 10.1021/acs.jmedchem.3c00234

**Published:** 2023-04-24

**Authors:** Andreu Vidal-Albalat, Tomas Kindahl, Rajeshwari Rajeshwari, Cecilia Lindgren, Nina Forsgren, Stanley Kitur, Laura Sela Tengo, Fredrik Ekström, Luna Kamau, Anna Linusson

**Affiliations:** † Department of Chemistry, Umeå University, SE-90187 Umeå, Sweden; ‡ CBRN Defence and Security, Swedish Defence Research Agency, SE-90621 Umeå, Sweden; § Centre for Biotechnology Research and Development, Kenya Medical Research Institute, PO Box 54840-00200 Nairobi, Kenya

## Abstract

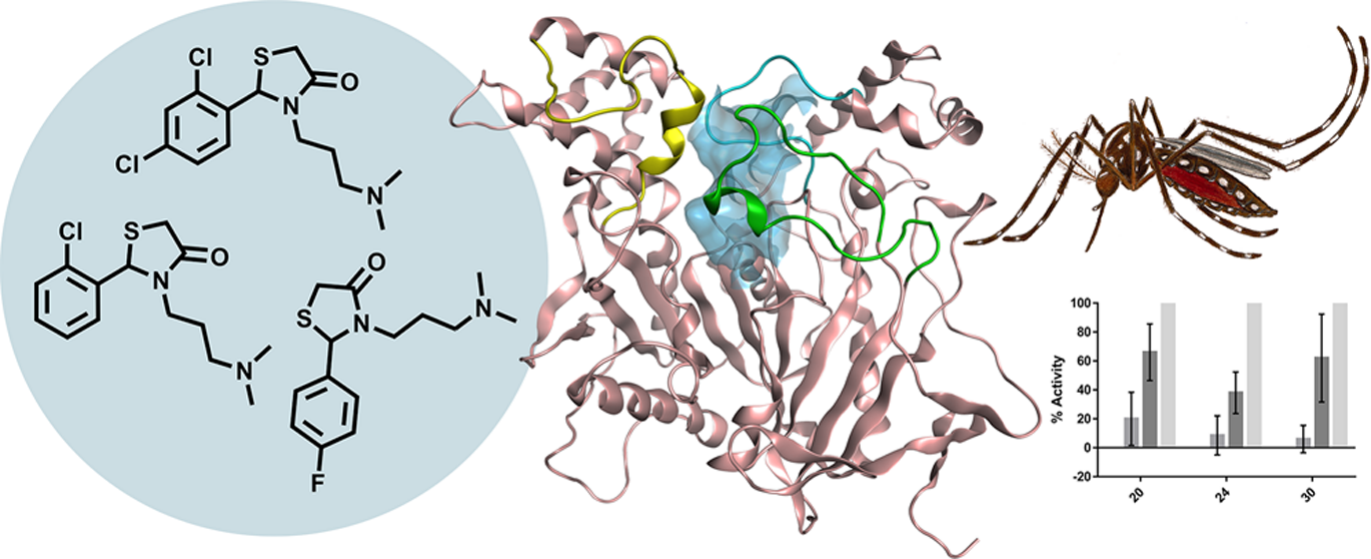

Insecticide resistance jeopardizes the prevention of infectious diseases such as malaria and dengue fever by vector control of disease-transmitting mosquitoes. Effective new insecticidal compounds with minimal adverse effects on humans and the environment are therefore urgently needed. Here, we explore noncovalent inhibitors of the well-validated insecticidal target acetylcholinesterase (AChE) based on a 4-thiazolidinone scaffold. The 4-thiazolidinones inhibit AChE1 from the mosquitoes *Anopheles gambiae* and *Aedes aegypti* at low micromolar concentrations. Their selectivity depends primarily on the substitution pattern of the phenyl ring; halogen substituents have complex effects. The compounds also feature a pendant aliphatic amine that was important for activity; little variation of this group is tolerated. Molecular docking studies suggested that the tight selectivity profiles of these compounds are due to competition between two binding sites. Three 4-thiazolidinones tested for in vivo insecticidal activity had similar effects on disease-transmitting mosquitoes despite a 10-fold difference in their in vitro activity.

## Introduction

Vector-borne diseases caused by viruses, parasites, and bacteria cause health, social, and economic problems that affect large numbers of people worldwide, especially in tropical and subtropical low- and middle-income countries. Mosquitoes are key vectors that transmit pathogens to the humans and animals on which they feed. In particular, mosquitoes of the *Anopheles*, *Aedes*, and *Culex* genera spread diseases such as malaria, dengue, chikungunya, and West Nile fever. In this study, we focus on the mosquito species *Aedes aegypti* (*Ae. aegypti*) and *Anopheles gambiae* (*An. gambiae*) that transmit, for example, dengue fever and malaria, respectively. Malaria alone was linked to over 600 000 deaths in 2020 according to the latest report by the World Health Organization (WHO).^1^ Although the estimated numbers of malaria infections and related deaths have decreased significantly since 2000, the incidence of infection has plateaued over the past seven years and increased in 2019 due to the disruption of services caused by the COVID-19 pandemic.^1,2^ The most efficient intervention for suppressing the spread of mosquito-borne diseases is vector control, which relies heavily on the effectiveness of the active ingredients in adulticides and larvicides. For example, insecticide-treated nets and indoor residual spraying played major roles in reducing the number of clinical cases of malaria between 2000 and 2015.^2^ However, the extensive use of a small number of insecticide classes has led to growing insecticide resistance in mosquito populations, threatening the effectiveness of current vector control strategies.^3−8^ Consequently, there is an urgent need to develop new active ingredients with different mechanisms of action. Additional tools for vector control that are being studied include biocontrol,^9−12^ genetic engineering of mosquitos,^9,13^ new chemical tools such as semiochemicals^14^ and nanoparticles,^15^ and strategies using various combinations of these tools.^16^

**Figure 1:**
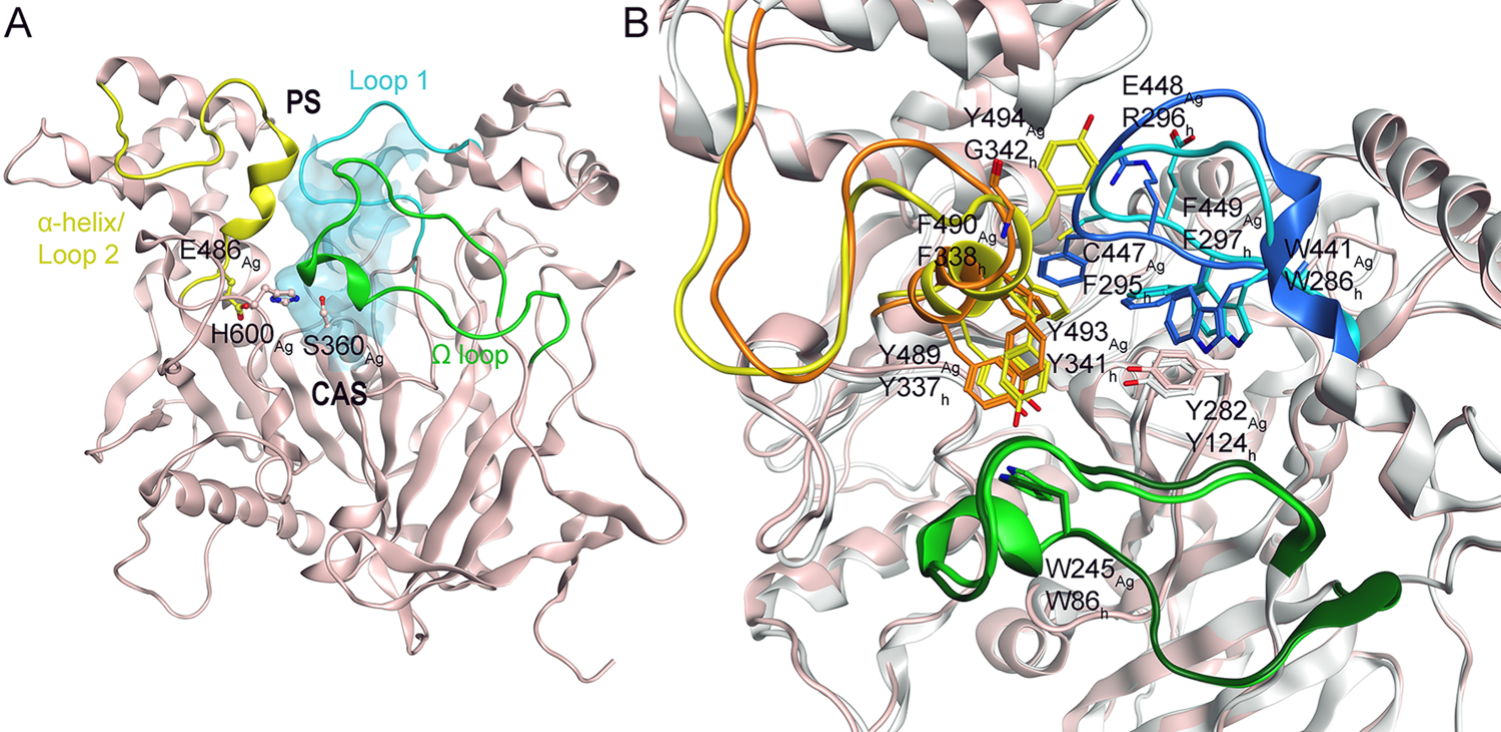
(A) Tertiary structure of *Ag*AChE1 with the active site gorge highlighted in cyan, displaying the catalytic triad (Ser360, His600, and Glu486) in the catalytic active site (CAS) as ball and sticks. The peripheral site (PS) is lined by loop 1 (blue), α-helix/loop 2 (yellow), and the Ω loop (green). (B) *Ag*AChE1 (pink ribbons) superpositioned to *h*AChE (gray ribbons) and amino acid residues important for inhibitor binding are displayed as sticks. Loop 1 is colored light and dark blue, α-helix/loop 2 is colored yellow and orange, and Ω loop is colored light and dark green for *Ag*AChE1 and *h*AChE, respectively. The figures are based on protein coordinates of previously published crystal structures (PDB codes 5X61 and 4EY4, for *Ag*AChE1 and *h*AchE, respectively).

An important molecular target for mosquito control is the enzyme acetylcholinesterase (AChE), which is present in both humans and animals.^17,18^ This essential enzyme terminates cholinergic neurotransmission by rapidly degrading the neurotransmitter acetylcholine in the synaptic cleft. Structural studies have shown that AChE has a deep and narrow active site gorge that is lined with aromatic amino acid residues.^19^ Three loops at the entrance of the gorge constitute the peripheral site (loops 1 and 2, plus the Ω loop), while the active site features a catalytic triad (serine, histidine, and glutamate) and is located near the bottom of the gorge (Figure 1A). Although humans and mammals express one AChE enzyme, most insects including mosquitoes express two AChE isoforms, designated AChE1 and AChE2.^20^ AChE1 is the more strongly expressed isoform in mosquitoes^21^ and is known to hydrolyze the same substrates as its vertebrate analogues, including human AChE (*h*AChE), with similar kinetic properties.^22,23^ Structural and sequence comparisons of *An. gambiae* AChE1 (*Ag*AChE1) and *h*AChE1 have shown that their active sites are identical but there are some differences in the three loops at the entrance of the gorge (Figure 1B). The conservation of the AChE active site across diverse organisms is an important disadvantage for two of the most widely used AChE-targeting insecticide classes, organophosphates and carbamates, because they both inhibit AChE by covalent bonding with the serine of the catalytic triad.^18,24^ This can cause severe poisoning and death in humans who are overexposed to these insecticides while applying them, and also gives rise to off-target ecotoxicity in beneficial species such as honeybees. Furthermore, mutations that confer resistance to these agents in disease-transmitting mosquitoes by preventing covalent inhibition occur in the active site gorge of AChE1. One of the most widespread resistance-conferring mutations is G122S-*Ag*AChE1,^25^ which is not present in *Ae. aegypti* expressing *Ae*AChE1.

To overcome the problems of toxicity towards other species and growing insecticide resistance, several attempts have been made to develop new chemical entities with the same mechanism of action as organophosphates and carbamates.^26−34^ Although improvements have been achieved, it has been difficult to obtain both selectivity for AChE1 over *h*AChE and potency against the resistance-conferring mutant G122S-*Ag*AChE1.^27−29,31,33^ An approach based on a different mechanism of action involves designing covalent inhibitors targeting a cysteine residue near the active site of *Ag*AChE1 (Cys447_Ag_), which is mutated into Phe295_h_ in *h*AChE (Figure 1B), in order to increase selectivity.^35,36^ While this approach is interesting, recent findings have raised questions about its viability because the compounds developed to date lack appreciable selectivity, and are proposed to have a different mode of irreversible binding not related to cysteine-targeted binding.^37^ Furthermore, no mosquitocidal activity has been reported for cysteine-targeting inhibitors. As an alternative to targeting the active site, there have been efforts to develop noncovalent inhibitors, which could potentially complement covalent inhibitors.^38−42^ Although studies in this area have yielded promising results, more research is needed to fully evaluate the potential of such inhibitors as active ingredients for vector control. Interestingly, phenotypic screening of compound libraries^43,44^ and plant extracts^45,46^ against mosquito larvae have revealed compounds targeting AChE1, highlighting its importance as a molecular target for a wider range of chemicals than organophosphates and carbamates. To clarify the potential of noncovalent inhibitors to serve as safe and efficient active ingredients, we here explore the inhibitory activity of a new chemical scaffold against AChE1 enzymes from the mosquitoes *An. gambiae* and *Ae. aegypti*. Our investigation focuses on the structure–activity relationships (SARs) for potency against wild-type AChE1 and selectivity over *h*AChE as well as identifying prerequisites for G122S-*Ag*AChE1 inhibition.

## Results and Discussion

### Identification of 4-Thiazolidinones as *Ag*AChE1 Inhibitors

**Figure 2:**
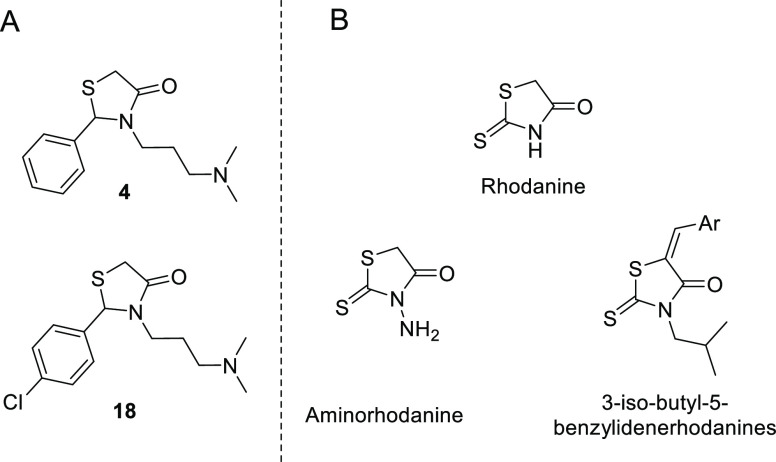
(A) 4-thiazolidinones with potential A*g*AChE1 inhibitory activity discovered through HTS. (B) Rhodamine derivatives previously tested as insecticides (bottom) and the parent compound rhodanine (top).

In a previously reported high-throughput screening (HTS) of 17 000 compounds against recombinantly expressed *Ag*AChE1 and *Ae*AChE1,^23,42^ we identified 4-thiazolidinones **4** and **18** as potential inhibitors of mosquito AChE1 (Figure 2). Compounds of this class are discussed extensively in the medicinal chemistry and patent literature because they exhibit diverse biological activities including anticancer, antibiotic, antiviral, antifungal, and cardiovascular activity. Consequently, their potential applications have been reviewed in detail.^47,48^ It has also been suggested that 4-thiazolidinones could be used to treat insect-borne diseases such as malaria^49,50^ and dengue fever.^51^ However, their use as insecticides for vector control has not previously been investigated to our knowledge. The most closely related compounds used for this purpose appear to be rhodanine derivatives^52,53^ bearing a thiocarbonyl in the 2-position of the ring, whose electronic properties and reactivity differ significantly from our HTS hits (Figure 2).

**Scheme 1:**
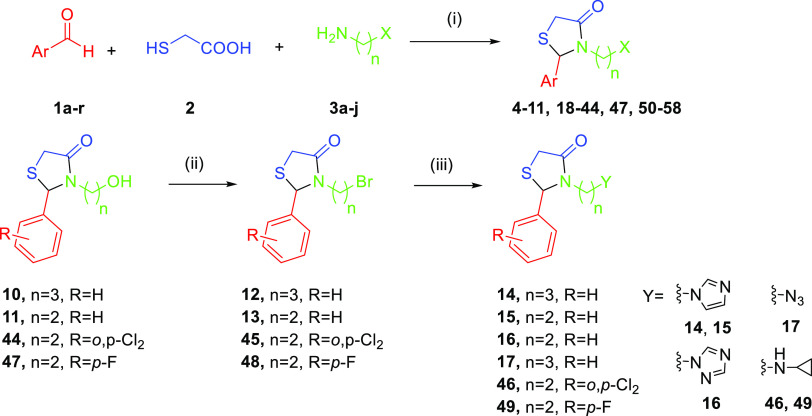
One-Pot and Stepwise Synthesis of 4-Thiazolidinone Analogues Conditions: (i) toluene, reflux, or EtOH, DIPEA (0 or 1 equiv), 120 °C, MW irradiation. (ii) CBr_4_, PPh_3_, DCM, 0 °C. (iii) DMF or ACN, base (K_2_CO_3_ 3 equiv or NaH 1 equiv), rt or 80 °C.

Several publications have described the synthesis of 4-thiazolidinones from commercially available reagents.^48−51,54^ The 4-thiazolidinone scaffold is readily prepared via a multicomponent reaction between an aldehyde (**1**), thioglycolic acid (**2**), and an amine (**3**) in which the starting materials are refluxed together using a Dean–Stark trap or molecular sieves to remove water (Scheme 1). We adapted this approach to prepare 4-thiazolidinones starting from diamines **3** and used microwave-assisted synthesis to shorten reaction times while achieving yields comparable to those of refluxing methods. The microwave-assisted strategy permits the use of diverse functional groups in starting materials **1** and **3**, allowing a wide range of compounds to be prepared in a simple one-pot process. We also designed a stepwise synthesis of these compounds for use in cases where the desired amine **3** is not commercially available (Scheme 1, bottom). In this alternative pathway, the initial multicomponent 4-thiazolidinone synthesis is performed using an amine with a terminal alcohol group, which is then transformed into the corresponding bromide via the Appel reaction, allowing the desired product to be finally obtained via an *S*_N_2 reaction. Using the one-pot approach, our screening hits **4** and **18** were synthesized as racemic mixtures in one step. Moreover, after performing an extractive work-up to remove water-soluble impurities, **4** and **18** were conveniently isolated as easily-handled solid salts by slow addition of HCl in ether to induce precipitation followed by filtration. This allowed us to verify the activity of **4** and **18** against *Ag*AChE1; their half-maximal inhibitory concentrations (IC_50_) were determined to be 5.4 and 0.86 μM, respectively, confirming the activity observed in the HTS. In addition, both **4** and **18** showed weak activity against *h*AChE, with IC_50_ values above 200 μM.

### Design Strategy

**Scheme 2:**
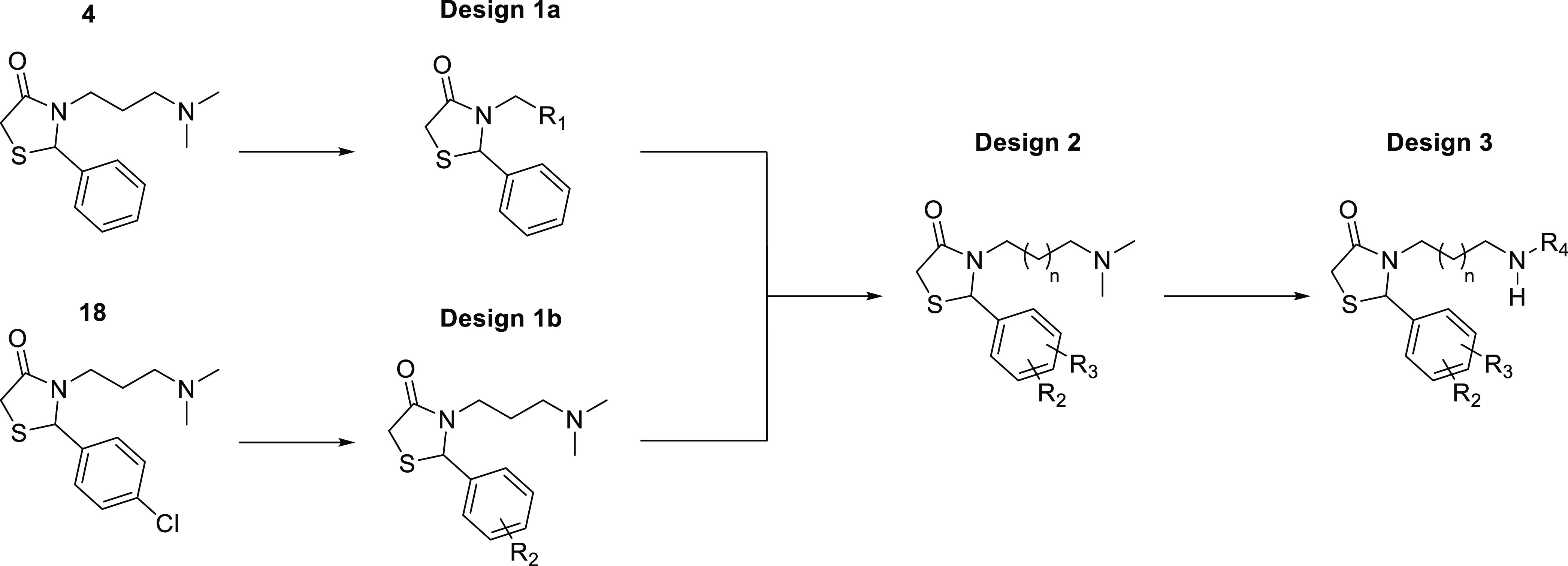
Design Strategy Based on the Two Hit Compounds

We designed two sets of 4-thiazolidinones for synthesis and biochemical evaluation. The first set had **4** as their parent compound (Design 1a), while the second set was derived from **18** (Design 1b; Scheme 2). The set 1a compounds were designed to investigate the effects of varying the substituents of the aliphatic amine and of replacing the amine itself with hydroxy, azido, aromatic, or heteroaromatic groups (Table S1). The effect of varying the length of the pendant carbon chain was also investigated. The set 1b compounds were designed to investigate the effect of modifying the aromatic ring by adding electron-donating or electron-withdrawing substituents at various positions, introducing fused rings, or replacing the phenyl ring with a heteroarene (Table 1). We then designed a third set of derivatives (Design 2) that combined the aromatic substituents and alkyl chain lengths with the most promising effects on activity from designs 1a and 1b while retaining the terminal dimethylamine moiety of **4** and **18** (Tables 2 and S2). Finally, a fourth set (Design 3; Scheme 2) was designed in which the best aromatic scaffolds from design sets 1 and 2 were combined with various secondary amine motifs (Tables 3 and S3). All of the designed and synthesized compounds were tested for potency towards *Ag*AChE1 and *h*AChE by measuring their IC_50_ values. Some compounds were also tested against the mutant G122S-*Ag*AChE1 and *Aa*AChE1, and a few were selected for enzyme kinetics studies and evaluation of their in vivo activity against *An. gambiae* and *Ae. aegypti*.

**Table 1:**
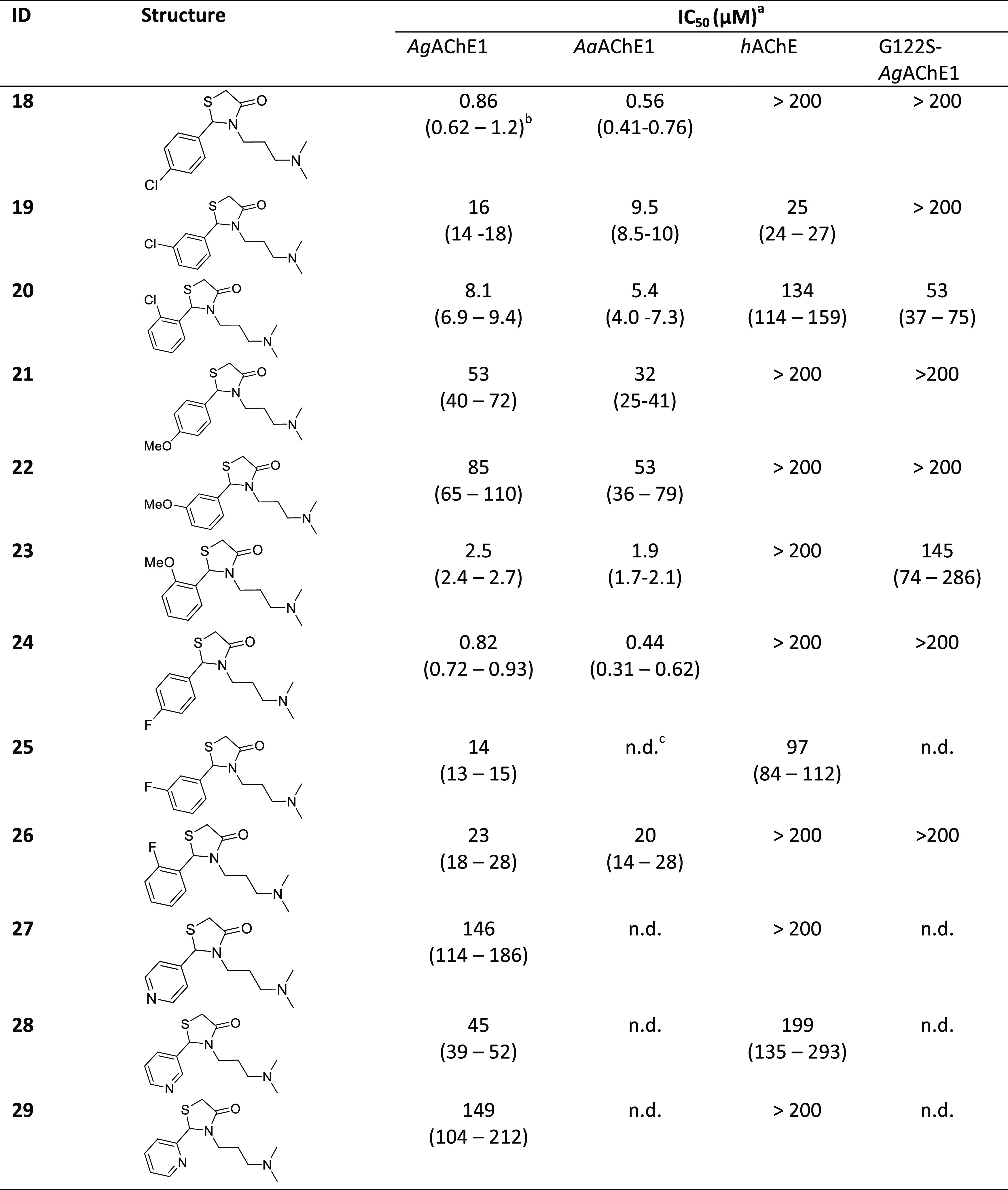
Chemical Structures and IC_50_ Values for Compounds in Set 1b

a Compounds tested as HCl salts unless specified.

b 95% confidence interval given in parentheses.

c n.d. = not determined.

**Table 2:**
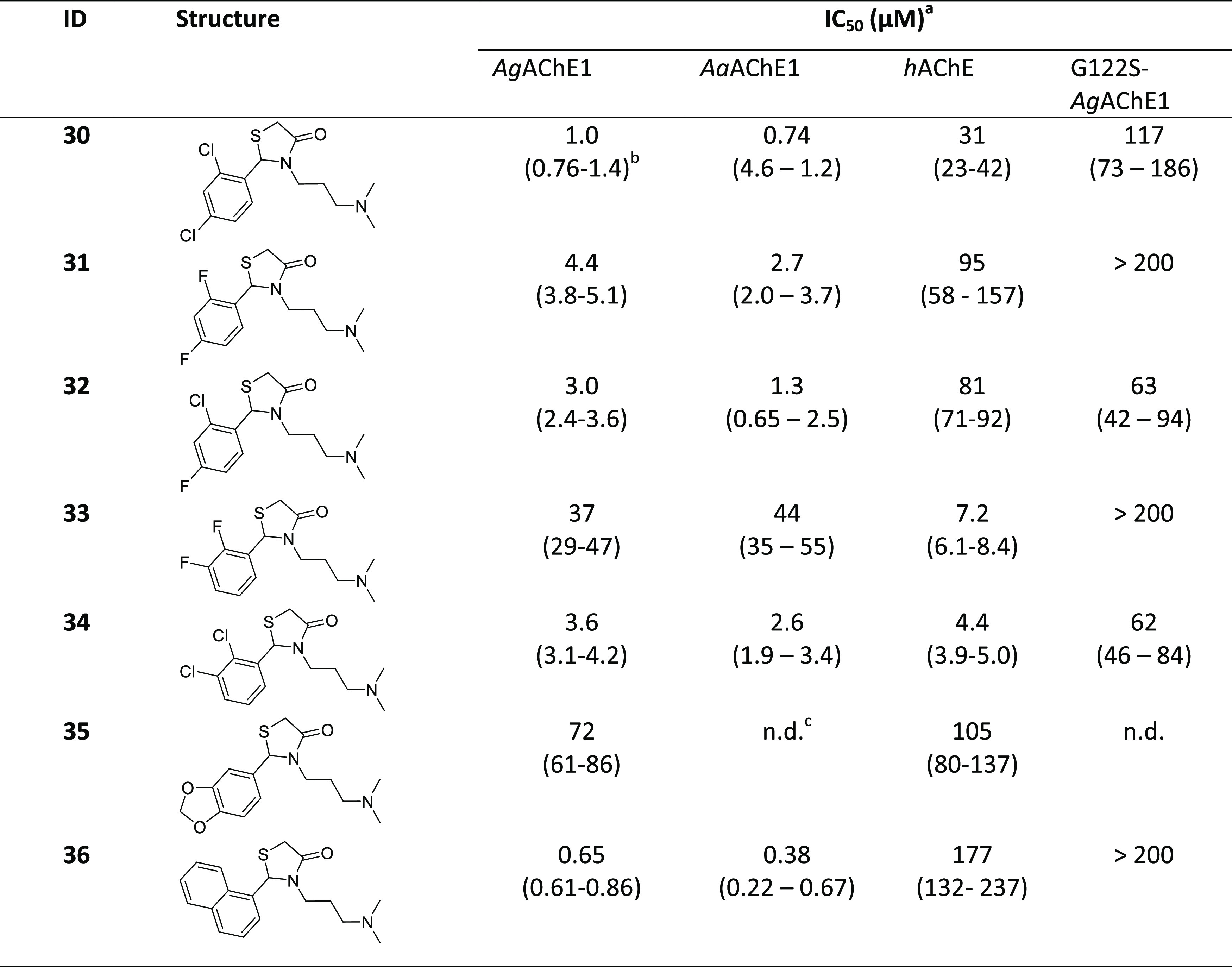
Chemical Structures and IC_50_ Values for Compounds in Set 2

a Compounds tested as HCl salts unless specified.

b 95% confidence interval given in parentheses.

c n.d. = not determined.

**Table 3:**
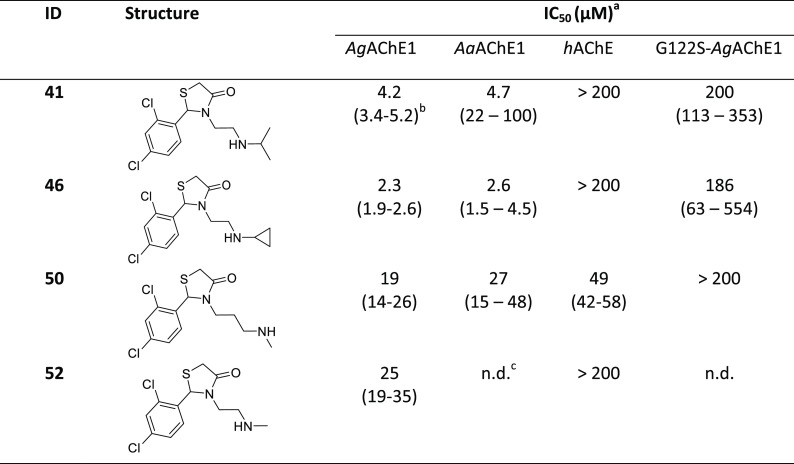
Chemical Structures and IC_50_ Values for Compounds in Set 3

a Compounds tested as HCl salts unless specified.

b 95% confidence interval given in parentheses.

c n.d. = not determined.

### Design 1a: Exploration of N-Substitution Based on **4**

All compounds in set 1a (**5**–**17**, Table S1) were synthesized as shown in Scheme 1 and tested for inhibitory activity against *Ag*AChE1 and *h*AChE. If the 2-phenyl-4-thiazolidinone unit was left unmodified, modifying the pendant amine moiety by changing the N-substituents had a clear adverse effect on *Ag*AChE1 inhibition; no compounds modified in this way exhibited superior or even similar potency to **4**. None of the analogues of **4** achieved complete inhibition of the enzyme even at high concentrations (1 mM). Shortening the chain length as in **5** reduced inhibitory potency toward the mosquito enzyme considerably compared to **4** (IC_50_ = 161 vs 5.4 μM, respectively). Replacing the dimethylamino moiety with a diethylamino group also reduced activity in analogues with 3- or 2-carbon alkyl chains (**6** and **7**, respectively). Similar results were observed upon replacing the dimethylamino unit with a heterocyclic substituent such as a morpholine (**8**), imidazole (**14**, **15**), or triazole (**16**). Compounds lacking a pendant amino group (**9**, **10**, **17**) showed no detectable inhibitory activity towards *Ag*AChE1 even at a concentration of 1 mM. The reference compound **4** and all the analogues shown in Table S1 exhibited little activity against *h*AChE even at high concentrations. Overall, these experiments clearly showed that retaining the pendant dimethylamine moiety with a 3-carbon chain is important for *Ag*AChE1 inhibition.

### Design 1b: Exploration of Aromatic Substitution Based on **18**

The compounds in set 1b, in which the aromatic moiety of lead compound **18** was modified, are listed in Table 1 and were synthesized following Scheme 1. Biochemical evaluation of these compounds revealed that the types and positions (ortho, meta, or para) of the substituents on the phenyl ring strongly affected *Ag*AChE1 inhibition. For example, shifting the para chlorine atom of the parent compound **18** to the ortho position (as in compound **20**) or the meta position (as in compound **19**) increased the IC_50_ values from 0.86 to 8.1 μM and 16 μM, respectively. However, the ortho methoxy-substituted compound **23** had an IC_50_ value of 2.5 μM against *Ag*AChE1, whereas its para and meta substituted analogues **21** and **22** were less active (IC_50_ = 53 and 85 μM, respectively). The para compound **24** was the strongest inhibitor of the three fluorinated analogues (0.82 μM), followed by the meta substituted **25** and the ortho substituted **26** (IC_50_ = 14 and 23 μM, respectively). Replacing the phenyl ring with a pyridine moiety reduced activity against the mosquito enzyme; the pyridinyl compounds **27**, **28**, and **29** were the weakest inhibitors of this subset. The set 1b compounds were substantially less active against *h*AChE than *Ag*AChE1; only the meta chlorinated compound **19** exhibited even moderate inhibition of *h*AChE (IC_50_ = 25 μM). The other analogues were even less active toward the human enzyme (Table 2). For example, the para and ortho chlorinated analogues **18** and **20** had IC_50_ values above 100 μM, while the methoxy analogues (**21**, **22**, and **23**) had IC_50_ values > 200 μM.

Overall, the para substituted chlorinated and fluorinated analogues **18** and **24** were the strongest inhibitors. Encouragingly, these two were also poor inhibitors of *h*AChE and thus showed the highest selectivity in the subset. The least selective compound was the meta chlorinated compound **19**, which was equipotent against the human and mosquito enzymes.

### Design 2: Follow-Up Design Based on Initial Findings

In Design 2, we investigated the effect of varying the aromatic moiety in more detail by examining disubstituted analogues bearing Cl and F atoms in different positions (Table 2). We also examined the effect of replacing the phenyl ring with larger aromatic moieties such as naphthyl and benzodioxole groups. All of the 11 analogues in this set had a pendant 3-carbon alkyl chain with a terminal dimethylamino moiety like the parent compound **18** and the compounds listed in Table 1. Separately, we prepared some analogues bearing the new aromatic groups with a 2-carbon dimethylamino chain to see if the loss of activity due to modification of the pendant amine would be as noticeable as in set 1a (Table S2).

The evaluation of these compounds against *Ag*AChE1 (Table 2) showed that the naphthyl analogue **36** had the lowest IC_50_ value of the series (0.65 μM), meaning that larger substituents were tolerated. However, the increased hydrophobic character of **36** reduced its solubility at higher concentrations. The ortho, para dichlorinated compound **30** had a similar inhibitory potency to its para monosubstituted counterpart. On the other hand, the ortho, para difluorinated analogue **31** had an IC_50_ of 4.4 μM, while compound **32**, which contains both chlorine and fluorine, had an IC_50_ of 3.0 μM. The ortho, meta dichlorinated compound **34** (3.6 μM) was a weaker inhibitor of *Ag*AChE1 than its ortho, para dichlorinated analogue **30** but was a stronger binder than the corresponding difluoro species **33** (37 μM). Replacing the phenyl group with a benzodioxole moiety, as in **35**, reduced inhibitory activity towards *Ag*AChE1. The two-carbon chain analogues **37**, **38**, **39**, and **40** were substantially weaker than their 3-carbon chain counterparts, confirming the results obtained with set 1a (Tables S1 and S2). In addition, the biochemical evaluations indicated that the SAR of these compounds for inhibition of *h*AChE differed from that for inhibition of *Ag*AChE1. Based on their IC_50_ values, most of the tested compounds (including **30**, **31**, and **32**) were at least 20 times weaker inhibitors against human AChE than against the mosquito enzyme, and **36** was 2 orders of magnitude stronger binder against the mosquito enzyme. Interestingly, the compounds with ortho, meta disubustituted aromatic moieties (**33** and **34**) were rather strong inhibitors against the human enzyme, with IC_50_ values of 7.2, and 4.4 μM, respectively. As a result, **34** was an equipotent inhibitor of the human and mosquito enzymes, while **33** inhibited the human enzyme even more strongly than *Ag*AChE1. The 2-carbon chain compounds **37**, **38**, **39**, and **40** were inactive even at highest tested concentrations (Table S2).

### Design 3: Focused Exploration of Secondary Amines

In the third design set, we synthesized and biochemically evaluated compounds to investigate the effect of replacing the pendant tertiary amine with a secondary amine. We combined three of the most promising aromatic scaffolds (**24**, **30**, and **36**) with four secondary amines connected to the 4-thiazolidinone core by 2- and 3-carbon chains: an isopropylamine, a cyclopropylamine, and a monomethylamine connected via a 2-carbon chain, and a monomethylamine connected via a 3-carbon chain (Tables 3 and S3). Four compounds combining the 2-carbon chain isopropylamine moiety with fluorinated aromatic scaffolds (cf. **55**, **56**, **57**, and **58**) were also investigated; see Table S3.

The compounds with more sterically demanding secondary amines (cyclopropylamines and isopropylamines) were stronger inhibitors of *Ag*AChE1 than the compounds with monomethyl amines. The most active compound in this series was **46**, which has an ortho, para dichlorophenyl ring and a pendant cyclopropylamine; its IC_50_ value was 2.3 μM. The second most active compound was its isopropyl analogue **41** (4.2 μM), while the methylamine analogues **50** and **52** were less active (Table 3). Interestingly, the ortho and para chlorinated analogues were stronger inhibitors than their para fluorinated and naphthyl counterparts when the secondary amine was introduced (Tables 3 and S3) even though these aromatic substituents conferred similar activity in the previous compound sets featuring pendant tertiary amines. Among the para fluorophenyl compounds, the cyclopropylamine **49** had the lowest IC_50_ value (11 μM), followed by the isopropylamine analogue **42** (18 μM). The naphthyl compounds **54** and **43** were over 10 times weaker binders than their tertiary amine analogues. The other fluorinated scaffolds gave IC_50_ values above 30 μM when combined with a pendant isopropylamine. The ortho, para dichlorophenyl compound with a pendant methylamine unit (**50**) exhibited the highest activity against *h*AChE in this set, with an IC_50_ value of 49 μM; **50** was the only compound in this set that completely inhibited the human enzyme in vitro (Tables 3 and S3).

### Biochemical Evaluation against the Insecticide-Resistant Mutant Enzyme G122S-*Ag*AChE1

Some of the synthesized 4-thiazolidinones were selected for evaluation against the insecticide-resistant mutant G122S-*Ag*AChE1 (Tables 1–3, S2 and S3) based on their activity profiles against the wild-type mosquito and human enzymes. The results showed that the 4-thiazolidinones also inhibited the enzymatic activity of G122S-*Ag*AChE1, albeit with lower potency and a different SAR to that observed for the wild-type enzyme. The most active compounds had an ortho chlorinated aromatic ring, and a tertiary amine group: the singly substituted ortho chloro compound **20** had an IC_50_ value of 53 μM against G122S-*Ag*AChE1, whereas the ortho chloro, para fluoro compound **32** and the ortho, meta dichloro compound **34** had IC_50_ values of 63 and 62 μM, respectively. The ortho, para dichloro compound **30** was slightly less active against the mutant enzyme (117 μM). The para chloro lead compound **18** was substantially less active against the mutant; its IC_50_ value could not be determined under our assay conditions, indicating that it was above 200 μM, compared to 0.86 μM for wild-type *Ag*AChE1. The mutation of a glycine to a serine at position 122 in the binding gorge thus profoundly alters the 4-thiazolidinone SAR for the mosquito enzymes. This was further confirmed by the inactivity of the naphthyl analogue **36** against G122S-*Ag*AChE1 (IC_50_ > 200 μM) despite its good activity against wild-type *Ag*AChE1 (IC_50_ = 0.65 μM).

### Structure–Activity Relationships of 4-Thiazolidinones Inhibiting AChE

**Figure 3:**
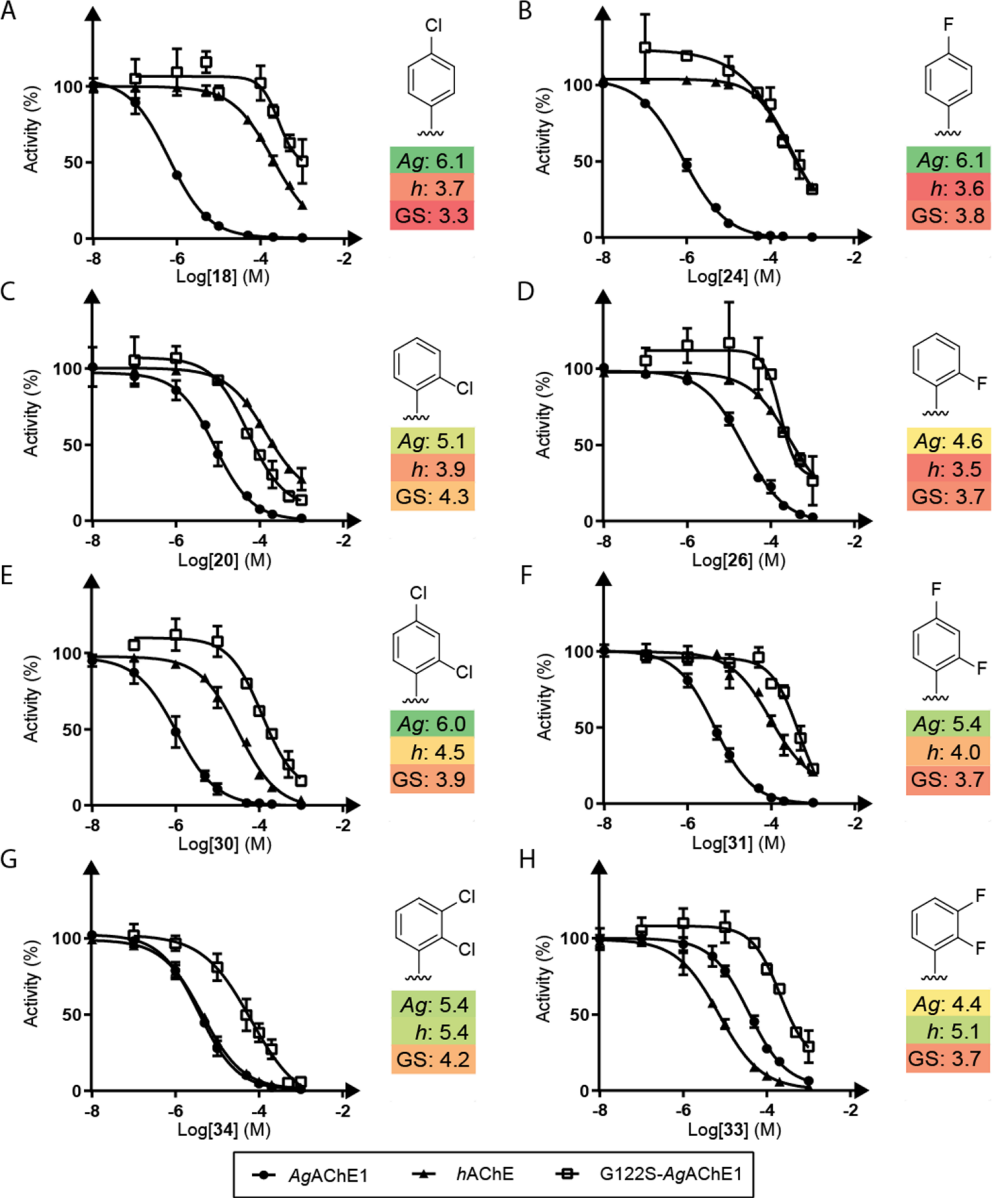
Inhibition profiles of 3-carbon chain dimethylamine 4-thiazolidinones with different halogenated aromatic substituents against *Ag*AChE1 (circular dots), *h*AChE (triangular dots), and G122S-*Ag*AChE1 (empty square dots). The monosubstituted inhibitors **18**, **24**, **20**, and **26** (A–D) and the disubstituted inhibitors **30**, **31**, **34**, and **33** (E–H) are shown. pIC50 values are given next to the dose–response curves and are color-coded, with dark green and dark red indicating the strongest and weakest inhibitors, respectively.

Variation of the aromatic substituent of the 4-thiazolidinone scaffold revealed that chlorinated and fluorinated inhibitors were the most promising inhibitors of *Ag*AChE, with the para substituted inhibitors **18** and **24** being particularly strong binders. Interestingly, the compounds’ inhibition profiles for the studied AChEs depended on their substitution patterns: the para substituted compounds **18** and **24** showed the highest selectivity for the wild-type mosquito enzyme over the human enzyme but exhibited poor activity against the resistant mutant G122S-*Ag*AChE (Figure 3a,b). Conversely, the ortho chloro analogue **20** exhibited greater potency against G122S-*Ag*AChE but was a weaker inhibitor of *Ag*AChE, leading to poor selectivity over *h*AChE (Figure 3c). Its fluorinated analogue **26** was a poor binder overall, and therefore less attractive (Figure 3d). Substitution at both the ortho and para positions in the dichlorinated **30** and the difluorinated **31** led to increased inhibitory activity against both *Ag*AChE and *h*AChE (Figure 3e–f), but **30** was a weaker inhibitor of the resistant mutant than its ortho monochlorinated counterpart **20**. Changing the disubstitution pattern from ortho/para to ortho/meta (**34** and **33**) dramatically altered the inhibition profiles: both the dichloro and difluoro ortho, meta analogues were completely unselective for the mosquito enzyme, and the ortho, meta difluoro compound **33** was actually a stronger inhibitor against the human enzyme than against *Ag*AChE (Figure 3g,h). Removing the halogen from the ortho position to obtain the singly-halogenated meta substituted compounds **19** and **25** led to a general loss of inhibitory activity in the case of **19**, but the meta fluorinated compound **25** was a stronger inhibitor than **33** against *Ag*AChE while being weaker against *h*AChE and thus regained selectivity for the mosquito enzyme (Table 1).

An SAR analysis of the pendant dimethylamine using analogues of the para fluorinated compound **24** and the ortho/para dichlorinated compound **30** revealed that the monofluorinated scaffold was more sensitive to modification of the pendant alkylamine (Tables 3 and S3). For example, replacing the 3-carbon chain dimethylamine moiety with a cyclopropylamine connected via a 2-carbon chain led to a 13-fold loss of potency for the para fluorinated scaffold (**49** vs **24**) but only a 2-fold loss of potency for the ortho, para dichlorinated scaffold (**46** vs **30**). A similar pattern was seen for the isopropylamines (cf. **42** and **41** vs **24** and **30**, respectively). The inhibition profiles of the cyclopropylamine derivatives closely resembled those of the 3-carbon chain dimethylamines.

### Molecular Docking of 4-Thiazolidinones to AChEs

To further investigate the SAR of 4-thiazolidinones, we performed molecular docking simulations of eight inhibitors to *Ag*AChE1, *h*AChE, and the mutant enzyme G122S-*Ag*AChE1. The chosen inhibitors had fluoro or chloro substituents in the ortho and/or para positions of the phenyl ring together with a pendant 3-carbon dimethylamine chain (Figure S5). The three-dimensional (3D) structures of the enzymes used in this analysis were previously determined by X-ray crystallography (PDB codes 5X61, 4EY4/6O5V and 6ARX for *Ag*AChE1, *h*AChE, and G122S-*Ag*AChE1, respectively). The crystal structures of AChEs reveal several waters in the active site gorge, thus we have performed docking to AChE structures with included and excluded water molecules (Tables S23–S27). The R and S enantiomers of the inhibitors with positively charged dimethylamines were separately docked into the binding gorge of the AChEs using the Schrödinger software package and multiple docking poses for each inhibitor were analyzed.

**Figure 4:**
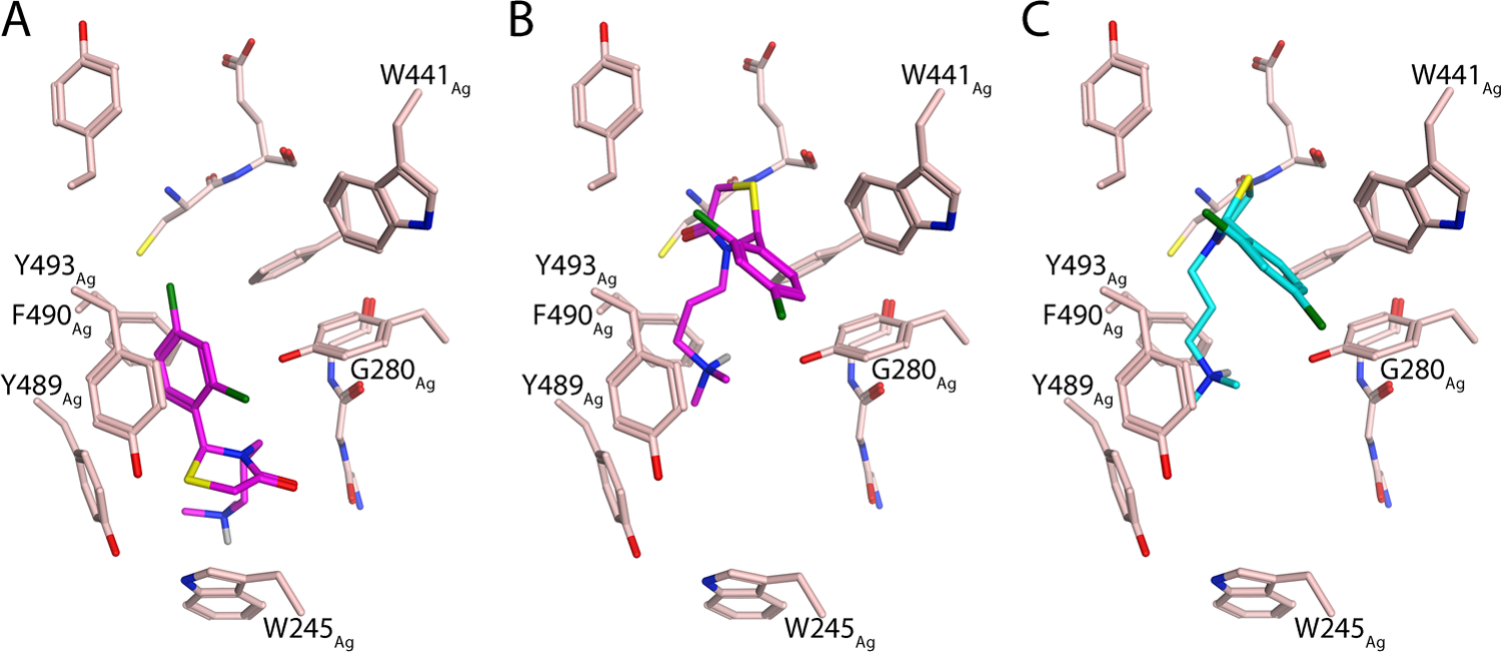
Docking poses of selected 4-thiazolidinones binding to *Ag*AChE1: pose A and B of **30S** (A, B) and pose B of **30R** (C). Dockings were performed to protein coordinates of a previously published crystal structure (PDB code 5X61).

**Figure 5:**
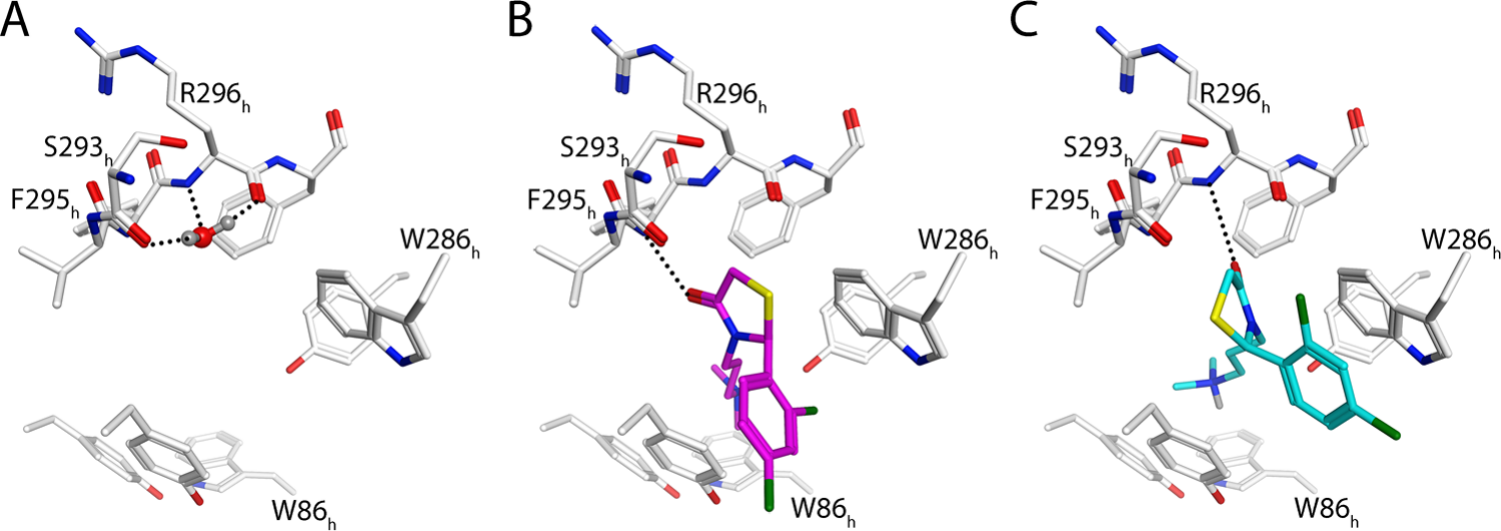
Docking poses of selected 4-thiazolidinones binding to *h*AChE were restricted by a water molecule (A) and only when this water molecule was removed, pose B of **30S** and **30R** was observed (B, C). Dockings were performed to protein coordinates of a previously published crystal structure (PDB code 6O5V).

The docking studies clearly showed that the inhibitors adopted different binding poses in each of the target enzymes. The dockings with included waters gave in general fewer poses. In *Ag*AChE1 without waters, the docked 4-thiazolidinones had two main poses: pose A, in which they bound at the bottom of the gorge, close to the catalytic triad; and pose B, in which they bound at the peripheral site (Figure 4). The dockings to *Ag*AChE1 structure with waters resulted in fewer number of poses of type A, although still observed. Only pose B was observed when docking with *h*AChE without waters. Interestingly, when waters were included it revealed that in order for inhibitors to form pose B, a water molecule had to move (Figure 5). Docking with G122S-*Ag*AChE1 with and without waters generally yielded few promising poses, most of which had significantly lower docking scores than those for the other two enzymes. Nevertheless, pose B was also observed for G122S-*Ag*AChE1. The enantiomeric preferences of each binding pose were complex. For *Ag*AChE1, pose A was only observed for the S enantiomers, whereas pose B could be adopted by both R and S enantiomers in *Ag*AChE1 (with and without waters) and *h*AChE (without waters). The few promising type B poses of 4-thiazolidinones binding to G122S-*Ag*AChE1 all involved R enantiomers.

In *Ag*AChE1, the 4-thiazolidinones bind to the active site (pose A) via two main interactions: an interaction between the charged protonated nitrogen of the dimethylamine moiety and Trp245_Ag_ (cf. Trp86_h_), and an arene–arene stacking interaction between the phenyl rings of Tyr493_Ag_ (cf. Tyr341_h_) and the 4-thiazolidinone. The aromatic rings of Tyr489_Ag_ and Phe490_Ag_ also participate in many-body arene–arene interactions with the 4-thiazolidinones, as previously reported for inhibitors of mouse AChE (cf. Tyr337_h_ and Phe338_h_).^55,56^ A superposition of the structures of *Ag*AChE and *h*AChE showed that the side chains of Tyr489_Ag_/Tyr337_h_ have different conformations in these enzymes. The conformation of Tyr489_Ag_ makes the binding pocket of *Ag*AChE somewhat larger than that of *h*AChE, which allowed the 4-thiazolidinones to be docked into the active site of the former but not the latter. This is consistent with the findings of Carlier et al., whose studies on carbamate AChE1 inhibitors revealed that larger β- and γ-branched 1-alkylpyrazol-4-yl methylcarbamates are more selective for inhibition of *Ag*AChE versus *h*AChE than their smaller analogues.^33^ The G122S mutation is not close in space to the docked pose A (the shortest heavy atom distance between the mutant residue and the inhibitor is 5.2 Å), so there does not appear to be a direct steric clash that would prevent the 4-thiazolidinones from adopting binding poses at the bottom of the gorge. The binding gorge residue exhibiting the largest conformational difference between G122S-*Ag*AChE and *Ag*AChE is Tyr493_Ag_ (cf. Tyr341_h_), which makes the gorge in the G122S mutant slightly narrower than that in the wild-type enzyme. This may explain the lack of A-type binding poses for the mutant.

Both enantiomers of all of the 4-thiazolidinones binding in pose B formed similar interactions at the peripheral sites of the studied AChE species: the phenyl moiety formed a non-optimal arene–arene interaction with Trp441_Ag_/Trp286_h_; the carbonyl of the thiazolidinone ring formed a hydrogen bond with backbone NH moieties of amino acid residues in loop 1; and the 3-carbon chain with the positively charged (protonated) dimethylamine moiety projected down into the gorge and formed N^+^CH–arene interactions with Tyr493_Ag_/Tyr341_h_, Tyr489_Ag_/Tyr337_h_, or Phe490_Ag_/Phe338_h_. In *Ag*AChE1, the interacting moieties of the R and S enantiomers overlapped whereas in *h*AChE there were more pronounced differences between the enantiomers: the S enantiomers formed hydrogen bonds with the NH of Phe295_h_, whereas the R enantiomers formed hydrogen bonds with Arg296_h_. Both B poses in *h*AChE resulted in clashes between the −CH_2_– part of the five-membered ring, and the water molecule forming H-bonds with Ser293_h_ and Arg296_h_ (Figure 5a). None of the investigated crystal structures (Tables S24 and S25) showed ligands replacing this water molecule. The differences between the binding poses and the water patterns in *Ag*ACHE and *h*AChE depended on amino acid sequence differences in loop 1 and the Ω-loop. Loop 1 of *Ag*AChE1 is three residues shorter than the corresponding loop in *h*AChE, and the Ile231_Ag_ residue in the Ω-loop of the mosquito enzyme is replaced by a tyrosine residue (Tyr72_h_) in the human enzyme, which explained the conformational differences between the corresponding docked poses and differences in water interactions. For G122S-*Ag*AChE, only the R enantiomers adopted pose B and the binding pose of the 4-thiazolidinones was similar to that seen with the wild-type enzyme. However, the N^+^CH–arene interactions differed because the conformation of Tyr493_Ag_ in G122S-*Ag*AChE differs from that in the human and wild-type mosquito enzymes (as discussed above), which probably explains why the docking scores for the mutant were lower.

Comparing the docking poses of the different compounds more closely, the docking studies suggested that the ortho and para substituted 4-thiazolidinones were able to bind in a deeper position within the binding gorge of *Ag*AChE (pose A) than in *h*ACHE and G122S-*Ag*AChE, which is consistent with their selectivity profiles as inhibitors. The −Cl and −F substituents of **18** and **24** formed contacts with hydrogens of the aromatic ring of Phe490_Ag_ (cf. Phe338_h_) and the β carbon of Tyr493_Ag_ (cf. Tyr341_h_), respectively, suggesting that electrostatic interactions involving these halogen substituents may favor this binding mode. In contrast, pose B at the peripheral site could be adopted by all docked compounds in all three investigated AChE species, although a water molecule needed to be replaced in *h*AChE. It should be noted that the side chain of Trp286_h_ can adopt different conformations in mammalian AChE in the presence of different ligands, as demonstrated by X-ray crystallography.^57^ Few crystal structures of *Ag*AChE have been published, so there is insufficient experimental data to draw conclusions about the mobility of amino acid side chains in the peripheral site of this enzyme. However, based on amino acid sequence differences we can assume that it differs from that in *h*AChE. We therefore hypothesize that the observed dependence of inhibitory potency on the substitution pattern of the phenyl ring in 4-thiazolidinone derivatives is partly due to interactions with Trp441_Ag_/Trp286_h_ in the binding site. Accordingly, we propose that the greater potency of the ortho, meta disubstituted compounds **33** and **34** towards *h*AChE and their reduced selectivity compared to other inhibitors are due to favorable interactions with Trp286_h_ in conformations other than the docked poses. In all of the binding poses, the pendant dimethylamine moiety formed key interactions with aromatic enzyme residues, in accordance with the SAR analysis.

### Evaluation of Insecticidal Effects of 4-Thiazolidinones on Disease-Transmitting Mosquitoes

**Figure 6:**
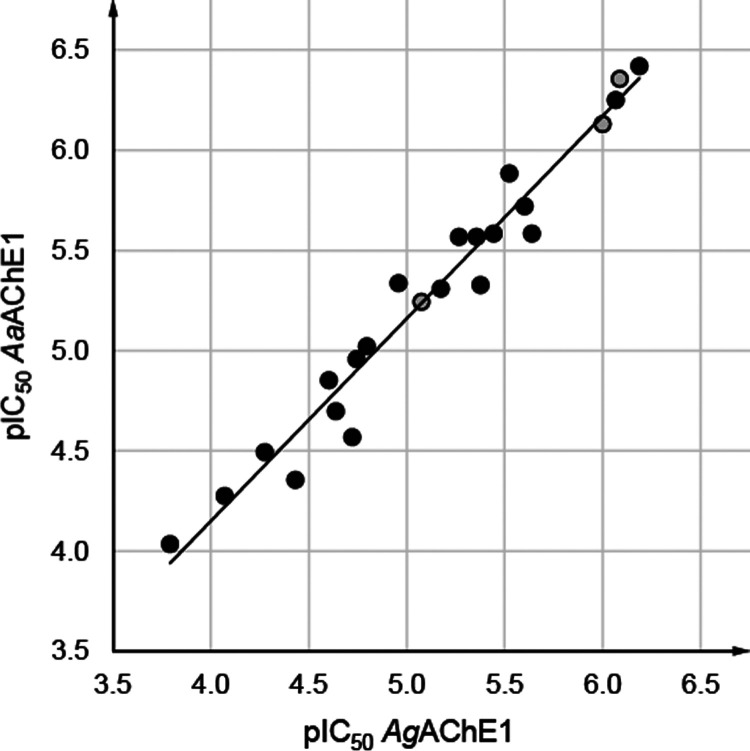
In vitro inhibition of AChE1 enzymes from mosquito species *An. gambiae* (*x*-axis) and *Ae. aegypti* (*y*-axis) by the studied 4-thiazolidinones expressed as pIC_50_. The square of the Pearson correlation coefficient *r*^2^ for the two mosquito enzymes’ pIC_50_ values is 0.96.

The insecticidal potential of selected 4-thiazolidinones was evaluated against the mosquito species *An. gambiae* and *Ae. aegypti*. First, 23 compounds were tested for inhibitory activity against recombinantly expressed *Aa*AChE1, which confirmed that their IC_50_ values for both mosquito enzymes were comparable and that they exhibited similar SAR patterns (Figure 6, Tables 1–3, S2 and S3). Three 4-thiazolidinones with different potency (**20**, **24**, and **30**) were then selected for further investigation. Inhibition kinetics was used to determine the compounds’ inhibition constant (*K*_i_) and mode of inhibition (Table 4). The selected compounds were investigated for inhibition of *Ag*AChE1, G122S-*Ag*AChE1, and *h*AChE using different substrate and inhibitor concentrations (Tables S28–S34). We found that the inhibitors decreased the maximum velocity (*V*_max_) in a dose-dependent manner. In contrast, the *K*_m_ values (substrate concentration at 50% of *V*_max_) were constant. These observations are consistent with a noncompetitive inhibition mechanism for all compounds and enzymes. The kinetic inhibition constants of **20**, **24**, and **30** determined by noncompetitive inhibition curve fitting of kinetic data are shown in Tables 4 and S35. For *Ag*AChE1, the kinetic inhibition constants were in good agreement with the IC_50_ values, while there were some differences for G122S-*Ag*AChE1 and *h*AChE. Compound **30**, with a poor IC_50_ value of 117 μM, was shown to have a moderate *K*_i_ value of 19 μM toward G122S-*Ag*AChE1. In contrast, inhibition of *h*AChE was slightly weaker according to the *K*_i_ values of **20** and **30**, compared to the IC_50_ measurements.

**Table 4:** Kinetic Inhibition Constants (*K*_i_) and IC_50_ Values of Noncompetitive Inhibitors Selected for In Vivo Studies^b^

Enzyme	ID	*K*_i_ (μM)^a^	IC_50_ (μM)^a^
*Ag*AChE1	**20**	**5.4**	**8.1**
		(4.2–6.8)	(6.9–9.4)
	**24**	**0.73**	**0.82**
		(0.57–0.95)	(0.72–0.93)
	**30**	**1.0**	**1.0**
		(0.9–1.2)	(0.76–1.4)
G122S-*Ag*AChE1	**20**	**54**	**53**
		(42–70)	(37–75)
	**24**	n.d.^c^	**>200**
	**30**	**19**	**117**
		(14–24)	(73–186)
*h*AChE	**20**	**231**	**134**
		(199–267)	(114–159)
	**24**	n.d.^c^	**>200**
	**30**	**65**	**31**
		(58–74)	(23–42)

a Compounds tested as HCl salts.

b 95% confidence interval given in parentheses.

c n.d. = not determined.

**Figure 7:**
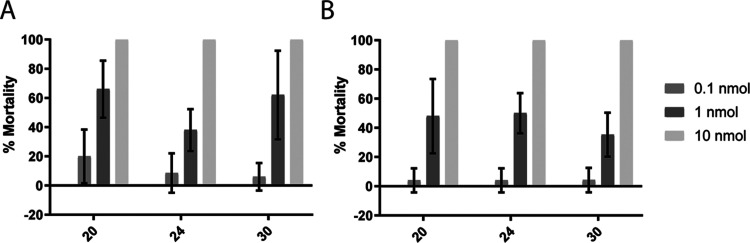
In vivo insecticidal activity of 4-thiazolidinones **20**, **24**, and **30** against *An. gambiae* (A) and *Ae. aegypti* (B) after topical application of 0.1, 1, and 10 nmol substance per mosquito, corresponding to 0.03, 0.3, and 3 μg per mosquito.

Topical application of **20**, **24**, and **30** to *An. gambiae* and *Ae. aegypti* resulted in efficiently killed mosquitoes, causing 100% mortality in both species at a dosage of 3 μg per mosquito (10 nmol/mosquito). The compounds also showed a clear dose–response pattern: insecticidal activity decreased markedly (to approximately 50% mortality) when the dosage was reduced to 0.3 μg per mosquito, and was not detectable when the dosage was reduced further to 0.03 μg (Figure 7). It is interesting that the 4-thiazolidinones were equally effective against both *An. gambiae* and *Ae. aegypti* because previous studies have found that *Ae. aegypti* is less sensitive to AChE1 inhibitors than *Ag. gambiae*.^41,42,58^ The estimated LD_50_ value of 0.3 μg/mosquito for the 4-thiazolidinones is approximately 55 and 170 times higher, respectively, than the LD_50_ values of the currently used insecticides propoxur (LD_50_ = 0.0054 μg/mosquito) and bendiocarb (LD_50_ = 0.0018 μg/mosquito),^58^ but 7 times lower than the those of the previously reported noncovalent phenoxyacetamide-based inhibitors.^42^ Discrepancies between in vitro and in vivo potency for compounds containing aliphatic amine moieties have previously been attributed to poor penetration of the exoskeleton.^42^ However, the agreement between the in vivo and in vitro results for the 4-thiazolidinones was better than that for previously reported insecticidal compounds containing aliphatic amines such as thiourea- and phenoxyacetamide-based AChE1 inhibitors^41,42^ and dopamine receptor antagonists.^59^ Interestingly, the in vivo insecticidal efficacies of the three compounds did not differ greatly despite the pronounced differences in their in vitro potency. A similar lack of correlation between AChE1 inhibition in vitro and mosquito LD_50_ values was previously observed for carbamates.^33^ These findings illustrate the complexity of testing with whole organisms and the importance of physicochemical and pharmacokinetic parameters whose influence is not reflected in the results of enzyme kinetics experiments.

## Conclusions

The 4-thiazolidinones identified through HTS proved to be inhibitors of wild-type AChE1 enzymes from the mosquito species *An. gambiae* and *Ae. aegypti*. The in vitro SARs of these compounds were somewhat restrictive, however, because their inhibitory activity was highly sensitive to even minor modifications of their chemical structures. For example, any change in the structure of the pendant aliphatic tertiary amine moiety reduced inhibitory potency. Studies on compounds having differently substituted phenyl rings showed that the substitution patterns giving the strongest AChE1 inhibition differed from those giving the strongest *h*AChE inhibition, which is promising for avoiding off-organism toxic effects due to unwanted inhibition of human AChE. The problem of increasing insecticide resistance prompted us to test the most promising 4-thiazolidinones against the mutant G122S-*Ag*AChE1 enzyme. This revealed that 4-thiazolidinones can inhibit the mutated *Ag*AChE1, albeit with reduced potency and a different SAR from that seen for the wild-type enzyme. Thus, the challenge to obtain both high selectivity for AChE1 over *h*AChE and strong potency against the resistance-conferring mutant G122S-AgAChE1 needs to be pursued further. Molecular docking studies revealed some possible binding modes of these compounds and provided tentative structural explanations for the observed SAR trends. Notably, the 4-thiazolidinones docked into the lower part of the binding gorge of *Ag*AChE1, which was not the case for *h*AChE and G122S-*Ag*AChE1. In vivo experiments showed that the 4-thiazolidinones killed *An. gambiae* and *Ae. aegypti* mosquitoes. However, relatively high doses were needed and compounds with rather different potencies in vitro had similar insecticidal effects in vivo. This highlights the importance of considering factors in addition to in vitro potency against AChE1 in future efforts to develop novel noncovalent insecticidal agents. It also shows that within a given insecticidal efficacy window there may be several compounds with different selectivity and physicochemical profiles to choose from.

## Experimental Section

### General Chemistry

All commercially available reagents and solvents (except dichloromethane) were purchased from Sigma-Aldrich, Acros, Fluorochem, Fisher Scientific, or VWR and used without further purification. Dichloromethane and DMF were dried in a solvent drying system (Glass Contour Solvent Systems, SG Water). 4 Å molecular sieves were activated at 180 °C in the oven for at least 24 h prior to use. Microwave reactions were performed in sealed glass vials in Biotage Initiator EXP EU and Biotage Initiator+ EU (Biotage Sweden, AB) systems. When necessary, reaction conversion rates were monitored by TLC on aluminum sheets coated with silica gel 60 F_254_ from Merck; spots were visualized by UV detection (254 nm) or by staining with KMnO_4_ solution. Liquid chromatography–mass spectrometry (LC–MS) analyses were performed using a 6130 Quadrupole (Agilent Technologies) mass spectrometer connected to an Agilent 1260 Infinity LC system with an Agilent Proshell 120 EC-C18 2.7 μm 3 × 50 mm column. The eluent was H_2_O/CH_3_CN (0.1% HCOOH) and detection was performed at 210 and 254 nm. When necessary, compounds were purified with a Biotage Isolera One automated flash chromatography system (eluents given in brackets) using KP-SIL 50 μm or Biotage SNAP Ultra 25 μm silica gel disposable cartridges. NMR spectra were acquired on a Bruker DRX 400 or 600 MHz instrument at 298 K unless otherwise stated (Supporting Information, pages S42–S116). The δ values were referenced to the residual solvent signals of CDCl_3_ (7.26 ppm), DMSO-*d*_6_ (2.50 ppm), or CD_3_OD (3.31 ppm) as internal standards for ^1^H and CDCl_3_ (77.16 ppm), DMSO-*d*_6_ (39.52 ppm), or CD_3_OD (49.00 ppm) as internal standards for ^13^C. For ^19^F NMR internal standard of CFCl_3_ was used. All compounds are >95% pure by high-performance liquid chromatography (HPLC) analysis.

### One-Pot Synthesis of Thiazolidinone Scaffold

#### Method A

The corresponding aldehyde (2 mmol) and the corresponding diamine (2 mmol) were added to a 100 mL round-bottom flask containing dry toluene (20 mL) and equipped with a magnetic bar, a reflux condenser, and 4 Å molecular sieves. The mixture was refluxed for 4 h, then allowed to cool down, then mercaptoacetic acid (6 mmol) was added while stirring, and the mixture was refluxed for 3 extra hours, and then allowed to cool down to 25 °C. The organic solution was washed three times with an aqueous saturated NaHCO_3_ solution (20 mL each), dried over Na_2_SO_4_, filtered, and solvent was removed under vacuum to afford a crude oil. Purification proceeded as detailed individually for each compound.

#### Method B^60^

In a microwave-suitable glass vial equipped with a magnetic bar and previously activated 4 Å molecular sieves, the corresponding amine **3** (2.0 mmol) was solved in absolute ethanol (99%, 4.5 mL) and the corresponding aldehyde **1** (4.0 mmol) and mercaptoacetic acid **2** (6.0 mmol) were added, the vial sealed with a suitable cap, and the reaction mixture heated up to 120 °C by microwave irradiation and stirred at this temperature for 30 min. Then, the reaction mixture was allowed to cool down, remaining pressure excess released with a needle, and the reaction mixture was diluted with 50 mL of EtOAc, washed with 2 M NaOH aqueous solution (2 × 10 mL), distilled water (10 mL), and brine (20 mL). The organic phase was collected, dried over Na_2_SO_4_, and concentrated under vacuum to afford an oily crude containing unreacted aldehyde and the desired 4-thiazolidinone. Purification proceeded as detailed individually for each compound.

#### Method C^60^

In a microwave-suitable glass vial equipped with a magnetic bar and previously activated 4 Å molecular sieves, the corresponding amine **3** (2.0 mmol) was solved in absolute ethanol (99%, 4.5 mL) and the corresponding aldehyde **1** (4.0 mmol), mercaptoacetic acid **2** (6.0 mmol), and diisopropylethylamine (2.0 mmol) were added, the vial sealed with a suitable cap, and the reaction mixture heated up to 120 °C by microwave irradiation and stirred at this temperature for 30 min. Then, the reaction mixture was allowed to cool down, remaining pressure excess released with a needle, and the reaction mixture was diluted with 50 mL of EtOAc, washed with 2 M NaOH aqueous solution (2 × 10 mL), distilled water (10 mL), and brine (20 mL). The organic phase was collected, dried over Na_2_SO_4_, and concentrated under vacuum to afford an oily crude containing unreacted aldehyde and the desired 4-thiazolidinone. Purification proceeded as detailed individually for each compound.

### Conversion of Alcohols into Bromines

#### Method D

Following a modified experimental from the literature.^61^ The corresponding alcohol (1.5 mmol) was dissolved in 5 mL of CH_2_Cl_2_ and cooled down to 0 °C. Triphenylphosphine (1.1 equiv) was added, followed by the addition of CBr_4_ (1.1 equiv), and the mixture was stirred at 0 °C. After 2 h of reaction time, TLC indicated full conversion. Then, the reaction mixture was concentrated in vacuo and the residue was purified by automated flash chromatography to afford the corresponding bromine. The bromine was used immediately for the next reaction due to suspected decomposition.

### Intermediates

#### 3-(2-Hydroxyethyl)-2-phenylthiazolidin-4-one (**11**)

Synthesized following the Method A section in a 10 mmol scale. Crude purified by automated flash column chromatography using KP-Sil cartridge and an eluent gradient of Heptane/EtOAc (50:50 to 0:100) to afford 1.4 g of a colorless viscous oil (63% yield). Characterization data in accordance with previously reported in the literature.^62^

#### 3-(3-Bromopropyl)-2-phenylthiazolidin-4-one (**12**)

Synthesized following the Method D section. Crude purified by automated flash column chromatography using KP-Sil cartridge and an eluent gradient of Heptane/EtOAc (100:0 to 50:50) to afford 333 mg of a pale-yellow oil (74% yield). Used immediately due to suspected instability. ^1^H NMR (400 MHz, Chloroform-*d*) δ 7.46–7.33 (m, 3H), 7.32–7.27 (m, 2H), 5.62 (d, *J* = 2.0 Hz, 1H) (N-CH-S), 3.80 (dd, *J* = 15.5, 2.0 Hz, 1H) (S-CH_a_-CO), 3.73–3.56 (m, 2H) (S-CH_b_-CO + Br-CH_a_-), 3.40–3.16 (m, 2H) (Br-CH_2_-CH_2_-), 2.91 (ddd, *J* = 13.9, 7.8, 5.9 Hz, 1H) (Br-CH_b_-), 2.07 (ddq, *J* = 14.3, 8.1, 6.2 Hz, 1H) (N-CH_b_), 1.99–1.83 (m, 1H) (N-CH_a_).

#### 3-(2-Bromoethyl)-2-phenylthiazolidin-4-one (**13**)

Synthesized following the Method D section. Crude purified by automated flash column chromatography using KP-Sil cartridge and an eluent gradient of Heptane/EtOAc (100:0 to 50:50) to afford 386 mg of a pale-yellow oil (90% yield). Used immediately due to suspected instability. ^1^H NMR (400 MHz, Chloroform-*d*) δ 7.45–7.36 (m, 3H), 7.34–7.30 (m, 2H), 5.83 (d, *J* = 1.8 Hz, 1H) (N-CH-S), 3.99 (dt, *J* = 14.4, 6.1 Hz, 1H) (N-CH_a_-CH_2_), 3.84–3.66 (m, 2H), 3.51 (dt, *J* = 10.3, 6.9 Hz, 1H) (Br-CH_b_-CH_2_), 3.26 (dt, *J* = 10.3, 6.0 Hz, 1H) (Br-CH_a_-CH_2_), 3.13 (dt, *J* = 14.1, 6.9 Hz, 1H) (N-CH_b_-CH_2_).

#### 2-(2,4-Dichlorophenyl)-3-(2-hydroxyethyl)thiazolidin-4-one (**44**)

Synthesized following the Method C section. Crude purified by automated flash column chromatography using KP-Sil cartridge and an eluent gradient of Heptane/EtOAc (50:50 to 0:100) to afford 584 mg of a pale-yellow viscous oil (86% yield). ^1^H NMR (400 MHz, Chloroform-*d*) δ 7.44 (d, *J* = 2.1 Hz, 1H), 7.31 (dd, *J* = 8.4, 2.1 Hz, 1H), 7.20 (d, *J* = 8.4 Hz, 1H), 6.20 (d, *J* = 1.8 Hz, 1H), 3.86–3.73 (m, 4H), 3.69 (d, *J* = 15.7 Hz, 1H), 2.98 (ddd, *J* = 14.2, 6.4, 4.1 Hz, 1H).

#### 3-(2-Bromoethyl)-2-(2,4-dichlorophenyl)thiazolidin-4-one (**45**)

Synthesized following the Method D section using 2.4 mmol of **44** as limiting reagent. Crude purified by automated flash column chromatography using KP-Sil cartridge and an eluent gradient of Heptane/EtOAc (100:0 to 50:50) to afford 700 mg of a colorless oil (82% yield). Used immediately due to suspected instability. ^1^H NMR (400 MHz, Chloroform-*d*) δ 7.45 (d, *J* = 2.0 Hz, 1H), 7.31 (dd, *J* = 8.4, 2.1 Hz, 1H), 7.15 (d, *J* = 8.4 Hz, 1H), 6.23 (d, *J* = 1.5 Hz, 1H), 4.15 (dt, *J* = 14.6, 6.2 Hz, 1H), 3.77–3.72 (m, 1H), 3.70 (d, *J* = 15.7 Hz, 1H), 3.55 (dt, *J* = 10.4, 6.9 Hz, 1H), 3.38 (dt, *J* = 10.3, 6.0 Hz, 1H), 3.07 (dt, *J* = 14.1, 6.8 Hz, 1H) ppm.

#### 2-(4-Fluorophenyl)-3-(2-hydroxyethyl)thiazolidin-4-one (**47**)

Synthesized following the Method C section. Crude purified by automated flash column chromatography using KP-Sil cartridge and an eluent gradient of Heptane/EtOAc (50:50 to 0:100) to afford 1538 mg of a white solid (80% yield). ^1^H NMR (600 MHz, Chloroform-*d*) δ 7.38–7.30 (m, 2H), 7.14–7.01 (m, 2H), 5.79 (s, 1H), 3.81 (dd, *J* = 15.7, 2.1 Hz, 1H), 3.74 (d, *J* = 15.7 Hz, 1H), 3.71–3.58 (m, 3H), 3.05–2.87 (m, 1H) ppm. ^13^C NMR (151 MHz, CDCl3) δ 172.7, 163.20 (d, *J* = 249.1 Hz), 135.05 (d, *J* = 3.2 Hz), 129.38 (d, *J* = 8.6 Hz), 116.28 (d, *J* = 21.9 Hz), 64.2, 60.8, 45.9, 33.0 ppm.

#### 3-(2-Bromoethyl)-2-(4-fluorophenyl)thiazolidin-4-one (**48**)

Synthesized following the Method D section using 2.4 mmol of **44** as limiting reagent. Crude purified by automated flash column chromatography using KP-Sil cartridge and an eluent gradient of Heptane/EtOAc (100:0 to 50:50) to afford 400 mg of a colorless oil (80% yield). Used immediately due to suspected instability. ^1^H NMR (400 MHz, Chloroform-*d*) δ 7.38–7.29 (m, 2H), 7.17–6.98 (m, 2H), 5.85 (s, 1H), 3.99 (dt, *J* = 14.6, 5.9 Hz, 1H), 3.84–3.71 (m, 2H), 3.52 (ddd, *J* = 10.4, 7.5, 6.3 Hz, 1H), 3.28 (ddd, *J* = 10.4, 6.2, 5.5 Hz, 1H), 3.10 (ddd, *J* = 14.1, 7.5, 6.2 Hz, 1H) ppm. ^19^F NMR (376 MHz, CDCl_3_) δ −111.3 ppm.

### Final Compounds

#### 3-(3-(Dimethylamino)propyl)-2-phenylthiazolidin-4-one Hydrochloride (**4**)

Synthesized following the Method A section. Purified by dissolution of the crude in 10 mL of ethyl acetate and precipitation by the dropwise addition of HCl in Et_2_O (1 M, 2 mL) while stirring. The solid was filtered, washed with Et_2_O, and collected to afford 312 mg of a white powder (52% yield). HPLC purity 95.7%. ^1^H NMR (400 MHz, Methanol-*d*4) δ 7.60–7.21 (m, 5H), 5.88 (d, *J* = 1.9 Hz, 1H), 3.89 (dd, *J* = 15.7, 1.9 Hz, 1H), 3.78 (d, *J* = 15.7 Hz, 1H), 3.54 (dt, *J* = 14.2, 7.1 Hz, 1H), 3.16–2.95 (m, 3H), 1.94–1.68 (m, 2H) ppm; ^13^C NMR (100 MHz, Methanol-*d*4) δ 174.6, 140.7, 130.5, 130.2, 128.7, 65.1, 56.3, 43.6, 43.4, 41.1, 33.4, 23.6 ppm. HRMS *m*/*z* [M + H]^+^ calcd 299.0980, found 299.0987.

#### 3-(2-(Dimethylamino)ethyl)-2-phenylthiazolidin-4-one Hydrochloride (**5**)

Synthesized following the Method A section. Purified by dissolution of the crude in 10 mL of ethyl acetate and precipitation by the dropwise addition of HCl in Et_2_O (1 M, 2 mL) while stirring. The solid was filtered, washed with Et_2_O, and collected to afford 572 mg of a white powder (85% yield). ^1^H NMR (400 MHz, *d*_6_-DMSO) δ 10.82 (br s, 1H), 7.50–7.35 (m, 5H), 6.02 (s, 1H), 3.97–3.88 (m, 1H), 3.82 (s, 2H), 3.27–3.17 (m, 1H), 3.11–3.02 (m, 1H), 2.80 (dt, *J* = 15.0, 5.0 Hz, 1H), 2.72 (s, 6H) ppm; ^13^C NMR (100 MHz, *d*_6_-DMSO) δ 171.6, 139.2, 129.1, 129.0, 127.5, 61.5, 52.5, 42.8, 41.5, 37.1, 32.2 ppm. HRMS *m*/*z* [M + H]^+^ calcd 251.1213, found 251.1215.

#### 3-(3-(Diethylamino)propyl)-2-phenylthiazolidin-4-one Hydrochloride (**6**)

Synthesized following the Method A section. Purified by dissolution of the crude in 10 mL of ethyl acetate and precipitation by the dropwise addition of HCl in Et_2_O (1 M, 2 mL) while stirring. The solid was filtered, washed with Et_2_O, and collected to afford 352 mg of a white powder (54% yield). ^1^H NMR (400 MHz, *d*_6_-DMSO) δ 10.72 (br s, 1H), 7.46–7.34 (m, 5H), 5.96 (d, *J* = 1.5 Hz, 1H), 3.88 (dd, *J* = 15.5, 1.5 Hz, 1H), 3.72 (d, *J* = 15.5 Hz, 1H), 3.60–3.50 (m, 1H), 3.07–2.95 (m, 4H), 2.92–2.82 (m, 2H), 2.71–2.61 (m, 1H), 1.87–1.74 (m, 2H), 1.17 (t, *J* = 7.2 Hz, 3H), 1.15 (t, *J* = 7.2 Hz, 3H) ppm; ^13^C NMR (100 MHz, *d*_6_-DMSO) δ 171.0, 140.0, 128.9, 128.9, 127.1, 61.7, 47.8, 46.1, 45.3, 39.6, 31.9, 20.8, 8.4, 8.1 ppm. HRMS *m*/*z* [M + H]^+^ calcd 293.1682, found 293.1677.

#### 3-(2-(Diethylamino)ethyl)-2-phenylthiazolidin-4-one Hydrochloride (**7**)

Synthesized following the Method A section. Purified by dissolution of the crude in 10 mL of ethyl acetate and precipitation by the dropwise addition of HCl in Et_2_O (1 M, 2 mL) while stirring. The solid was filtered, washed with Et_2_O, and collected to afford 479 mg of a white powder (76% yield). ^1^H NMR (400 MHz, *d*_6_-DMSO) δ 10.81 (br s, 1H), 7.49–7.35 (m, 5H), 5.99 (d, *J* = 1.5 Hz, 1H), 3.87 (dd, *J* = 15.5, 1.7 Hz, 1H), 3.84–3.75 (m, 1H), 3.76 (d, *J* = 15.5 Hz, 1H), 3.18–3.09 (m, 1H), 3.09–3.00 (m, 4H), 3.00–2.87 (m, 2H), 1.14 (t, *J* = 7.2 Hz, 3H), 1.13 (t, *J* = 7.2 Hz, 3H) ppm; ^13^C NMR (100 MHz, *d*_6_-DMSO) δ 171.4, 139.5, 129.1, 129.0, 127.4, 61.9, 46.4, 46.2, 46.0, 36.9, 31.9, 8.2, 8.1 ppm. HRMS *m*/*z* [M + H]^+^ calcd 279.1526, found 279.1530.

#### 3-(3-Morpholinopropyl)-2-phenylthiazolidin-4-one (**8**)

Synthesized following the Method A section. Purified by dissolution of the crude in 10 mL of ethyl acetate and precipitation by the dropwise addition of HCl in Et_2_O (1 M, 2 mL) while stirring. The expected precipitate was not formed. Solvent evaporated to give an oil. The oil was diluted with 1 M aq HCl and washed twice with Et_2_O. Aqueous phase was basified by dropwise addition of NaOH (aq 2 M), then extracted with dichloromethane, and the organic phase was dried over Na_2_SO_4_ and evaporated under vacuum to afford 338 mg a yellow oil that solidified over time (55% yield). ^1^H NMR (400 MHz, CDCl_3_) δ 7.43–7.31 (m, 5H), 5.84 (d, *J* = 1.8 Hz, 1H), 3.85 (dd, *J* = 15.5, 1.8 Hz, 1H), 3.67 (d, *J* = 15.5 Hz, 1H), 3.53–3.41 (m, 5H), 2.70–2.60 (m, 1H), 2.26–2.07 (m, 6H), 1.68–1.54 (m, 1H), 1.48–1.35 (m, 1H) ppm; ^13^C NMR (100 MHz, CDCl_3_) δ 170.5, 140.4, 128.8, 128.7, 127.1, 66.1, 62.2, 55.2, 53.0, 40.7, 32.0, 23.1 ppm. HRMS *m*/*z* [M + H]^+^ calcd 307.1475, found 307.1475.

#### 3-(4-Fluorophenethyl)-2-phenylthiazolidin-4-one (**9**)

Synthesized following the Method A section. Purified by automated liquid chromatography using KP-Sil 50 g cartridge and Heptane/EtOAc gradient (100:0 to 0:100) as eluent. Product was obtained as 405 mg of a colorless oil (67% yield). ^1^H NMR (400 MHz, CDCl_3_) δ 7.39–7.30 (m, 3H), 7.25–7.20 (m, 2H), 7.06–6.99 (m, 2H), 6.99–6.91 (m, 2H), 5.34 (d, *J* = 1.8 Hz, 1H), 3.86–3.79 (m, 1H), 3.77 (dd, *J* = 15.6, 2.0 Hz, 1H), 3.64 (d, *J* = 15.6 Hz, 1H), 2.94–2.76 (m, 2H), 2.67–2.56 (m, 1H) ppm; ^13^C NMR (100 MHz, CDCl_3_) δ 171.1, 161.7 (d, *J* = 245 Hz), 139.2, 134.0 (d, *J* = 3 Hz), 130.1 (d, *J* = 8 Hz), 129.2, 129.1, 127.1, 115.4 (d, *J* = 21 Hz), 63.9, 44.5, 32.8, 32.5 ppm; ^19^F NMR (376 MHz, CDCl_3_) δ −116.9 ppm. HRMS *m*/*z* [M + H]^+^ calcd 302.1010, found 302.0994.

#### 3-(3-Hydroxypropyl)-2-phenylthiazolidin-4-one (**10**)

Synthesized following the Method C section. Purified by automated liquid chromatography using Biotage Sfär Duo 25 g cartridge and Heptane/EtOAc gradient (50:50 to 0:100) as eluent. Product was obtained as 468 mg of a colorless oil (99% yield). ^1^H NMR (400 MHz, CDCl_3_) δ 7.41–7.34 (m, 3H), 7.34–7.28 (m, 2H), 3.84 (dd, *J* = 15.7, 2.2 Hz, 1H), 3.72 (d, *J* = 15.7 Hz, 1H), 3.69–3.61 (m, 1H), 3.59–3.52 (m, 1H), 3.50–3.42 (m, 1H), 3.12 (br s, 1H), 3.02–2.93 (m, 1H), 1.62–1.51 (m, 1H), 1.51–1.40 (m, 1H) ppm; ^13^C NMR (100 MHz, CDCl_3_) δ 172.5, 139.1, 129.5, 129.2, 127.3, 64.3, 58.5, 39.6, 33.0, 29.6 ppm. Spectral data matches with previously reported in the literature.

#### 3-(3-(1*H*-Imidazol-1-yl)propyl)-2-phenylthiazolidin-4-one (**14**)

NaH (0.40 mmol) was stirred in 4 mL of dry deaminated DMF, and the mixture cooled down to 0 °C. Then, 1*H*-imidazole (0.40 mmol) was added, and the mixture was stirred at 0 °C for 15 min. Then, a solution of the corresponding alkyl bromide (0.31 mmol) in dry deaminated DMF (1 mL) was added dropwise. The mixture was allowed to warm up to room temperature and stirred overnight. After, the reaction was diluted with EtOAc (15 mL) and washed 3 times with NaHCO_3_ (aq sat., 5 mL each). The organic phase was extracted three times with HCl (aq, 1 M, 5 mL each). The acidic aqueous phase was made basic by the addition of NaOH pellets and extracted three times with EtOAc (5 mL each). The organic phase was dried over Na_2_SO_4_, filtered, and concentrated under vacuum to afford 31 mg of a yellow oil (35% yield). The oil was made solid by trituration in Et_2_O for handling. ^1^H NMR (400 MHz, Chloroform-*d*) δ 7.42–7.34 (m, 4H), 7.30–7.24 (m, 2H), 7.01 (t, *J* = 1.1 Hz, 1H), 6.79 (t, *J* = 1.3 Hz, 1H), 5.50 (d, *J* = 1.9 Hz, 1H), 3.94– 3.83 (m, 2H), 3.82 (dd, *J* = 13.6, 2.1 Hz, 1H), 3.69 (d, *J* = 15.6 Hz, 1H), 3.49 (dt, *J* = 14.5, 7.5 Hz, 1H), 2.90 (ddd, *J* = 13.8, 7.6, 5.9 Hz, 1H), 1.97–1.70 (m, 2H). ^13^C NMR (100 MHz, CDCl_3_) δ 171.6, 139.0, 136.9, 129.6, 129.5, 129.2, 127.1, 118.5, 64.0, 44.5, 40.7, 32.8, 28.5 ppm. HRMS *m*/*z* [M + H]^+^ calcd 288.1165, found 288.1170.

#### 3-(2-(1*H*-Imidazol-1-yl)ethyl)-2-phenylthiazolidin-4-one Hydrochloride (**15**)

NaH (0.40 mmol) was stirred in 4 mL of dry deaminated DMF, and the mixture cooled down to 0 °C. Then, 1*H*-imidazole (0.40 mmol) was added, and the mixture was stirred at 0 °C for 15 min. Then, a solution of the corresponding alkyl bromide (0.31 mmol) in dry deaminated DMF (1 mL) was added dropwise. The mixture was allowed to warm up to room temperature and stirred overnight. After, the reaction was diluted with EtOAc (15 mL) and washed 3 times with NaHCO_3_ (aq sat., 5 mL each). The organic phase was extracted three times with HCl (aq, 1 M, 5 mL each). The acidic aqueous phase was made basic by the addition of NaOH pellets and extracted three times with EtOAc (5 mL each). The organic phase was dried over Na_2_SO_4_, filtered, and concentrated under vacuum. Purified by dissolution of the crude in 1 mL of ethyl acetate and precipitation by the dropwise addition of HCl in Et_2_O (1 M, 0.5 mL) while stirring. The solid was filtered to afford the product as white crystals (24 mg, 25% yield). ^1^H NMR (600 MHz, DMSO-*d*_6_) δ 9.16 (s, 1H), 7.73–7.69 (m, 1H), 7.66–7.63 (m, 1H), 7.44–7.32 (m, 6H), 5.90 (d, *J* = 1.7 Hz, 1H), 4.41 (ddd, *J* = 13.8, 9.3, 4.1 Hz, 1H), 4.26 (dt, *J* = 14.0, 4.3 Hz, 1H), 3.98 (ddd, *J* = 14.2, 9.4, 4.4 Hz, 1H), 3.81 (dd, *J* = 15.7, 1.8 Hz, 1H), 3.57 (d, *J* = 15.7 Hz, 1H), 2.98 (dt, *J* = 14.8, 4.3 Hz, 1H) ppm. ^13^C NMR (151 MHz, DMSO) δ 171.4, 139.7, 135.7, 129.1, 129.0, 126.9, 122.3, 119.9, 61.4, 45.6, 42.4, 31.4 ppm. HRMS *m*/*z* [M + H]^+^ calcd 274.1009, found 274.1007.

#### 3-(2-(1*H*-1,2,4-triazol-1-yl)ethyl)-2-phenylthiazolidin-4-one (**16**)

NaH (0.40 mmol) was stirred in 4 mL of dry deaminated DMF, and the mixture cooled down to 0 °C. Then, 1,2,4-triazole (0.40 mmol) was added, and the mixture was stirred at 0 °C for 15 min. Then, a solution of the corresponding alkyl bromide (0.31 mmol) in dry deaminated DMF (1 mL) was added dropwise. The mixture was allowed to warm up to room temperature and stirred overnight. After, the reaction was diluted with EtOAc (15 mL) and washed 3 times with NaHCO_3_ (aq sat., 5 mL each). The organic phase was extracted three times with HCl (aq, 1 M, 5 mL each). The acidic aqueous phase was made basic by the addition of NaOH pellets and extracted 3 times with EtOAc (5 mL each). The organic phase was dried over Na_2_SO_4_, filtered, and concentrated under vacuum. Purified by trituration in Et_2_O to afford a pale-yellow solid (47 mg, 44% yield). ^1^H NMR (400 MHz, Chloroform-*d*) δ 8.04 (bs, 1H), 7.98 (bs, 1H), 7.42–7.33 (m, 3H), 7.21–7.17 (m, 2H), 5.11 (d, *J* = 1.9 Hz, 1H), 4.44 (ddd, *J* = 13.8, 8.4, 5.3 Hz, 1H), 4.18 (dt, *J* = 14.0, 5.0 Hz, 1H), 3.87 (dt, *J* = 14.4, 5.1 Hz, 1H), 3.76 (dd, *J* = 15.7, 1.9 Hz, 1H), 3.66 (d, *J* = 15.7 Hz, 1H), 3.27 (ddd, *J* = 14.0, 8.4, 5.2 Hz, 1H) ppm. ^13^C NMR (100 MHz, CDCl_3_) δ 172.1, 152.6, 143.8, 138.7, 129.7, 129.4, 127.3, 64.3, 46.6, 43.1, 32.6 ppm. HRMS *m*/*z* [M + H]^+^ calcd 275.0961, found 275.0949.

#### 3-(3-Azidopropyl)-2-phenylthiazolidin-4-one (**17**)

The corresponding alkyl bromide (0.33 mmol) was dissolved in a screw-capped vial with 3 mL of dry deaminated DMF. Then, NaN_3_ (0.50 mmol) and potassium iodide (0.03 mmol) were added. The vial was sealed, and the mixture was heated to 80 °C in an oil bath for 18 h while stirring. The mixture was poured into 15 mL of Et_2_O and washed three times with distilled water (5 mL each). The organic phase was dried over Na_2_SO_4_, filtered, and concentrated under vacuum to afford 72 mg of a yellow oil without further purification (82% yield). ^1^H NMR (400 MHz, CDCl_3_) δ 7.43–7.33 (m, 3H), 7.33–7.28 (m, 2H), 5.61 (d, *J* = 1.8 Hz, 1H), 3.82 (dd, *J* = 15.6, 1.9 Hz, 1H), 3.69 (d, *J* = 15.6 Hz, 1H), 3.67–3.59 (m, 1H), 3.32–3.19 (m, 2H), 2.89–2.79 (m, 1H), 1.79–1.58 (m, 2H) ppm; ^13^C NMR (100 MHz, CDCl_3_) δ 171.5, 139.4, 129.5, 129.3, 127.1, 63.9, 49.0, 40.8, 32.9, 26.4 ppm. HRMS *m*/*z* [M + H]^+^ calcd 263.0961, found 263.0947.

#### 2-(4-Chlorophenyl)-3-(3-(dimethylamino)propyl)thiazolidin-4-one Hydrochloride (**18**)

Synthesized following the Method A section. Purified by dissolution of the crude in 10 mL of ethyl acetate and precipitation by the dropwise addition of HCl in Et_2_O (1 M, 2 mL) while stirring. The solid was filtered, washed with Et_2_O, and collected to afford 572 mg of white crystals (85% yield). HPLC purity 100%. ^1^H NMR (400 MHz, Methanol-*d*_4_) δ 7.58–7.20 (m, 4H), 5.92 (d, *J* = 1.9 Hz, 1H), 3.89 (dd, *J* = 15.8, 1.5 Hz, 1H), 3.78 (d, *J* = 15.7 Hz, 1H), 3.58 (dt, *J* = 14.1, 6.9 Hz, 1H), 3.21–3.01 (m, 2H), 2.95 (dt, *J* = 13.5, 6.1 Hz, 1H), 2.86 (s, 6H), 2.17–1.62 (m, 2H) ppm. ^13^C NMR (100 MHz, MeOD) δ 174.4, 139.7, 136.1, 130.4, 130.3, 64.1, 56.2, 43.6, 43.4, 41.1, 33.3, 23.6 ppm. HRMS *m*/*z* [M + H]^+^ calcd 299.0980, found 299.0987.

#### 2-(3-Chlorophenyl)-3-(3-(dimethylamino)propyl)thiazolidin-4-one Hydrochloride (**19**)

Synthesized following the Method A section. Purified by dissolution of the crude in 10 mL of ethyl acetate and precipitation by the dropwise addition of HCl in Et_2_O (1 M, 2 mL) while stirring. The solid was filtered, washed with Et_2_O, and collected to afford 483 mg of white crystals (72% yield). HPLC purity 99.3%. ^1^H NMR (400 MHz, *d*_6_-DMSO) δ 11.04 (br s, 1H), 7.53 (s, 1H), 7.48–7.38 (m, 3H), 5.99 (s, 1H), 3.90 (d, *J* = 15.5 Hz, 1H), 3.71 (d, *J* = 15.5 Hz, 1H), 3.63–3.52 (m, 1H), 2.99–2.86 (m, 2H), 2.73–2.58 (m, 7H), 1.93–1.74 (m, 2H); ^13^C NMR (100 MHz, *d*_6_-DMSO) δ 171.0, 142.6, 133.5, 130.9, 128.8, 127.0, 125.8, 60.7, 53.5, 42.2, 41.2, 39.4, 31.9, 21.4. HRMS *m*/*z* [M + H]^+^ calcd 299.0980, found 299.0986.

#### 2-(2-Chlorophenyl)-3-(3-(dimethylamino)propyl)thiazolidin-4-one Hydrochloride (**20**)

Synthesized following the Method A section. Purified by dissolution of the crude in 10 mL of ethyl acetate and precipitation by the dropwise addition of HCl in Et_2_O (1 M, 2 mL) while stirring. The solid was filtered, washed with Et_2_O, and collected to afford 494 mg of white crystals (74% yield). HPLC purity 95.8%. ^1^H NMR (400 MHz, DMSO-*d*_6_) δ 10.94 (bs, 1H), 7.56–7.49 (m, 1H), 7.44–7.37 (m, 2H), 7.33–7.27 (m, 1H), 6.16 (d, *J* = 1.5 Hz, 1H), 3.75 (dd, *J* = 15.5, 1.6 Hz, 1H), 3.72–3.60 (m, 2H), 3.03–2.90 (m, 3H), 2.74–2.63 (m, 7H), 1.95–1.84 (m, 2H) ppm. ^13^C NMR (100 MHz, DMSO) δ 171.5, 136.8, 131.9, 130.3, 130.2, 128.0 (x2), 59.1, 53.7, 42.0, 41.5, 40.0, 31.4, 21.7 ppm. HRMS *m*/*z* [M + H]^+^ calcd 299.0980, found 299.0977.

#### 3-(3-(Dimethylamino)propyl)-2-(4-methoxyphenyl)thiazolidin-4-one Hydrochloride (**21**)

Synthesized following the Method A section. Purified by dissolution of the crude in 10 mL of ethyl acetate and precipitation by the dropwise addition of HCl in Et_2_O (1 M, 2 mL) while stirring. The solid was filtered, washed with Et_2_O, and collected to afford 307 mg of white crystals (46% yield). HPLC purity 98.8%. ^1^H NMR (400 MHz, *d*_6_-DMSO) δ 11.07 (br s, 1H), 7.39 (d, *J* = 8.5 Hz, 2H), 6.95 (d, *J* = 8.5 Hz, 2H), 5.91 (s, 1H), 3.82 (d, *J* = 15.4 Hz, 1H), 3.76 (s, 3H), 3.71 (d, *J* = 15.4 Hz, 1H), 3.57–3.45 (m, 1H), 2.97–2.81 (m, 2H), 2.72–2.57 (m, 7H), 1.89–1.69 (m, 2H); ^13^C NMR (100 MHz, *d*_6_-DMSO) δ 170.8, 159.6, 131.1, 128.9, 114.2, 61.4, 55.2, 53.5, 42.2, 41.2, 39.2, 32.1, 21.4. HRMS *m*/*z* [M + H]^+^ calcd 295.1475, found 295.1483.

#### 3-(3-(Dimethylamino)propyl)-2-(3-methoxyphenyl)thiazolidin-4-one Hydrochloride (**22**)

Synthesized following the Method B section. Purified by dissolution of the crude in 10 mL of diethyl ether and 1 mL of MeOH, followed by precipitation by the dropwise addition of HCl in Et_2_O (1 M, 2 mL) while stirring. The solid was filtered, washed with Et_2_O, and collected to afford 592 mg of white crystals (89% yield). HPLC purity 99.4%. ^1^H NMR (400 MHz, Methanol-*d*_4_) δ 7.35 (t, *J* = 7.9 Hz, 1H), 7.05–6.99 (m, 2H), 6.96 (ddd, *J* = 8.3, 2.6, 0.9 Hz, 1H), 5.85 (d, *J* = 1.9 Hz, 1H), 3.88 (ddd, *J* = 15.7, 2.0, 0.6 Hz, 1H), 3.81 (s, 3H), 3.77 (d, *J* = 15.7 Hz, 1H), 3.55 (dt, *J* = 14.4, 7.2 Hz, 1H), 3.16–2.95 (m, 3H), 2.85 (s, 3H), 2.83 (s, 3H), 1.93–1.72 (m, 2H) ppm. ^13^C NMR (100 MHz, MeOD) δ 174.64, 161.73, 142.23, 131.39, 120.64, 115.77, 114.24, 65.01, 56.30, 55.88, 43.61, 43.32, 41.13, 33.35, 23.67 ppm. HRMS *m*/*z* [M + H]^+^ calcd 295.1475, found 295.1479.

#### 3-(3-(Dimethylamino)propyl)-2-(2-methoxyphenyl)thiazolidin-4-one Hydrochloride (**23**)

Synthesized following the Method A section. Purified by the dissolution of the crude in 10 mL of ethyl acetate, followed by precipitation by the dropwise addition of HCl in Et_2_O (1 M, 2 mL) while stirring. The solid was filtered, washed with Et_2_O, and collected to afford 291 mg of white crystals (44% yield). HPLC purity 97.5%. ^1^H NMR (400 MHz, *d*_6_-DMSO) δ 10.90 (br s, 1H), 7.35 (dt, *J* = 1.5, 8.2 Hz, 1H), 7.17 (dt, *J* = 1.5, 7.5 Hz, 1H), 7.08 (d, *J* = 8.2 Hz, 1H), 6.97 (t, *J* = 7.5 Hz, 1H), 6.04 (s, 1H), 3.83 (s, 3H), 3.71 (d, *J* = 15.4 Hz, 1H), 3.61 (d, *J* = 15.4 Hz, 1H), 3.65–3.54 (m, 1H), 3.00–2.86 (m, 2H), 2.71–2.61 (m, 7H), 1.90–1.78 (m, 2H); ^13^C NMR (100 MHz, *d*_6_-DMSO) δ 171.5, 156.8, 129.9, 127.3, 126.8, 120.6, 111.6, 57.3, 55.8, 53.8, 42.0, 41.6, 39.8, 31.8, 21.6 ppm. HRMS *m*/*z* [M + H]^+^ calcd 295.1475, found 295.1476.

#### 3-(3-(Dimethylamino)propyl)-2-(4-fluorophenyl)thiazolidin-4-one Hydrochloride (**24**)

Synthesized following the Method A section. Purified by dissolution of the crude in 3 mL of ethyl acetate and 10 mL of diethyl ether, followed by precipitation by the dropwise addition of HCl in Et_2_O (1 M, 2 mL) while stirring. The solid was filtered, washed with Et_2_O, and collected to afford 286 mg of white crystals (45% yield). HPLC purity 99.1%. ^1^H NMR (400 MHz, Methanol-*d*_4_) δ 7.57–7.46 (m, 2H), 7.26–7.05 (m, 2H), 5.92 (d, *J* = 1.9 Hz, 1H), 3.89 (dd, *J* = 15.7, 2.0 Hz, 1H), 3.78 (d, *J* = 15.6 Hz, 1H), 3.55 (dt, *J* = 14.4, 7.2 Hz, 1H), 3.08 (ddt, *J* = 14.8, 9.3, 6.7 Hz, 2H), 3.02–2.91 (m, 1H), 2.85 (s, 6H), 1.95–1.67 (m, 2H) ppm. ^13^C NMR (100 MHz, MeOD) δ 174.4, 164.6 (d, *J* = 247.3 Hz), 136.7 (d, *J* = 3.1 Hz), 131.0 (d, *J* = 8.6 Hz), 117.0 (d, *J* = 22.1 Hz), 64.2, 56.2, 43.5, 43.4, 41.0, 33.4, 23.6 ppm. ^19^F NMR (376 MHz, MeOD) δ −114.0 ppm. HRMS *m*/*z* [M + H]^+^ calcd 283.1275, found 283.1287.

#### 3-(3-(Dimethylamino)propyl)-2-(3-fluorophenyl)thiazolidin-4-one Hydrochloride (**25**)

Synthesized following the Method B section. Purified by dissolution of the crude in 1 mL of methanol and 20 mL of diethyl ether, followed by precipitation by the dropwise addition of HCl in Et_2_O (1 M, 2 mL) while stirring. The solid was filtered, washed with diethyl ether, and collected to afford 480 mg of pale-yellow crystals (75% yield). ^1^H NMR (600 MHz, DMSO-*d*_6_) δ 10.54 (s, 1H), 7.46 (td, *J* = 7.9, 5.9 Hz, 1H), 7.33–7.26 (m, 2H), 7.24–7.16 (m, 1H), 5.95 (d, *J* = 1.8 Hz, 1H), 3.91 (dd, *J* = 15.5, 1.9 Hz, 1H), 3.71 (d, *J* = 15.5 Hz, 1H), 3.60–3.52 (m, 1H), 2.99–2.86 (m, 2H), 2.67 (m, 7H), 1.87–1.68 (m, 2H) ppm. ^13^C NMR (151 MHz, DMSO) δ 171.1, 162.3 (d, *J* = 244.8 Hz), 143.1 (d, *J* = 6.9 Hz), 131.1 (d, *J* = 8.2 Hz), 123.2 (d, *J* = 2.7 Hz), 115.8 (d, *J* = 21.1 Hz), 114.0 (d, *J* = 22.3 Hz), 61.0 (d, *J* = 2.0 Hz), 53.7, 42.2, 41.6, 40.1, 31.9, 21.6 ppm. ^19^F NMR (376 MHz, DMSO) δ −111.8 ppm. HRMS *m*/*z* [M + H]^+^ calcd 283.1275, found 283.1275.

#### 3-(3-(Dimethylamino)propyl)-2-(2-fluorophenyl)thiazolidin-4-one Hydrochloride (**26**)

Synthesized following the Method B section. Purified by dissolution of the crude in 1 mL of methanol and 20 mL of diethyl ether, followed by precipitation by the dropwise addition of HCl in Et_2_O (1 M, 2 mL) while stirring. The solid was filtered, washed with diethyl ether, and collected to afford 545 mg of pale-yellow crystals (85% yield). HPLC purity 100%. ^1^H NMR (600 MHz, Methanol-*d*_4_) δ 7.54–7.38 (m, 2H), 7.28–7.24 (m, 1H), 7.21–7.16 (m, 1H), 6.12 (d, *J* = 2.0 Hz, 1H), 3.88 (dd, *J* = 15.7, 2.0 Hz, 1H), 3.75 (dd, *J* = 15.7, 0.9 Hz, 1H), 3.67–3.60 (m, 1H), 3.15–3.06 (m, 2H), 3.00 (dt, *J* = 14.4, 6.3 Hz, 1H), 2.87 (s, 3H), 2.85 (s, 3H), 1.95–1.82 (m, 1H) ppm. ^13^C NMR (151 MHz, MeOD) δ 174.66, 162.12 (d, *J* = 247.7 Hz), 132.34 (d, *J* = 8.6 Hz), 129.95 (d, *J* = 3.0 Hz), 128.02 (d, *J* = 10.9 Hz), 126.17 (d, *J* = 3.7 Hz), 117.27 (d, *J* = 21.4 Hz), 59.31 (d, *J* = 3.7 Hz), 56.35, 43.55, 43.48, 41.23, 33.21, 23.71 ppm. ^19^F NMR (376 MHz, MeOD) δ −120.3 ppm. HRMS *m*/*z* [M + H]^+^ calcd 283.1275, found 283.1278.

#### 3-(3-(Dimethylamino)propyl)-2-(pyridin-4-yl)thiazolidin-4-one (**26**)

Synthesized following a modified the Method A section. Once the reaction was complete, the compound was extracted with a solution of aqueous NaHCO_3_ (3 times, 10 mL each). Then, the aqueous phase was extracted with CH_2_Cl_2_ (3 times, 20 mL each). The organic phases were combined, dried over Na_2_SO_4_, filtered, and concentrated under vacuum to afford 304 mg of a yellow oil. No further purification was needed (57% yield). ^1^H NMR (400 MHz, CDCl_3_) δ 8.56 (dd, *J* = 4.3, 1.6 Hz, 2H), 7.12 (dd, *J* = 4.3, 1.6 Hz, 2H), 5.55 (d, *J* = 1.8 Hz, 1H), 3.73 (dd, *J* = 15.5, 1.9 Hz, 1H), 3.69–3.60 (m, 1H), 3.60 (d, *J* = 15.5 Hz, 1H), 2.70–2.61 (m, 1H), 2.21–2.08 (m, 2H), 2.06 (s, 6H), 1.68–1.56 (m, 1H), 1.56–1.44 (m, 1H) ppm; ^13^C NMR (100 MHz, CDCl_3_) δ 171.1, 150.6, 149.0, 121.2, 62.1, 56.5, 45.2, 41.5, 32.5, 25.0 ppm. HRMS *m*/*z* [M + H]^+^ calcd 266.1322, found 266.1316.

#### 3-(3-(Dimethylamino)propyl)-2-(pyridin-3-yl)thiazolidin-4-one (**28**)

Synthesized following a modified the Method A section. Once the reaction was complete, the compound was extracted with a solution of aqueous NaHCO_3_ (3 times, 10 mL each). Then, the aqueous phase was extracted with CH_2_Cl_2_ (3 times, 20 mL each). The organic phases were combined, dried over Na_2_SO_4_, filtered, and concentrated under vacuum to afford 302 mg of a yellow oil. No further purification was needed (57% yield). ^1^H NMR (400 MHz, Chloroform-*d*) δ 8.51 (dd, *J* = 4.8, 1.6 Hz, 1H), 8.46 (d, *J* = 2.4 Hz, 1H), 7.58 (dt, *J* = 8.0, 2.0 Hz, 1H), 7.27–7.20 (m, 1H), 5.61 (d, *J* = 2.0 Hz, 1H), 3.72 (dd, *J* = 15.5, 2.0 Hz, 1H), 3.61 (d, *J* = 15.4 Hz, 1H), 3.60–3.42 (m, 1H), 2.64 (ddd, *J* = 14.2, 8.4, 6.0 Hz, 1H), 2.19–2.03 (m, 2H), 2.03 (s, 6H), 1.66–1.53 (m, 1H), 1.51–1.38 (m, 1H) ppm. ^13^C NMR (100 MHz, CDCl_3_) δ 170.8, 150.6, 148.6, 135.4, 134.7, 123.9, 61.2, 56.5, 45.2, 41.2, 32.7, 24.9 ppm. HRMS *m*/*z* [M + H]^+^ calcd 266.1322, found 266.1323.

#### 3-(3-(Dimethylamino)propyl)-2-(pyridin-2-yl)thiazolidin-4-one (**29**)

Synthesized following a modified the Method A section. Once the reaction was complete, the compound was extracted with a solution of aqueous NaHCO_3_ (3 times, 10 mL each). Then, the aqueous phase was extracted with CH_2_Cl_2_ (3 times, 20 mL each). The organic phases were combined, dried over Na_2_SO_4_, filtered, and concentrated under vacuum to afford 424 mg of a pale-yellow solid. No further purification was needed (80% yield). ^1^H NMR (400 MHz, CDCl_3_) δ 8.48 (ddd, *J* = 4.9, 1.8, 1.0 Hz, 1H), 7.63 (td, *J* = 7.7, 1.8 Hz, 1H), 7.20–7.14 (m, 2H), 5.63 (d, *J* = 1.9 Hz, 1H), 3.77 (dd, *J* = 15.3, 1.8 Hz, 1H), 3.70–3.60 (m, 1H), 3.53 (d, *J* = 15.3 Hz, 1H), 2.74–2.64 (m, 1H), 2.22–2.07 (m, 2H), 2.05 (s, 6H), 1.69–1.56 (m, 1H), 1.56–1.44 (m, 1H) ppm; ^13^C NMR (100 MHz, CDCl_3_) δ 171.6, 159.4, 149.8, 137.3, 123.4, 120.4, 64.4, 56.7, 45.3, 41.7, 32.5, 25.0 ppm. HRMS *m*/*z* [M + H]^+^ calcd 266.1322, found 266.1318.

#### 2-(2,4-Dichlorophenyl)-3-(3-(dimethylamino)propyl)thiazolidin-4-one Hydrochloride (**30**)

Synthesized following the Method A section. Purified by dissolution of the crude in 10 mL of ethyl acetate, followed by precipitation by the dropwise addition of HCl in Et_2_O (1 M, 2 mL) while stirring. The solid was filtered, washed with Et_2_O, and collected to afford 540 mg of white crystals (73% yield). HPLC purity 99.6%. ^1^H NMR (400 MHz, Methanol-*d*_4_) δ 7.55 (d, *J* = 1.9 Hz, 1H), 7.43 (dd, *J* = 8.4, 2.0 Hz, 1H), 7.38 (d, *J* = 8.4 Hz, 1H), 6.24 (d, *J* = 1.8 Hz, 1H), 3.83 (dd, *J* = 15.8, 1.8 Hz, 1H), 3.78–3.65 (m, 2H), 3.23–3.07 (m, 2H), 3.02–2.90 (m, 1H), 2.89 (s, 6H), 2.04–1.90 (m, 2H) ppm. ^13^C NMR (100 MHz, MeOD) δ 174.8, 137.0, 136.3, 134.8, 131.1, 130.3, 129.3, 61.1, 56.3, 43.5, 41.4, 32.9, 23.7 ppm. HRMS *m*/*z* [M + H]^+^ calcd 333.0590, found 333.0588.

#### 2-(2,4-Difluorophenyl)-3-(3-(dimethylamino)propyl)thiazolidin-4-one Hydrochloride (**31**)

Synthesized following the Method B section. Purified by dissolution of the crude in 1 mL of ethyl acetate and 20 mL of diethyl ether, followed by precipitation by the dropwise addition of HCl in Et_2_O (1 M, 2 mL) while stirring. The solid was filtered, washed with diethyl ether, and collected to afford 552 mg of white crystals (82% yield). HPLC purity 99.8%. ^1^H NMR (600 MHz, Methanol-*d*_4_) δ 7.68–7.34 (m, 1H), 7.22–6.85 (m, 2H), 6.11 (d, *J* = 2.0 Hz, 1H), 3.88 (dd, *J* = 15.7, 2.0 Hz, 1H), 3.75 (dd, *J* = 15.6, 0.9 Hz, 1H), 3.63 (dt, *J* = 14.6, 7.3 Hz, 1H), 3.18–3.05 (m, 2H), 3.02–2.94 (m, 1H), 2.88 (s, 3H), 2.86 (s, 3H), 2.00–1.80 (m, 2H) ppm. ^13^C NMR (151 MHz, Methanol-*d*_4_) δ 174.5, 164.8 (dd, *J* = 250.1, 12.4 Hz), 162.4 (dd, *J* = 250.9, 12.2 Hz), 131.5 (dd, *J* = 10.1, 4.6 Hz), 124.4 (dd, *J* = 11.4, 3.9 Hz), 113.2 (dd, *J* = 21.8, 3.6 Hz), 105.6 (t, *J* = 25.9 Hz), 58.9 (d, *J* = 2.9 Hz), 56.3, 43.6, 43.5, 41.3, 33.2 (d, *J* = 2.2 Hz), 23.7 ppm. ^19^F NMR (376 MHz, Methanol-*d*_4_) δ −110.10 (d, *J* = 8.2 Hz), −115.83 (d, *J* = 8.4 Hz) ppm. HRMS *m*/*z* [M + H]^+^ calcd 301.1181, found 301.1180.

#### 2-(2-Chloro-4-fluorophenyl)-3-(3-(dimethylamino)propyl)thiazolidin-4-one Hydrochloride (**32**)

Synthesized following the Method B section at 1 mmol scale. Purified by dissolution of the crude in 10 mL of diethyl ether, followed by precipitation by the dropwise addition of HCl in Et_2_O (1 M, 1 mL) while stirring. The solid was filtered, washed with diethyl ether, and collected to afford 312 mg of white crystals (88% yield). HPLC purity 99.9%. ^1^H NMR (400 MHz, Methanol-*d*_4_) δ 7.44 (dd, *J* = 8.8, 5.9 Hz, 1H), 7.34 (dd, *J* = 8.5, 2.6 Hz, 1H), 7.20 (td, *J* = 8.4, 2.6 Hz, 1H), 6.25 (d, *J* = 1.9 Hz, 1H), 3.84 (dd, *J* = 15.7, 1.9 Hz, 1H), 3.78–3.57 (m, 2H), 3.13 (ddd, *J* = 9.0, 6.7, 4.1 Hz, 2H), 2.99–2.90 (m, 1H), 2.88 (s, 6H), 2.08–1.81 (m, 2H) ppm. ^13^C NMR (100 MHz, MeOD) δ 174.87, 163.93 (d, *J* = 250.9 Hz), 135.06 (d, *J* = 10.6 Hz), 134.25, 130.97, 118.71 (d, *J* = 25.5 Hz), 116.26 (d, *J* = 21.8 Hz), 61.10, 56.37, 43.54, 41.29, 32.89, 23.76 ppm. ^19^F NMR (376 MHz, MeOD) δ −112.30 ppm. HRMS *m*/*z* [M + H]^+^ calcd 317.0885, found 317.0885.

#### 2-(2,3-Difluorophenyl)-3-(3-(dimethylamino)propyl)thiazolidin-4-one Hydrochloride (**33**)

Synthesized following the Method B section. Purified by dissolution of the crude in 1 mL of ethyl acetate and 20 mL of diethyl ether, followed by precipitation by the dropwise addition of HCl in Et_2_O (1 M, 2 mL) while stirring. The solid was filtered, washed with diethyl ether, and collected to afford 518 mg of white crystals (77% yield). HPLC purity 96.2%. ^1^H NMR (600 MHz, Methanol-*d*_4_) δ 7.40–7.27 (m, 1H), 7.25–7.17 (m, 2H), 6.14 (d, *J* = 1.9 Hz, 1H), 3.88 (dd, *J* = 15.7, 2.0 Hz, 1H), 3.74 (d, *J* = 15.7 Hz, 1H), 3.66 (dt, *J* = 14.6, 7.4 Hz, 1H), 3.18–3.05 (m, 2H), 2.98 (dt, *J* = 14.5, 6.3 Hz, 1H), 2.86 (s, 6H), 1.98–1.86 (m, 2H) ppm. ^13^C NMR (151 MHz, MeOD) δ 173.19, 150.61 (dd, *J* = 248.2, 12.2 Hz), 148.59 (dd, *J* = 249.9, 13.5 Hz), 129.50 (d, *J* = 8.0 Hz), 125.04 (dd, *J* = 7.0, 4.7 Hz), 123.20 (dd, *J* = 3.6, 1.8 Hz), 117.77 (d, *J* = 17.3 Hz), 57.46, 54.96, 42.14, 39.87, 31.68, 31.66, 22.33 ppm. ^19^F NMR (376 MHz, Methanol-*d*_4_) δ −139.84 (d, *J* = 19.8 Hz), −146.25 (d, *J* = 19.7 Hz) ppm. HRMS *m*/*z* [M + H]^+^ calcd 301.1181, found 301.1182.

#### 2-(2,3-Dichlorophenyl)-3-(3-(dimethylamino)propyl)thiazolidin-4-one Hydrochloride (**34**)

Synthesized following the Method B section. Purified by dissolution of the crude in 3 mL of ethanol and 30 mL of diethyl ether, followed by precipitation by the dropwise addition of HCl in Et_2_O (1 M, 2 mL) while stirring. The solid was filtered, washed with diethyl ether, and collected to afford 552 mg of white crystals (75% yield). HPLC purity 99.2%. ^1^H NMR (600 MHz, DMSO-*d*_6_) δ 10.47 (bs, 1H), 7.66 (dd, *J* = 8.0, 1.5 Hz, 1H), 7.44 (t, *J* = 7.9 Hz, 1H), 7.22 (bs, 1H), 6.17 (d, *J* = 1.6 Hz, 1H), 3.79–3.67 (m, 2H), 3.69–3.63 (m, 1H), 3.05–2.90 (m, 2H), 2.76–2.70 (m, 1H), 2.70 (d, *J* = 4.8 Hz, 3H), 2.69 (d, *J* = 4.8 Hz, 3H), 1.99–1.79 (m, 2H) ppm. ^13^C NMR (151 MHz, DMSO) δ 171.77, 139.95, 132.73, 130.43, 129.82, 128.97, 125.46, 59.55, 53.95, 42.22, 41.78, 40.20, 31.18, 21.84 ppm. HRMS *m*/*z* [M + H]^+^ calcd 333.0590, found 333.0591.

#### 2-(Benzo[*d*][1,3]dioxol-5-yl)-3-(3-(dimethylamino)propyl)thiazolidin-4-one Hydrochloride (**35**)

Synthesized following the Method A section. Purified by dissolution of the crude in 10 mL of ethyl acetate, followed by precipitation by the dropwise addition of HCl in Et_2_O (1 M, 2 mL) while stirring. The solid was filtered, washed with Et_2_O, and collected to afford 361 mg of pale-brown crystals (52% yield). ^1^H NMR (400 MHz, *d*_6_-DMSO) δ 10.60 (br s, 1H), 7.02 (s, 1H), 6.95 (d, *J* = 7.9 Hz, 1H), 6.90 (d, *J* = 7.9 Hz, 1H), 6.04 (s, 2H), 5.85 (s, 1H), 3.87 (d, *J* = 15.4 Hz, 1H), 3.69 (d, *J* = 15.4 Hz, 1H), 3.57–3.45 (m, 1H), 2.99–2.83 (m, 2H), 2.76–2.59 (m, 7H), 1.87–1.68 (m, 2H) ppm; ^13^C NMR (100 MHz, *d*_6_-DMSO) δ 170.8, 147.9, 147.8, 133.4, 121.3, 108.1, 107.2, 101.4, 61.7, 53.7, 42.2, 41.4, 39.2, 32.0, 21.5 ppm. HRMS *m*/*z* [M + H]^+^ calcd 309.1268, found 309.1272.

#### 3-(3-(Dimethylamino)propyl)-2-(naphthalen-1-yl)thiazolidin-4-one Hydrochloride (**36**)

Synthesized following the Method A section. Purified by dissolution of the crude in 10 ml of ethyl acetate, followed by precipitation by the dropwise addition of HCl in Et_2_O (1 M, 2 mL) while stirring. The solid was filtered, washed with Et_2_O, and collected to afford 577 mg of white crystals (82% yield). HPLC purity 99.7%. ^1^H NMR (400 MHz, *d*_6_-DMSO) δ 10.93 (br s, 1H), 8.13 (d, *J* = 8.2 Hz, 1H), 8.03–7.98 (m, 1H), 7.94 (d, *J* = 8.0 Hz, 1H), 7.66–7.56 (m, 2H), 7.53 (t, *J* = 7.6 Hz, 1H), 7.41–7.09 (m, 1H), 6.96–6.62 (m, 1H), 3.91–3.55 (m, 3H), 3.06–2.87 (m, 2H), 2.87–2.73 (m, 1H), 2.73–2.55 (m, 6H), 2.11–1.72 (m, 2H) ppm; ^13^C NMR (150 MHz, *d*_6_-DMSO) δ 171.9, 135.6, 133.7, 129.8, 128.9, 128.5, 126.7, 126.2, 125.6, 122.8, 121.5, 57.7, 53.7, 42.2, 41.4, 40.2, 31.5, 21.8 ppm. HRMS *m*/*z* [M + H]^+^ calcd 315.1526, found 315.1518.

#### 3-(2-(Dimethylamino)ethyl)-2-(4-fluorophenyl)thiazolidin-4-one Hydrochloride (**37**)

Synthesized following the Method B section. Purified by dissolution of the crude in 1 mL of ethyl acetate and 20 mL of diethyl ether, followed by precipitation by the dropwise addition of HCl in Et_2_O (1 M, 2 mL) while stirring. The solid was filtered, washed with diethyl ether, and collected to afford 580 mg of white crystals (95% yield). ^1^H NMR (600 MHz, DMSO-*d*_6_) δ 10.46 (bs, 1H), 7.54 (dd, *J* = 8.6, 5.5 Hz, 2H), 7.25 (t, *J* = 8.8 Hz, 2H), 6.00 (s, 1H), 3.90 (ddd, *J* = 15.1, 9.0, 6.0 Hz, 1H), 3.86–3.74 (m, 2H), 3.25–3.20 (m, 1H), 3.07–3.01 (m, 1H), 2.80 (dt, *J* = 15.2, 5.2 Hz, 1H), 2.74 (s, 6H) ppm. ^13^C NMR (151 MHz, DMSO) δ 171.53, 162.27 (d, *J* = 245.7 Hz), 135.47 (d, *J* = 3.1 Hz), 129.86 (d, *J* = 8.6 Hz), 115.85 (d, *J* = 21.7 Hz), 60.72, 52.64, 42.91, 41.60, 37.04, 32.16 ppm. ^19^F NMR (376 MHz, DMSO) δ −112.1 ppm. HRMS *m*/*z* [M + H]^+^ calcd 269.1119, found 269.1120.

#### 2-(2,4-Dichlorophenyl)-3-(2-(dimethylamino)ethyl)thiazolidin-4-one Hydrochloride (**38**)

Synthesized following the Method B section. Purified by dissolution of the crude in 5 mL of ethyl acetate and 25 mL of diethyl ether, followed by precipitation by the dropwise addition of HCl in Et_2_O (1 M, 2 mL) while stirring. The solid was filtered, washed with diethyl ether, and collected to afford 615 mg of white crystals (86% yield). ^1^H NMR (600 MHz, Methanol-*d*_4_) δ 7.59 (t, *J* = 1.6 Hz, 1H), 7.45 (dt, *J* = 8.3, 1.8 Hz, 1H), 7.35 (d, *J* = 8.4 Hz, 1H), 6.25 (s, 1H), 4.06 (ddd, *J* = 14.7, 8.9, 5.1 Hz, 1H), 3.89–3.79 (m, 1H), 3.73 (d, *J* = 15.9 Hz, 1H), 3.43 (ddd, *J* = 13.8, 8.8, 5.2 Hz, 1H), 3.22 (dt, *J* = 13.4, 5.0 Hz, 1H), 3.13 (dd, *J* = 15.4, 5.1 Hz, 1H), 2.99 (s, 3H), 2.95 (s, 3H) ppm. ^13^C NMR (100 MHz, MeOD) δ 175.6, 136.6, 136.5, 134.9, 131.3, 130.1, 129.4, 60.9, 56.2, 44.8, 43.3, 39.7, 32.6 ppm. HRMS *m*/*z* [M + H]^+^ calcd 319.0433, found 319.0434.

#### 2-(2,3-Difluorophenyl)-3-(2-(dimethylamino)ethyl)thiazolidin-4-one Hydrochloride (**39**)

Synthesized following the Method B section. Purified by dissolution of the crude in 5 mL of ethyl acetate and 25 mL of diethyl ether, followed by precipitation by the dropwise addition of HCl in Et_2_O (1 M, 2 mL) while stirring. The solid was filtered, washed with diethyl ether, and collected to afford 638 mg of white crystals (99% yield). ^1^H NMR (600 MHz, DMSO-*d*_6_) δ 10.72 (bs, 1H), 7.47 (ddt, *J* = 10.4, 7.8, 3.8 Hz, 1H), 7.29–7.19 (m, 2H), 6.27 (s, 1H), 4.00 (ddd, *J* = 15.1, 8.5, 6.6 Hz, 1H), 3.86–3.63 (m, 2H), 3.30–3.23 (m, 1H), 3.18–3.12 (m, 1H), 2.96 (ddd, *J* = 15.0, 6.5, 4.3 Hz, 1H), 2.74 (s, 6H) ppm. ^13^C NMR (151 MHz, DMSO) δ 172.13, 150.36 (dd, *J* = 246.6, 11.9 Hz), 148.33 (dd, *J* = 249.8, 13.4 Hz), 129.83 (d, *J* = 7.4 Hz), 125.90 (dd, *J* = 7.2, 4.4 Hz), 124.12 (bs), 118.48 (d, *J* = 17.1 Hz), 56.52, 53.07, 43.17, 42.16, 37.90, 32.23 ppm. ^19^F NMR (376 MHz, DMSO-*d*_6_) δ −137.87 (d, *J* = 21.2 Hz), −144.36 (d, *J* = 21.5 Hz) ppm. HRMS *m*/*z* [M + H]^+^ calcd 287.1024, found 287.1024.

#### 3-(2-(Dimethylamino)ethyl)-2-(naphthalen-1-yl)thiazolidin-4-one Hydrochloride (**40**)

Synthesized following the Method B section. Purified by dissolution of the crude in 5 mL of ethyl acetate and 25 mL of diethyl ether, followed by precipitation by the dropwise addition of HCl in Et_2_O (1 M, 2 mL) while stirring. The solid was filtered, washed with diethyl ether, and collected to afford 556 mg of pale-yellow crystals (83% yield). HPLC purity 100%. ^1^H NMR (400 MHz, Methanol-*d*_4_) δ 8.12 (d, *J* = 8.4 Hz, 1H), 8.00–7.86 (m, 2H), 7.66–7.49 (m, 3H), 7.47–7.41 (m, 1H), 6.80 (bs, 1H), 4.13–4.00 (m, 1H), 3.95–3.71 (m, 2H), 3.45–3.34 (m, 1H), 3.28–3.09 (m, 2H), 2.91 (s, 6H) ppm. ^13^C NMR (151 MHz, MeOD) δ 176.0, 135.7, 131.7, 130.3, 128.0, 127.4, 126.5, 123.3, 60.0, 56.3, 44.1, 40.1, 32.8 ppm. HRMS *m*/*z* [M + H]^+^ calcd 301.1369, found 301.1369.

#### 2-(2,4-Dichlorophenyl)-3-(2-(isopropylamino)ethyl)thiazolidin-4-one Hydrochloride (**41**)

Synthesized following the Method C section. Purified by dissolution of the crude in 5 mL of ethyl acetate and 15 mL of diethyl ether, followed by precipitation by the dropwise addition of HCl in Et_2_O (1 M, 2 mL) while stirring. The solid was filtered, washed with diethyl ether, and collected to afford 335 mg of white crystals (45% yield). HPLC purity 99.8%. ^1^H NMR (400 MHz, DMSO-*d*_6_) δ 9.10 (s, 2H), 7.71 (d, *J* = 2.1 Hz, 1H), 7.50 (dd, *J* = 8.4, 2.2 Hz, 1H), 7.35 (d, *J* = 8.4 Hz, 1H), 6.25 (s, 1H), 3.89 (dt, *J* = 12.6, 6.0 Hz, 1H), 3.76–3.67 (m, 2H), 3.26 (p, *J* = 6.5 Hz, 1H), 3.15–3.05 (m, 1H), 2.96 (ddt, *J* = 18.9, 11.6, 7.0 Hz, 2H), 1.22 (d, *J* = 1.8 Hz, 3H), 1.20 (d, *J* = 1.7 Hz, 3H) ppm. ^13^C NMR (100 MHz, DMSO) δ 172.0, 135.9, 133.8, 132.9, 129.8, 128.1, 58.9, 49.4, 40.4, 39.6, 31.4, 18.45, 18.39 ppm. HRMS *m*/*z* [M + H]^+^ calcd 333.0590, found 333.0586.

#### 2-(4-Fluorophenyl)-3-(2-(isopropylamino)ethyl)thiazolidin-4-one Hydrochloride (**42**)

Synthesized following the Method C section. Purified by dissolution of the crude in 1 mL of methanol and 20 mL of diethyl ether, followed by precipitation by the dropwise addition of HCl in Et_2_O (1 M, 2 mL) while stirring. A yellowish goo was formed instead of crystals. Solvent was removed by pipette and the remaining material was triturated and sonicated with chloroform to afford 415 mg of pale-yellow powder (65% yield). HPLC purity 99.4%. ^1^H NMR (600 MHz, DMSO-*d*_6_) δ 8.92 (bs, 1H), 8.72 (bs, 1H), 7.55–7.48 (m, 2H), 7.29–7.21 (m, 2H), 5.96 (d, *J* = 1.8 Hz, 1H), 3.86 (dd, *J* = 15.5, 1.9 Hz, 1H), 3.73 (d, *J* = 15.3 Hz, 1H), 3.74–3.68 (m, 1H), 3.27–3.20 (m, 1H), 3.05–2.75 (m, 3H), 1.18 (t, *J* = 6.1 Hz, 6H) ppm. ^13^C NMR (151 MHz, DMSO) δ 171.52, 162.26 (d, *J* = 245.4 Hz), 135.95 (d, *J* = 2.9 Hz), 129.75 (d, *J* = 8.8 Hz), 115.82 (d, *J* = 22.0 Hz), 61.20, 49.49, 40.47, 38.83, 31.98, 18.38 ppm. ^19^F NMR (376 MHz, DMSO) δ −112.5 ppm. HRMS *m*/*z* [M + H]^+^ calcd 283.1275, found 283.1272.

#### 3-(2-(Isopropylamino)ethyl)-2-(naphthalen-1-yl)thiazolidin-4-one Hydrochloride (**43**)

Synthesized following the Method C section. Purified by dissolution of the crude in 1 mL of ethyl acetate and 20 mL of diethyl ether, followed by precipitation by the dropwise addition of HCl in Et_2_O (1 M, 2 mL) while stirring. The solid was filtered, washed with diethyl ether, and collected to afford 532 mg of pale-yellow crystals (76% yield). ^1^H NMR (400 MHz, DMSO-*d*_6_) δ 8.84 (s, 2H), 8.08 (d, *J* = 8.2 Hz, 1H), 8.00 (dd, *J* = 7.8, 1.8 Hz, 1H), 7.94 (d, *J* = 8.2 Hz, 1H), 7.70–7.47 (m, 3H), 7.42–7.39 (m, 1H), 6.81 (bs, 1H), 3.94 (dd, *J* = 10.8, 4.6 Hz, 1H), 3.77 (s, 2H), 3.33–3.07 (m, 3H), 3.03–2.93 (m, 1H), 1.20 (d, *J* = 6.4 Hz, 6H) ppm. ^13^C NMR (100 MHz, DMSO) δ 172.1, 133.6, 129.7, 128.9, 128.7, 126.5, 125.9, 125.3, 122.4, 59.3, 49.5, 40.7, 39.6, 31.5, 18.1 ppm. HRMS *m*/*z* [M + H]^+^ calcd 315.1526, found 315.1519.

#### 3-(2-(Cyclopropylamino)ethyl)-2-(2,4-dichlorophenyl)thiazolidin-4-one Hydrochloride (**46**)

300 μL of a 1 M stock solution of the corresponding bromide in CHCl_3_ was added into an MW-suitable vial equipped with a magnet bar. Then, 0.2 mL of cyclopropylamine (10 equiv) was added while stirring. The reaction vessel was sealed and then heated in the MW reactor for 30 min at 100 °C. Then, the reaction mixture was diluted with EtOAc (15 mL), washed with Na_2_CO_3_ (sat., 5 mL), brine (5 mL), dried over Na_2_SO_4_, filtered, and concentrated under vacuum. The crude oil was dissolved in 1 mL of EtOAc and then diluted with Et_2_O (10 mL). Then, 0.3 mL of a HCl solution (2 M in Et_2_O) was added dropwise while stirring. The precipitated solid was collected by filtration to afford the desired product as 53 mg of pale-yellow crystals (48% yield). HPLC purity 95.9%. ^1^H NMR (600 MHz, DMSO-*d*_6_) δ 8.98 (bs, 1H), 8.84 (bs, 1H), 7.74 (d, *J* = 2.1 Hz, 1H), 7.51 (dd, *J* = 8.4, 2.1 Hz, 1H), 7.35 (bs, 1H), 6.20 (d, *J* = 1.7 Hz, 1H), 3.95–3.86 (m, 1H), 3.76 (dd, *J* = 15.6, 1.8 Hz, 1H), 3.69 (d, *J* = 15.5 Hz, 1H), 3.19–3.08 (m, 2H), 2.96–2.89 (m, 1H), 2.77–2.67 (m, 1H), 0.88–0.77 (m, 2H), 0.75–0.68(m, 2H) ppm. ^13^C NMR (151 MHz, DMSO) δ 172.0, 136.1, 133.9, 132.9, 129.8, 128.2 (x2), 58.9, 44.5, 39.6, 31.5, 29.7, 3.5 (x2) ppm. HRMS *m*/*z* [M + H]^+^ calcd 331.0433, found 331.0433.

#### 3-(2-(Cyclopropylamino)ethyl)-2-(4-fluorophenyl)thiazolidin-4-one Hydrochloride (**49**)

650 μL of a 1 M solution of the corresponding bromide in DCM was placed in a vial equipped with a stirring bar, and then pyridine (1 equiv) and cyclopropylamine (3 equiv) were added. The mixture was stirred at 40 °C overnight. The crude mixture was diluted with DCM (10 mL) and a white precipitate appeared. The contents were filtered over paper, and the filtered solvent was removed under vacuum. The crude was purified by flash chromatography using Biotage Isolera System, equipped with Biotage Sfär cartridge and DCM/MeOH/NH_3_ as solvent system. The product was obtained as 74 mg of a yellowish oil (31% yield). A small fraction was crystallized with HCl (2 M in Et_2_O) for characterization and testing. ^1^H NMR (400 MHz, DMSO-*d*_6_) δ 9.28 (bs, 1H), 9.05 (bs, 1H), 7.56–7.48 (m, 2H), 7.31–7.20 (m, 2H), 5.97 (d, *J* = 1.7 Hz, 1H), 3.86 (dd, *J* = 15.3, 1.9 Hz, 1H), 3.83–3.68 (m, 2H), 3.08–2.97 (m, 2H), 2.89 (dt, *J* = 14.3, 5.8 Hz, 1H), 2.71–2.61 (m, 1H), 0.91–0.80 (m, 2H), 0.75–0.64 (m, 2H) ppm. ^13^C NMR (100 MHz, DMSO) δ 171.49, (100 MHz, DMSO-*d*_6_) δ 162.23 (d, *J* = 245.4 Hz), 135.86 (d, *J* = 3.0 Hz), 129.74 (d, *J* = 8.5 Hz), 115.81 (d, *J* = 21.7 Hz), 61.04, 44.01, 38.46, 32.02, 29.56, 2.97, 2.94 ppm. ^19^F NMR (376 MHz, DMSO) δ −112.72 ppm. HRMS *m*/*z* [M + H]^+^ calcd 281.1119, found 281.1123.

#### 2-(2,4-Dichlorophenyl)-3-(3-(methylamino)propyl)thiazolidin-4-one Hydrochloride (**50**)

Synthesized following the Method C section. Purified by dissolution of the crude in 5 mL of ethanol and 15 mL of ethyl acetate, followed by precipitation by the dropwise addition of HCl in Et_2_O (1 M, 2 mL) while stirring. The solid was filtered, washed with diethyl ether, and collected to afford 339 mg of white crystals (48% yield). HPLC purity 95.9%. ^1^H NMR (400 MHz, Methanol-*d*_4_) δ 7.56 (d, *J* = 2.1 Hz, 1H), 7.49–7.33 (m, 2H), 6.22 (d, *J* = 2.0 Hz, 1H), 3.84 (dd, *J* = 15.7, 2.0 Hz, 1H), 3.78–3.64 (m, 2H), 3.07–2.87 (m, 3H), 2.69 (s, 3H), 2.04–1.80 (m, 2H) ppm. ^13^C NMR (100 MHz, MeOD) δ 174.9, 137.0, 136.4, 134.9, 131.1, 130.3, 129.3, 61.1, 47.7, 41.4, 33.6, 32.8, 25.1 ppm. HRMS *m*/*z* [M + H]^+^ calcd 319.0433, found 319.0434.

#### 2-(4-Fluorophenyl)-3-(3-(methylamino)propyl)thiazolidin-4-one Hydrochloride (**51**)

Synthesized following the Method C section. Purified by dissolution of the crude in 1 mL of methanol and 20 mL of diethyl ether, followed by precipitation by the dropwise addition of HCl in Et_2_O (1 M, 2 mL) while stirring. The solid was filtered, washed with diethyl ether, and collected to afford 340 mg of pale-yellow crystals (56% yield). HPLC purity 95.1%. ^1^H NMR (400 MHz, Methanol-*d*_4_) δ 7.56–7.41 (m, 2H), 7.16 (t, *J* = 8.7 Hz, 2H), 5.88 (d, *J* = 2.0 Hz, 1H), 3.90 (dd, *J* = 15.7, 2.0 Hz, 1H), 3.77 (d, *J* = 15.7 Hz, 1H), 3.55 (dt, *J* = 14.5, 7.2 Hz, 1H), 3.02–2.84 (m, 3H), 2.65 (s, 3H), 1.85–1.74 (m, 1H), 1.74–1.63 (m, 1H) ppm. ^13^C NMR (100 MHz, MeOD) δ 174.6, 164.58 (d, *J* = 247.3 Hz), 136.73 (d, *J* = 3.2 Hz), 130.90 (d, *J* = 8.6 Hz), 116.99 (d, *J* = 22.2 Hz), 64.4, 47.7, 41.1, 33.7, 33.3, 25.2 ppm. ^19^F NMR (376 MHz, MeOD) δ −114.1 ppm. HRMS *m*/*z* [M + H]^+^ calcd 269.1119, found 269.1117.

#### 2-(2,4-Dichlorophenyl)-3-(2-(methylamino)ethyl)thiazolidin-4-one Hydrochloride (**52**)

Synthesized following the Method C section. Purified by dissolution of the crude in 10 mL of ethyl acetate and 10 mL of diethyl ether, followed by precipitation by the dropwise addition of HCl in Et_2_O (1 M, 2 mL) while stirring. The solid was filtered, washed with diethyl ether, and collected to afford 353 mg of pale-yellow crystals (52% yield). ^1^H NMR (400 MHz, DMSO-*d*_6_) δ 9.26 (bs, 2H), 7.70 (d, *J* = 2.1 Hz, 1H), 7.49 (dd, *J* = 8.4, 2.2 Hz, 1H), 7.38–7.32 (m, *J* = 8.5 Hz, 1H), 6.24 (s, 1H), 3.89 (dt, *J* = 13.9, 6.5 Hz, 1H), 3.72 (s, 2H), 3.05–2.97 (m, 2H), 2.96–2.87 (m, 1H), 2.49 (s, 3H) ppm. ^13^C NMR (100 MHz, DMSO) δ 172.2, 135.9, 133.8, 132.9, 129.8, 128.1 (x2), 58.9, 45.1, 39.0, 32.5, 31.6 ppm. HRMS *m*/*z* [M + H]^+^ calcd 305.0277, found 305.0278.

#### 2-(4-Fluorophenyl)-3-(2-(methylamino)ethyl)thiazolidin-4-one Hydrochloride (**53**)

Synthesized following the Method C section. Purified by dissolution of the crude in 1 mL of ethyl acetate and 20 mL of diethyl ether, followed by precipitation by the dropwise addition of HCl in Et_2_O (1 M, 2 mL) while stirring. The solid was filtered, washed with diethyl ether, and collected to afford 417 mg of pale-yellow crystals (72% yield). ^1^H NMR (400 MHz, DMSO-*d*_6_) δ 9.15 (bs, 2H), 7.78–7.39 (m, 2H), 7.35–7.06 (m, 2H), 6.00 (s, 1H), 3.86–3.78 (m, 1H), 3.75 (m, 1H), 3.04–2.96 (m, 1H), 2.97–2.88 (m, 1H), 2.88–2.79 (m, 1H), 2.47 (s, 3H) ppm. ^13^C NMR (100 MHz, DMSO) δ 171.60, 163.46, 161.02, 135.76, 135.73, 129.90, 129.81, 115.90, 115.69, 61.11, 44.93, 38.51, 32.41, 32.19 ppm. ^19^F NMR (376 MHz, DMSO) δ −112.7 ppm. HRMS *m*/*z* [M + H]^+^ calcd 255.0962, found 255.0959.

#### *N*-Methyl-2-(2-(naphthalen-1-yl)-4-oxothiazolidin-3-yl)ethan-1-aminium Chloride (**54**)

Synthesized following the Method C section. Purified by dissolution of the crude in 1 mL of ethyl acetate and 20 mL of diethyl ether, followed by precipitation by the dropwise addition of HCl in Et_2_O (1 M, 2 mL) while stirring. The solid was filtered, washed with diethyl ether, and collected to afford 440 mg of yellow crystals (68% yield). ^1^H NMR (600 MHz, DMSO-*d*_6_) δ 9.11 (bs, 1H), 8.09 (d, *J* = 8.4 Hz, 1H), 8.00 (dd, *J* = 8.1, 1.5 Hz, 1H), 7.94 (d, *J* = 8.2 Hz, 1H), 7.62 (ddd, *J* = 8.4, 6.8, 1.5 Hz, 1H), 7.59 (ddd, *J* = 8.0, 6.8, 1.2 Hz, 1H), 7.54 (t, *J* = 7.7 Hz, 1H), 7.39 (bs, 1H), 6.84 (bs, 1H), 4.02–3.91 (m, 1H), 3.85–3.70 (m, 2H), 3.06 (m, 3H), 2.50 (s, 3H) ppm. ^13^C NMR (151 MHz, DMSO) δ 172.0, 133.6, 129.7, 128.8. 128.6, 126.4, 125.8, 125.2, 122.3, 58.9, 45.2, 38.9, 32.2, 31.5 ppm. HRMS *m*/*z* [M + H]^+^ calcd 287.1213, found 287.1215.

#### 2-(3-Fluorophenyl)-3-(2-(isopropylamino)ethyl)thiazolidin-4-one Hydrochloride (**55**)

Synthesized following the Method C section. Purified by dissolution of the crude in 1 mL of ethyl acetate and 10 mL of diethyl ether, followed by precipitation by the dropwise addition of HCl in Et_2_O (1 M, 2 mL) while stirring. The solid was filtered, washed with diethyl ether, and collected to afford 637 mg of white crystals (99% yield). ^1^H NMR (600 MHz, DMSO-*d*_6_) δ 9.00 (bs, 1H), 8.78 (bs, 1H), 7.46 (td, *J* = 8.0, 5.9 Hz, 1H), 7.33 (dt, *J* = 10.0, 2.1 Hz, 1H), 7.29 (dt, *J* = 7.7, 1.2 Hz, 1H), 7.24–7.17 (m, 1H), 5.98 (d, *J* = 1.8 Hz, 1H), 3.89 (dd, *J* = 15.5, 1.9 Hz, 1H), 3.80–3.61 (m, 2H), 3.28–3.18 (m, 1H), 3.06–2.80 (m, 3H), 1.19 (d, *J* = 6.4 Hz, 3H), 1.18 (d, *J* = 6.4 Hz 3H) ppm. ^13^C NMR (151 MHz, DMSO-*d*_6_) δ 162.4 (d, *J* = 244.9 Hz), 142.9 (d, *J* = 6.9 Hz), 131.2 (d, *J* = 8.3 Hz), 123.4 (d, *J* = 2.7 Hz), 115.9 (d, *J* = 21.0 Hz), 114.3 (d, *J* = 22.1 Hz), 61.3 (d, *J* = 2.1 Hz), 49.6, 40.5, 39.0, 31.9, 18.4, 18.4 ppm. ^19^F NMR (376 MHz, DMSO) δ −111.8 ppm. HRMS *m*/*z* [M + H]^+^ calcd 283.1275, found 283.1276.

#### 2-(2-Fluorophenyl)-3-(2-(isopropylamino)ethyl)thiazolidin-4-one Hydrochloride (**56**)

Synthesized following the Method C section. Purified by dissolution of the crude in 1 mL of ethanol and 10 mL of diethyl ether, followed by precipitation by the dropwise addition of HCl in Et_2_O (1 M, 2 mL) while stirring. The solid was filtered, washed with diethyl ether, and collected to afford 510 mg of pale-yellow crystals (80% yield). ^1^H NMR (600 MHz, DMSO-*d*_6_) δ 9.14 (bs, 1H), 8.88 (bs, 1H), 7.61–7.36 (m, 2H), 7.34–7.21 (m, 2H), 6.20 (d, *J* = 1.6 Hz, 1H), 3.90–3.80 (m, 1H), 3.79–3.69 (m, 2H), 3.30–3.21 (m, 1H), 3.13–3.04 (m, 1H), 3.01–2.95 (m, 1H), 2.94–2.85 (m, 1H), 1.20 (d, *J* = 2.6 Hz, 3H), 1.19 (d, *J* = 2.6 Hz, 3H) ppm. ^13^C NMR (151 MHz, DMSO) δ 171.68, 160.05 (d, *J* = 247.5 Hz), 130.93 (d, *J* = 8.5 Hz), 128.59 (d, *J* = 3.0 Hz), 126.81 (d, *J* = 10.9 Hz), 125.04 (d, *J* = 3.3 Hz), 116.20 (d, *J* = 20.8 Hz), 56.67 (d, *J* = 3.2 Hz), 49.47, 40.42, 38.7, 31.74, 18.42, 18.37 ppm. ^19^F NMR (376 MHz, DMSO) δ −118.6 ppm. HRMS *m*/*z* [M + H]^+^ calcd 283.1275, found 283.1274.

#### 2-(2,4-Difluorophenyl)-3-(2-(isopropylamino)ethyl)thiazolidin-4-one Hydrochloride (**57**)

Synthesized following the Method C section. Purified by dissolution of the crude in 1 mL of ethyl acetate and 10 mL of diethyl ether, followed by precipitation by the dropwise addition of HCl in Et_2_O (1 M, 2 mL) while stirring. The solid was filtered, washed with diethyl ether, and collected to afford 609 mg of white crystals (91% yield). ^1^H NMR (600 MHz, DMSO-*d*_6_) δ 9.09 (bs, 1H), 8.86 (bs, 1H), 7.66–7.45 (m, 1H), 7.34 (m, 1H), 7.16 (td, *J* = 8.5, 2.5 Hz, 1H), 6.19 (d, *J* = 1.7 Hz, 1H), 3.82 (m, 1H), 3.79–3.66 (m, 2H), 3.26 (m, 1H), 3.07 (m, 1H), 3.02–2.94 (m, 1H), 2.93–2.85 (m, 1H), 1.21 (d, *J* = 2.5 Hz, 3H), 1.20 (d, *J* = 2.5 Hz, 3H) ppm. ^13^C NMR (151 MHz, DMSO) δ 171.59, 162.48 (dd, *J* = 248.2, 12.6 Hz), 160.30 (dd, *J* = 250.7, 12.6 Hz), 130.32 (dd, *J* = 10.2, 4.6 Hz), 123.27 (dd, *J* = 11.1, 3.7 Hz), 112.11 (dd, *J* = 21.4, 3.4 Hz), 104.87 (t, *J* = 25.7 Hz), 56.4 (d, *J* = 1.8 Hz), 49.49, 40.44, 39.62, 31.80, 18.42, 18.38 ppm. ^19^F NMR (376 MHz, DMSO-*d*_6_) δ −108.26 (d, *J* = 8.7 Hz), −113.92 (d, *J* = 8.4 Hz) ppm. HRMS *m*/*z* [M + H]^+^ calcd 301.1181, found 301.1181.

#### 2-(2,3-Difluorophenyl)-3-(2-(isopropylamino)ethyl)thiazolidin-4-one Hydrochloride (**58**)

Synthesized following the Method C section. Purified by dissolution of the crude in 1 mL of ethyl acetate and 10 mL of diethyl ether, followed by precipitation by the dropwise addition of HCl in Et_2_O (1 M, 2 mL) while stirring. The solid was filtered, washed with diethyl ether, and collected to afford 601 mg of white crystals (89% yield). ^1^H NMR (600 MHz, DMSO-*d*_6_) δ 9.08 (bs, 1H), 8.86 (bs, 1H), 7.47 (dtd, *J* = 10.1, 8.0, 1.7 Hz, 1H), 7.38–6.98 (m, 2H), 6.25 (d, *J* = 1.7 Hz, 1H), 3.84 (dt, *J* = 14.1, 7.1 Hz, 1H), 3.81–3.70 (m, 2H), 3.27 (dq, *J* = 12.0, 5.9 Hz, 1H), 3.15–3.05 (m, 1H), 3.05–2.97 (m, 1H), 2.97–2.79 (m, 1H), 1.21 (d, *J* = 6.5 Hz, 3H), 1.20 (d, *J* = 6.5 Hz, 3H) ppm. ^13^C NMR (151 MHz, DMSO-*d*_6_) δ 171.7, 149.8 (dd, *J* = 246.6, 12.0 Hz), 147.8 (dd, *J* = 249.5, 13.4 Hz), 129.6 (d, *J* = 7.7 Hz), 125.5 (dd, *J* = 7.1, 4.3 Hz), 123.7 (d, *J* = 3.2 Hz), 117.9 (d, *J* = 17.0 Hz), 56.3 (m), 49.5, 40.5, 39.2, 31.7, 18.5, 18.4 ppm. ^19^F NMR (376 MHz, DMSO-*d*_6_) δ −137.99 (d, *J* = 21.2 Hz), −144.56 (d, *J* = 21.3 Hz) ppm. HRMS *m*/*z* [M + H]^+^ calcd 301.1181, found 301.1181.

### In Vitro Enzymatic Activity Assay

IC_50_ values were determined for recombinant *Aa*AChE1, *Ag*AChE1, G122S-AgAChE1, and *h*AChE and compared to previously reported and validated data obtained using commercial compounds.^23^ Freshly prepared stock solutions of the compounds were prepared from solid material in DMSO at a concentration of 100 mM. Working dilutions thereof were prepared in either 0.1 M sodium phosphate buffer (pH 7.4) or Milli-Q water, depending on the solubility of the compounds. Compound solutions of eight different concentrations up to a maximum of 1 mM were used. Activity measurements were performed using secreted non-purified proteins in growth medium, and enzymatic activity was measured using the Ellman assay^63^ adapted to a 96-well format. Liquid handling of a buffer solution containing buffer, enzyme, and reagent was performed using a QIAgility robotic benchtop instrument (Qiagen). Compounds were added manually and reactions were then initiated by adding the substrate using the FlexStation 3 Multi-Mode Microplate Reader (Molecular Devices). The assay was performed at 30 °C in a final assay volume of 200 μL of 0.1 M phosphate buffer (pH 7.4) containing 0.2 mM of the reagent 5,5′-dithiobis(2-nitrobenzoic acid) and 1 mM of the substrate acetylthiocholine iodide. The enzymatic reaction was measured after 30 s uniform incubation by monitoring changes in the absorbance of individual wells at 412 nm over 60 s in the same FlexStation 3 Multi-Mode Microplate Reader as mentioned above. The average slope determined for eight positive (uninhibited) controls on each plate was taken to represent 100% activity and the activity observed in the sample wells was quantified in relation to this value. IC_50_ values were calculated using nonlinear regression (curve fitting) in GraphPad Prism and the log [inhibitor] vs response variable slope equation was fitted using four parameters. For all four targets, the compounds were tested at least twice at different time points and with newly prepared dilutions from solid material of each replicate.

Enzyme kinetics, including *K*_i_ determinations, were made using a similar protocol as for the IC_50_ experiments as described above. Eight concentrations of the substrate acetylthiocholine iodide were used based on the *K*_m_ value for each enzyme^23^ and kinetic experiments were run for each of the eight concentrations of the inhibitor, which were designed based on the IC_50_ values. When preliminary data showed that the *K*_i_ value differed from the IC_50_ value, the concentrations were altered to improve the experimental design. The enzyme kinetic experiments were made in triplicates. Michaelis–Menten and noncompetitive inhibition curve fitting (nonlinear regression) were made in GraphPad Prism.

### Molecular Docking

Dockings were made to crystal structures of AChE without and with included water molecules. Water molecules in the active site gorge were investigated using crystal structures with resolution ≤2.3 Å (Tables S23–S25). For the mosquito enzymes, only two G122S-*Ag*AChE1 structures matched the criteria, while 20 *h*AChE and 19 *m*AChE structures were used for the human enzyme. The structures were superimposed and water molecules that were present in more than 60% of the structures were included in the dockings with waters (Tables S26 and S27). Water molecules that according to the crystal structures could be replaced by inhibitors were not included. The 3D coordinates of *h*AChE, *Ag*AChE1, and G122S-*Ag*AChE1 were obtained from previously published crystal structures (PDB codes 4EY4 (excluded waters), 6O5V (included waters), 5X61, and 6ARX),^64−66^ and the structures were aligned with that of a previously reported noncovalent inhibitor *m*AChE complex (PDB code 5FUM).^42^ The residue Tyr489_Ag_ (Tyr337_h_) was manually modified for each enzyme to match the “open” conformation of the residue in 5FUM. The structures were minimized with Macromodel using constraints on all heavy atoms with a constant force of 100 and a freedom value of 0.2 Å. The protein structures were then minimized and optimized with the Protein Preparation tool in Maestro, and the grid receptor was generated with Glide by using the aligned ligand from 5FUM as centroid, resulting in a cubic box of 20 Å length. Inhibitors were docked using Glide within the Schrödinger software^67^ with Standard Precision settings, writing 50 poses per ligand and performing a post-minimization on the resulting conformations. Finally, the inhibitors were ranked by docking score and docking poses with Glide score values ≤ −8.2 were visually analyzed.

### Mosquito Rearing and In Vivo Testing with *Anopheles* and *Aedes* mosquitoes

The *An. gambiaes.s.* Kisumu and *Ae. aegypti* Mombasa strains, both from Kenya, were used for in vivo testing of the intrinsic activity of selected compounds. These mosquitoes have been colonized at KEMRI for over 20 years and are routinely tested to verify their susceptibility to permethrin and deltamethrin in accordance with WHO tube bioassay guidelines using diagnostic concentrations of 0.75% permethrin and 0.05% deltamethrin impregnated on filter paper. Mosquito rearing is conducted in an insectary maintained at 27–28 °C and approximately 80% humidity on a 12/12 h light and darkness cycle and maintained at optimal larval concentrations to avoid possible effects of competition. Mosquito larvae are fed on finely ground Sera Vipan staple diet (Sera, Germany) and reared in tap water that is dechlorinated by allowing it to stand in a bucket in the insectary chamber for 24 h. Adult mosquitoes are fed on a fresh 10% (w/v) glucose solution meal daily and on hamster (*Mesocricetus auratus*) as a source of blood meals when egg production is desired.

In vivo testing of the compounds’ intrinsic insecticidal activity was performed in accordance with WHO guidelines for testing adulticides and larvicides. This involves topical application of the compounds at different concentrations in acetone on the upper part of the protonum of mosquitoes using a micropipette. Five-day-old female non-blood-fed mosquitoes were used, and testing was performed on batches of 5 mosquitoes at a time. Each batch of 5 mosquitoes was placed in a 500 mL paper cup and anesthetized by placing the cup in a −20 °C freezer for 3 min. Mosquitoes were then gently poured onto a freezer pack cooled to −20 °C and overlaid with paper towel. The compound in acetone was then deposited on the upper part of the protonum. A total volume of 0.1 μL of the compound in acetone at the required concentration was applied to each mosquito. As a negative control, 0.1 μL of pure acetone was applied on some mosquitoes. After the topical application, the mosquitoes were returned to the paper cups and placed back in the insectary, where they were given a glucose meal and maintained under standard conditions. Mosquito mortality was recorded after 24 and 48 h.

## Supporting Information Available

Starting materials structures; tables of complete IC_50_ results; dose–response IC50 curves against *Ag*AChE1, *Aa*AChE1, *h*AChE, and G122S-AgAChE1; in vivo raw data; molecular docking material; inhibition kinetics data; NMR spectra; and analytical HPLC (PDF)
Molecular formula string (CSV)
Coordinates of ligands docked to *Ag*AChE1 structures (PDB) and *h*AChE structures (PDB)

## Supplementary Material

jm3c00234_si_002.csv

jm3c00234_si_003.pdb

jm3c00234_si_004.pdb

## Abbreviations Used

AChE: acetylcholinesterase
*Ae. aegypti*: *Aedes aegypti*
*An. gambiae*: *Anopheles gambiae*
WHO: World Health Organization
*h*AChE: human acetylcholinesterase
*Ag*AChE1: *Anopheles gambiae* acetylcholinesterase 1, *Ae*AChE1, *Aedes aegypti* acetylcholinesterase 1
SAR: structure–activity relationship
HTS: high-throughput screening
IC_50_: half-maximal inhibitory concentrations

## References

[ref1] World Health Organization. Ending the Neglect to Attain the Sustainable Development Goals: A Road Map for Neglected Tropical Diseases 2021–2030. 2020.

[ref2] Bhatt S.; Weiss D. J.; Cameron E.; Bisanzio D.; Mappin B.; Dalrymple U.; Battle K.; Moyes C. L.; Henry A.; Eckhoff P. A.; Wenger E. A.; Briet O.; Penny M. A.; Smith T. A.; Bennett A.; Yukich J.; Eisele T. P.; Griffin J. T.; Fergus C. A.; Lynch M.; Lindgren F.; Cohen J. M.; Murray C. L. J.; Smith D. L.; Hay S. I.; Cibulskis R. E.; Gething P. W. The effect of malaria control on Plasmodium falciparum in Africa between 2000 and 2015. Nature 2015, 526, 207–211. 10.1038/nature15535. 26375008 PMC4820050

[ref3] Hemingway J.; Hawkes N. J.; McCarroll L.; Ranson H. The molecular basis of insecticide resistance in mosquitoes. Insect Biochem. Mol. Biol. 2004, 34, 653–665. 10.1016/j.ibmb.2004.03.018. 15242706

[ref4] Rivero A.; Vezilier J.; Weill M.; Read A. F.; Gandon S. Insecticide control of vector-borne diseases: when is insecticide resistance a problem?. PLoS Pathog. 2010, 6, e1001000 10.1371/journal.ppat.1001000. 20700451 PMC2916878

[ref5] Ranson H.; N’Guessan R.; Lines J.; Moiroux N.; Nkuni Z.; Corbel V. Pyrethroid resistance in African Anopheline mosquitoes: what are the implications for malaria control?. Trends Parasitol. 2011, 27, 91–98. 10.1016/j.pt.2010.08.004. 20843745

[ref6] Hemingway J. The role of vector control in stopping the transmission of malaria: threats and opportunities. Philos. Trans. R. Soc., B 2014, 369, 20130431 10.1098/rstb.2013.0431. PMC4024224 24821917

[ref7] Weetman D.; Djogbenou L. S.; Lucas E. Copy number variation (CNV) and insecticide resistance in mosquitoes: evolving knowledge or an evolving problem?. Curr. Opin. Insect Sci. 2018, 27, 82–88. 10.1016/j.cois.2018.04.005. 30025639 PMC6056009

[ref8] Tangena J. A.; Hendriks C. M. J.; Devine M.; Tammaro M.; Trett A. E.; Williams I.; DePina A. J.; Sisay A.; Herizo R.; Kafy H. T.; Chizema E.; Were A.; Rozier J.; Coleman M.; Moyes C. L. Indoor residual spraying for malaria control in sub-Saharan Africa 1997 to 2017: an adjusted retrospective analysis. Malar. J. 2020, 19, 150 10.1186/s12936-020-03216-6. 32276585 PMC7149868

[ref9] Xi Z.; Joshi D. Chapter 14 - Genetic control of malaria and dengue using Wolbachia. In Genetic Control of Malaria and Dengue; Adelman Z. N., Ed.; Academic Press, 2016; pp 305–333.

[ref10] Patterson E. I.; Villinger J.; Muthoni J. N.; Dobel-Ober L.; Hughes G. L. Exploiting insect-specific viruses as a novel strategy to control vector-borne disease. Curr. Opin. Insect Sci. 2020, 39, 50–56. 10.1016/j.cois.2020.02.005. 32278312 PMC7302987

[ref11] Yen P. S.; Failloux A. B. A review: Wolbachia-based population replacement for mosquito control shares common points with genetically modified control approaches. Pathogens 2020, 9, 404 10.3390/pathogens9050404. 32456036 PMC7281599

[ref12] Huang W.; Wang S.; Jacobs-Lorena M. Use of microbiota to fight mosquito-borne disease. Front. Genet. 2020, 11, 196 10.3389/fgene.2020.00196. 32211030 PMC7076131

[ref13] López Del Amo V.; Bishop A. L.; Sanchez C. H. M.; Bennett J. B.; Feng X.; Marshall J. M.; Bier E.; Gantz V. M. A transcomplementing gene drive provides a flexible platform for laboratory investigation and potential field deployment. Nat. Commun. 2020, 11, 352 10.1038/s41467-019-13977-7. 31953404 PMC6969112

[ref14] Wooding M.; Naude Y.; Rohwer E.; Bouwer M. Controlling mosquitoes with semiochemicals: a review. Parasites Vectors 2020, 13, 80 10.1186/s13071-020-3960-3. 32066499 PMC7027039

[ref15] Suresh M.; Jeevanandam J.; Chan Y. S.; Danquah M. K.; Kalaiarasi J. M. V. Opportunities for metal oxide nanoparticles as a potential mosquitocide. BioNanoScience 2020, 10, 292–310. 10.1007/s12668-019-00703-2.

[ref16] Sougoufara S.; Ottih E. C.; Tripet F. The need for new vector control approaches targeting outdoor biting Anopheline malaria vector communities. Parasites Vectors 2020, 13, 295 10.1186/s13071-020-04170-7. 32522290 PMC7285743

[ref17] Toutant J.-P. Insect acetylcholinesterase: catalytic properties, tissue distribution and molecular form. Prog. Neurobiol. 1989, 32, 423–446. 10.1016/0301-0082(89)90031-2. 2660188

[ref18] Casida J. E.; Durkin K. A. Neuroactive insecticides: targets, selectivity, resistance, and secondary effects. Annu. Rev. Entomol. 2013, 58, 99–117. 10.1146/annurev-ento-120811-153645. 23317040

[ref19] Sussman J. L.; Harel M.; Frolow F.; Oefner C.; Goldman A.; Toker L.; Silman I. Atomic structure of acetylcholinesterase from Torpedo californica: a prototypic acetylcholine-binding protein. Science 1991, 253, 872–879. 10.1126/science.1678899. 1678899

[ref20] Weill M.; Fort P.; Berthomieu A.; Dubois M. P.; Pasteur N.; Raymond M. A novel acetylcholinesterase gene in mosquitoes codes for the insecticide target and is non-homologous to the ace gene in Drosophila. Proc. Biol. Sci. 2002, 269, 2007–2016. 10.1098/rspb.2002.2122. 12396499 PMC1691131

[ref21] Kim Y. H.; Lee S. H. Which acetylcholinesterase functions as the main catalytic enzyme in the Class Insecta?. Insect. Biochem. Mol. Biol. 2013, 43, 47–53. 10.1016/j.ibmb.2012.11.004. 23168079

[ref22] Alout H.; Djogbenou L.; Berticat C.; Chandre F.; Weill M. Comparison of *Anopheles gambiae* and *Culex pipiens* acetycholinesterase 1 biochemical properties. Comp. Biochem. Physiol., Part B: Biochem. Mol. Biol. 2008, 150, 271–277. 10.1016/j.cbpb.2008.03.008. 18455457

[ref23] Engdahl C.; Knutsson S.; Fredriksson S. A.; Linusson A.; Bucht G.; Ekstrom F. Acetylcholinesterases from the disease vectors *Aedes aegypti* and *Anopheles gambiae*: functional characterization and comparisons with vertebrate orthologues. PLoS One 2015, 10, e0138598 10.1371/journal.pone.0138598. 26447952 PMC4598118

[ref24] Schmidt M.; Hrabcova V.; Jun D.; Kuca K.; Musilek K. Vector control and insecticidal resistance in the African malaria mosquito Anopheles gambiae. Chem. Res. Toxicol. 2018, 31, 534–547. 10.1021/acs.chemrestox.7b00285. 29847927

[ref25] Weill M.; Lutfalla G.; Mogensen K.; Chandre F.; Berthomieu A.; Berticat C.; Pasteur N.; Philips A.; Fort P.; Raymond M. Insecticide resistance in mosquito vectors. Nature 2003, 423, 136–137. 10.1038/423136b. 12736674

[ref26] Carlier P. R.; Anderson T. D.; Wong D. M.; Hsu D. C.; Hartsel J.; Ma M.; Wong E. A.; Choudhury R.; Lam P. C.; Totrov M. M.; Bloomquist J. R. Towards a species-selective acetylcholinesterase inhibitor to control the mosquito vector of malaria, *Anopheles gambiae*. Chem.-Biol. Interact. 2008, 175, 368–375. 10.1016/j.cbi.2008.04.037. 18554580

[ref27] Hartsel J. A.; Wong D. M.; Mutunga J. M.; Ma M.; Anderson T. D.; Wysinski A.; Islam R.; Wong E. A.; Paulson S. L.; Li J.; Lam P. C.; Totrov M. M.; Bloomquist J. R.; Carlier P. R. Re-engineering aryl methylcarbamates to confer high selectivity for inhibition of *Anopheles gambiae* versus human acetylcholinesterase. Bioorg. Med. Chem. Lett. 2012, 22, 4593–4598. 10.1016/j.bmcl.2012.05.103. 22738634 PMC3389130

[ref28] Wong D. M.; Li J. Y.; Chen Q. H.; Han Q.; Mutunga J. M.; Wysinski A.; Anderson T. D.; Ding H. Z.; Carpenetti T. L.; Verma A.; Islam R.; Paulson S. L.; Lam P. C. H.; Totrov M.; Bloomquist J. R.; Carlier P. R. Select small core structure carbamates exhibit high contact toxicity to ″carbamate-resistant″ strain malaria mosquitoes, *Anopheles gambiae* (Akron). PLoS One 2012, 7, e46712 10.1371/journal.pone.0046712. 23049714 PMC3462181

[ref29] Wong D. M.; Li J.; Lam P. C.; Hartsel J. A.; Mutunga J. M.; Totrov M.; Bloomquist J. R.; Carlier P. R. Aryl methylcarbamates: potency and selectivity towards wild-type and carbamate-insensitive (G119S) *Anopheles gambiae* acetylcholinesterase, and toxicity to G3 strain An. gambiae. Chem. Biol. Interact 2013, 203, 314–318. 10.1016/j.cbi.2012.09.001. 22989775 PMC3578073

[ref30] Camerino E.; Wong D. M.; Tong F.; Korber F.; Gross A. D.; Islam R.; Viayna E.; Mutunga J. M.; Li J.; Totrov M. M.; Bloomquist J. R.; Carlier P. R. Difluoromethyl ketones: Potent inhibitors of wild type and carbamate-insensitive G119S mutant *Anopheles gambiae* acetylcholinesterase. Bioorg. Med. Chem. Lett. 2015, 25, 4405–4411. 10.1016/j.bmcl.2015.09.019. 26386602 PMC4593063

[ref31] Verma A.; Wong D. M.; Islam R.; Tong F.; Ghavami M.; Mutunga J. M.; Slebodnick C.; Li J.; Viayna E.; Lam P. C.; Totrov M. M.; Bloomquist J. R.; Carlier P. R. 3-Oxoisoxazole-2(3H)-carboxamides and isoxazol-3-yl carbamates: Resistance-breaking acetylcholinesterase inhibitors targeting the malaria mosquito, *Anopheles gambiae*. Bioorg. Med. Chem. 2015, 23, 1321–1340. 10.1016/j.bmc.2015.01.026. 25684426 PMC4346421

[ref32] Mutunga J. M.; Chen Q. H.; Wong D. M.; Lam P. C.; Li J.; Totrov M. M.; Gross A. D.; Carlier P. R.; Bloomquist J. R. Bivalent carbamates as novel control agents of the malaria mosquito, *Anopheles gambiae*. Chimia (Aarau) 2016, 70, 704–708. 10.2533/chimia.2016.704. 27779928

[ref33] Carlier P. R.; Chen Q. H.; Verma A.; Wong D. M.; Mutunga J. M.; Muller J.; Islam R.; Shimozono A. M.; Tong F.; Li J.; Totrov M.; Bloomquist J. R. Select beta- and gamma-branched 1-alkylpyrazol-4-yl methylcarbamates exhibit high selectivity for inhibition of Anopheles gambiae versus human acetylcholinesterase. Pest. Biochem. Physiol. 2018, 151, 32–39. 10.1016/j.pestbp.2018.02.003. PMC6277143 30524149

[ref34] Mutunga J. M.; Ma M.; Chen Q. H.; Hartsel J. A.; Wong D. M.; Ding S.; Totrov M.; Carlier P. R.; Bloomquist J. R. Mosquito acetylcholinesterase as a target for novel phenyl-substituted carbamates. Int. J. Environ. Res. Public Health 2019, 16, 1500 10.3390/ijerph16091500. 31035318 PMC6539584

[ref35] Pang Y.-P.; Ekstrom F.; Polsinelli G. A.; Gao Y.; Rana S.; Hua D. H.; Andersson B.; Andersson P. O.; Peng L.; Singh S. K.; Mishra R. K.; Zhu K. Y.; Fallon A. M.; Ragsdale D. W.; Brimijoin S. Selective and irreversible inhibitors of mosquito acetylcholinesterases for controlling malaria and other mosquito-borne diseases. PLoS One 2009, 4, e6851 10.1371/journal.pone.0006851. 19714254 PMC2731169

[ref36] Dou D.; Park J. G.; Rana S.; Madden B. J.; Jiang H.; Pang Y. P. Novel selective and irreversible mosquito acetylcholinesterase inhibitors for controlling malaria and other mosquito-borne diseases. Sci. Rep. 2013, 3, 1068 10.1038/srep01068. 23323211 PMC3545233

[ref37] Gorecki L.; Andrys R.; Schmidt M.; Kucera T.; Psotka M.; Svobodova B.; Hrabcova V.; Hepnarova V.; Bzonek P.; Jun D.; Kuca K.; Korabecny J.; Musilek K. Cysteine-targeted insecticides against A. gambiae acetylcholinesterase are neither selective nor reversible inhibitors. ACS Med. Chem. Lett. 2020, 11, 65–71. 10.1021/acsmedchemlett.9b00477. 31938465 PMC6956356

[ref38] Alout H.; Labbe P.; Berthomieu A.; Djogbenou L.; Leonetti J. P.; Fort P.; Weill M. Novel AChE inhibitors for sustainable insecticide resistance management. PLoS One 2012, 7, e47125 10.1371/journal.pone.0047125. 23056599 PMC3466212

[ref39] Engdahl C.; Knutsson S.; Ekstrom F.; Linusson A. Discovery of Selective Inhibitors Targeting Acetylcholinesterase 1 from Disease-Transmitting Mosquitoes. J. Med. Chem. 2016, 59, 9409–9421. 10.1021/acs.jmedchem.6b00967. 27598521

[ref40] Hrabcova V.; Korabecny J.; Manyova B.; Matouskova L.; Kucera T.; Dolezal R.; Musilek K.; Gorecki L.; Nepovimova E.; Kuca K.; Jun D. Bis-isoquinolinium and bis-pyridinium acetylcholinesterase inhibitors: in vitro screening of probes for novel selective insecticides. RSC Adv. 2017, 7, 39279–39291. 10.1039/C7RA05838A.

[ref41] Knutsson S.; Kindahl T.; Engdahl C.; Nikjoo D.; Forsgren N.; Kitur S.; Ekstrom F.; Kamau L.; Linusson A. N-Aryl-N’-ethyleneaminothioureas effectively inhibit acetylcholinesterase 1 from disease-transmitting mosquitoes. Eur. J. Med. Chem. 2017, 134, 415–427. 10.1016/j.ejmech.2017.03.050. 28433681

[ref42] Knutsson S.; Engdahl C.; Kumari R.; Forsgren N.; Lindgren C.; Kindahl T.; Kitur S.; Wachira L.; Kamau L.; Ekstrom F.; Linusson A. Noncovalent inhibitors of mosquito acetylcholinesterase 1 with resistance-breaking potency. J. Med. Chem. 2018, 61, 10545–10557. 10.1021/acs.jmedchem.8b01060. 30339371

[ref43] Venugopala K. N.; Ramachandra P.; Tratrat C.; Gleiser R. M.; Bhandary S.; Chopra D.; Morsy M. A.; Aldhubiab B. E.; Attimarad M.; Nair A. B.; Sreeharsha N.; Venugopala R.; Deb P. K.; Chandrashekharappa S.; Khalil H. E.; Alwassil O. I.; Abed S. N.; Bataineh Y. A.; Palenge R.; Haroun M.; Pottathil S.; Girish M. B.; Akrawi S. H.; Mohanlall V. Larvicidal activities of 2-aryl-2,3-dihydroquinazolin-4-ones against malaria vector Anopheles arabiensis, in silico ADMET prediction and molecular target investigation. Molecules 2020, 25, 1316 10.3390/molecules25061316. 32183140 PMC7144721

[ref44] Franca S. B.; de Lima L. C. B.; Cunha C. R. D.; Anunciacao D. S.; da Silva E. F.; Barros M.; Lima D. J. D. Larvicidal activity and in silico studies of cinnamic acid derivatives against *Aedes aegypti* (Diptera: Culicidae). Bioorg. Med. Chem. 2021, 44, 116299 10.1016/j.bmc.2021.116299. 34225166

[ref45] Sandhanam S. D.; Ganesan P.; Stalin A.; Michael G. P.; Balakrishna K.; Pandikumar P.; Ignacimuthu S.; Naif A. A. Effect of compound isolated from *Lawsonia inermis* (L.) (Myrtales: Lythraceae) on the immature stages of filarial vector Culex quinquefasciatus Say (Diptera: Culicidae) and its docking analysis with Acetylcholinesterase (AChE1). Biocatal. Agric. Biotechnol. 2018, 15, 210–218. 10.1016/j.bcab.2018.06.004.

[ref46] De-Campos-Bortolucci W.; Marko-de-Oliveira H. L.; Roque-Oliva L.; Goncalves J. E.; Piau-Júnior R.; Mariano-Fernandez C. M.; Barros-Colauto N.; Linde G. A.; Gazim Z. C. Crude extract of the tropical tree *Gallesia integrifolia* (Phytolaccaceae) for the control of *Aedes aegypti* (Diptera: Culicidae) larvae. Rev. Biol. Trop. 2021, 69, 153–169. 10.15517/rbt.v69i1.41225.

[ref47] Nirwan S.; Chahal V.; Kakkar R. Thiazolidinones: synthesis, reactivity, and their biological applications. J. Heterocycl. Chem. 2019, 56, 1239–1253. 10.1002/jhet.3514.

[ref48] Tripathi A. C.; Gupta S. J.; Fatima G. N.; Sonar P. K.; Verma A.; Saraf S. K. 4-Thiazolidinones: the advances continue. Eur. J. Med. Chem. 2014, 72, 52–77. 10.1016/j.ejmech.2013.11.017. 24355348

[ref49] Solomon V. R.; Haq W.; Srivastava K.; Puri S. K.; Katti S. B. Synthesis and antimalarial activity of side chain modified 4-aminoquinoline derivatives. J. Med. Chem. 2007, 50, 394–398. 10.1021/jm061002i. 17228883

[ref50] Rojas Ruiz F. A.; Garcia-Sanchez R. N.; Estupinan S. V.; Gomez-Barrio A.; Torres Amado D. F.; Perez-Solorzano B. M.; Nogal-Ruiz J. J.; Martinez-Fernandez A. R.; Kouznetsov V. V. Synthesis and antimalarial activity of new heterocyclic hybrids based on chloroquine and thiazolidinone scaffolds. Bioorg. Med. Chem. 2011, 19, 4562–4573. 10.1016/j.bmc.2011.06.025. 21723734

[ref51] Manvar D.; Kucukguzel I.; Erensoy G.; Tatar E.; Deryabasogullari G.; Reddy H.; Talele T. T.; Cevik O.; Kaushik-Basu N. Discovery of conjugated thiazolidinone-thiadiazole scaffold as anti-dengue virus polymerase inhibitors. Biochem. Biophys. Res. Commun. 2016, 469, 743–747. 10.1016/j.bbrc.2015.12.042. 26697747

[ref52] Brown F. C. 4-Thiazolidinones. Chem. Rev. 1961, 61, 463–521. 10.1021/cr60213a002.

[ref53] Inamori Y.; Okamoto Y.; Takegawa Y.; Tsujibo H.; Sakagami Y.; Kumeda Y.; Shibata M.; Numata A. Insecticidal and antifungal activities of aminorhodanine derivatives. Biosci. Biotechnol. Biochem. 1998, 62, 1025–1027. 10.1271/bbb.62.1025. 9648238

[ref54] Kaur Manjal S.; Kaur R.; Bhatia R.; Kumar K.; Singh V.; Shankar R.; Kaur R.; Rawal R. K. Synthetic and medicinal perspective of thiazolidinones: A review. Bioorg. Chem. 2017, 75, 406–423. 10.1016/j.bioorg.2017.10.014. 29102723

[ref55] Andersson C. D.; Forsgren N.; Akfur C.; Allgardsson A.; Berg L.; Engdahl C.; Qian W. X.; Ekstrom F.; Linusson A. Divergent structure-activity relationships of structurally similar acetylcholinesterase inhibitors. J. Med. Chem. 2013, 56, 7615–7624. 10.1021/jm400990p. 23984975

[ref56] Andersson C. D.; Mishra B. K.; Forsgren N.; Ekstrom F.; Linusson A. Physical mechanisms governing substituent effects on arene-arene interactions in a protein milieu. J. Phys. Chem. B 2020, 124, 6529–6539. 10.1021/acs.jpcb.0c03778. 32610016 PMC7467712

[ref57] Ekström F.; Pang Y. P.; Boman M.; Artursson E.; Akfur C.; Borjegren S. Crystal structures of acetylcholinesterase in complex with HI-6, Ortho-7 and obidoxime: Structural basis for differences in the ability to reactivate tabun conjugates. Biochem. Pharm. 2006, 72, 597–607. 10.1016/j.bcp.2006.05.027. 16876764

[ref58] Swale D. R.; Carlier P. R.; Hartsel J. A.; Ma M.; Bloomquist J. R. Mosquitocidal carbamates with low toxicity to agricultural pests: an advantageous property for insecticide resistance management. Pest. Manage. Sci. 2015, 71, 1158–1164. 10.1002/ps.3899. PMC4348351 25185896

[ref59] Nuss A. B.; Ejendal K. F.; Doyle T. B.; Meyer J. M.; Lang E. G.; Watts V. J.; Hill C. A. Dopamine receptor antagonists as new mode-of-action insecticide leads for control of Aedes and Culex mosquito vectors. PLoS Neglected Trop. Dis. 2015, 9, e0003515 10.1371/journal.pntd.0003515. PMC4368516 25793586

[ref60] Miller D. D.; Gududuru V.; Nguyen V.; Dalton J. T. Efficient microwave enhanced synthesis of 4-thiazolidinones. Synlett 2004, 2357–2358. 10.1055/s-2004-832811.

[ref61] Baughman T. W.; Sworen J. C.; Wagener K. B. The facile preparation of alkenyl metathesis synthons. Tetrahedron 2004, 60, 10943–10948. 10.1016/j.tet.2004.09.021.

[ref62] Kukharev B. F.; Stankevich V. K.; Klimenko G. R. Synthesis of N-(2-hydroxyalkyl)-4-thiazolidinones from oxazolidines. Russ. Chem. Bull. 1997, 46, 2107–2109. 10.1007/BF02495261.

[ref63] Ellman G. L.; Courtney K. D.; Andres V. Jr.; Featherstone R. M. A new and rapid colorimetric determination of acetylcholinesterase activity. Biochem. Pharm. 1961, 7, 88–95. 10.1016/0006-2952(61)90145-9. 13726518

[ref64] Han Q.; Wong D. M.; Robinson H.; Ding H.; Lam P. C. H.; Totrov M. M.; Carlier P. R.; Li J. Crystal structure of acetylcholinesterase catalytic subunits of the malaria vector *Anopheles gambiae*. Insect Sci. 2018, 25, 721–724. 10.1111/1744-7917.12450. 28247978 PMC5581290

[ref65] Cheung J.; Rudolph M. J.; Burshteyn F.; Cassidy M. S.; Gary E. N.; Love J.; Franklin M. C.; Height J. J. Structures of human acetylcholinesterase in complex with pharmacologically important ligands. J. Med. Chem. 2012, 55, 10282–10286. 10.1021/jm300871x. 23035744

[ref66] Cheung J.; Mahmood A.; Kalathur R.; Liu L.; Carlier P. R. Structure of the G119S mutant acetylcholinesterase of the malaria vector *Anopheles gambiae* reveals basis of insecticide resistance. Structure 2018, 26, 130–136 e2. 10.1016/j.str.2017.11.021. 29276037 PMC5752620

[ref67] Schrödinger Release 2019-1; Schrödinger, LLC: New York, NY, 2019.

