# Supplementary material for: Structure–Activity Relationships Reveal Beneficial Selectivity Profiles of Inhibitors Targeting Acetylcholinesterase of Disease-Transmitting Mosquitoes
Source: J Med Chem. 2023 Apr 24;66(9):6333–53. doi: 10.1021/acs.jmedchem.3c00234 (PMC10184127; doi:10.1021/acs.jmedchem.3c00234)
Supplement: Supplementary file 1 — jm3c00234_si_001.pdf [file jm3c00234_si_001.pdf]

SUPPORTING INFORMATION

Structure-Activity Relationships Reveal Beneficial Selectivity Profiles of Inhibitors Targeting Acetylcholinesterase of Disease-Transmitting Mosquitoes

Andreu Vidal-Albalat,[a] Tomas Kindahl,[a] Rajeshwari Rajeshwari,[a] Cecilia Lindgren,[a] Nina Forsgren,[b] Stanely Kitur,[c] Laura Sela Tengo,[c] Fredrik Ekström,[b] Luna Kamau,[c] Anna Linusson[a],*

a. Department of Chemistry, Umeå University, SE-90187, Umeå, Sweden.
b. CBRN Defense and Security, Swedish Defense Research Agency, SE-90621 Umeå, Sweden.
c. Centre for Biotechnology Research and Development, Kenya Medical Research Institute, 00200 Nairobi, Kenya
*Corresponding author. E-mail addresses: anna.linusson@umu.se

Starting materials structures S2

Tables of chemical structures and IC_{50} values S4

Dose Response IC_{50} curves *Ag*AChE1, *Aa*AChE1, and *h*AChE S12

Dose Response IC_{50} curves G122S-*Ag*AChE1 .... S18

In vivo raw data S21

Molecular docking S39

Inhibition kinetics S42

NMR spectra S46

Analytical HPLC S121

Starting materials structures

Aldehydes **1**

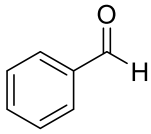

1a

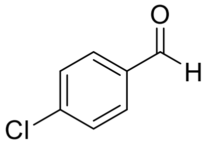

1b

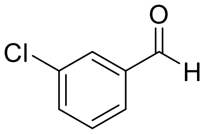

1c

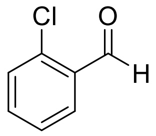

1d

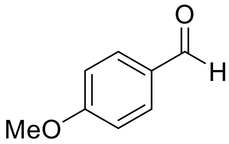

1e

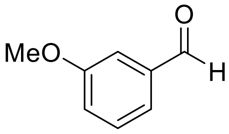

1f

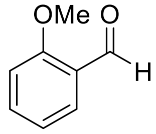

1g

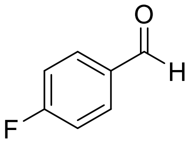

1h

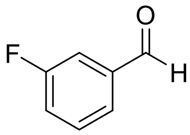

1i

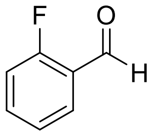

1j

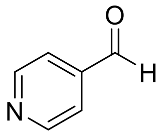

1k

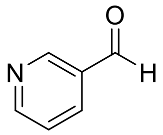

1l

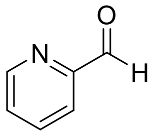

1m

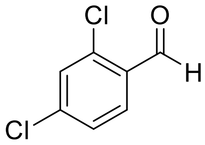

1n

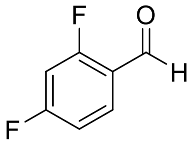

1o

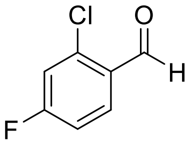

1p

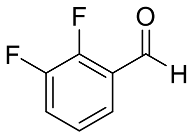

1q

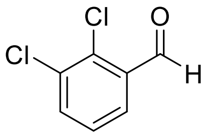

1r

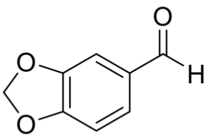

1s

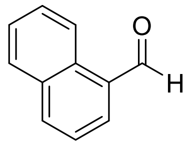

1t

Figure S1. Aldehydes as starting materials.

Amines **3**

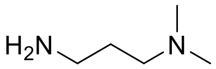

3a

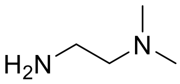

3b

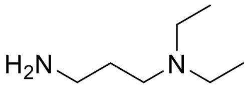

3c

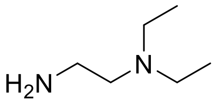

3d

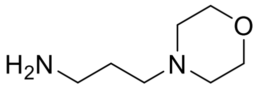

3e

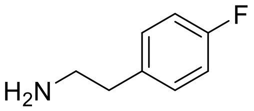

3f

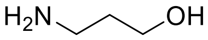

3g

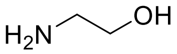

3h

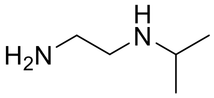

3i

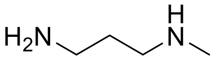

3j

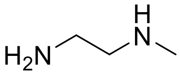

3k

Figure S2. Amines as starting materials.

Tables of chemical structures and IC$_{50}$ values

Table S1. Chemical structures and IC$_{50}$ values for compounds in set 1a.

ID	Structure[a]	IC$_{50}$ (µM)[b]			
		*Ag*AChE1	*Aa*AChE1	*h*AChE	G122S-*Ag*AChE1
**4**	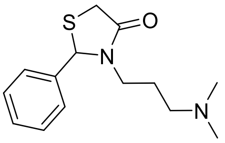	5.4 (5.1 – 5.8)	2.7 (2.5-2.9)	> 200	166 (83 – 332)
**5**	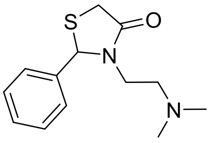	161 (130 – 199)	92 (71-120)	> 200	n.d.[c]
**6**	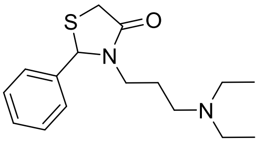	227 (141 – 365)	n.d.[c]	> 200	n.d.[c]
**7**	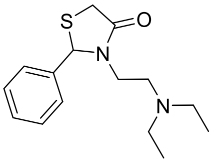	> 200	n.d.[c]	> 200	n.d.[c]
**8**[d]	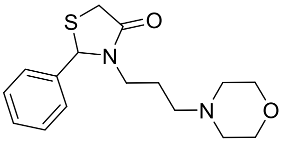	> 200	n.d.[c]	> 200	n.d.[c]
**9**[d]	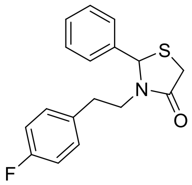	Inactive[e]	n.d.[c]	> 200	n.d.[c]
**10**[d]	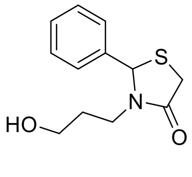	> 200	n.d.[c]	> 200	n.d.[c]
**14**[d]	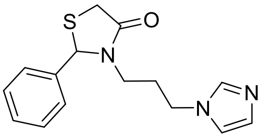	175 (147 – 208)	n.d.[c]	> 200	n.d.[c]

**15**[d]	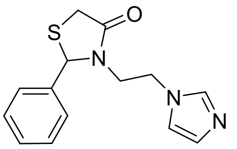	> 200	n.d.[c]	190 (143-252)	n.d.[c]
**16**[d]	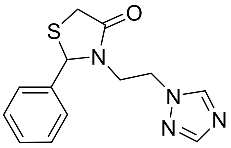	> 200	n.d.[c]	> 200	n.d.[c]
**17**[d]	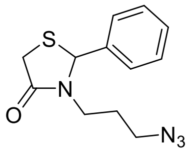	Inactive[e]	n.d.[c]	> 200	n.d.[c]

[a]Compounds tested as HCl salts unless specified. [b]95% confidence interval given in parenthesis. [c]n.d. = no data. [d]compound tested as a neutral species. [e]Inactive at 1000 µM.

Table S2. Chemical structures and IC_{50} values for compounds in set 1b.

ID	Structure	IC_{50} (μM)[a]			
		*Ag*AChE1	*Aa*AChE1	*h*AChE	G122S-*Ag*AChE1
**18**	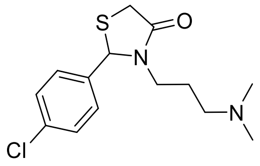	0.86 (0.62 – 1.2)	0.56 (0.41-0.76)	> 200	> 200
**19**	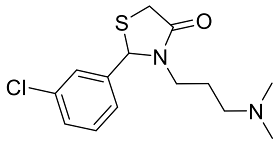	16 (14 -18)	9.5 (8.5-10)	25 (24 – 27)	> 200
**20**	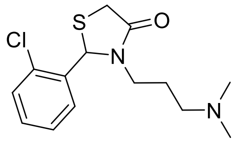	8.1 (6.9 – 9.4)	5.4 (4.0 -7.3)	134 (114 – 159)	53 (37 – 75)
**21**	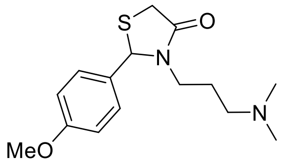	53 (40 – 72)	32 (25-41)	> 200	164 (60 – 453)
**22**	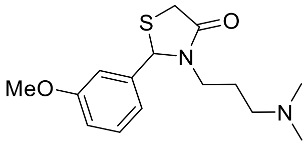	85 (65 – 110)	53 (36 – 79)	> 200	> 200
**23**	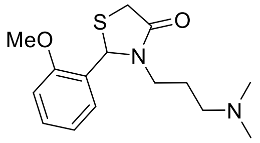	2.5 (2.4 – 2.7)	1.9 (1.7-2.1)	> 200	145 (74 – 286)
**24**	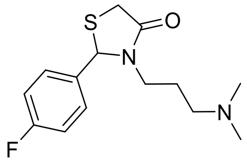	0.82 (0.72 – 0.93)	0.44 (0.31 – 0.62)	> 200	162 (54 – 482)
**25**	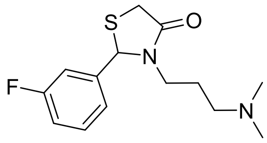	14 (13 – 15)	n.d.[c]	97 (84 – 112)	n.d.[c]

**26**	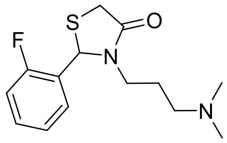	23 (18 – 28)	20 (14 – 28)	> 200	> 200
**27**	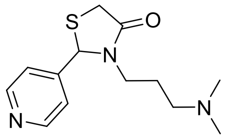	146 (114 – 186)	n.d.[c]	> 200	n.d.[c]
**28**	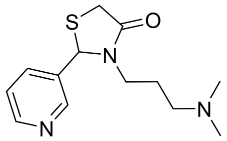	45 (39 – 52)	n.d.[c]	199 (135 – 293)	n.d.[c]
**29**	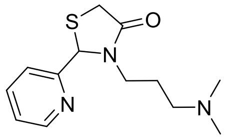	149 (104 – 212)	n.d.[c]	> 200	n.d.[c]

[a]Compounds tested as HCl salts unless specified. [b]95% confidence interval given in parenthesis. [c]n.d. = not determined.

Table S3. Chemical structures and IC_{50} values for compounds in set 2.

**ID**	**Structure**	**IC_{50} (μM)[a]**			
		*Ag*AChE1	*Aa*AChE1	*h*AChE	G122S-*Ag*AChE1
**30**	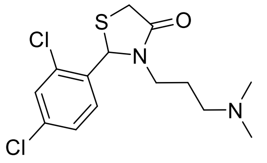	1.0 (0.76-1.4)	0.74 (4.6 – 1.2)[d]	31 (23-42)	117 (73 – 186)
**31**	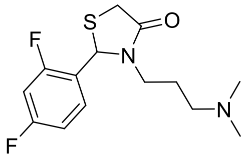	4.4 (3.8-5.1)	2.7 (2.0 – 3.7)	95 (58 - 157)	> 200
**32**	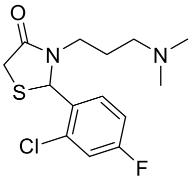	3.0 (2.4-3.6)	1.3 (0.65 – 2.5)	81 (71-92)	63 (42 – 94)
**33**	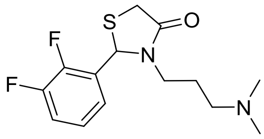	37 (29-47)	44 (35 – 55)	7.2 (6.1-8.4)	> 200
**34**	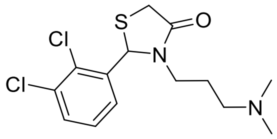	3.6 (3.1-4.2)	2.6 (1.9 – 3.4)	4.4 (3.9-5.0)	62 (46 – 84)
**35**	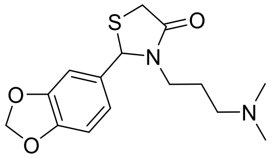	72 (61-86)	n.d.[c]	105 (80-137)	n.d.[c]
**36**	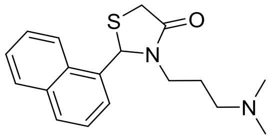	0.65 (0.61-0.86)	0.38 (0.22 – 0.67)	177 (132-237)	> 200
**37**	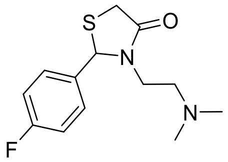	22 (20-25)	n.d.[c]	> 200	n.d.[c]

**38**	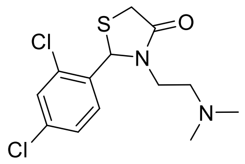	15 (13-18)	n.d.[c]	> 200	n.d.[c]
**39**	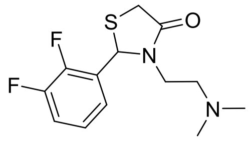	> 200	n.d.[c]	> 200	n.d.[c]
**40**	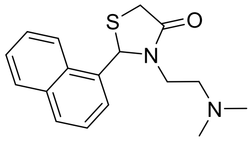	6.7 (5.1-8.7)	4.9 (2.3 – 10)	> 200	> 200

[a]Compounds tested as HCl salts unless specified. [b]95% confidence interval given in parenthesis. [c]n.d. = not determined.

Table S4. Chemical structures and IC_{50} values for compounds in design set 3.

ID	Structure	IC_{50} (μM)[a]			
		*Ag*AChE1	*Aa*AChE1	*h*AChE	G122S-*Ag*AChE1
**41**	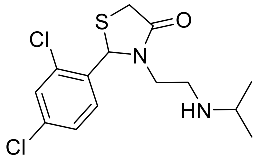	4.2 (3.4-5.2)	4.7 (22 – 100)	> 200	200 (113 – 353)
**42**	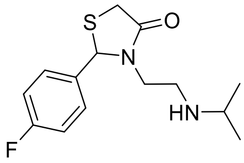	18 (16-20)	11 (4.9 – 25)	> 200	> 200
**43**	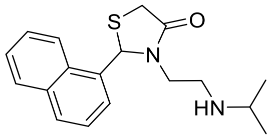	29 (24-36)	n.d.[c]	> 200	n.d.[c]
**46**	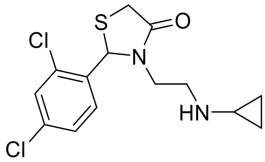	2.3 (1.9-2.6)	2.6 (1.5 – 4.5)	> 200	186 (63 – 554)
**49**	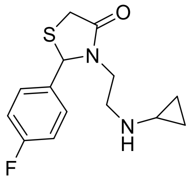	11 (7.8-14)	4.6 (4.1 – 5.3)	> 200	n.d.[c]
**50**	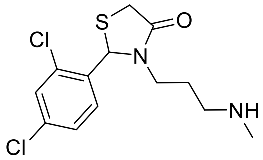	19 (14-26)	27 (15 – 48)	49 (42-58)	> 200
**51**	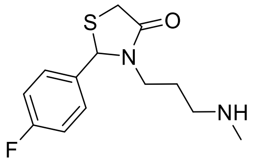	25 (20-31)	14 (8.3 – 23)	> 200	> 200
**52**	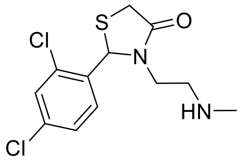	25 (19-35)	n.d.[c]	> 200	n.d.[c]

**53**	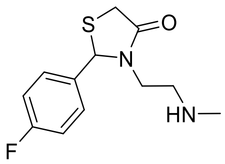	40 (35-45)	n.d.[c]	> 200	n.d.[c]
**54**	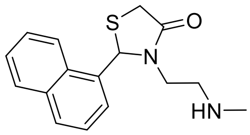	75 (54-103)	n.d.[c]	> 200	n.d.[c]
**55**	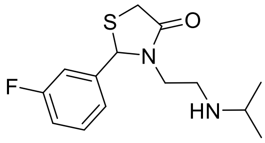	64 (48-84)	n.d.[c]	> 200	n.d.[c]
**56**	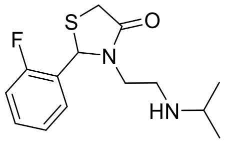	108 (75-156)	n.d.[c]	> 200	n.d.[c]
**57**	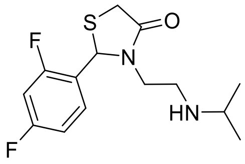	31 (25-38)	n.d.[c]	> 200	n.d.[c]
**58**	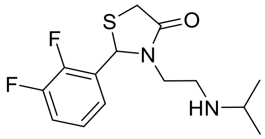	88 (66-119)	n.d.[c]	> 200	n.d.[c]

[a]Compounds tested as HCl salts unless specified. [b]95% confidence interval given in parenthesis. [c]n.d. = not determined.

Dose response IC_{50} curves *Ag*AChE1, *Aa*AChE1, and *h*AChE:

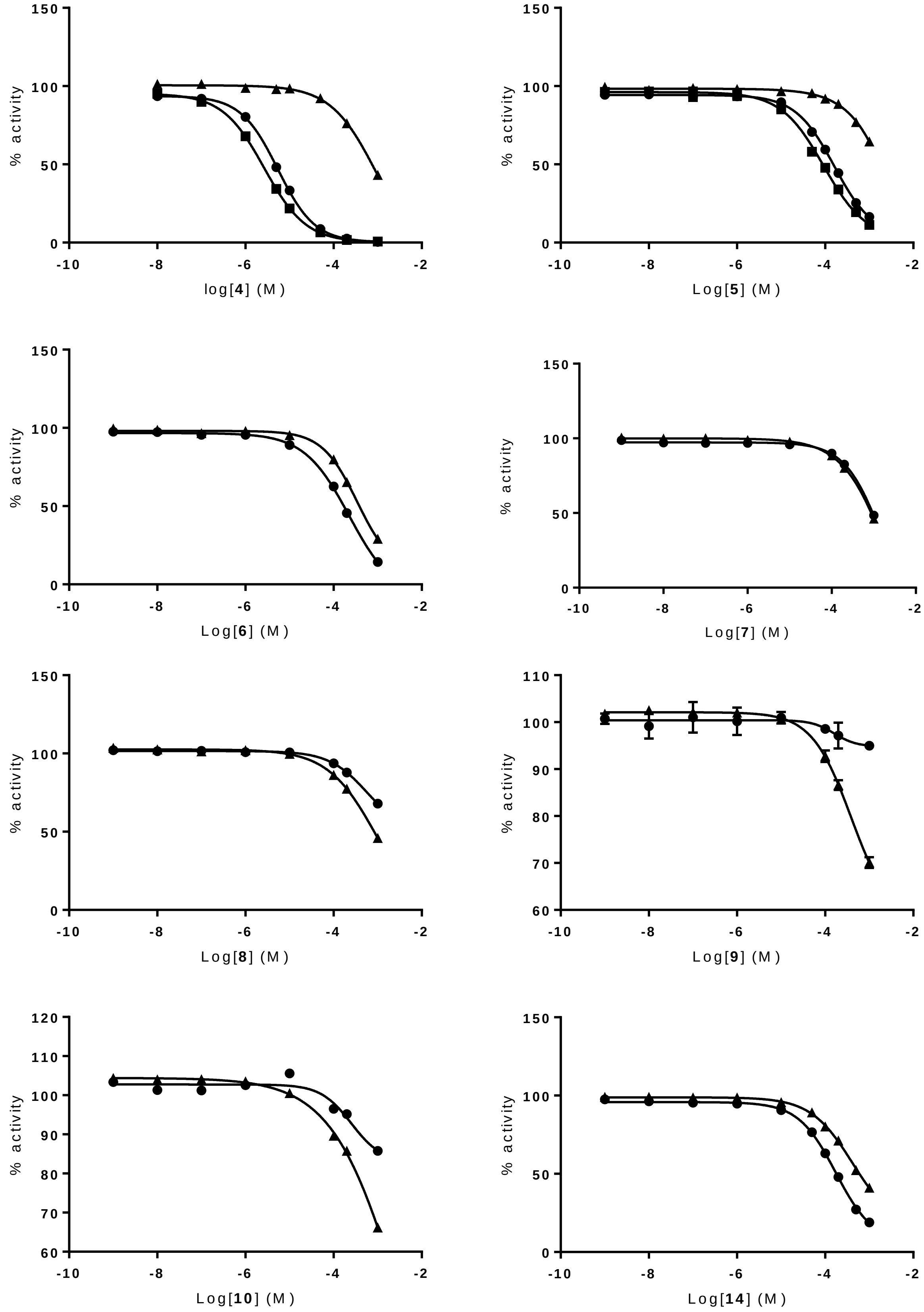

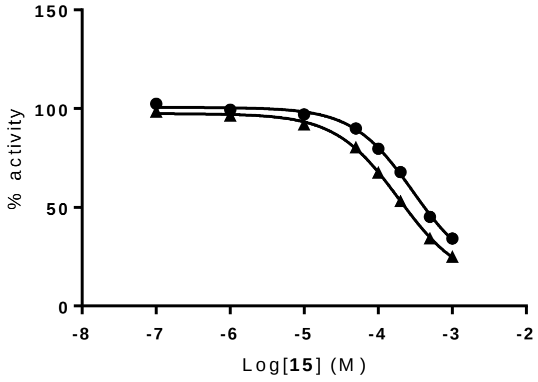
% activity
Log[15] (M)

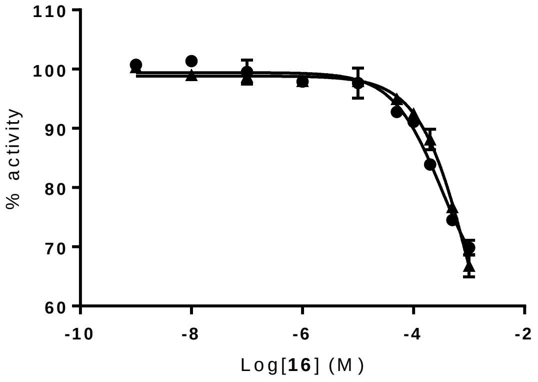
% activity
Log[16] (M)

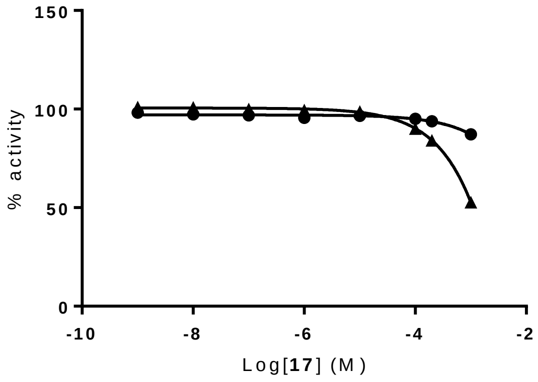
% activity
Log[17] (M)

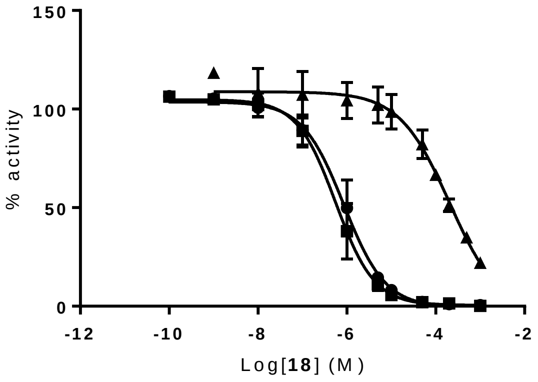
% activity
Log[18] (M)

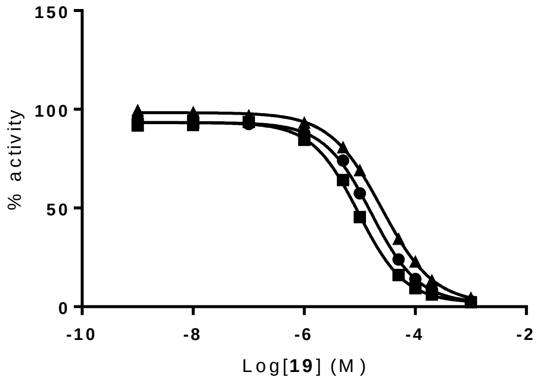
% activity
Log[19] (M)

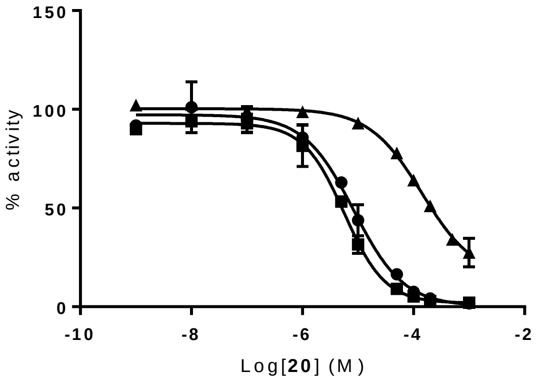
% activity
Log[20] (M)

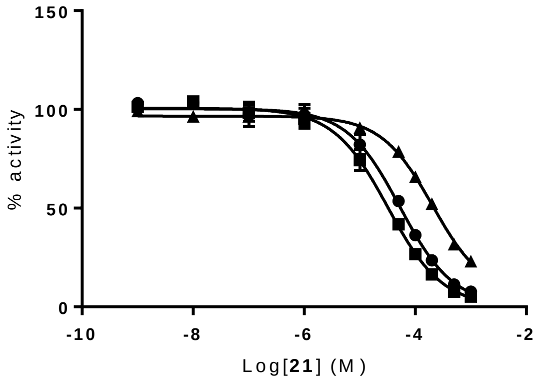
% activity
Log[21] (M)

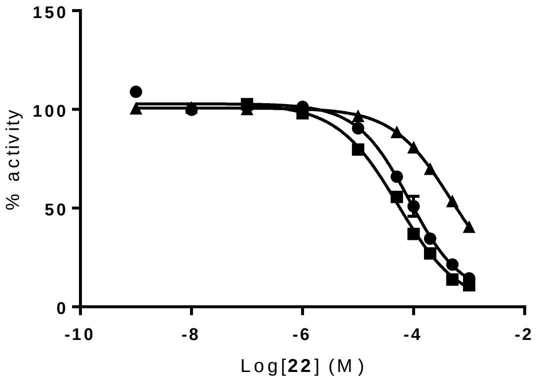
% activity
Log[22] (M)

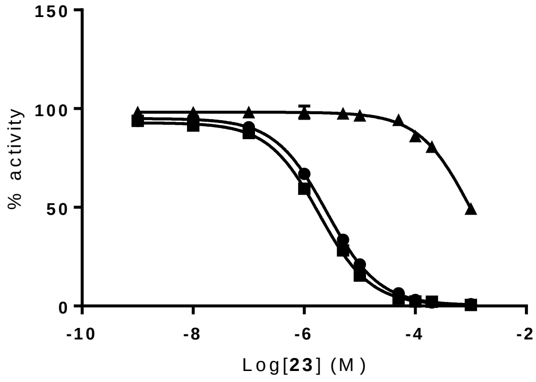
150
100
50
0
% activity
-10
-8
-6
-4
-2
Log[23] (M)

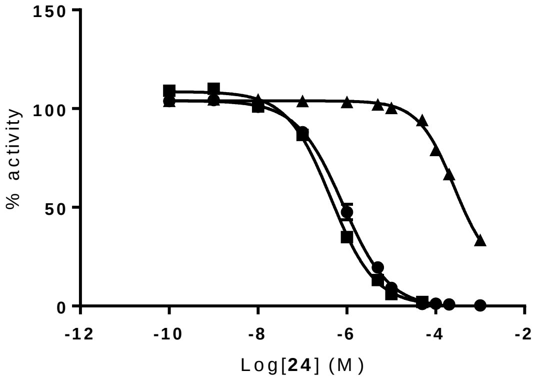
150
100
50
0
% activity
-12
-10
-8
-6
-4
-2
Log[24] (M)

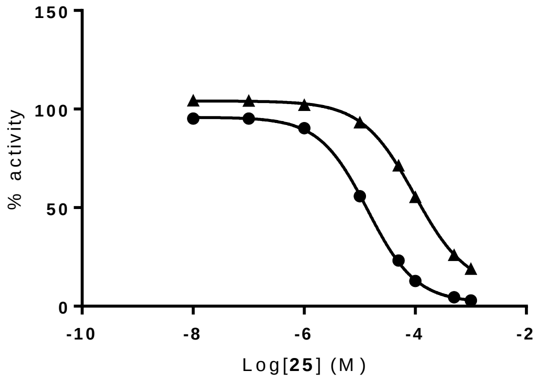
150
100
50
0
% activity
-10
-8
-6
-4
-2
Log[25] (M)

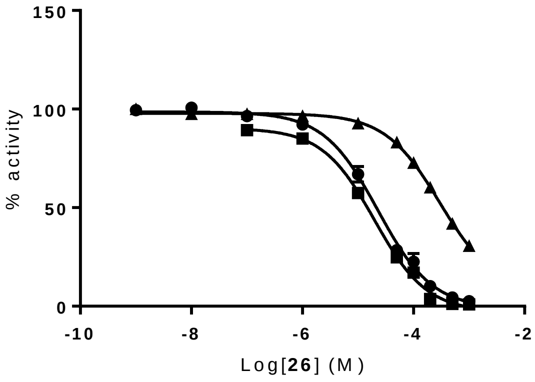
150
100
50
0
% activity
-10
-8
-6
-4
-2
Log[26] (M)

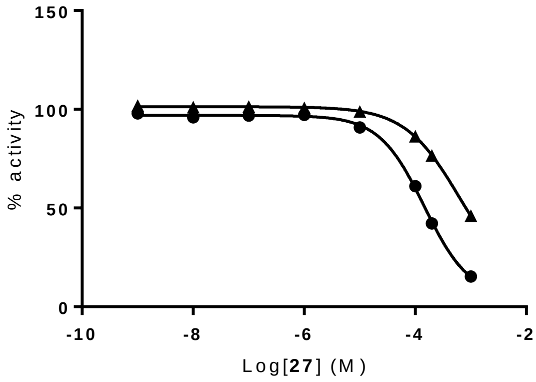
150
100
50
0
% activity
-10
-8
-6
-4
-2
Log[27] (M)

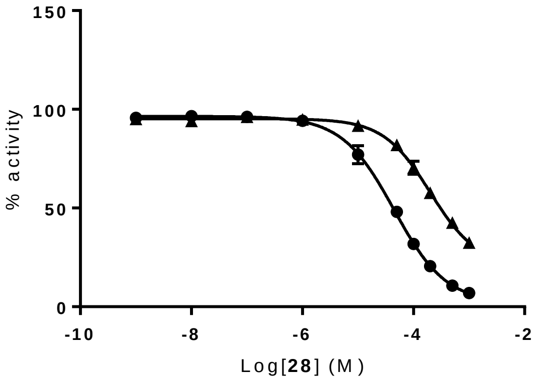
150
100
50
0
% activity
-10
-8
-6
-4
-2
Log[28] (M)

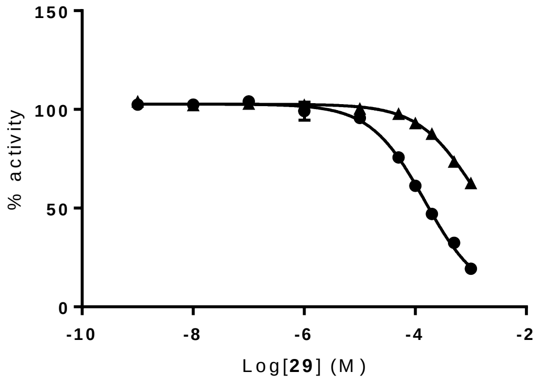
150
100
50
0
% activity
-10
-8
-6
-4
-2
Log[29] (M)

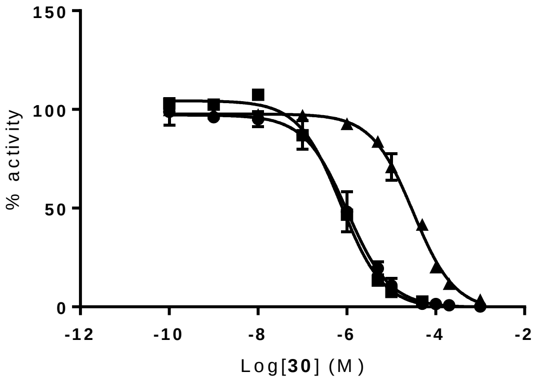
150
100
50
0
% activity
-12
-10
-8
-6
-4
-2
Log[30] (M)

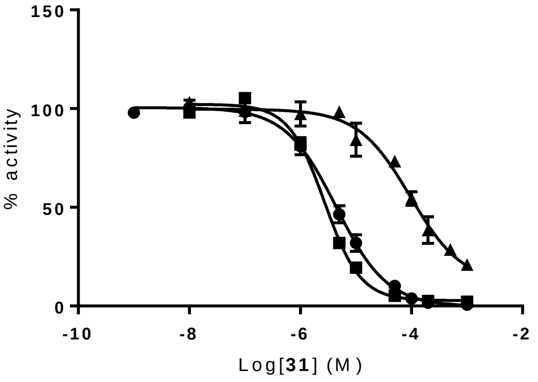
150
100
50
0
% activity
-10
-8
-6
-4
-2
Log[31] (M)

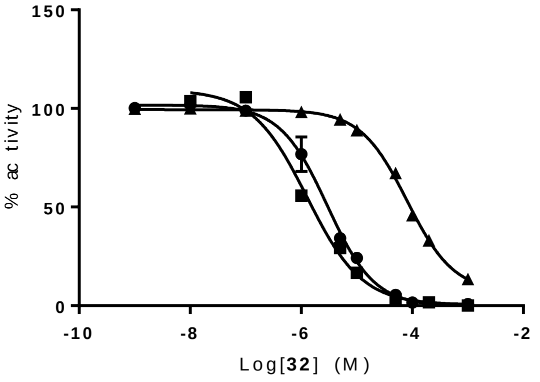
150
100
50
0
% ac tivity
-10
-8
-6
-4
-2
Log[32] (M)

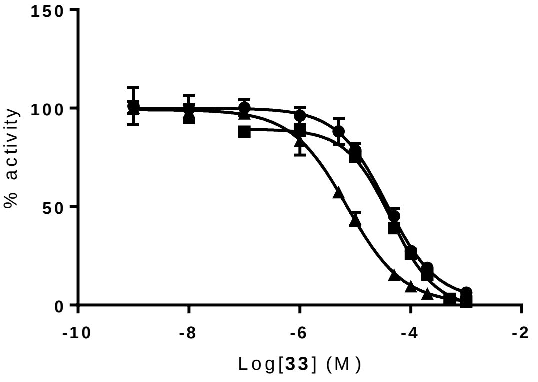
150
100
50
0
% activity
-10
-8
-6
-4
-2
Log[33] (M)

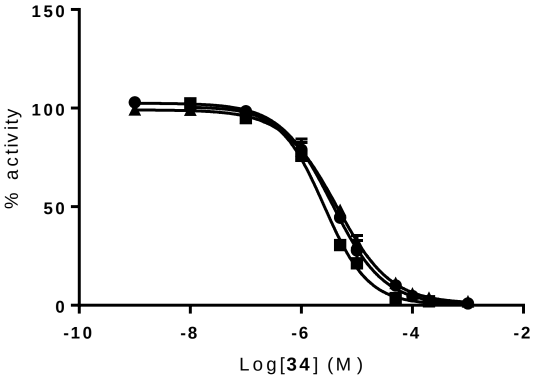
150
100
50
0
% activity
-10
-8
-6
-4
-2
Log[34] (M)

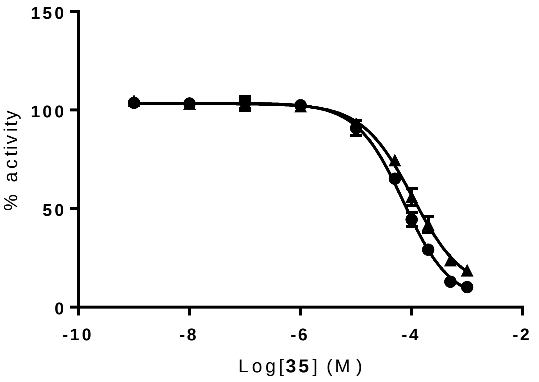
150
100
50
0
% activity
-10
-8
-6
-4
-2
Log[35] (M)

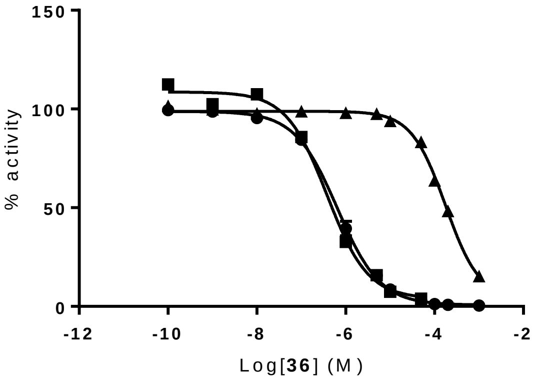
150
100
50
0
% activity
-12
-10
-8
-6
-4
-2
Log[36] (M)

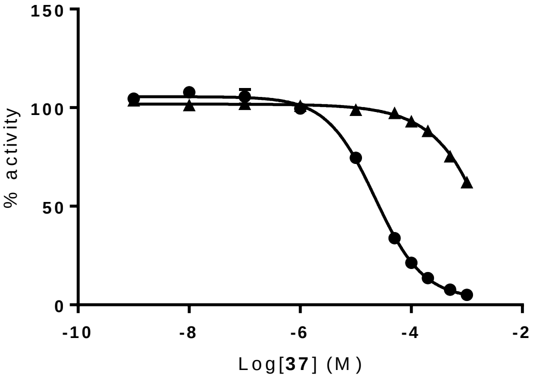
150
100
50
0
% activity
-10
-8
-6
-4
-2
Log[37] (M)

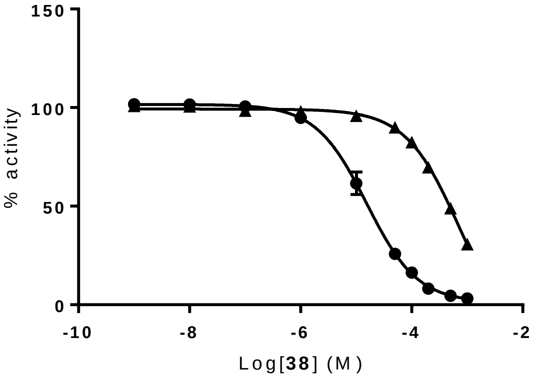
150
100
50
0
% activity
-10
-8
-6
-4
-2
Log[38] (M)

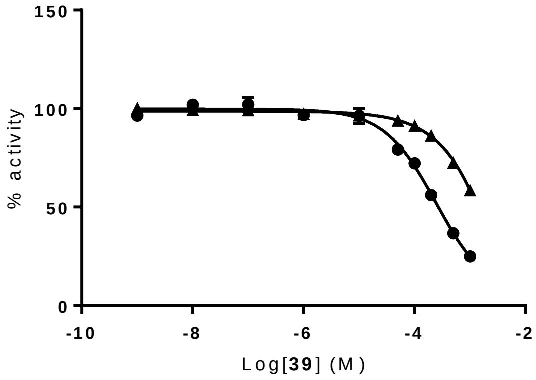
150
100
50
0
% activity
-10
-8
-6
-4
-2
Log[39] (M)

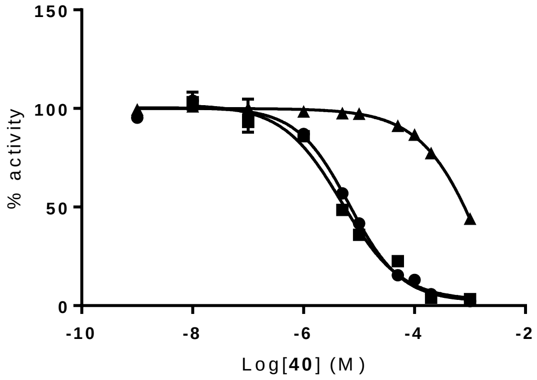
150
100
50
0
% activity
-10
-8
-6
-4
-2
Log[40] (M)

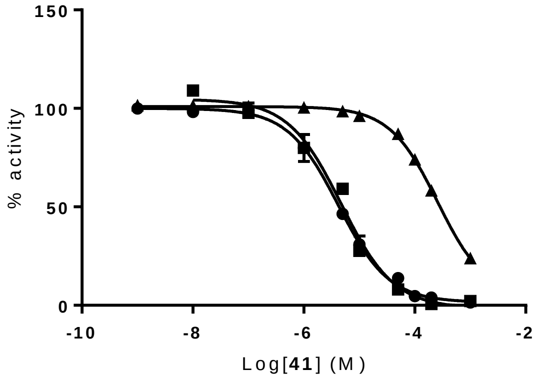
150
100
50
0
% activity
-10
-8
-6
-4
-2
Log[41] (M)

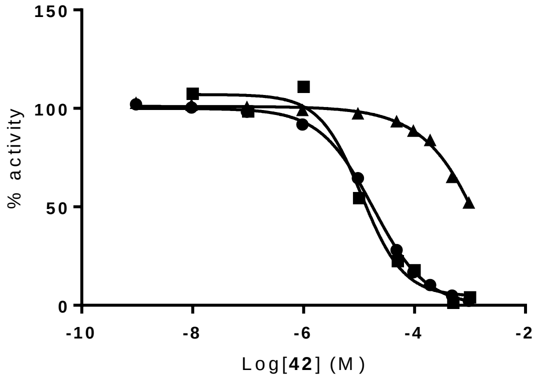
150
100
50
0
% activity
-10
-8
-6
-4
-2
Log[42] (M)

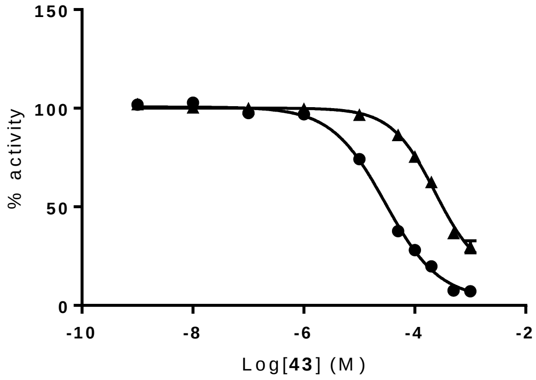
150
100
50
0
% activity
-10
-8
-6
-4
-2
Log[43] (M)

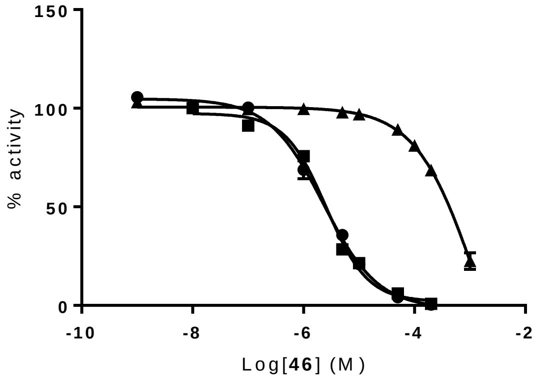
150
100
50
0
% activity
-10
-8
-6
-4
-2
Log[46] (M)

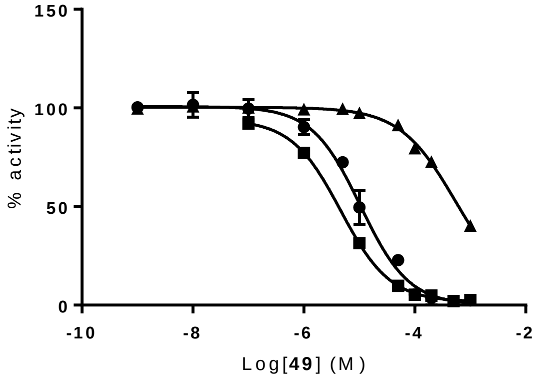
150
100
50
0
% activity
-10
-8
-6
-4
-2
Log[49] (M)

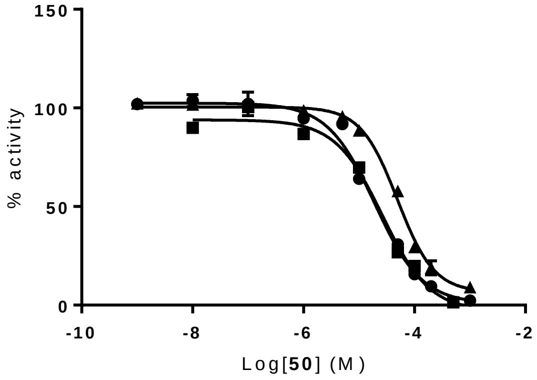
150
100
50
0
% activity
-10
-8
-6
-4
-2
Log[50] (M)

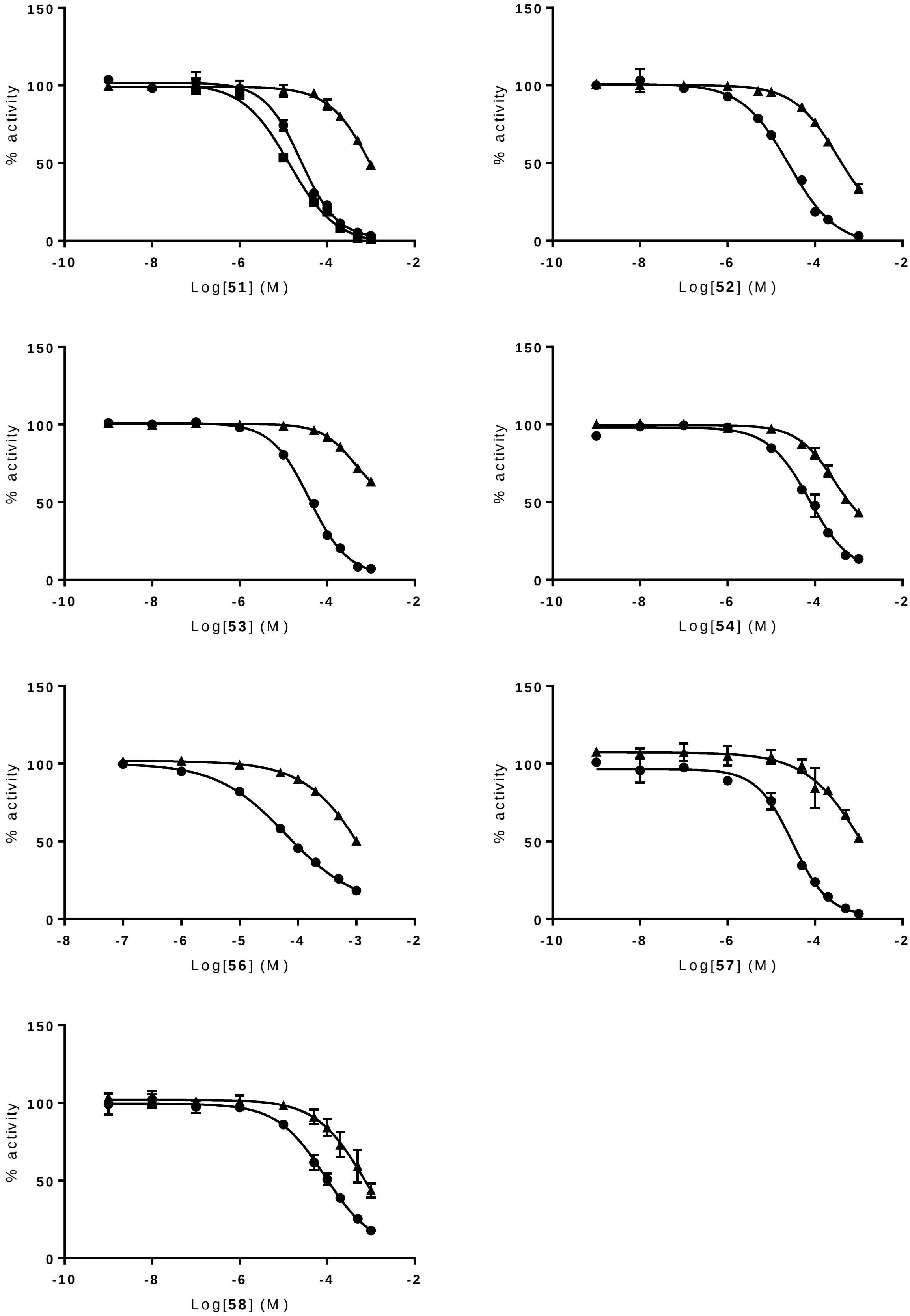

Figure S3. Dose response IC_{50} curves against *Ag*AChE1 (circular dots), *Aa*AChE1 (square dots), and *h*AChE (triangular dots).

Dose response IC_{50} curves G122S-*Ag*AChE1:

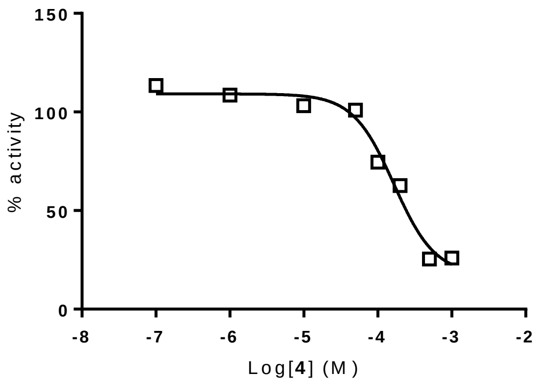

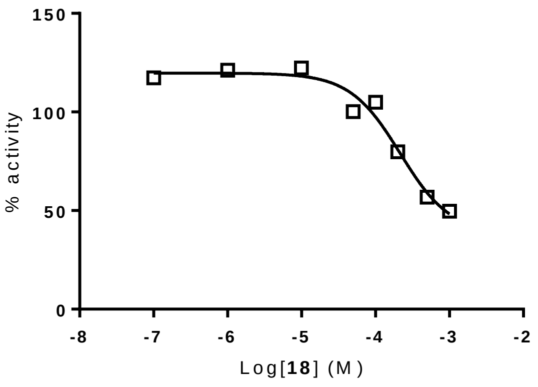

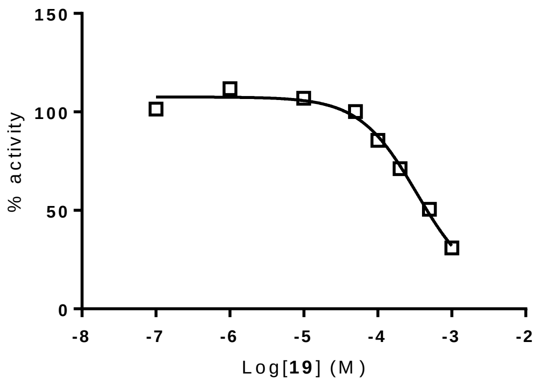

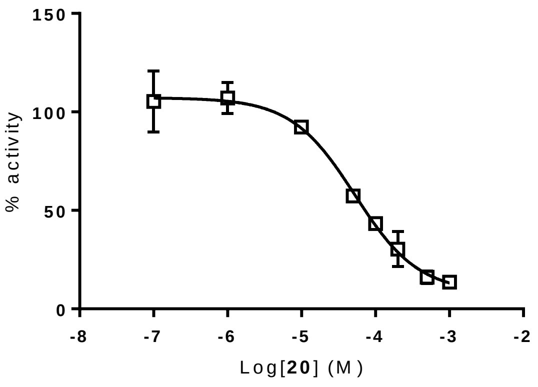

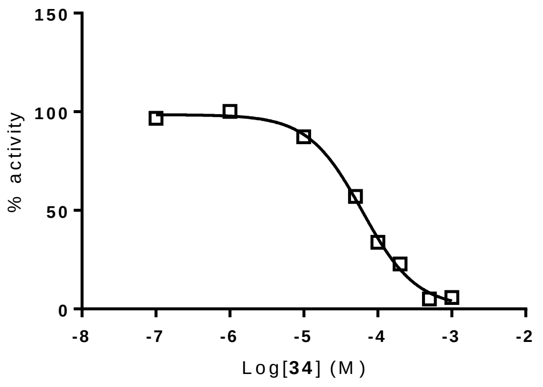

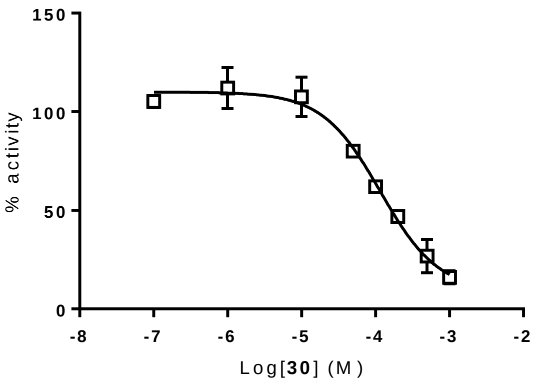

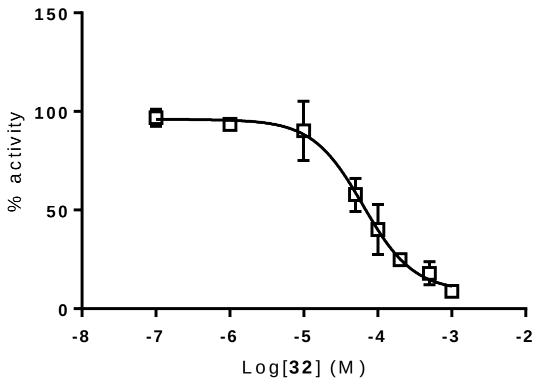

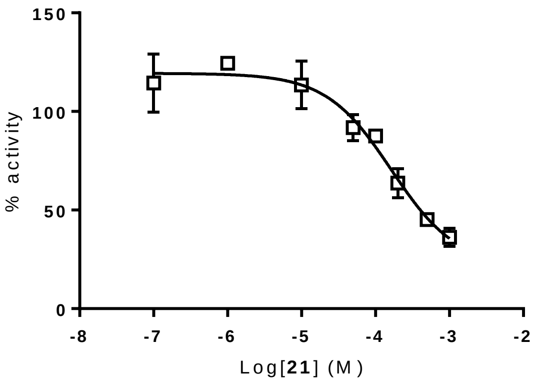

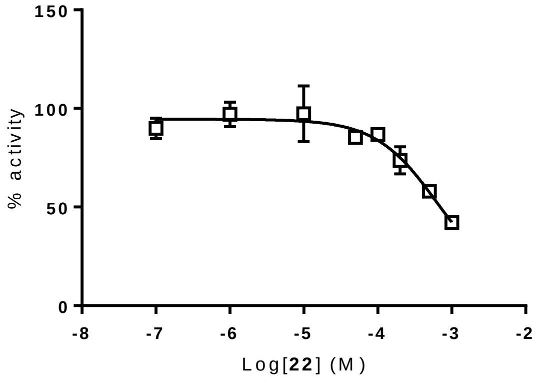
150
100
50
0
% activity
-8 -7 -6 -5 -4 -3 -2
Log[22] (M)

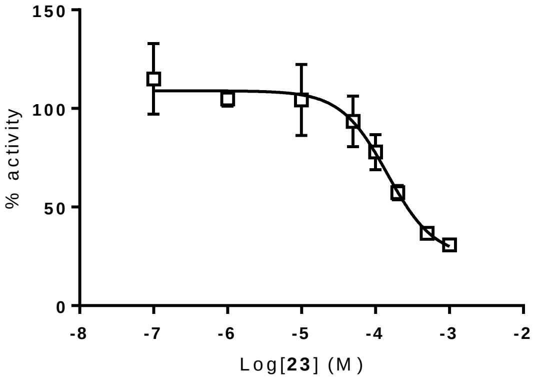
150
100
50
0
% activity
-8 -7 -6 -5 -4 -3 -2
Log[23] (M)

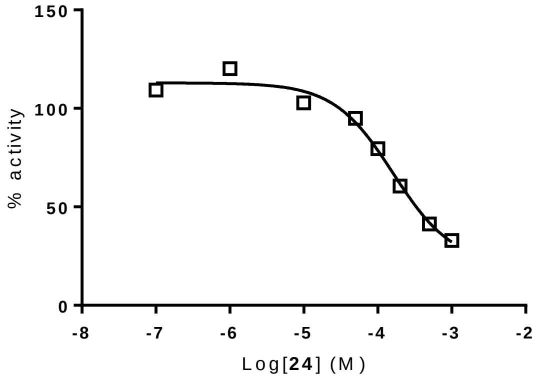
150
100
50
0
% activity
-8 -7 -6 -5 -4 -3 -2
Log[24] (M)

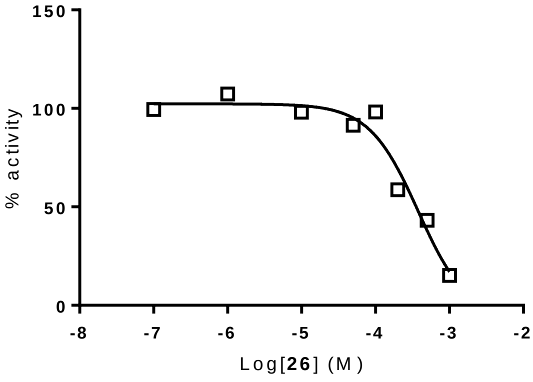
150
100
50
0
% activity
-8 -7 -6 -5 -4 -3 -2
Log[26] (M)

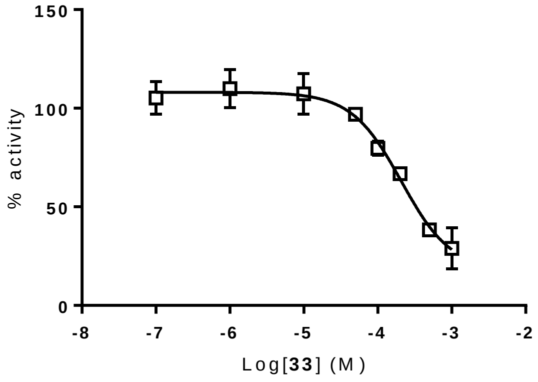
150
100
50
0
% activity
-8 -7 -6 -5 -4 -3 -2
Log[33] (M)

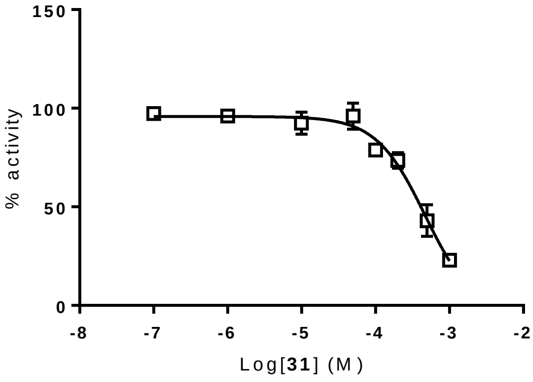
150
100
50
0
% activity
-8 -7 -6 -5 -4 -3 -2
Log[31] (M)

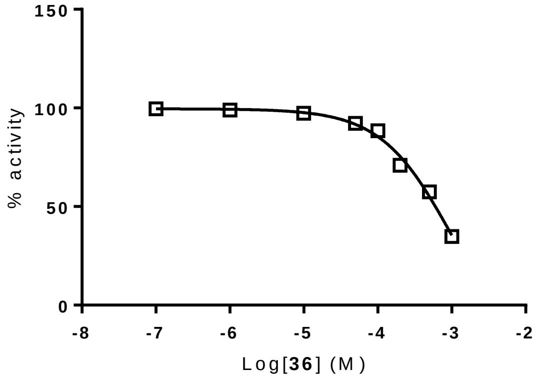
150
100
50
0
% activity
-8 -7 -6 -5 -4 -3 -2
Log[36] (M)

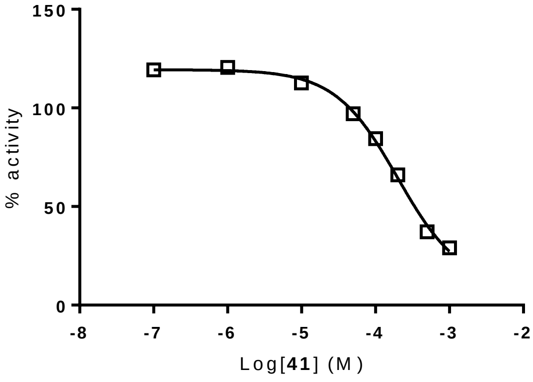
150
100
50
0
% activity
-8 -7 -6 -5 -4 -3 -2
Log[41] (M)

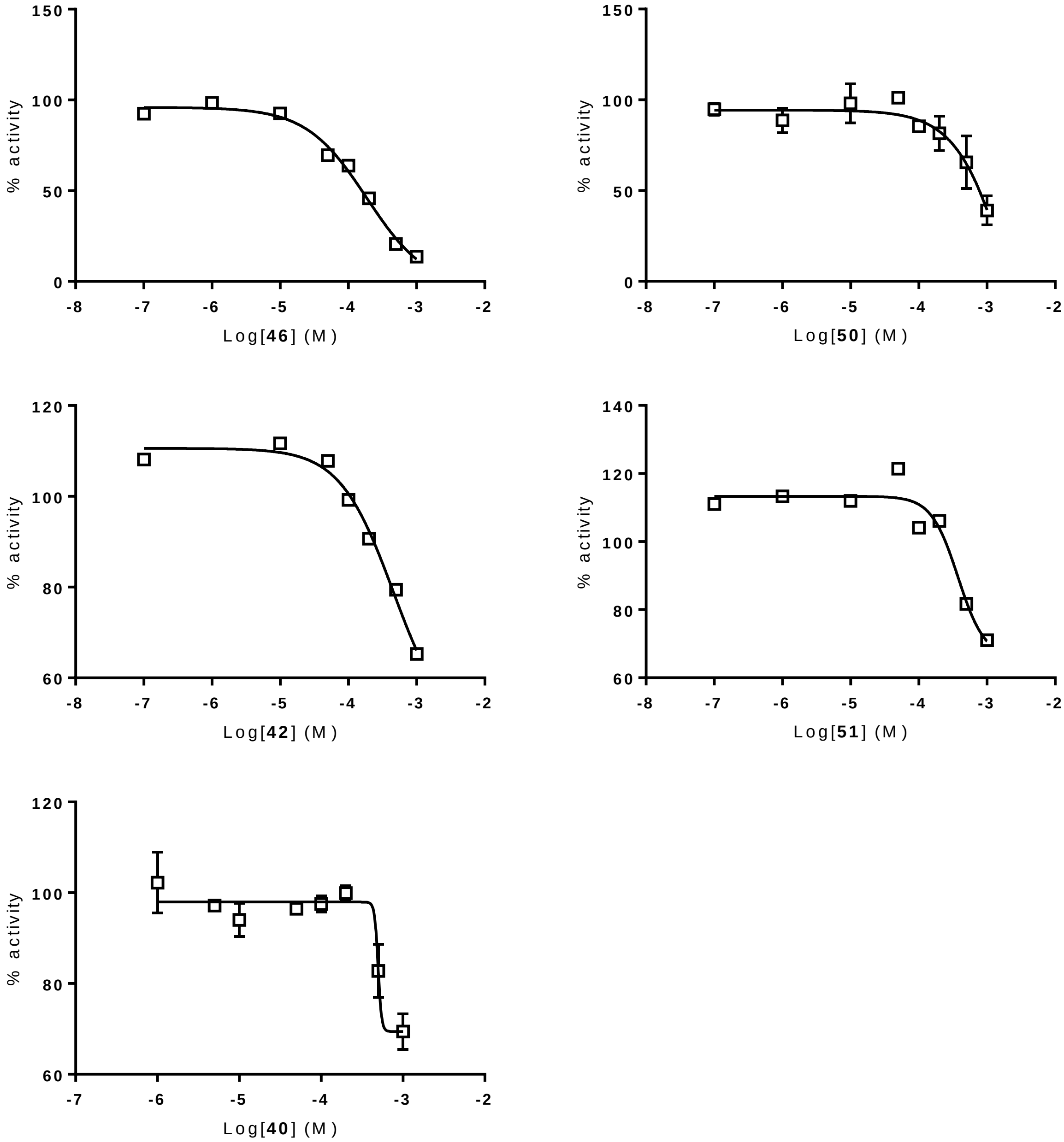

Figure S4. Dose response IC_{50} curves for G122S-*Ag*AChE1.

In vivo raw data

Table S5. In vivo raw data for compound **20** as insecticide towards *An. gambiae* at a dose of 10 nmol/mosquito.

Compound	Dose	Exp. number	No. of Mosquitoes	Mortality			
	(nmol)			24 h Mortality	% 24 h Mortality	48 h Mortality	% 48 h Mortality
Acetone	Control	1	5	0	0.00	0	0.00
		2	5	0	0.00	0	0.00
		3	5	1	20.00	1	20.00
		4	5	0	0.00	0	0.00
		5	5	0	0.00	0	0.00
		6	5	0	0.00	0	0.00
		7	5	0	0.00	0	0.00
		8	5	0	0.00	0	0.00
		9	5	0	0.00	0	0.00
		10	5	0	0.00	1	20.00
		**Sum**	**50**	**1**	**2.00**	**2**	**4.00**
**20**	10 nmol	1	5	5	100.00	5	100.00
		2	5	5	100.00	5	100.00
		3	5	5	100.00	5	100.00
		4	5	5	100.00	5	100.00
		5	5	5	100.00	5	100.00
		6	5	5	100.00	5	100.00
		7	5	5	100.00	5	100.00
		8	5	5	100.00	5	100.00
		9	5	5	100.00	5	100.00
		10	5	5	100.00	5	100.00
		11	5	5	100.00	5	100.00
		12	5	5	100.00	5	100.00
		13	5	5	100.00	5	100.00
		14	5	5	100.00	5	100.00
		15	5	5	100.00	5	100.00
		16	5	5	100.00	5	100.00
		17	5	5	100.00	5	100.00
		18	5	5	100.00	5	100.00
		19	5	5	100.00	5	100.00
		20	5	5	100.00	5	100.00
		**Sum**	**100**	**100**	**100.00**	**100**	**100.00**

Table S6. In vivo raw data for compound **20** as insecticide towards *An. gambiae* at a dose of 1 nmol/mosquito.

Compound	Dose	Exp. number	No. of Mosquitoes	Mortality			
	(nmol)			24 h Mortality	% 24 h Mortality	48 h Mortality	% 48 h Mortality
Acetone	Control	1	5	0	0.00	0	0.00
		2	5	0	0.00	0	0.00
		3	5	0	0.00	0	0.00
		4	5	0	0.00	0	0.00
		5	5	0	0.00	0	0.00
		6	5	0	0.00	0	0.00
		7	5	0	0.00	0	0.00
		8	5	0	0.00	0	0.00
		9	5	0	0.00	0	0.00
		10	5	0	0.00	0	0.00
		**Sum**	**50**	**0**	**0.00**	**0**	**0.00**
**20**	1 nmol	1	5	3	60.00	3	60.00
		2	5	3	60.00	3	60.00
		3	5	2	40.00	2	40.00
		4	5	2	40.00	2	40.00
		5	5	3	60.00	3	60.00
		6	5	4	80.00	4	80.00
		7	5	5	100.00	5	100.00
		8	5	4	80.00	4	80.00
		9	5	3	60.00	3	60.00
		10	5	4	80.00	4	80.00
		11	5	5	100.00	5	100.00
		12	5	2	40.00	2	40.00
		13	5	3	60.00	3	60.00
		14	5	3	60.00	3	60.00
		15	5	3	60.00	3	60.00
		16	5	3	60.00	3	60.00
		17	5	4	80.00	4	80.00
		18	5	5	100.00	5	100.00
		19	5	2	40.00	2	40.00
		20	5	3	60.00	3	60.00
		**Sum**	**100**	**66**	**66.00**	**66**	**66.00**

Table S7. In vivo raw data for compound **20** as insecticide towards *An. gambiae* at a dose of 0.1 nmol/mosquito.

Compound	Dose	Exp. number	No. of Mosquitoes	Mortality			
	(nmol)			24 h Mortality	% 24 h Mortality	48 h Mortality	% 48 h Mortality
Acetone	Control	1	5	0	0.00	0	0.00
		2	5	0	0.00	0	0.00
		3	5	0	0.00	0	0.00
		4	5	0	0.00	0	0.00
		5	5	0	0.00	0	0.00
		6	5	0	0.00	0	0.00
		7	5	0	0.00	0	0.00
		8	5	0	0.00	0	0.00
		9	5	0	0.00	0	0.00
		10	5	0	0.00	0	0.00
		**Sum**	**50**	**0**	**0.00**	**0**	**0.00**
**20**	0.1 nmol	1	5	1	20.00	1	20.00
		2	5	2	40.00	2	40.00
		3	5	0	0.00	0	0.00
		4	5	2	40.00	2	40.00
		5	5	1	20.00	1	20.00
		6	5	2	40.00	2	40.00
		7	5	2	40.00	2	40.00
		8	5	2	40.00	2	40.00
		9	5	1	20.00	1	20.00
		10	5	2	40.00	2	40.00
		11	5	0	0.00	0	0.00
		12	5	0	0.00	0	0.00
		13	5	0	0.00	0	0.00
		14	5	0	0.00	1	20.00
		15	5	1	20.00	1	20.00
		16	5	2	40.00	2	40.00
		17	5	0	0.00	0	0.00
		18	5	2	40.00	2	40.00
		19	5	0	0.00	1	20.00
		20	5	0	0.00	0	0.00
		**Sum**	**100**	**20**	**20.00**	**22**	**22.00**

Table S8. In vivo raw data for compound **24** as insecticide towards *An. gambiae* at a dose of 10 nmol/mosquito.

Compound	Dose	Exp. number	No. of Mosquitoes	Mortality			
	(nmol)			24 h Mortality	% 24 h Mortality	48 h Mortality	% 48 h Mortality
Acetone	Control	1	5	0	0.00	0	0.00
		2	5	0	0.00	0	0.00
		3	5	0	0.00	0	0.00
		4	5	0	0.00	0	0.00
		5	5	0	0.00	0	0.00
		6	5	0	0.00	0	0.00
		7	5	0	0.00	0	0.00
		8	5	0	0.00	0	0.00
		**Sum**	**40**	**0**	**0.00**	**0**	**0.00**
**24**	10 nmol	1	5	5	100.00	5	100.00
		2	5	5	100.00	5	100.00
		3	5	5	100.00	5	100.00
		4	5	5	100.00	5	100.00
		5	5	5	100.00	5	100.00
		6	5	5	100.00	5	100.00
		7	5	5	100.00	5	100.00
		8	5	5	100.00	5	100.00
		9	5	5	100.00	5	100.00
		10	5	5	100.00	5	100.00
		11	5	5	100.00	5	100.00
		12	5	5	100.00	5	100.00
		13	5	5	100.00	5	100.00
		14	5	5	100.00	5	100.00
		15	5	5	100.00	5	100.00
		16	5	5	100.00	5	100.00
		17	5	5	100.00	5	100.00
		18	5	5	100.00	5	100.00
		19	5	5	100.00	5	100.00
		20	5	5	100.00	5	100.00
		**Sum**	**100**	**100**	**100.00**	**100**	**100.00**

Table S9. In vivo raw data for compound **24** as insecticide towards *An. gambiae* at a dose of 1 nmol/mosquito.

Compound	Dose	Exp. number	No. of Mosquitoes	Mortality			
	(nmol)			24 h Mortality	% 24 h Mortality	48 h Mortality	% 48 h Mortality
Acetone	Control	1	5	0	0.00	0	0.00
		2	5	0	0.00	0	0.00
		3	5	0	0.00	0	0.00
		4	5	0	0.00	0	0.00
		5	5	0	0.00	0	0.00
		6	5	0	0.00	0	0.00
		7	5	0	0.00	0	0.00
		8	5	0	0.00	0	0.00
		9	5	0	0.00	0	0.00
		10	5	0	0.00	0	0.00
		**Sum**	**50**	**0**	**0.00**	**0**	**0.00**
**24**	1 nmol	1	5	2	40.00	2	40.00
		2	5	2	40.00	2	40.00
		3	5	1	20.00	1	20.00
		4	5	2	40.00	2	40.00
		5	5	2	40.00	2	40.00
		6	5	2	40.00	2	40.00
		7	5	2	40.00	2	40.00
		8	5	2	40.00	2	40.00
		9	5	2	40.00	2	40.00
		10	5	2	40.00	2	40.00
		11	5	2	40.00	3	60.00
		12	5	2	40.00	2	40.00
		13	5	4	80.00	4	80.00
		14	5	1	20.00	1	20.00
		15	5	0	0.00	0	0.00
		16	5	2	40.00	2	40.00
		17	5	2	40.00	2	40.00
		18	5	2	40.00	3	60.00
		19	5	2	40.00	3	60.00
		20	5	2	40.00	2	40.00
		**Sum**	**100**	**38**	**38.00**	**41**	**41.00**

Table S10. In vivo raw data for compound **24** as insecticide towards *An. gambiae* at a dose of 0.1 nmol/mosquito.

Compound	Dose	Exp. number	No. of Mosquitoes	Mortality			
	(nmol)			24 h Mortality	% 24 h Mortality	48 h Mortality	% 48 h Mortality
Acetone	Control	1	5	0	0.00	0	0.00
		2	5	0	0.00	0	0.00
		3	5	0	0.00	0	0.00
		4	5	0	0.00	0	0.00
		5	5	0	0.00	0	0.00
		6	5	0	0.00	0	0.00
		7	5	0	0.00	0	0.00
		8	5	0	0.00	0	0.00
		9	5	0	0.00	0	0.00
		10	5	0	0.00	0	0.00
		**Sum**	**50**	**0**	**0.00**	**0**	**0.00**
**24**	0.1 nmol	1	5	0	0.00	0	0.00
		2	5	0	0.00	0	0.00
		3	5	0	0.00	0	0.00
		4	5	0	0.00	0	0.00
		5	5	0	0.00	0	0.00
		6	5	1	20.00	1	20.00
		7	5	1	20.00	1	20.00
		8	5	0	0.00	0	0.00
		9	5	0	0.00	0	0.00
		10	5	2	40.00	2	40.00
		11	5	0	0.00	0	0.00
		12	5	1	20.00	1	20.00
		13	5	1	20.00	1	20.00
		14	5	0	0.00	0	0.00
		15	5	0	0.00	0	0.00
		16	5	0	0.00	0	0.00
		17	5	0	0.00	0	0.00
		18	5	1	20.00	1	20.00
		19	5	0	0.00	0	0.00
		20	5	0	0.00	0	0.00
		21	5	2	40.00	2	40.00
		**Sum**	**105**	**9**	**8.57**	**9**	**8.57**

Table S11. In vivo raw data for compound **30** as insecticide towards *An. gambiae* at a dose of 10 nmol/mosquito.

Compound	Dose	Exp. number	No. of Mosquitoes	Mortality			
	(nmol)			24 h Mortality	% 24 h Mortality	48 h Mortality	% 48 h Mortality
Acetone	Control	1	5	0	0.00	0	0.00
		2	5	0	0.00	0	0.00
		3	5	0	0.00	0	0.00
		4	5	0	0.00	0	0.00
		5	5	0	0.00	0	0.00
		6	5	0	0.00	0	0.00
		7	5	0	0.00	0	0.00
		8	5	0	0.00	0	0.00
		9	5	0	0.00	0	0.00
		10	5	0	0.00	0	0.00
		**Sum**	**50**	**0**	**0.00**	**0**	**0.00**
**30**	10 nmol	1	5	5	100.00	5	100.00
		2	5	5	100.00	5	100.00
		3	5	5	100.00	5	100.00
		4	5	5	100.00	5	100.00
		5	5	5	100.00	5	100.00
		6	5	5	100.00	5	100.00
		7	5	5	100.00	5	100.00
		8	5	5	100.00	5	100.00
		9	5	5	100.00	5	100.00
		10	5	5	100.00	5	100.00
		11	5	5	100.00	5	100.00
		12	5	5	100.00	5	100.00
		13	5	5	100.00	5	100.00
		14	5	5	100.00	5	100.00
		15	5	5	100.00	5	100.00
		16	5	5	100.00	5	100.00
		17	5	5	100.00	5	100.00
		18	5	5	100.00	5	100.00
		19	5	5	100.00	5	100.00
		20	5	5	100.00	5	100.00
		**Sum**	**100**	**100**	**100.00**	**100**	**100.00**

Table S12. In vivo raw data for compound **30** as insecticide towards *An. gambiae* at a dose of 1 nmol/mosquito.

Compound	Dose	Exp. number	No. of Mosquitoes	Mortality			
	(nmol)			24 h Mortality	% 24 h Mortality	48 h Mortality	% 48 h Mortality
Acetone	Control	1	5	0	0.00	0	0.00
		2	5	0	0.00	0	0.00
		3	5	0	0.00	0	0.00
		4	5	0	0.00	0	0.00
		5	5	0	0.00	0	0.00
		6	5	0	0.00	0	0.00
		7	5	0	0.00	0	0.00
		8	5	0	0.00	0	0.00
		9	5	0	0.00	0	0.00
		10	5	0	0.00	0	0.00
		**Sum**	**50**	**0**	**0.00**	**0**	**0.00**
**30**	1 nmol	1	5	1	20.00	1	20.00
		2	5	4	80.00	4	80.00
		3	5	3	60.00	3	60.00
		4	5	2	40.00	2	40.00
		5	5	4	80.00	4	80.00
		6	5	0	0.00	0	0.00
		7	5	4	80.00	4	80.00
		8	5	4	80.00	4	80.00
		9	5	3	60.00	4	80.00
		10	5	5	100.00	5	100.00
		11	5	3	60.00	3	60.00
		12	5	4	80.00	4	80.00
		13	5	5	100.00	5	100.00
		14	5	1	20.00	2	40.00
		15	5	3	60.00	3	60.00
		16	5	2	40.00	2	40.00
		17	5	5	100.00	5	100.00
		18	5	1	20.00	1	20.00
		19	5	3	60.00	3	60.00
		20	5	5	100.00	5	100.00
		**Sum**	**100**	**62**	**62.00**	**64**	**64.00**

Table S13. In vivo raw data for compound **30** as insecticide towards *An. gambiae* at a dose of 0.1 nmol/mosquito.

Compound	Dose	Exp. number	No. of Mosquitoes	Mortality			
	(nmol)			24 h Mortality	% 24 h Mortality	48 h Mortality	% 48 h Mortality
Acetone	Control	1	5	0	0.00	0	0.00
		2	5	0	0.00	0	0.00
		3	5	0	0.00	0	0.00
		4	5	0	0.00	0	0.00
		5	5	0	0.00	0	0.00
		6	5	1	20.00	0	0.00
		7	5	0	0.00	0	0.00
		8	5	0	0.00	0	0.00
		9	5	0	0.00	0	0.00
		10	5	0	0.00	0	0.00
		**Sum**	**50**	**1**	**2.00**	**0**	**0.00**
**30**	0.1 nmol	1	5	0	0.00	1	20.00
		2	5	0	0.00	1	20.00
		3	5	0	0.00	1	20.00
		4	5	0	0.00	2	40.00
		5	5	0	0.00	0	0.00
		6	5	0	0.00	0	0.00
		7	5	0	0.00	0	0.00
		8	5	1	20.00	1	20.00
		9	5	0	0.00	0	0.00
		10	5	1	20.00	1	20.00
		11	5	0	0.00	0	0.00
		12	5	0	0.00	0	0.00
		13	5	1	20.00	1	20.00
		14	5	0	0.00	0	0.00
		15	5	1	20.00	1	20.00
		16	5	0	0.00	0	0.00
		17	5	0	0.00	0	0.00
		18	5	1	20.00	1	20.00
		19	5	0	0.00	0	0.00
		20	5	1	20.00	1	20.00
		**Sum**	**100**	**6**	**6.00**	**11**	**11.00**

Table S14. In vivo raw data for compound **20** as insecticide towards *Ae. aegypti* at a dose of 10 nmol/mosquito.

Compound	Dose	Exp. number	No. of Mosquitoes	Mortality			
	(nmol)			24 h Mortality	% 24 h Mortality	48 h Mortality	% 48 h Mortality
Acetone	Control	1	5	0	0.00	0	0.00
		2	5	0	0.00	0	0.00
		3	5	0	0.00	0	0.00
		4	5	0	0.00	0	0.00
		5	5	0	0.00	0	0.00
		6	5	0	0.00	0	0.00
		7	5	0	0.00	0	0.00
		8	5	2	40.00	2	40.00
		9	5	0	0.00	0	0.00
		10	5	0	0.00	0	0.00
		**Sum**	**50**	**2**	**4.00**	**2**	**4.00**
**20**	10 nmol	1	5	5	100.00	5	100.00
		2	5	5	100.00	5	100.00
		3	5	5	100.00	5	100.00
		4	5	5	100.00	5	100.00
		5	5	5	100.00	5	100.00
		6	5	5	100.00	5	100.00
		7	5	5	100.00	5	100.00
		8	5	5	100.00	5	100.00
		9	5	5	100.00	5	100.00
		10	5	5	100.00	5	100.00
		11	5	5	100.00	5	100.00
		12	5	5	100.00	5	100.00
		13	5	5	100.00	5	100.00
		14	5	5	100.00	5	100.00
		15	5	5	100.00	5	100.00
		16	5	5	100.00	5	100.00
		17	5	5	100.00	5	100.00
		18	5	5	100.00	5	100.00
		19	5	5	100.00	5	100.00
		20	5	5	100.00	5	100.00
		**Sum**	**100**	**100**	**100.00**	**100**	**100.00**

Table S15. In vivo raw data for compound **20** as insecticide towards *Ae. aegypti* at a dose of 1 nmol/mosquito.

Compound	Dose	Exp. number	No. of Mosquitoes	Mortality			
	(nmol)			24 h Mortality	% 24 h Mortality	48 h Mortality	% 48 h Mortality
Acetone	Control	1	5	0	0.00	0	0.00
		2	5	0	0.00	0	0.00
		3	5	0	0.00	0	0.00
		4	5	0	0.00	0	0.00
		5	5	0	0.00	0	0.00
		6	5	0	0.00	0	0.00
		7	5	0	0.00	0	0.00
		8	5	0	0.00	0	0.00
		9	5	0	0.00	0	0.00
		10	5	0	0.00	0	0.00
		**Sum**	**50**	**0**	**0.00**	**0**	**0.00**
**20**	1 nmol	1	5	2	40.00	2	40.00
		2	5	1	20.00	1	20.00
		3	5	2	40.00	2	40.00
		4	5	3	60.00	3	60.00
		5	5	2	40.00	2	40.00
		6	5	1	20.00	2	40.00
		7	5	2	40.00	3	60.00
		8	5	3	60.00	3	60.00
		9	5	5	100.00	5	100.00
		10	5	3	60.00	3	60.00
		11	5	3	60.00	4	80.00
		12	5	1	20.00	1	20.00
		13	5	0	0.00	0	0.00
		14	5	3	60.00	3	60.00
		15	5	2	40.00	2	40.00
		16	5	3	60.00	3	60.00
		17	5	3	60.00	3	60.00
		18	5	1	20.00	1	20.00
		19	5	5	100.00	5	100.00
		20	5	3	60.00	3	60.00
		**Sum**	**100**	**48**	**48.00**	**51**	**51.00**

Table S16. In vivo raw data for compound **20** as insecticide towards *Ae. aegypti* at a dose of 0.1 nmol/mosquito.

Compound	Dose	Exp. number	No. of Mosquitoes	Mortality			
	(nmol)			24 h Mortality	% 24 h Mortality	48 h Mortality	% 48 h Mortality
Acetone	Control	1	5	0	0.00	0	0.00
		2	5	0	0.00	0	0.00
		3	5	0	0.00	0	0.00
		4	5	0	0.00	0	0.00
		5	5	0	0.00	0	0.00
		6	5	0	0.00	0	0.00
		**Sum**	**30**	**0**	**0.00**	**0**	**0.00**
**20**	0.1 nmol	1	5	0	0.00	0	0.00
		2	5	0	0.00	0	0.00
		3	5	0	0.00	0	0.00
		4	5	0	0.00	0	0.00
		5	5	0	0.00	0	0.00
		6	5	0	0.00	0	0.00
		7	5	1	20.00	1	20.00
		8	5	0	0.00	0	0.00
		9	5	0	0.00	0	0.00
		10	5	1	20.00	1	20.00
		11	5	0	0.00	0	0.00
		12	5	0	0.00	0	0.00
		13	5	0	0.00	0	0.00
		14	5	0	0.00	0	0.00
		15	5	0	0.00	0	0.00
		16	5	0	0.00	0	0.00
		17	5	0	0.00	0	0.00
		18	5	1	20.00	1	20.00
		19	5	0	0.00	0	0.00
		20	5	1	20.00	1	20.00
		**Sum**	**100**	**4**	**4.00**	**4**	**4.00**

Table S17. In vivo raw data for compound **24** as insecticide towards *Ae. aegypti* at a dose of 10 nmol/mosquito.

**Compound**	**Dose**	**Exp. number**	**No. of Mosquitoes**	**Mortality**			
	**(nmol)**			**24 h Mortality**	**% 24 h Mortality**	**48 h Mortality**	**% 48 h Mortality**
Acetone	Control	1	5	0	0.00	0	0.00
		2	5	0	0.00	0	0.00
		3	5	0	0.00	0	0.00
		4	5	0	0.00	0	0.00
		5	5	0	0.00	0	0.00
		**Sum**	**25**	**0**	**0.00**	**0**	**0.00**
**24**	10 nmol	1	5	5	100.00	5	100.00
		2	5	5	100.00	5	100.00
		3	5	5	100.00	5	100.00
		4	5	5	100.00	5	100.00
		5	5	5	100.00	5	100.00
		6	5	5	100.00	5	100.00
		7	5	5	100.00	5	100.00
		8	5	5	100.00	5	100.00
		9	5	5	100.00	5	100.00
		10	5	5	100.00	5	100.00
		11	5	5	100.00	5	100.00
		12	5	5	100.00	5	100.00
		13	5	5	100.00	5	100.00
		14	5	5	100.00	5	100.00
		15	5	5	100.00	5	100.00
		16	5	5	100.00	5	100.00
		17	5	5	100.00	5	100.00
		18	5	5	100.00	5	100.00
		19	5	5	100.00	5	100.00
		20	5	5	100.00	5	100.00
		**Sum**	**100**	**100**	**100.00**	**100**	**100.00**

Table S18. In vivo raw data for compound **24** as insecticide towards *Ae. aegypti* at a dose of 1 nmol/mosquito.

Compound	Dose	Exp. number	No. of Mosquitoes	Mortality			
	(nmol)			24 h Mortality	% 24 h Mortality	48 h Mortality	% 48 h Mortality
Acetone	Control	1	5	0	0.00	0	0.00
		2	5	0	0.00	0	0.00
		3	5	0	0.00	0	0.00
		4	5	0	0.00	0	0.00
		5	5	0	0.00	0	0.00
		6	5	0	0.00	0	0.00
		7	5	0	0.00	0	0.00
		8	5	0	0.00	0	0.00
		9	5	0	0.00	0	0.00
		10	5	0	0.00	0	0.00
		**Sum**	**50**	**0**	**0.00**	**0**	**0.00**
**24**	1 nmol	1	5	2	40.00	2	40.00
		2	5	3	60.00	3	60.00
		3	5	3	60.00	3	60.00
		4	5	2	40.00	2	40.00
		5	5	2	40.00	2	40.00
		6	5	2	40.00	2	40.00
		7	5	3	60.00	3	60.00
		8	5	4	80.00	4	80.00
		9	5	3	60.00	3	60.00
		10	5	2	40.00	2	40.00
		11	5	2	40.00	2	40.00
		12	5	3	60.00	3	60.00
		13	5	3	60.00	3	60.00
		14	5	3	60.00	3	60.00
		15	5	2	40.00	2	40.00
		16	5	2	40.00	2	40.00
		17	5	1	20.00	1	20.00
		18	5	3	60.00	3	60.00
		19	5	3	60.00	3	60.00
		20	5	2	40.00	2	40.00
		**Sum**	**100**	**50**	**50.00**	**50**	**50.00**

Table S19. In vivo raw data for compound **24** as insecticide towards *Ae. aegypti* at a dose of 0.1 nmol/mosquito.

Compound	Dose	Exp. number	No. of Mosquitoes	Mortality			
	(nmol)			24 h Mortality	% 24 h Mortality	48 h Mortality	% 48 h Mortality
Acetone	Control	1	5	0	0.00	0	0.00
		2	5	0	0.00	0	0.00
		3	5	0	0.00	0	0.00
		4	5	0	0.00	0	0.00
		5	5	0	0.00	0	0.00
		6	5	0	0.00	0	0.00
		**Sum**	**30**	**0**	**0.00**	**0**	**0.00**
**24**	0.1 nmol	1	5	0	0.00	0	0.00
		2	5	0	0.00	0	0.00
		3	5	1	20.00	1	20.00
		4	5	0	0.00	0	0.00
		5	5	0	0.00	0	0.00
		6	5	0	0.00	0	0.00
		7	5	0	0.00	0	0.00
		8	5	0	0.00	0	0.00
		9	5	1	20.00	1	20.00
		10	5	0	0.00	0	0.00
		11	5	0	0.00	0	0.00
		12	5	0	0.00	0	0.00
		13	5	1	20.00	1	20.00
		14	5	0	0.00	0	0.00
		15	5	0	0.00	0	0.00
		16	5	1	20.00	1	20.00
		17	5	0	0.00	0	0.00
		18	5	0	0.00	0	0.00
		19	5	0	0.00	0	0.00
		20	5	0	0.00	0	0.00
		**Sum**	**100**	**4**	**4.00**	**4**	**4.00**

Table S20. In vivo raw data for compound **30** as insecticide towards *Ae. aegypti* at a dose of 10 nmol/mosquito.

Compound	Dose	Exp. number	No. of Mosquitoes	Mortality			
	(nmol)			24 h Mortality	% 24 h Mortality	48 h Mortality	% 48 h Mortality
Acetone	Control	1	5	0	0.00	0	0.00
		2	5	0	0.00	0	0.00
		3	5	0	0.00	0	0.00
		4	5	0	0.00	0	0.00
		5	5	0	0.00	0	0.00
		6	5	0	0.00	0	0.00
		7	5	0	0.00	0	0.00
		8	5	0	0.00	0	0.00
		9	5	0	0.00	0	0.00
		10	5	0	0.00	0	0.00
		**Sum**	**50**	**0**	**0.00**	**0**	**0.00**
**30**	10 nmol	1	5	5	100.00	5	100.00
		2	5	5	100.00	5	100.00
		3	5	5	100.00	5	100.00
		4	5	5	100.00	5	100.00
		5	5	5	100.00	5	100.00
		6	5	4	80.00	4	80.00
		7	5	5	100.00	5	100.00
		8	5	4	80.00	4	80.00
		9	5	4	80.00	4	80.00
		10	5	5	100.00	5	100.00
		11	5	5	100.00	5	100.00
		12	5	5	100.00	5	100.00
		13	5	5	100.00	5	100.00
		14	5	5	100.00	5	100.00
		15	5	5	100.00	5	100.00
		16	5	5	100.00	5	100.00
		17	5	5	100.00	5	100.00
		18	5	5	100.00	5	100.00
		19	5	5	100.00	5	100.00
		20	5	5	100.00	5	100.00
		**Sum**	**100**	**97**	**97.00**	**97**	**97.00**

Table S21. In vivo raw data for compound **30** as insecticide towards *Ae. aegypti* at a dose of 1 nmol/mosquito.

Compound	Dose	Exp. number	No. of Mosquitoes	Mortality			
	(nmol)			24 h Mortality	% 24 h Mortality	48 h Mortality	% 48 h Mortality
Acetone	Control	1	5	0	0.00	0	0.00
		2	5	0	0.00	0	0.00
		3	5	0	0.00	0	0.00
		4	5	0	0.00	0	0.00
		5	5	0	0.00	0	0.00
		**Sum**	**25**	**0**	**0.00**	**0**	**0.00**
**30**	1 nmol	1	5	2	40.00	2	40.00
		2	5	2	40.00	2	40.00
		3	5	3	60.00	3	60.00
		4	5	3	60.00	3	60.00
		5	5	2	40.00	2	40.00
		6	5	2	40.00	2	40.00
		7	5	1	20.00	1	20.00
		8	5	2	40.00	2	40.00
		9	5	2	40.00	2	40.00
		10	5	2	40.00	2	40.00
		11	5	1	20.00	1	20.00
		12	5	0	0.00	0	0.00
		13	5	1	20.00	2	40.00
		14	5	2	40.00	2	40.00
		15	5	2	40.00	2	40.00
		16	5	2	40.00	2	40.00
		17	5	1	20.00	1	20.00
		**Sum**	**85**	**30**	**35.29**	**31**	**36.47**

Table S22. In vivo raw data for compound **30** as insecticide towards *Ae. aegypti* at a dose of 0.1 nmol/mosquito.

Compound	Dose	Exp. number	No. of Mosquitoes	Mortality			
	(nmol)			24 h Mortality	% 24 h Mortality	48 h Mortality	% 48 h Mortality
Acetone	Control	1	5	0	0.00	0	0.00
		2	5	0	0.00	0	0.00
		3	5	0	0.00	0	0.00
		4	5	0	0.00	0	0.00
		5	5	0	0.00	0	0.00
		6	5	0	0.00	0	0.00
		**Sum**	**30**	**0**	**0.00**	**0**	**0.00**
**30**	0.1 nmol	1	5	0	0.00	0	0.00
		2	5	0	0.00	0	0.00
		3	5	1	20.00	1	20.00
		4	5	0	0.00	0	0.00
		5	5	0	0.00	0	0.00
		6	5	0	0.00	0	0.00
		7	5	1	20.00	1	20.00
		8	5	1	20.00	1	20.00
		9	5	0	0.00	0	0.00
		10	5	0	0.00	0	0.00
		11	5	0	0.00	0	0.00
		12	5	0	0.00	0	0.00
		13	5	0	0.00	0	0.00
		14	5	0	0.00	0	0.00
		15	5	0	0.00	0	0.00
		16	5	0	0.00	0	0.00
		17	5	0	0.00	0	0.00
		18	5	1	20.00	1	20.00
		19	5	0	0.00	0	0.00
		**Sum**	**95**	**4**	**4.21**	**4**	**4.21**

Molecular docking

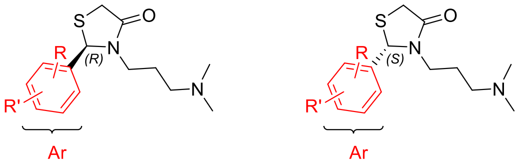

18: Ar = 4-Cl-*Ph*
20: Ar = 2-Cl-*Ph*
24: Ar = 4-F-*Ph*
26: Ar = 2-F-*Ph*
30: Ar = 2,4-Cl_2-*Ph*
31: Ar = 2,4-F_2-*Ph*
33: Ar = 2,3-F_2-*Ph*
34: Ar = 2,3-Cl_2-*Ph*

Figure S5. Structure of selected compounds for docking studies.

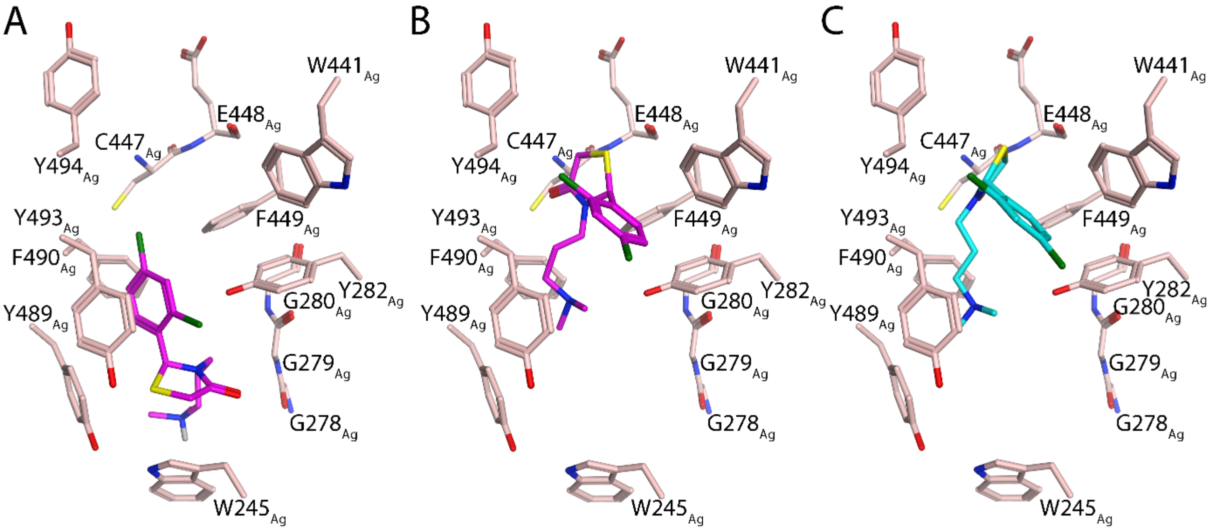

Figure S6. Docking poses of the 4-Thiazolidinones binding to *Ag*AChE1s (waters excluded): (a) pose A and **30S**, (b) pose B of **30S** and (c) pose B of **30R**.

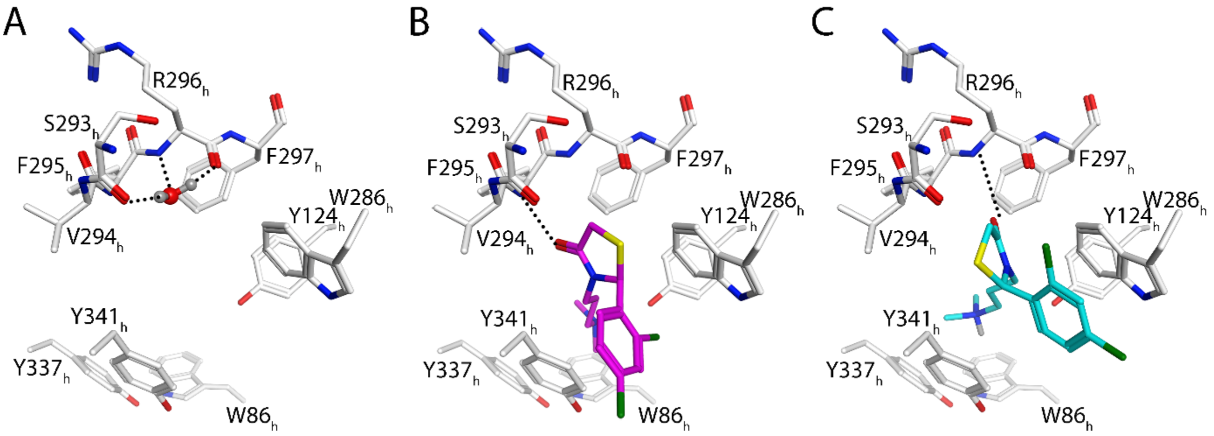

Figure S7. Docking poses of selected 4-thiazolidinones binding to *h*AChE were restricted by a water molecule (A) and only when this water molecule was removed were pose B of **30S** and **30R** observed (B and C).

Table S23. Crystal structures of G122S-*Ag*AChE1 included for water analysis.

No	PDB code	Resolution
1	6ARY	2.257 Å
2	6ARX	2.302 Å

Table S24. Crystal structures of *h*AChE included for water analysis.

No	PDB code	Resolution
1	4M0E	2 Å
2	6NTO	2.052 Å
3	6O69	2.081 Å
4	5HF5	2.152 Å
5	6O5V	2.152 Å
6	4EY4	2.156 Å
7	6WVO	2.19 Å
8	5HF9	2.2 Å
9	5HFA	2.201 Å
10	6CQZ	2.216 Å
11	6NTL	2.25 Å
12	6U37	2.25 Å
13	6WUZ	2.253 Å
14	6CQT	2.273 Å
15	6CQW	2.278 Å
16	6WVQ	2.289 Å
17	5HF6	2.3 Å
18	6O4X	2.3 Å
19	4EY5	2.3012 Å
20	4M0F	2.304 Å

Table S25. Crystal structures of *m*AChE included for water analysis.

No	PDB code	Resolution
1	5DTI	2.003 Å
2	2HA2	2.05 Å
3	4B82	2.1 Å
4	4B85	2.1 Å
5	5EIE	2.1 Å
6	2HA5	2.15 Å
7	2WHQ	2.15 Å
8	1N5M	2.2 Å
9	2GYU	2.2 Å
10	2HA0	2.2 Å
11	2WHP	2.2 Å
12	1N5R	2.25 Å

13	2HA3	2.25 Å
14	2HA6	2.25 Å
15	4ARB	2.25 Å
16	4B7Z	2.3 Å
17	5FOQ	2.3 Å
18	7R02	2.3 Å
19	7R2F	2.3 Å

Table S26. Waters considered in molecular docking based on analysis of crystal structures in Table S26.

**Waters (PDB: 6ARY)**	**Frequence**
HOH909	2/2
HOH986	2/2

Table S27. Waters considered in molecular docking based on analysis of crystal structures in Tables S27 and S28.

***h*AChE**		***m*ACHE**	
**Waters (PDB: 6O5V)**	**Frequence**	**Waters (PDB: 4B82)**	**Frequence**
HOH864	20/20	HOH2189	19/19
HOH746	19/20	HOH2190	19/19
HOH1014	19/20	HOH2139	19/19
HOH803	19/20	HOH2199	18/19
HOH1018	20/20	HOH2144	18/19
HOH876	20/20	HOH2127	18/19
HOH738	14/20	HOH2122	12/19
-[a]	14/20	HOH2117	13/19
HOH934	17/20	HOH2356	14/19
HOH822	20/20	HOH2194	18/19
HOH710	20/20	HOH2340	16/19

[a]Not present in the structure 6O5V

Inhibition kinetics

Table S28. Michaelis-Menten kinetics for the *Ag*AChE1 and the substrate acetylthiocholine iodide at different concentrations of the inhibitor **20**.

Conc. Inhibitor[a]	V_{max}[b] (abs/min)		K_m (µM)		R^2
0 µM	**202**	(171-240)[c]	**43**	(27-68)	**0.86**
0.001 µM	**175**	(147-208)	**34**	(20-55)	**0.83**
0.01 µM	**187**	(158-224)	**67**	(43-104)	**0.90**
0.1 µM	**230**	(200-265)	**68**	(48-96)	**0.92**
1 µM	**145**	(113-188)	**43**	(22-83)	**0.75**
3 µM	**119**	(95-152)	**43**	(22-80)	**0.76**
10 µM	**94**	(64-158)	**105**	(42-289)	**0.72**
30 µM	**47**	(37-63)	**107**	(60-198)	**0.84**
100 µM	**19**	(14-29)	**187**	(101-408)	**0.85**

[a]Compounds tested as HCl salts. **[b]**Apparent max velocity (abs/min) at the experimental conditions. [c]95% confidence interval given in parenthesis.

Table S29. Michaelis-Menten kinetics for the *Ag*AChE1 and the substrate acetylthiocholine iodide at different concentrations of the inhibitor **24**.

Conc. Inhibitor[a]	V_{max}[b] (abs/min)		K_m (µM)		R^2
0 µM	**256**	(218-305)[c]	**98**	(69-139)	**0,95**
0.001 µM	**173**	(146-207)	**54**	(35-83)	**0,92**
0.01 µM	**193**	(157-243)	**84**	(50-146)	**0,89**
0.1 µM	**134**	(119-153)	**58**	(42-79)	**0,95**
0.3 µM	**110**	(91-134)	**60**	(38-94)	**0,91**
1 µM	**120**	(100-147)	**94**	(61-154)	**0,94**
3 µM	**30**	(24-37)	**42**	(25-69)	**0,83**
10 µM	**33**	(22-86)	**233**	(97-993)	**0,90**
100 µM	**-**[d]		**-**		**-**

[a]Compounds tested as HCl salts. **[b]**Apparent max velocity (abs/min) at the experimental conditions. [c]95% confidence interval given in parenthesis. [d]The enzyme activity was too low for modelling.

Table S30. Michaelis-Menten kinetics for the *Ag*AChE1 and the substrate acetylthiocholine iodide at different concentrations of the inhibitor **30**.

Conc. Inhibitor[a]	V_{max}[b] (abs/min)		K_m (µM)		R^2
0 µM	**200**	(183-219)[c]	**55**	(42-71)	**0.97**
0.001 µM	**252**	(227-281)	**68**	(51-89)	**0.96**
0.01 µM	**251**	(230-274)	**72**	(58-90)	**0.98**
0.1 µM	**248**	(220-282)	**99**	(75-130)	**0.97**
0.3 µM	**163**	(143-187)	**58**	(40-83)	**0.93**

1 µM	**135**	(110-168)	**72**	(43-124)	**0.90**
3 µM	**56**	(40-84)	**52**	(19-143)	**0.63**
10 µM	**20**	(15-27)	**73**	(37-149)	**0.82**

[a]Compounds tested as HCl salts. [b]Apparent max velocity (abs/min) at the experimental conditions. [c]95% confidence interval given in parenthesis.

Table S31. Michaelis-Menten kinetics for the G122S-*Ag*AChE1 and the substrate acetylthiocholine iodide at different concentrations of the inhibitor **20**.

**Conc. Inhibitor[a]**	**V_{max}[b] (abs/min)**		**K_m (µM)**		**R^2**
0 µM	**104**	(83-133)[c]	**47**	(24-87)	**0,84**
0.1 µM	**93**	(76-115)	**75**	(45-123)	**0,90**
1 µM	**83**	(71-96)	**45**	(29-68)	**0,93**
10 µM	**71**	(60-84)	**43**	(26-69)	**0,90**
30 µM	**53**	(42-68)	**66**	(36-114)	**0,81**
100 µM	**37**	(31-45)	**42**	(21-75)	**0,84**
300 µM	**24**	(20-30)	**58**	(32-103)	**0,84**
500 µM	**-[d]**		**-**		**-**

[a]Compounds tested as HCl salts. [b]Apparent max velocity (abs/min) at the experimental conditions. [c]95% confidence interval given in parenthesis. [d]The enzyme activity was too low for modelling.

Table S32. Michaelis-Menten kinetics for the G122S-*Ag*AChE1 and the substrate acetylthiocholine iodide at different concentrations of the inhibitor **30**.

**Conc. Inhibitor[a]**	**V_{max}[b] (abs/min)**		**K_m (µM)**		**R^2**
0 µM	**244**	(214-280)[c]	**45**	(30-67)	**0.93**
0.1 µM	**189**	(152-237)	**41**	(21-77)	**0.89**
3 µM	**184**	(139-247)	**56**	(25-112)	**0.78**
10 µM	**140**	(113-176)	**40**	(19-77)	**0.81**
30 µM	**80**	(60-111)	**64**	(30-134)	**0.80**
100 µM	**49**	(33-76)	**50**	(11-145)	**0.62**
300 µM	**21**	(16-30)	**23**	(5-75)	**0.70**
500 µM	**-[d]**		**-**		**-**

[a]Compounds tested as HCl salts. [b]Apparent max velocity at the experimental conditions. [c]95% confidence interval given in parenthesis. [d]The enzyme activity was too low for modelling.

Table S33. Michaelis-Menten kinetics for the *h*AChE and the substrate acetylthiocholine iodide at different concentrations of the inhibitor **20**.

Conc. Inhibitor[a]	V_{max}[b] (abs/min)		K_m (µM)		R^2
0 µM	**569**	(468-719)[c]	**235**	(161-356)	**0,88**
0.1 µM	**469**	(370-625)	**178**	(107-306)	**0,84**
1 µM	**576**	(459-757)	**210**	(133-340)	**0,86**
10 µM	**562**	(440-762)	**268**	(171-440)	**0,87**
30 µM	**515**	(401-699)	**242**	(152-402)	**0,84**
100 µM	**553**	(385-936)	**407**	(226-854)	**0,82**
300 µM	**372**	(251-694)	**463**	(246-1084)	**0,83**
500 µM	**324**	(212-754)	**649**	(337-1913)	**0,89**
1000 µM	**74**	(54-107)	**76**	(27-191)	**0,46**

[a]Compounds tested as HCl salts. [b]Apparent max velocity (abs/min) at the experimental conditions. [c]95% confidence interval given in parenthesis.

Table S34. Michaelis-Menten kinetics for the *h*AChE and the substrate acetylthiocholine iodide at different concentrations of the inhibitor **30**.

Conc. Inhibitor[a]	V_{max}[b] (abs/min)		K_m (µM)		R^2
0 µM	**452**	(389-536)[c]	**200**	(148-275)	**0.95**
0.1 µM	**543**	(418-760)	**278**	(171-482)	**0.89**
1 µM	**484**	(429-554)	**193**	(151-249)	**0.96**
5 µM	**473**	(403-569)	**196**	(141-276)	**0.93**
10 µM	**511**	(428-627)	**218**	(154-314)	**0.93**
50 µM	**229**	(194-278)	**140**	(94-208)	**0.90**
100 µM	**145**	(114-196)	**151**	(84-272)	**0.81**
200 µM	**101**	(80-133)	**158**	(93-273)	**0.84**
500 µM	**95**	(69-151)	**365**	(209-728)	**0.88**

[a]Compounds tested as HCl salts. [a]Apparent max velocity at the experimental conditions. [b]95% confidence interval given in parenthesis.

Table S35. Non-competitive enzyme inhibition curve fitting (non-linear regression) of experiments in Tables S29-S34 to determine kinetic inhibition constants

Enzyme	ID[a]	Ki (μM)		V_{max} [b] (abs/min)		K_m (μM)		R^2
*Ag*AChE1	**20**	**5.4**	(4.2-6.8)	**193**	(182-207)[c]	**49**	(41-58)	**0.87**
	**24**	**0.73**	(0.57-0.95)	**191**	(177-207)	**73**	(61-87)	**0.91**
	**30**	**1.0**	(0.9-1.2)	**235**	(224-247)	**68**	(60-76)	**0.93**
G122S-*Ag*AChE1	**20**	**54**	(42-70)	**90**[d]	(82-98)	**51**	(41-64)	**0,86**
	**24**	n.d.[e]		n.d.		n.d.		n.d.
	**30**	**19**	(14-24)	**217**[d]	(197-239)	**49**	(37-62)	**0.95**
*h*AChE	**20**	**231**	(199-267)	**580**	(532-636)	**243**	(208-286)	**0.89**
	**24**	n.d.		n.d.		n.d.		n.d.
	**30**	**65**	(58-74)	**498**	(465-535)	**207**	(181-236)	**0.88**

[a]Compounds tested as HCl salts. [b]Apparent max velocity at the experimental conditions. [c]95% confidence interval given in parenthesis. [d]Different enzyme concentrations due to limited material. [e]n.d.= not determined.

NMR spectra

4

AL239 full charac MeOD.1.fid

Parameter	Value
Data File Name	C:/ Users/ anvi0182/ Desktop/ NMR/ AL239 full charac MeOD/ 1/ fid
Title	AL239 full charac MeOD.1.fid
Comment	
Origin	Bruker BioSpin GmbH
Owner	nmr
Site	
Instrument	spect
Author	
Solvent	MeOD
Temperature	298.0
Pulse Sequence	zg30
Experiment	1D
Probe	Z116098_0046 (PA BBO 400S1 BBF-H-D-05 Z SP)
Number of Scans	16
Receiver Gain	69.1
Relaxation Delay	1.0000
Pulse Width	9.7800
Presaturation Frequency	
Acquisition Time	4.1069
Acquisition Date	2020-07-17T17:49:25
Modification Date	2020-07-17T17:49:25
Class	
Spectrometer Frequency	399.60
Spectral Width	7978.7
Lowest Frequency	-1529.3
Nucleus	1H
Acquired Size	32768
Spectral Size	65536

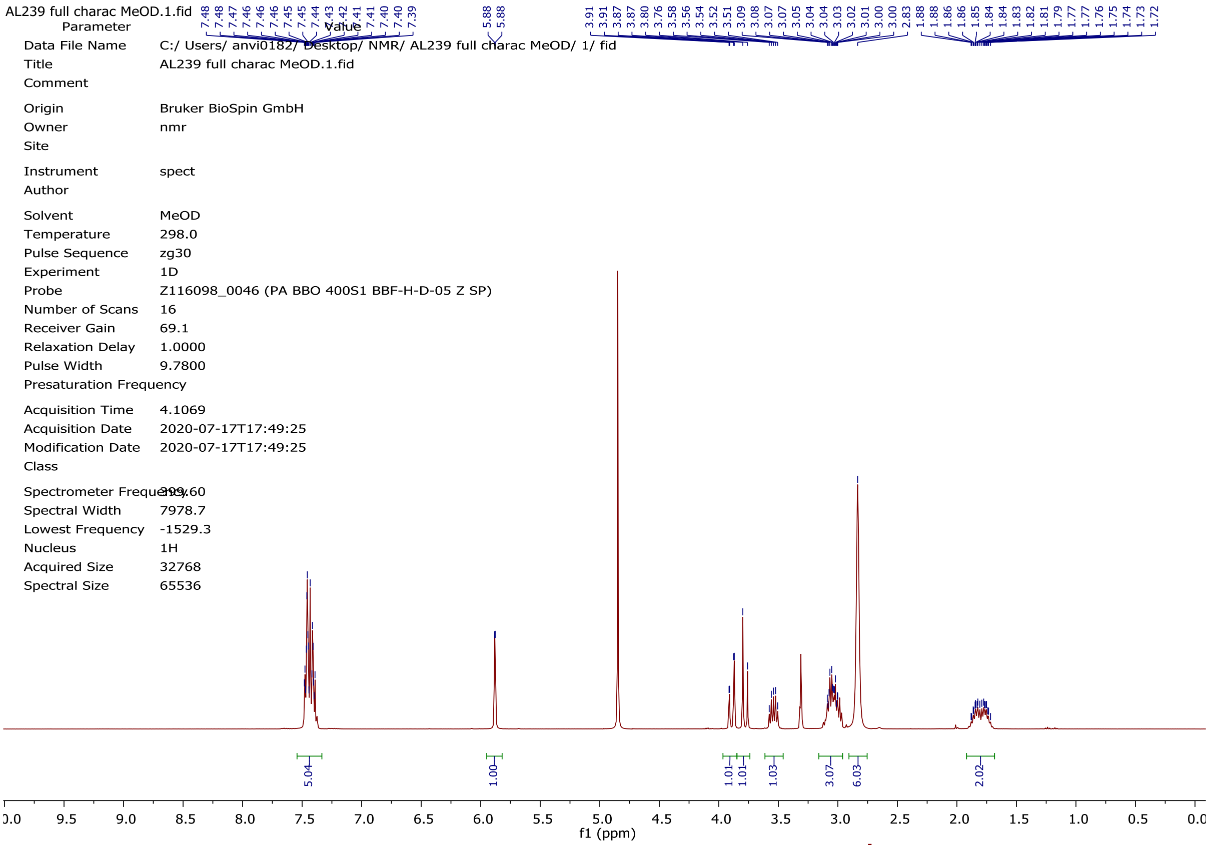

Parameter	Value
Data File Name	C:/ Users/ anvi0182/ Desktop/ NMR/ AL239 full charac MeOD/ 3/ fid
Title	AL239 full charac MeOD.3.fid
Comment	
Origin	Bruker BioSpin GmbH
Owner	nmr
Site	
Instrument	spect
Author	
Solvent	MeOD
Temperature	298.0
Pulse Sequence	zgpg30
Experiment	1D
Probe	Z116098_0046 (PA BBO 400S1 BBF-H-D-05 Z SP)
Number of Scans	1024
Receiver Gain	191.2
Relaxation Delay	2.0000
Pulse Width	10.0000
Presaturation Frequency	
Acquisition Time	1.3631
Acquisition Date	2020-07-17T23:17:19
Modification Date	2020-07-17T23:17:19
Class	
Spectrometer Frequency	100.49
Spectral Width	24038.5
Lowest Frequency	-1834.4
Nucleus	13C
Acquired Size	32768
Spectral Size	65536

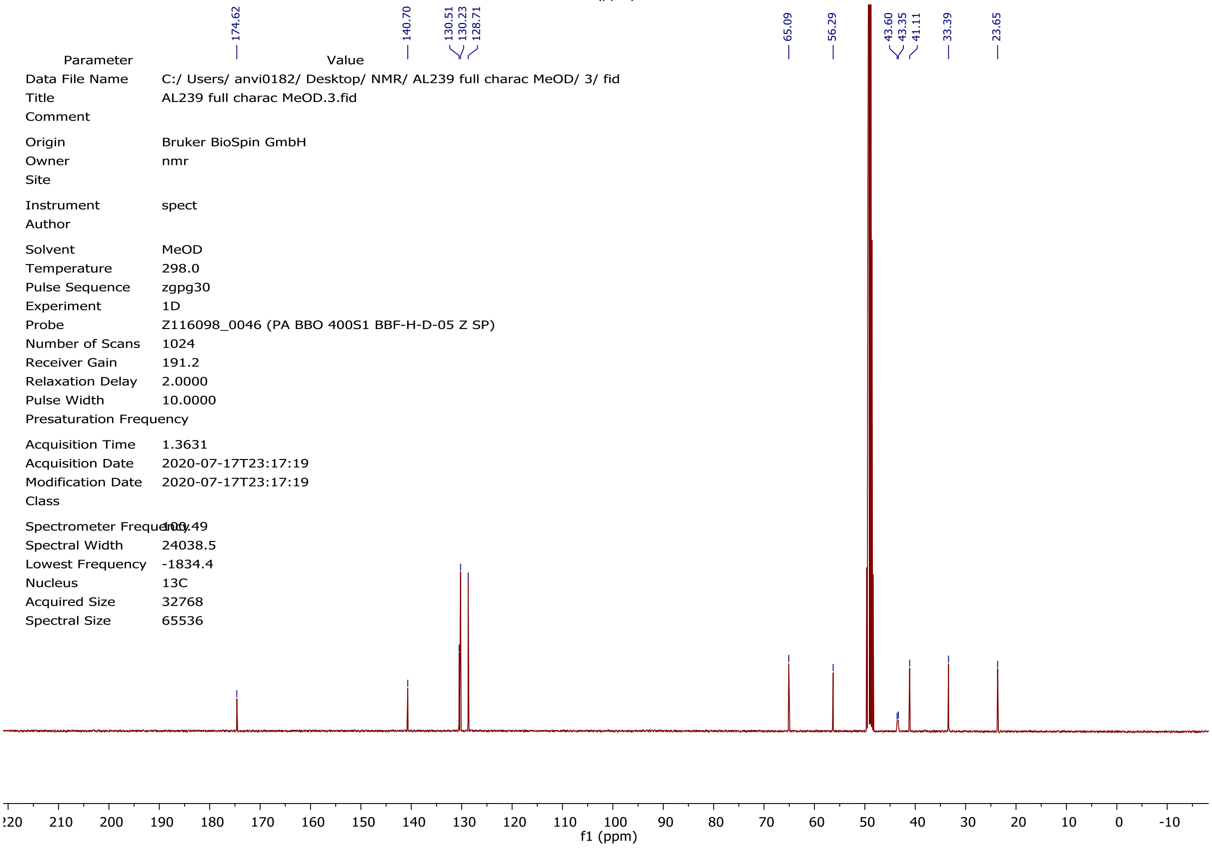

5

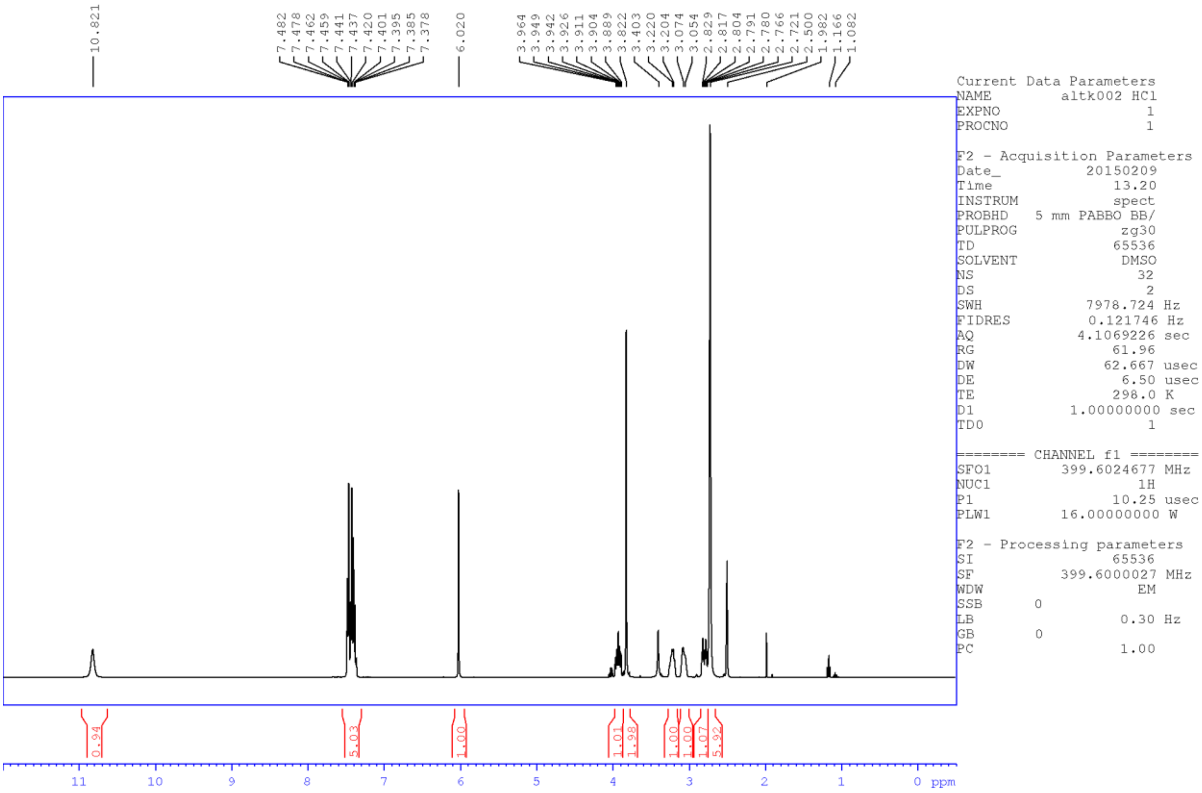

10.821
7.482 7.478 7.462 7.459 7.441 7.437 7.420 7.401 7.395 7.385 7.378
6.020
3.964 3.949 3.942 3.926 3.911 3.904 3.889 3.822 3.403 3.220 3.204 3.074 3.054 2.829 2.817 2.804 2.791 2.780 2.766 2.721 2.500 1.982 1.166 1.082
0.94 5.03 1.00 1.01 1.98 1.00 1.00 1.07 5.92
11 10 9 8 7 6 5 4 3 2 1 0 ppm
Current Data Parameters
NAME altk002 HCl
EXPNO 1
PROCNO 1
F2 - Acquisition Parameters
Date_ 20150209
Time 13.20
INSTRUM spect
PROBHD 5 mm PABBO BB/
PULPROG zg30
TD 65536
SOLVENT DMSO
NS 32
DS 2
SWH 7978.724 Hz
FIDRES 0.121746 Hz
AQ 4.1069226 sec
RG 61.96
DW 62.667 usec
DE 6.50 usec
TE 298.0 K
D1 1.00000000 sec
TD0 1
======== CHANNEL f1 ========
SFO1 399.6024677 MHz
NUC1 1H
P1 10.25 usec
PLW1 16.00000000 W
F2 - Processing parameters
SI 65536
SF 399.6000027 MHz
WDW EM
SSB 0
LB 0.30 Hz
GB 0
PC 1.00

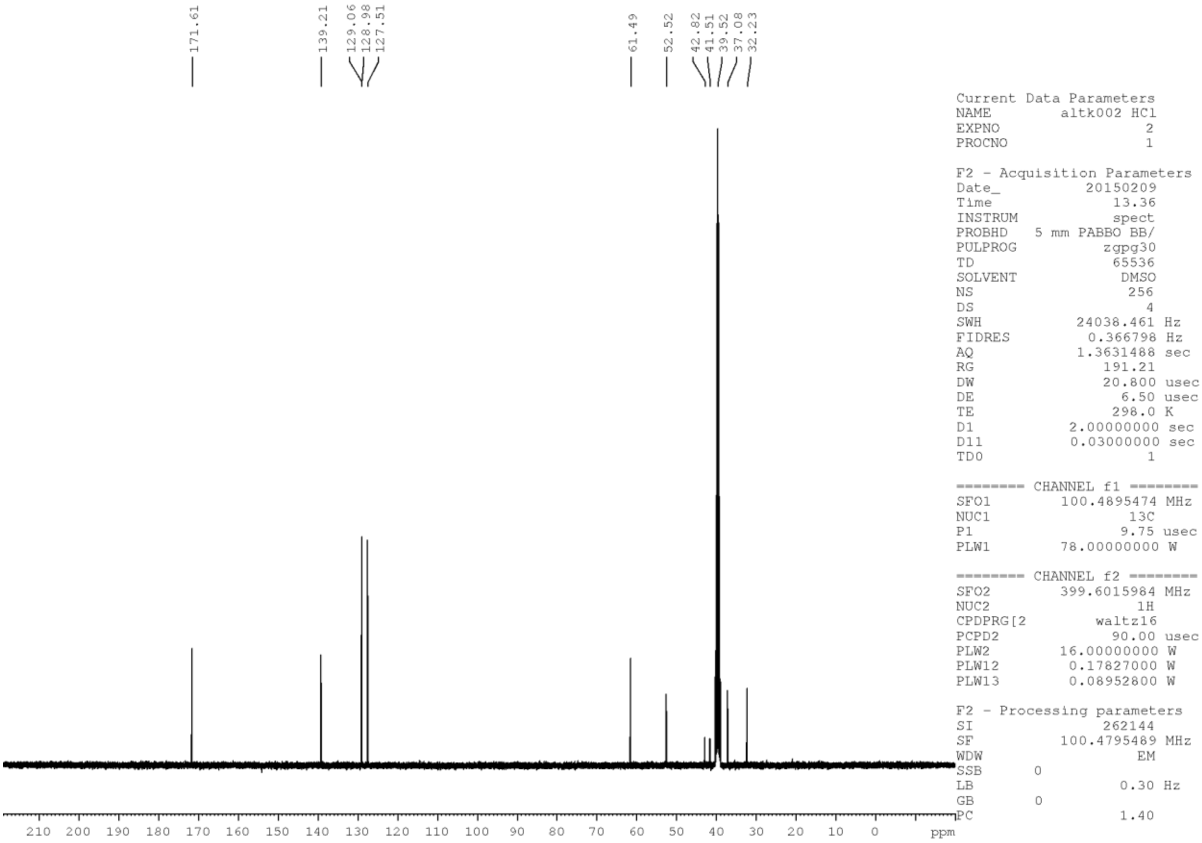

171.61
139.21
129.06 128.98 127.51
61.49
52.52
42.82 41.51 39.52 37.08 32.23
210 200 190 180 170 160 150 140 130 120 110 100 90 80 70 60 50 40 30 20 10 0 ppm
Current Data Parameters
NAME altk002 HCl
EXPNO 2
PROCNO 1
F2 - Acquisition Parameters
Date_ 20150209
Time 13.36
INSTRUM spect
PROBHD 5 mm PABBO BB/
PULPROG zgpg30
TD 65536
SOLVENT DMSO
NS 256
DS 4
SWH 24038.461 Hz
FIDRES 0.366798 Hz
AQ 1.3631488 sec
RG 191.21
DW 20.800 usec
DE 6.50 usec
TE 298.0 K
D1 2.00000000 sec
D11 0.03000000 sec
TD0 1
======== CHANNEL f1 ========
SFO1 100.4895474 MHz
NUC1 13C
P1 9.75 usec
PLW1 78.00000000 W
======== CHANNEL f2 ========
SFO2 399.6015984 MHz
NUC2 1H
CPDPRG[2 waltz16
PCPD2 90.00 usec
PLW2 16.00000000 W
PLW12 0.17827000 W
PLW13 0.08952800 W
F2 - Processing parameters
SI 262144
SF 100.4795489 MHz
WDW EM
SSB 0
LB 0.30 Hz
GB 0
PC 1.40

6

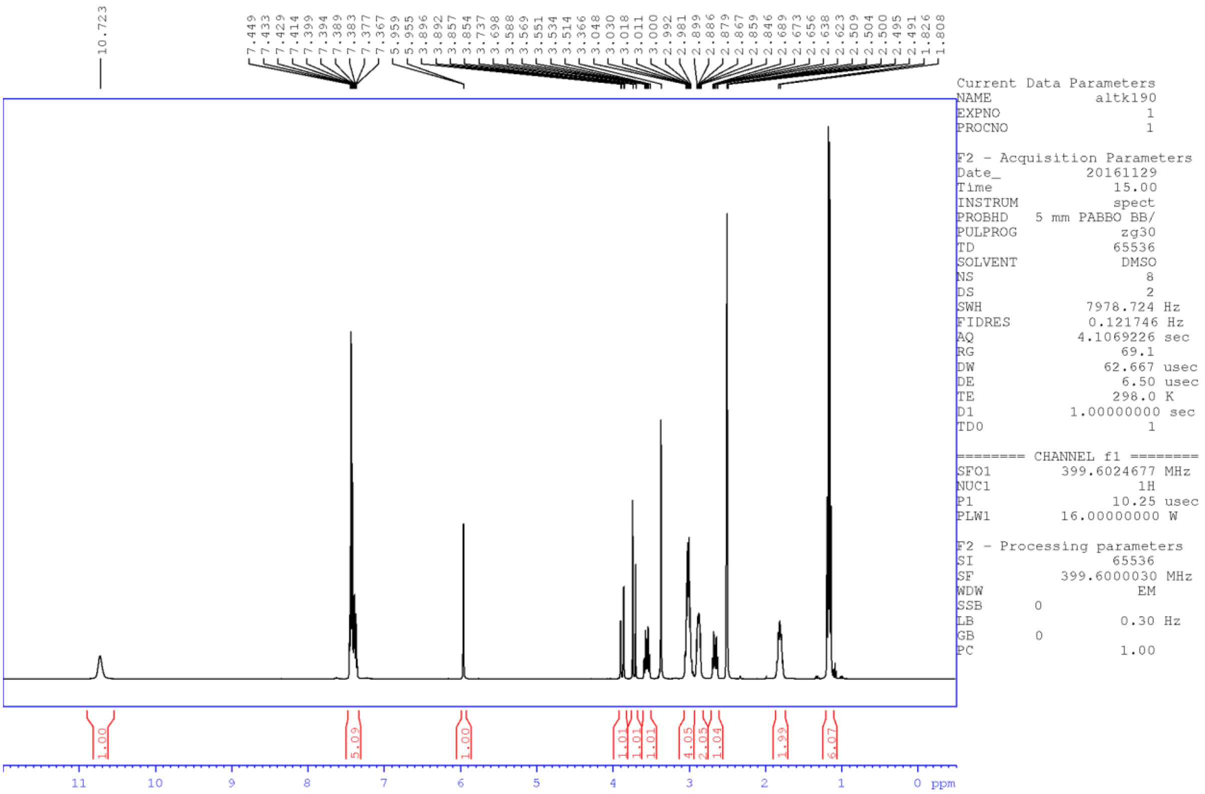
10.723
7.449 7.433 7.429 7.414 7.399 7.394 7.389 7.383 7.377 7.367 5.959 5.955 3.896 3.892 3.857 3.854 3.737 3.698 3.588 3.569 3.551 3.534 3.514 3.366 3.048 3.030 3.018 3.011 3.000 2.992 2.981 2.899 2.886 2.879 2.867 2.859 2.846 2.689 2.673 2.656 2.638 2.623 2.509 2.504 2.500 2.495 2.491 1.826 1.808
Current Data Parameters
NAME altk190
EXPNO 1
PROCNO 1
F2 - Acquisition Parameters
Date_ 20161129
Time 15.00
INSTRUM spect
PROBHD 5 mm PABBO BB/
PULPROG zg30
TD 65536
SOLVENT DMSO
NS 8
DS 2
SWH 7978.724 Hz
FIDRES 0.121746 Hz
AQ 4.1069226 sec
RG 69.1
DW 62.667 usec
DE 6.50 usec
TE 298.0 K
D1 1.00000000 sec
TD0 1
======== CHANNEL f1 ========
SFO1 399.6024677 MHz
NUC1 1H
P1 10.25 usec
PLW1 16.00000000 W
F2 - Processing parameters
SI 65536
SF 399.6000030 MHz
WDW EM
SSB 0
LB 0.30 Hz
GB 0
PC 1.00
1.00 5.09 1.00 1.01 1.01 1.01 1.05 2.05 1.04 1.99 6.07
11 10 9 8 7 6 5 4 3 2 1 0 ppm

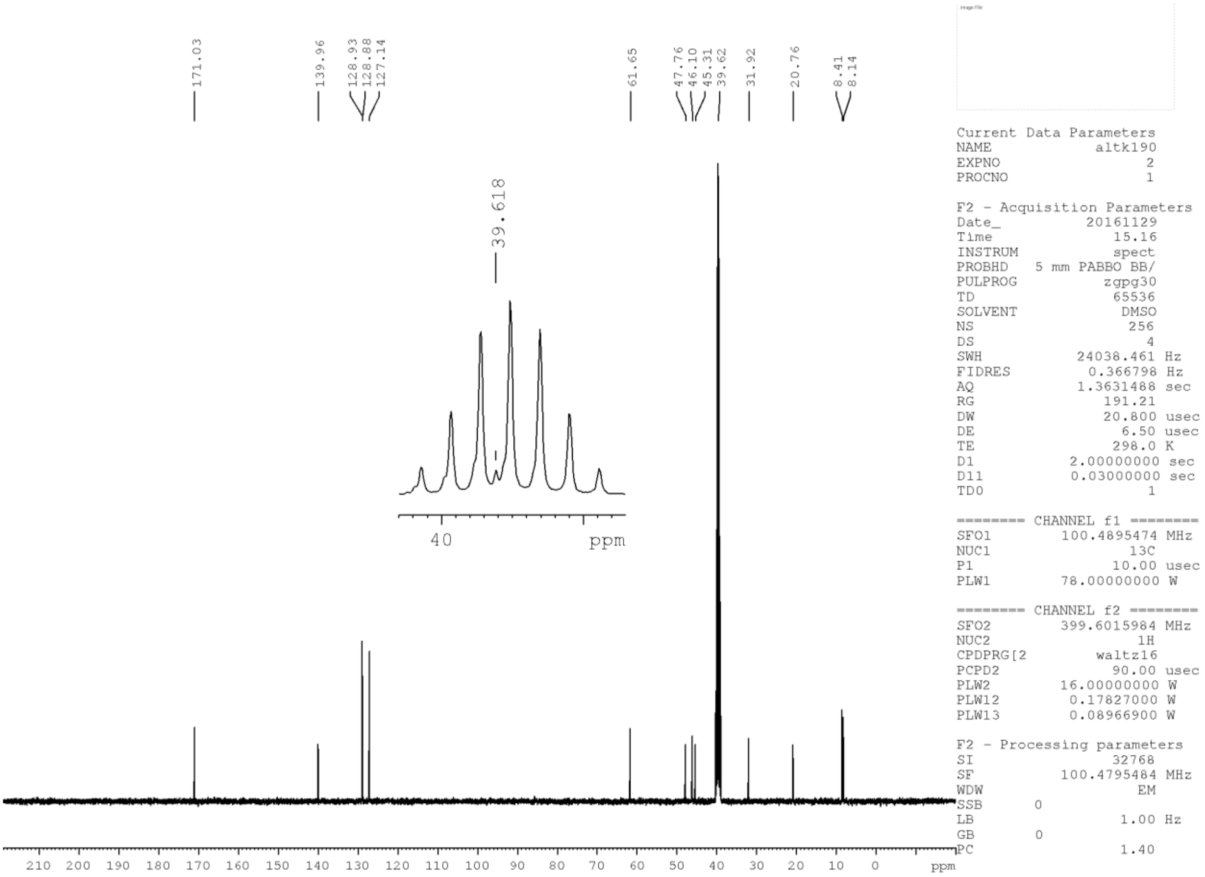
171.03
139.96 128.93 128.88 127.14
61.65
47.76 46.10 45.31 39.62
31.92
20.76
8.41 8.14
39.618
40 ppm
Current Data Parameters
NAME altk190
EXPNO 2
PROCNO 1
F2 - Acquisition Parameters
Date_ 20161129
Time 15.16
INSTRUM spect
PROBHD 5 mm PABBO BB/
PULPROG zgpg30
TD 65536
SOLVENT DMSO
NS 256
DS 4
SWH 24038.461 Hz
FIDRES 0.366798 Hz
AQ 1.3631488 sec
RG 191.21
DW 20.800 usec
DE 6.50 usec
TE 298.0 K
D1 2.00000000 sec
D11 0.03000000 sec
TD0 1
======== CHANNEL f1 ========
SFO1 100.4895474 MHz
NUC1 13C
P1 10.00 usec
PLW1 78.00000000 W
======== CHANNEL f2 ========
SFO2 399.6015984 MHz
NUC2 1H
CPDPRG[2 waltz16
PCPD2 90.00 usec
PLW2 16.00000000 W
PLW12 0.17827000 W
PLW13 0.08966900 W
F2 - Processing parameters
SI 32768
SF 100.4795484 MHz
WDW EM
SSB 0
LB 1.00 Hz
GB 0
PC 1.40
210 200 190 180 170 160 150 140 130 120 110 100 90 80 70 60 50 40 30 20 10 0 ppm

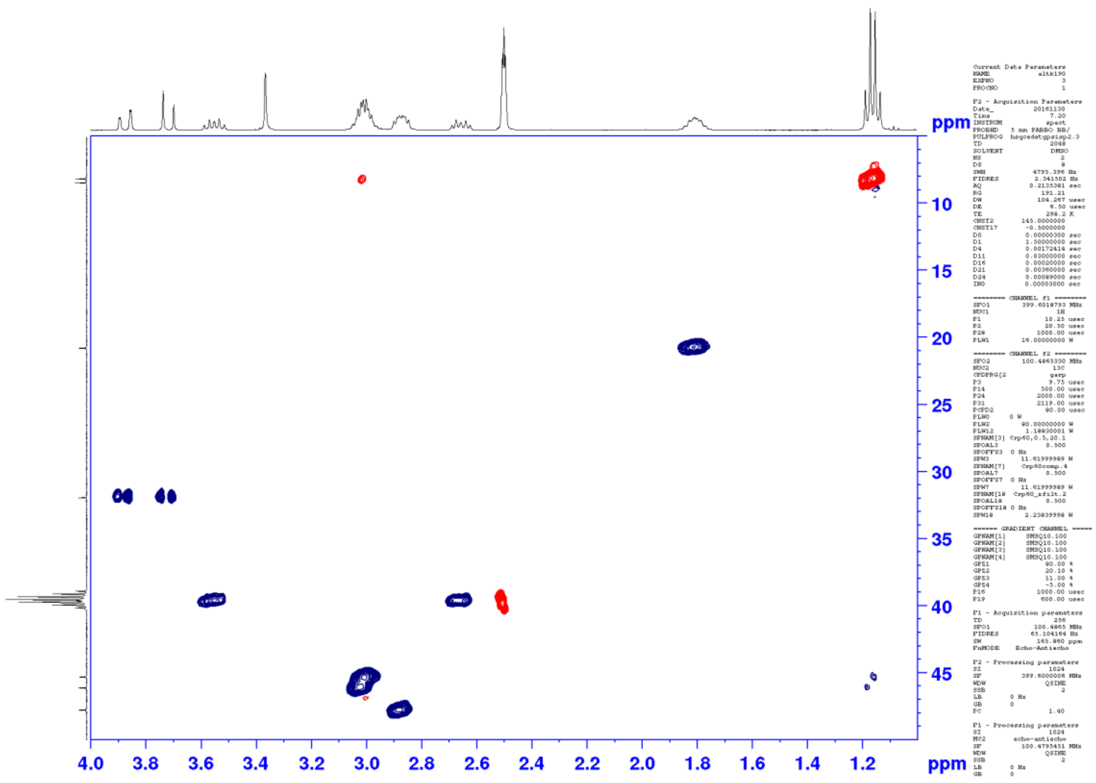

ppm
10
15
20
25
30
35
40
45
4.0 3.8 3.6 3.4 3.2 3.0 2.8 2.6 2.4 2.2 2.0 1.8 1.6 1.4 1.2
ppm
Current Data Parameters
NAME altk190
EXPNO 3
PROCNO 1
F2 - Acquisition Parameters
Date_ 20161130
Time 7.20
INSTRUM spect
PROBHD 5 mm PABBO BB/
PULPROG hsqcedetgpsisp2.3
TD 2048
SOLVENT DMSO
NS 2
DS 8
SWH 4795.396 Hz
FIDRES 2.341502 Hz
AQ 0.2135381 sec
RG 191.21
DW 104.267 usec
DE 6.50 usec
TE 298.2 K
CNST2 145.0000000
CNST17 -0.5000000
D0 0.00000300 sec
D1 1.50000000 sec
D4 0.00172414 sec
D11 0.03000000 sec
D16 0.00020000 sec
D21 0.00360000 sec
D24 0.00089000 sec
IN0 0.00003000 sec
======== CHANNEL f1 ========
SFO1 399.6018793 MHz
NUC1 1H
P1 10.25 usec
P2 20.50 usec
P28 1000.00 usec
PLW1 16.00000000 W
======== CHANNEL f2 ========
SFO2 100.4865330 MHz
NUC2 13C
CPDPRG[2 garp
P3 9.75 usec
P14 500.00 usec
P24 2000.00 usec
P31 2119.00 usec
PCPD2 80.00 usec
PLW0 0 W
PLW2 80.00000000 W
PLW12 1.18830001 W
SPNAM[3] Crp60,0.5,20.1
SPOAL3 0.500
SPOFFS3 0 Hz
SPW3 11.61999989 W
SPNAM[7] Crp60comp.4
SPOAL7 0.500
SPOFFS7 0 Hz
SPW7 11.61999989 W
SPNAM[18 Crp60_xfilt.2
SPOAL18 0.500
SPOFFS18 0 Hz
SPW18 2.23839998 W
====== GRADIENT CHANNEL =====
GPNAM[1] SMSQ10.100
GPNAM[2] SMSQ10.100
GPNAM[3] SMSQ10.100
GPNAM[4] SMSQ10.100
GPZ1 80.00 %
GPZ2 20.10 %
GPZ3 11.00 %
GPZ4 -5.00 %
P16 1000.00 usec
P19 600.00 usec
F1 - Acquisition parameters
TD 256
SFO1 100.4865 MHz
FIDRES 65.104164 Hz
SW 165.860 ppm
FnMODE Echo-Antiecho
F2 - Processing parameters
SI 1024
SF 399.6000006 MHz
WDW QSINE
SSB 2
LB 0 Hz
GB 0
PC 1.40
F1 - Processing parameters
SI 1024
MC2 echo-antiecho
SF 100.4795451 MHz
WDW QSINE
SSB 2
LB 0 Hz
GB 0

7

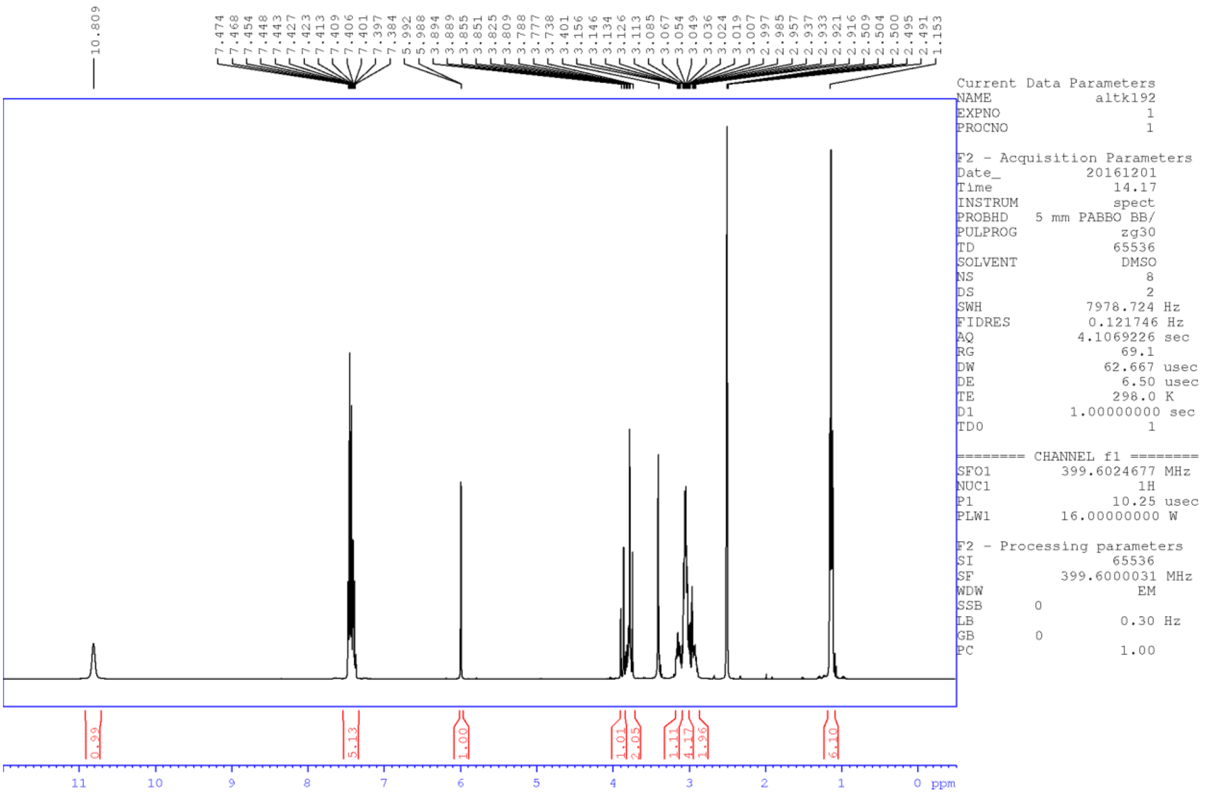
10.809
7.474 7.468 7.454 7.448 7.443 7.427 7.423 7.413 7.409 7.406 7.401 7.397 7.384
5.992 5.988
3.894 3.889 3.855 3.851 3.825 3.809 3.788 3.777 3.738 3.401 3.156 3.146 3.134 3.126 3.113 3.085 3.067 3.054 3.049 3.036 3.024 3.019 3.007 2.997 2.985 2.957 2.937 2.933 2.921 2.916 2.509 2.504 2.500 2.495 2.491 1.153
0.99 5.13 1.00 1.01 2.05 1.11 1.17 1.96 6.10
11 10 9 8 7 6 5 4 3 2 1 0 ppm
Current Data Parameters
NAME altk192
EXPNO 1
PROCNO 1
F2 - Acquisition Parameters
Date_ 20161201
Time 14.17
INSTRUM spect
PROBHD 5 mm PABBO BB/
PULPROG zg30
TD 65536
SOLVENT DMSO
NS 8
DS 2
SWH 7978.724 Hz
FIDRES 0.121746 Hz
AQ 4.1069226 sec
RG 69.1
DW 62.667 usec
DE 6.50 usec
TE 298.0 K
D1 1.00000000 sec
TD0 1
======== CHANNEL f1 ========
SFO1 399.6024677 MHz
NUC1 1H
P1 10.25 usec
PLW1 16.00000000 W
F2 - Processing parameters
SI 65536
SF 399.6000031 MHz
WDW EM
SSB 0
LB 0.30 Hz
GB 0
PC 1.00

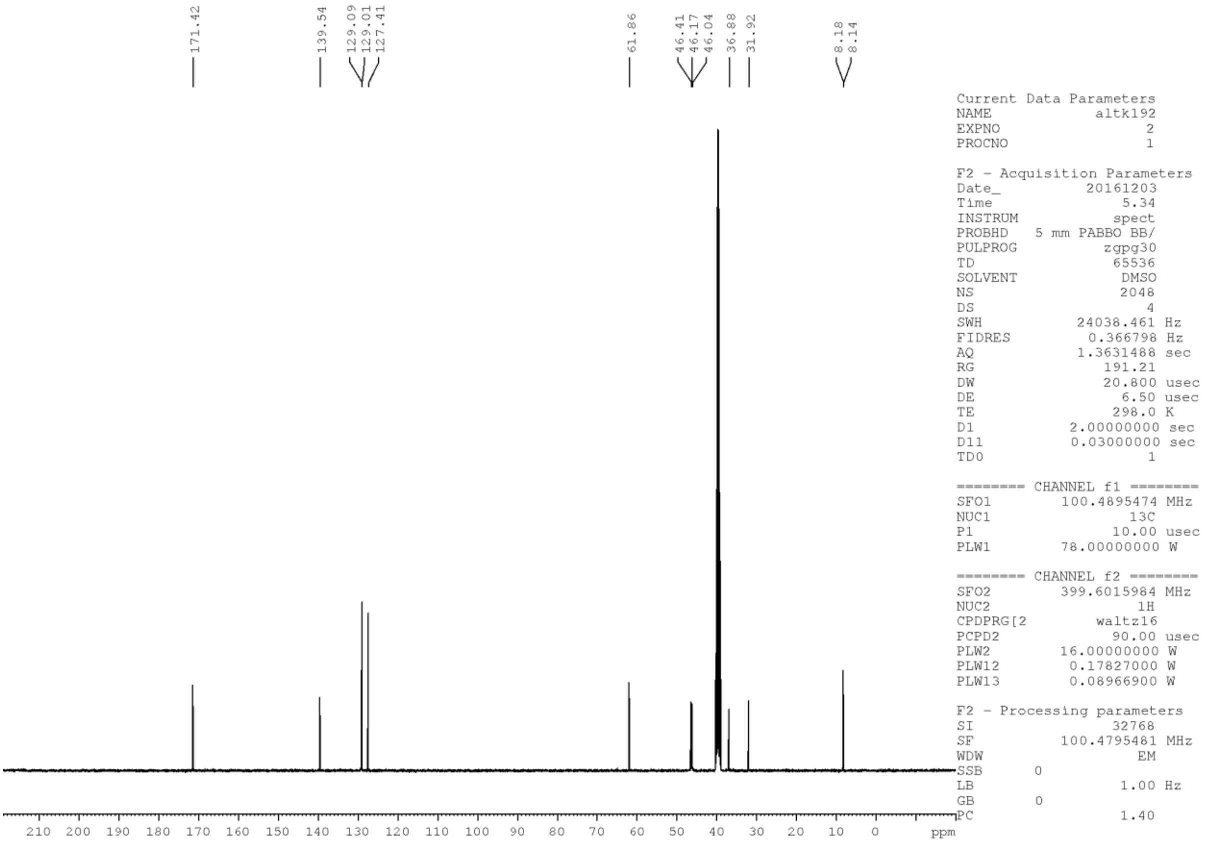
171.42
139.54 129.09 129.01 127.41
61.86
46.41 46.17 46.04 36.88 31.92
8.18 8.14
210 200 190 180 170 160 150 140 130 120 110 100 90 80 70 60 50 40 30 20 10 0 ppm
Current Data Parameters
NAME altk192
EXPNO 2
PROCNO 1
F2 - Acquisition Parameters
Date_ 20161203
Time 5.34
INSTRUM spect
PROBHD 5 mm PABBO BB/
PULPROG zgpg30
TD 65536
SOLVENT DMSO
NS 2048
DS 4
SWH 24038.461 Hz
FIDRES 0.366798 Hz
AQ 1.3631488 sec
RG 191.21
DW 20.800 usec
DE 6.50 usec
TE 298.0 K
D1 2.00000000 sec
D11 0.03000000 sec
TD0 1
======== CHANNEL f1 ========
SFO1 100.4895474 MHz
NUC1 13C
P1 10.00 usec
PLW1 78.00000000 W
======== CHANNEL f2 ========
SFO2 399.6015984 MHz
NUC2 1H
CPDPRG[2 waltz16
PCPD2 90.00 usec
PLW2 16.00000000 W
PLW12 0.17827000 W
PLW13 0.08966900 W
F2 - Processing parameters
SI 32768
SF 100.4795481 MHz
WDW EM
SSB 0
LB 1.00 Hz
GB 0
PC 1.40

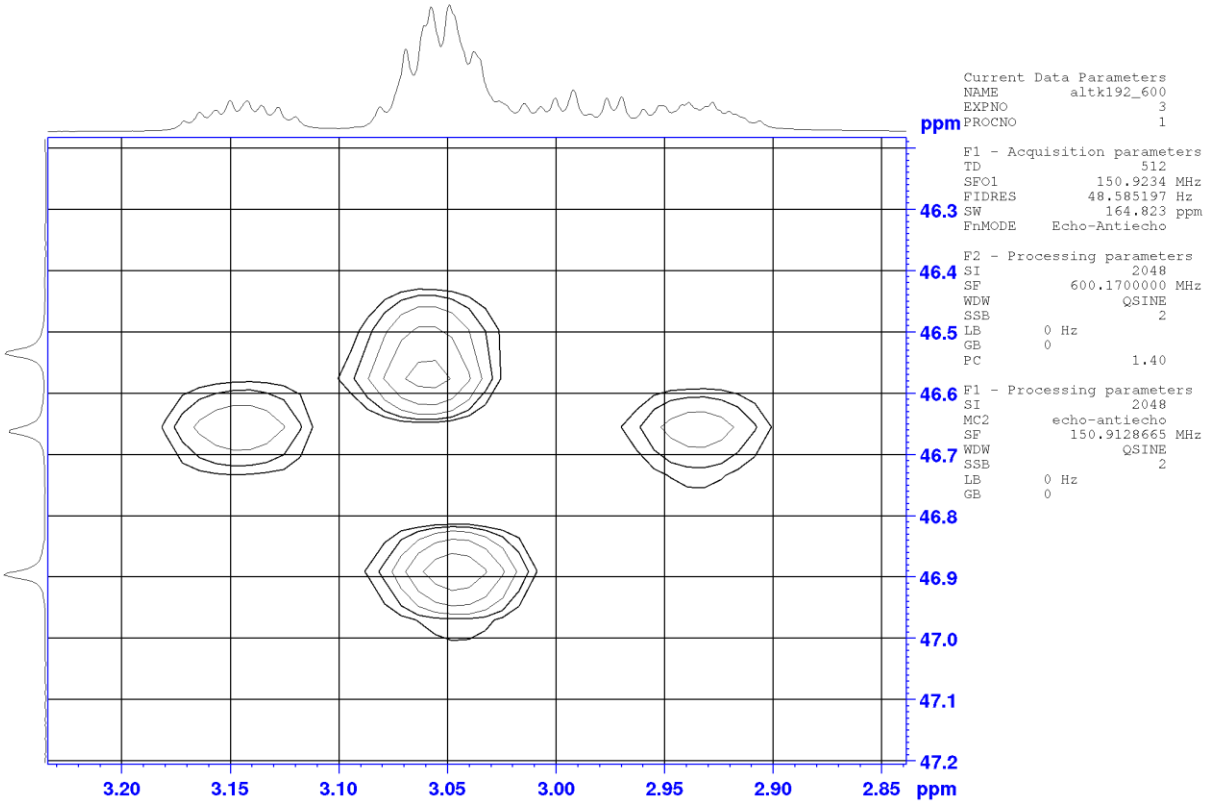

ppm
46.3
46.4
46.5
46.6
46.7
46.8
46.9
47.0
47.1
47.2
3.20
3.15
3.10
3.05
3.00
2.95
2.90
2.85
ppm
Current Data Parameters
NAME altk192_600
EXPNO 3
PROCNO 1
F1 - Acquisition parameters
TD 512
SFO1 150.9234 MHz
FIDRES 48.585197 Hz
SW 164.823 ppm
FnMODE Echo-Antiecho
F2 - Processing parameters
SI 2048
SF 600.1700000 MHz
WDW QSINE
SSB 2
LB 0 Hz
GB 0
PC 1.40
F1 - Processing parameters
SI 2048
MC2 echo-antiecho
SF 150.9128665 MHz
WDW QSINE
SSB 2
LB 0 Hz
GB 0

8

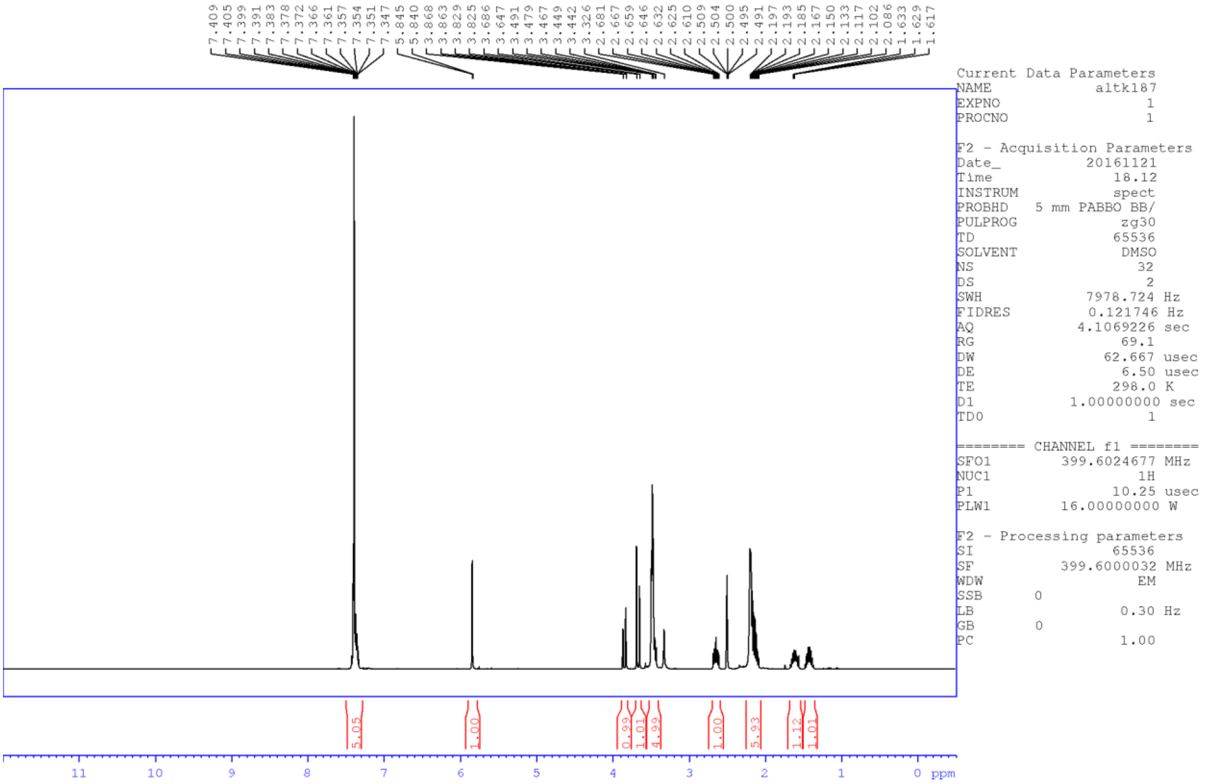
7.409 7.405 7.399 7.391 7.383 7.378 7.372 7.366 7.361 7.357 7.354 7.351 7.347 5.845 5.840 3.868 3.863 3.829 3.825 3.686 3.647 3.491 3.479 3.467 3.449 3.442 3.326 2.681 2.667 2.659 2.646 2.632 2.625 2.610 2.509 2.504 2.500 2.495 2.491 2.197 2.193 2.185 2.167 2.150 2.133 2.117 2.102 2.086 1.633 1.629 1.617
5.05 1.00 0.99 1.01 4.99 1.00 5.93 1.12 1.01
11 10 9 8 7 6 5 4 3 2 1 0 ppm
Current Data Parameters
NAME altk187
EXPNO 1
PROCNO 1
F2 - Acquisition Parameters
Date_ 20161121
Time 18.12
INSTRUM spect
PROBHD 5 mm PABBO BB/
PULPROG zg30
TD 65536
SOLVENT DMSO
NS 32
DS 2
SWH 7978.724 Hz
FIDRES 0.121746 Hz
AQ 4.1069226 sec
RG 69.1
DW 62.667 usec
DE 6.50 usec
TE 298.0 K
D1 1.00000000 sec
TD0 1
======== CHANNEL f1 ========
SFO1 399.6024677 MHz
NUC1 1H
P1 10.25 usec
PLW1 16.00000000 W
F2 - Processing parameters
SI 65536
SF 399.6000032 MHz
WDW EM
SSB 0
LB 0.30 Hz
GB 0
PC 1.00

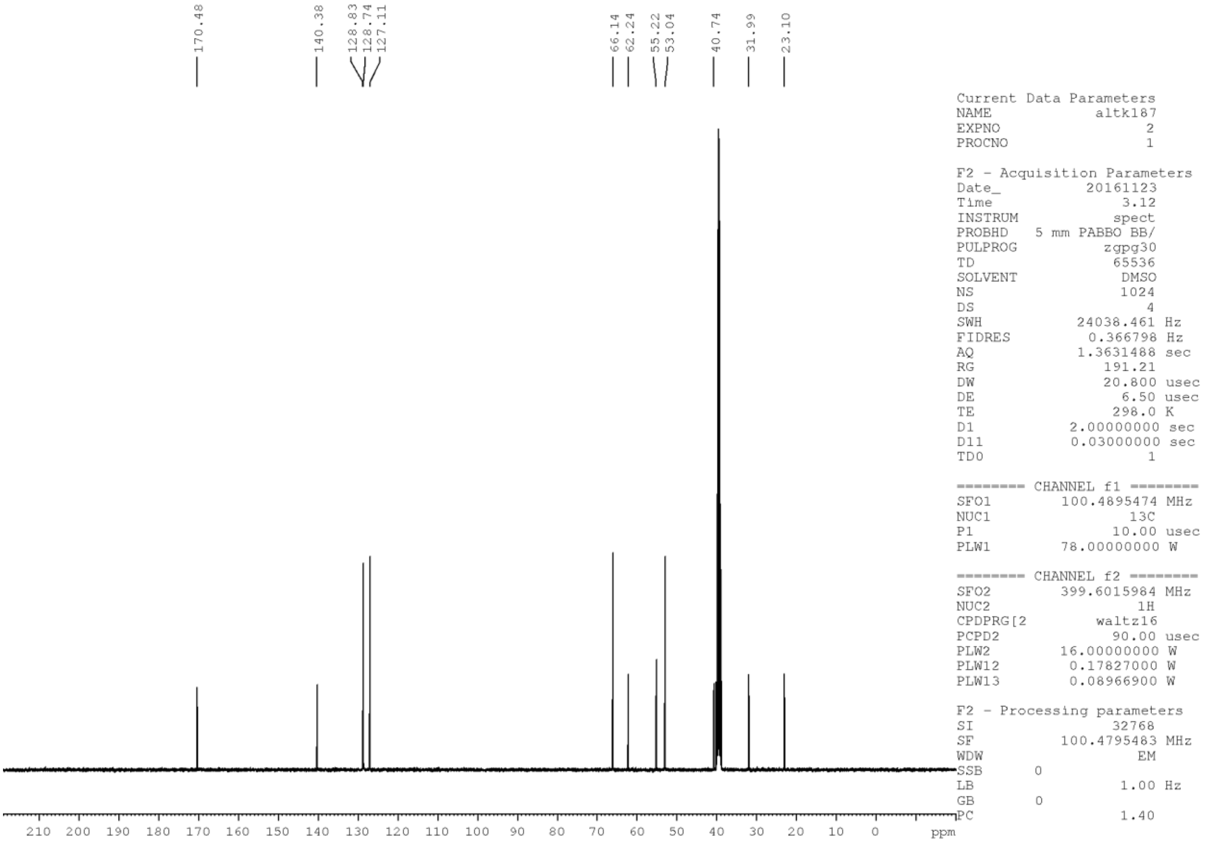
170.48 140.38 128.83 128.74 127.11 66.14 62.24 55.22 53.04 40.74 31.99 23.10
210 200 190 180 170 160 150 140 130 120 110 100 90 80 70 60 50 40 30 20 10 0 ppm
Current Data Parameters
NAME altk187
EXPNO 2
PROCNO 1
F2 - Acquisition Parameters
Date_ 20161123
Time 3.12
INSTRUM spect
PROBHD 5 mm PABBO BB/
PULPROG zgpg30
TD 65536
SOLVENT DMSO
NS 1024
DS 4
SWH 24038.461 Hz
FIDRES 0.366798 Hz
AQ 1.3631488 sec
RG 191.21
DW 20.800 usec
DE 6.50 usec
TE 298.0 K
D1 2.00000000 sec
D11 0.03000000 sec
TD0 1
======== CHANNEL f1 ========
SFO1 100.4895474 MHz
NUC1 13C
P1 10.00 usec
PLW1 78.00000000 W
======== CHANNEL f2 ========
SFO2 399.6015984 MHz
NUC2 1H
CPDPRG[2 waltz16
PCPD2 90.00 usec
PLW2 16.00000000 W
PLW12 0.17827000 W
PLW13 0.08966900 W
F2 - Processing parameters
SI 32768
SF 100.4795483 MHz
WDW EM
SSB 0
LB 1.00 Hz
GB 0
PC 1.40

9

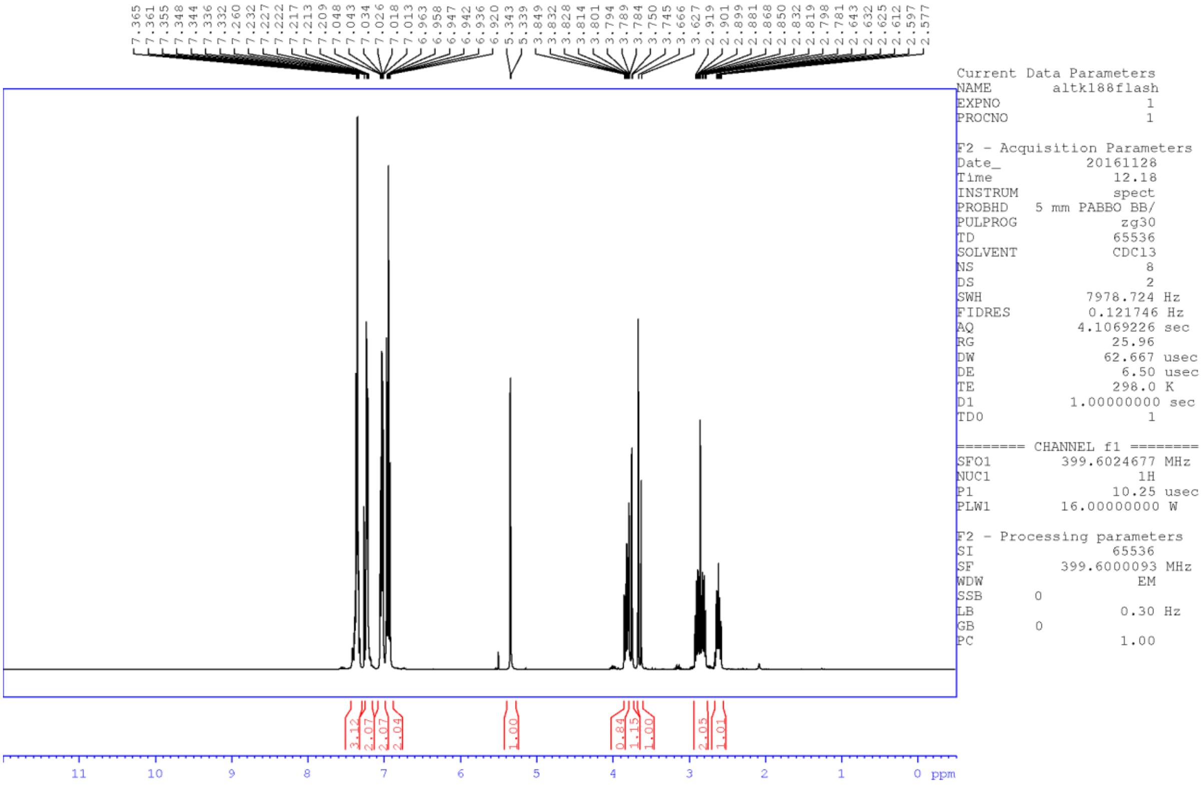

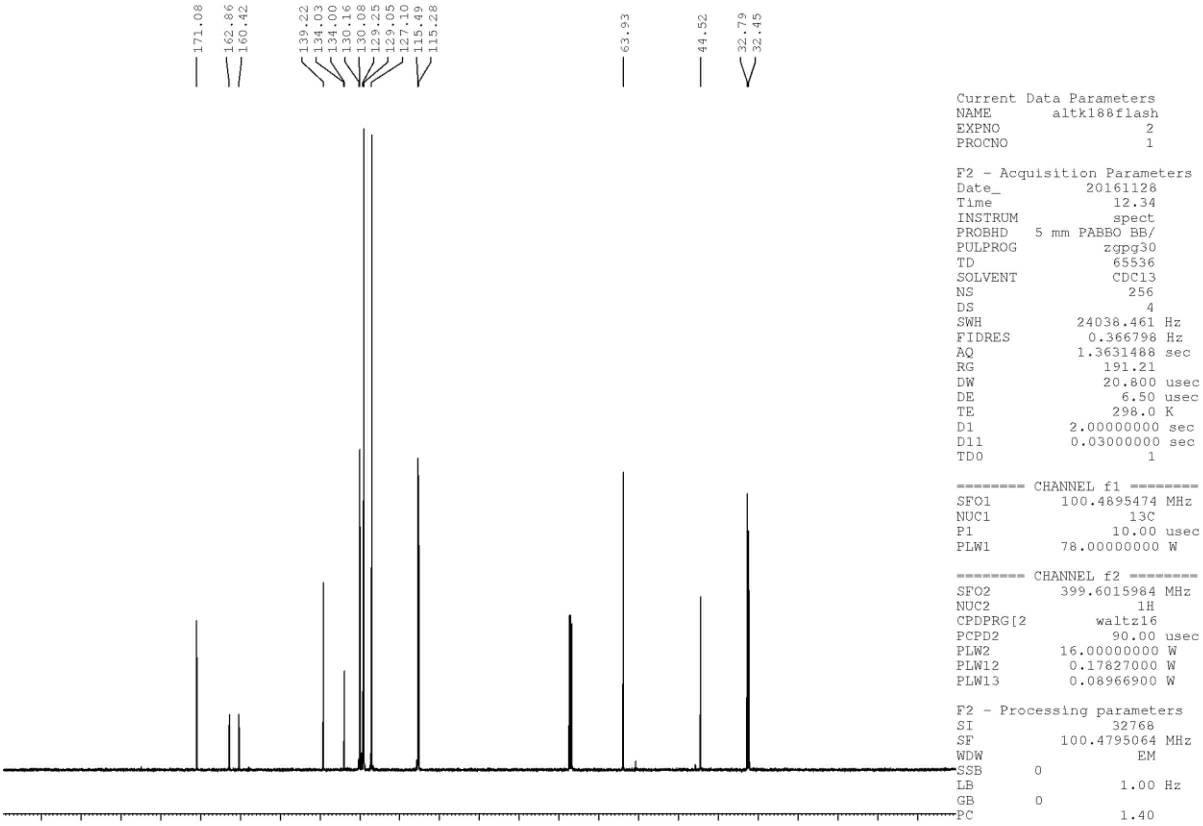

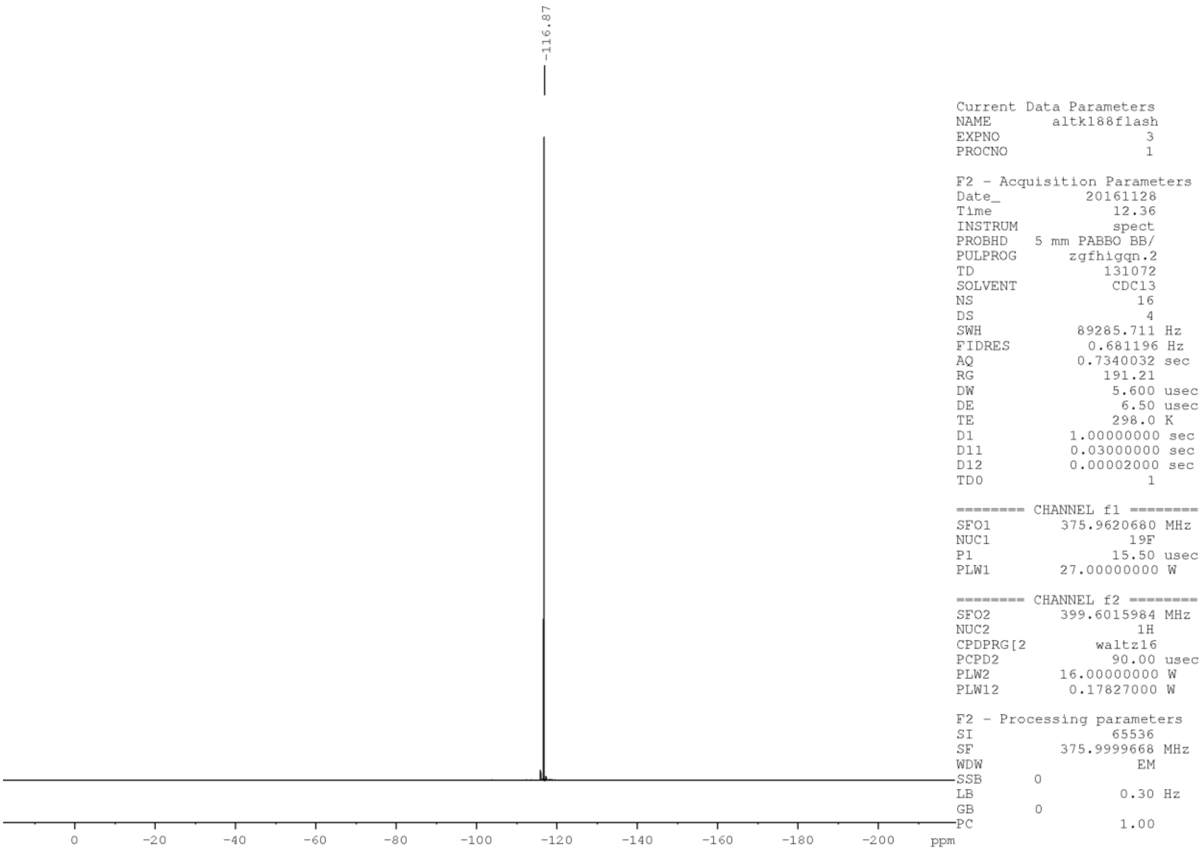

Current Data Parameters
NAME altk188flash
EXPNO 3
PROCNO 1

F2 - Acquisition Parameters
Date_ 20161128
Time 12.36
INSTRUM spect
PROBHD 5 mm PABBO BB/
PULPROG zgfhigqn.2
TD 131072
SOLVENT CDCl3
NS 16
DS 4
SWH 89285.711 Hz
FIDRES 0.681196 Hz
AQ 0.7340032 sec
RG 191.21
DW 5.600 usec
DE 6.50 usec
TE 298.0 K
D1 1.00000000 sec
D11 0.03000000 sec
D12 0.00002000 sec
TD0 1

======== CHANNEL f1 ========
SFO1 375.9620680 MHz
NUC1 19F
P1 15.50 usec
PLW1 27.00000000 W

======== CHANNEL f2 ========
SFO2 399.6015984 MHz
NUC2 1H
CPDPRG[2 waltz16
PCPD2 90.00 usec
PLW2 16.00000000 W
PLW12 0.17827000 W

F2 - Processing parameters
SI 65536
SF 375.9999668 MHz
WDW EM
SSB 0
LB 0.30 Hz
GB 0
PC 1.00

10

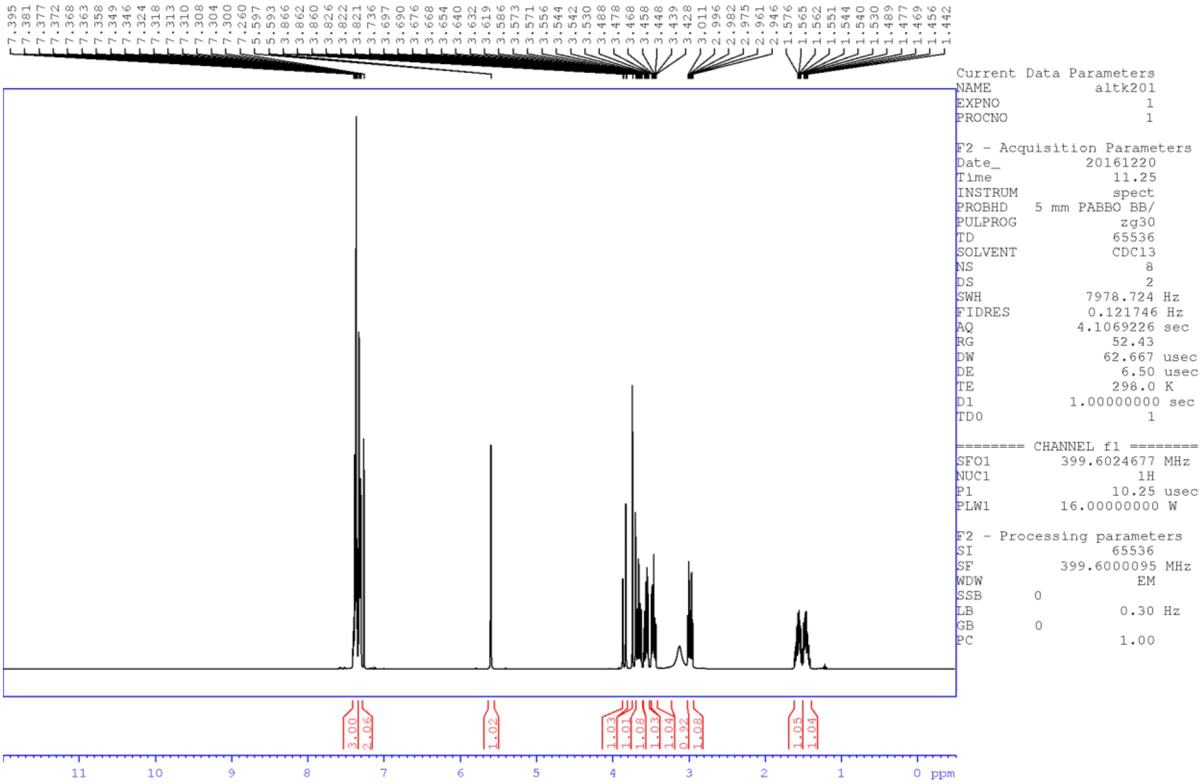

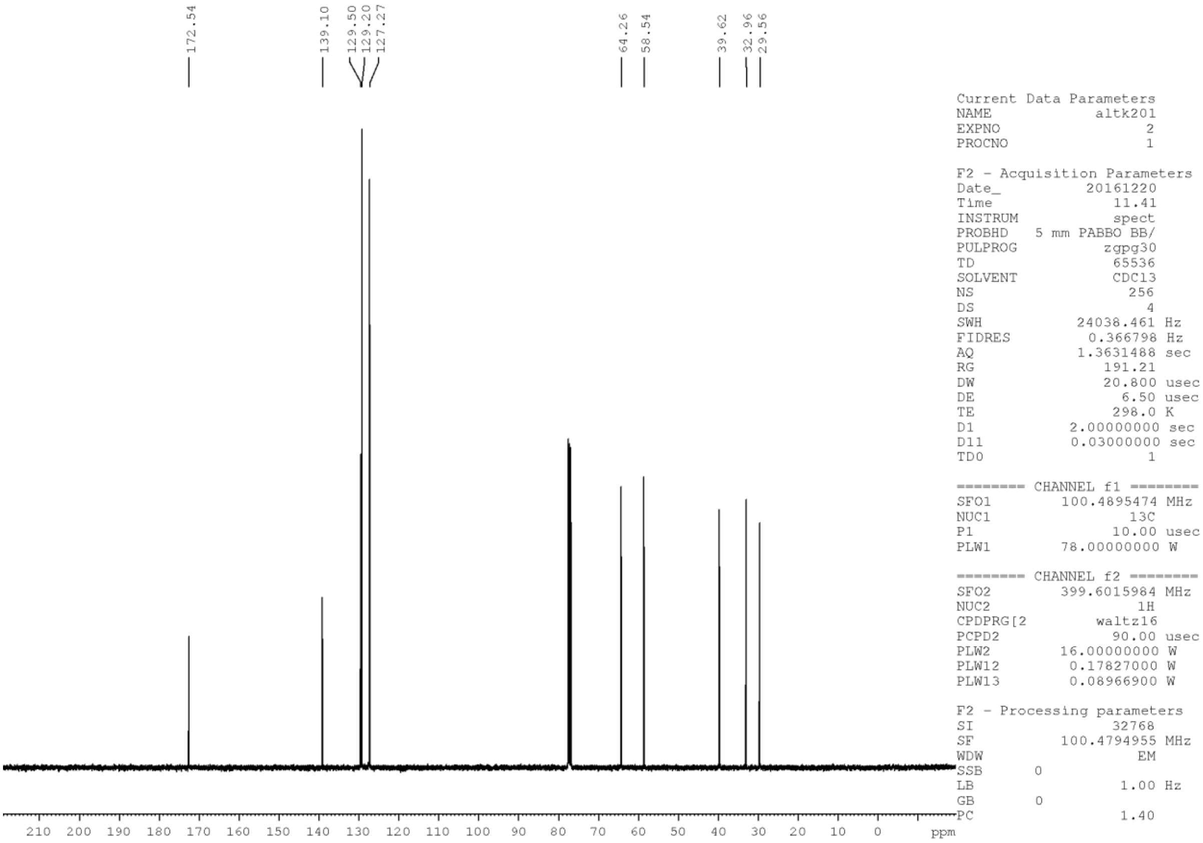

14

altk216fbcr.1.fid

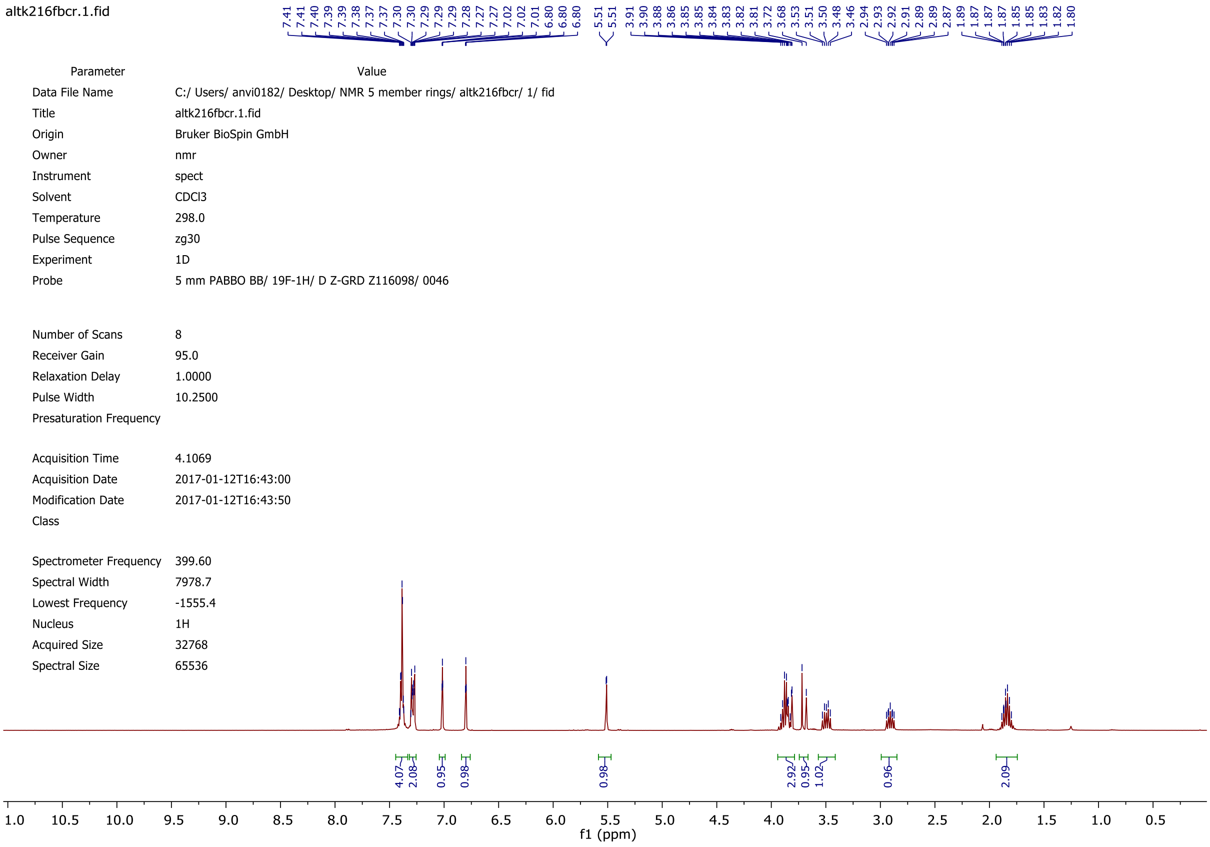

Parameter	Value
Data File Name	C:/ Users/ anvi0182/ Desktop/ NMR 5 member rings/ altk216fbcr/ 1/ fid
Title	altk216fbcr.1.fid
Origin	Bruker BioSpin GmbH
Owner	nmr
Instrument	spect
Solvent	CDCl3
Temperature	298.0
Pulse Sequence	zg30
Experiment	1D
Probe	5 mm PABBO BB/ 19F-1H/ D Z-GRD Z116098/ 0046
Number of Scans	8
Receiver Gain	95.0
Relaxation Delay	1.0000
Pulse Width	10.2500
Presaturation Frequency	
Acquisition Time	4.1069
Acquisition Date	2017-01-12T16:43:00
Modification Date	2017-01-12T16:43:50
Class	
Spectrometer Frequency	399.60
Spectral Width	7978.7
Lowest Frequency	-1555.4
Nucleus	1H
Acquired Size	32768
Spectral Size	65536

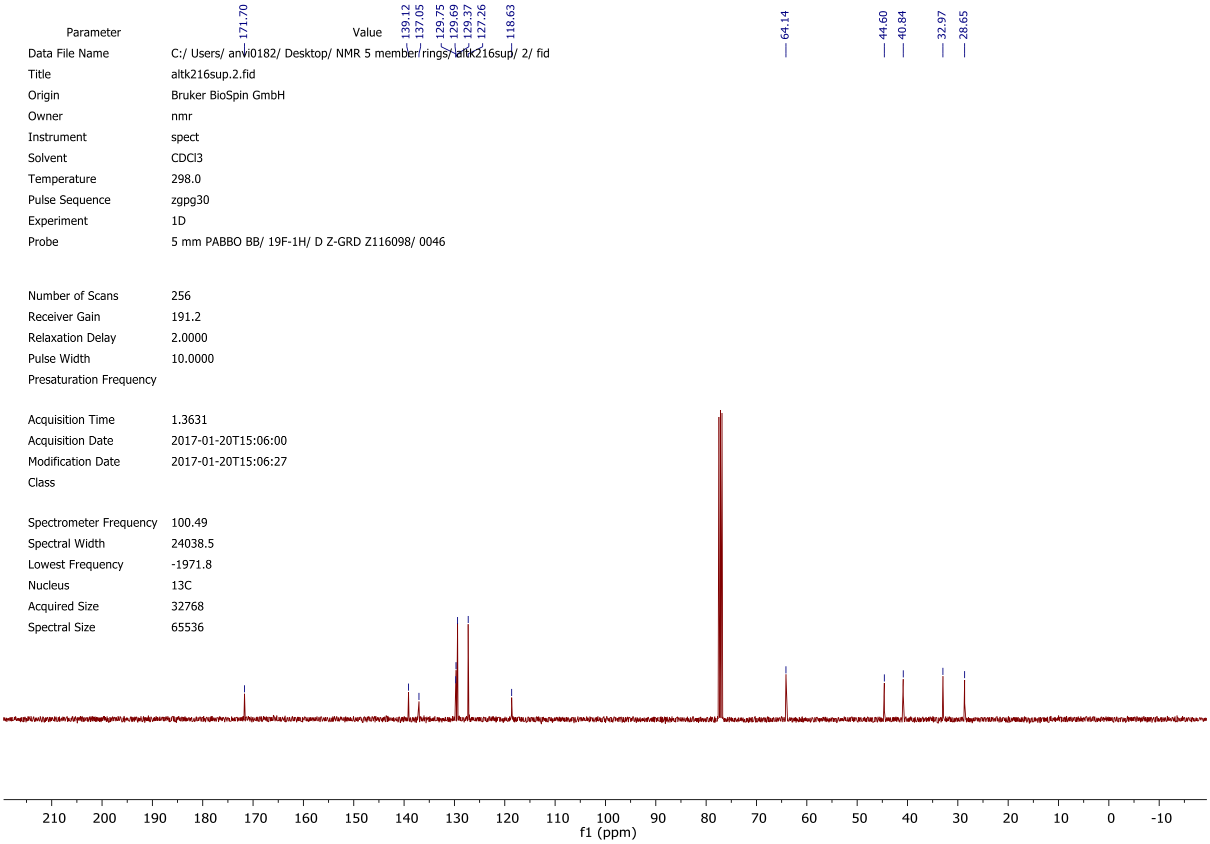

Parameter	Value
Data File Name	C:/ Users/ anvi0182/ Desktop/ NMR 5 member rings/ altk216sup/ 2/ fid
Title	altk216sup.2.fid
Origin	Bruker BioSpin GmbH
Owner	nmr
Instrument	spect
Solvent	CDCl3
Temperature	298.0
Pulse Sequence	zgpg30
Experiment	1D
Probe	5 mm PABBO BB/ 19F-1H/ D Z-GRD Z116098/ 0046
Number of Scans	256
Receiver Gain	191.2
Relaxation Delay	2.0000
Pulse Width	10.0000
Presaturation Frequency	
Acquisition Time	1.3631
Acquisition Date	2017-01-20T15:06:00
Modification Date	2017-01-20T15:06:27
Class	
Spectrometer Frequency	100.49
Spectral Width	24038.5
Lowest Frequency	-1971.8
Nucleus	13C
Acquired Size	32768
Spectral Size	65536

15

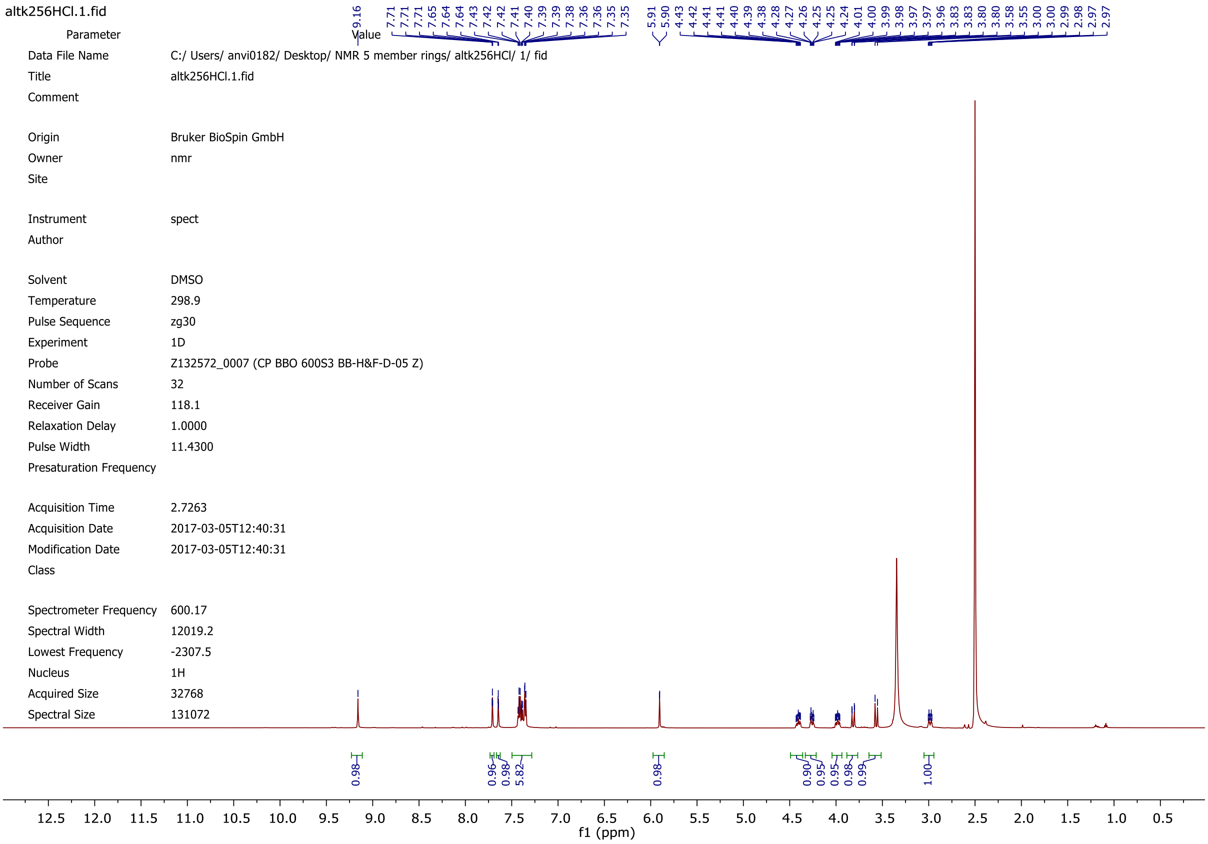
altk256HCl.1.fid
Parameter Value
Data File Name C:/ Users/ anvi0182/ Desktop/ NMR 5 member rings/ altk256HCl/ 1/ fid
Title altk256HCl.1.fid
Comment
Origin Bruker BioSpin GmbH
Owner nmr
Site
Instrument spect
Author
Solvent DMSO
Temperature 298.9
Pulse Sequence zg30
Experiment 1D
Probe Z132572_0007 (CP BBO 600S3 BB-H&F-D-05 Z)
Number of Scans 32
Receiver Gain 118.1
Relaxation Delay 1.0000
Pulse Width 11.4300
Presaturation Frequency
Acquisition Time 2.7263
Acquisition Date 2017-03-05T12:40:31
Modification Date 2017-03-05T12:40:31
Class
Spectrometer Frequency 600.17
Spectral Width 12019.2
Lowest Frequency -2307.5
Nucleus 1H
Acquired Size 32768
Spectral Size 131072
9.16 7.71 7.71 7.71 7.65 7.64 7.43 7.42 7.42 7.41 7.40 7.39 7.39 7.38 7.36 7.36 7.35 7.35 5.91 5.90 4.43 4.42 4.41 4.41 4.40 4.39 4.38 4.28 4.27 4.26 4.25 4.25 4.24 4.01 4.00 3.99 3.98 3.97 3.97 3.96 3.83 3.83 3.80 3.80 3.58 3.55 3.00 3.00 2.99 2.98 2.97 2.97
0.98 0.96 0.98 5.82 0.98 0.90 0.95 0.95 0.98 0.99 1.00
12.5 12.0 11.5 11.0 10.5 10.0 9.5 9.0 8.5 8.0 7.5 7.0 6.5 6.0 5.5 5.0 4.5 4.0 3.5 3.0 2.5 2.0 1.5 1.0 0.5
f1 (ppm)

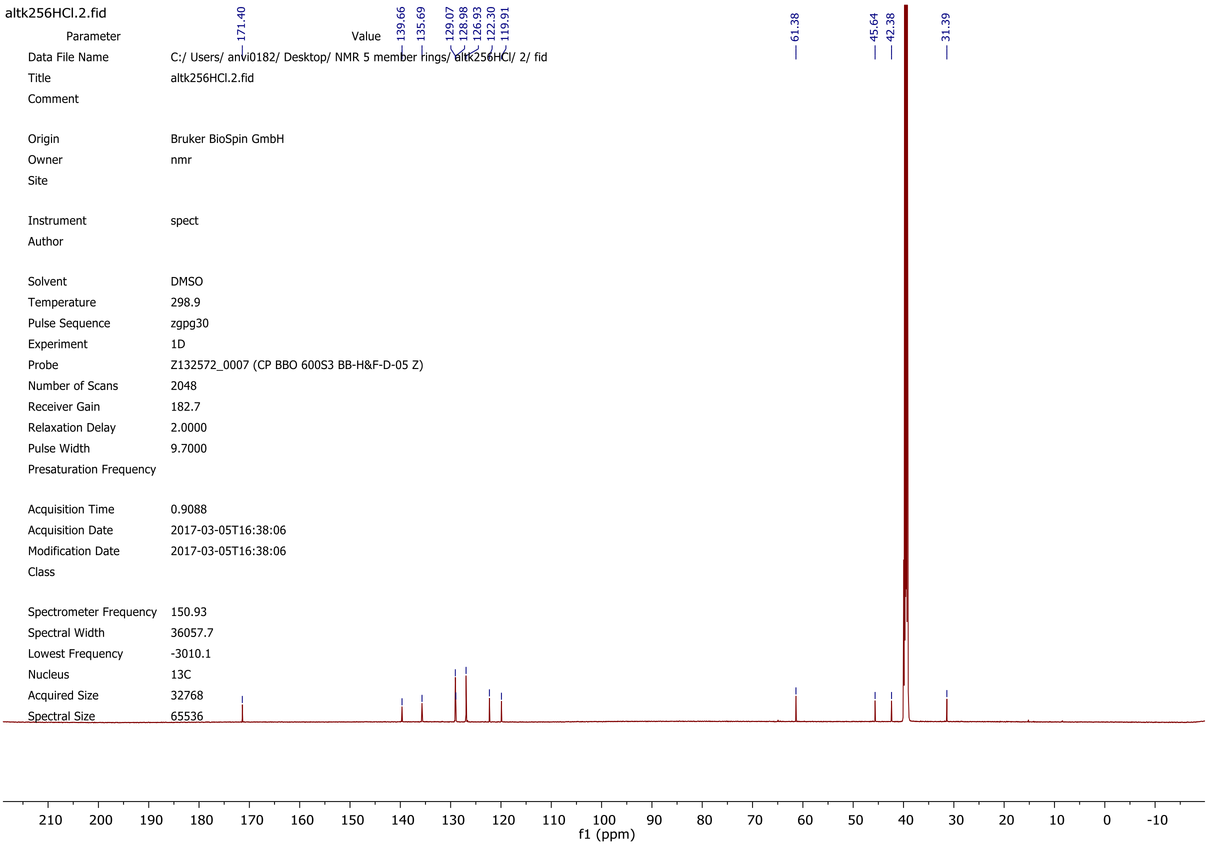
altk256HCl.2.fid
Parameter Value
Data File Name C:/ Users/ anvi0182/ Desktop/ NMR 5 member rings/ altk256HCl/ 2/ fid
Title altk256HCl.2.fid
Comment
Origin Bruker BioSpin GmbH
Owner nmr
Site
Instrument spect
Author
Solvent DMSO
Temperature 298.9
Pulse Sequence zgpg30
Experiment 1D
Probe Z132572_0007 (CP BBO 600S3 BB-H&F-D-05 Z)
Number of Scans 2048
Receiver Gain 182.7
Relaxation Delay 2.0000
Pulse Width 9.7000
Presaturation Frequency
Acquisition Time 0.9088
Acquisition Date 2017-03-05T16:38:06
Modification Date 2017-03-05T16:38:06
Class
Spectrometer Frequency 150.93
Spectral Width 36057.7
Lowest Frequency -3010.1
Nucleus 13C
Acquired Size 32768
Spectral Size 65536
171.40 139.66 135.69 129.07 128.98 126.93 122.30 119.91 61.38 45.64 42.38 31.39
210 200 190 180 170 160 150 140 130 120 110 100 90 80 70 60 50 40 30 20 10 0 -10
f1 (ppm)

16

altk257cr.1.fid

Parameter	Value
Data File Name	C:/ Users/ anvi0182/ Desktop/ NMR 5 member rings/ altk257cr/ 1/ fid
Title	altk257cr.1.fid
Comment	
Origin	Bruker BioSpin GmbH
Owner	nmr
Site	
Instrument	spect
Author	
Solvent	CDCl3
Temperature	298.0
Pulse Sequence	zg30
Experiment	1D
Probe	Z116098_0046 (PA BBO 400S1 BBF-H-D-05 Z SP)
Number of Scans	8
Receiver Gain	107.6
Relaxation Delay	1.0000
Pulse Width	10.2500
Presaturation Frequency	
Acquisition Time	4.1069
Acquisition Date	2017-03-03T15:51:11
Modification Date	2017-03-03T15:51:11
Class	
Spectrometer Frequency	399.60
Spectral Width	7978.7
Lowest Frequency	-1531.3
Nucleus	1H
Acquired Size	32768
Spectral Size	65536

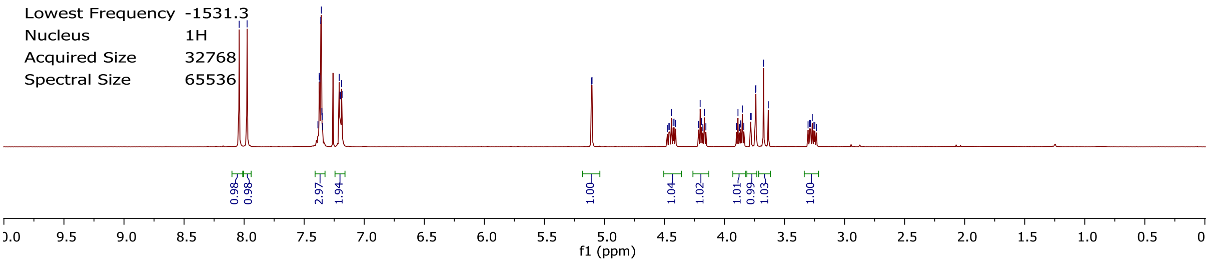

Parameter	Value
Data File Name	C:/ Users/ anvi0182/ Desktop/ NMR 5 member rings/ altk257cr/ 2/ fid
Title	altk257cr.2.fid
Comment	
Origin	Bruker BioSpin GmbH
Owner	nmr
Site	
Instrument	spect
Author	
Solvent	CDCl3
Temperature	298.0
Pulse Sequence	zgpg30
Experiment	1D
Probe	Z116098_0046 (PA BBO 400S1 BBF-H-D-05 Z SP)
Number of Scans	256
Receiver Gain	191.2
Relaxation Delay	2.0000
Pulse Width	10.0000
Presaturation Frequency	
Acquisition Time	1.3631
Acquisition Date	2017-03-03T16:07:09
Modification Date	2017-03-03T16:07:09
Class	
Spectrometer Frequency	100.49
Spectral Width	24038.5
Lowest Frequency	-1960.8
Nucleus	13C
Acquired Size	32768
Spectral Size	65536

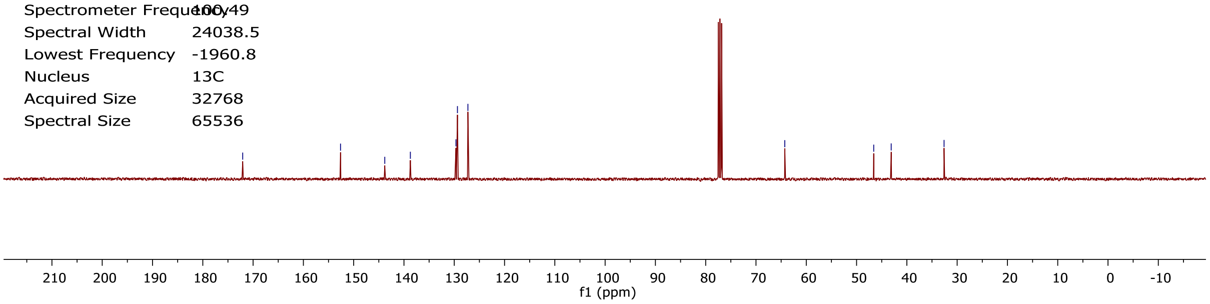

17

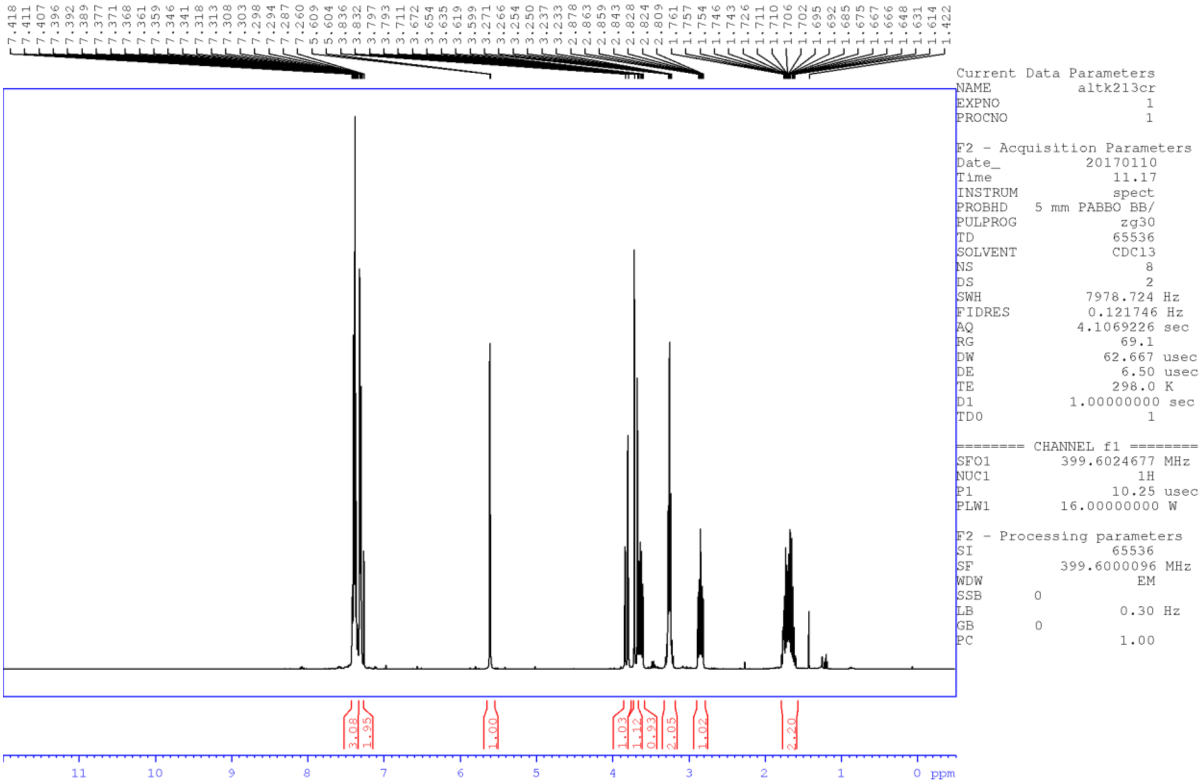
7.418 7.411 7.407 7.396 7.392 7.389 7.377 7.371 7.368 7.361 7.359 7.346 7.341 7.318 7.313 7.308 7.303 7.298 7.294 7.287 7.260 5.609 5.604 3.836 3.832 3.797 3.793 3.711 3.672 3.654 3.635 3.619 3.599 3.271 3.266 3.254 3.250 3.237 3.233 2.878 2.863 2.859 2.843 2.828 2.824 2.809 1.761 1.757 1.754 1.746 1.743 1.726 1.711 1.710 1.706 1.702 1.695 1.692 1.685 1.675 1.667 1.666 1.648 1.631 1.614 1.422
3.08 1.95 1.00 1.03 1.12 0.93 2.05 1.02 2.20
11 10 9 8 7 6 5 4 3 2 1 0 ppm
Current Data Parameters
NAME altk213cr
EXPNO 1
PROCNO 1
F2 - Acquisition Parameters
Date_ 20170110
Time 11.17
INSTRUM spect
PROBHD 5 mm PABBO BB/
PULPROG zg30
TD 65536
SOLVENT CDCl3
NS 8
DS 2
SWH 7978.724 Hz
FIDRES 0.121746 Hz
AQ 4.1069226 sec
RG 69.1
DW 62.667 usec
DE 6.50 usec
TE 298.0 K
D1 1.00000000 sec
TD0 1
======== CHANNEL f1 ========
SFO1 399.6024677 MHz
NUC1 1H
P1 10.25 usec
PLW1 16.00000000 W
F2 - Processing parameters
SI 65536
SF 399.6000096 MHz
WDW EM
SSB 0
LB 0.30 Hz
GB 0
PC 1.00

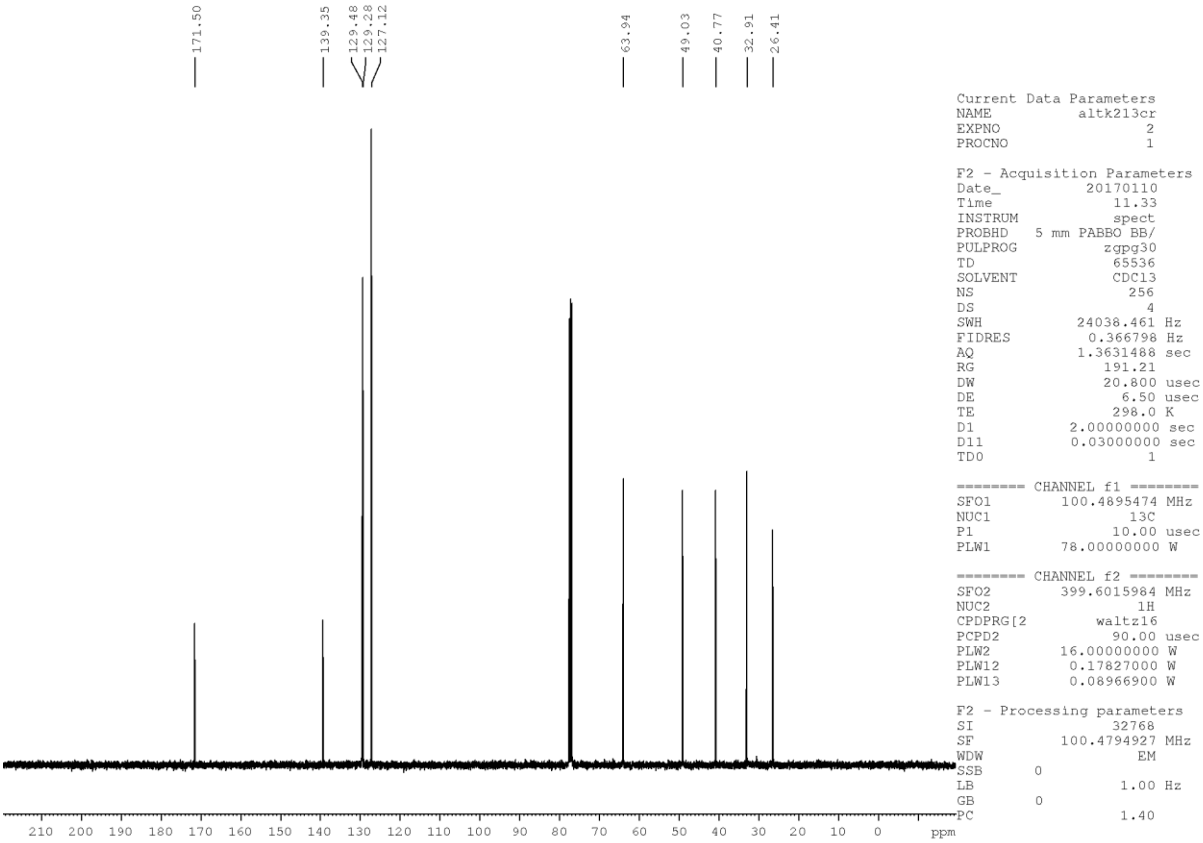
171.50 139.35 129.48 129.28 127.12 63.94 49.03 40.77 32.91 26.41
210 200 190 180 170 160 150 140 130 120 110 100 90 80 70 60 50 40 30 20 10 0 ppm
Current Data Parameters
NAME altk213cr
EXPNO 2
PROCNO 1
F2 - Acquisition Parameters
Date_ 20170110
Time 11.33
INSTRUM spect
PROBHD 5 mm PABBO BB/
PULPROG zgpg30
TD 65536
SOLVENT CDCl3
NS 256
DS 4
SWH 24038.461 Hz
FIDRES 0.366798 Hz
AQ 1.3631488 sec
RG 191.21
DW 20.800 usec
DE 6.50 usec
TE 298.0 K
D1 2.00000000 sec
D11 0.03000000 sec
TD0 1
======== CHANNEL f1 ========
SFO1 100.4895474 MHz
NUC1 13C
P1 10.00 usec
PLW1 78.00000000 W
======== CHANNEL f2 ========
SFO2 399.6015984 MHz
NUC2 1H
CPDPRG[2 waltz16
PCPD2 90.00 usec
PLW2 16.00000000 W
PLW12 0.17827000 W
PLW13 0.08966900 W
F2 - Processing parameters
SI 32768
SF 100.4794927 MHz
WDW EM
SSB 0
LB 1.00 Hz
GB 0
PC 1.40

18

AL209 full charac MeOD.1.fid

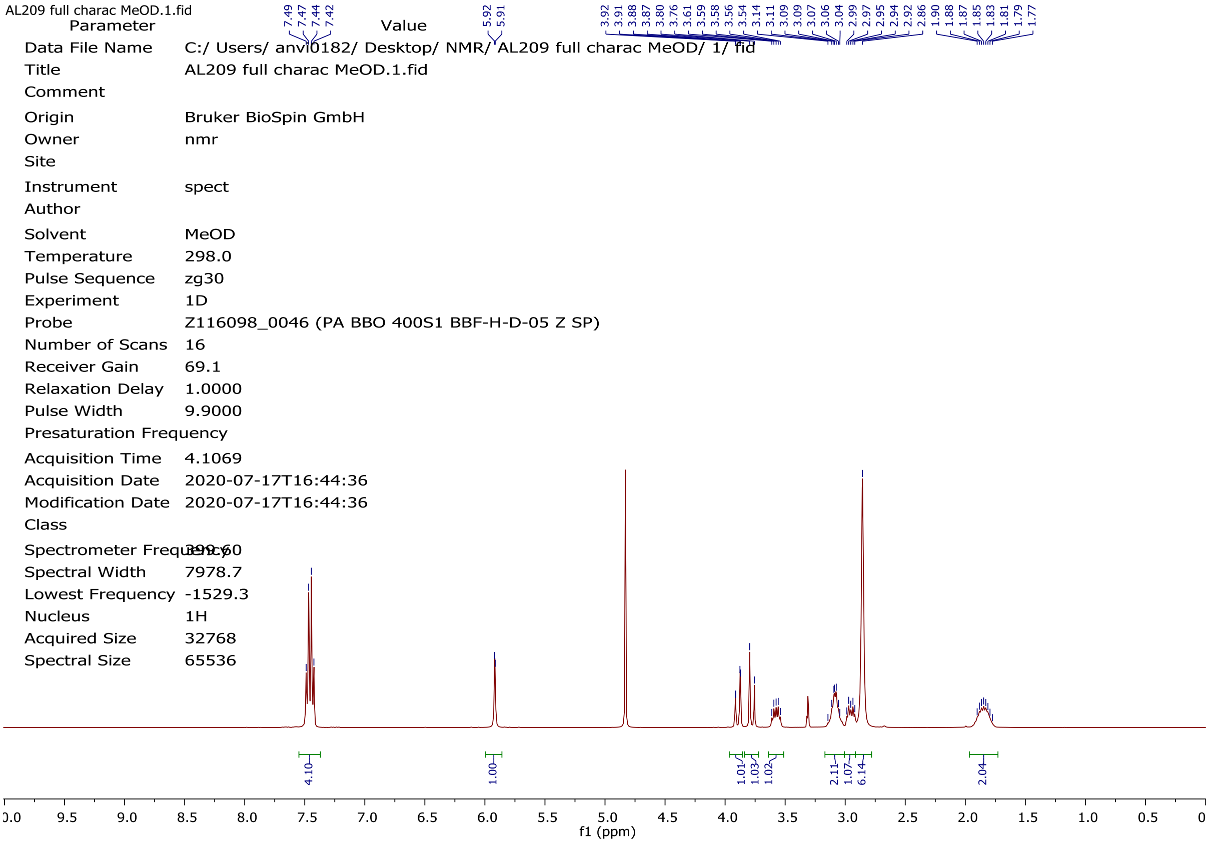

Parameter	Value
Data File Name	C:/ Users/ anvi0182/ Desktop/ NMR/ AL209 full charac MeOD/ 1/ fid
Title	AL209 full charac MeOD.1.fid
Comment	
Origin	Bruker BioSpin GmbH
Owner	nmr
Site	
Instrument	spect
Author	
Solvent	MeOD
Temperature	298.0
Pulse Sequence	zg30
Experiment	1D
Probe	Z116098_0046 (PA BBO 400S1 BBF-H-D-05 Z SP)
Number of Scans	16
Receiver Gain	69.1
Relaxation Delay	1.0000
Pulse Width	9.9000
Presaturation Frequency	
Acquisition Time	4.1069
Acquisition Date	2020-07-17T16:44:36
Modification Date	2020-07-17T16:44:36
Class	
Spectrometer Frequency	399.60
Spectral Width	7978.7
Lowest Frequency	-1529.3
Nucleus	1H
Acquired Size	32768
Spectral Size	65536

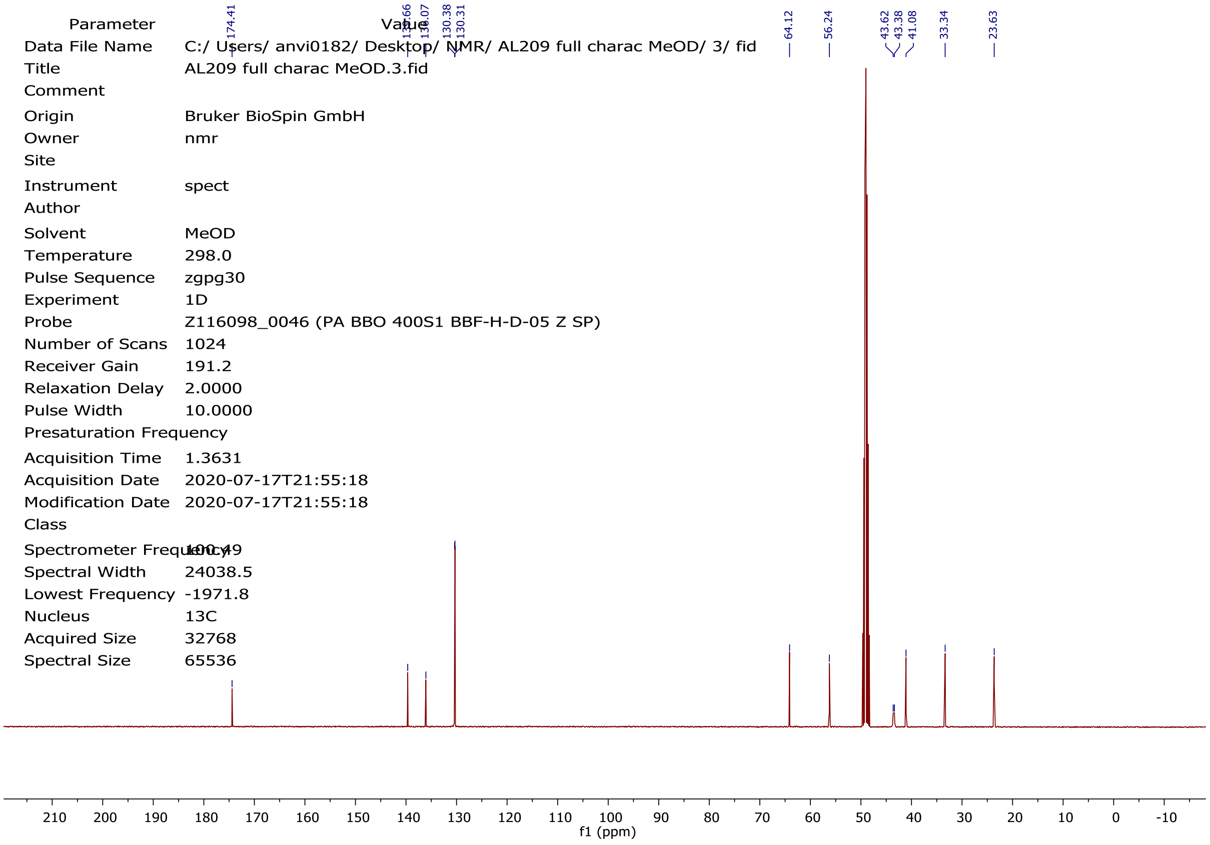

Parameter	Value
Data File Name	C:/ Users/ anvi0182/ Desktop/ NMR/ AL209 full charac MeOD/ 3/ fid
Title	AL209 full charac MeOD.3.fid
Comment	
Origin	Bruker BioSpin GmbH
Owner	nmr
Site	
Instrument	spect
Author	
Solvent	MeOD
Temperature	298.0
Pulse Sequence	zgpg30
Experiment	1D
Probe	Z116098_0046 (PA BBO 400S1 BBF-H-D-05 Z SP)
Number of Scans	1024
Receiver Gain	191.2
Relaxation Delay	2.0000
Pulse Width	10.0000
Presaturation Frequency	
Acquisition Time	1.3631
Acquisition Date	2020-07-17T21:55:18
Modification Date	2020-07-17T21:55:18
Class	
Spectrometer Frequency	100.49
Spectral Width	24038.5
Lowest Frequency	-1971.8
Nucleus	13C
Acquired Size	32768
Spectral Size	65536

19

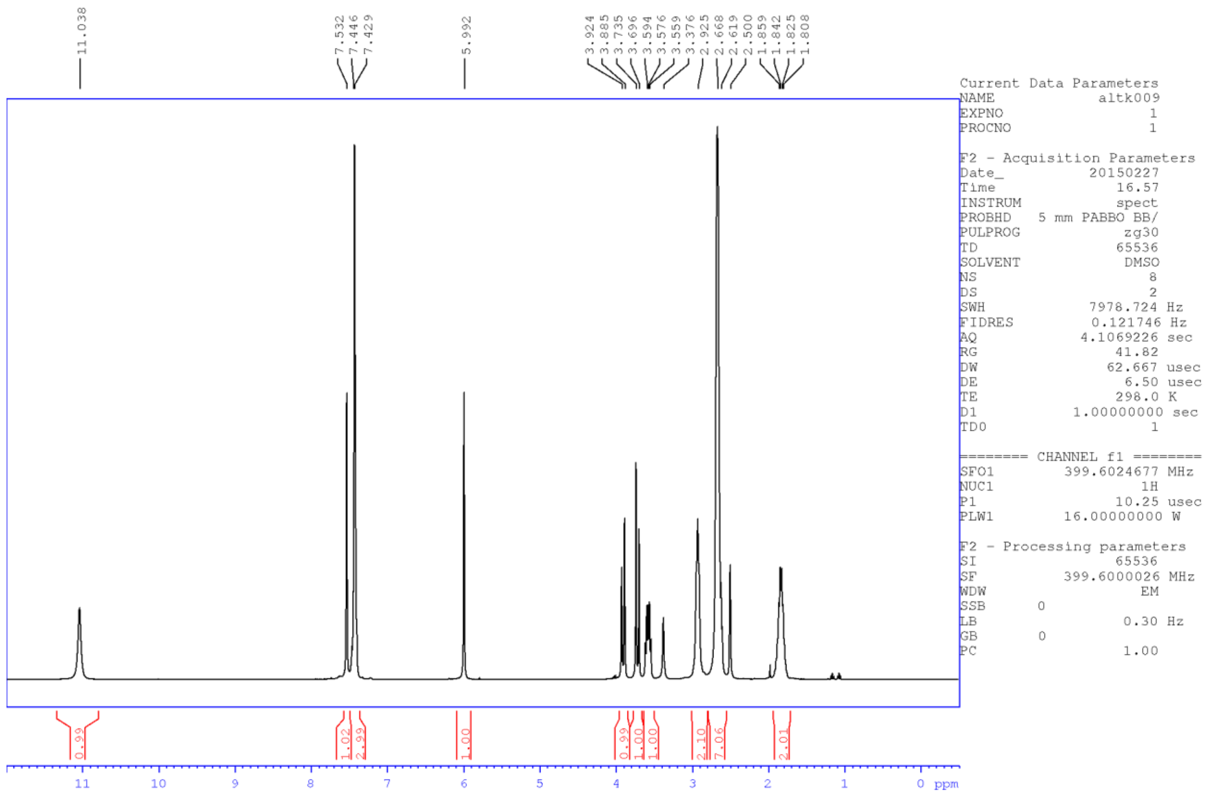
11.038
7.532
7.446
7.429
5.992
3.924
3.885
3.735
3.696
3.594
3.576
3.559
3.376
2.925
2.668
2.619
2.500
1.859
1.842
1.825
1.808
0.99
1.02
2.99
1.00
0.99
1.00
1.00
2.10
7.06
2.01
11
10
9
8
7
6
5
4
3
2
1
0 ppm
Current Data Parameters
NAME altk009
EXPNO 1
PROCNO 1
F2 - Acquisition Parameters
Date_ 20150227
Time 16.57
INSTRUM spect
PROBHD 5 mm PABBO BB/
PULPROG zg30
TD 65536
SOLVENT DMSO
NS 8
DS 2
SWH 7978.724 Hz
FIDRES 0.121746 Hz
AQ 4.1069226 sec
RG 41.82
DW 62.667 usec
DE 6.50 usec
TE 298.0 K
D1 1.00000000 sec
TD0 1
======== CHANNEL f1 ========
SFO1 399.6024677 MHz
NUC1 1H
P1 10.25 usec
PLW1 16.00000000 W
F2 - Processing parameters
SI 65536
SF 399.6000026 MHz
WDW EM
SSB 0
LB 0.30 Hz
GB 0
PC 1.00

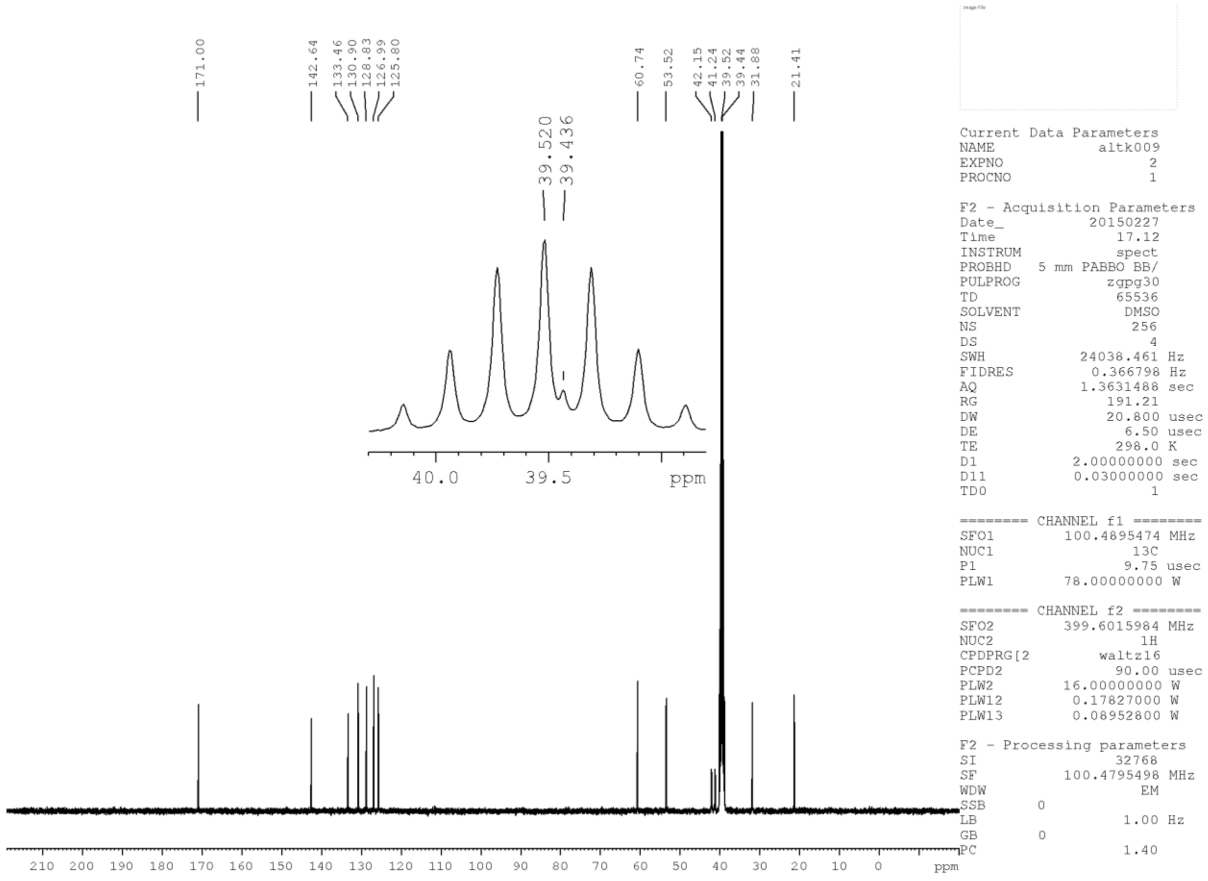
171.00
142.64
133.46
130.90
128.83
126.99
125.80
60.74
53.52
42.15
41.24
39.52
39.44
31.88
21.41
39.520
39.436
40.0
39.5
ppm
210
200
190
180
170
160
150
140
130
120
110
100
90
80
70
60
50
40
30
20
10
0
ppm
Current Data Parameters
NAME altk009
EXPNO 2
PROCNO 1
F2 - Acquisition Parameters
Date_ 20150227
Time 17.12
INSTRUM spect
PROBHD 5 mm PABBO BB/
PULPROG zgpg30
TD 65536
SOLVENT DMSO
NS 256
DS 4
SWH 24038.461 Hz
FIDRES 0.366798 Hz
AQ 1.3631488 sec
RG 191.21
DW 20.800 usec
DE 6.50 usec
TE 298.0 K
D1 2.00000000 sec
D11 0.03000000 sec
TD0 1
======== CHANNEL f1 ========
SFO1 100.4895474 MHz
NUC1 13C
P1 9.75 usec
PLW1 78.00000000 W
======== CHANNEL f2 ========
SFO2 399.6015984 MHz
NUC2 1H
CPDPRG[2 waltz16
PCPD2 90.00 usec
PLW2 16.00000000 W
PLW12 0.17827000 W
PLW13 0.08952800 W
F2 - Processing parameters
SI 32768
SF 100.4795498 MHz
WDW EM
SSB 0
LB 1.00 Hz
GB 0
PC 1.40

20

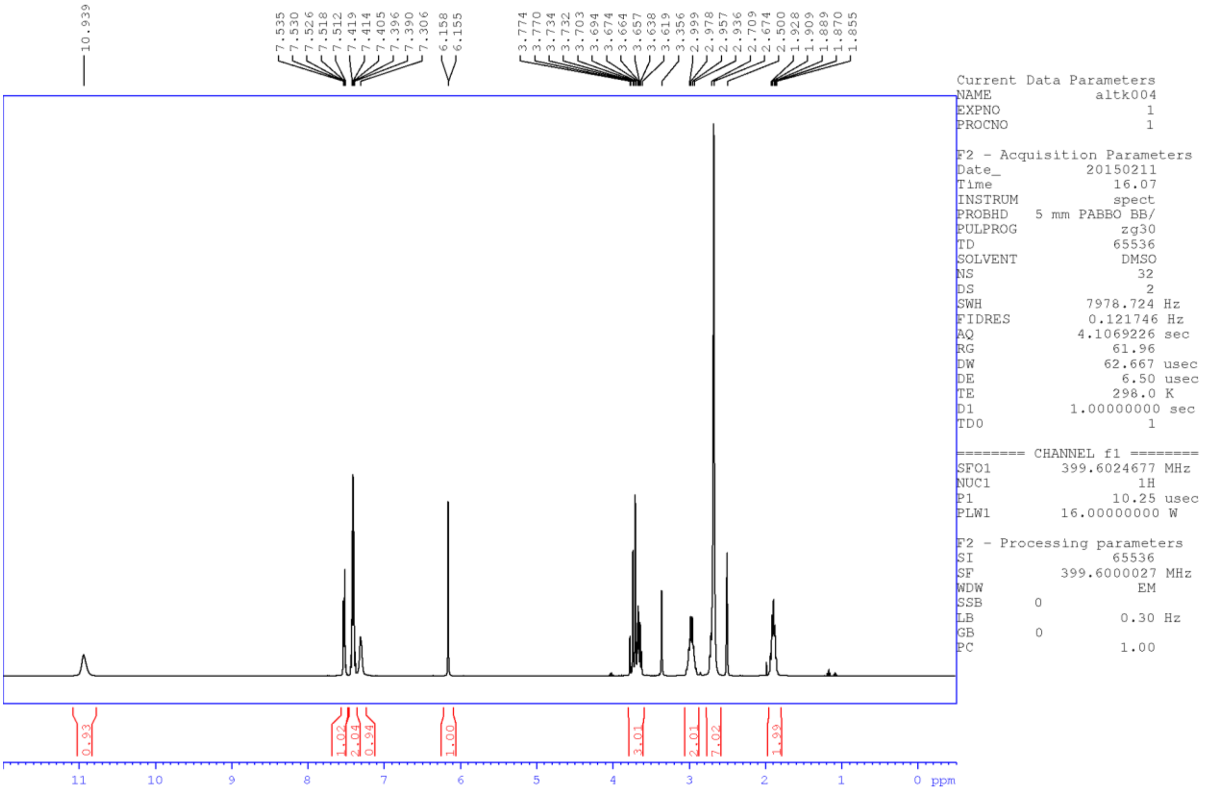
10.939
7.535 7.530 7.526 7.518 7.512 7.419 7.414 7.405 7.396 7.390 7.306
6.158 6.155
3.774 3.770 3.734 3.732 3.703 3.694 3.674 3.664 3.657 3.638 3.619 3.356 2.999 2.978 2.957 2.936 2.709 2.674 2.500 1.928 1.909 1.889 1.870 1.855
0.93 1.02 2.04 0.94 1.00 3.01 2.01 7.02 1.99
11 10 9 8 7 6 5 4 3 2 1 0 ppm
Current Data Parameters
NAME altk004
EXPNO 1
PROCNO 1
F2 - Acquisition Parameters
Date_ 20150211
Time 16.07
INSTRUM spect
PROBHD 5 mm PABBO BB/
PULPROG zg30
TD 65536
SOLVENT DMSO
NS 32
DS 2
SWH 7978.724 Hz
FIDRES 0.121746 Hz
AQ 4.1069226 sec
RG 61.96
DW 62.667 usec
DE 6.50 usec
TE 298.0 K
D1 1.00000000 sec
TD0 1
======== CHANNEL f1 ========
SFO1 399.6024677 MHz
NUC1 1H
P1 10.25 usec
PLW1 16.00000000 W
F2 - Processing parameters
SI 65536
SF 399.6000027 MHz
WDW EM
SSB 0
LB 0.30 Hz
GB 0
PC 1.00

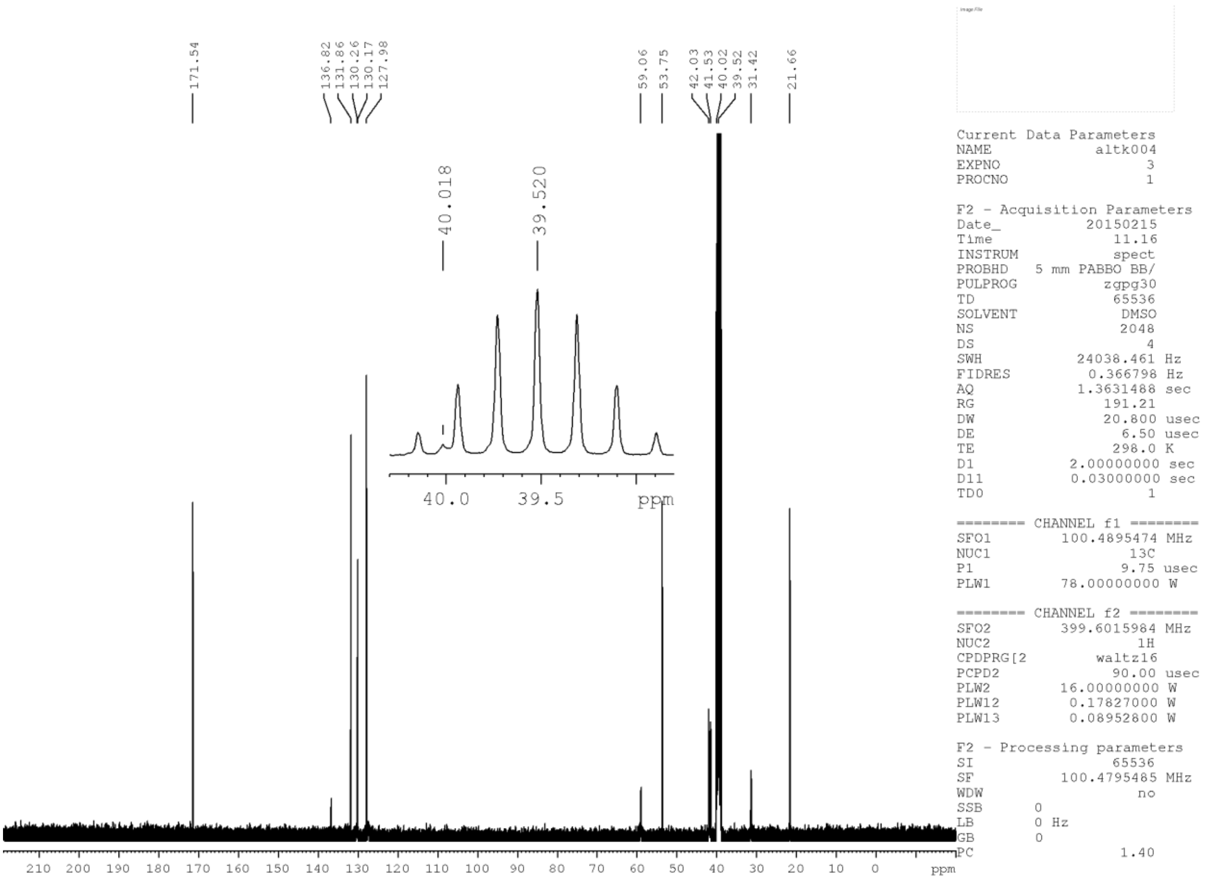
171.54
136.82 131.86 130.26 130.17 127.98
59.06 53.75 42.03 41.53 40.02 39.52 31.42 21.66
40.018
39.520
40.0 39.5 ppm
210 200 190 180 170 160 150 140 130 120 110 100 90 80 70 60 50 40 30 20 10 0 ppm
Current Data Parameters
NAME altk004
EXPNO 3
PROCNO 1
F2 - Acquisition Parameters
Date_ 20150215
Time 11.16
INSTRUM spect
PROBHD 5 mm PABBO BB/
PULPROG zgpg30
TD 65536
SOLVENT DMSO
NS 2048
DS 4
SWH 24038.461 Hz
FIDRES 0.366798 Hz
AQ 1.3631488 sec
RG 191.21
DW 20.800 usec
DE 6.50 usec
TE 298.0 K
D1 2.00000000 sec
D11 0.03000000 sec
TD0 1
======== CHANNEL f1 ========
SFO1 100.4895474 MHz
NUC1 13C
P1 9.75 usec
PLW1 78.00000000 W
======== CHANNEL f2 ========
SFO2 399.6015984 MHz
NUC2 1H
CPDPRG[2 waltz16
PCPD2 90.00 usec
PLW2 16.00000000 W
PLW12 0.17827000 W
PLW13 0.08952800 W
F2 - Processing parameters
SI 65536
SF 100.4795485 MHz
WDW no
SSB 0
LB 0 Hz
GB 0
PC 1.40

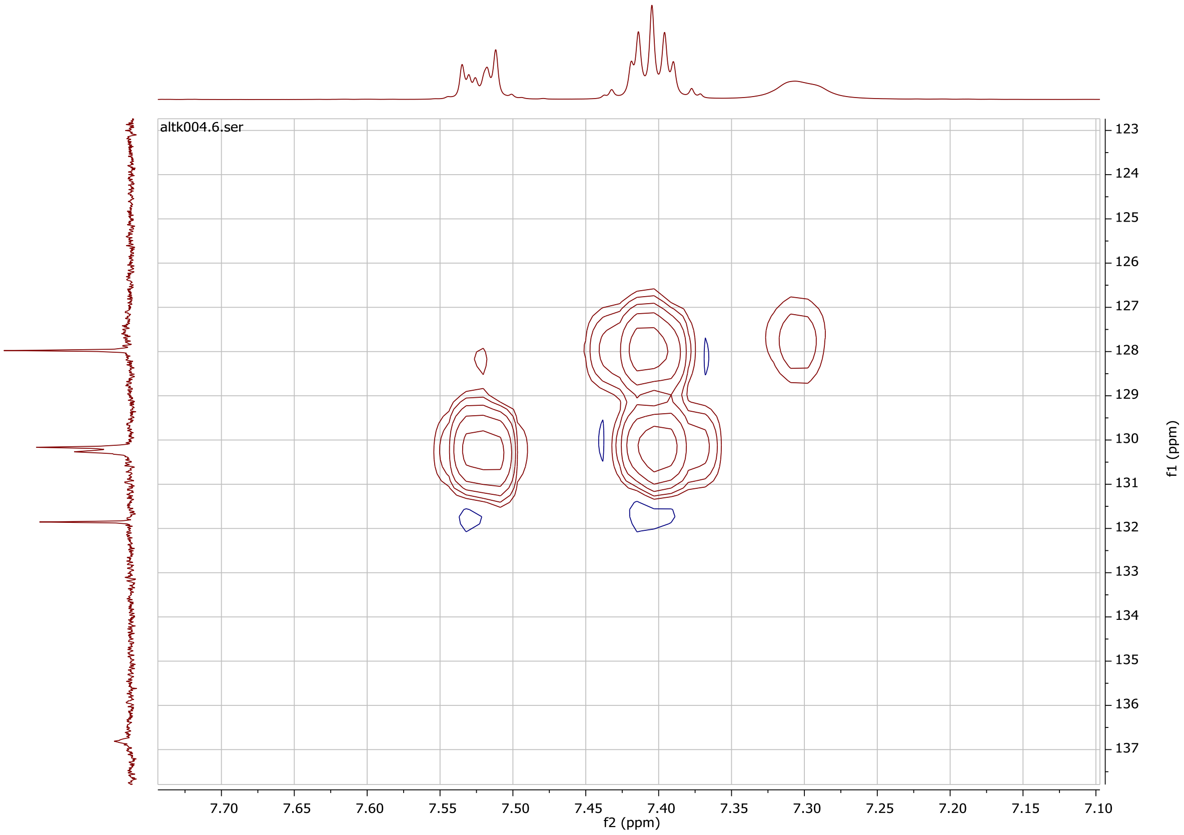
altk004.6.ser
7.70
7.65
7.60
7.55
7.50
7.45
7.40
7.35
7.30
7.25
7.20
7.15
7.10
f2 (ppm)
123
124
125
126
127
128
129
130
131
132
133
134
135
136
137
f1 (ppm)

21

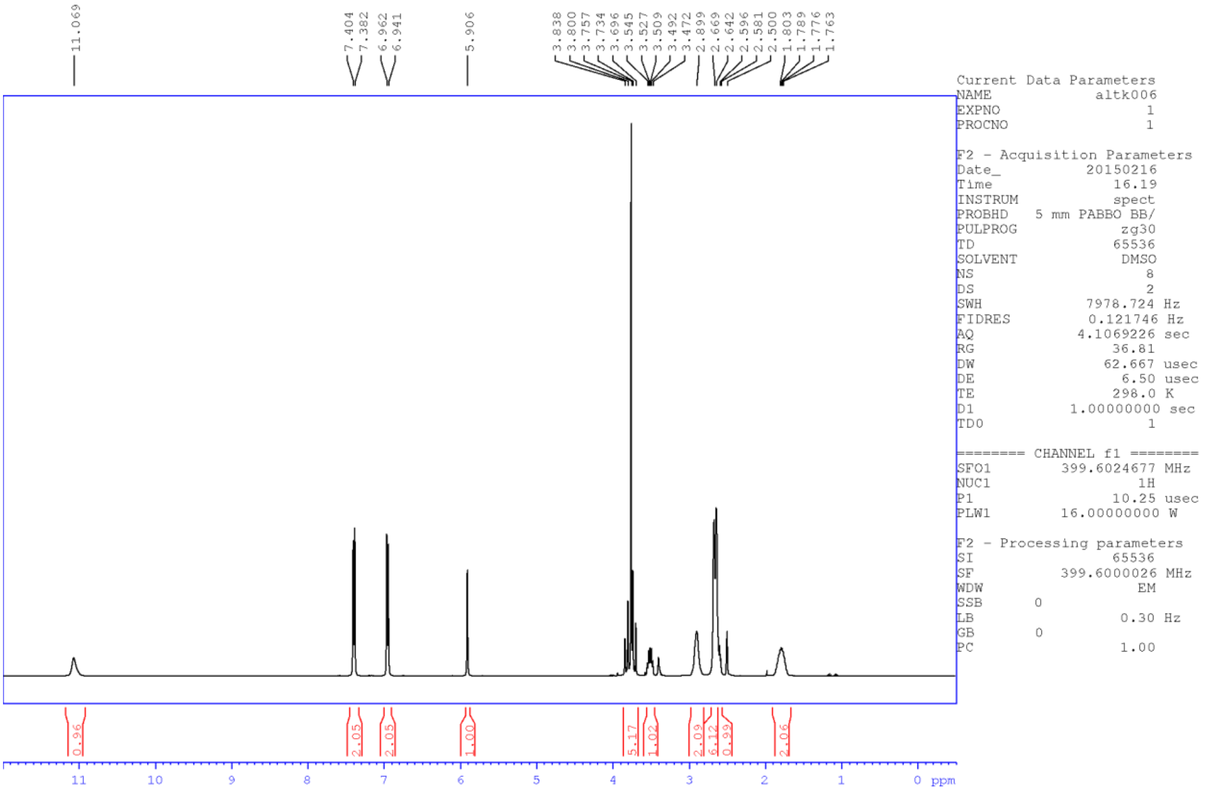
11.069
7.404
7.382
6.962
6.941
5.906
3.838
3.800
3.757
3.734
3.696
3.545
3.527
3.509
3.492
3.472
2.899
2.669
2.642
2.596
2.581
2.500
1.803
1.789
1.776
1.763
Current Data Parameters
NAME altk006
EXPNO 1
PROCNO 1
F2 - Acquisition Parameters
Date_ 20150216
Time 16.19
INSTRUM spect
PROBHD 5 mm PABBO BB/
PULPROG zg30
TD 65536
SOLVENT DMSO
NS 8
DS 2
SWH 7978.724 Hz
FIDRES 0.121746 Hz
AQ 4.1069226 sec
RG 36.81
DW 62.667 usec
DE 6.50 usec
TE 298.0 K
D1 1.00000000 sec
TD0 1
======== CHANNEL f1 ========
SFO1 399.6024677 MHz
NUC1 1H
P1 10.25 usec
PLW1 16.00000000 W
F2 - Processing parameters
SI 65536
SF 399.6000026 MHz
WDW EM
SSB 0
LB 0.30 Hz
GB 0
PC 1.00
0.96
2.05
2.05
1.00
5.17
1.02
2.09
6.12
0.99
2.06
11
10
9
8
7
6
5
4
3
2
1
0 ppm

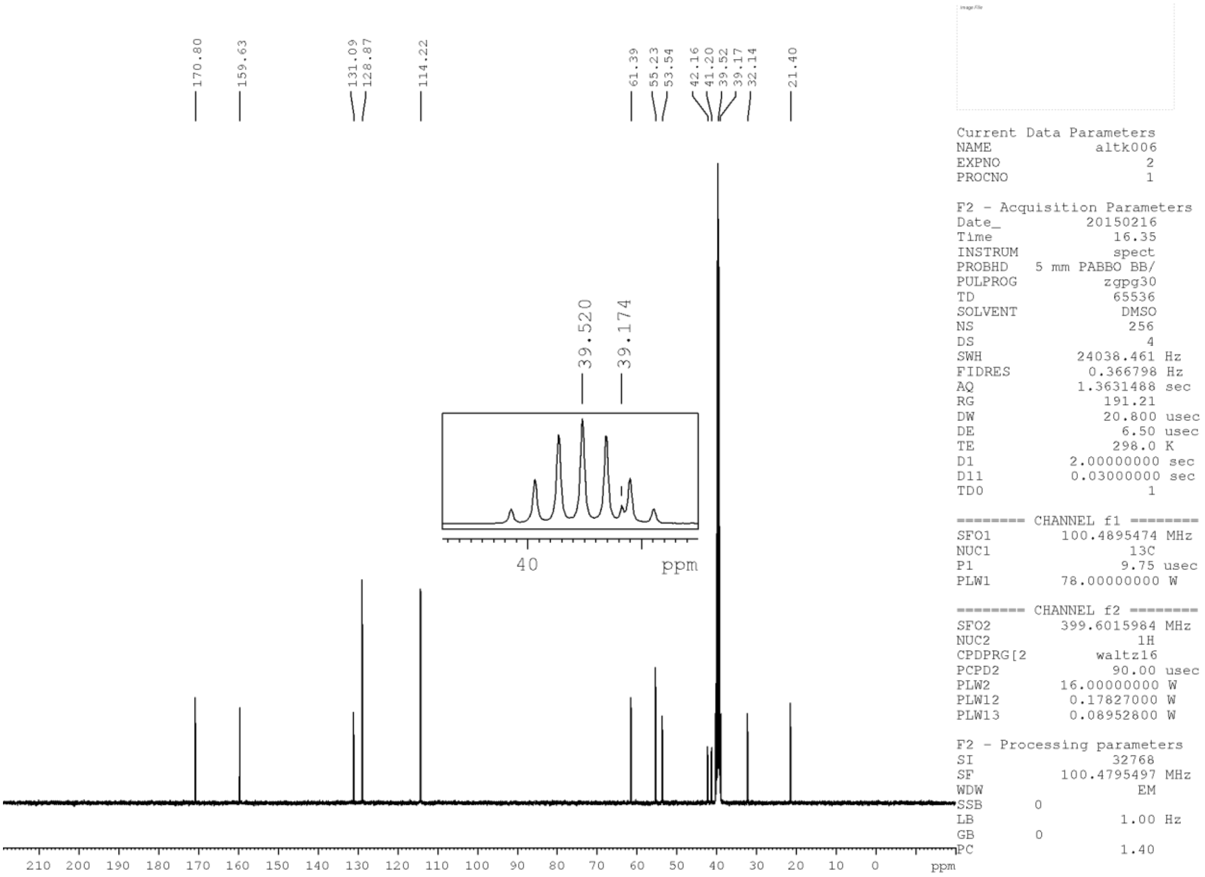
170.80
159.63
131.09
128.87
114.22
61.39
55.23
53.54
42.16
41.20
39.52
39.17
32.14
21.40
39.520
39.174
40
ppm
Current Data Parameters
NAME altk006
EXPNO 2
PROCNO 1
F2 - Acquisition Parameters
Date_ 20150216
Time 16.35
INSTRUM spect
PROBHD 5 mm PABBO BB/
PULPROG zgpg30
TD 65536
SOLVENT DMSO
NS 256
DS 4
SWH 24038.461 Hz
FIDRES 0.366798 Hz
AQ 1.3631488 sec
RG 191.21
DW 20.800 usec
DE 6.50 usec
TE 298.0 K
D1 2.00000000 sec
D11 0.03000000 sec
TD0 1
======== CHANNEL f1 ========
SFO1 100.4895474 MHz
NUC1 13C
P1 9.75 usec
PLW1 78.00000000 W
======== CHANNEL f2 ========
SFO2 399.6015984 MHz
NUC2 1H
CPDPRG[2 waltz16
PCPD2 90.00 usec
PLW2 16.00000000 W
PLW12 0.17827000 W
PLW13 0.08952800 W
F2 - Processing parameters
SI 32768
SF 100.4795497 MHz
WDW EM
SSB 0
LB 1.00 Hz
GB 0
PC 1.40
210 200 190 180 170 160 150 140 130 120 110 100 90 80 70 60 50 40 30 20 10 0 ppm

22

AVA-280-1 full charac 27052020.1.fid

Parameter	Value
Data File Name	C:/ Users/ anvi0182/ Desktop/ NMR/ AVA-280-1 full charac 27052020/ 1/ fid
Title	AVA-280-1 full charac 27052020.1.fid
Comment	
Origin	Bruker BioSpin GmbH
Owner	nmr
Site	
Instrument	spect
Author	
Solvent	MeOD
Temperature	298.0
Pulse Sequence	zg30
Experiment	1D
Probe	Z116098_0046 (PA BBO 400S1 BBF-H-D-05 Z SP)
Number of Scans	16
Receiver Gain	69.1
Relaxation Delay	1.0000
Pulse Width	9.8000
Presaturation Frequency	
Acquisition Time	4.1069
Acquisition Date	2020-05-26T11:13:02
Modification Date	2020-05-26T11:13:02
Class	
Spectrometer Frequency	399.60
Spectral Width	7978.7
Lowest Frequency	-1529.3
Nucleus	1H
Acquired Size	32768
Spectral Size	65536

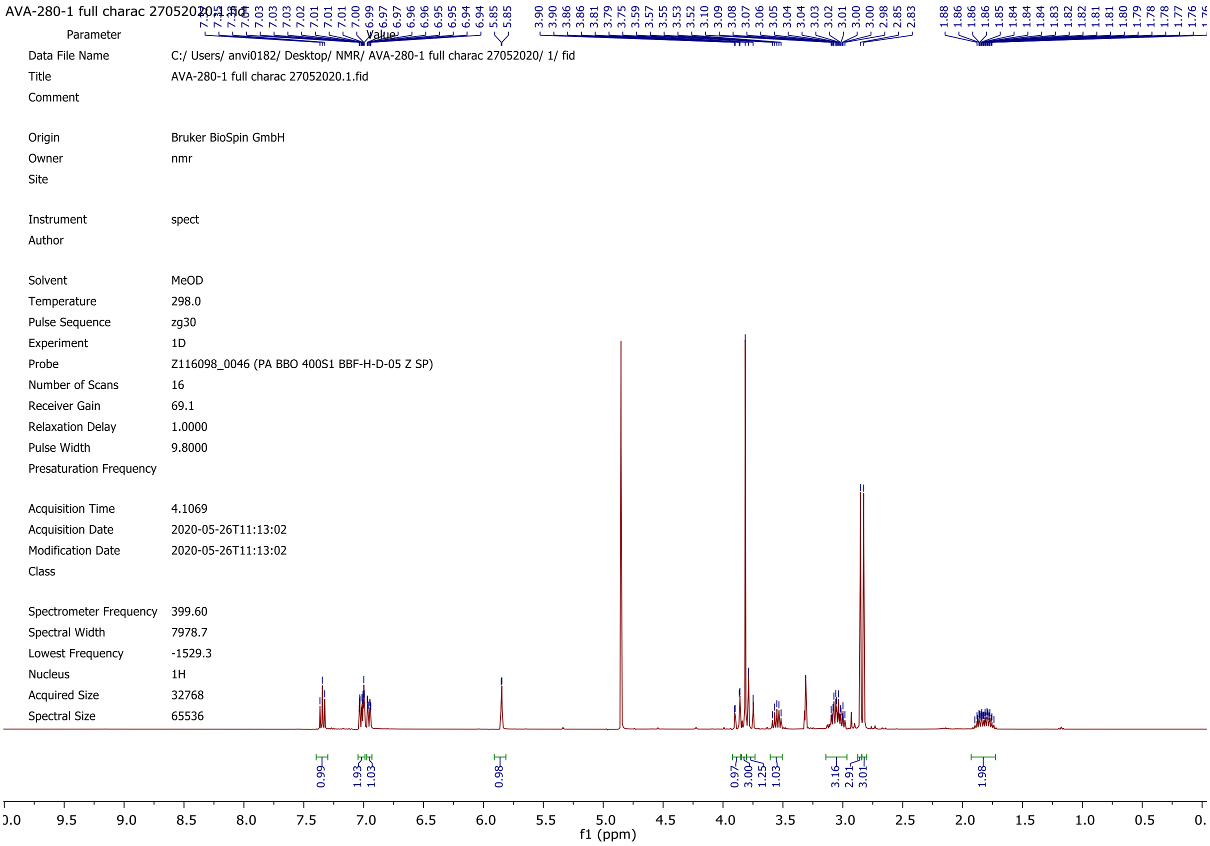

AVA-280-1 full charac 27052020.2.fid

Parameter	Value
Data File Name	C:/ Users/ anvi0182/ Desktop/ NMR/ AVA-280-1 full charac 27052020/ 2/ fid
Title	AVA-280-1 full charac 27052020.2.fid
Comment	
Origin	Bruker BioSpin GmbH
Owner	nmr
Site	
Instrument	spect
Author	
Solvent	MeOD
Temperature	298.0
Pulse Sequence	zgpg30
Experiment	1D
Probe	Z116098_0046 (PA BBO 400S1 BBF-H-D-05 Z SP)
Number of Scans	1024
Receiver Gain	191.2
Relaxation Delay	2.0000
Pulse Width	10.0000
Presaturation Frequency	
Acquisition Time	1.3631
Acquisition Date	2020-05-26T22:41:33
Modification Date	2020-05-26T22:41:33
Class	
Spectrometer Frequency	100.49
Spectral Width	24038.5
Lowest Frequency	-1833.5
Nucleus	13C
Acquired Size	32768
Spectral Size	65536

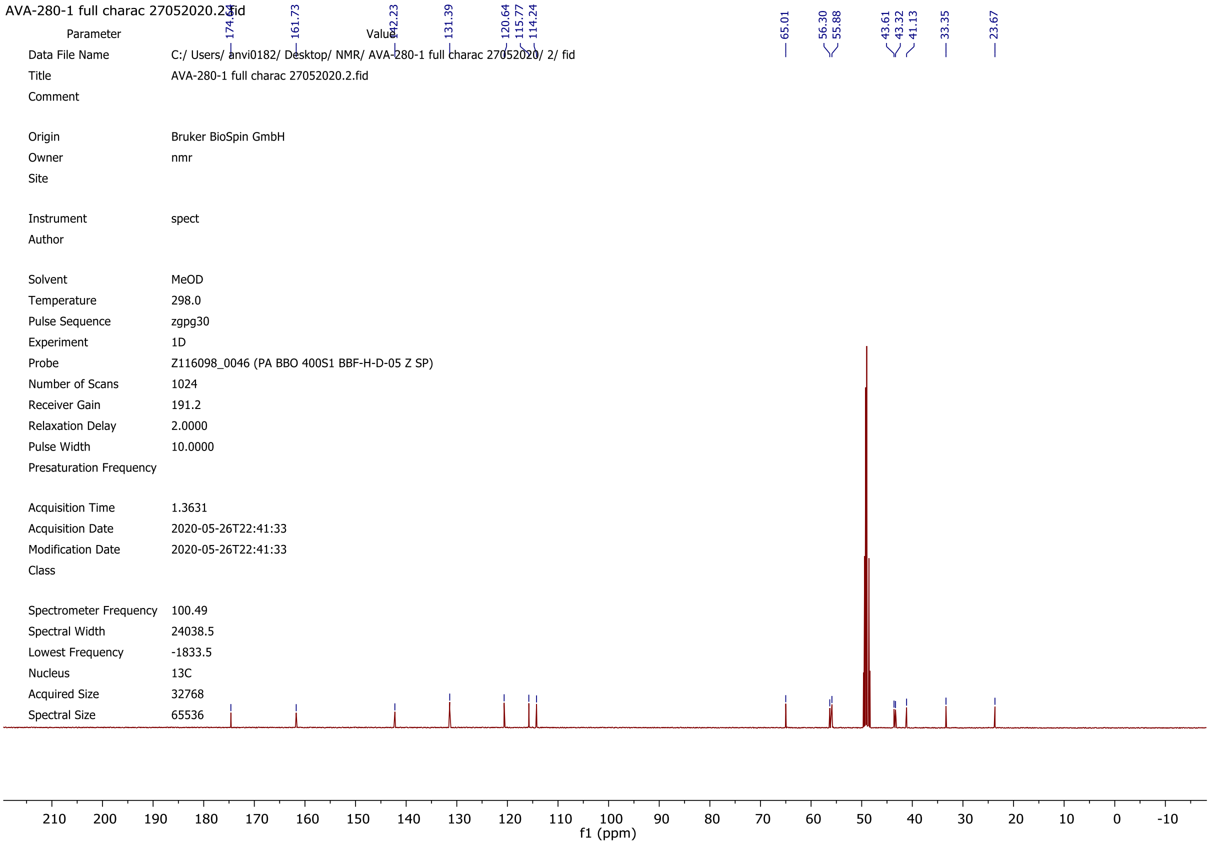

23

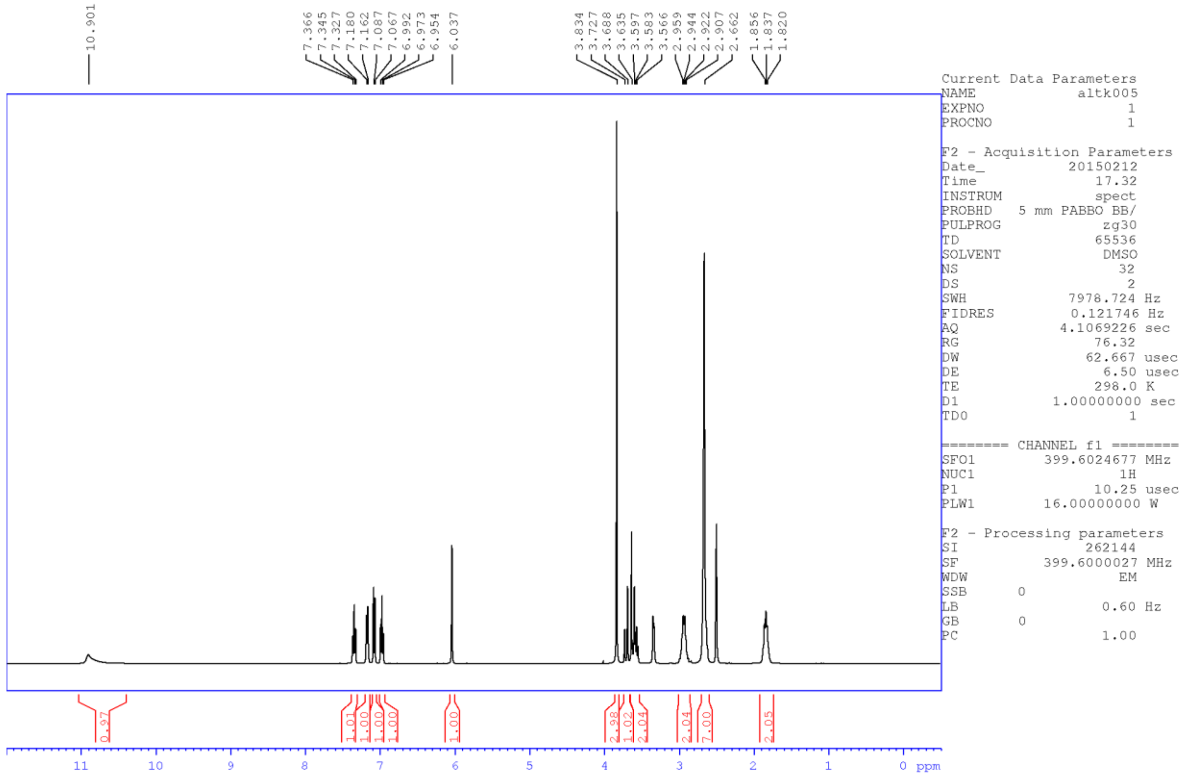
10.901
7.366
7.345
7.327
7.180
7.162
7.087
7.067
6.992
6.973
6.954
6.037
3.834
3.727
3.688
3.635
3.597
3.583
3.566
2.959
2.944
2.922
2.907
2.662
1.856
1.837
1.820
Current Data Parameters
NAME altk005
EXPNO 1
PROCNO 1
F2 - Acquisition Parameters
Date_ 20150212
Time 17.32
INSTRUM spect
PROBHD 5 mm PABBO BB/
PULPROG zg30
TD 65536
SOLVENT DMSO
NS 32
DS 2
SWH 7978.724 Hz
FIDRES 0.121746 Hz
AQ 4.1069226 sec
RG 76.32
DW 62.667 usec
DE 6.50 usec
TE 298.0 K
D1 1.00000000 sec
TD0 1
======== CHANNEL f1 ========
SFO1 399.6024677 MHz
NUC1 1H
P1 10.25 usec
PLW1 16.00000000 W
F2 - Processing parameters
SI 262144
SF 399.6000027 MHz
WDW EM
SSB 0
LB 0.60 Hz
GB 0
PC 1.00
0.97
1.01
1.00
1.00
1.00
1.00
2.98
1.02
2.04
2.04
7.00
2.05
11
10
9
8
7
6
5
4
3
2
1
0 ppm

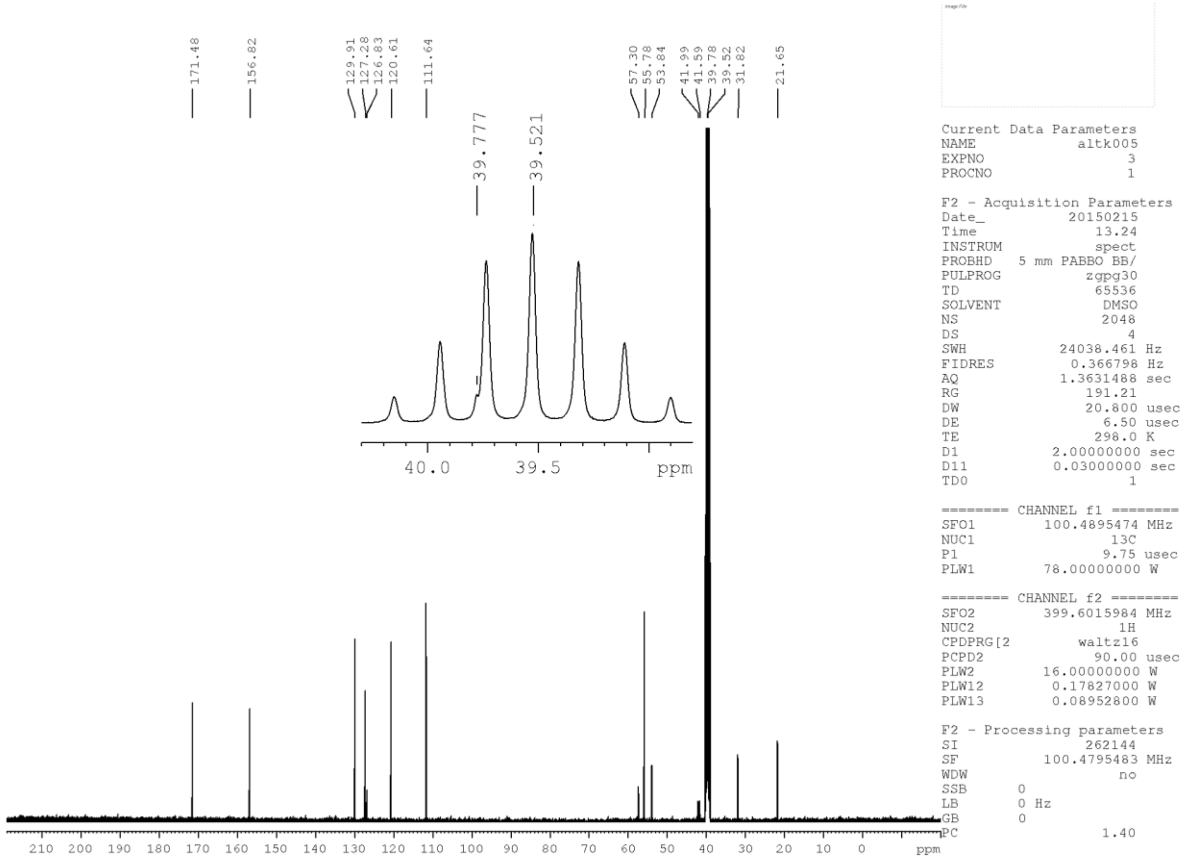
171.48
156.82
129.91
127.28
126.83
120.61
111.64
57.30
55.78
53.84
41.99
41.59
39.78
39.52
31.82
21.65
39.777
39.521
40.0
39.5
ppm
Current Data Parameters
NAME altk005
EXPNO 3
PROCNO 1
F2 - Acquisition Parameters
Date_ 20150215
Time 13.24
INSTRUM spect
PROBHD 5 mm PABBO BB/
PULPROG zgpg30
TD 65536
SOLVENT DMSO
NS 2048
DS 4
SWH 24038.461 Hz
FIDRES 0.366798 Hz
AQ 1.3631488 sec
RG 191.21
DW 20.800 usec
DE 6.50 usec
TE 298.0 K
D1 2.00000000 sec
D11 0.03000000 sec
TD0 1
======== CHANNEL f1 ========
SFO1 100.4895474 MHz
NUC1 13C
P1 9.75 usec
PLW1 78.00000000 W
======== CHANNEL f2 ========
SFO2 399.6015984 MHz
NUC2 1H
CPDPRG[2 waltz16
PCPD2 90.00 usec
PLW2 16.00000000 W
PLW12 0.17827000 W
PLW13 0.08952800 W
F2 - Processing parameters
SI 262144
SF 100.4795483 MHz
WDW no
SSB 0
LB 0 Hz
GB 0
PC 1.40
210 200 190 180 170 160 150 140 130 120 110 100 90 80 70 60 50 40 30 20 10 0 ppm

24

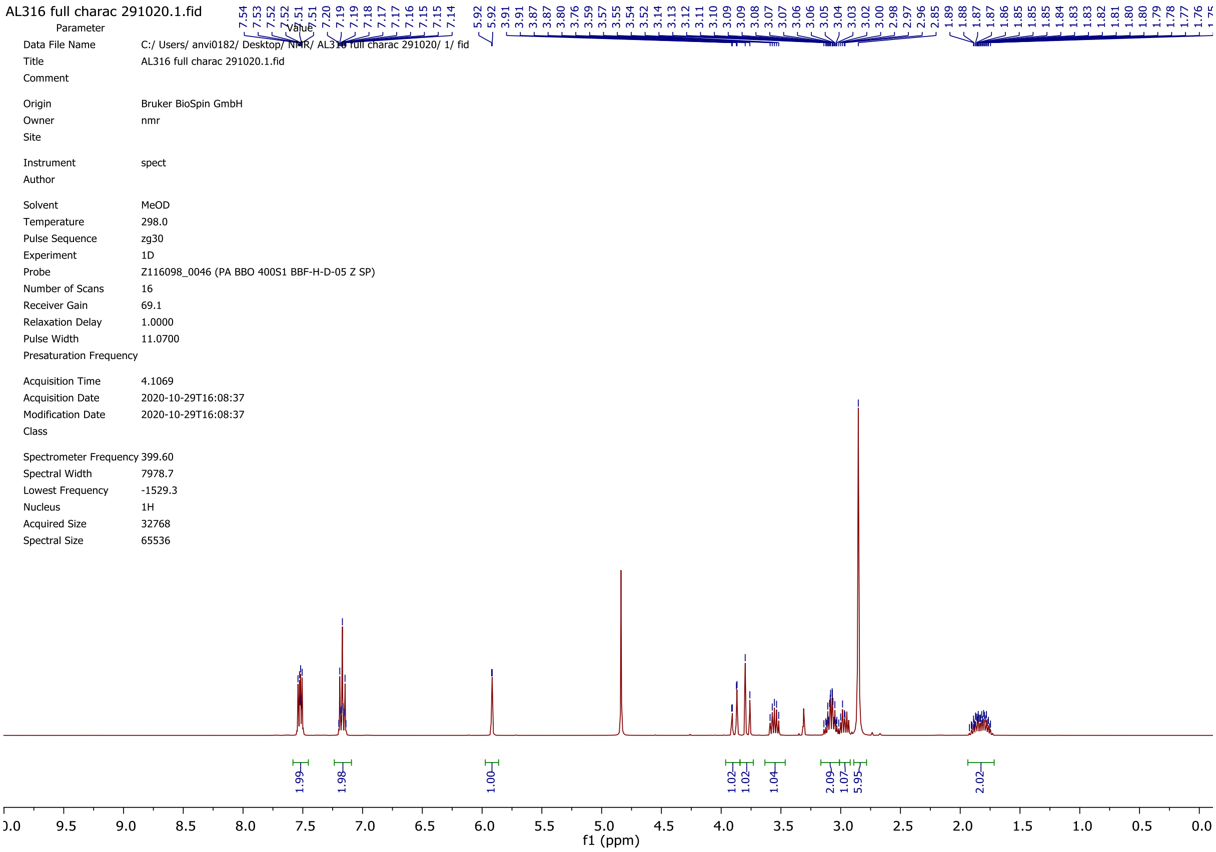
AL316 full charac 291020.1.fid
7.54 7.53 7.52 7.52 7.51 7.51 7.20 7.19 7.19 7.18 7.17 7.17 7.16 7.15 7.15 7.14 5.92 5.92 3.91 3.87 3.80 3.76 3.59 3.57 3.55 3.54 3.52 3.14 3.13 3.12 3.11 3.10 3.09 3.09 3.08 3.07 3.07 3.06 3.06 3.05 3.04 3.03 3.02 3.00 2.98 2.97 2.96 2.85 1.89 1.88 1.87 1.87 1.86 1.85 1.85 1.85 1.84 1.83 1.83 1.82 1.81 1.80 1.80 1.79 1.78 1.77 1.76
Parameter Value
Data File Name C:/ Users/ anvi0182/ Desktop/ NMR/ AL316 full charac 291020/ 1/ fid
Title AL316 full charac 291020.1.fid
Comment
Origin Bruker BioSpin GmbH
Owner nmr
Site
Instrument spect
Author
Solvent MeOD
Temperature 298.0
Pulse Sequence zg30
Experiment 1D
Probe Z116098_0046 (PA BBO 400S1 BBF-H-D-05 Z SP)
Number of Scans 16
Receiver Gain 69.1
Relaxation Delay 1.0000
Pulse Width 11.0700
Presaturation Frequency
Acquisition Time 4.1069
Acquisition Date 2020-10-29T16:08:37
Modification Date 2020-10-29T16:08:37
Class
Spectrometer Frequency 399.60
Spectral Width 7978.7
Lowest Frequency -1529.3
Nucleus 1H
Acquired Size 32768
Spectral Size 65536
1.99 1.98 1.00 1.02 1.02 1.04 2.09 1.07 5.95 2.02
10.0 9.5 9.0 8.5 8.0 7.5 7.0 6.5 6.0 5.5 5.0 4.5 4.0 3.5 3.0 2.5 2.0 1.5 1.0 0.5 0.0
f1 (ppm)

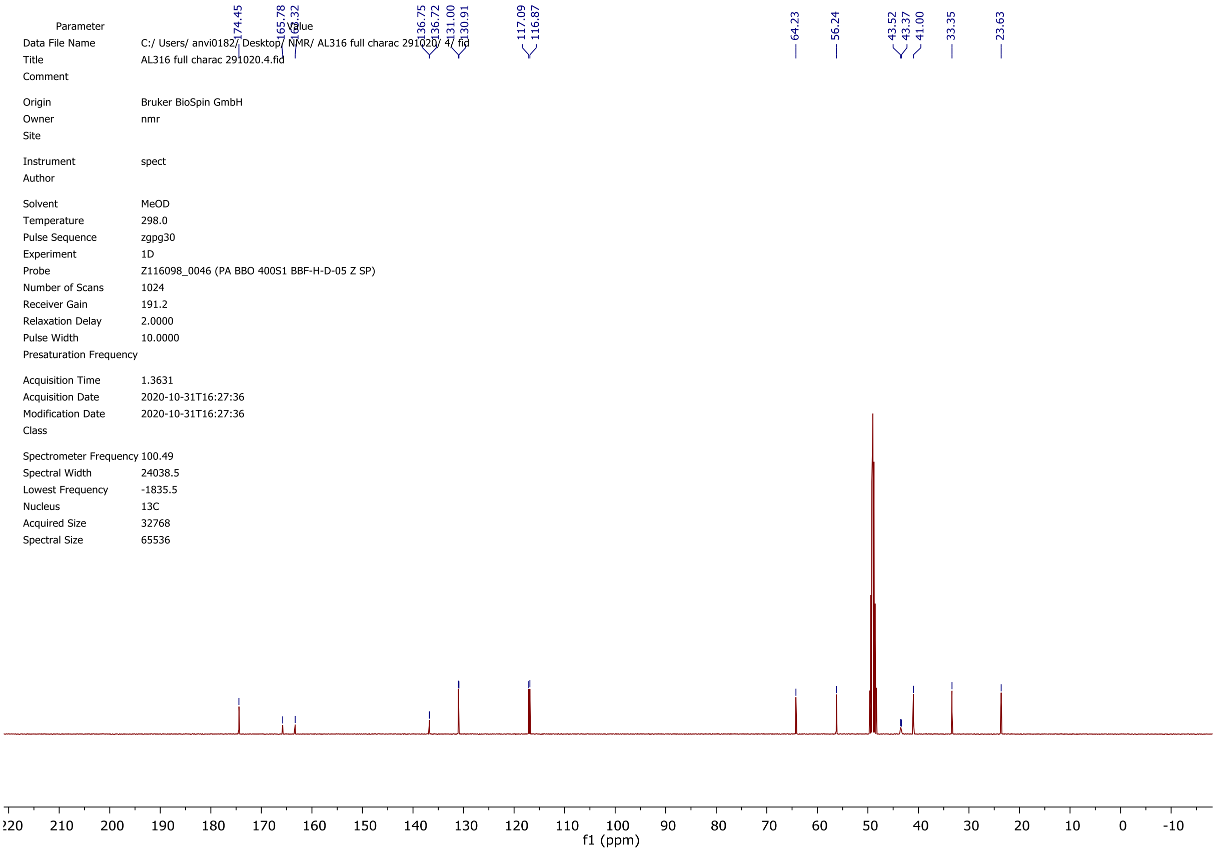
174.45 165.78 163.32 136.75 136.72 131.00 130.91 117.09 116.87 64.23 56.24 43.52 43.37 41.00 33.35 23.63
Parameter Value
Data File Name C:/ Users/ anvi0182/ Desktop/ NMR/ AL316 full charac 291020/ 4/ fid
Title AL316 full charac 291020.4.fid
Comment
Origin Bruker BioSpin GmbH
Owner nmr
Site
Instrument spect
Author
Solvent MeOD
Temperature 298.0
Pulse Sequence zgpg30
Experiment 1D
Probe Z116098_0046 (PA BBO 400S1 BBF-H-D-05 Z SP)
Number of Scans 1024
Receiver Gain 191.2
Relaxation Delay 2.0000
Pulse Width 10.0000
Presaturation Frequency
Acquisition Time 1.3631
Acquisition Date 2020-10-31T16:27:36
Modification Date 2020-10-31T16:27:36
Class
Spectrometer Frequency 100.49
Spectral Width 24038.5
Lowest Frequency -1835.5
Nucleus 13C
Acquired Size 32768
Spectral Size 65536
220 210 200 190 180 170 160 150 140 130 120 110 100 90 80 70 60 50 40 30 20 10 0 -10
f1 (ppm)

AL316 full charac 291020.2.fid

Parameter	Value
Data File Name	C:/ Users/ anvi0182/ Desktop/ NMR/ AL316 full charac 291020/ 2/ fid
Title	AL316 full charac 291020.2.fid
Comment	
Origin	Bruker BioSpin GmbH
Owner	nmr
Site	
Instrument	spect
Author	
Solvent	MeOD
Temperature	298.0
Pulse Sequence	zgfhigqn.2
Experiment	1D
Probe	Z116098_0046 (PA BBO 400S1 BBF-H-D-05 Z SP)
Number of Scans	16
Receiver Gain	191.2
Relaxation Delay	1.0000
Pulse Width	15.5000
Presaturation Frequency	
Acquisition Time	0.7340
Acquisition Date	2020-10-29T16:10:53
Modification Date	2020-10-29T16:10:53
Class	
Spectrometer Frequency	375.96
Spectral Width	89285.7
Lowest Frequency	-82242.9
Nucleus	19F
Acquired Size	65536
Spectral Size	131072

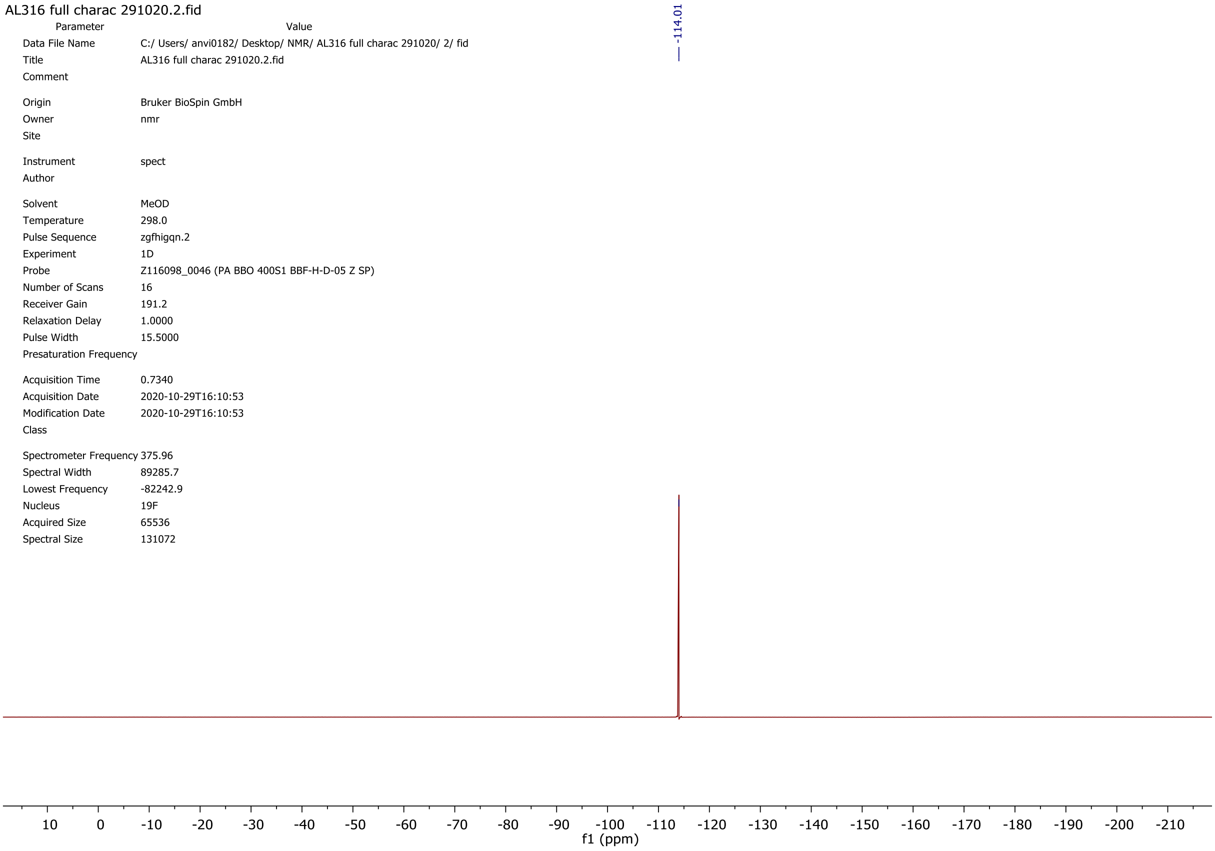

25

AVA-110-1 200619 DMSO full charac.1.fid

Parameter	Value
Data File Name	C:/ Users/ anvi0182/ Desktop/ NMR/ AVA-110-1 200619 DMSO full charac/ 1/ fid
Title	AVA-110-1 200619 DMSO full charac.1.fid
Comment	
Origin	Bruker BioSpin GmbH
Owner	nmr
Site	
Instrument	spect
Author	
Solvent	DMSO
Temperature	298.0
Pulse Sequence	zg30
Experiment	1D
Probe	Z132572_0007 (CP BBO 600S3 BB-H&F-D-05 Z)
Number of Scans	16
Receiver Gain	83.5
Relaxation Delay	1.0000
Pulse Width	11.4300
Presaturation Frequency	
Acquisition Time	2.7263
Acquisition Date	2019-06-20T14:22:40
Modification Date	2019-06-20T14:22:40
Class	
Spectrometer Frequency	600.17
Spectral Width	12019.2
Lowest Frequency	-2308.1
Nucleus	1H
Acquired Size	32768
Spectral Size	131072

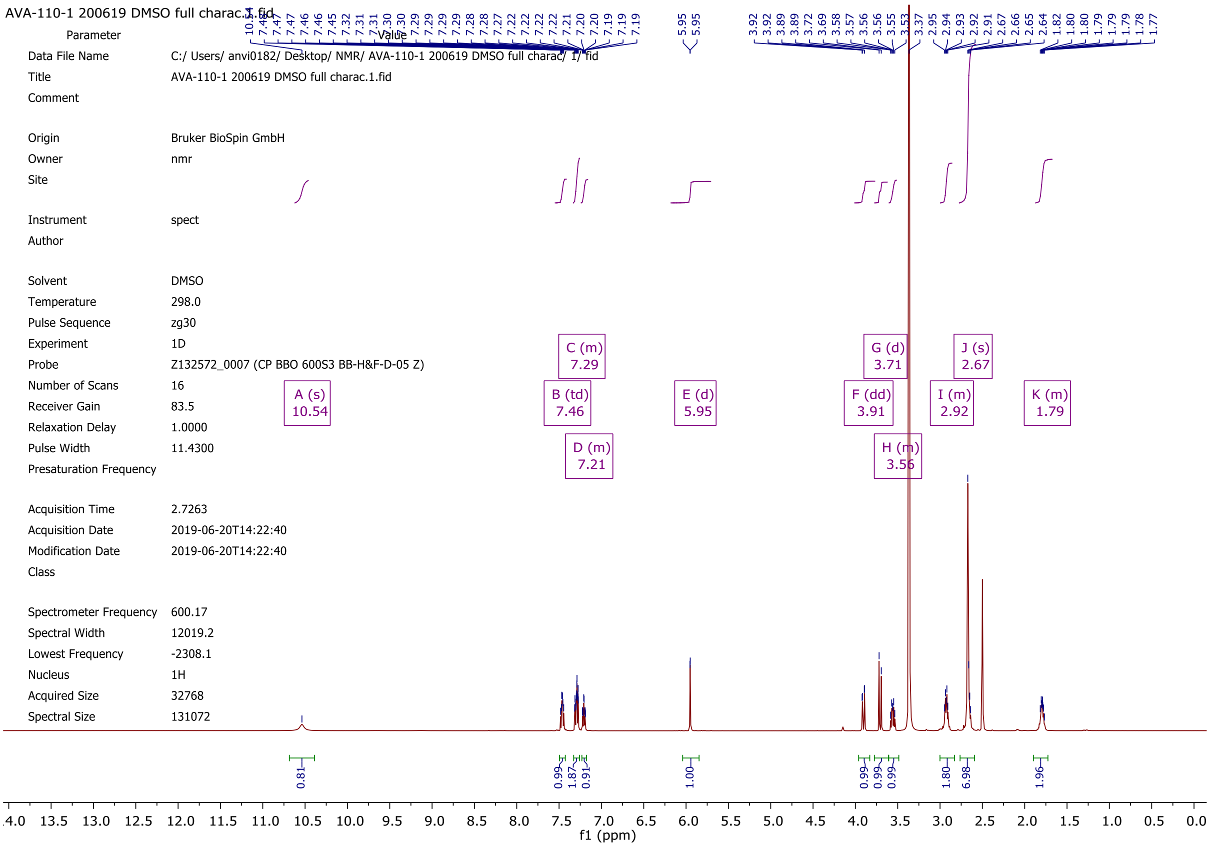

AVA-110-1 200619 DMSO full charac.2.fid

Parameter	Value
Data File Name	C:/ Users/ anvi0182/ Desktop/ NMR/ AVA-110-1 200619 DMSO full charac/ 2/ fid
Title	AVA-110-1 200619 DMSO full charac.2.fid
Comment	
Origin	Bruker BioSpin GmbH
Owner	nmr
Site	
Instrument	spect
Author	
Solvent	DMSO
Temperature	298.0
Pulse Sequence	zgpg30
Experiment	1D
Probe	Z132572_0007 (CP BBO 600S3 BB-H&F-D-05 Z)
Number of Scans	768
Receiver Gain	182.7
Relaxation Delay	2.0000
Pulse Width	9.7000
Presaturation Frequency	
Acquisition Time	0.9088
Acquisition Date	2019-06-21T07:24:41
Modification Date	2019-06-21T07:24:41
Class	
Spectrometer Frequency	150.93
Spectral Width	36057.7
Lowest Frequency	-3005.4
Nucleus	13C
Acquired Size	32768
Spectral Size	65536

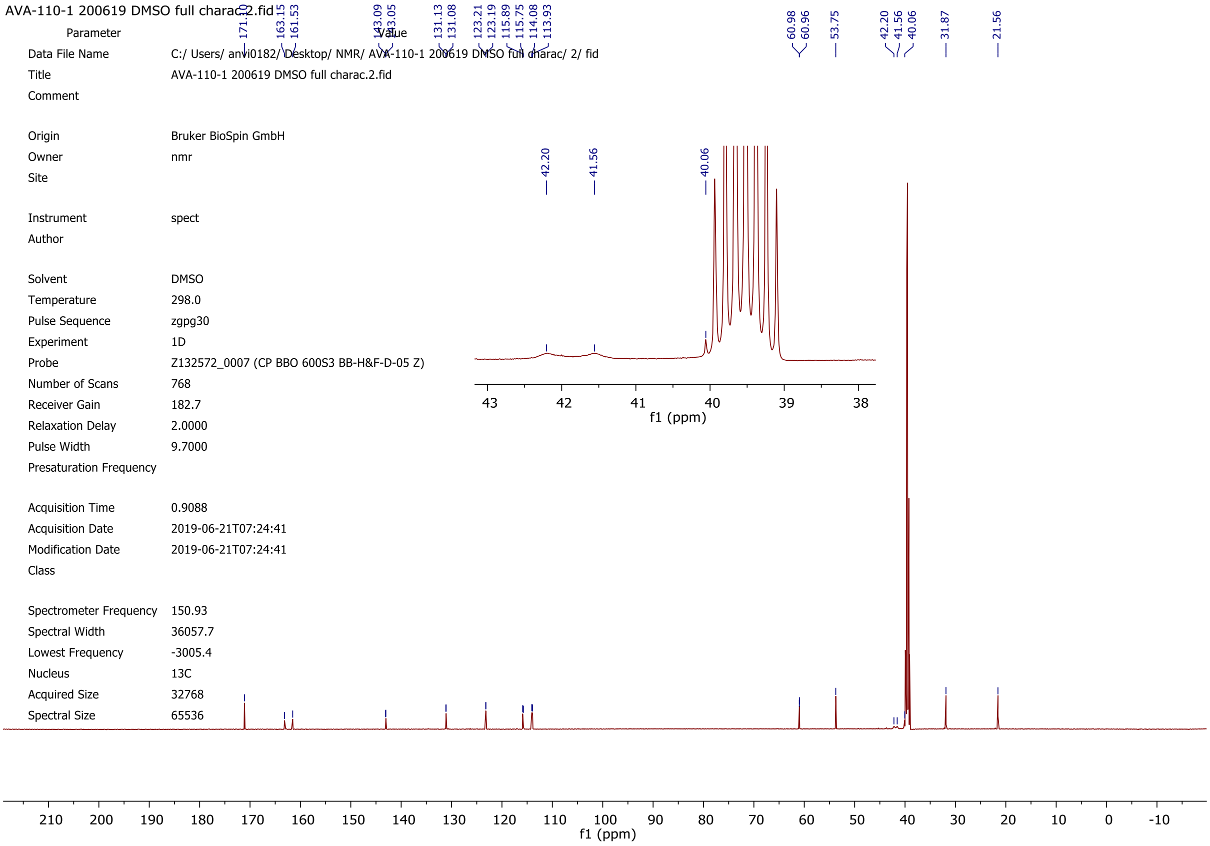

AL471 19F and 13C.1.fid

Parameter	Value
Data File Name	C:/ Users/ anvi0182/ Desktop/ NMR/ AL471 19F and 13C/ 1/ fid
Title	AL471 19F and 13C.1.fid
Comment	
Origin	Bruker BioSpin GmbH
Owner	nmr
Site	
Instrument	spect
Author	
Solvent	DMSO
Temperature	298.0
Pulse Sequence	zgfhigqn.2
Experiment	1D
Probe	Z116098_0046 (PA BBO 400S1 BBF-H-D-05 Z SP)
Number of Scans	16
Receiver Gain	191.2
Relaxation Delay	1.0000
Pulse Width	15.5000
Presaturation Frequency	
Acquisition Time	0.7340
Acquisition Date	2020-07-08T21:22:23
Modification Date	2020-07-08T21:22:23
Class	
Spectrometer Frequency	375.96
Spectral Width	89285.7
Lowest Frequency	-82242.9
Nucleus	19F
Acquired Size	65536
Spectral Size	131072

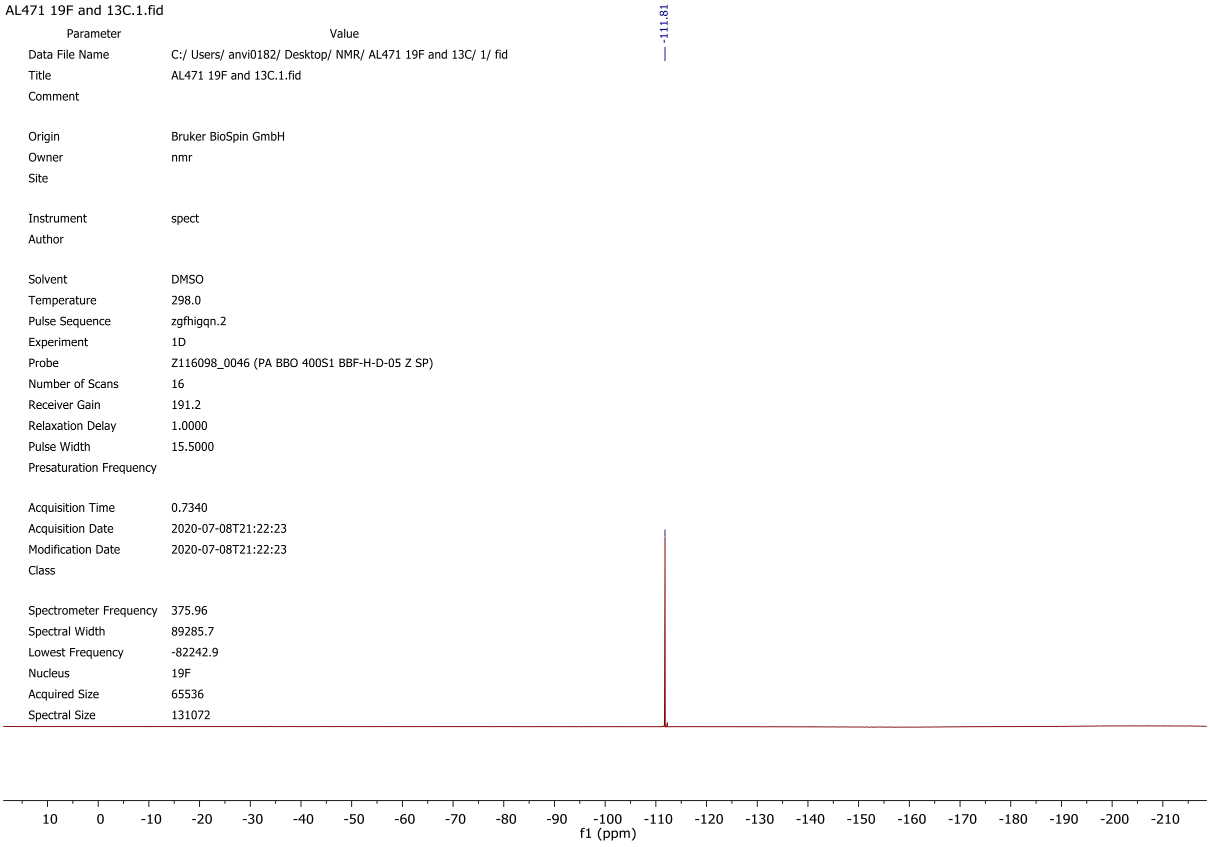

26

AVA-108-1 200619 MeOD full charac.1.fid

Parameter	Value
Data File Name	C:/ Users/ anvi0182/ Desktop/ NMR/ AVA-108-1 200619 MeOD full charac/ 1/ fid
Title	AVA-108-1 200619 MeOD full charac.1.fid
Origin	Bruker BioSpin GmbH
Owner	nmr
Instrument	spect
Solvent	MeOD
Temperature	298.0
Pulse Sequence	zg30
Experiment	1D
Probe	Z132572_0007 (CP BBO 600S3 BB-H&F-D-05 Z)
Number of Scans	16
Receiver Gain	91.6
Relaxation Delay	1.0000
Pulse Width	11.4300
Presaturation Frequency	
Acquisition Time	2.7263
Acquisition Date	2019-06-20T13:57:41
Modification Date	2019-06-20T13:57:41
Class	
Spectrometer Frequency	600.17
Spectral Width	12019.2
Lowest Frequency	-2314.1
Nucleus	1H
Acquired Size	32768
Spectral Size	131072

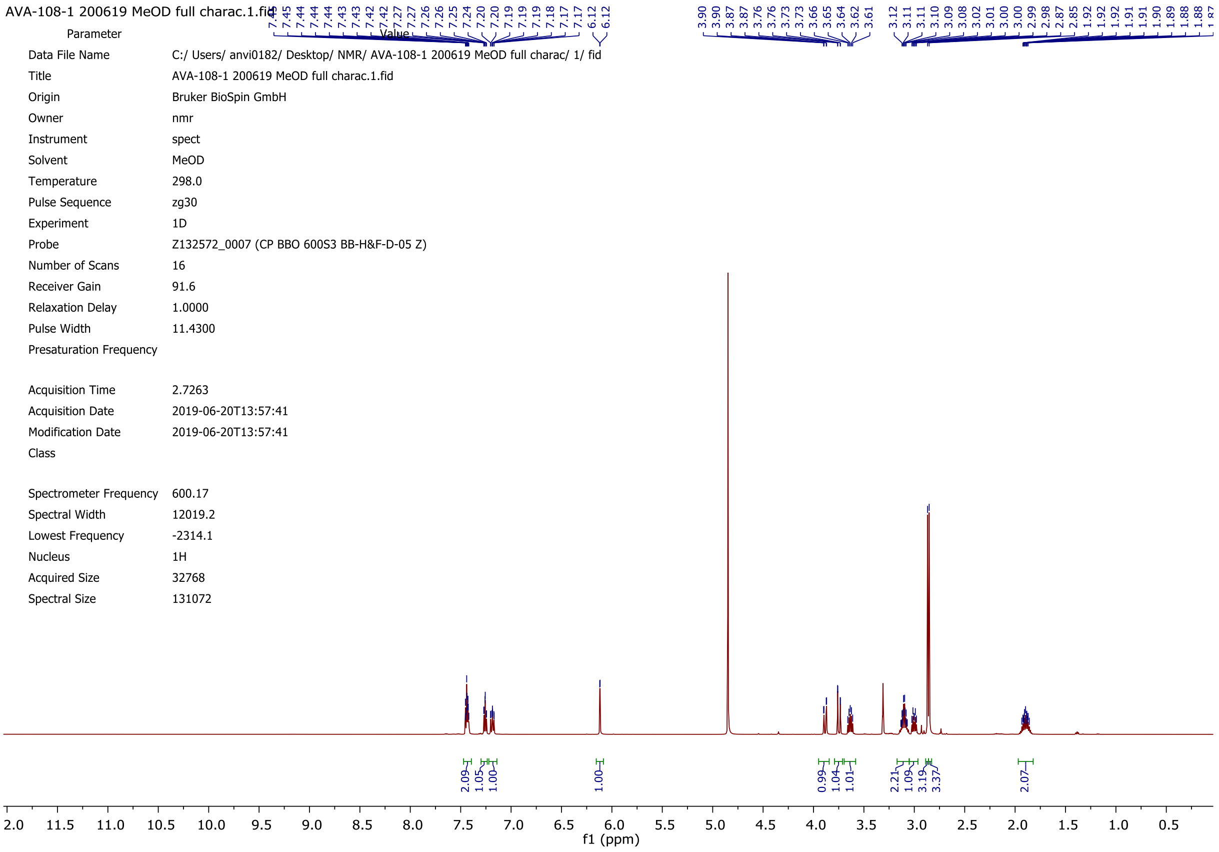

AVA-108-1 200619 MeOD full charac.2.fid

Parameter	Value
Data File Name	C:/ Users/ anvi0182/ Desktop/ NMR/ AVA-108-1 200619 MeOD full charac/ 2/ fid
Title	AVA-108-1 200619 MeOD full charac.2.fid
Origin	Bruker BioSpin GmbH
Owner	nmr
Instrument	spect
Solvent	MeOD
Temperature	298.0
Pulse Sequence	zgpg30
Experiment	1D
Probe	Z132572_0007 (CP BBO 600S3 BB-H&F-D-05 Z)
Number of Scans	768
Receiver Gain	182.7
Relaxation Delay	2.0000
Pulse Width	9.7000
Presaturation Frequency	
Acquisition Time	0.9088
Acquisition Date	2019-06-21T04:30:54
Modification Date	2019-06-21T04:30:54
Class	
Spectrometer Frequency	150.93
Spectral Width	36057.7
Lowest Frequency	-2729.1
Nucleus	13C
Acquired Size	32768
Spectral Size	65536

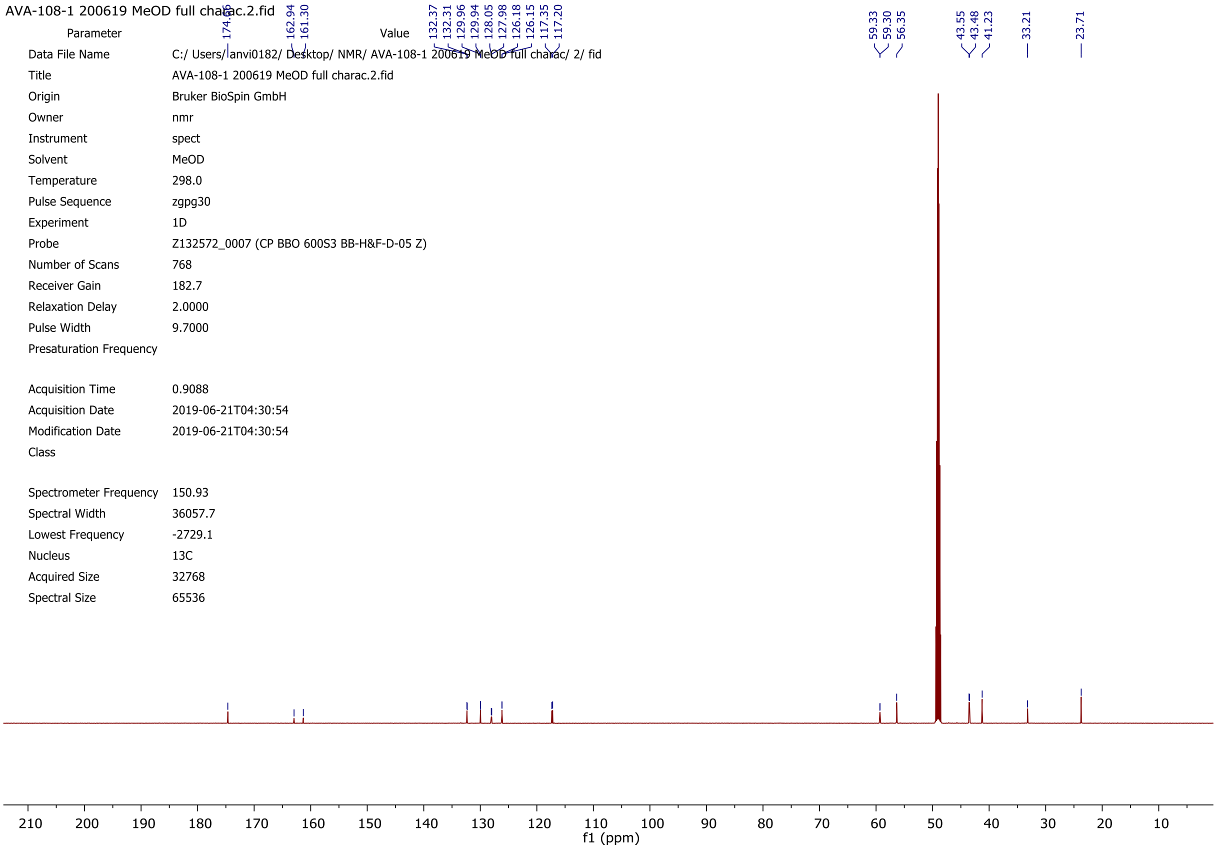

AL470 19F MeOD.1.fid

Parameter	Value
Data File Name	C:/ Users/ anvi0182/ Desktop/ NMR/ AL470 19F MeOD/ 1/ fid
Title	AL470 19F MeOD.1.fid
Origin	Bruker BioSpin GmbH
Owner	nmr
Instrument	spect
Solvent	MeOD
Temperature	298.0
Pulse Sequence	zgfhigqn.2
Experiment	1D
Probe	Z116098_0046 (PA BBO 400S1 BBF-H-D-05 Z SP)
Number of Scans	16
Receiver Gain	191.2
Relaxation Delay	1.0000
Pulse Width	15.5000
Presaturation Frequency	
Acquisition Time	0.7340
Acquisition Date	2020-07-13T16:40:18
Modification Date	2020-07-13T16:40:18
Class	
Spectrometer Frequency	375.96
Spectral Width	89285.7
Lowest Frequency	-82242.9
Nucleus	19F
Acquired Size	65536
Spectral Size	131072

-120.29

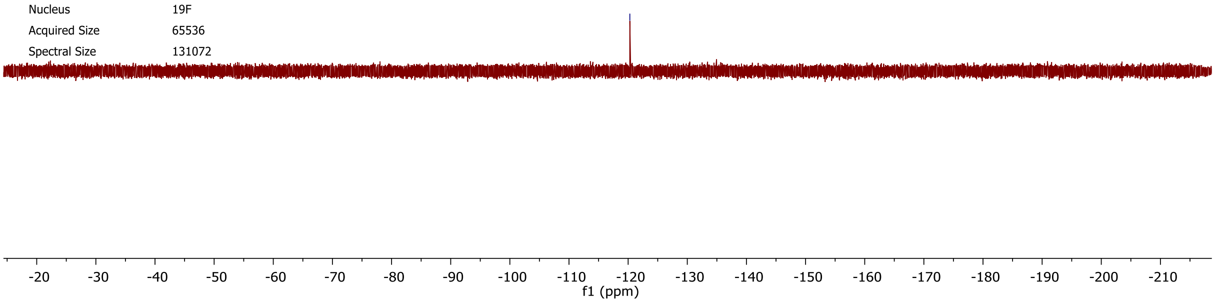

27

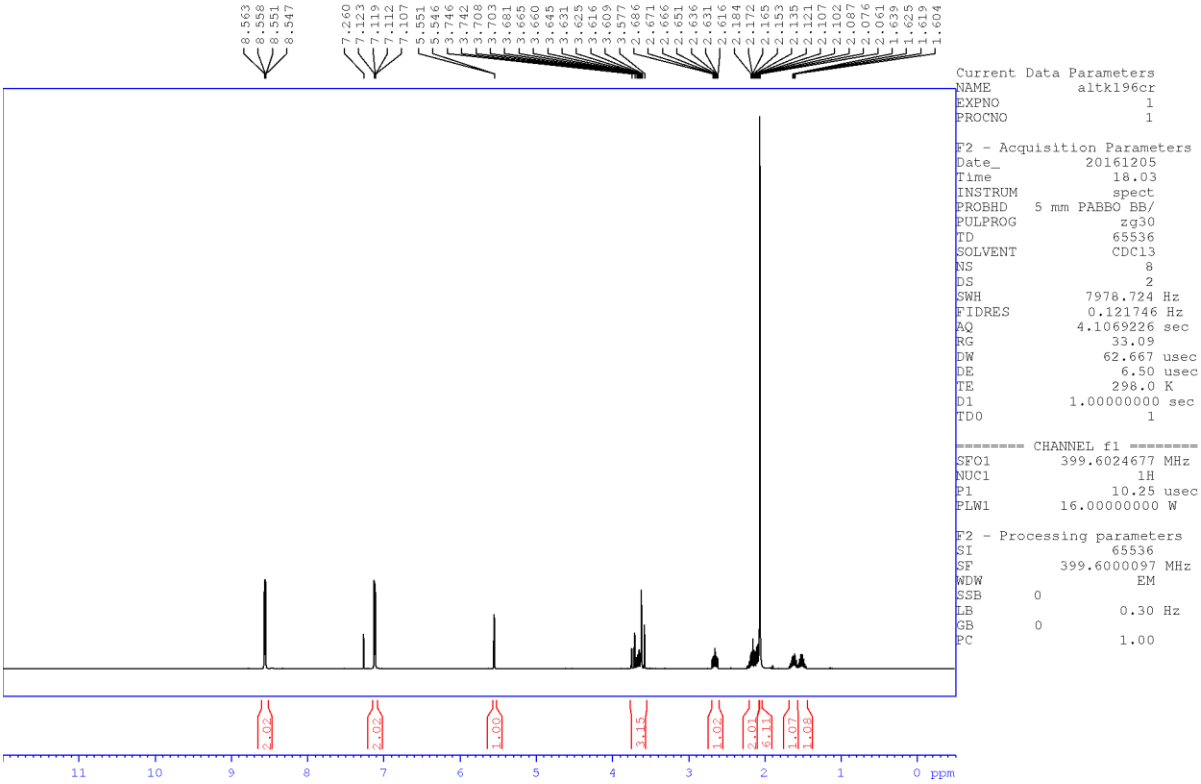
Current Data Parameters
NAME altk196cr
EXPNO 1
PROCNO 1
F2 - Acquisition Parameters
Date_ 20161205
Time 18.03
INSTRUM spect
PROBHD 5 mm PABBO BB/
PULPROG zg30
TD 65536
SOLVENT CDCl3
NS 8
DS 2
SWH 7978.724 Hz
FIDRES 0.121746 Hz
AQ 4.1069226 sec
RG 33.09
DW 62.667 usec
DE 6.50 usec
TE 298.0 K
D1 1.00000000 sec
TD0 1
======== CHANNEL f1 ========
SFO1 399.6024677 MHz
NUC1 1H
P1 10.25 usec
PLW1 16.00000000 W
F2 - Processing parameters
SI 65536
SF 399.6000097 MHz
WDW EM
SSB 0
LB 0.30 Hz
GB 0
PC 1.00

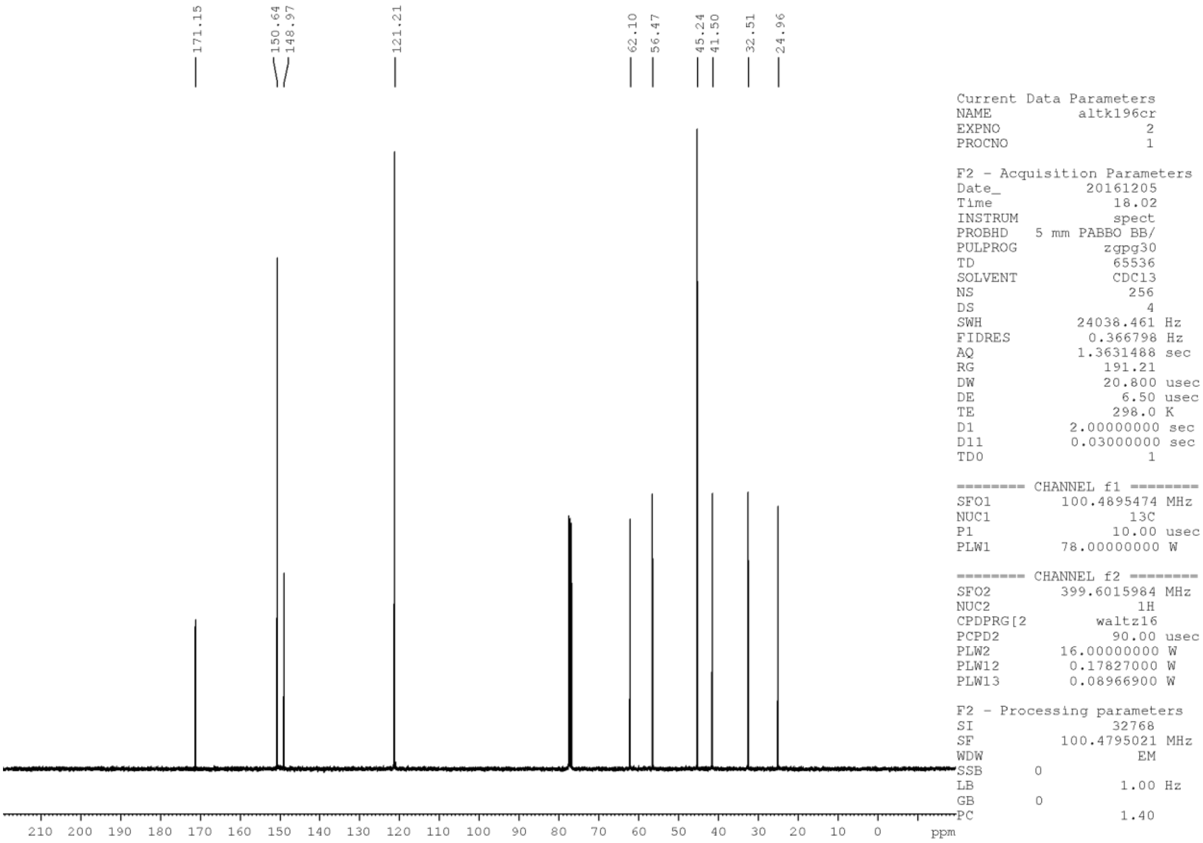
171.15
150.64
148.97
121.21
62.10
56.47
45.24
41.50
32.51
24.96
Current Data Parameters
NAME altk196cr
EXPNO 2
PROCNO 1
F2 - Acquisition Parameters
Date_ 20161205
Time 18.02
INSTRUM spect
PROBHD 5 mm PABBO BB/
PULPROG zgpg30
TD 65536
SOLVENT CDCl3
NS 256
DS 4
SWH 24038.461 Hz
FIDRES 0.366798 Hz
AQ 1.3631488 sec
RG 191.21
DW 20.800 usec
DE 6.50 usec
TE 298.0 K
D1 2.00000000 sec
D11 0.03000000 sec
TD0 1
======== CHANNEL f1 ========
SFO1 100.4895474 MHz
NUC1 13C
P1 10.00 usec
PLW1 78.00000000 W
======== CHANNEL f2 ========
SFO2 399.6015984 MHz
NUC2 1H
CPDPRG[2 waltz16
PCPD2 90.00 usec
PLW2 16.00000000 W
PLW12 0.17827000 W
PLW13 0.08966900 W
F2 - Processing parameters
SI 32768
SF 100.4795021 MHz
WDW EM
SSB 0
LB 1.00 Hz
GB 0
PC 1.40

28

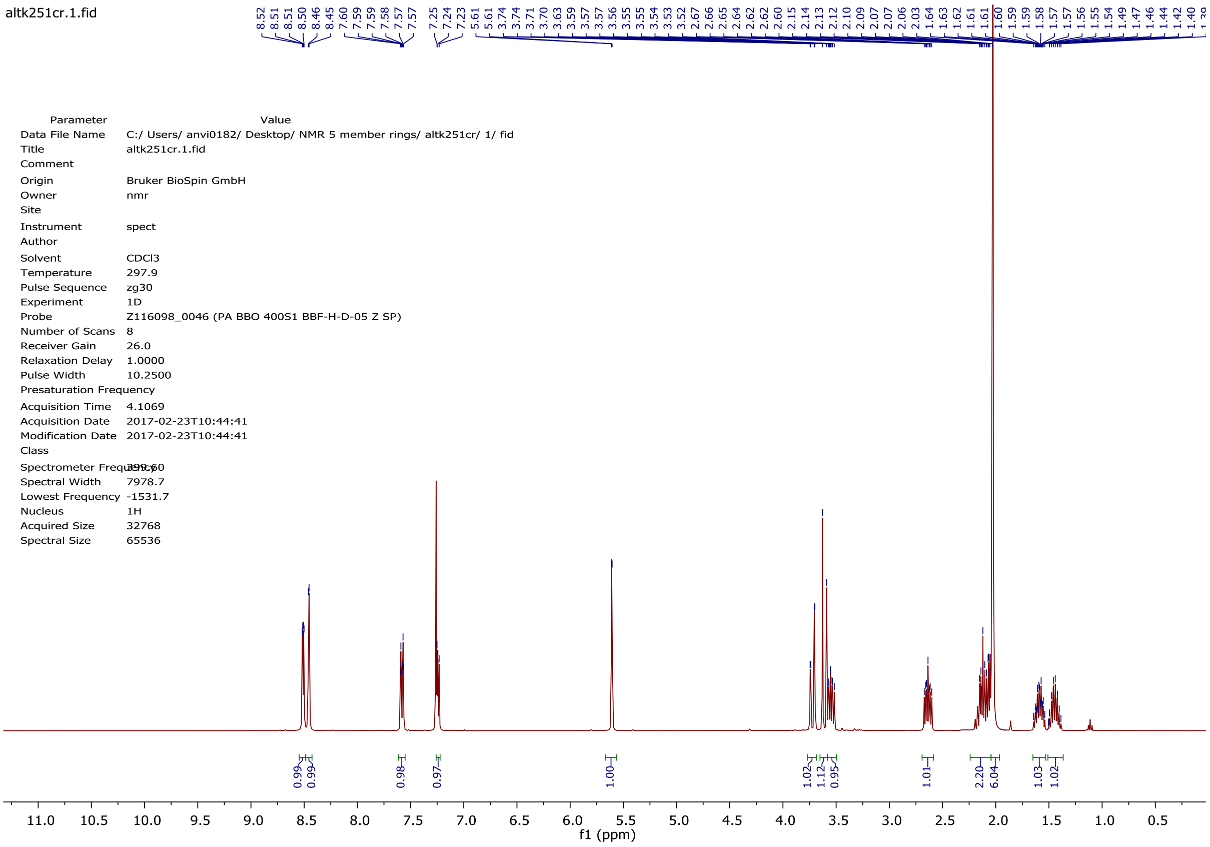

altk251cr.1.fid
8.52 8.51 8.51 8.50 8.46 8.45 7.60 7.59 7.59 7.58 7.57 7.57 7.25 7.24 7.23 5.61 5.61 3.74 3.74 3.71 3.70 3.63 3.59 3.57 3.57 3.56 3.55 3.55 3.54 3.53 3.52 2.67 2.66 2.65 2.64 2.62 2.62 2.60 2.15 2.14 2.13 2.12 2.10 2.09 2.07 2.07 2.06 2.03 1.64 1.63 1.62 1.61 1.61 1.60 1.59 1.59 1.58 1.57 1.57 1.56 1.55 1.54 1.49 1.47 1.46 1.44 1.42 1.40 [illegible]
Parameter Value
Data File Name C:/ Users/ anvi0182/ Desktop/ NMR 5 member rings/ altk251cr/ 1/ fid
Title altk251cr.1.fid
Comment
Origin Bruker BioSpin GmbH
Owner nmr
Site
Instrument spect
Author
Solvent CDCl3
Temperature 297.9
Pulse Sequence zg30
Experiment 1D
Probe Z116098_0046 (PA BBO 400S1 BBF-H-D-05 Z SP)
Number of Scans 8
Receiver Gain 26.0
Relaxation Delay 1.0000
Pulse Width 10.2500
Presaturation Frequency
Acquisition Time 4.1069
Acquisition Date 2017-02-23T10:44:41
Modification Date 2017-02-23T10:44:41
Class
Spectrometer Frequency 399.60
Spectral Width 7978.7
Lowest Frequency -1531.7
Nucleus 1H
Acquired Size 32768
Spectral Size 65536
0.99 0.99 0.98 0.97 1.00 1.02 1.12 0.95 1.01 2.20 6.04 1.03 1.02
11.0 10.5 10.0 9.5 9.0 8.5 8.0 7.5 7.0 6.5 6.0 5.5 5.0 4.5 4.0 3.5 3.0 2.5 2.0 1.5 1.0 0.5
f1 (ppm)

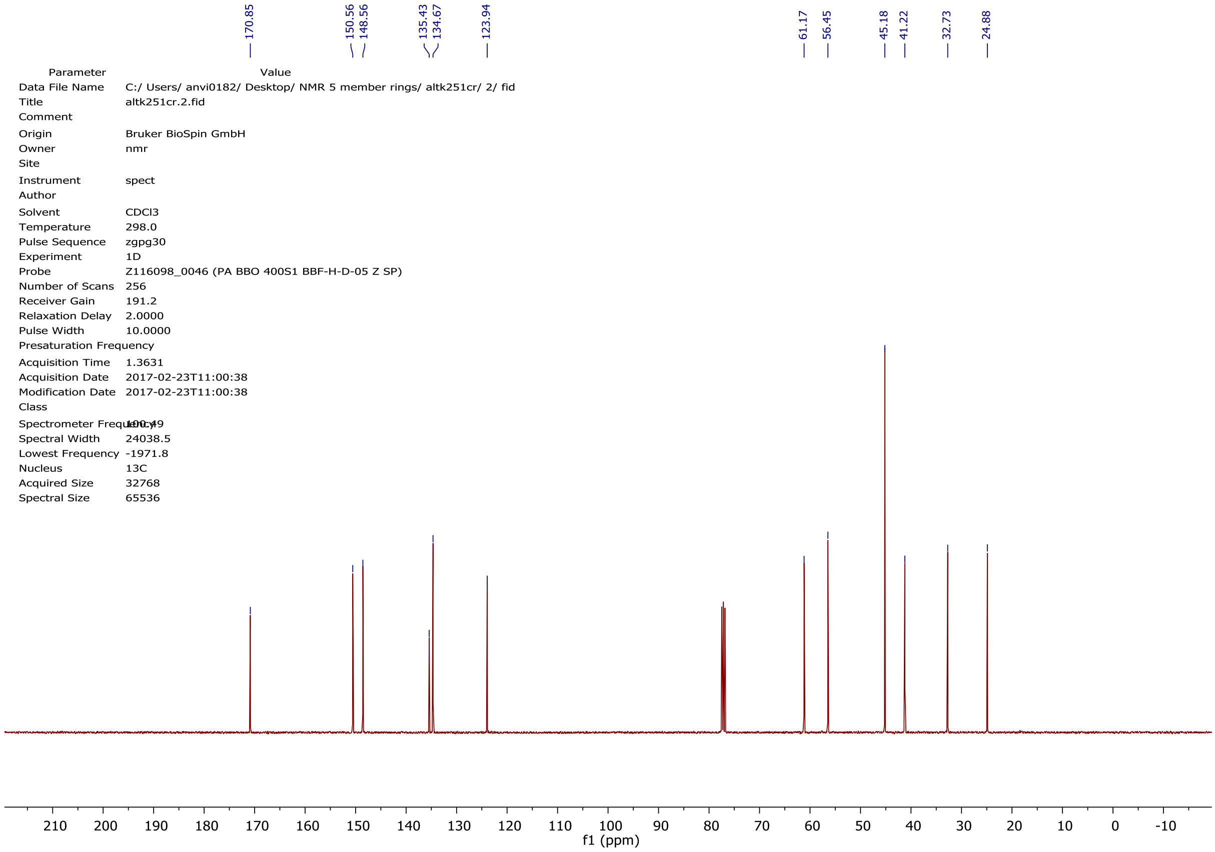

170.85 150.56 148.56 135.43 134.67 123.94 61.17 56.45 45.18 41.22 32.73 24.88
Parameter Value
Data File Name C:/ Users/ anvi0182/ Desktop/ NMR 5 member rings/ altk251cr/ 2/ fid
Title altk251cr.2.fid
Comment
Origin Bruker BioSpin GmbH
Owner nmr
Site
Instrument spect
Author
Solvent CDCl3
Temperature 298.0
Pulse Sequence zgpg30
Experiment 1D
Probe Z116098_0046 (PA BBO 400S1 BBF-H-D-05 Z SP)
Number of Scans 256
Receiver Gain 191.2
Relaxation Delay 2.0000
Pulse Width 10.0000
Presaturation Frequency
Acquisition Time 1.3631
Acquisition Date 2017-02-23T11:00:38
Modification Date 2017-02-23T11:00:38
Class
Spectrometer Frequency 100.49
Spectral Width 24038.5
Lowest Frequency -1971.8
Nucleus 13C
Acquired Size 32768
Spectral Size 65536
210 200 190 180 170 160 150 140 130 120 110 100 90 80 70 60 50 40 30 20 10 0 -10
f1 (ppm)

29

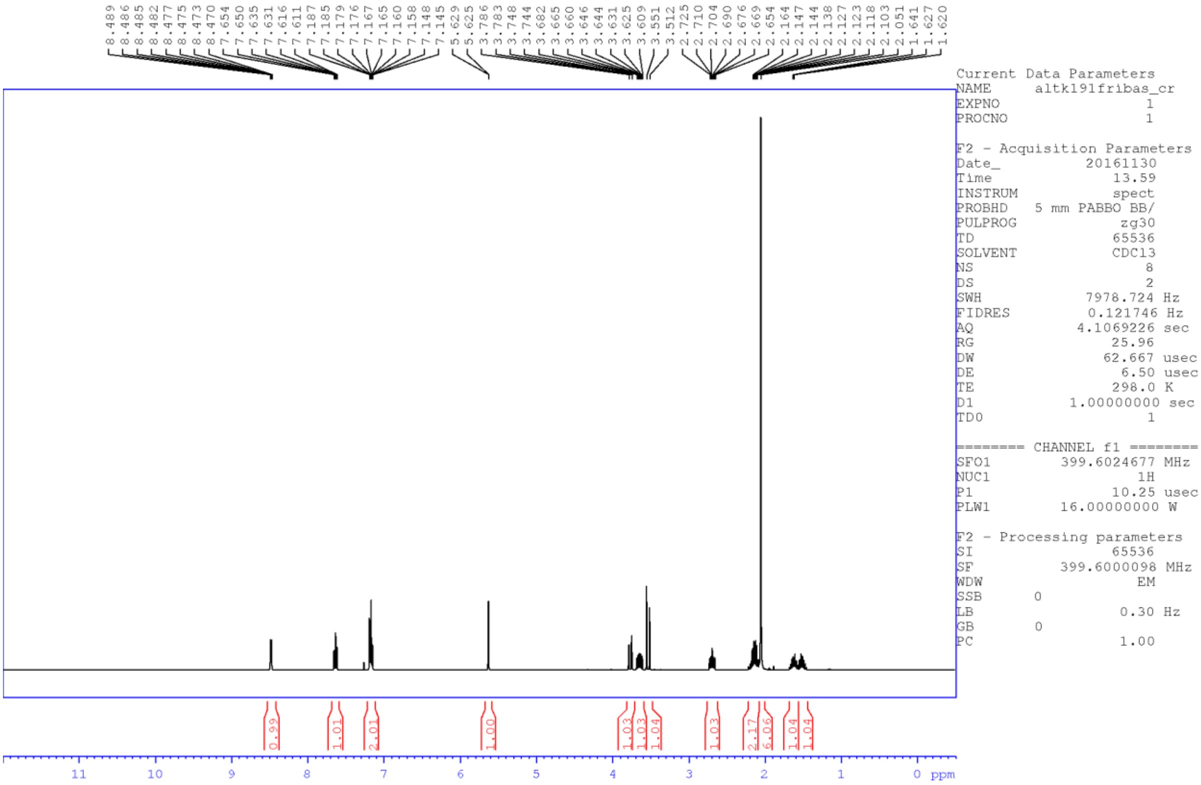

```
Current Data Parameters
NAME      altk191fribas_cr
EXPNO                    1
PROCNO                   1

F2 - Acquisition Parameters
Date_             20161130
Time                 13.59
INSTRUM              spect
PROBHD   5 mm PABBO BB/
PULPROG               zg30
TD                   65536
SOLVENT              CDCl3
NS                       8
DS                       2
SWH               7978.724 Hz
FIDRES            0.121746 Hz
AQ               4.1069226 sec
RG                   25.96
DW                  62.667 usec
DE                    6.50 usec
TE                   298.0 K
D1              1.00000000 sec
TD0                      1

======== CHANNEL f1 ========
SFO1           399.6024677 MHz
NUC1                    1H
P1                   10.25 usec
PLW1           16.00000000 W

F2 - Processing parameters
SI                   65536
SF             399.6000098 MHz
WDW                     EM
SSB      0
LB                    0.30 Hz
GB       0
PC                    1.00
```

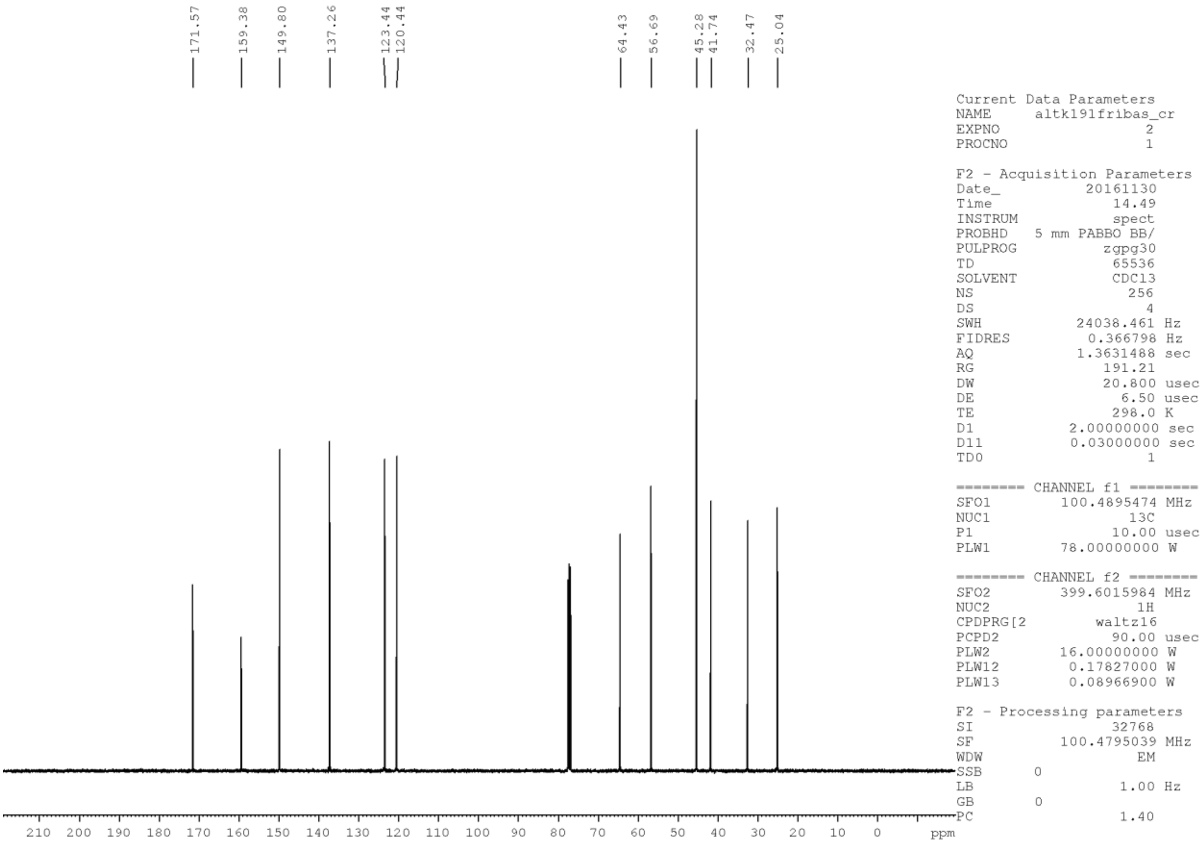

```
Current Data Parameters
NAME      altk191fribas_cr
EXPNO                    2
PROCNO                   1

F2 - Acquisition Parameters
Date_             20161130
Time                 14.49
INSTRUM              spect
PROBHD   5 mm PABBO BB/
PULPROG             zgpg30
TD                   65536
SOLVENT              CDCl3
NS                     256
DS                       4
SWH              24038.461 Hz
FIDRES            0.366798 Hz
AQ               1.3631488 sec
RG                  191.21
DW                  20.800 usec
DE                    6.50 usec
TE                   298.0 K
D1              2.00000000 sec
D11             0.03000000 sec
TD0                      1

======== CHANNEL f1 ========
SFO1           100.4895474 MHz
NUC1                   13C
P1                   10.00 usec
PLW1           78.00000000 W

======== CHANNEL f2 ========
SFO2           399.6015984 MHz
NUC2                    1H
CPDPRG[2           waltz16
PCPD2                90.00 usec
PLW2           16.00000000 W
PLW12           0.17827000 W
PLW13           0.08966900 W

F2 - Processing parameters
SI                   32768
SF             100.4795039 MHz
WDW                     EM
SSB      0
LB                    1.00 Hz
GB       0
PC                    1.40
```

30

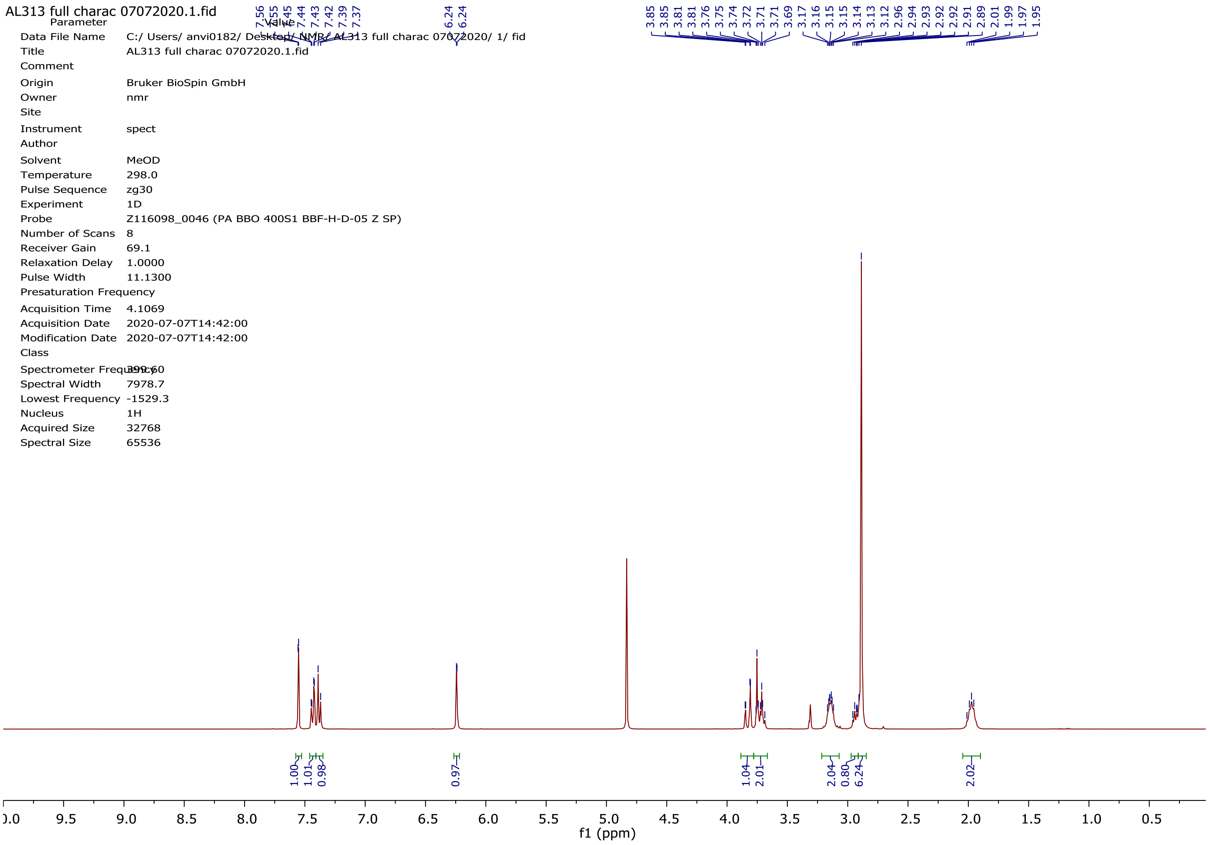
AL313 full charac 07072020.1.fid
Parameter Value
Data File Name C:/ Users/ anvi0182/ Desktop/ NMR/ AL313 full charac 07072020/ 1/ fid
Title AL313 full charac 07072020.1.fid
Comment
Origin Bruker BioSpin GmbH
Owner nmr
Site
Instrument spect
Author
Solvent MeOD
Temperature 298.0
Pulse Sequence zg30
Experiment 1D
Probe Z116098_0046 (PA BBO 400S1 BBF-H-D-05 Z SP)
Number of Scans 8
Receiver Gain 69.1
Relaxation Delay 1.0000
Pulse Width 11.1300
Presaturation Frequency
Acquisition Time 4.1069
Acquisition Date 2020-07-07T14:42:00
Modification Date 2020-07-07T14:42:00
Class
Spectrometer Frequency 399.60
Spectral Width 7978.7
Lowest Frequency -1529.3
Nucleus 1H
Acquired Size 32768
Spectral Size 65536
7.56 7.55 7.45 7.44 7.43 7.42 7.39 7.37
6.24 6.24
3.85 3.85 3.81 3.81 3.76 3.75 3.74 3.72 3.71 3.71 3.69 3.17 3.16 3.15 3.15 3.14 3.13 3.12 2.96 2.94 2.93 2.92 2.92 2.91 2.89 2.01 1.99 1.97 1.95
1.00 1.01 0.98 0.97 1.04 2.01 2.04 0.80 6.24 2.02
f1 (ppm)

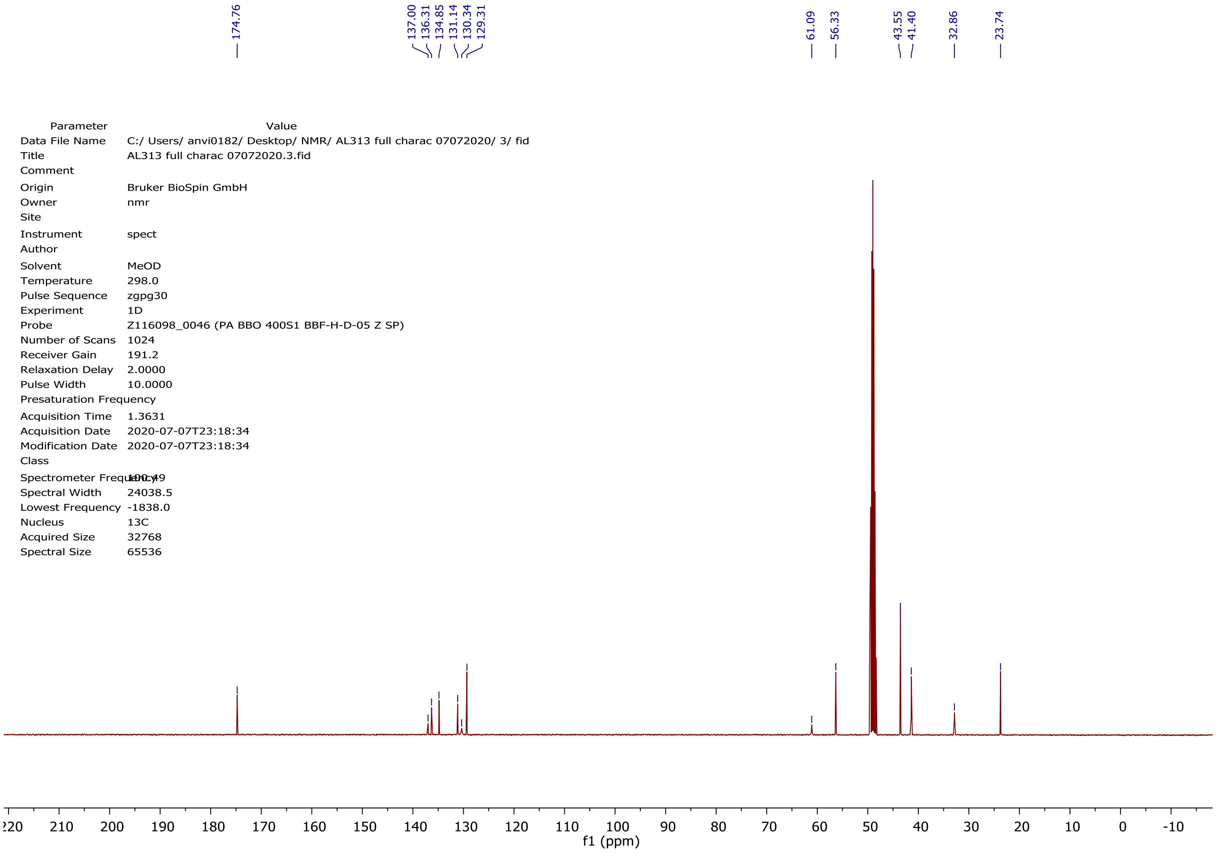
174.76
137.00 136.31 134.85 131.14 130.34 129.31
61.09 56.33 43.55 41.40 32.86 23.74
Parameter Value
Data File Name C:/ Users/ anvi0182/ Desktop/ NMR/ AL313 full charac 07072020/ 3/ fid
Title AL313 full charac 07072020.3.fid
Comment
Origin Bruker BioSpin GmbH
Owner nmr
Site
Instrument spect
Author
Solvent MeOD
Temperature 298.0
Pulse Sequence zgpg30
Experiment 1D
Probe Z116098_0046 (PA BBO 400S1 BBF-H-D-05 Z SP)
Number of Scans 1024
Receiver Gain 191.2
Relaxation Delay 2.0000
Pulse Width 10.0000
Presaturation Frequency
Acquisition Time 1.3631
Acquisition Date 2020-07-07T23:18:34
Modification Date 2020-07-07T23:18:34
Class
Spectrometer Frequency 100.49
Spectral Width 24038.5
Lowest Frequency -1838.0
Nucleus 13C
Acquired Size 32768
Spectral Size 65536
f1 (ppm)

31

AVA-114-1 200619 MeOD full charac.1.fid

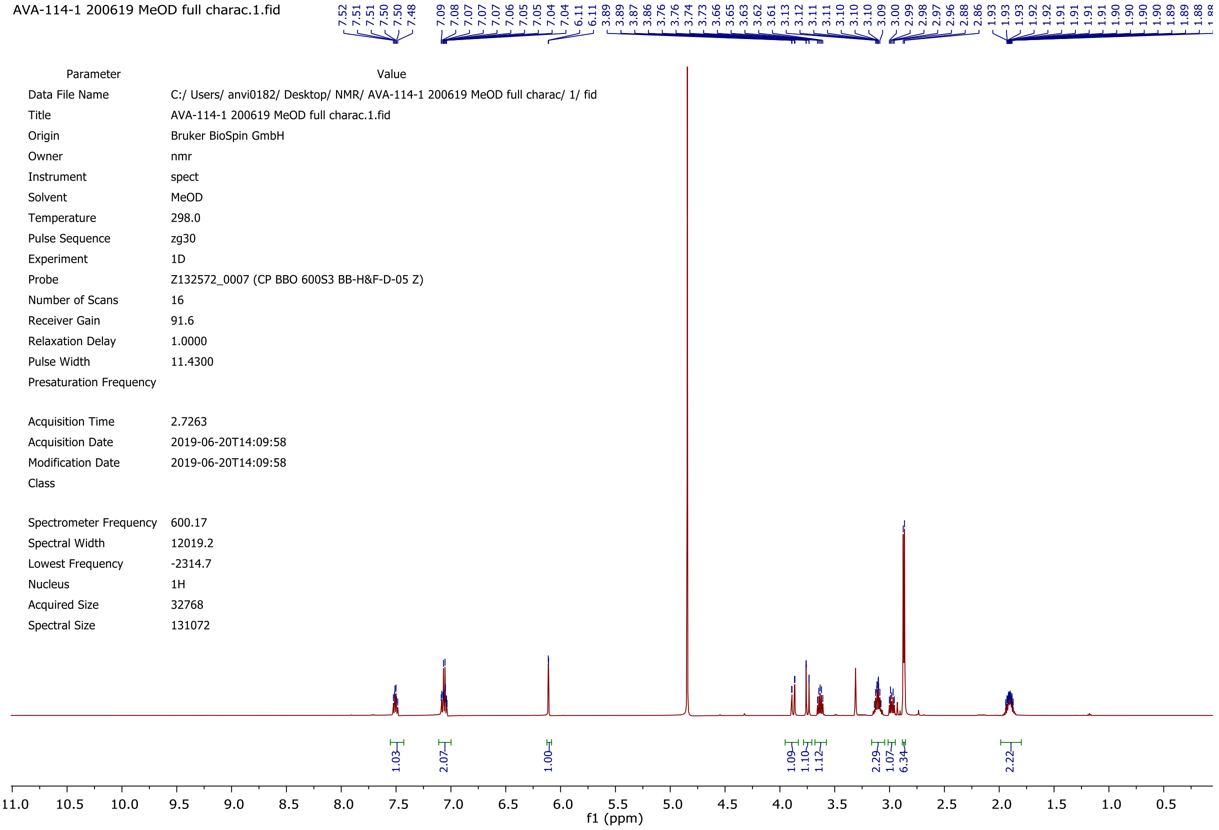

Parameter	Value
Data File Name	C:/ Users/ anvi0182/ Desktop/ NMR/ AVA-114-1 200619 MeOD full charac/ 1/ fid
Title	AVA-114-1 200619 MeOD full charac.1.fid
Origin	Bruker BioSpin GmbH
Owner	nmr
Instrument	spect
Solvent	MeOD
Temperature	298.0
Pulse Sequence	zg30
Experiment	1D
Probe	Z132572_0007 (CP BBO 600S3 BB-H&F-D-05 Z)
Number of Scans	16
Receiver Gain	91.6
Relaxation Delay	1.0000
Pulse Width	11.4300
Presaturation Frequency	
Acquisition Time	2.7263
Acquisition Date	2019-06-20T14:09:58
Modification Date	2019-06-20T14:09:58
Class	
Spectrometer Frequency	600.17
Spectral Width	12019.2
Lowest Frequency	-2314.7
Nucleus	1H
Acquired Size	32768
Spectral Size	131072

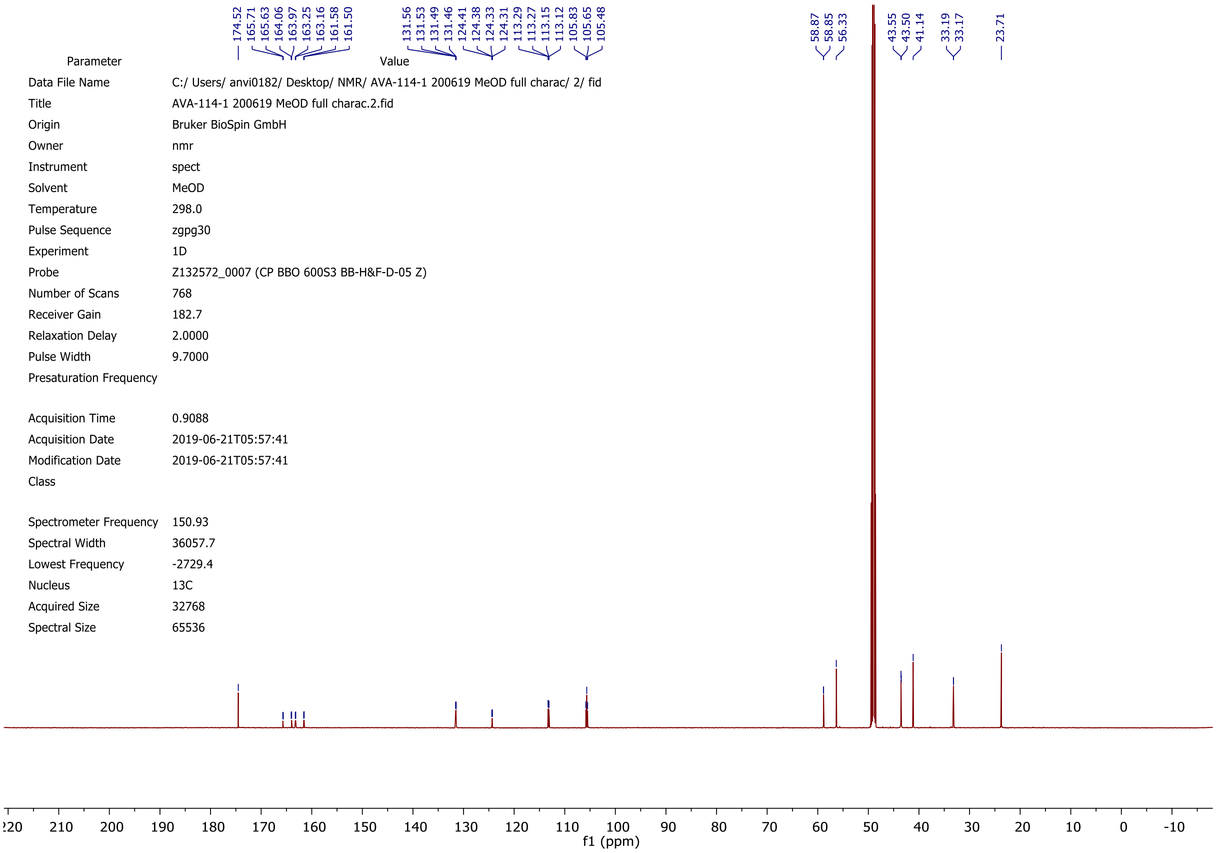

Parameter	Value
Data File Name	C:/ Users/ anvi0182/ Desktop/ NMR/ AVA-114-1 200619 MeOD full charac/ 2/ fid
Title	AVA-114-1 200619 MeOD full charac.2.fid
Origin	Bruker BioSpin GmbH
Owner	nmr
Instrument	spect
Solvent	MeOD
Temperature	298.0
Pulse Sequence	zgpg30
Experiment	1D
Probe	Z132572_0007 (CP BBO 600S3 BB-H&F-D-05 Z)
Number of Scans	768
Receiver Gain	182.7
Relaxation Delay	2.0000
Pulse Width	9.7000
Presaturation Frequency	
Acquisition Time	0.9088
Acquisition Date	2019-06-21T05:57:41
Modification Date	2019-06-21T05:57:41
Class	
Spectrometer Frequency	150.93
Spectral Width	36057.7
Lowest Frequency	-2729.4
Nucleus	13C
Acquired Size	32768
Spectral Size	65536

Parameter	Value
Data File Name	C:/ Users/ anvi0182/ Desktop/ NMR/ AL473 19F MeOD/ 1/ fid
Title	AL473 19F MeOD.1.fid
Origin	Bruker BioSpin GmbH
Owner	nmr
Instrument	spect
Solvent	MeOD
Temperature	298.0
Pulse Sequence	zgfhigqn.2
Experiment	1D
Probe	Z116098_0046 (PA BBO 400S1 BBF-H-D-05 Z SP)
Number of Scans	16
Receiver Gain	191.2
Relaxation Delay	1.0000
Pulse Width	15.5000
Presaturation Frequency	
Acquisition Time	0.7340
Acquisition Date	2020-07-13T16:52:14
Modification Date	2020-07-13T16:52:14
Class	
Spectrometer Frequency	375.96
Spectral Width	89285.7
Lowest Frequency	-82242.9
Nucleus	19F
Acquired Size	65536
Spectral Size	131072

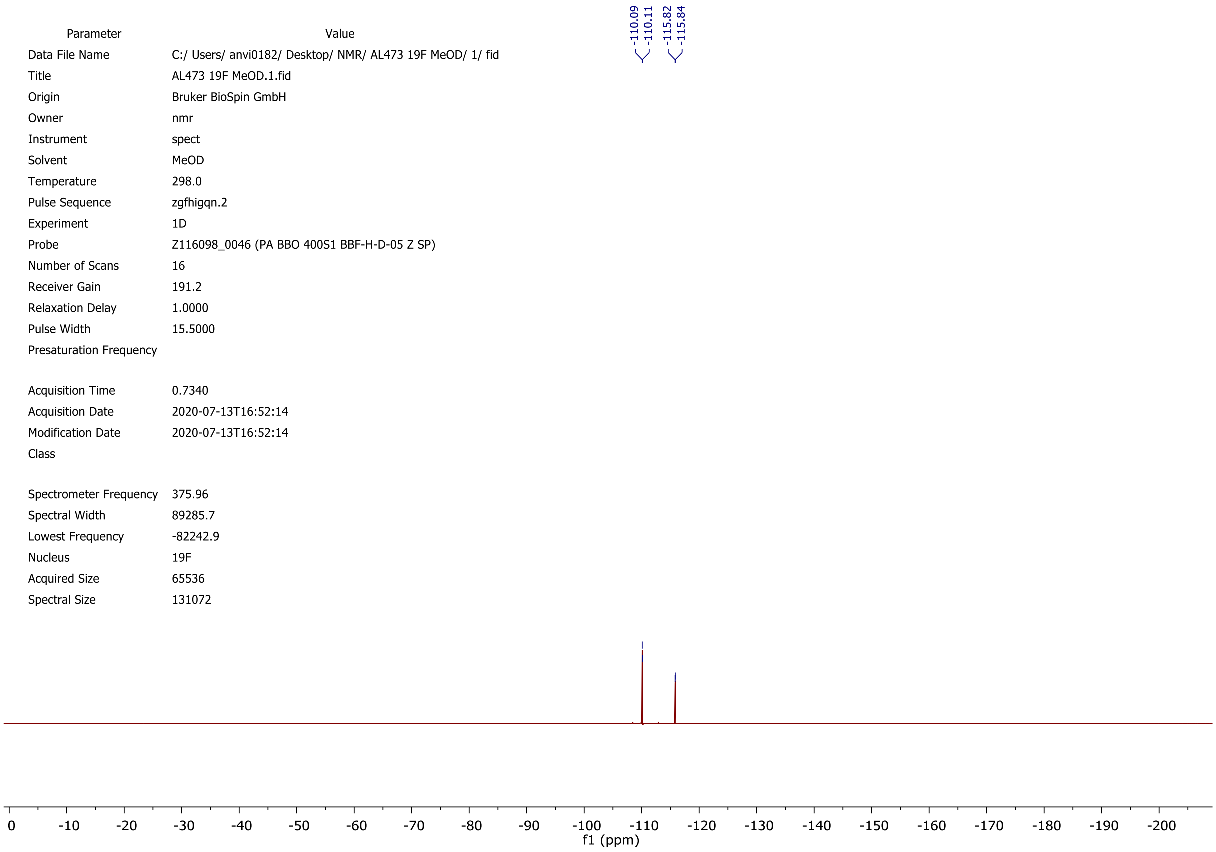

32

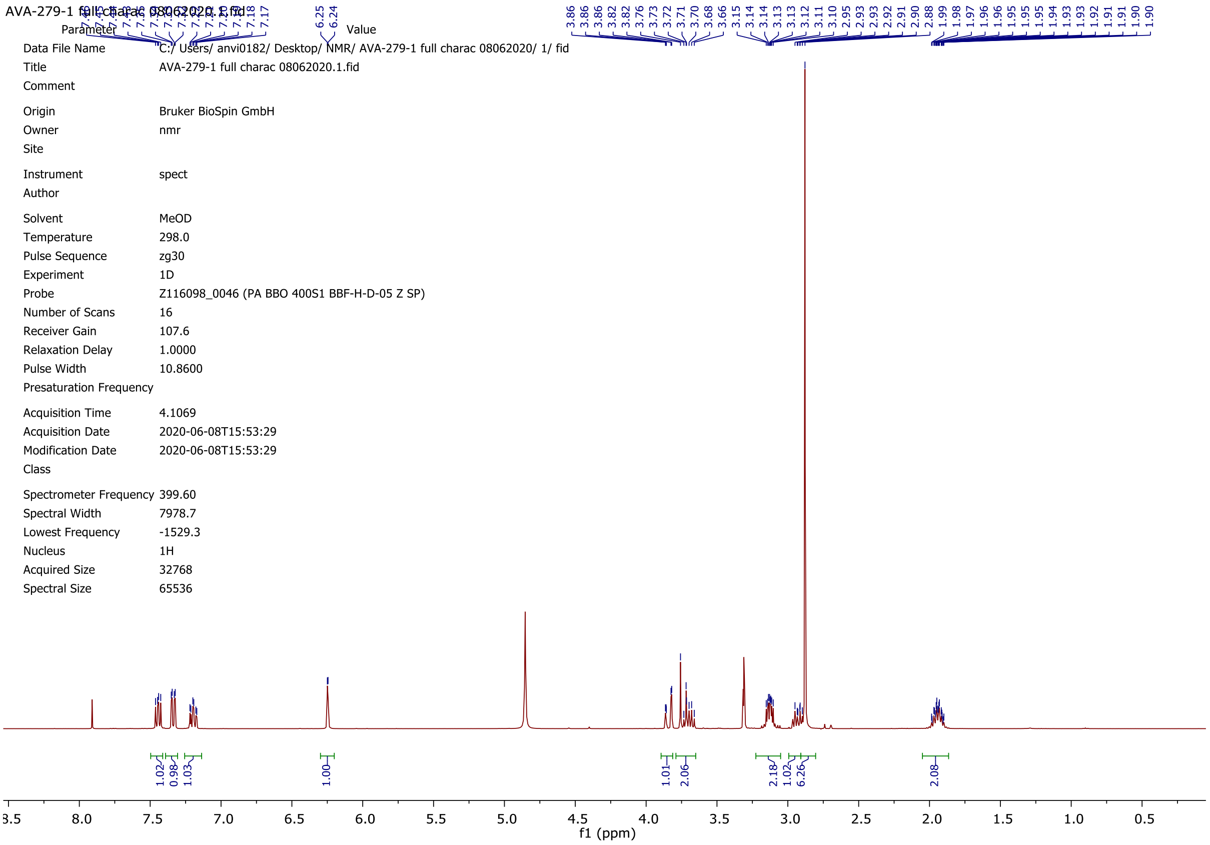
AVA-279-1 full charac 08062020.1.fid
Parameter Value
Data File Name C:/ Users/ anvi0182/ Desktop/ NMR/ AVA-279-1 full charac 08062020/ 1/ fid
Title AVA-279-1 full charac 08062020.1.fid
Comment
Origin Bruker BioSpin GmbH
Owner nmr
Site
Instrument spect
Author
Solvent MeOD
Temperature 298.0
Pulse Sequence zg30
Experiment 1D
Probe Z116098_0046 (PA BBO 400S1 BBF-H-D-05 Z SP)
Number of Scans 16
Receiver Gain 107.6
Relaxation Delay 1.0000
Pulse Width 10.8600
Presaturation Frequency
Acquisition Time 4.1069
Acquisition Date 2020-06-08T15:53:29
Modification Date 2020-06-08T15:53:29
Class
Spectrometer Frequency 399.60
Spectral Width 7978.7
Lowest Frequency -1529.3
Nucleus 1H
Acquired Size 32768
Spectral Size 65536
6.25 6.24
3.86 3.86 3.86 3.82 3.82 3.76 3.73 3.72 3.71 3.70 3.68 3.66 3.15 3.14 3.14 3.13 3.13 3.12 3.11 3.10 2.95 2.93 2.93 2.93 2.92 2.91 2.90 2.88 1.99 1.98 1.97 1.96 1.96 1.95 1.95 1.95 1.94 1.93 1.93 1.92 1.91 1.91 1.90 1.90
1.02 0.98 1.03 1.00 1.01 2.06 2.18 1.02 6.26 2.08
8.5 8.0 7.5 7.0 6.5 6.0 5.5 5.0 4.5 4.0 3.5 3.0 2.5 2.0 1.5 1.0 0.5
f1 (ppm)

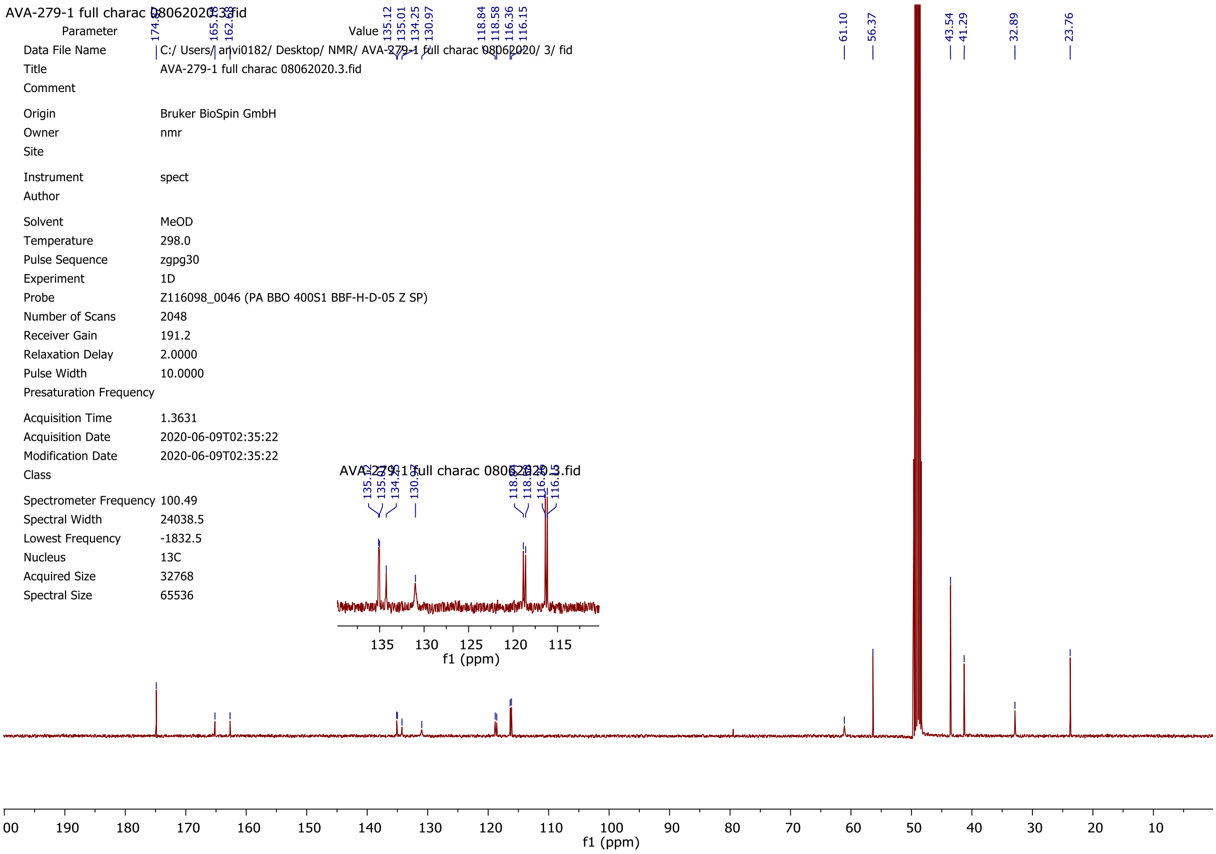
AVA-279-1 full charac 08062020.3.fid
Parameter Value
Data File Name C:/ Users/ anvi0182/ Desktop/ NMR/ AVA-279-1 full charac 08062020/ 3/ fid
Title AVA-279-1 full charac 08062020.3.fid
Comment
Origin Bruker BioSpin GmbH
Owner nmr
Site
Instrument spect
Author
Solvent MeOD
Temperature 298.0
Pulse Sequence zgpg30
Experiment 1D
Probe Z116098_0046 (PA BBO 400S1 BBF-H-D-05 Z SP)
Number of Scans 2048
Receiver Gain 191.2
Relaxation Delay 2.0000
Pulse Width 10.0000
Presaturation Frequency
Acquisition Time 1.3631
Acquisition Date 2020-06-09T02:35:22
Modification Date 2020-06-09T02:35:22
Class
Spectrometer Frequency 100.49
Spectral Width 24038.5
Lowest Frequency -1832.5
Nucleus 13C
Acquired Size 32768
Spectral Size 65536
135.12 135.01 134.25 130.97 118.84 118.58 116.36 116.15
61.10 56.37 43.54 41.29 32.89 23.76
135 130 125 120 115
f1 (ppm)
200 190 180 170 160 150 140 130 120 110 100 90 80 70 60 50 40 30 20 10
f1 (ppm)

AVA-279-1 full charac 08062020.2.fid

Parameter	Value
Data File Name	C:/ Users/ anvi0182/ Desktop/ NMR/ AVA-279-1 full charac 08062020/ 2/ fid
Title	AVA-279-1 full charac 08062020.2.fid
Comment	
Origin	Bruker BioSpin GmbH
Owner	nmr
Site	
Instrument	spect
Author	
Solvent	MeOD
Temperature	298.0
Pulse Sequence	zgfhigqn.2
Experiment	1D
Probe	Z116098_0046 (PA BBO 400S1 BBF-H-D-05 Z SP)
Number of Scans	16
Receiver Gain	191.2
Relaxation Delay	1.0000
Pulse Width	15.5000
Presaturation Frequency	
Acquisition Time	0.7340
Acquisition Date	2020-06-08T15:55:39
Modification Date	2020-06-08T15:55:39
Class	
Spectrometer Frequency	375.96
Spectral Width	89285.7
Lowest Frequency	-82242.9
Nucleus	19F
Acquired Size	65536
Spectral Size	131072

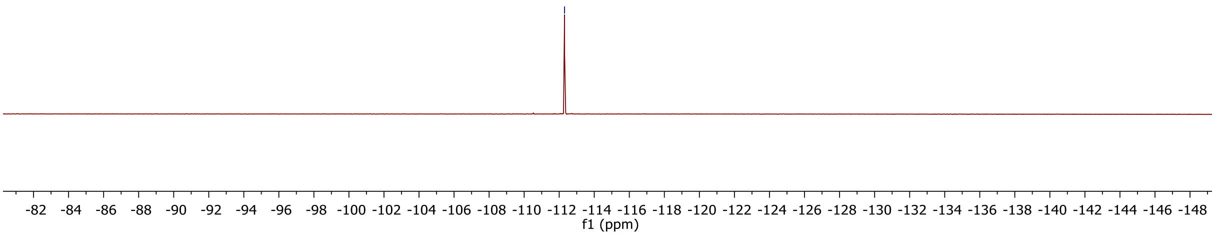

33

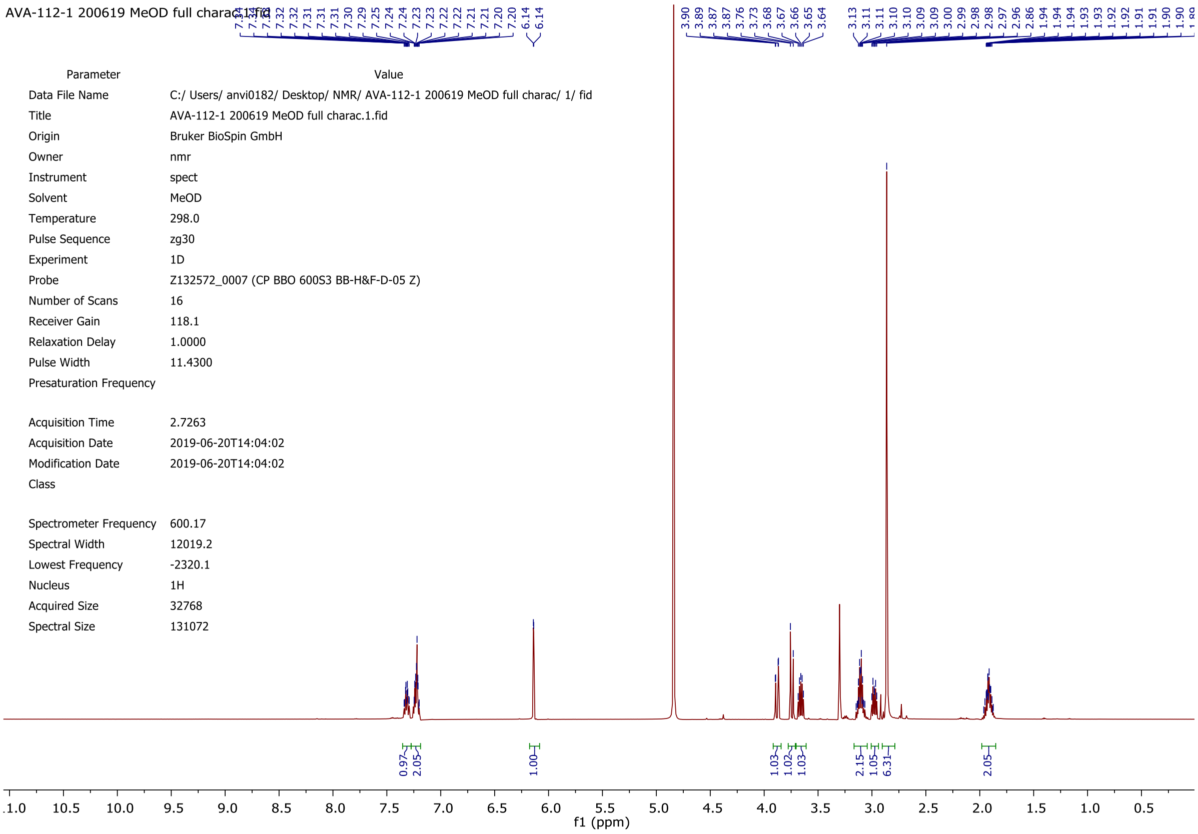

AVA-112-1 200619 MeOD full charac.1.fid
7.32 7.32 7.31 7.31 7.31 7.30 7.29 7.25 7.24 7.24 7.23 7.23 7.22 7.22 7.21 7.21 7.20 6.14 6.14
3.90 3.89 3.87 3.87 3.76 3.73 3.68 3.67 3.66 3.65 3.64
3.13 3.11 3.11 3.10 3.10 3.09 3.09 3.00 2.99 2.98 2.98 2.97 2.96 1.94 1.94 1.94 1.93 1.93 1.92 1.92 1.91 1.91 1.90
Parameter | Value
Data File Name | C:/ Users/ anvi0182/ Desktop/ NMR/ AVA-112-1 200619 MeOD full charac/ 1/ fid
Title | AVA-112-1 200619 MeOD full charac.1.fid
Origin | Bruker BioSpin GmbH
Owner | nmr
Instrument | spect
Solvent | MeOD
Temperature | 298.0
Pulse Sequence | zg30
Experiment | 1D
Probe | Z132572_0007 (CP BBO 600S3 BB-H&F-D-05 Z)
Number of Scans | 16
Receiver Gain | 118.1
Relaxation Delay | 1.0000
Pulse Width | 11.4300
Presaturation Frequency |
Acquisition Time | 2.7263
Acquisition Date | 2019-06-20T14:04:02
Modification Date | 2019-06-20T14:04:02
Class |
Spectrometer Frequency | 600.17
Spectral Width | 12019.2
Lowest Frequency | -2320.1
Nucleus | 1H
Acquired Size | 32768
Spectral Size | 131072
0.97 2.05 1.00 1.03 1.02 1.03 2.15 1.05 6.31 2.05
f1 (ppm)

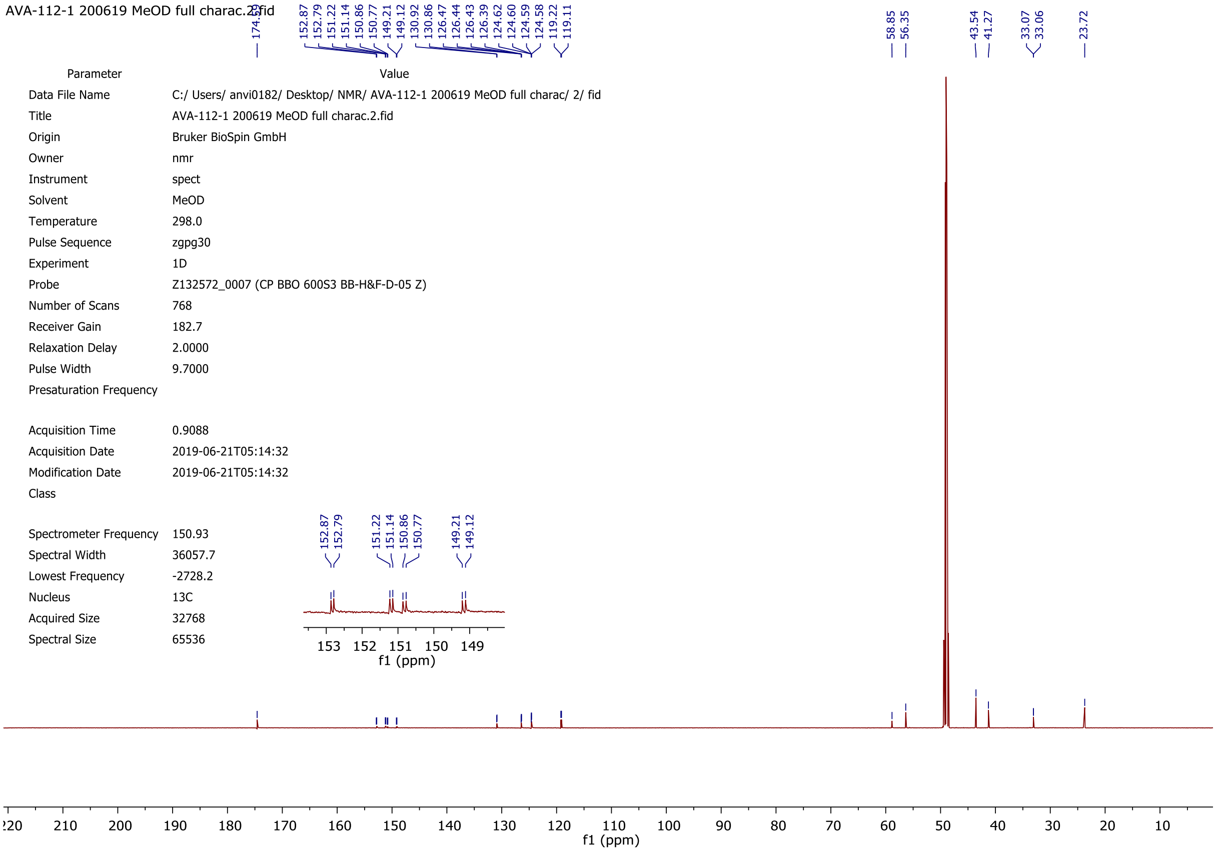

AVA-112-1 200619 MeOD full charac.2.fid
174.52 152.87 152.79 151.22 151.14 150.86 150.77 149.21 149.12 130.92 130.86 126.47 126.44 126.43 126.39 124.62 124.60 124.59 124.58 119.22 119.11
58.85 56.35 43.54 41.27 33.07 33.06 23.72
Parameter | Value
Data File Name | C:/ Users/ anvi0182/ Desktop/ NMR/ AVA-112-1 200619 MeOD full charac/ 2/ fid
Title | AVA-112-1 200619 MeOD full charac.2.fid
Origin | Bruker BioSpin GmbH
Owner | nmr
Instrument | spect
Solvent | MeOD
Temperature | 298.0
Pulse Sequence | zgpg30
Experiment | 1D
Probe | Z132572_0007 (CP BBO 600S3 BB-H&F-D-05 Z)
Number of Scans | 768
Receiver Gain | 182.7
Relaxation Delay | 2.0000
Pulse Width | 9.7000
Presaturation Frequency |
Acquisition Time | 0.9088
Acquisition Date | 2019-06-21T05:14:32
Modification Date | 2019-06-21T05:14:32
Class |
Spectrometer Frequency | 150.93
Spectral Width | 36057.7
Lowest Frequency | -2728.2
Nucleus | 13C
Acquired Size | 32768
Spectral Size | 65536
f1 (ppm)

AL472 19F MeOD.1.fid

Parameter	Value
Data File Name	C:/ Users/ anvi0182/ Desktop/ NMR/ AL472 19F MeOD/ 1/ fid
Title	AL472 19F MeOD.1.fid
Origin	Bruker BioSpin GmbH
Owner	nmr
Instrument	spect
Solvent	MeOD
Temperature	298.0
Pulse Sequence	zgfhigqn.2
Experiment	1D
Probe	Z116098_0046 (PA BBO 400S1 BBF-H-D-05 Z SP)
Number of Scans	16
Receiver Gain	191.2
Relaxation Delay	1.0000
Pulse Width	15.5000
Presaturation Frequency	
Acquisition Time	0.7340
Acquisition Date	2020-07-13T16:46:52
Modification Date	2020-07-13T16:46:52
Class	
Spectrometer Frequency	375.96
Spectral Width	89285.7
Lowest Frequency	-82242.9
Nucleus	19F
Acquired Size	65536
Spectral Size	131072

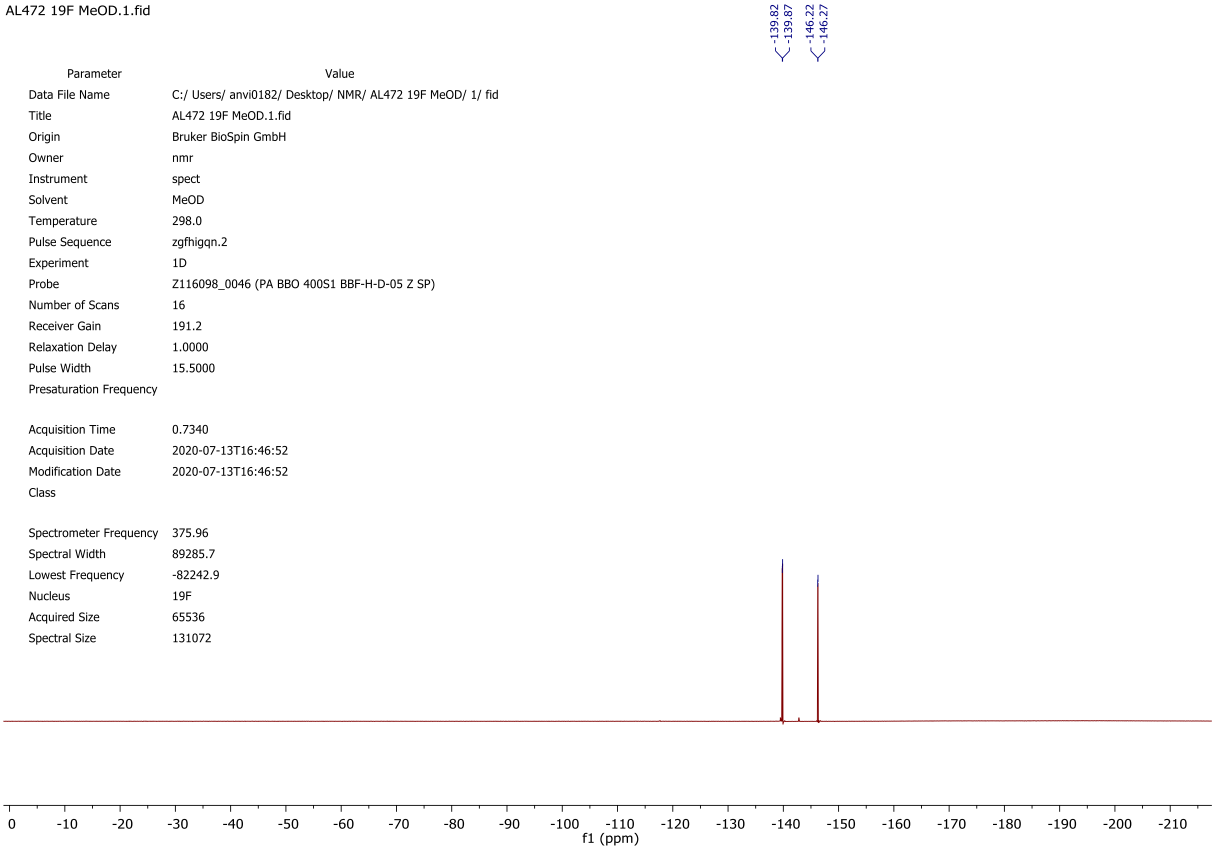

34

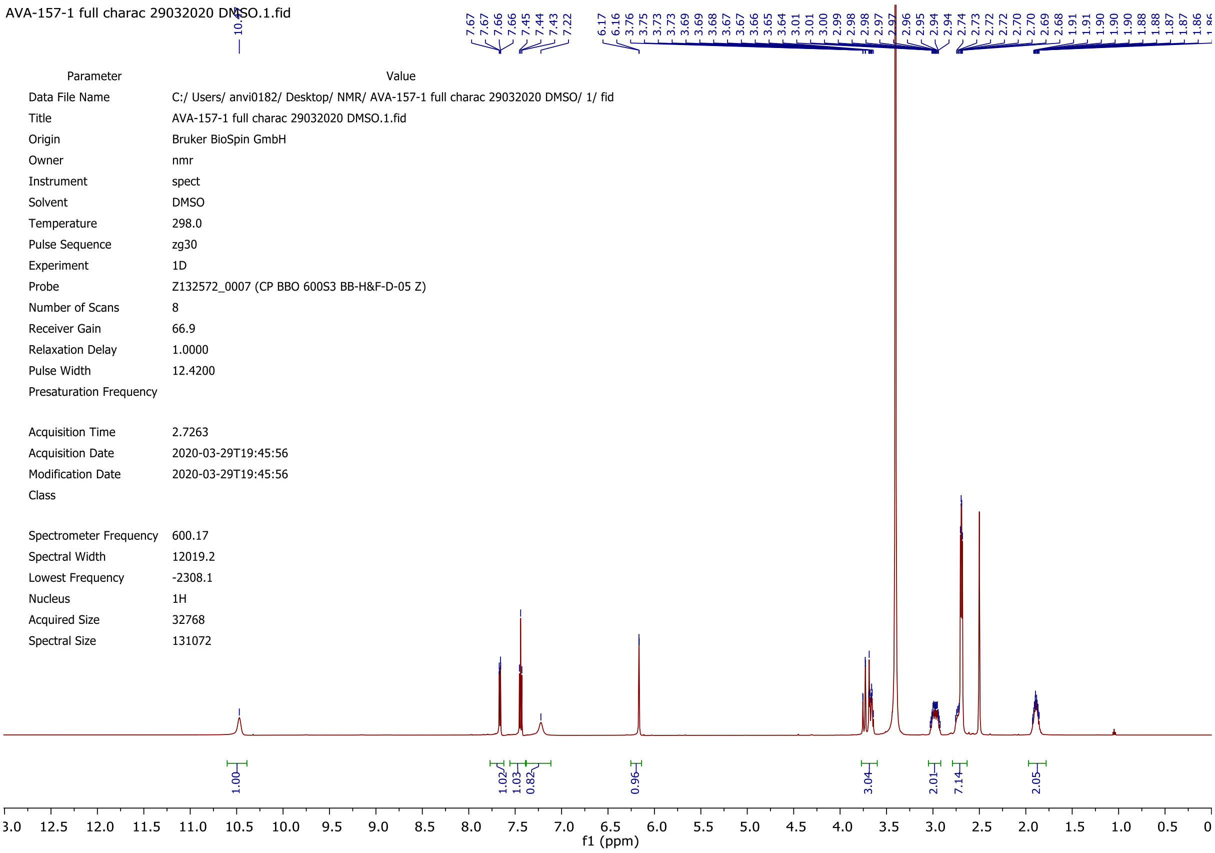

Parameter	Value
Data File Name	C:/ Users/ anvi0182/ Desktop/ NMR/ AVA-157-1 full charac 29032020 DMSO/ 1/ fid
Title	AVA-157-1 full charac 29032020 DMSO.1.fid
Origin	Bruker BioSpin GmbH
Owner	nmr
Instrument	spect
Solvent	DMSO
Temperature	298.0
Pulse Sequence	zg30
Experiment	1D
Probe	Z132572_0007 (CP BBO 600S3 BB-H&F-D-05 Z)
Number of Scans	8
Receiver Gain	66.9
Relaxation Delay	1.0000
Pulse Width	12.4200
Presaturation Frequency	
Acquisition Time	2.7263
Acquisition Date	2020-03-29T19:45:56
Modification Date	2020-03-29T19:45:56
Class	
Spectrometer Frequency	600.17
Spectral Width	12019.2
Lowest Frequency	-2308.1
Nucleus	1H
Acquired Size	32768
Spectral Size	131072

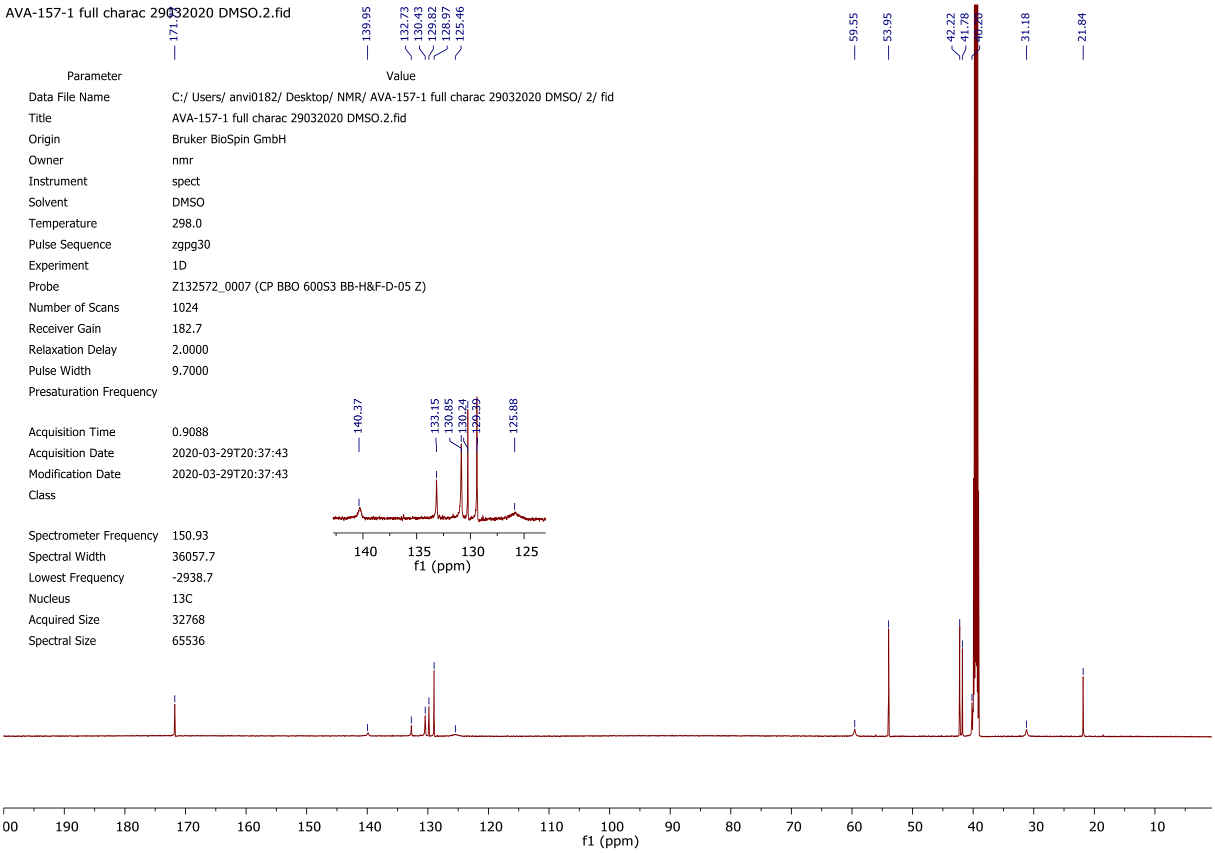

Parameter	Value
Data File Name	C:/ Users/ anvi0182/ Desktop/ NMR/ AVA-157-1 full charac 29032020 DMSO/ 2/ fid
Title	AVA-157-1 full charac 29032020 DMSO.2.fid
Origin	Bruker BioSpin GmbH
Owner	nmr
Instrument	spect
Solvent	DMSO
Temperature	298.0
Pulse Sequence	zgpg30
Experiment	1D
Probe	Z132572_0007 (CP BBO 600S3 BB-H&F-D-05 Z)
Number of Scans	1024
Receiver Gain	182.7
Relaxation Delay	2.0000
Pulse Width	9.7000
Presaturation Frequency	
Acquisition Time	0.9088
Acquisition Date	2020-03-29T20:37:43
Modification Date	2020-03-29T20:37:43
Class	
Spectrometer Frequency	150.93
Spectral Width	36057.7
Lowest Frequency	-2938.7
Nucleus	13C
Acquired Size	32768
Spectral Size	65536

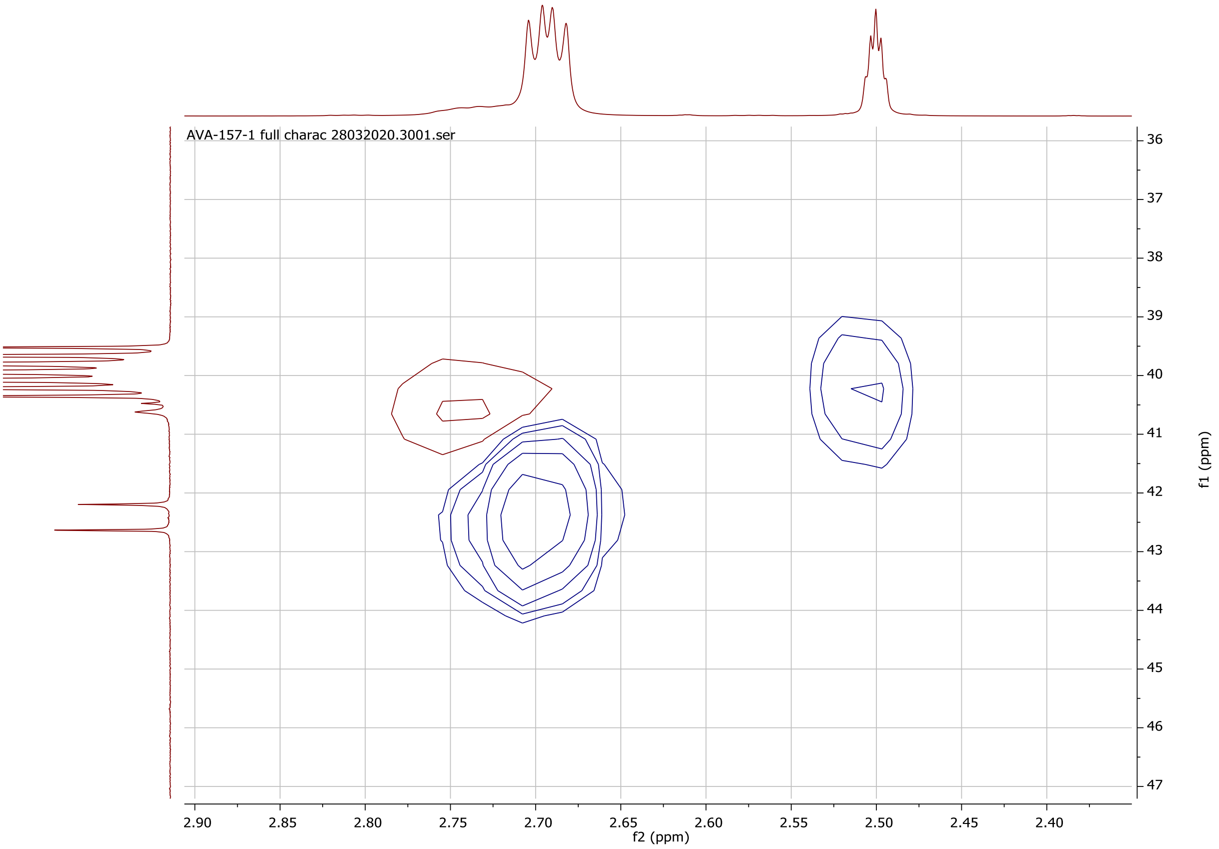
AVA-157-1 full charac 28032020.3001.ser
2.90
2.85
2.80
2.75
2.70
2.65
2.60
2.55
2.50
2.45
2.40
f2 (ppm)
36
37
38
39
40
41
42
43
44
45
46
47
f1 (ppm)

35

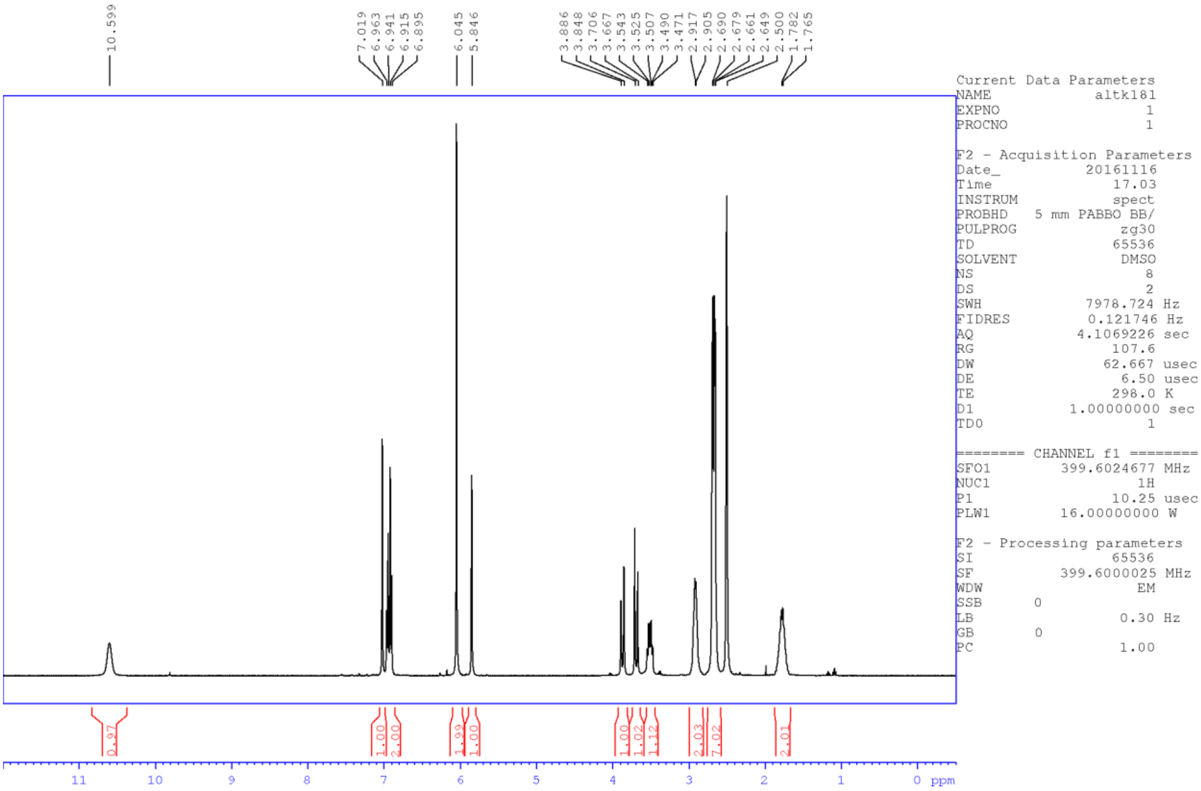
10.599
7.019 6.963 6.941 6.915 6.895
6.045 5.846
3.886 3.848 3.706 3.667 3.543 3.525 3.507 3.490 3.471 2.917 2.905 2.690 2.679 2.661 2.649 2.500 1.782 1.765
0.97 1.00 2.00 1.99 1.00 1.00 1.02 1.12 2.03 7.02 2.01
11 10 9 8 7 6 5 4 3 2 1 0 ppm
Current Data Parameters
NAME altk181
EXPNO 1
PROCNO 1
F2 - Acquisition Parameters
Date_ 20161116
Time 17.03
INSTRUM spect
PROBHD 5 mm PABBO BB/
PULPROG zg30
TD 65536
SOLVENT DMSO
NS 8
DS 2
SWH 7978.724 Hz
FIDRES 0.121746 Hz
AQ 4.1069226 sec
RG 107.6
DW 62.667 usec
DE 6.50 usec
TE 298.0 K
D1 1.00000000 sec
TD0 1
======== CHANNEL f1 ========
SFO1 399.6024677 MHz
NUC1 1H
P1 10.25 usec
PLW1 16.00000000 W
F2 - Processing parameters
SI 65536
SF 399.6000025 MHz
WDW EM
SSB 0
LB 0.30 Hz
GB 0
PC 1.00

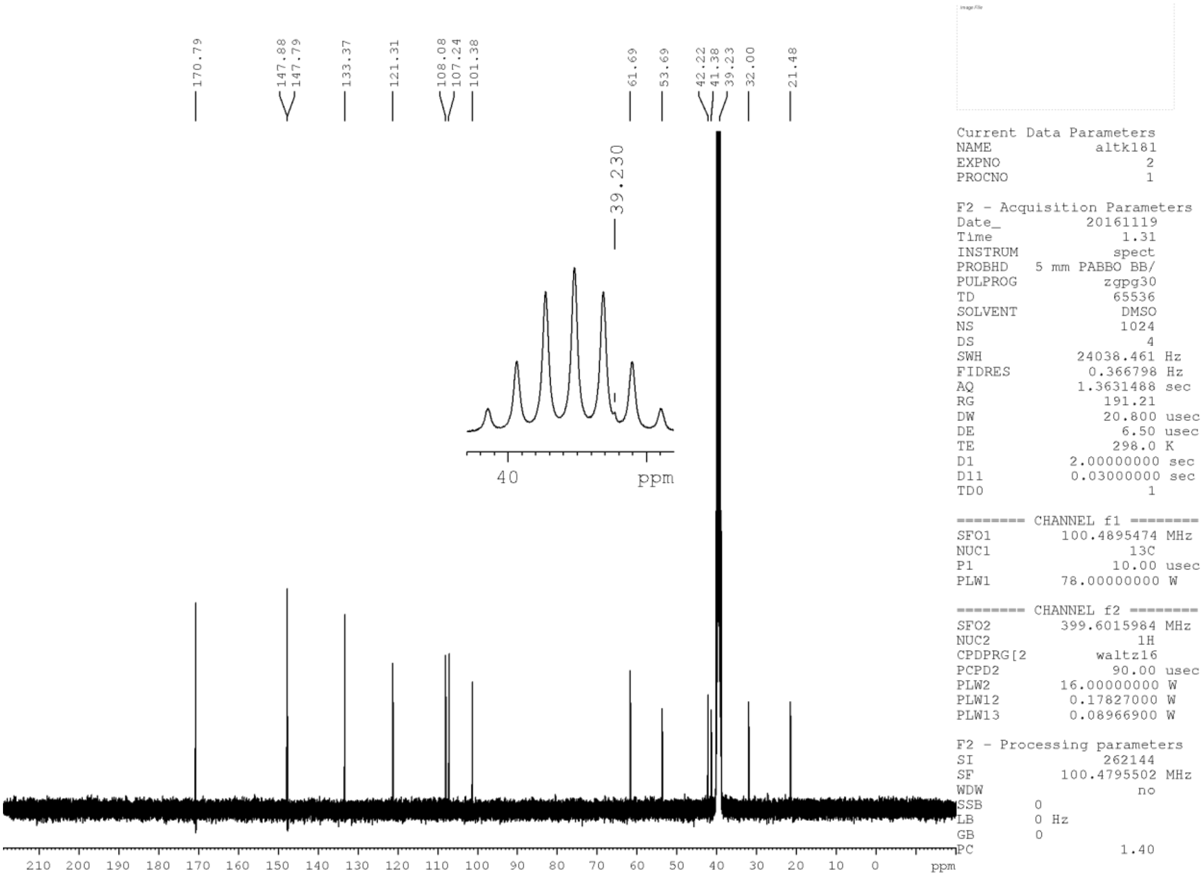
170.79
147.88 147.79
133.37
121.31
108.08 107.24 101.38
61.69
53.69
42.22 41.38 39.23
32.00
21.48
39.230
40 ppm
210 200 190 180 170 160 150 140 130 120 110 100 90 80 70 60 50 40 30 20 10 0 ppm
Current Data Parameters
NAME altk181
EXPNO 2
PROCNO 1
F2 - Acquisition Parameters
Date_ 20161119
Time 1.31
INSTRUM spect
PROBHD 5 mm PABBO BB/
PULPROG zgpg30
TD 65536
SOLVENT DMSO
NS 1024
DS 4
SWH 24038.461 Hz
FIDRES 0.366798 Hz
AQ 1.3631488 sec
RG 191.21
DW 20.800 usec
DE 6.50 usec
TE 298.0 K
D1 2.00000000 sec
D11 0.03000000 sec
TD0 1
======== CHANNEL f1 ========
SFO1 100.4895474 MHz
NUC1 13C
P1 10.00 usec
PLW1 78.00000000 W
======== CHANNEL f2 ========
SFO2 399.6015984 MHz
NUC2 1H
CPDPRG[2 waltz16
PCPD2 90.00 usec
PLW2 16.00000000 W
PLW12 0.17827000 W
PLW13 0.08966900 W
F2 - Processing parameters
SI 262144
SF 100.4795502 MHz
WDW no
SSB 0
LB 0 Hz
GB 0
PC 1.40

36

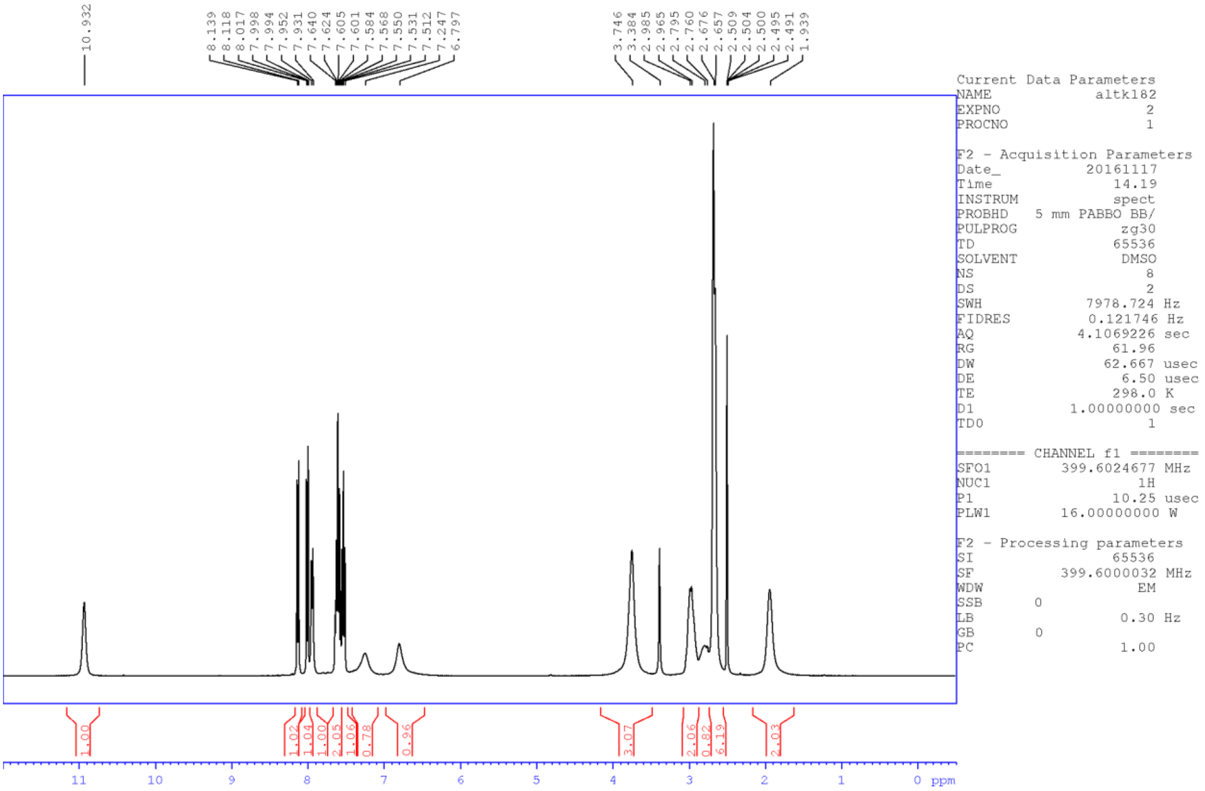
10.932
8.139 8.118 8.017 7.998 7.994 7.952 7.931 7.640 7.624 7.605 7.601 7.584 7.568 7.550 7.531 7.512 7.247 6.797
3.746 3.384 2.985 2.965 2.795 2.760 2.676 2.657 2.509 2.504 2.500 2.495 2.491 1.939
1.00 1.02 1.04 1.00 2.05 1.06 0.78 0.96 3.07 2.06 0.82 6.19 2.03
11 10 9 8 7 6 5 4 3 2 1 0 ppm
Current Data Parameters
NAME altk182
EXPNO 2
PROCNO 1
F2 - Acquisition Parameters
Date_ 20161117
Time 14.19
INSTRUM spect
PROBHD 5 mm PABBO BB/
PULPROG zg30
TD 65536
SOLVENT DMSO
NS 8
DS 2
SWH 7978.724 Hz
FIDRES 0.121746 Hz
AQ 4.1069226 sec
RG 61.96
DW 62.667 usec
DE 6.50 usec
TE 298.0 K
D1 1.00000000 sec
TD0 1
======== CHANNEL f1 ========
SFO1 399.6024677 MHz
NUC1 1H
P1 10.25 usec
PLW1 16.00000000 W
F2 - Processing parameters
SI 65536
SF 399.6000032 MHz
WDW EM
SSB 0
LB 0.30 Hz
GB 0
PC 1.00

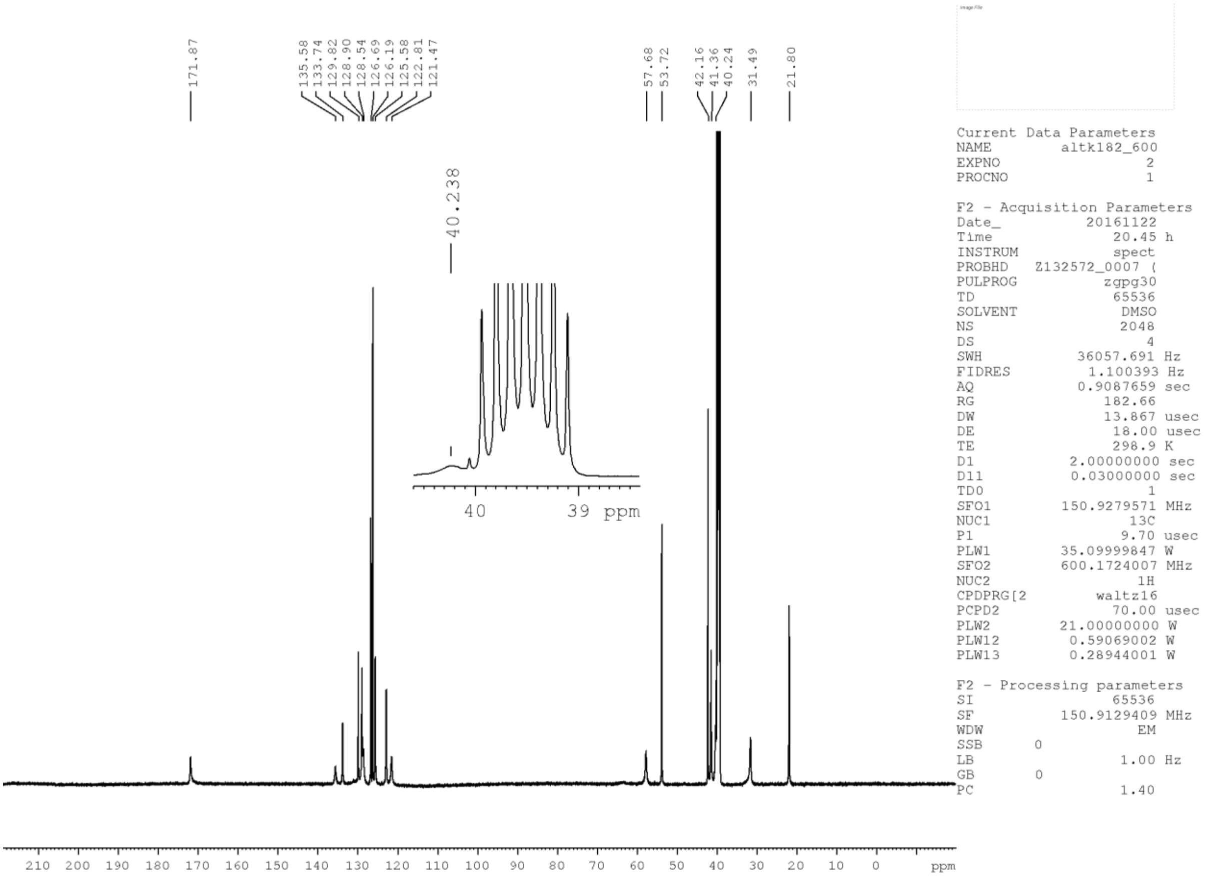
171.87
135.58 133.74 129.82 128.90 128.54 126.69 126.19 125.58 122.81 121.47
57.68 53.72
42.16 41.36 40.24
31.49
21.80
40.238
40 39 ppm
210 200 190 180 170 160 150 140 130 120 110 100 90 80 70 60 50 40 30 20 10 0 ppm
Current Data Parameters
NAME altk182_600
EXPNO 2
PROCNO 1
F2 - Acquisition Parameters
Date_ 20161122
Time 20.45 h
INSTRUM spect
PROBHD Z132572_0007 (
PULPROG zgpg30
TD 65536
SOLVENT DMSO
NS 2048
DS 4
SWH 36057.691 Hz
FIDRES 1.100393 Hz
AQ 0.9087659 sec
RG 182.66
DW 13.867 usec
DE 18.00 usec
TE 298.9 K
D1 2.00000000 sec
D11 0.03000000 sec
TD0 1
SFO1 150.9279571 MHz
NUC1 13C
P1 9.70 usec
PLW1 35.09999847 W
SFO2 600.1724007 MHz
NUC2 1H
CPDPRG[2 waltz16
PCPD2 70.00 usec
PLW2 21.00000000 W
PLW12 0.59069002 W
PLW13 0.28944001 W
F2 - Processing parameters
SI 65536
SF 150.9129409 MHz
WDW EM
SSB 0
LB 1.00 Hz
GB 0
PC 1.40

37

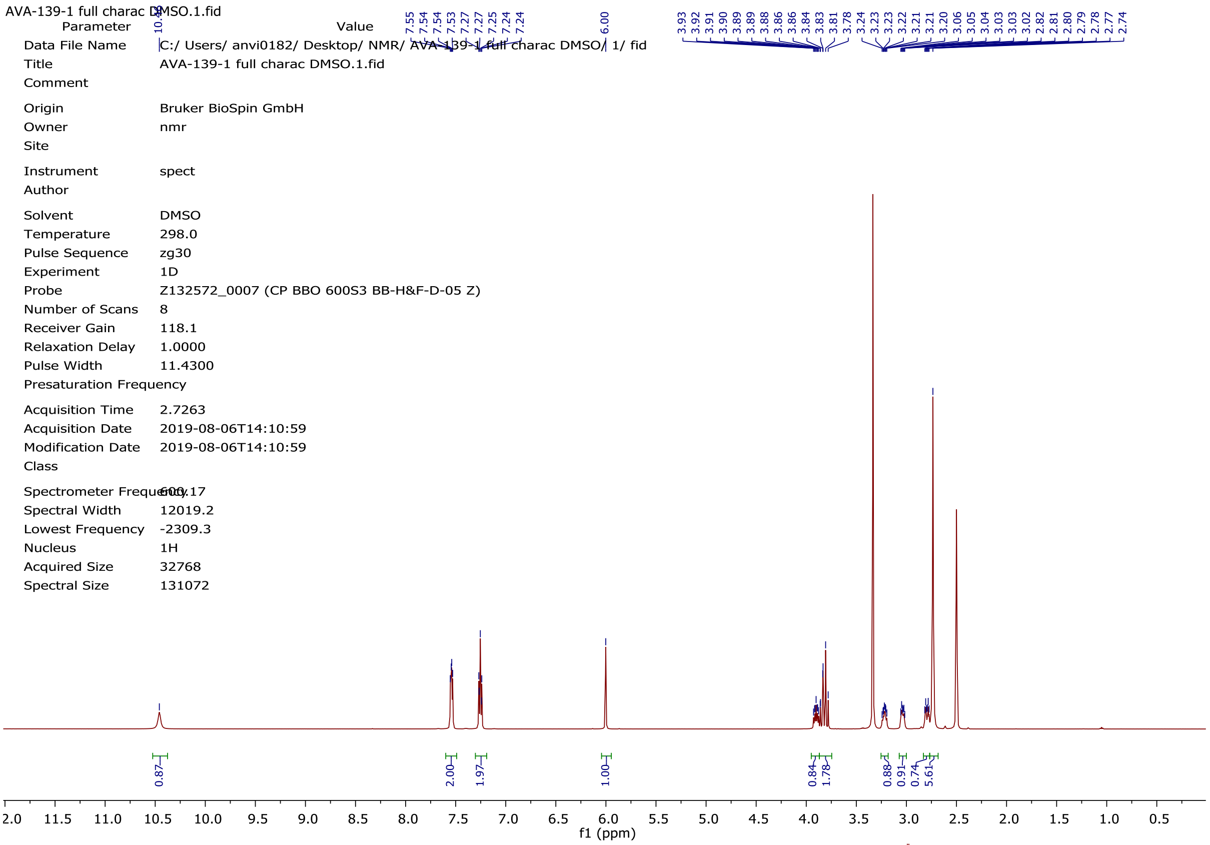

AVA-139-1 full charac DMSO.1.fid

Parameter	Value
Data File Name	C:/ Users/ anvi0182/ Desktop/ NMR/ AVA-139-1 full charac DMSO/ 1/ fid
Title	AVA-139-1 full charac DMSO.1.fid
Comment	
Origin	Bruker BioSpin GmbH
Owner	nmr
Site	
Instrument	spect
Author	
Solvent	DMSO
Temperature	298.0
Pulse Sequence	zg30
Experiment	1D
Probe	Z132572_0007 (CP BBO 600S3 BB-H&F-D-05 Z)
Number of Scans	8
Receiver Gain	118.1
Relaxation Delay	1.0000
Pulse Width	11.4300
Presaturation Frequency	
Acquisition Time	2.7263
Acquisition Date	2019-08-06T14:10:59
Modification Date	2019-08-06T14:10:59
Class	
Spectrometer Frequency	600.17
Spectral Width	12019.2
Lowest Frequency	-2309.3
Nucleus	1H
Acquired Size	32768
Spectral Size	131072

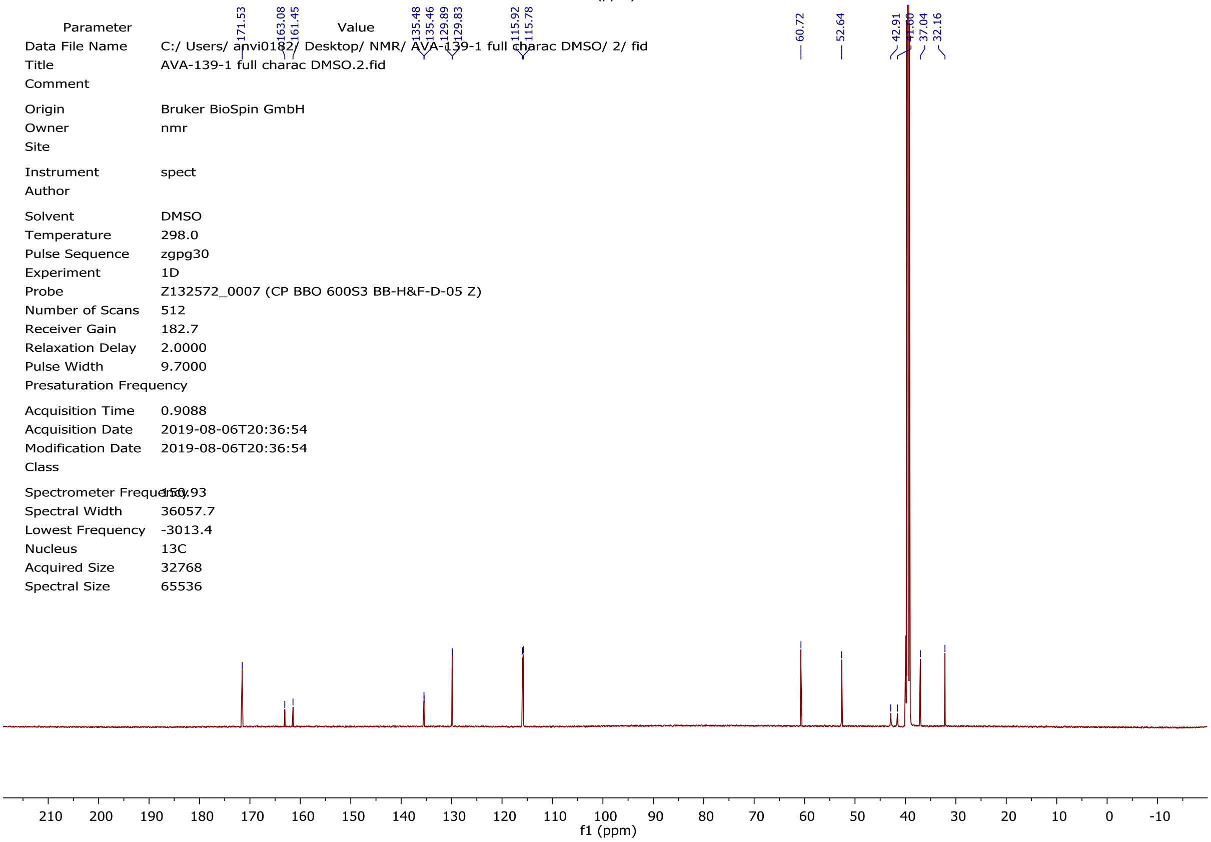

Parameter	Value
Data File Name	C:/ Users/ anvi0182/ Desktop/ NMR/ AVA-139-1 full charac DMSO/ 2/ fid
Title	AVA-139-1 full charac DMSO.2.fid
Comment	
Origin	Bruker BioSpin GmbH
Owner	nmr
Site	
Instrument	spect
Author	
Solvent	DMSO
Temperature	298.0
Pulse Sequence	zgpg30
Experiment	1D
Probe	Z132572_0007 (CP BBO 600S3 BB-H&F-D-05 Z)
Number of Scans	512
Receiver Gain	182.7
Relaxation Delay	2.0000
Pulse Width	9.7000
Presaturation Frequency	
Acquisition Time	0.9088
Acquisition Date	2019-08-06T20:36:54
Modification Date	2019-08-06T20:36:54
Class	
Spectrometer Frequency	150.93
Spectral Width	36057.7
Lowest Frequency	-3013.4
Nucleus	13C
Acquired Size	32768
Spectral Size	65536

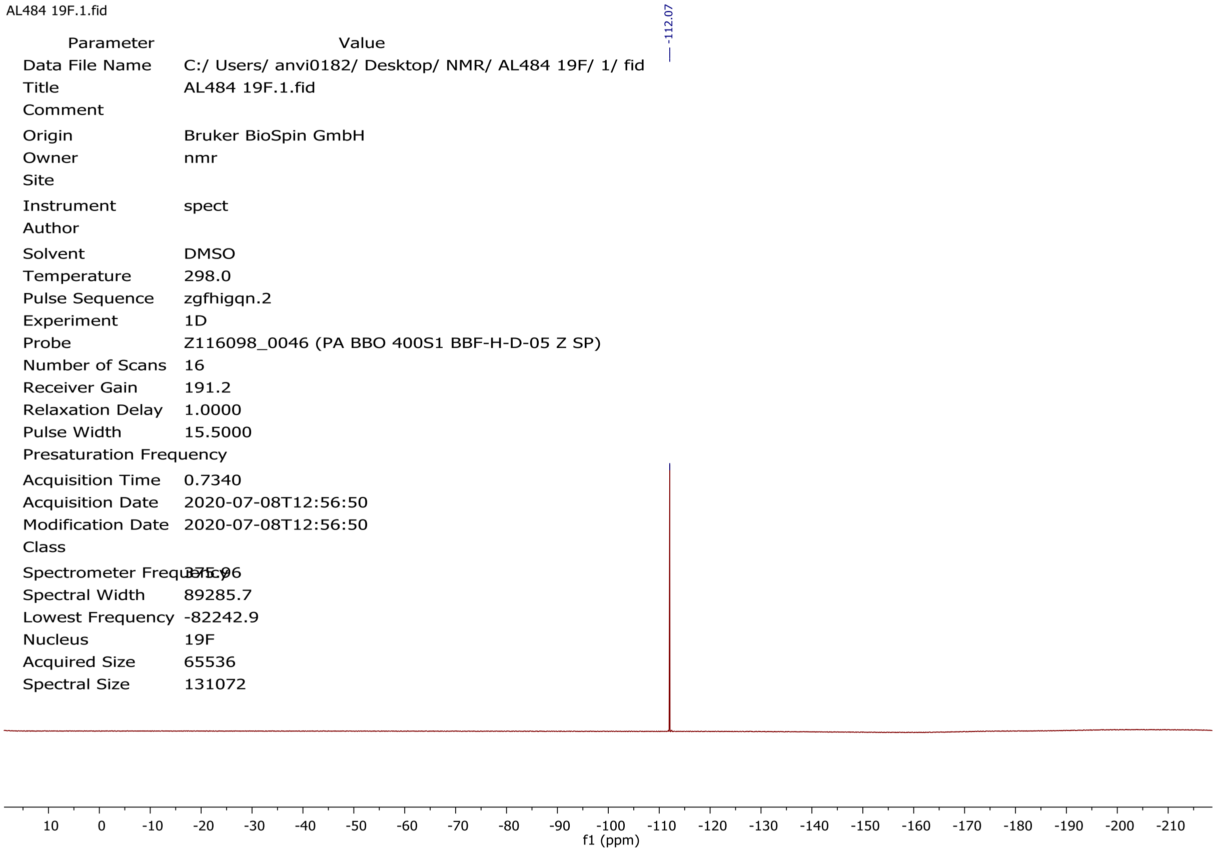

Parameter	Value
Data File Name	C:/ Users/ anvi0182/ Desktop/ NMR/ AL484 19F/ 1/ fid
Title	AL484 19F.1.fid
Comment	
Origin	Bruker BioSpin GmbH
Owner	nmr
Site	
Instrument	spect
Author	
Solvent	DMSO
Temperature	298.0
Pulse Sequence	zgfhigqn.2
Experiment	1D
Probe	Z116098_0046 (PA BBO 400S1 BBF-H-D-05 Z SP)
Number of Scans	16
Receiver Gain	191.2
Relaxation Delay	1.0000
Pulse Width	15.5000
Presaturation Frequency	
Acquisition Time	0.7340
Acquisition Date	2020-07-08T12:56:50
Modification Date	2020-07-08T12:56:50
Class	
Spectrometer Frequency	376.96
Spectral Width	89285.7
Lowest Frequency	-82242.9
Nucleus	19F
Acquired Size	65536
Spectral Size	131072

38

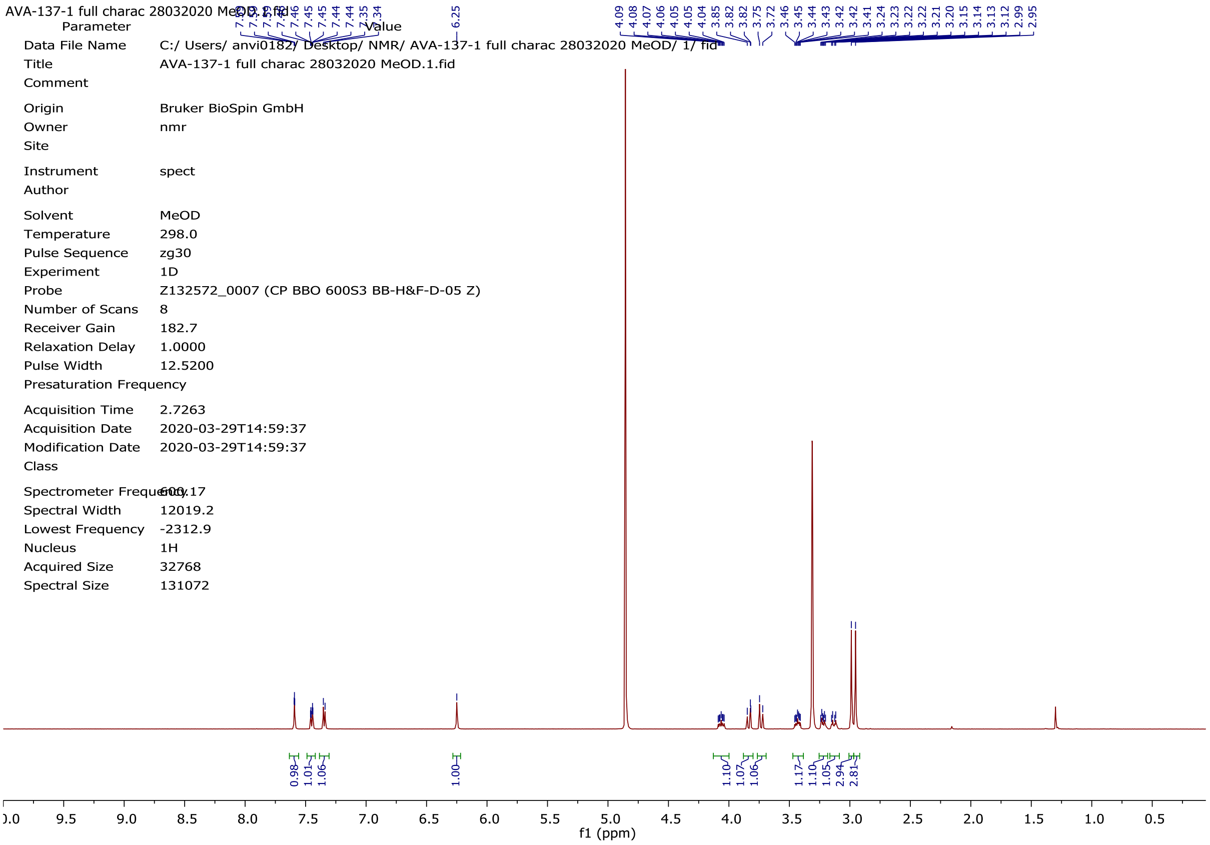
AVA-137-1 full charac 28032020 MeOD.1.fid
Parameter
Value
Data File Name C:/ Users/ anvi0182/ Desktop/ NMR/ AVA-137-1 full charac 28032020 MeOD/ 1/ fid
Title AVA-137-1 full charac 28032020 MeOD.1.fid
Comment
Origin Bruker BioSpin GmbH
Owner nmr
Site
Instrument spect
Author
Solvent MeOD
Temperature 298.0
Pulse Sequence zg30
Experiment 1D
Probe Z132572_0007 (CP BBO 600S3 BB-H&F-D-05 Z)
Number of Scans 8
Receiver Gain 182.7
Relaxation Delay 1.0000
Pulse Width 12.5200
Presaturation Frequency
Acquisition Time 2.7263
Acquisition Date 2020-03-29T14:59:37
Modification Date 2020-03-29T14:59:37
Class
Spectrometer Frequency 600.17
Spectral Width 12019.2
Lowest Frequency -2312.9
Nucleus 1H
Acquired Size 32768
Spectral Size 131072
7.46 7.45 7.45 7.44 7.44 7.35 7.34 6.25
4.09 4.08 4.07 4.06 4.05 4.05 4.05 4.04 3.85 3.82 3.82 3.75 3.72 3.46 3.45 3.44 3.43 3.42 3.42 3.41 3.24 3.23 3.22 3.22 3.21 3.20 3.15 3.14 3.13 3.12 2.99 2.95
0.98 1.01 1.06 1.00 1.10 1.07 1.06 1.17 1.10 1.05 2.94 2.81
9.5 9.0 8.5 8.0 7.5 7.0 6.5 6.0 5.5 5.0 4.5 4.0 3.5 3.0 2.5 2.0 1.5 1.0 0.5
f1 (ppm)

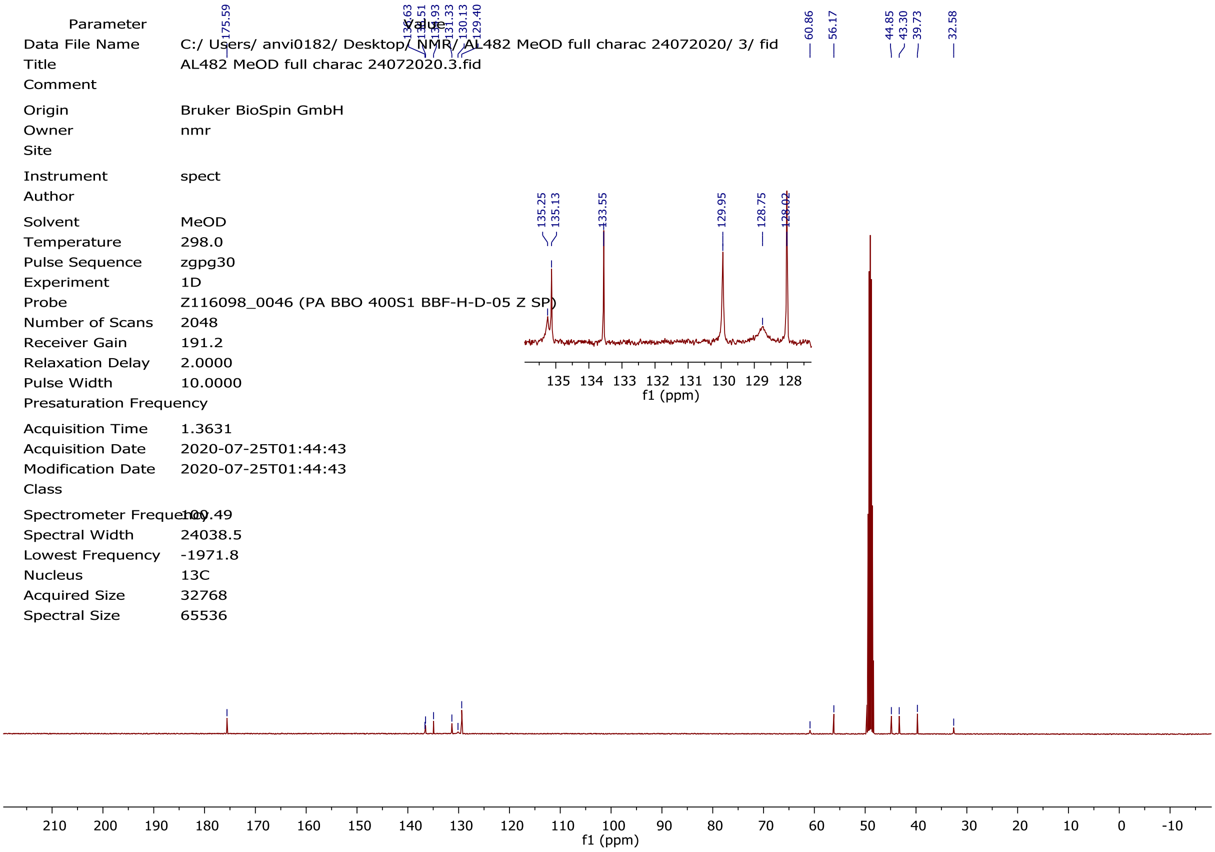
Parameter
Value
Data File Name C:/ Users/ anvi0182/ Desktop/ NMR/ AL482 MeOD full charac 24072020/ 3/ fid
Title AL482 MeOD full charac 24072020.3.fid
Comment
Origin Bruker BioSpin GmbH
Owner nmr
Site
Instrument spect
Author
Solvent MeOD
Temperature 298.0
Pulse Sequence zgpg30
Experiment 1D
Probe Z116098_0046 (PA BBO 400S1 BBF-H-D-05 Z SP)
Number of Scans 2048
Receiver Gain 191.2
Relaxation Delay 2.0000
Pulse Width 10.0000
Presaturation Frequency
Acquisition Time 1.3631
Acquisition Date 2020-07-25T01:44:43
Modification Date 2020-07-25T01:44:43
Class
Spectrometer Frequency 100.49
Spectral Width 24038.5
Lowest Frequency -1971.8
Nucleus 13C
Acquired Size 32768
Spectral Size 65536
175.59 136.63 135.51 133.93 131.33 130.13 129.40
60.86 56.17 44.85 43.30 39.73 32.58
135.25 135.13 133.55 129.95 128.75 128.02
135 134 133 132 131 130 129 128
f1 (ppm)
210 200 190 180 170 160 150 140 130 120 110 100 90 80 70 60 50 40 30 20 10 0 -10
f1 (ppm)

39

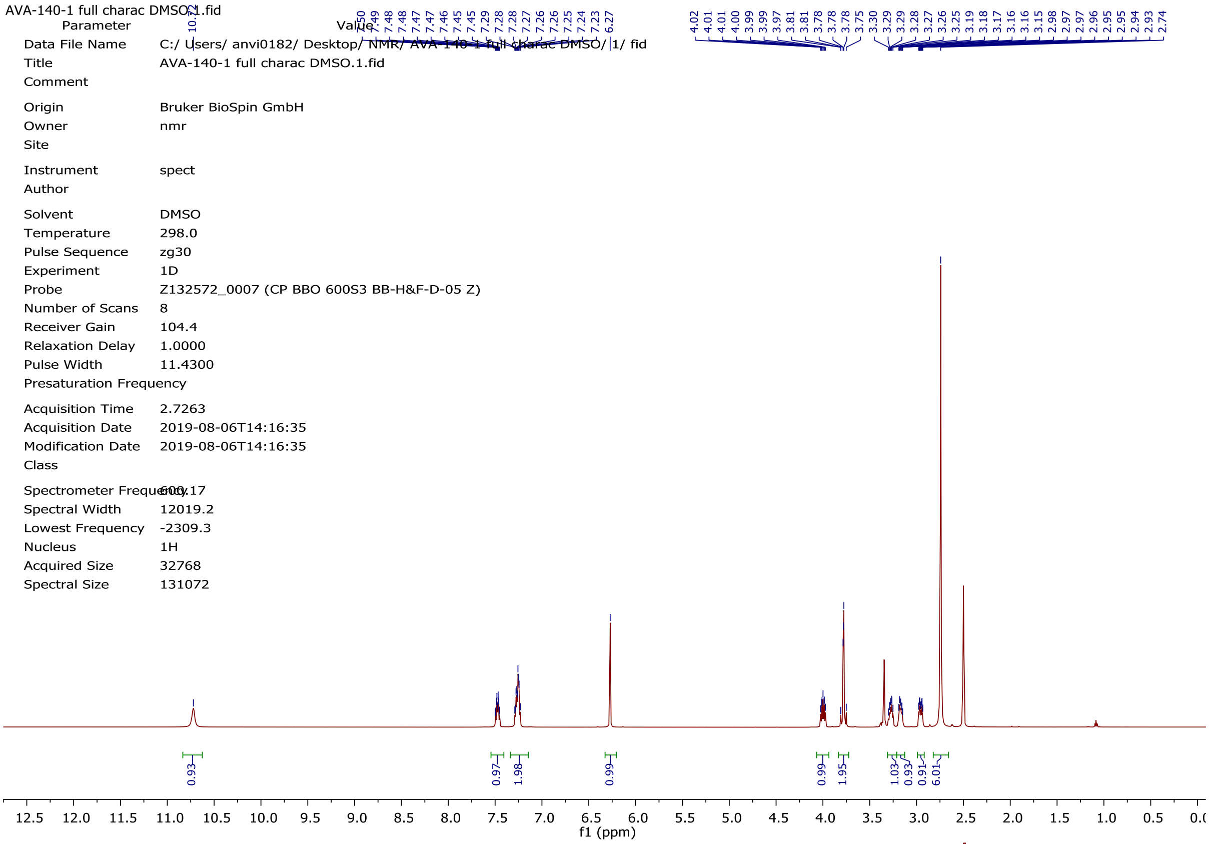

AVA-140-1 full charac DMSO.1.fid
Parameter Value
Data File Name C:/ Users/ anvi0182/ Desktop/ NMR/ AVA-140-1 full charac DMSO/ 1/ fid
Title AVA-140-1 full charac DMSO.1.fid
Comment
Origin Bruker BioSpin GmbH
Owner nmr
Site
Instrument spect
Author
Solvent DMSO
Temperature 298.0
Pulse Sequence zg30
Experiment 1D
Probe Z132572_0007 (CP BBO 600S3 BB-H&F-D-05 Z)
Number of Scans 8
Receiver Gain 104.4
Relaxation Delay 1.0000
Pulse Width 11.4300
Presaturation Frequency
Acquisition Time 2.7263
Acquisition Date 2019-08-06T14:16:35
Modification Date 2019-08-06T14:16:35
Class
Spectrometer Frequency 600.17
Spectral Width 12019.2
Lowest Frequency -2309.3
Nucleus 1H
Acquired Size 32768
Spectral Size 131072
10.72
7.50 7.49 7.48 7.48 7.47 7.47 7.46 7.45 7.45 7.29 7.28 7.28 7.27 7.26 7.26 7.25 7.24 7.23
6.27
4.02 4.01 4.01 4.00 3.99 3.99 3.97 3.81 3.78 3.78 3.78 3.75 3.30 3.29 3.29 3.28 3.27 3.26 3.25 3.19 3.18 3.17 3.16 3.16 3.15 2.98 2.97 2.97 2.96 2.95 2.95 2.94 2.93 2.74
0.93 0.97 1.98 0.99 0.99 1.95 1.03 0.93 0.91 6.01
f1 (ppm)

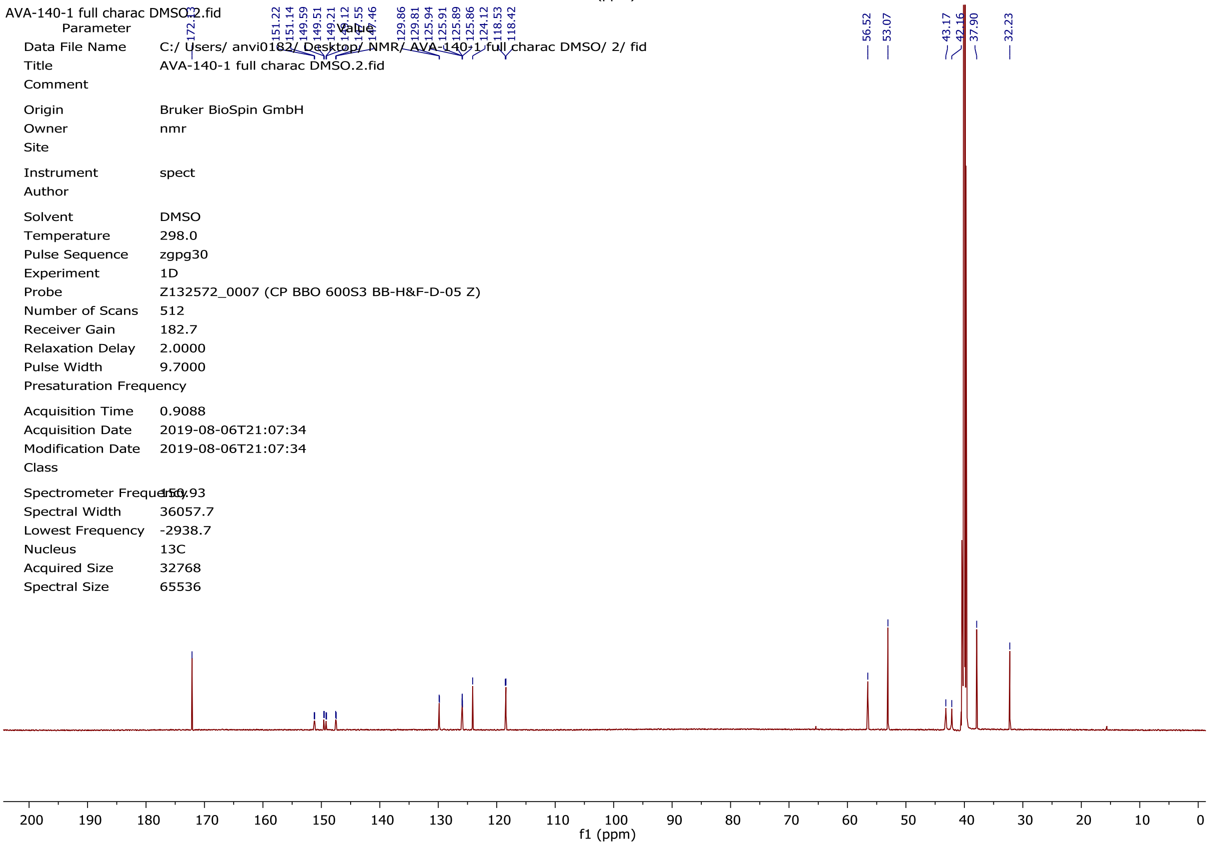

AVA-140-1 full charac DMSO.2.fid
Parameter Value
Data File Name C:/ Users/ anvi0182/ Desktop/ NMR/ AVA-140-1 full charac DMSO/ 2/ fid
Title AVA-140-1 full charac DMSO.2.fid
Comment
Origin Bruker BioSpin GmbH
Owner nmr
Site
Instrument spect
Author
Solvent DMSO
Temperature 298.0
Pulse Sequence zgpg30
Experiment 1D
Probe Z132572_0007 (CP BBO 600S3 BB-H&F-D-05 Z)
Number of Scans 512
Receiver Gain 182.7
Relaxation Delay 2.0000
Pulse Width 9.7000
Presaturation Frequency
Acquisition Time 0.9088
Acquisition Date 2019-08-06T21:07:34
Modification Date 2019-08-06T21:07:34
Class
Spectrometer Frequency 150.93
Spectral Width 36057.7
Lowest Frequency -2938.7
Nucleus 13C
Acquired Size 32768
Spectral Size 65536
172.13
151.22 151.14 149.59 149.51 149.21 149.12 147.55 147.46
129.86 129.81 125.94 125.91 125.89 125.86 124.12 118.53 118.42
56.52 53.07 43.17 42.16 37.90 32.23
f1 (ppm)

AL485 19F.1.fid

Parameter	Value
Data File Name	C:/ Users/ anvi0182/ Desktop/ NMR/ AL485 19F/ 1/ fid
Title	AL485 19F.1.fid
Origin	Bruker BioSpin GmbH
Owner	nmr
Instrument	spect
Solvent	DMSO
Temperature	298.0
Pulse Sequence	zgfhigqn.2
Experiment	1D
Probe	Z116098_0046 (PA BBO 400S1 BBF-H-D-05 Z SP)
Number of Scans	16
Receiver Gain	191.2
Relaxation Delay	1.0000
Pulse Width	15.5000
Presaturation Frequency	
Acquisition Time	0.7340
Acquisition Date	2020-07-08T13:01:54
Modification Date	2020-07-08T13:01:54
Class	
Spectrometer Frequency	375.96
Spectral Width	89285.7
Lowest Frequency	-82242.9
Nucleus	19F
Acquired Size	65536
Spectral Size	131072

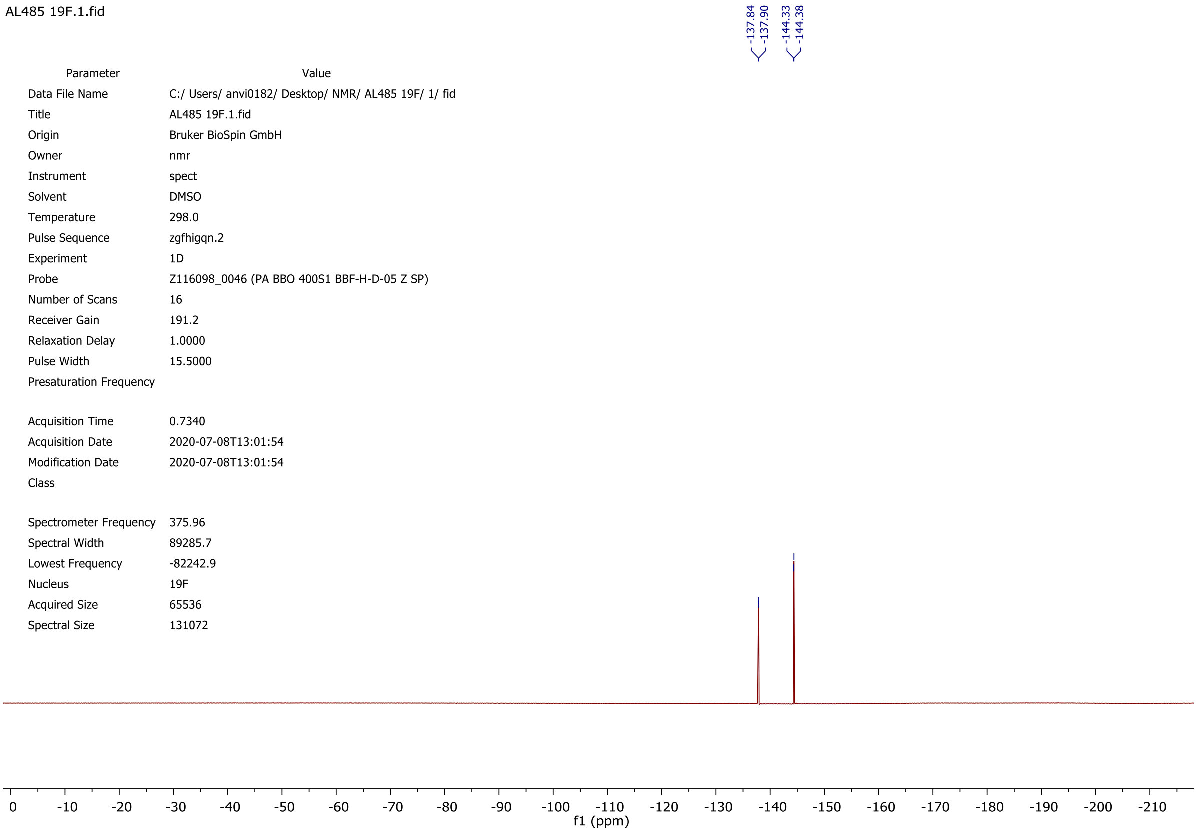

40

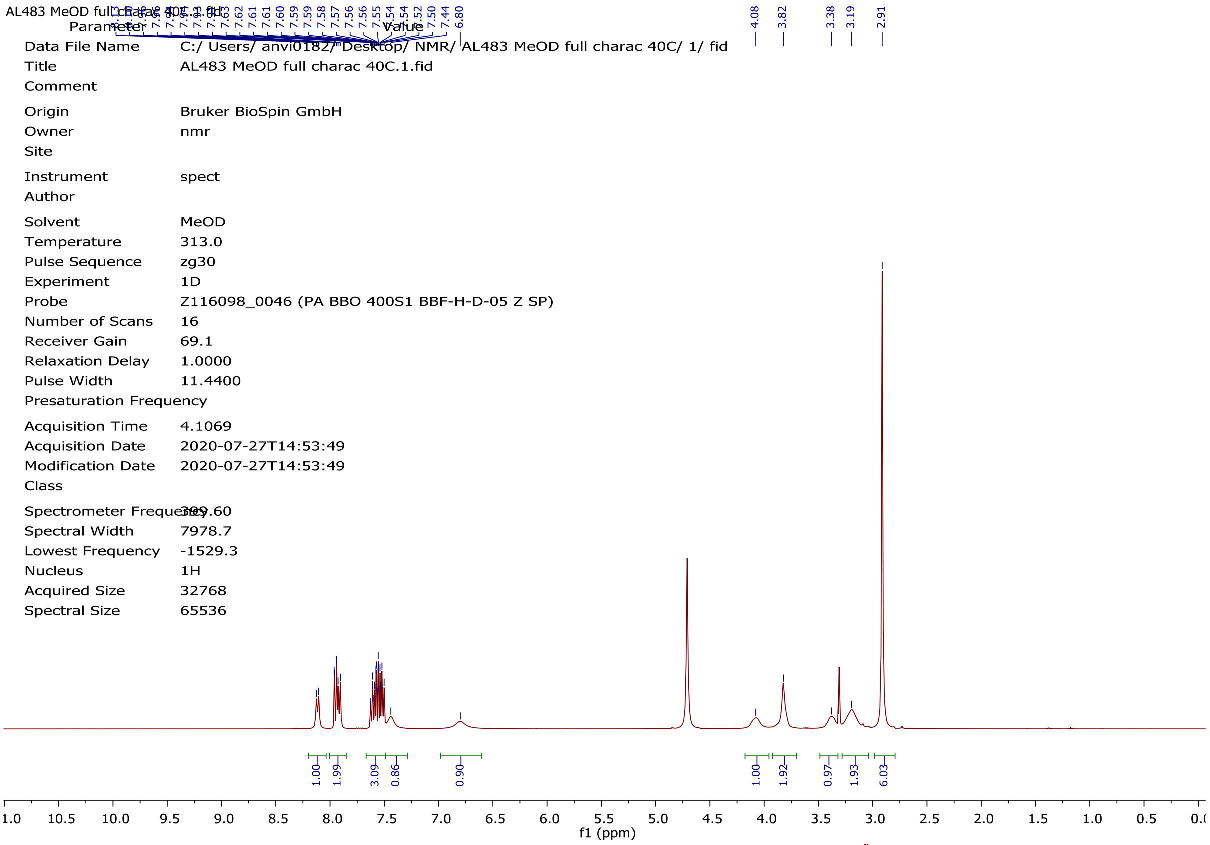

AL483 MeOD full charac 40C.1.fid
7.63 7.62 7.61 7.61 7.60 7.59 7.59 7.58 7.57 7.56 7.56 7.55 7.54 7.54 7.52 7.50 7.44 6.80 4.08 3.82 3.38 3.19 2.91
Parameter Value
Data File Name C:/ Users/ anvi0182/ Desktop/ NMR/ AL483 MeOD full charac 40C/ 1/ fid
Title AL483 MeOD full charac 40C.1.fid
Comment
Origin Bruker BioSpin GmbH
Owner nmr
Site
Instrument spect
Author
Solvent MeOD
Temperature 313.0
Pulse Sequence zg30
Experiment 1D
Probe Z116098_0046 (PA BBO 400S1 BBF-H-D-05 Z SP)
Number of Scans 16
Receiver Gain 69.1
Relaxation Delay 1.0000
Pulse Width 11.4400
Presaturation Frequency
Acquisition Time 4.1069
Acquisition Date 2020-07-27T14:53:49
Modification Date 2020-07-27T14:53:49
Class
Spectrometer Frequency 399.60
Spectral Width 7978.7
Lowest Frequency -1529.3
Nucleus 1H
Acquired Size 32768
Spectral Size 65536
1.00 1.99 3.09 0.86 0.90 1.00 1.92 0.97 1.93 6.03
11.0 10.5 10.0 9.5 9.0 8.5 8.0 7.5 7.0 6.5 6.0 5.5 5.0 4.5 4.0 3.5 3.0 2.5 2.0 1.5 1.0 0.5 0.0
f1 (ppm)

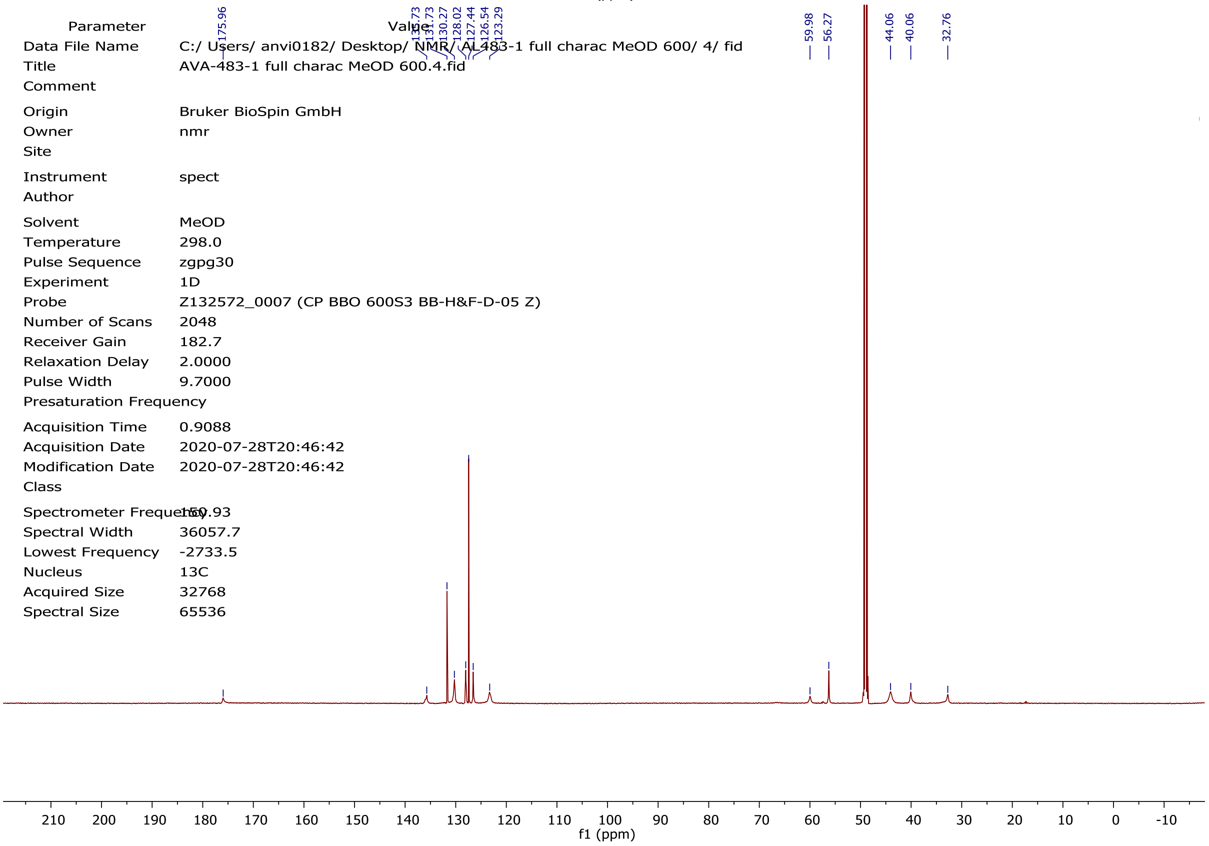

175.96 135.73 131.73 130.27 128.02 127.44 126.54 123.29 59.98 56.27 44.06 40.06 32.76
Parameter Value
Data File Name C:/ Users/ anvi0182/ Desktop/ NMR/ AL483-1 full charac MeOD 600/ 4/ fid
Title AVA-483-1 full charac MeOD 600.4.fid
Comment
Origin Bruker BioSpin GmbH
Owner nmr
Site
Instrument spect
Author
Solvent MeOD
Temperature 298.0
Pulse Sequence zgpg30
Experiment 1D
Probe Z132572_0007 (CP BBO 600S3 BB-H&F-D-05 Z)
Number of Scans 2048
Receiver Gain 182.7
Relaxation Delay 2.0000
Pulse Width 9.7000
Presaturation Frequency
Acquisition Time 0.9088
Acquisition Date 2020-07-28T20:46:42
Modification Date 2020-07-28T20:46:42
Class
Spectrometer Frequency 150.93
Spectral Width 36057.7
Lowest Frequency -2733.5
Nucleus 13C
Acquired Size 32768
Spectral Size 65536
210 200 190 180 170 160 150 140 130 120 110 100 90 80 70 60 50 40 30 20 10 0 -10
f1 (ppm)

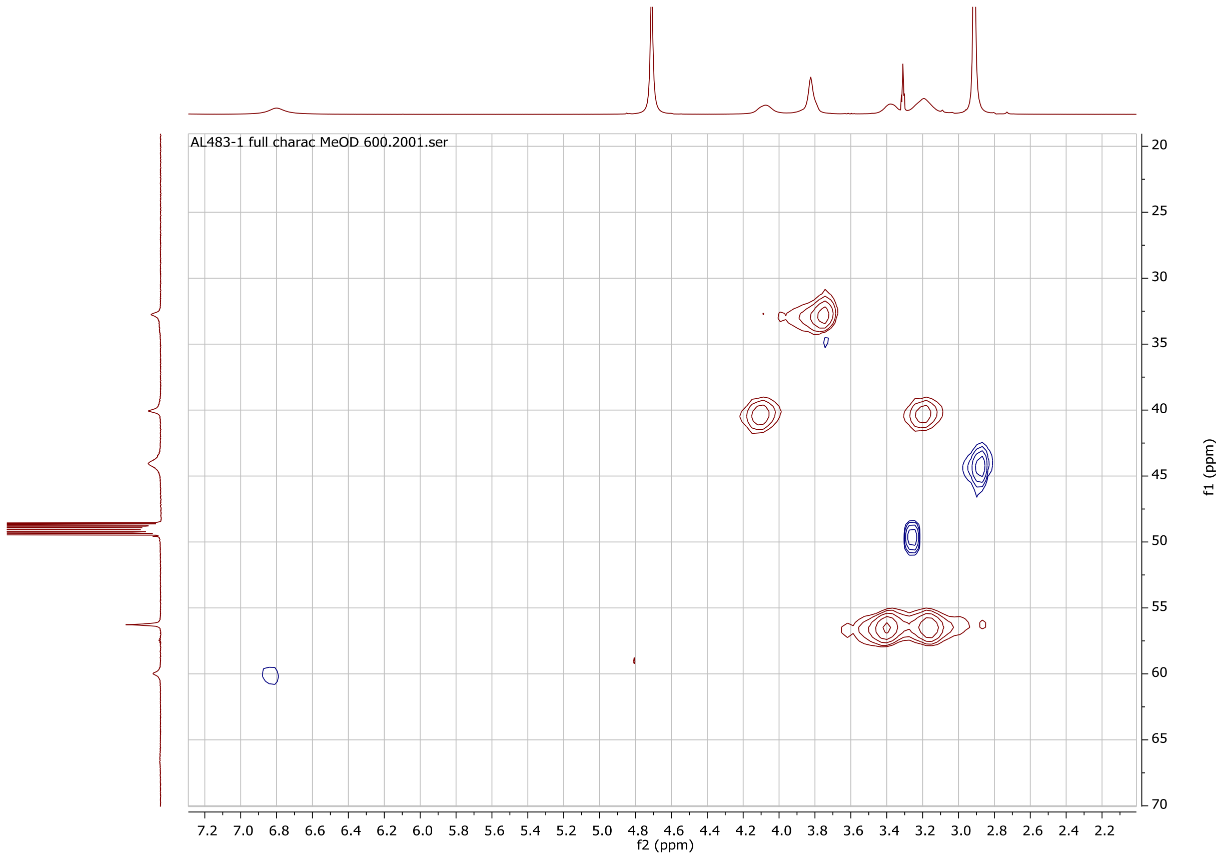
AL483-1 full charac MeOD 600.2001.ser
f2 (ppm)
f1 (ppm)

41

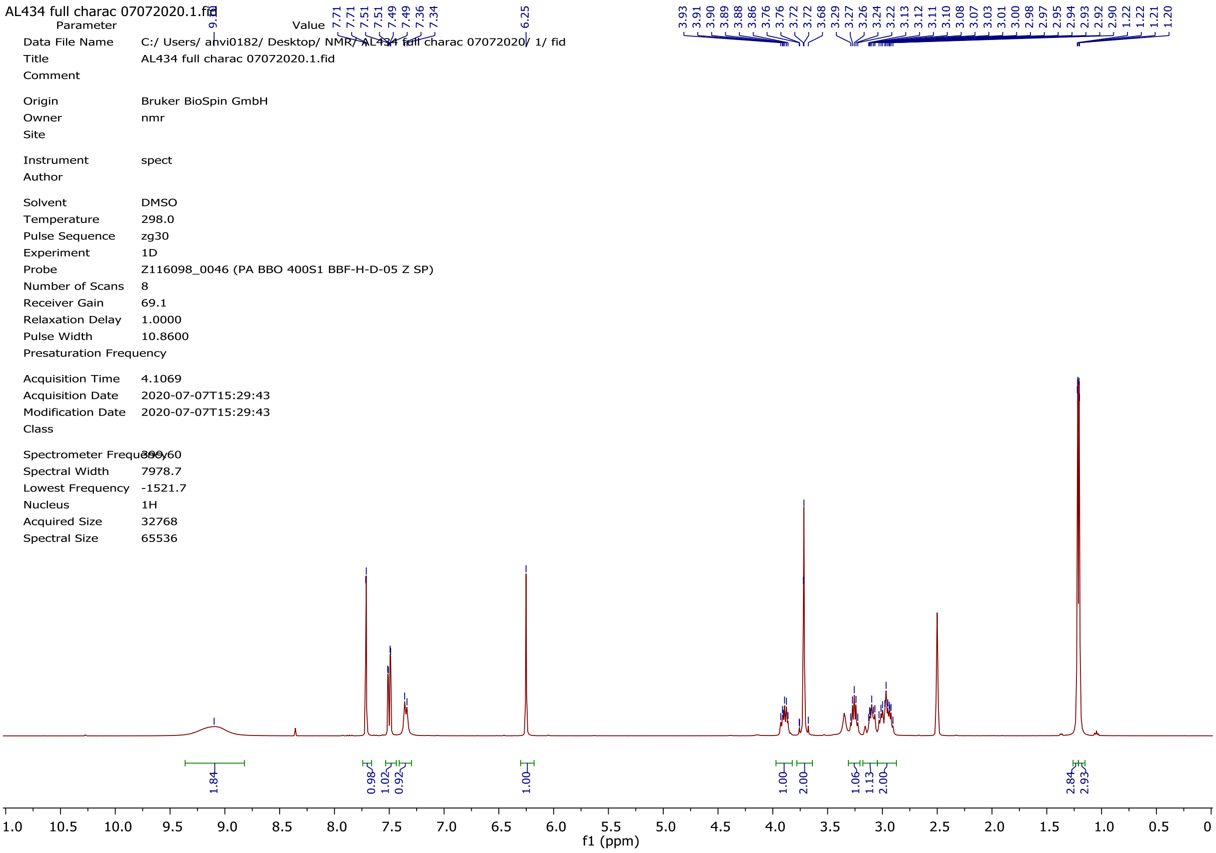
AL434 full charac 07072020.1.fid
Parameter Value
Data File Name C:/ Users/ anvi0182/ Desktop/ NMR/ AL434 full charac 07072020/ 1/ fid
Title AL434 full charac 07072020.1.fid
Comment
Origin Bruker BioSpin GmbH
Owner nmr
Site
Instrument spect
Author
Solvent DMSO
Temperature 298.0
Pulse Sequence zg30
Experiment 1D
Probe Z116098_0046 (PA BBO 400S1 BBF-H-D-05 Z SP)
Number of Scans 8
Receiver Gain 69.1
Relaxation Delay 1.0000
Pulse Width 10.8600
Presaturation Frequency
Acquisition Time 4.1069
Acquisition Date 2020-07-07T15:29:43
Modification Date 2020-07-07T15:29:43
Class
Spectrometer Frequency 399.60
Spectral Width 7978.7
Lowest Frequency -1521.7
Nucleus 1H
Acquired Size 32768
Spectral Size 65536
f1 (ppm)

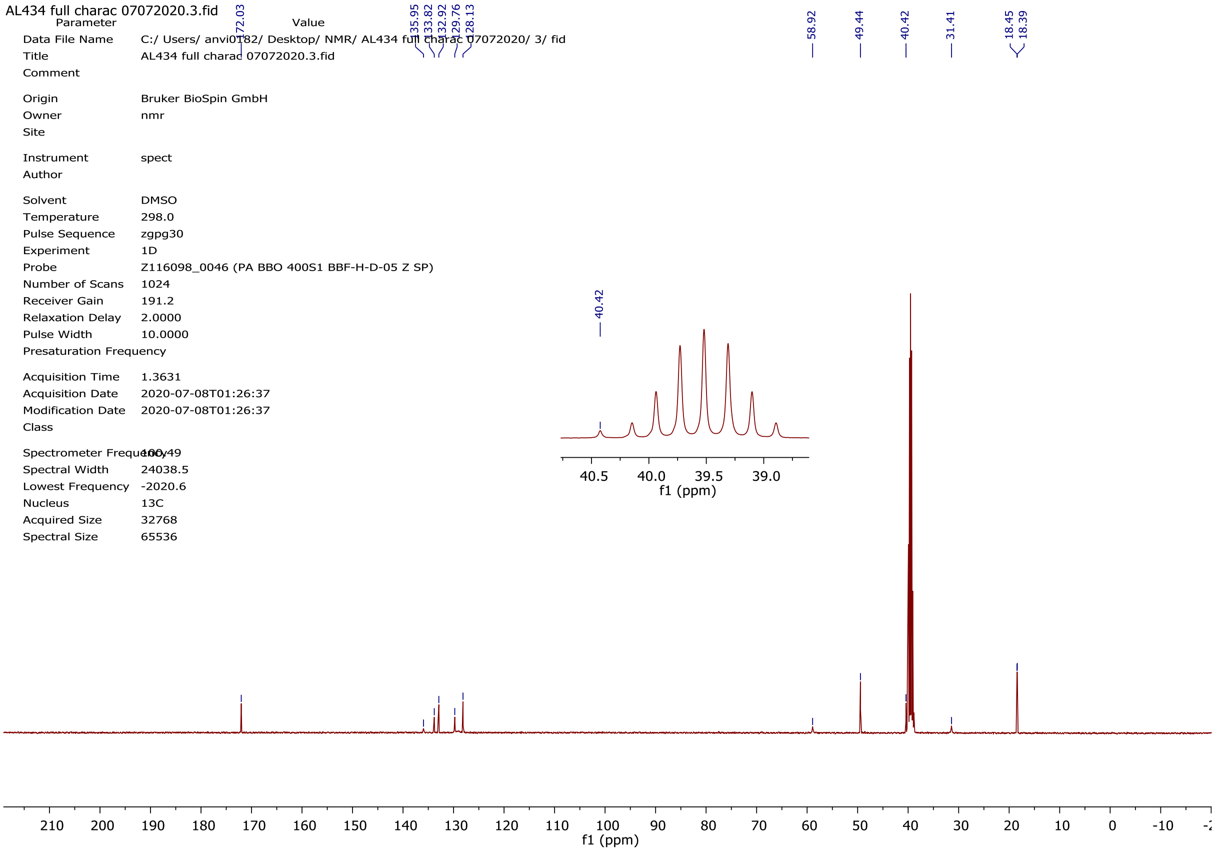
AL434 full charac 07072020.3.fid
Parameter Value
Data File Name C:/ Users/ anvi0182/ Desktop/ NMR/ AL434 full charac 07072020/ 3/ fid
Title AL434 full charac 07072020.3.fid
Comment
Origin Bruker BioSpin GmbH
Owner nmr
Site
Instrument spect
Author
Solvent DMSO
Temperature 298.0
Pulse Sequence zgpg30
Experiment 1D
Probe Z116098_0046 (PA BBO 400S1 BBF-H-D-05 Z SP)
Number of Scans 1024
Receiver Gain 191.2
Relaxation Delay 2.0000
Pulse Width 10.0000
Presaturation Frequency
Acquisition Time 1.3631
Acquisition Date 2020-07-08T01:26:37
Modification Date 2020-07-08T01:26:37
Class
Spectrometer Frequency 100.49
Spectral Width 24038.5
Lowest Frequency -2020.6
Nucleus 13C
Acquired Size 32768
Spectral Size 65536
172.03
135.95
133.82
132.92
129.76
128.13
58.92
49.44
40.42
31.41
18.45
18.39
f1 (ppm)

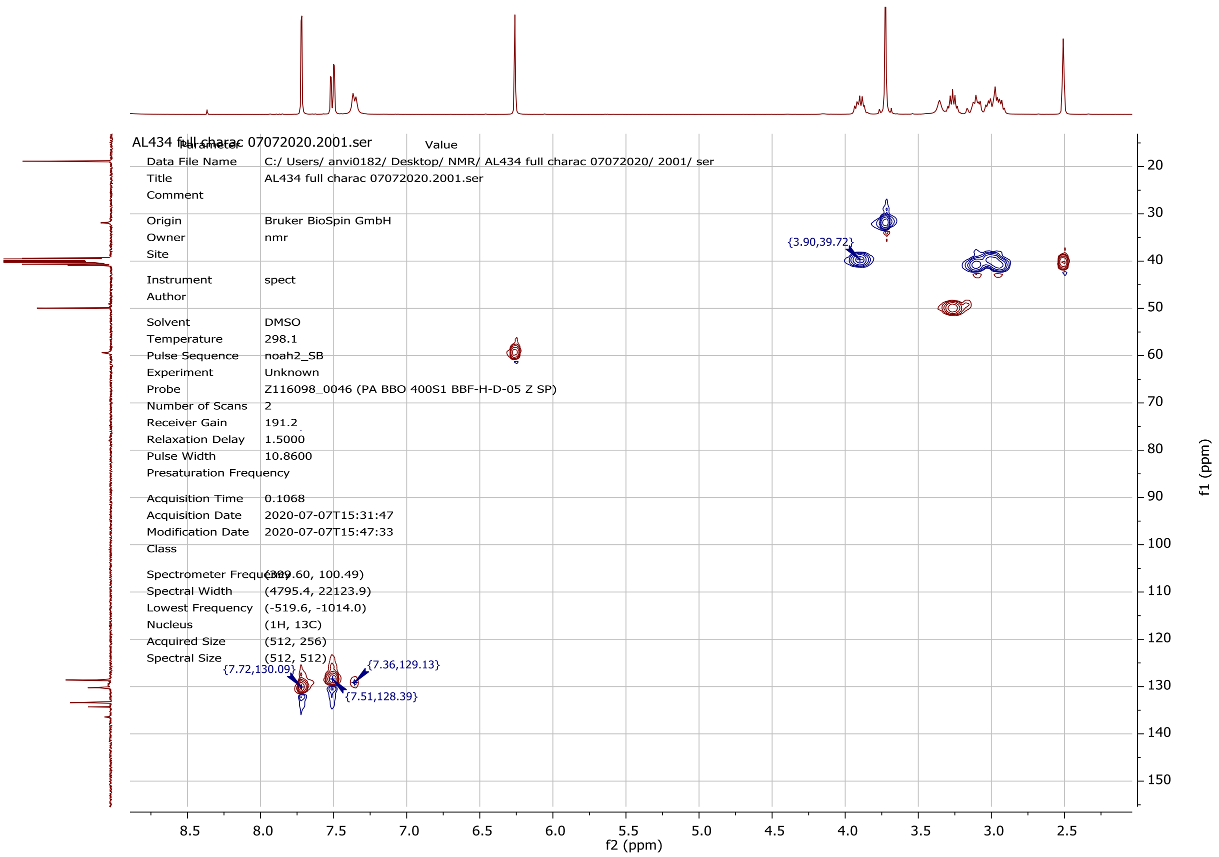
AL434 full charac 07072020.2001.ser
Parameter
Value
Data File Name
C:/ Users/ anvi0182/ Desktop/ NMR/ AL434 full charac 07072020/ 2001/ ser
Title
AL434 full charac 07072020.2001.ser
Comment
Origin
Bruker BioSpin GmbH
Owner
nmr
Site
Instrument
spect
Author
Solvent
DMSO
Temperature
298.1
Pulse Sequence
noah2_SB
Experiment
Unknown
Probe
Z116098_0046 (PA BBO 400S1 BBF-H-D-05 Z SP)
Number of Scans
2
Receiver Gain
191.2
Relaxation Delay
1.5000
Pulse Width
10.8600
Presaturation Frequency
Acquisition Time
0.1068
Acquisition Date
2020-07-07T15:31:47
Modification Date
2020-07-07T15:47:33
Class
Spectrometer Frequency
(399.60, 100.49)
Spectral Width
(4795.4, 22123.9)
Lowest Frequency
(-519.6, -1014.0)
Nucleus
(1H, 13C)
Acquired Size
(512, 256)
Spectral Size
(512, 512)
{3.90,39.72}
{7.72,130.09}
{7.36,129.13}
{7.51,128.39}
f2 (ppm)
f1 (ppm)

42

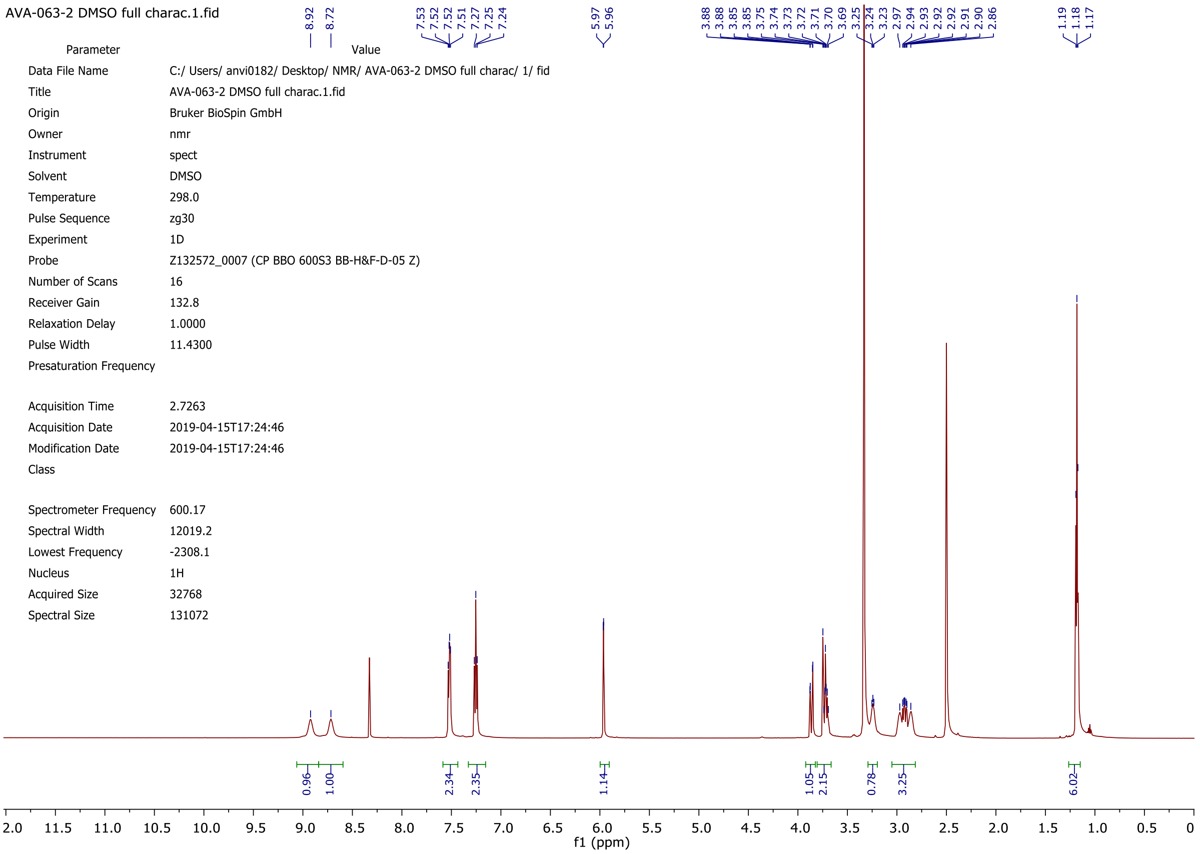

Parameter	Value
Data File Name	C:/ Users/ anvi0182/ Desktop/ NMR/ AVA-063-2 DMSO full charac/ 1/ fid
Title	AVA-063-2 DMSO full charac.1.fid
Origin	Bruker BioSpin GmbH
Owner	nmr
Instrument	spect
Solvent	DMSO
Temperature	298.0
Pulse Sequence	zg30
Experiment	1D
Probe	Z132572_0007 (CP BBO 600S3 BB-H&F-D-05 Z)
Number of Scans	16
Receiver Gain	132.8
Relaxation Delay	1.0000
Pulse Width	11.4300
Presaturation Frequency	
Acquisition Time	2.7263
Acquisition Date	2019-04-15T17:24:46
Modification Date	2019-04-15T17:24:46
Class	
Spectrometer Frequency	600.17
Spectral Width	12019.2
Lowest Frequency	-2308.1
Nucleus	1H
Acquired Size	32768
Spectral Size	131072

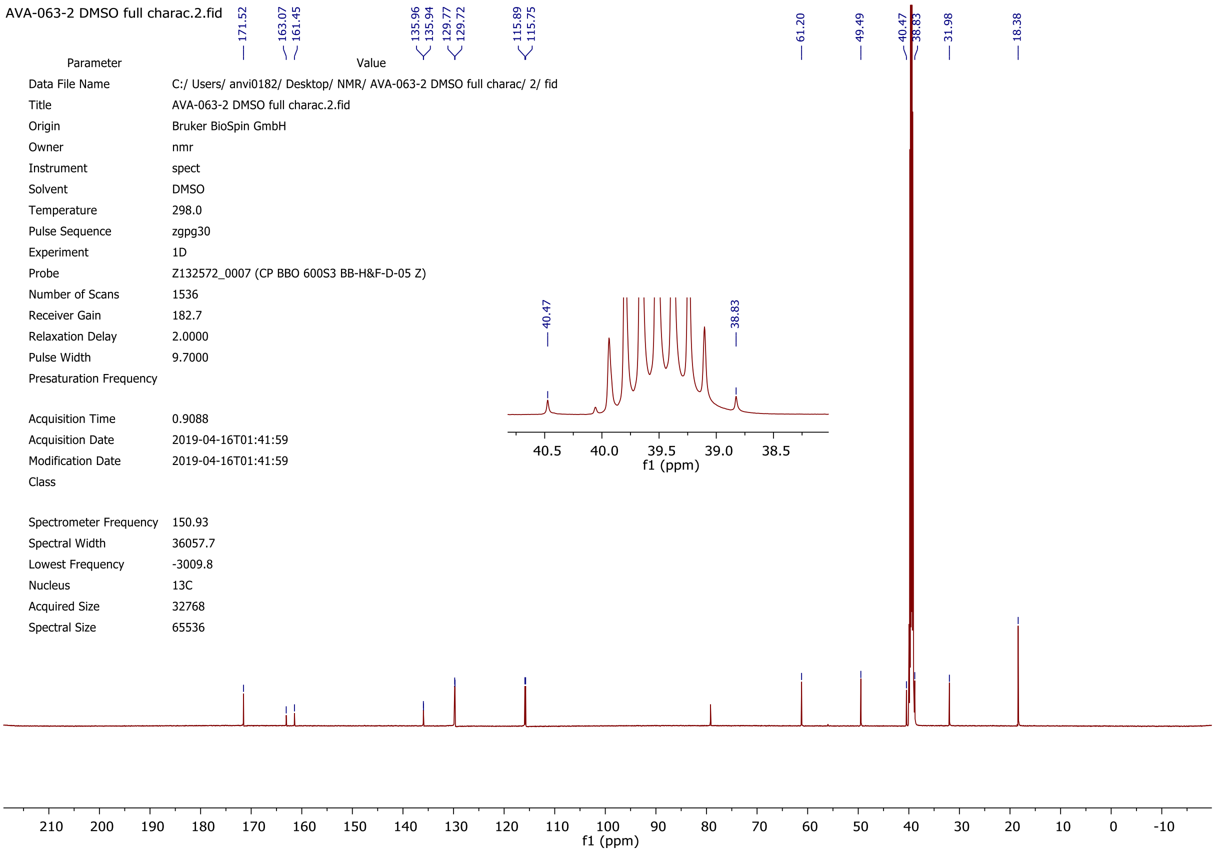

Parameter	Value
Data File Name	C:/ Users/ anvi0182/ Desktop/ NMR/ AVA-063-2 DMSO full charac/ 2/ fid
Title	AVA-063-2 DMSO full charac.2.fid
Origin	Bruker BioSpin GmbH
Owner	nmr
Instrument	spect
Solvent	DMSO
Temperature	298.0
Pulse Sequence	zgpg30
Experiment	1D
Probe	Z132572_0007 (CP BBO 600S3 BB-H&F-D-05 Z)
Number of Scans	1536
Receiver Gain	182.7
Relaxation Delay	2.0000
Pulse Width	9.7000
Presaturation Frequency	
Acquisition Time	0.9088
Acquisition Date	2019-04-16T01:41:59
Modification Date	2019-04-16T01:41:59
Class	
Spectrometer Frequency	150.93
Spectral Width	36057.7
Lowest Frequency	-3009.8
Nucleus	13C
Acquired Size	32768
Spectral Size	65536

AL449 19F and 13C DEPT.1.fid

Parameter	Value
Data File Name	C:/ Users/ anvi0182/ Desktop/ NMR/ AL449 19F and 13C DEPT/ 1/ fid
Title	AL449 19F and 13C DEPT.1.fid
Comment	
Origin	Bruker BioSpin GmbH
Owner	nmr
Site	
Instrument	spect
Author	
Solvent	DMSO
Temperature	298.0
Pulse Sequence	zgfhigqn.2
Experiment	1D
Probe	Z116098_0046 (PA BBO 400S1 BBF-H-D-05 Z SP)
Number of Scans	16
Receiver Gain	191.2
Relaxation Delay	1.0000
Pulse Width	15.5000
Presaturation Frequency	
Acquisition Time	0.7340
Acquisition Date	2020-07-08T20:32:09
Modification Date	2020-07-08T20:32:09
Class	
Spectrometer Frequency	376.96
Spectral Width	89285.7
Lowest Frequency	-82242.9
Nucleus	19F
Acquired Size	65536
Spectral Size	131072

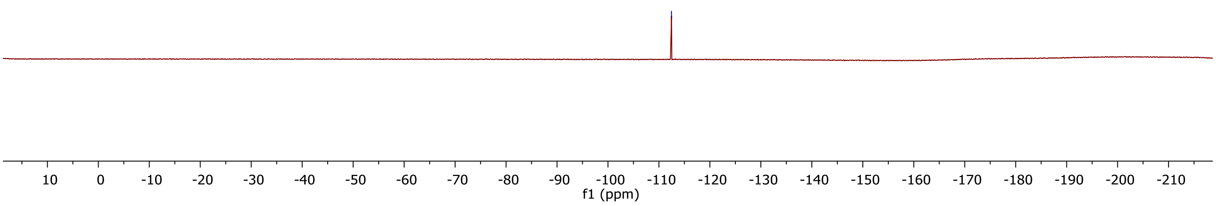

43

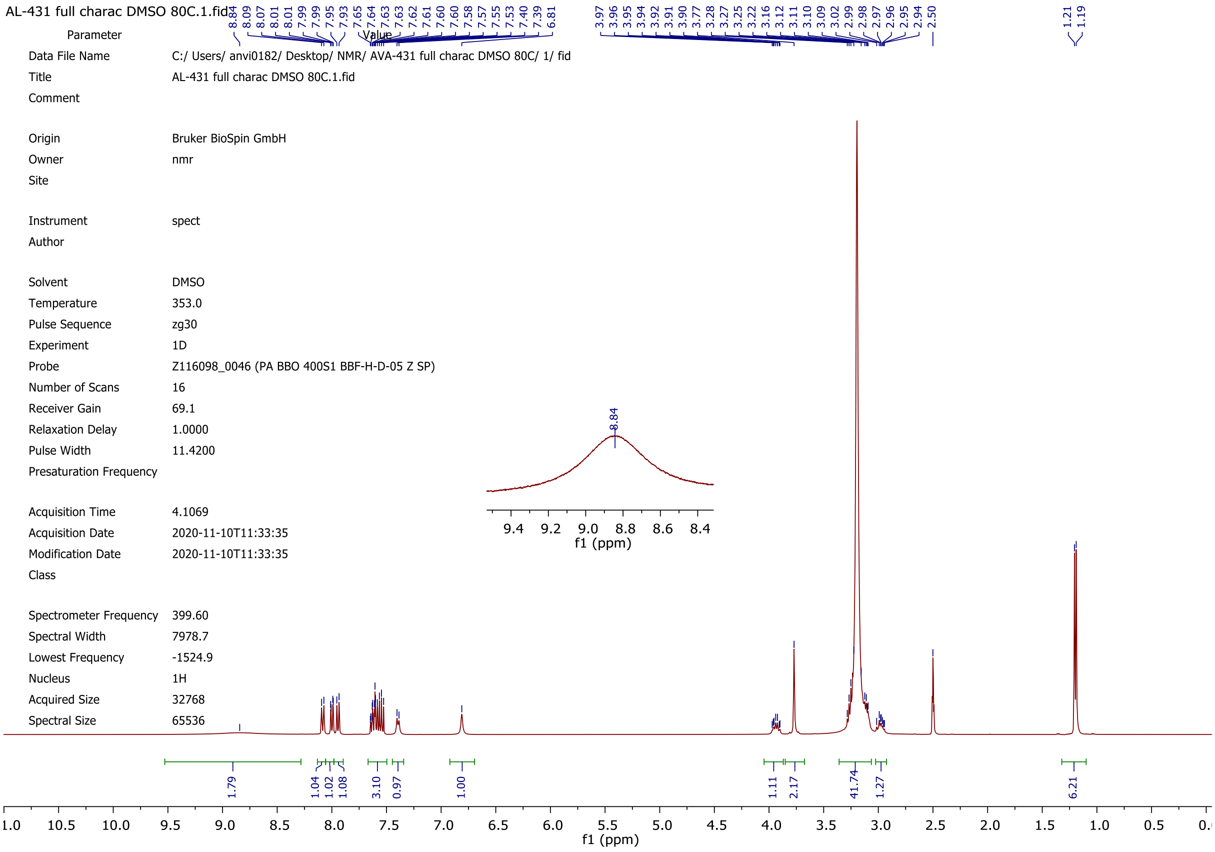
AL-431 full charac DMSO 80C.1.fid
8.84 8.09 8.07 8.01 8.01 7.99 7.95 7.93 7.65 7.64 7.63 7.63 7.62 7.61 7.60 7.60 7.58 7.57 7.55 7.53 7.40 7.39 6.81
3.97 3.96 3.95 3.94 3.92 3.91 3.90 3.77 3.28 3.27 3.25 3.22 3.16 3.12 3.11 3.10 3.09 3.02 2.99 2.98 2.97 2.96 2.95 2.94 2.50
1.21 1.19
Parameter
Value
Data File Name
C:/ Users/ anvi0182/ Desktop/ NMR/ AVA-431 full charac DMSO 80C/ 1/ fid
Title
AL-431 full charac DMSO 80C.1.fid
Comment
Origin
Bruker BioSpin GmbH
Owner
nmr
Site
Instrument
spect
Author
Solvent
DMSO
Temperature
353.0
Pulse Sequence
zg30
Experiment
1D
Probe
Z116098_0046 (PA BBO 400S1 BBF-H-D-05 Z SP)
Number of Scans
16
Receiver Gain
69.1
Relaxation Delay
1.0000
Pulse Width
11.4200
Presaturation Frequency
Acquisition Time
4.1069
Acquisition Date
2020-11-10T11:33:35
Modification Date
2020-11-10T11:33:35
Class
Spectrometer Frequency
399.60
Spectral Width
7978.7
Lowest Frequency
-1524.9
Nucleus
1H
Acquired Size
32768
Spectral Size
65536
8.84
9.4 9.2 9.0 8.8 8.6 8.4
f1 (ppm)
1.79 1.04 1.02 1.08 3.10 0.97 1.00 1.11 2.17 41.74 1.27 6.21
1.0 10.5 10.0 9.5 9.0 8.5 8.0 7.5 7.0 6.5 6.0 5.5 5.0 4.5 4.0 3.5 3.0 2.5 2.0 1.5 1.0 0.5 0.
f1 (ppm)

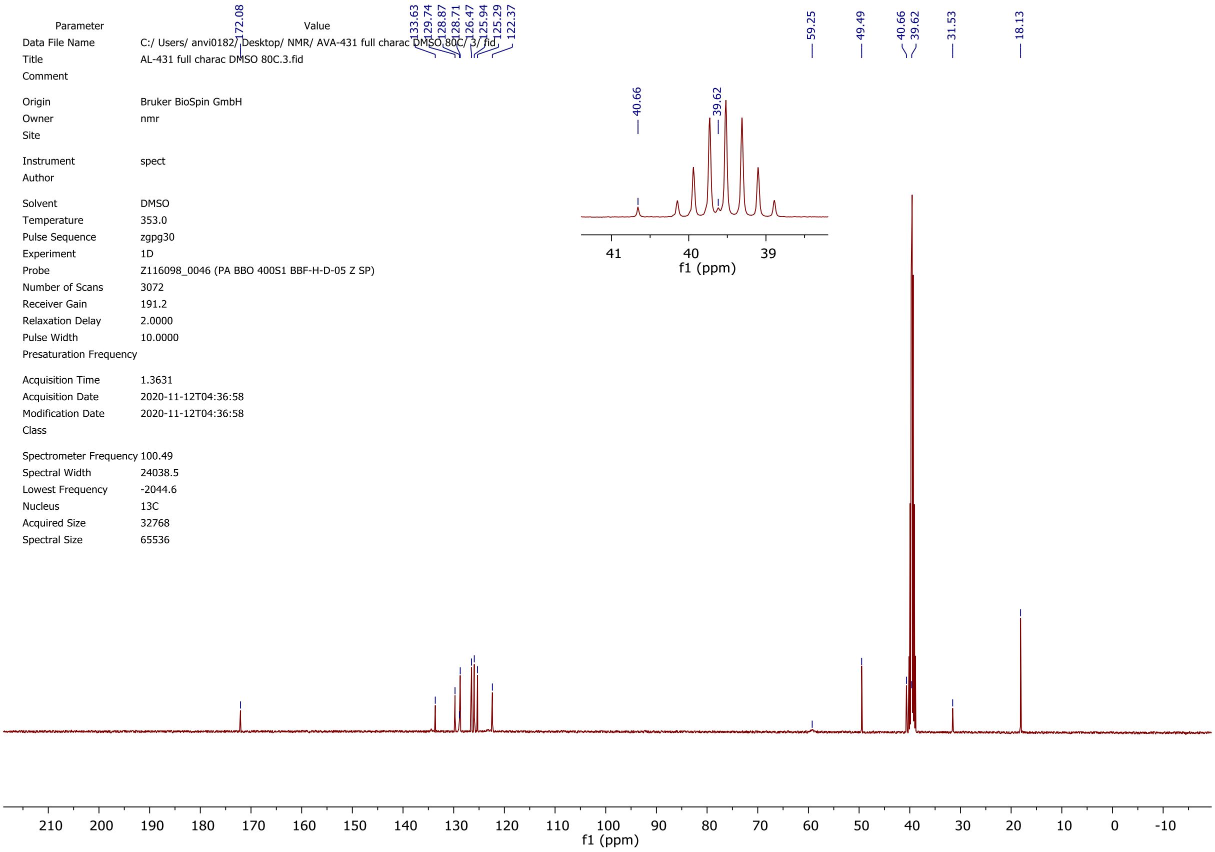
172.08
133.63 129.74 128.87 128.71 126.47 125.94 125.29 122.37
59.25 49.49 40.66 39.62 31.53 18.13
Parameter
Value
Data File Name
C:/ Users/ anvi0182/ Desktop/ NMR/ AVA-431 full charac DMSO 80C/ 3/ fid
Title
AL-431 full charac DMSO 80C.3.fid
Comment
Origin
Bruker BioSpin GmbH
Owner
nmr
Site
Instrument
spect
Author
Solvent
DMSO
Temperature
353.0
Pulse Sequence
zgpg30
Experiment
1D
Probe
Z116098_0046 (PA BBO 400S1 BBF-H-D-05 Z SP)
Number of Scans
3072
Receiver Gain
191.2
Relaxation Delay
2.0000
Pulse Width
10.0000
Presaturation Frequency
Acquisition Time
1.3631
Acquisition Date
2020-11-12T04:36:58
Modification Date
2020-11-12T04:36:58
Class
Spectrometer Frequency
100.49
Spectral Width
24038.5
Lowest Frequency
-2044.6
Nucleus
13C
Acquired Size
32768
Spectral Size
65536
40.66
39.62
41 40 39
f1 (ppm)
210 200 190 180 170 160 150 140 130 120 110 100 90 80 70 60 50 40 30 20 10 0 -10
f1 (ppm)

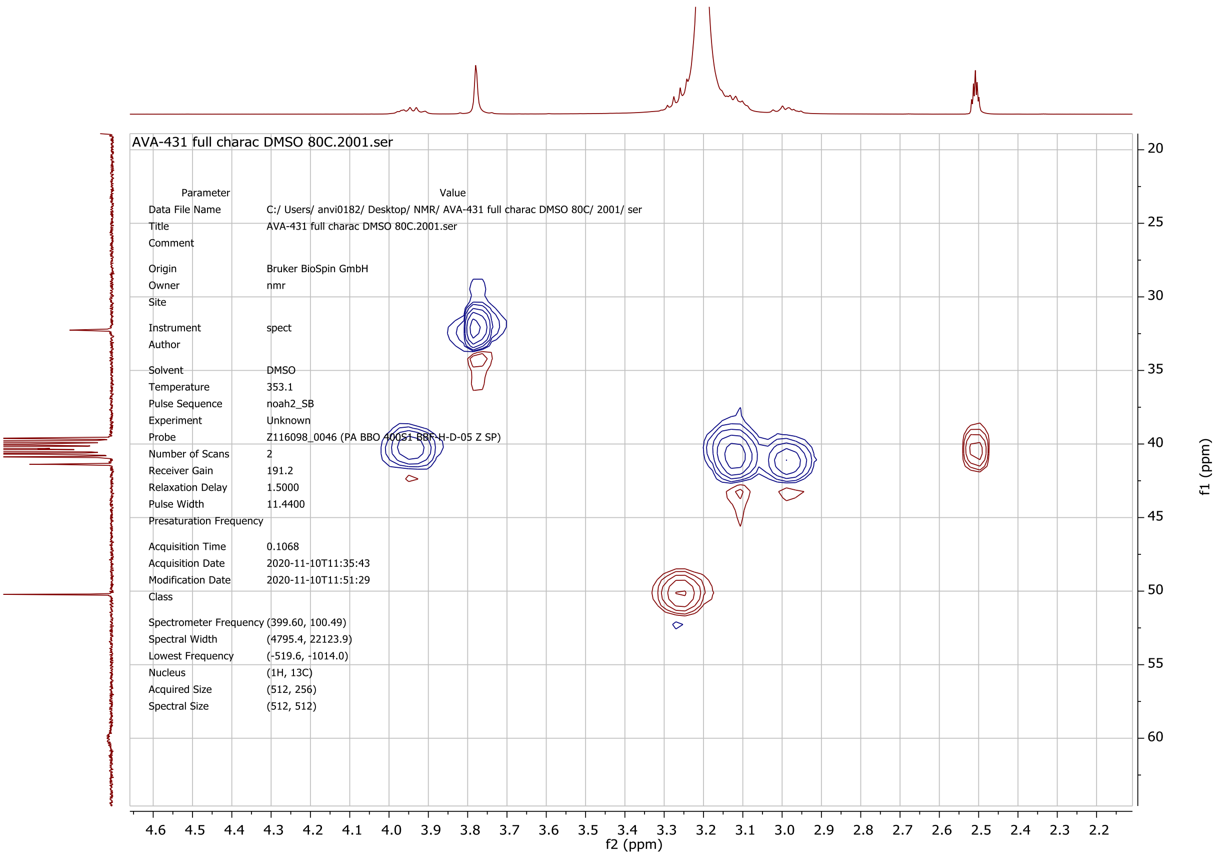

AVA-431 full charac DMSO 80C.2001.ser
Parameter
Value
Data File Name
C:/ Users/ anvi0182/ Desktop/ NMR/ AVA-431 full charac DMSO 80C/ 2001/ ser
Title
AVA-431 full charac DMSO 80C.2001.ser
Comment
Origin
Bruker BioSpin GmbH
Owner
nmr
Site
Instrument
spect
Author
Solvent
DMSO
Temperature
353.1
Pulse Sequence
noah2_SB
Experiment
Unknown
Probe
Z116098_0046 (PA BBO 400S1 BBF-H-D-05 Z SP)
Number of Scans
2
Receiver Gain
191.2
Relaxation Delay
1.5000
Pulse Width
11.4400
Presaturation Frequency
Acquisition Time
0.1068
Acquisition Date
2020-11-10T11:35:43
Modification Date
2020-11-10T11:51:29
Class
Spectrometer Frequency
(399.60, 100.49)
Spectral Width
(4795.4, 22123.9)
Lowest Frequency
(-519.6, -1014.0)
Nucleus
(1H, 13C)
Acquired Size
(512, 256)
Spectral Size
(512, 512)
f2 (ppm)
f1 (ppm)

46

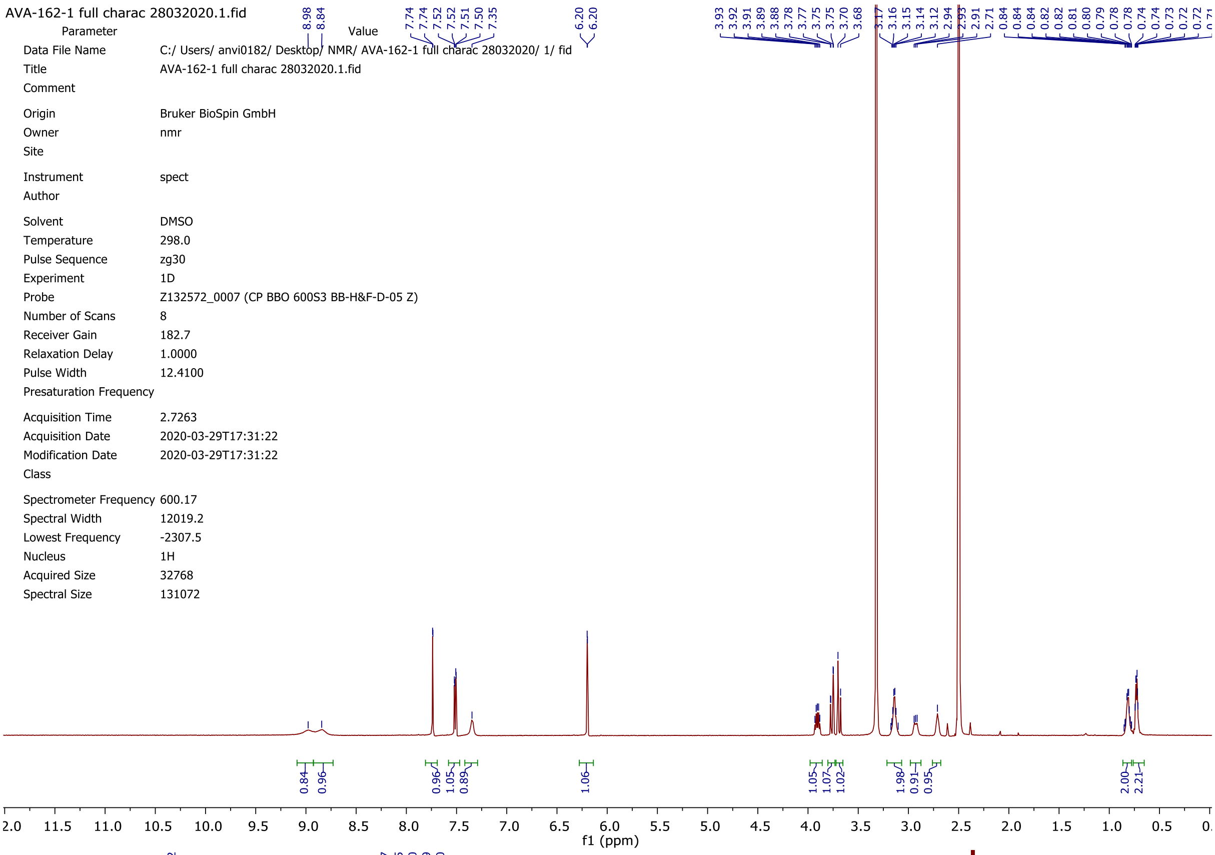

AVA-162-1 full charac 28032020.1.fid
Parameter Value
Data File Name C:/ Users/ anvi0182/ Desktop/ NMR/ AVA-162-1 full charac 28032020/ 1/ fid
Title AVA-162-1 full charac 28032020.1.fid
Comment
Origin Bruker BioSpin GmbH
Owner nmr
Site
Instrument spect
Author
Solvent DMSO
Temperature 298.0
Pulse Sequence zg30
Experiment 1D
Probe Z132572_0007 (CP BBO 600S3 BB-H&F-D-05 Z)
Number of Scans 8
Receiver Gain 182.7
Relaxation Delay 1.0000
Pulse Width 12.4100
Presaturation Frequency
Acquisition Time 2.7263
Acquisition Date 2020-03-29T17:31:22
Modification Date 2020-03-29T17:31:22
Class
Spectrometer Frequency 600.17
Spectral Width 12019.2
Lowest Frequency -2307.5
Nucleus 1H
Acquired Size 32768
Spectral Size 131072
8.98 8.84 7.74 7.74 7.52 7.52 7.51 7.50 7.35 6.20 6.20
3.93 3.92 3.91 3.89 3.88 3.78 3.77 3.75 3.75 3.70 3.68 3.17 3.16 3.15 3.14 3.12 2.94 2.93 2.91 2.71
0.84 0.84 0.82 0.82 0.81 0.80 0.79 0.78 0.78 0.74 0.74 0.73 0.72 0.72
0.84 0.96 0.96 1.05 0.89 1.06 1.05 1.07 1.02 1.98 0.91 0.95 2.00 2.21
f1 (ppm)

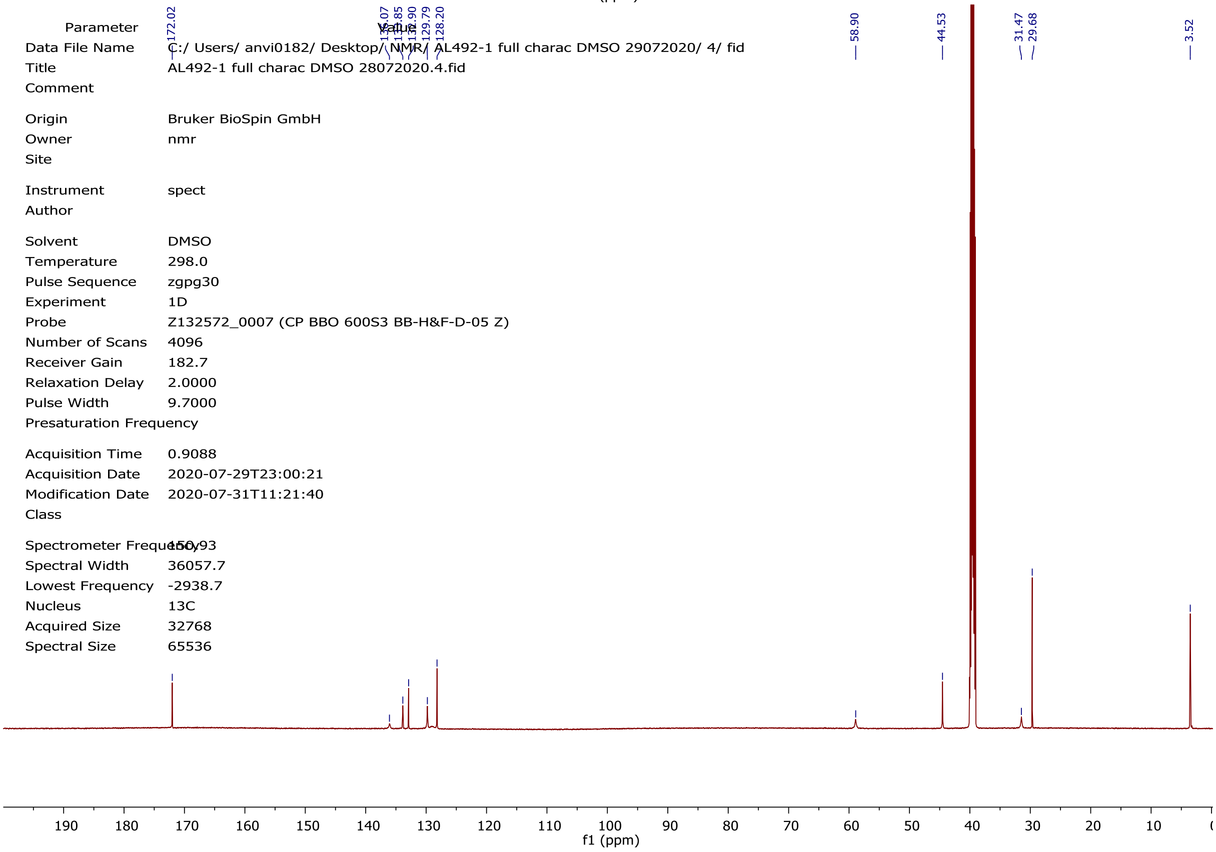

Parameter Value
Data File Name C:/ Users/ anvi0182/ Desktop/ NMR/ AL492-1 full charac DMSO 29072020/ 4/ fid
Title AL492-1 full charac DMSO 28072020.4.fid
Comment
Origin Bruker BioSpin GmbH
Owner nmr
Site
Instrument spect
Author
Solvent DMSO
Temperature 298.0
Pulse Sequence zgpg30
Experiment 1D
Probe Z132572_0007 (CP BBO 600S3 BB-H&F-D-05 Z)
Number of Scans 4096
Receiver Gain 182.7
Relaxation Delay 2.0000
Pulse Width 9.7000
Presaturation Frequency
Acquisition Time 0.9088
Acquisition Date 2020-07-29T23:00:21
Modification Date 2020-07-31T11:21:40
Class
Spectrometer Frequency 150.93
Spectral Width 36057.7
Lowest Frequency -2938.7
Nucleus 13C
Acquired Size 32768
Spectral Size 65536
172.02 129.79 128.20 58.90 44.53 31.47 29.68 3.52
f1 (ppm)

Parameter	Value
Data File Name	C:/ Users/ anvi0182/ Desktop/ NMR/ AL492-1 full charac DMSO 29072020/ 3/ fid
Title	AL492-1 full charac DMSO 28072020.3.fid
Comment	
Origin	Bruker BioSpin GmbH
Owner	nmr
Site	
Instrument	spect
Author	
Solvent	DMSO
Temperature	298.0
Pulse Sequence	deptsp135
Experiment	DEPT-135
Probe	Z132572_0007 (CP BBO 600S3 BB-H&F-D-05 Z)
Number of Scans	512
Receiver Gain	182.7
Relaxation Delay	2.0000
Pulse Width	9.7000
Presaturation Frequency	
Acquisition Time	1.3631
Acquisition Date	2020-07-29T19:35:56
Modification Date	2020-07-31T11:21:39
Class	
Spectrometer Frequency	150.92
Spectral Width	24038.5
Lowest Frequency	-52.9
Nucleus	13C
Acquired Size	32768
Spectral Size	65536

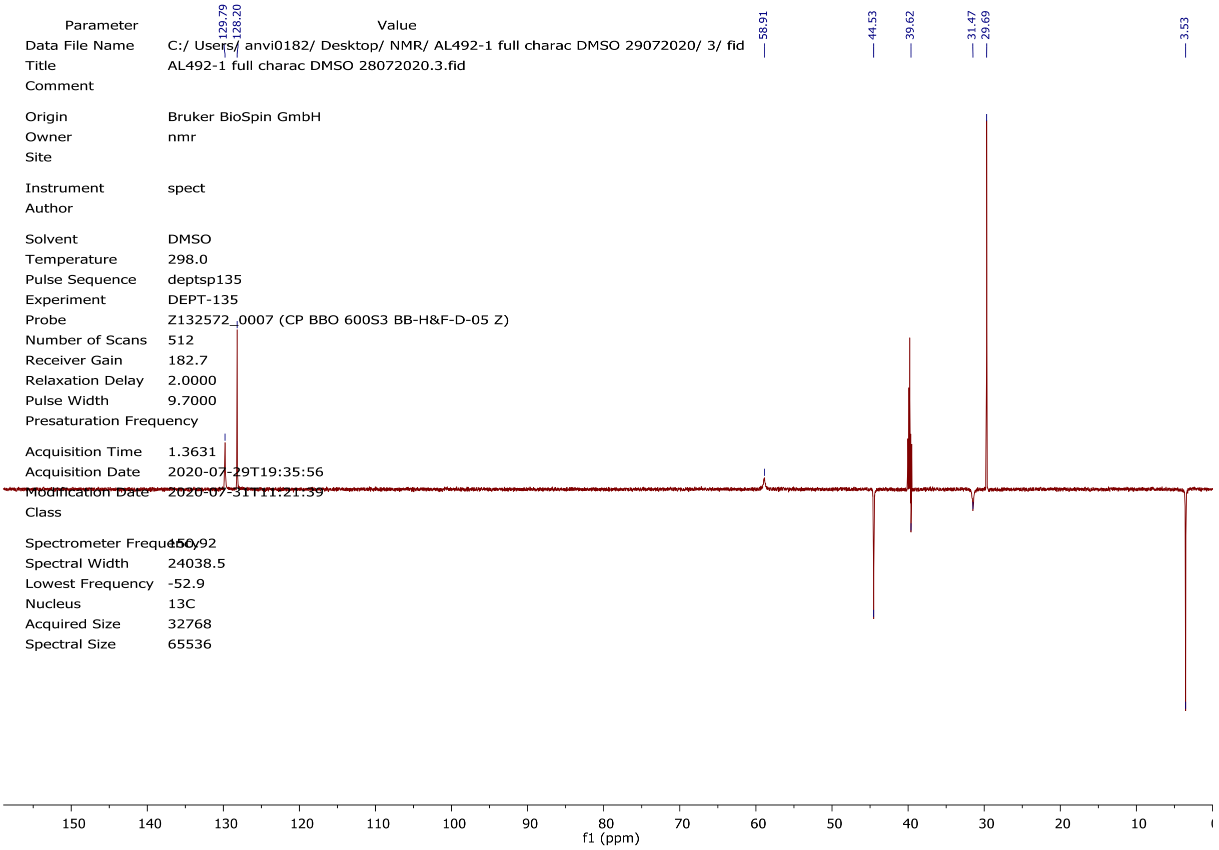

49

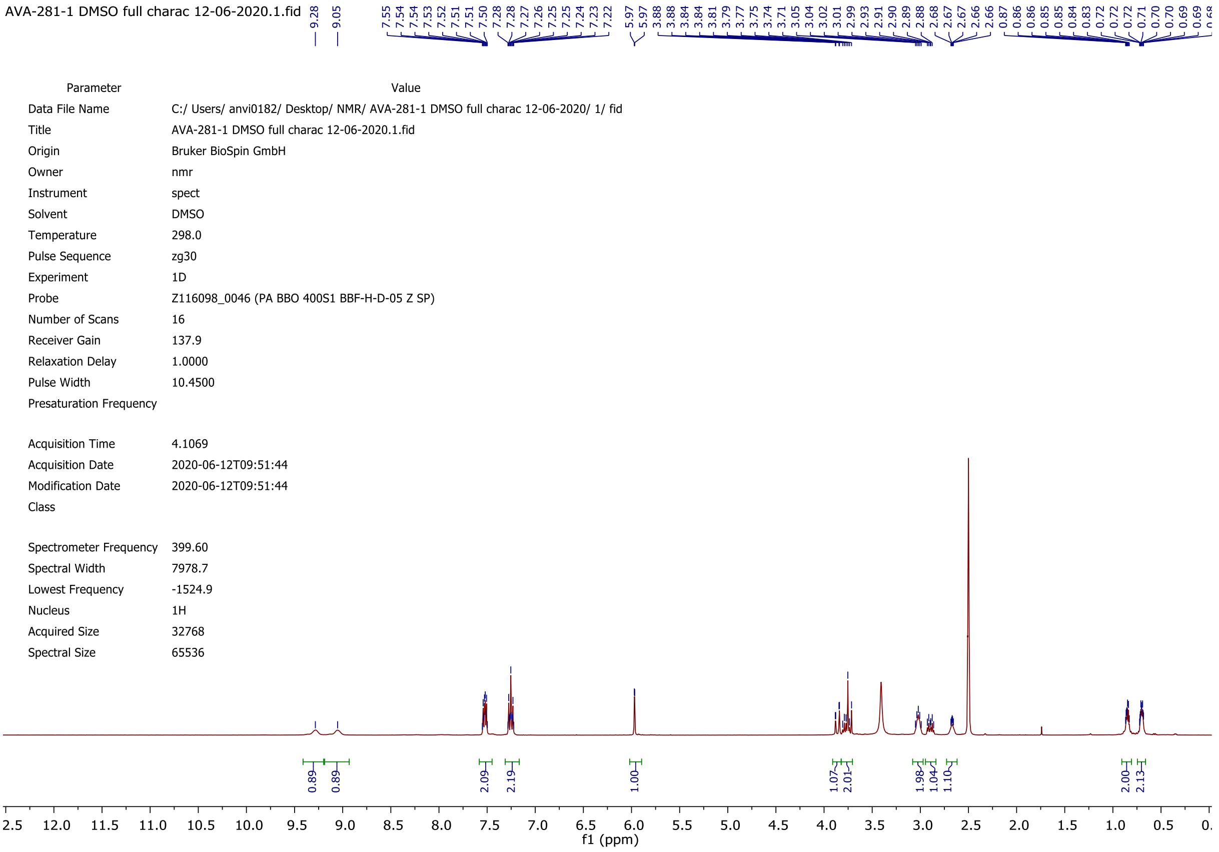
AVA-281-1 DMSO full charac 12-06-2020.1.fid
9.28
9.05
7.55 7.54 7.54 7.53 7.52 7.51 7.51 7.50 7.28 7.28 7.27 7.26 7.25 7.25 7.24 7.23 7.22
5.97 5.97
3.88 3.88 3.84 3.84 3.81 3.79 3.77 3.75 3.74 3.71 3.05 3.04 3.02 3.01 2.99 2.93 2.91 2.90 2.89 2.88 2.68 2.67 2.67 2.66
0.87 0.86 0.85 0.85 0.84 0.83 0.72 0.72 0.72 0.71 0.70 0.69 0.69
Parameter
Value
Data File Name
C:/ Users/ anvi0182/ Desktop/ NMR/ AVA-281-1 DMSO full charac 12-06-2020/ 1/ fid
Title
AVA-281-1 DMSO full charac 12-06-2020.1.fid
Origin
Bruker BioSpin GmbH
Owner
nmr
Instrument
spect
Solvent
DMSO
Temperature
298.0
Pulse Sequence
zg30
Experiment
1D
Probe
Z116098_0046 (PA BBO 400S1 BBF-H-D-05 Z SP)
Number of Scans
16
Receiver Gain
137.9
Relaxation Delay
1.0000
Pulse Width
10.4500
Presaturation Frequency
Acquisition Time
4.1069
Acquisition Date
2020-06-12T09:51:44
Modification Date
2020-06-12T09:51:44
Class
Spectrometer Frequency
399.60
Spectral Width
7978.7
Lowest Frequency
-1524.9
Nucleus
1H
Acquired Size
32768
Spectral Size
65536
0.89
0.89
2.09
2.19
1.00
1.07
2.01
1.98
1.04
1.10
2.00
2.13
f1 (ppm)

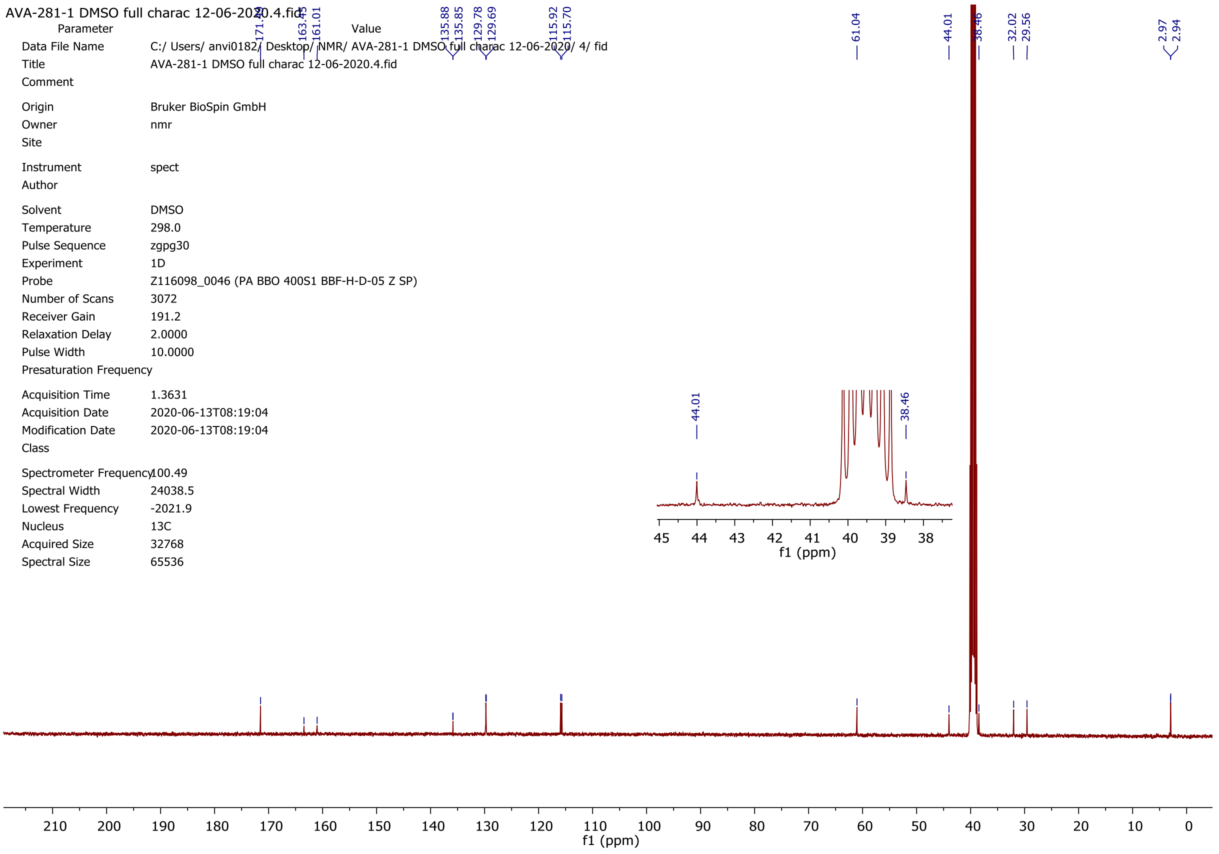
AVA-281-1 DMSO full charac 12-06-2020.4.fid
161.01
135.88 135.85
129.78 129.69
115.92 115.70
61.04
44.01
38.46
32.02
29.56
2.97 2.94
Parameter
Value
Data File Name
C:/ Users/ anvi0182/ Desktop/ NMR/ AVA-281-1 DMSO full charac 12-06-2020/ 4/ fid
Title
AVA-281-1 DMSO full charac 12-06-2020.4.fid
Comment
Origin
Bruker BioSpin GmbH
Owner
nmr
Site
Instrument
spect
Author
Solvent
DMSO
Temperature
298.0
Pulse Sequence
zgpg30
Experiment
1D
Probe
Z116098_0046 (PA BBO 400S1 BBF-H-D-05 Z SP)
Number of Scans
3072
Receiver Gain
191.2
Relaxation Delay
2.0000
Pulse Width
10.0000
Presaturation Frequency
Acquisition Time
1.3631
Acquisition Date
2020-06-13T08:19:04
Modification Date
2020-06-13T08:19:04
Class
Spectrometer Frequency
100.49
Spectral Width
24038.5
Lowest Frequency
-2021.9
Nucleus
13C
Acquired Size
32768
Spectral Size
65536
44.01
38.46
f1 (ppm)
f1 (ppm)

AVA-281-1 DMSO full charac 12-06-2020.2.fid

Parameter	Value
Data File Name	C:/ Users/ anvi0182/ Desktop/ NMR/ AVA-281-1 DMSO full charac 12-06-2020/ 2/ fid
Title	AVA-281-1 DMSO full charac 12-06-2020.2.fid
Comment	
Origin	Bruker BioSpin GmbH
Owner	nmr
Site	
Instrument	spect
Author	
Solvent	DMSO
Temperature	298.0
Pulse Sequence	zgfhigqn.2
Experiment	1D
Probe	Z116098_0046 (PA BBO 400S1 BBF-H-D-05 Z SP)
Number of Scans	16
Receiver Gain	191.2
Relaxation Delay	1.0000
Pulse Width	15.5000
Presaturation Frequency	
Acquisition Time	0.7340
Acquisition Date	2020-06-12T09:53:45
Modification Date	2020-06-12T09:53:45
Class	
Spectrometer Frequency	375.96
Spectral Width	89285.7
Lowest Frequency	-82242.9
Nucleus	19F
Acquired Size	65536
Spectral Size	131072

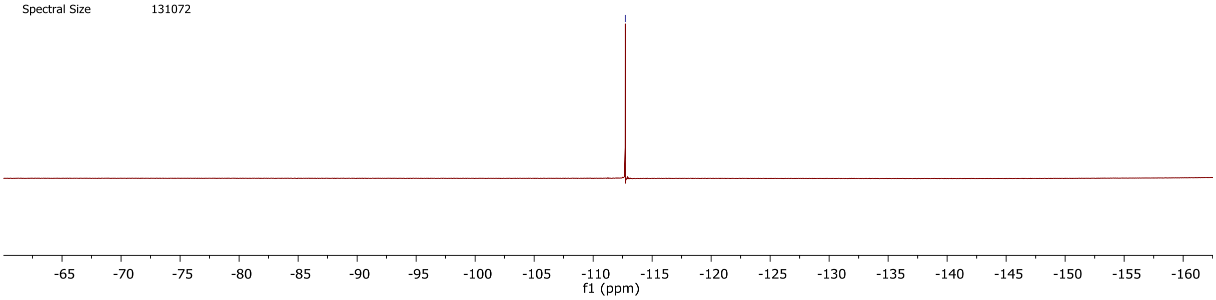

50

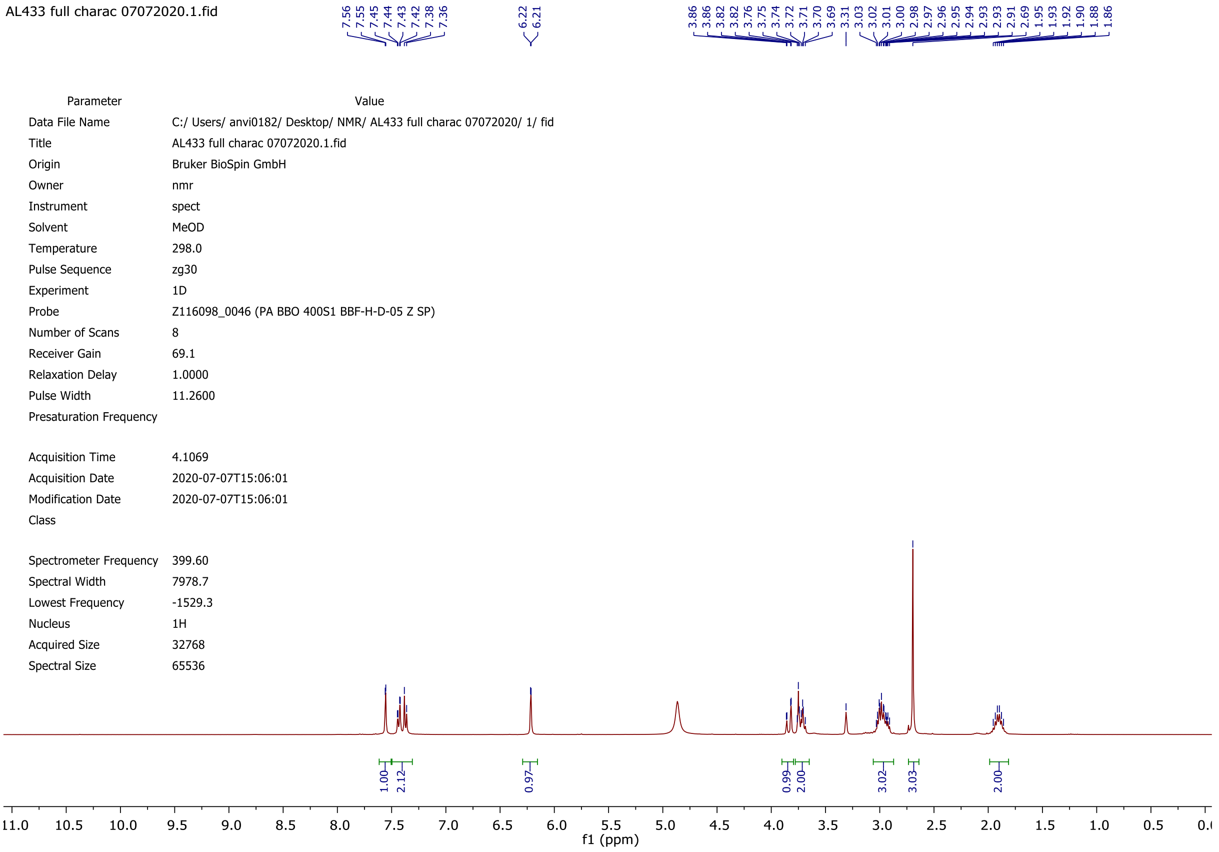

Parameter	Value
Data File Name	C:/ Users/ anvi0182/ Desktop/ NMR/ AL433 full charac 07072020/ 1/ fid
Title	AL433 full charac 07072020.1.fid
Origin	Bruker BioSpin GmbH
Owner	nmr
Instrument	spect
Solvent	MeOD
Temperature	298.0
Pulse Sequence	zg30
Experiment	1D
Probe	Z116098_0046 (PA BBO 400S1 BBF-H-D-05 Z SP)
Number of Scans	8
Receiver Gain	69.1
Relaxation Delay	1.0000
Pulse Width	11.2600
Presaturation Frequency	
Acquisition Time	4.1069
Acquisition Date	2020-07-07T15:06:01
Modification Date	2020-07-07T15:06:01
Class	
Spectrometer Frequency	399.60
Spectral Width	7978.7
Lowest Frequency	-1529.3
Nucleus	1H
Acquired Size	32768
Spectral Size	65536

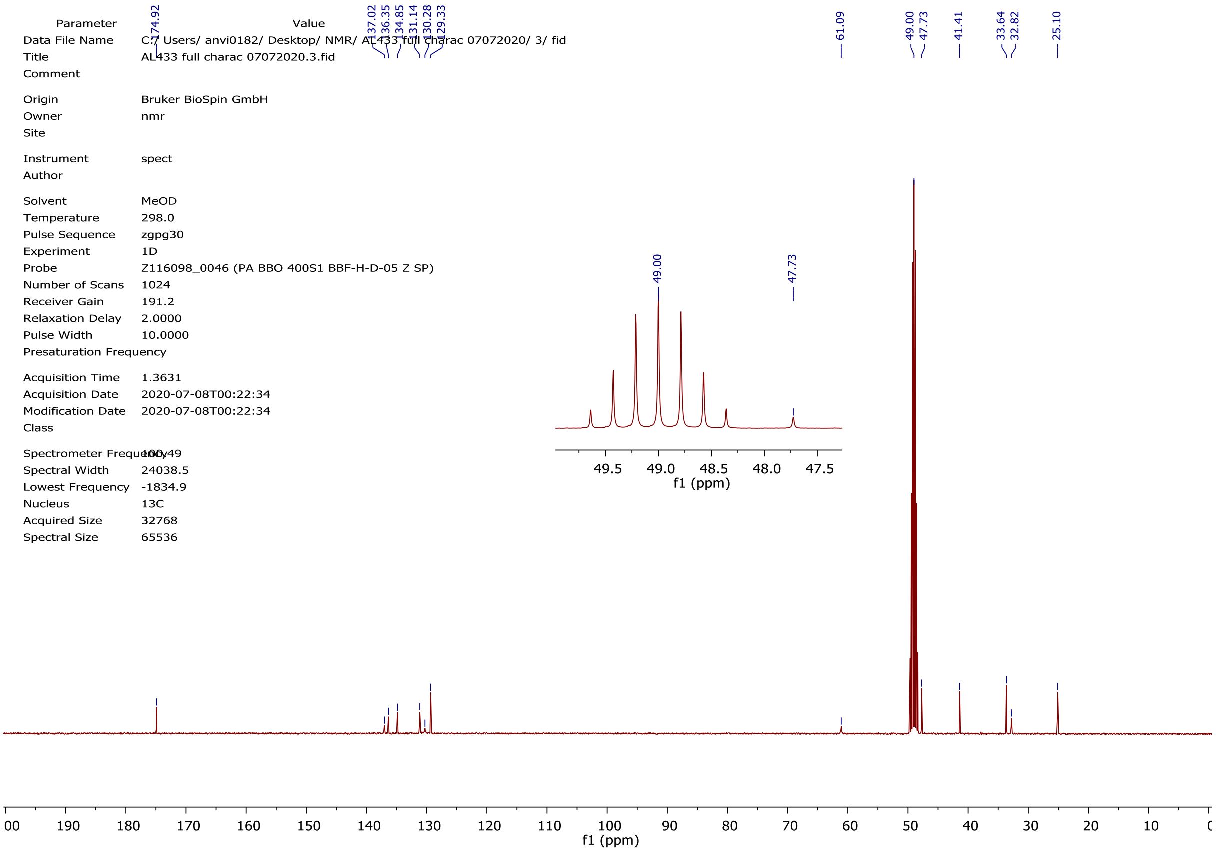

Parameter	Value
Data File Name	C:/ Users/ anvi0182/ Desktop/ NMR/ AL433 full charac 07072020/ 3/ fid
Title	AL433 full charac 07072020.3.fid
Comment	
Origin	Bruker BioSpin GmbH
Owner	nmr
Site	
Instrument	spect
Author	
Solvent	MeOD
Temperature	298.0
Pulse Sequence	zgpg30
Experiment	1D
Probe	Z116098_0046 (PA BBO 400S1 BBF-H-D-05 Z SP)
Number of Scans	1024
Receiver Gain	191.2
Relaxation Delay	2.0000
Pulse Width	10.0000
Presaturation Frequency	
Acquisition Time	1.3631
Acquisition Date	2020-07-08T00:22:34
Modification Date	2020-07-08T00:22:34
Class	
Spectrometer Frequency	100.49
Spectral Width	24038.5
Lowest Frequency	-1834.9
Nucleus	13C
Acquired Size	32768
Spectral Size	65536

51

AL450 full charac MeOD 10072020.1.fid

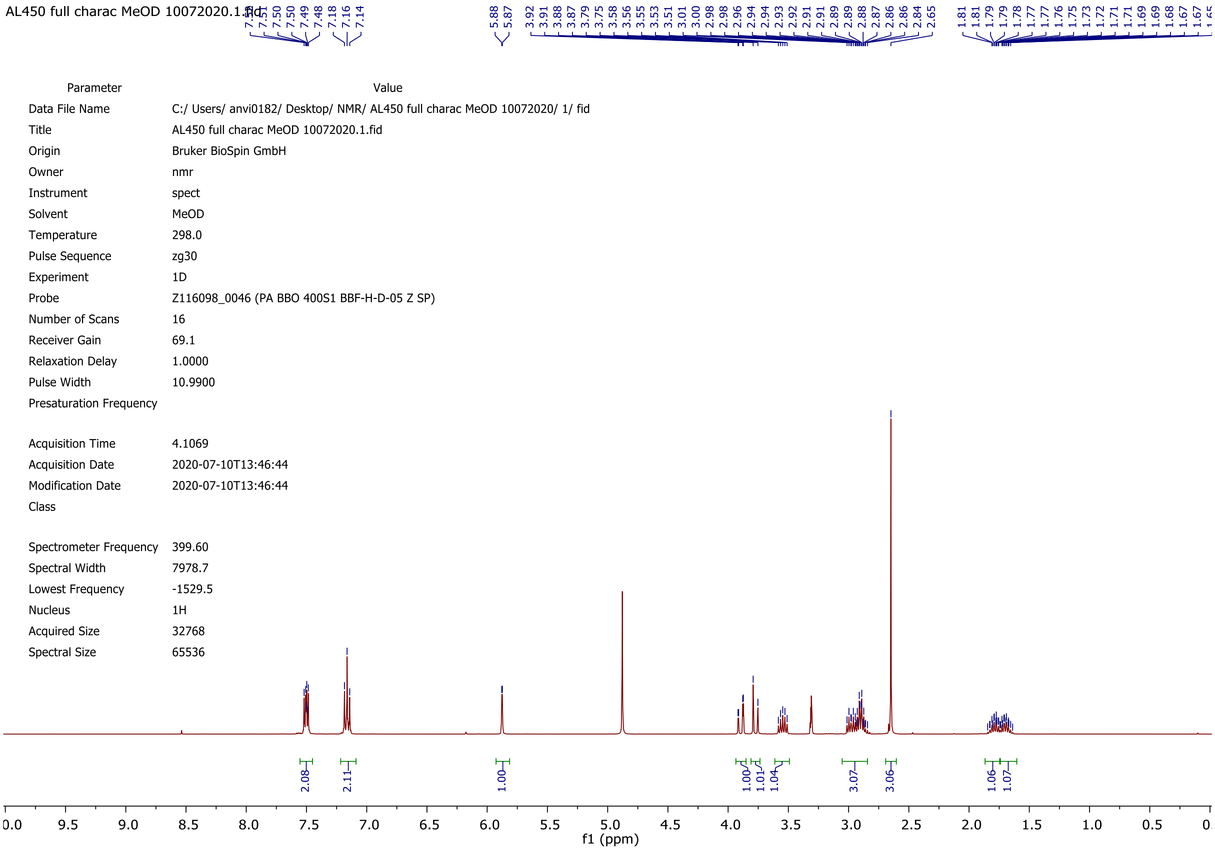

Parameter	Value
Data File Name	C:/ Users/ anvi0182/ Desktop/ NMR/ AL450 full charac MeOD 10072020/ 1/ fid
Title	AL450 full charac MeOD 10072020.1.fid
Origin	Bruker BioSpin GmbH
Owner	nmr
Instrument	spect
Solvent	MeOD
Temperature	298.0
Pulse Sequence	zg30
Experiment	1D
Probe	Z116098_0046 (PA BBO 400S1 BBF-H-D-05 Z SP)
Number of Scans	16
Receiver Gain	69.1
Relaxation Delay	1.0000
Pulse Width	10.9900
Presaturation Frequency	
Acquisition Time	4.1069
Acquisition Date	2020-07-10T13:46:44
Modification Date	2020-07-10T13:46:44
Class	
Spectrometer Frequency	399.60
Spectral Width	7978.7
Lowest Frequency	-1529.5
Nucleus	1H
Acquired Size	32768
Spectral Size	65536

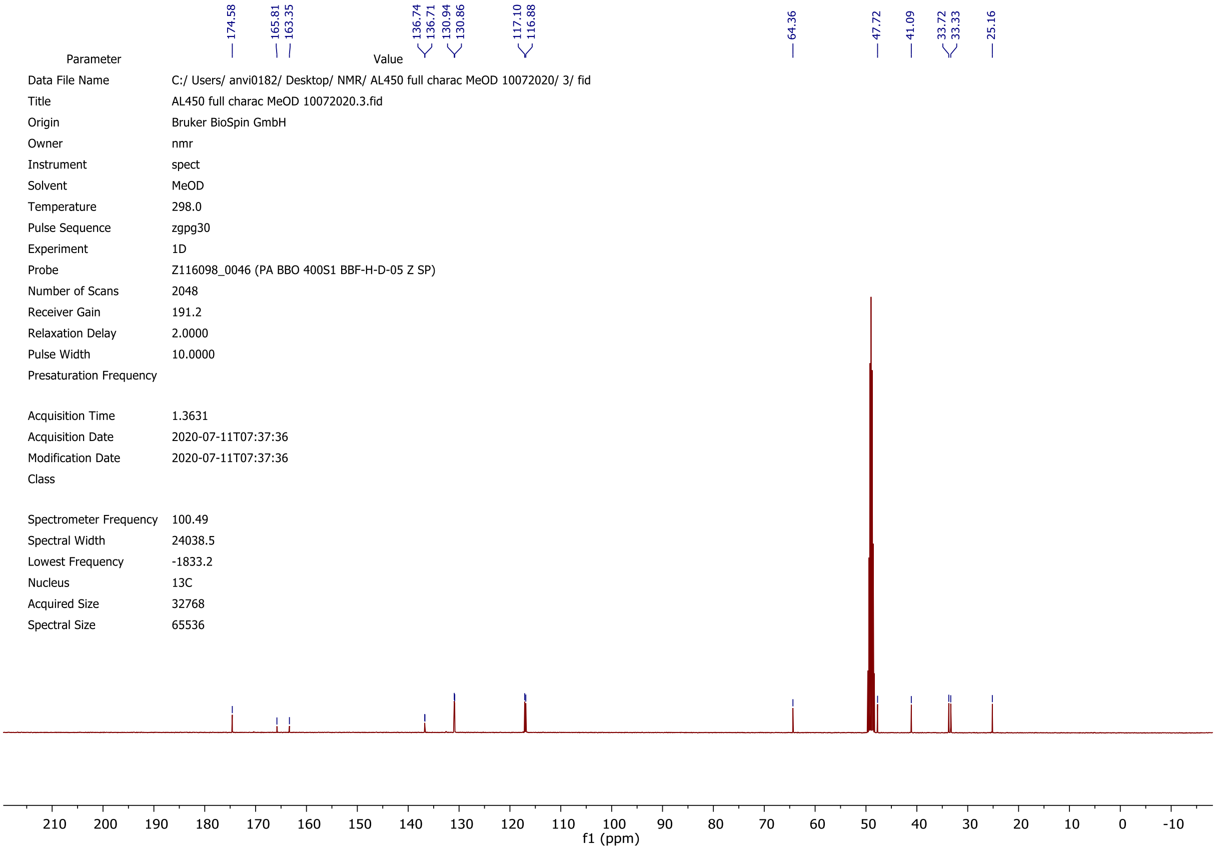

Parameter	Value
Data File Name	C:/ Users/ anvi0182/ Desktop/ NMR/ AL450 full charac MeOD 10072020/ 3/ fid
Title	AL450 full charac MeOD 10072020.3.fid
Origin	Bruker BioSpin GmbH
Owner	nmr
Instrument	spect
Solvent	MeOD
Temperature	298.0
Pulse Sequence	zgpg30
Experiment	1D
Probe	Z116098_0046 (PA BBO 400S1 BBF-H-D-05 Z SP)
Number of Scans	2048
Receiver Gain	191.2
Relaxation Delay	2.0000
Pulse Width	10.0000
Presaturation Frequency	
Acquisition Time	1.3631
Acquisition Date	2020-07-11T07:37:36
Modification Date	2020-07-11T07:37:36
Class	
Spectrometer Frequency	100.49
Spectral Width	24038.5
Lowest Frequency	-1833.2
Nucleus	13C
Acquired Size	32768
Spectral Size	65536

AL450 full charac MeOD 10072020.5.fid

Parameter	Value
Data File Name	C:/ Users/ anvi0182/ Desktop/ NMR/ AL450 full charac MeOD 10072020/ 5/ fid
Title	AL450 full charac MeOD 10072020.5.fid
Comment	
Origin	Bruker BioSpin GmbH
Owner	nmr
Site	
Instrument	spect
Author	
Solvent	MeOD
Temperature	298.0
Pulse Sequence	zgfhigqn.2
Experiment	1D
Probe	Z116098_0046 (PA BBO 400S1 BBF-H-D-05 Z SP)
Number of Scans	16
Receiver Gain	191.2
Relaxation Delay	1.0000
Pulse Width	15.5000
Presaturation Frequency	
Acquisition Time	0.7340
Acquisition Date	2020-07-11T07:59:14
Modification Date	2020-07-11T07:59:14
Class	
Spectrometer Frequency	376.96
Spectral Width	89285.7
Lowest Frequency	-82242.9
Nucleus	19F
Acquired Size	65536
Spectral Size	131072

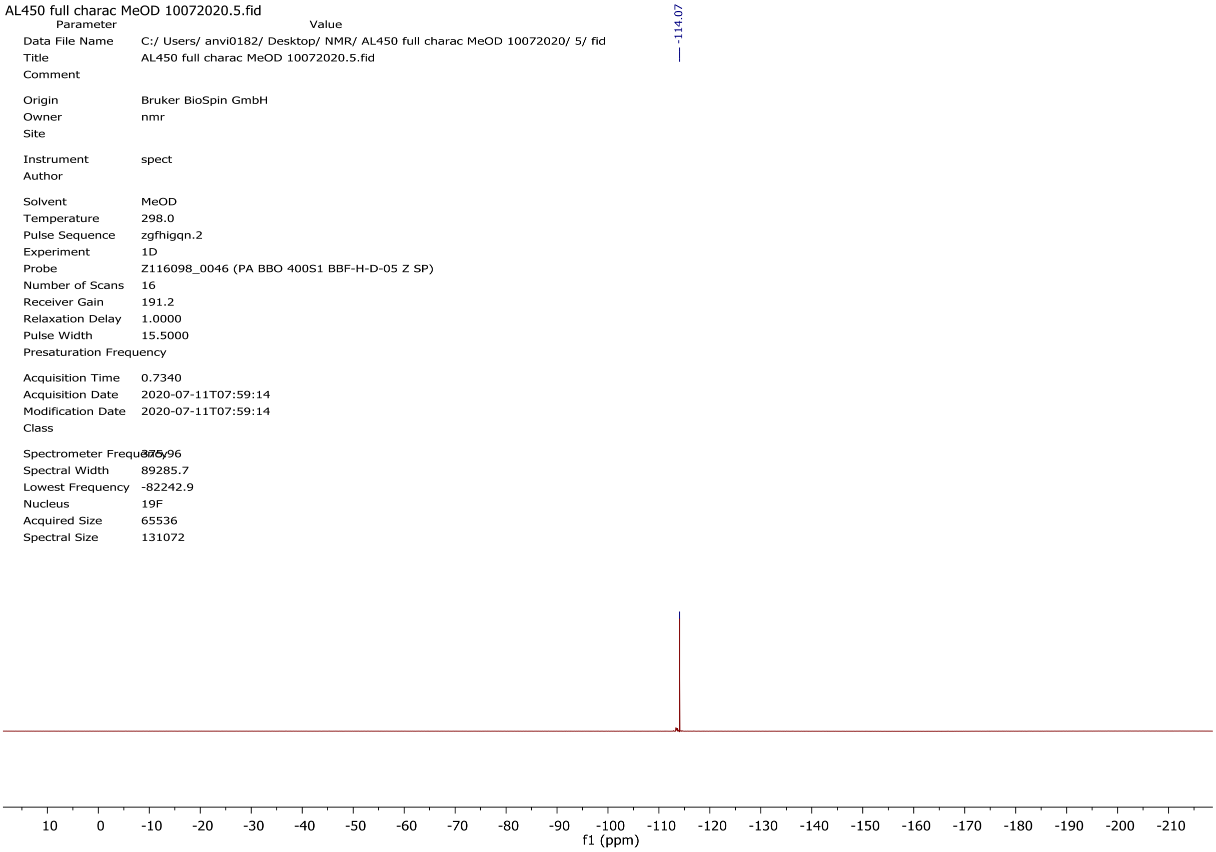

52

AVA-046-1 HCl full charac DMSO.1.fid

Parameter	Value
Data File Name	C:/ Users/ anvi0182/ Desktop/ NMR/ AVA-046-1 HCl full charac DMSO/ 1/ fid
Title	AVA-046-1 HCl full charac DMSO.1.fid
Origin	Bruker BioSpin GmbH
Owner	nmr
Instrument	spect
Solvent	DMSO
Temperature	298.0
Pulse Sequence	zg30
Experiment	1D
Probe	Z116098_0046 (PA BBO 400S1 BBF-H-D-05 Z SP)
Number of Scans	16
Receiver Gain	107.6
Relaxation Delay	1.0000
Pulse Width	10.6200
Presaturation Frequency	
Acquisition Time	4.1069
Acquisition Date	2018-12-16T19:33:51
Modification Date	2018-12-16T19:33:51
Class	
Spectrometer Frequency	399.60
Spectral Width	7978.7
Lowest Frequency	-1524.9
Nucleus	1H
Acquired Size	32768
Spectral Size	65536

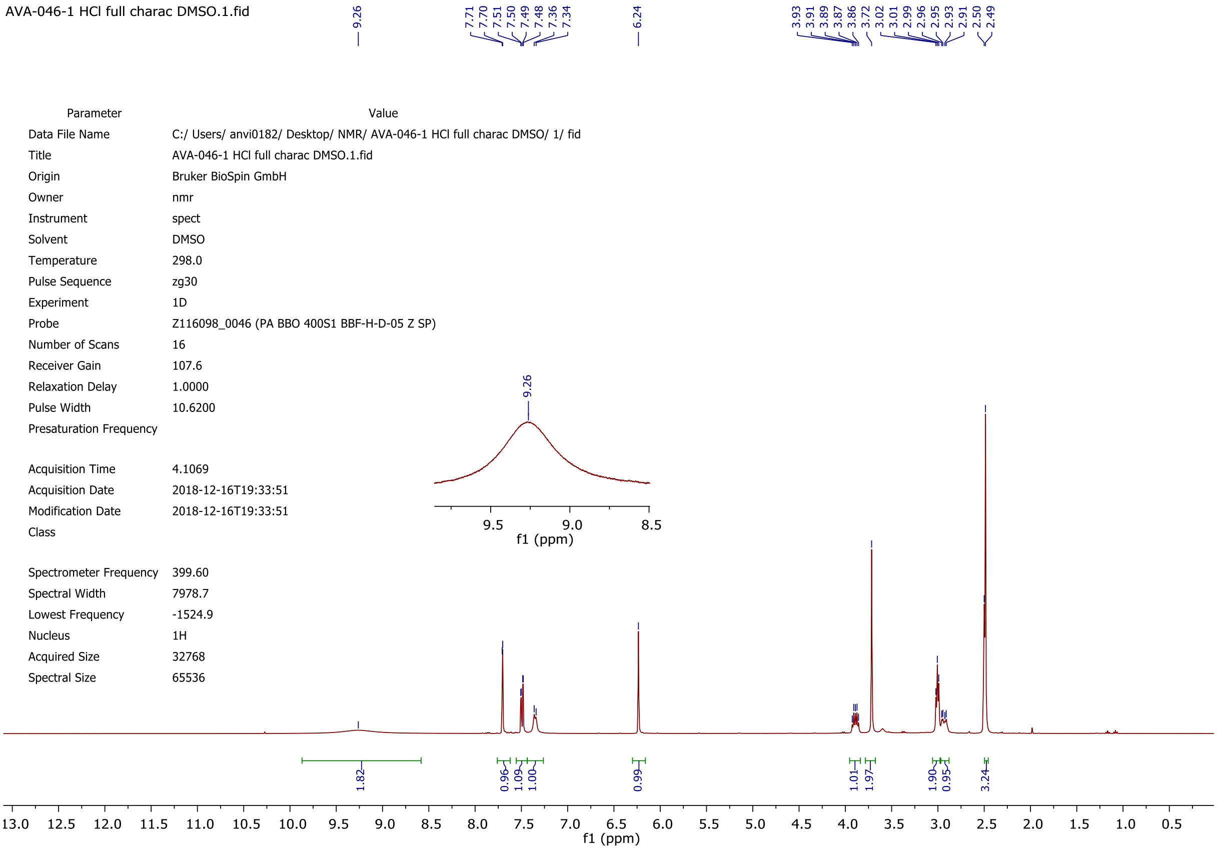

Parameter	Value
Data File Name	C:/ Users/ anvi0182/ Desktop/ NMR/ AL432 full charac DMSO 14072020/ 4/ fid
Title	AL432 full charac DMSO 14072020.4.fid
Comment	
Origin	Bruker BioSpin GmbH
Owner	nmr
Site	
Instrument	spect
Author	
Solvent	DMSO
Temperature	298.0
Pulse Sequence	zgpg30
Experiment	1D
Probe	Z116098_0046 (PA BBO 400S1 BBF-H-D-05 Z SP)
Number of Scans	3072
Receiver Gain	191.2
Relaxation Delay	2.0000
Pulse Width	10.0000
Presaturation Frequency	
Acquisition Time	1.3631
Acquisition Date	2020-07-14T22:01:10
Modification Date	2020-07-14T22:01:10
Class	
Spectrometer Frequency	100.49
Spectral Width	24038.5
Lowest Frequency	-2019.9
Nucleus	13C
Acquired Size	32768
Spectral Size	65536

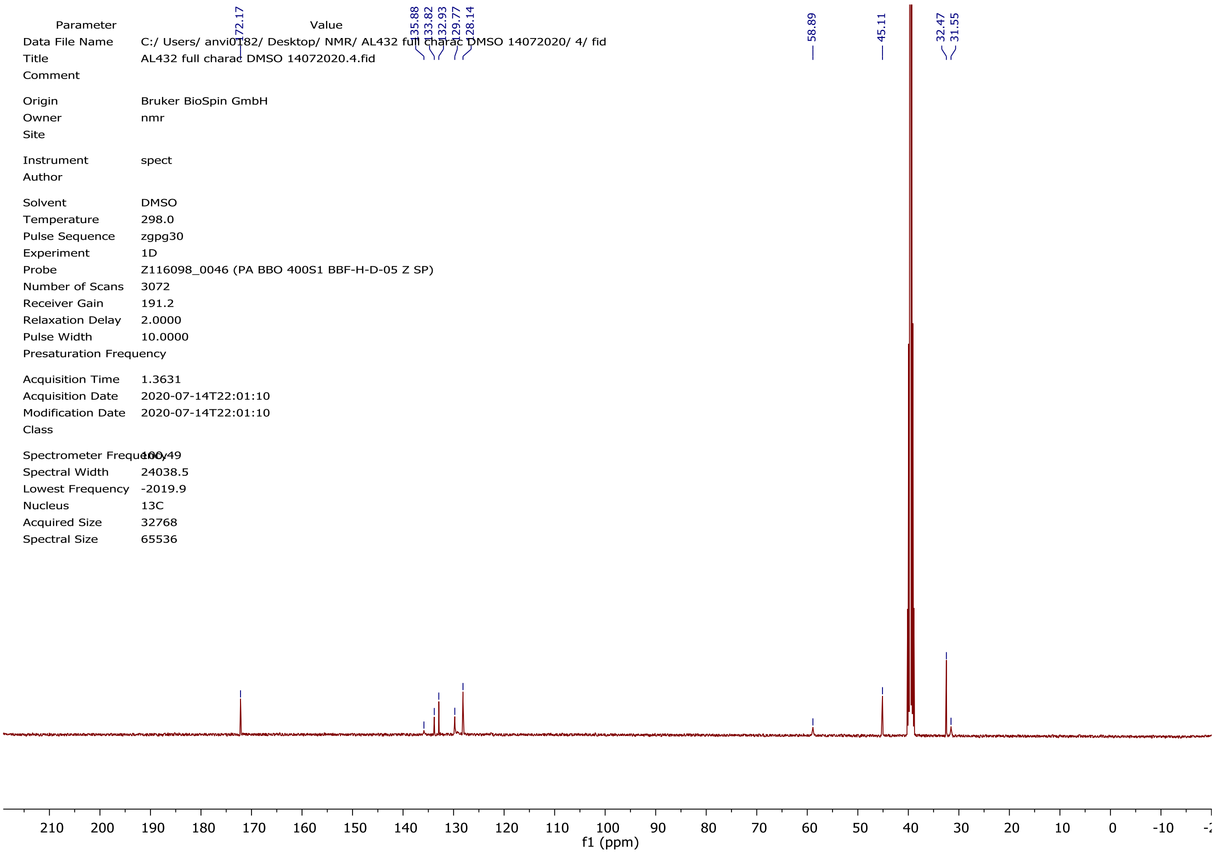

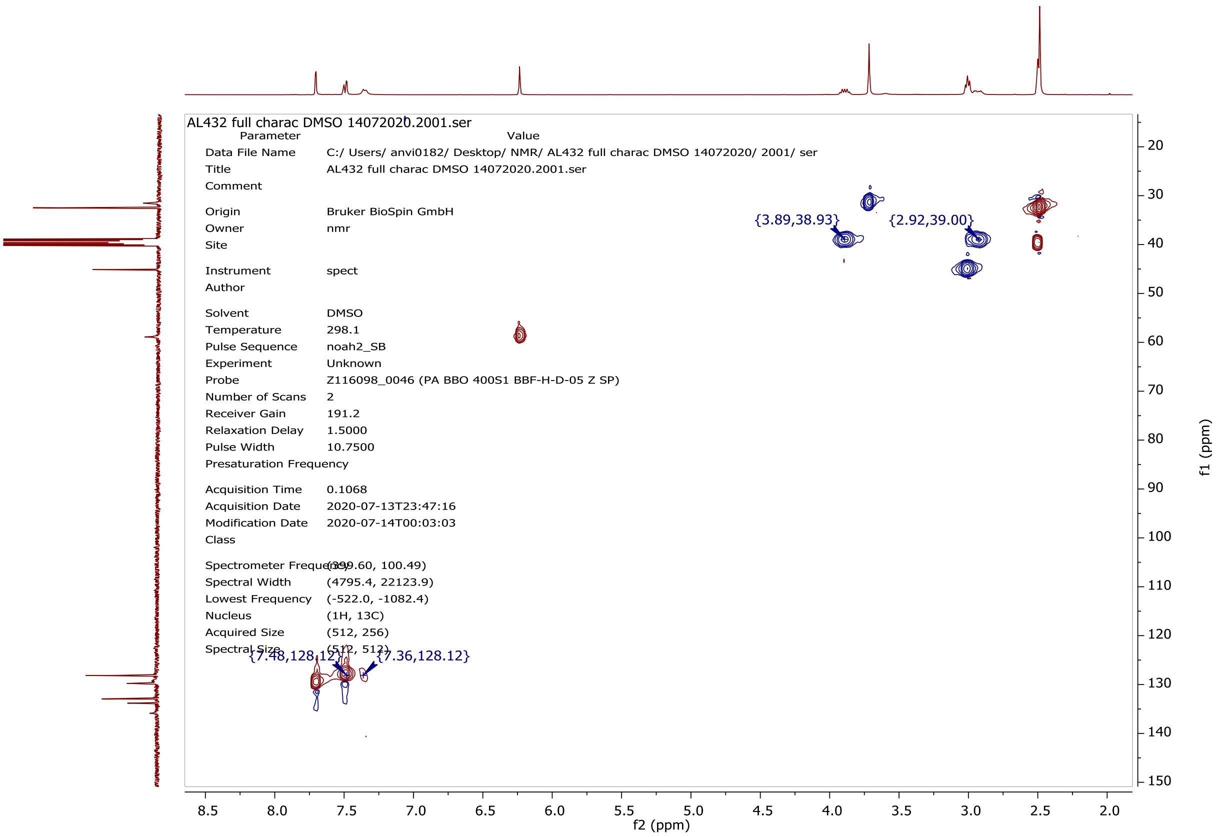
AL432 full charac DMSO 14072020.2001.ser
Parameter
Value
Data File Name
C:/ Users/ anvi0182/ Desktop/ NMR/ AL432 full charac DMSO 14072020/ 2001/ ser
Title
AL432 full charac DMSO 14072020.2001.ser
Comment
Origin
Bruker BioSpin GmbH
Owner
nmr
Site
Instrument
spect
Author
Solvent
DMSO
Temperature
298.1
Pulse Sequence
noah2_SB
Experiment
Unknown
Probe
Z116098_0046 (PA BBO 400S1 BBF-H-D-05 Z SP)
Number of Scans
2
Receiver Gain
191.2
Relaxation Delay
1.5000
Pulse Width
10.7500
Presaturation Frequency
Acquisition Time
0.1068
Acquisition Date
2020-07-13T23:47:16
Modification Date
2020-07-14T00:03:03
Class
Spectrometer Frequency
(399.60, 100.49)
Spectral Width
(4795.4, 22123.9)
Lowest Frequency
(-522.0, -1082.4)
Nucleus
(1H, 13C)
Acquired Size
(512, 256)
Spectral Size
(512, 512)
{3.89,38.93}
{2.92,39.00}
{7.48,128.12}
{7.36,128.12}
f2 (ppm)
f1 (ppm)

53

AVA-028-1 HCl full charac DMSO.1.fid

Parameter	Value
Data File Name	C:/ Users/ anvi0182/ Desktop/ NMR/ AVA-028-1 HCl full charac DMSO/ 1/ fid
Title	AVA-028-1 HCl full charac DMSO.1.fid
Origin	Bruker BioSpin GmbH
Owner	nmr
Instrument	spect
Solvent	DMSO
Temperature	298.0
Pulse Sequence	zg30
Experiment	1D
Probe	Z116098_0046 (PA BBO 400S1 BBF-H-D-05 Z SP)
Number of Scans	16
Receiver Gain	84.7
Relaxation Delay	1.0000
Pulse Width	10.6700
Presaturation Frequency	
Acquisition Time	4.1069
Acquisition Date	2018-12-16T18:26:43
Modification Date	2018-12-16T18:26:43
Class	
Spectrometer Frequency	399.60
Spectral Width	7978.7
Lowest Frequency	-1524.9
Nucleus	1H
Acquired Size	32768
Spectral Size	65536

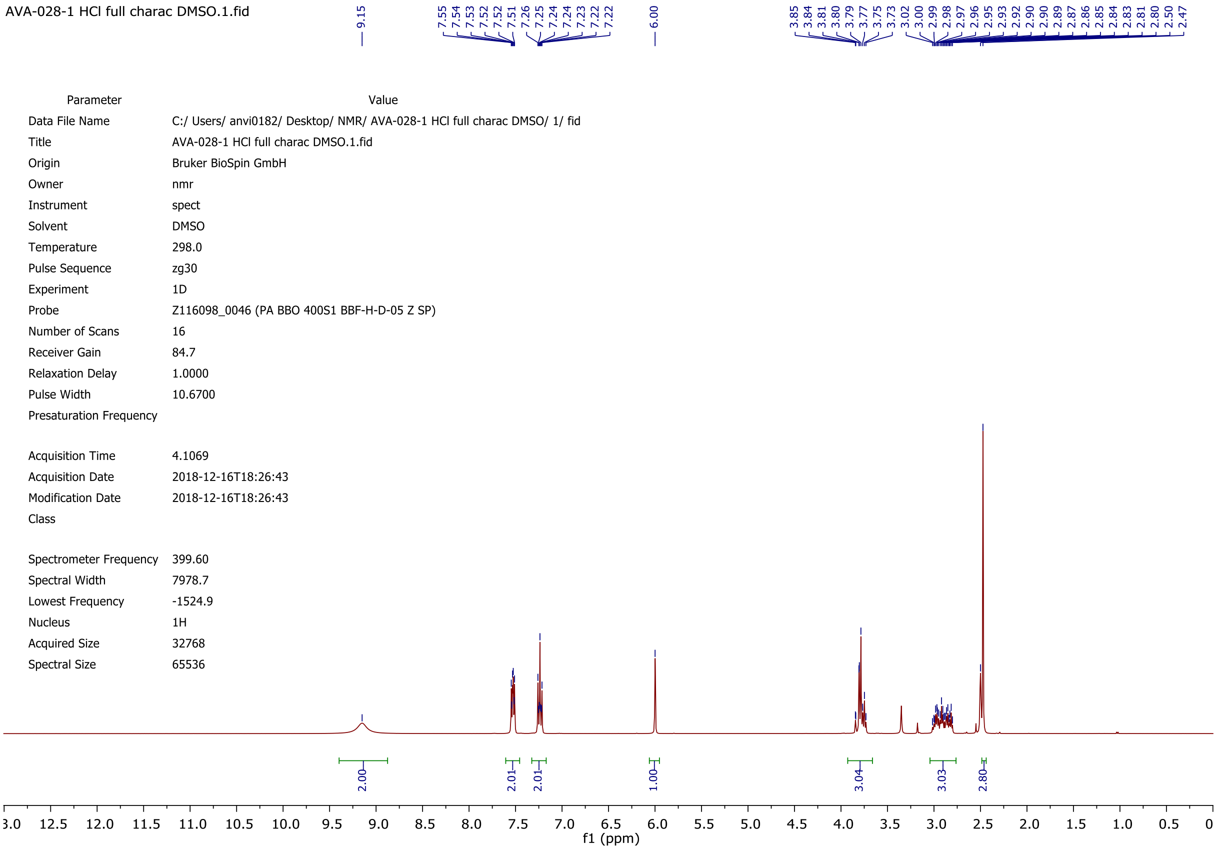

AVA-028-1 HCl full charac DMSO.2.fid

Parameter	Value
Data File Name	C:/ Users/ anvi0182/ Desktop/ NMR/ AVA-028-1 HCl full charac DMSO/ 2/ fid
Title	AVA-028-1 HCl full charac DMSO.2.fid
Origin	Bruker BioSpin GmbH
Owner	nmr
Instrument	spect
Solvent	DMSO
Temperature	298.0
Pulse Sequence	zgpg30
Experiment	1D
Probe	Z116098_0046 (PA BBO 400S1 BBF-H-D-05 Z SP)
Number of Scans	1024
Receiver Gain	191.2
Relaxation Delay	2.0000
Pulse Width	10.0000
Presaturation Frequency	
Acquisition Time	1.3631
Acquisition Date	2018-12-16T19:27:14
Modification Date	2018-12-16T19:27:14
Class	
Spectrometer Frequency	100.49
Spectral Width	24038.5
Lowest Frequency	-2019.5
Nucleus	13C
Acquired Size	32768
Spectral Size	65536

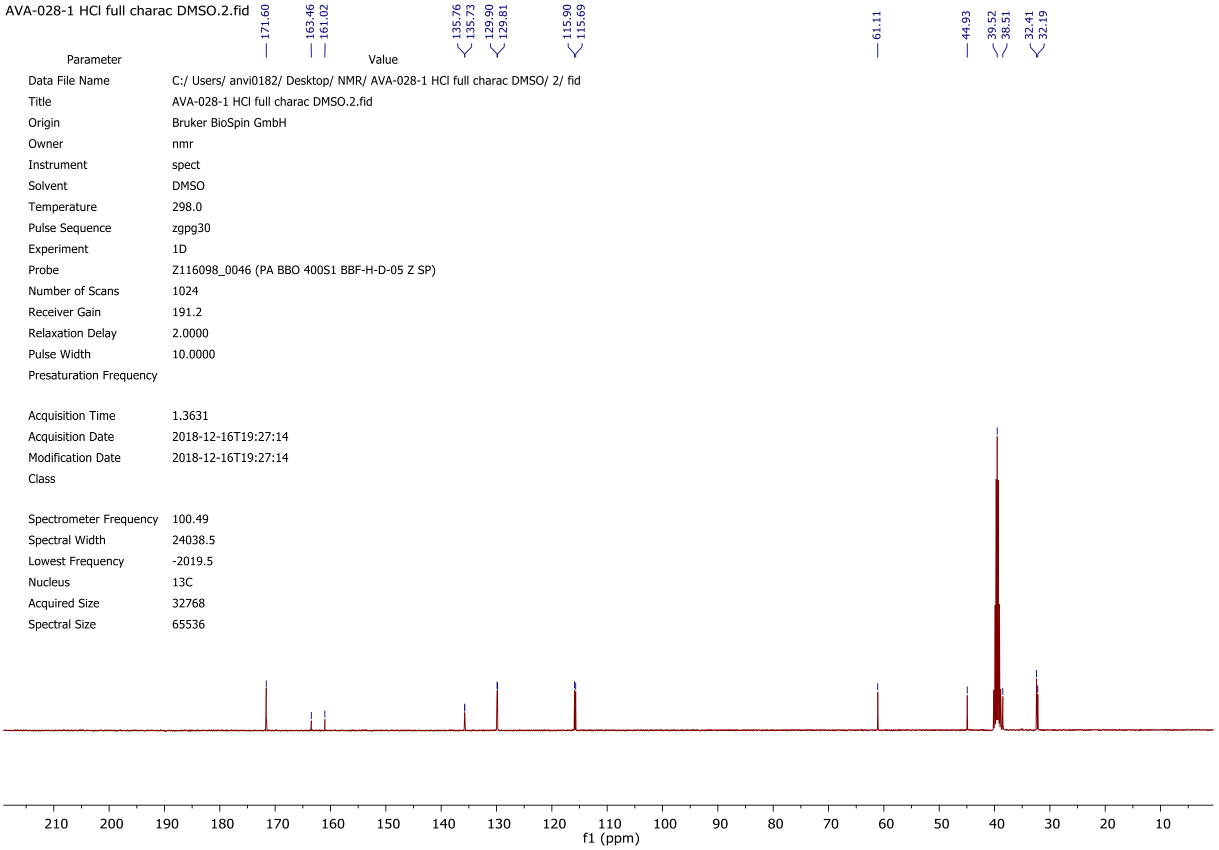

AL430 19F DMSO.1.fid

Parameter	Value
Data File Name	C:/ Users/ anvi0182/ Desktop/ NMR/ AL430 19F DMSO/ 1/ fid
Title	AL430 19F DMSO.1.fid
Comment	
Origin	Bruker BioSpin GmbH
Owner	nmr
Site	
Instrument	spect
Author	
Solvent	DMSO
Temperature	298.0
Pulse Sequence	zgfhigqn.2
Experiment	1D
Probe	Z116098_0046 (PA BBO 400S1 BBF-H-D-05 Z SP)
Number of Scans	16
Receiver Gain	191.2
Relaxation Delay	1.0000
Pulse Width	15.5000
Presaturation Frequency	
Acquisition Time	0.7340
Acquisition Date	2020-07-17T18:01:28
Modification Date	2020-07-17T18:01:28
Class	
Spectrometer Frequency	376.96
Spectral Width	89285.7
Lowest Frequency	-82242.9
Nucleus	19F
Acquired Size	65536
Spectral Size	131072

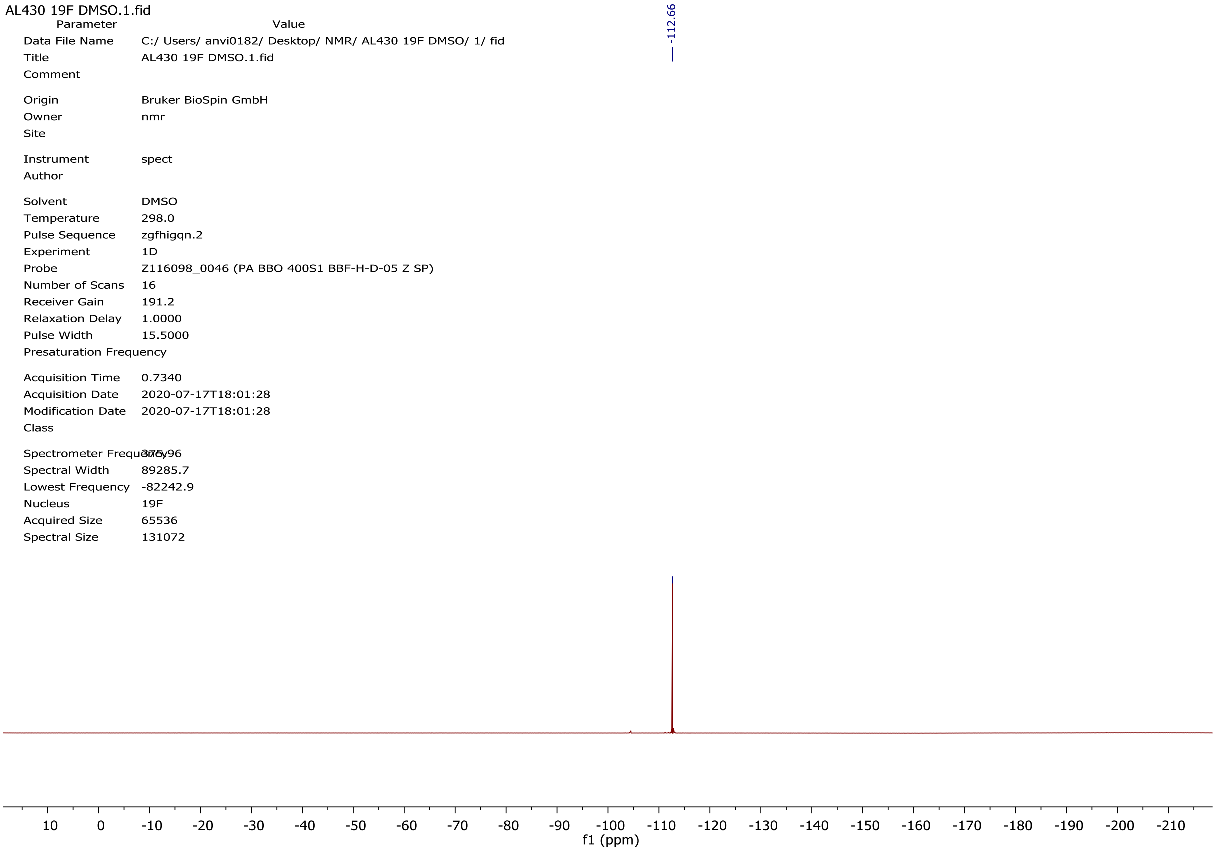

54

AVA-442-1 full charac 600 70C.1.fid

Parameter	Value
Data File Name	C:/ Users/ anvi0182/ Desktop/ NMR/ AVA-442-1 full charac 600 70C/ 1/ fid
Title	AVA-442-1 full charac 600 70C.1.fid
Origin	Bruker BioSpin GmbH
Owner	nmr
Instrument	spect
Solvent	DMSO
Temperature	343.0
Pulse Sequence	zg30
Experiment	1D
Probe	Z132572_0007 (CP BBO 600S3 BB-H&F-D-05 Z)
Number of Scans	16
Receiver Gain	104.4
Relaxation Delay	1.0000
Pulse Width	12.4000
Presaturation Frequency	
Acquisition Time	2.7263
Acquisition Date	2020-11-07T13:23:32
Modification Date	2020-11-07T13:23:32
Class	
Spectrometer Frequency	600.17
Spectral Width	12019.2
Lowest Frequency	-2308.1
Nucleus	1H
Acquired Size	32768
Spectral Size	131072

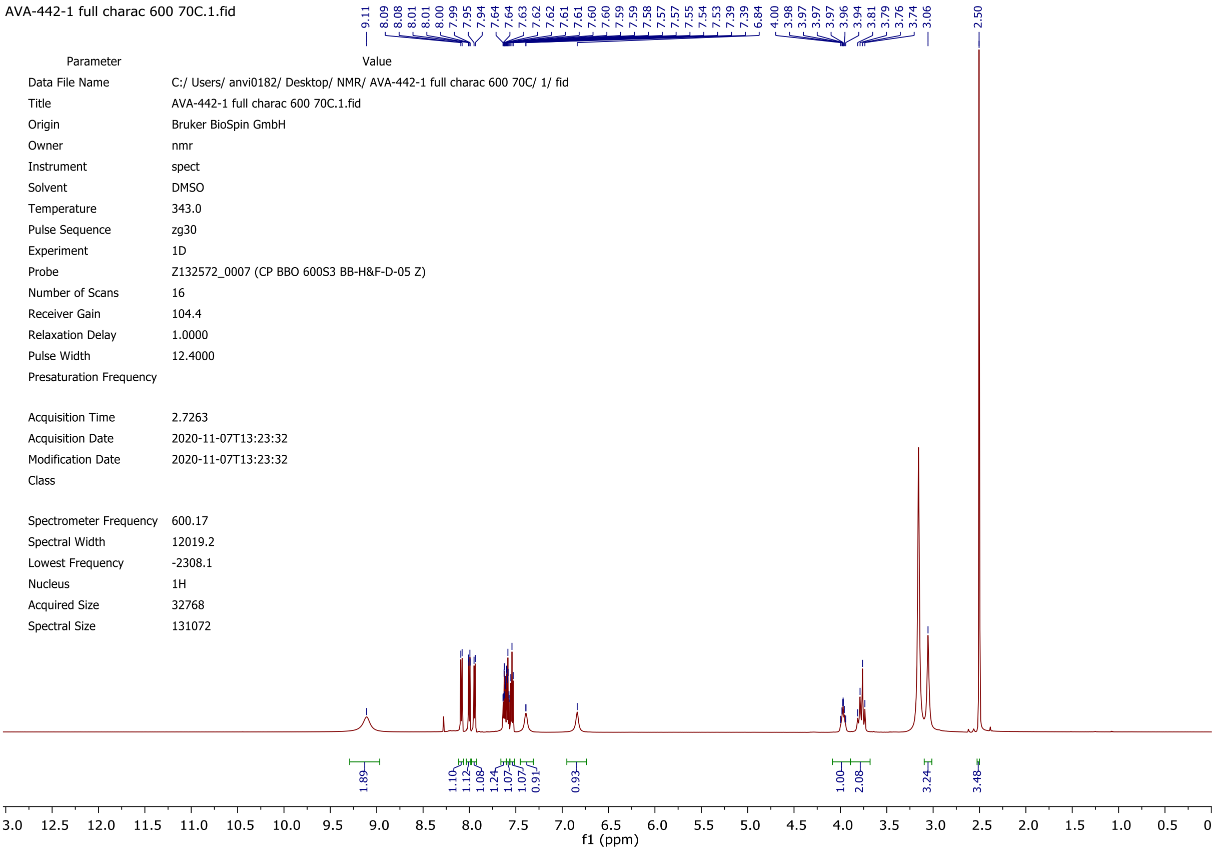

Parameter	Value
Data File Name	C:/ Users/ anvi0182/ Desktop/ NMR/ AVA-442-1 full charac 600 70C/ 2/ fid
Title	AVA-442-1 full charac 600 70C.2.fid
Comment	
Origin	Bruker BioSpin GmbH
Owner	nmr
Site	
Instrument	spect
Author	
Solvent	DMSO
Temperature	343.0
Pulse Sequence	zgpg30
Experiment	1D
Probe	Z132572_0007 (CP BBO 600S3 BB-H&F-D-05 Z)
Number of Scans	3072
Receiver Gain	182.7
Relaxation Delay	2.0000
Pulse Width	9.7000
Presaturation Frequency	
Acquisition Time	0.9088
Acquisition Date	2020-11-07T15:56:52
Modification Date	2020-11-07T15:56:52
Class	
Spectrometer Frequency	150.93
Spectral Width	36057.7
Lowest Frequency	-3058.0
Nucleus	13C
Acquired Size	32768
Spectral Size	65536

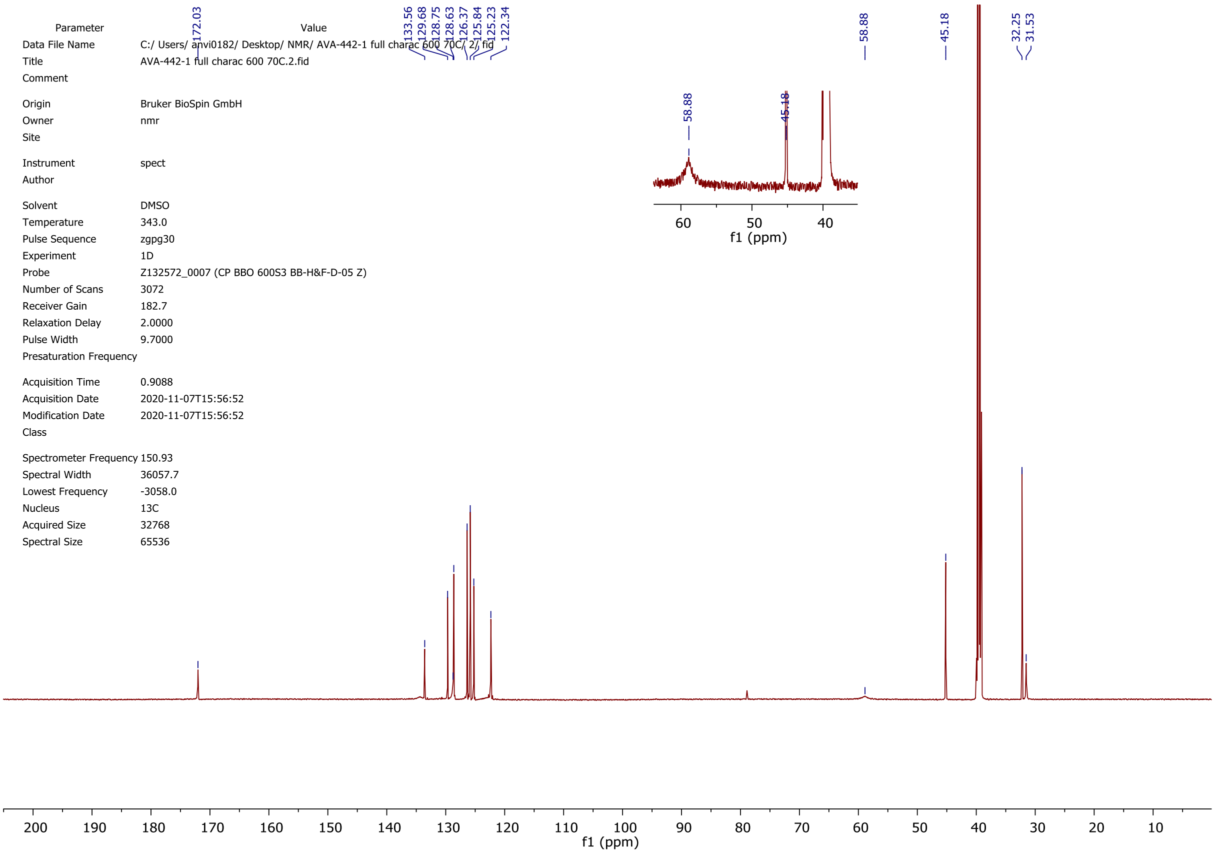

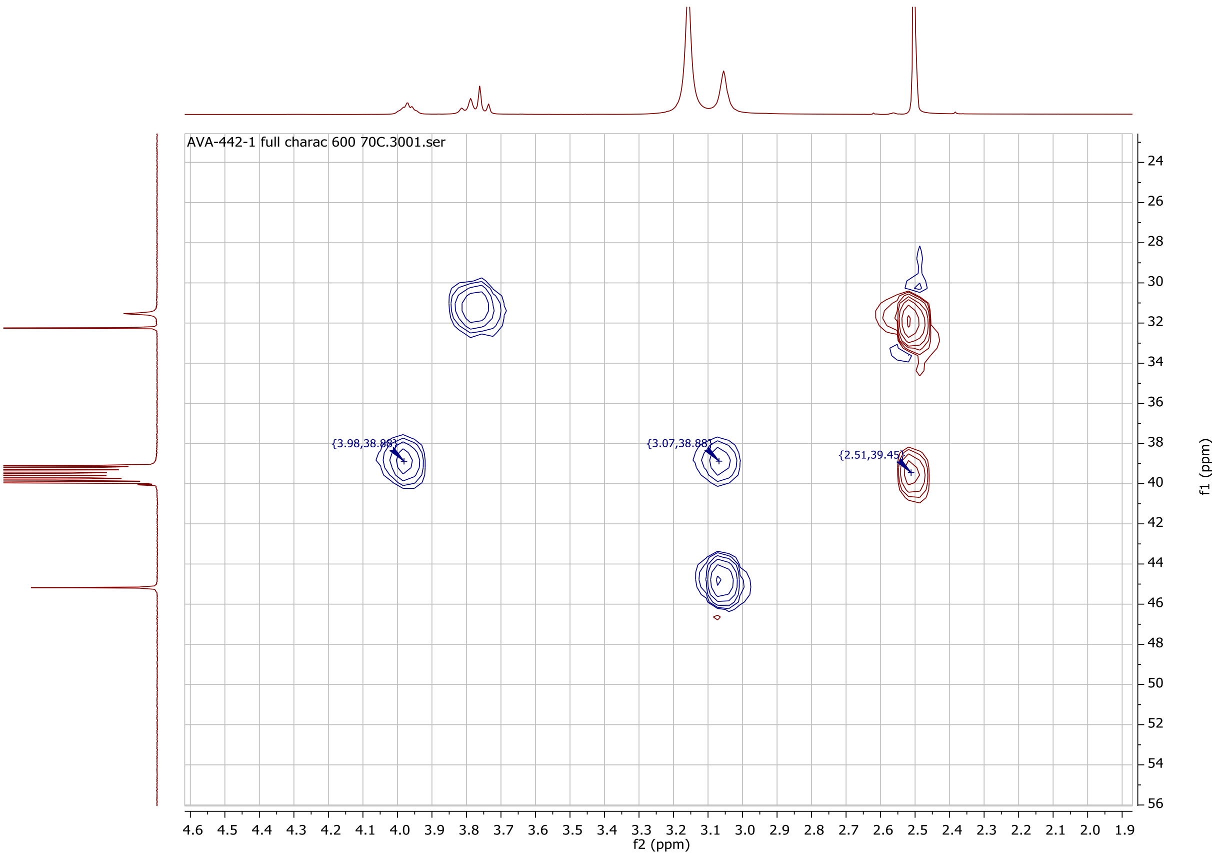
AVA-442-1 full charac 600 70C.3001.ser
{3.98,38.88}
{3.07,38.88}
{2.51,39.45}
f2 (ppm)
f1 (ppm)

55

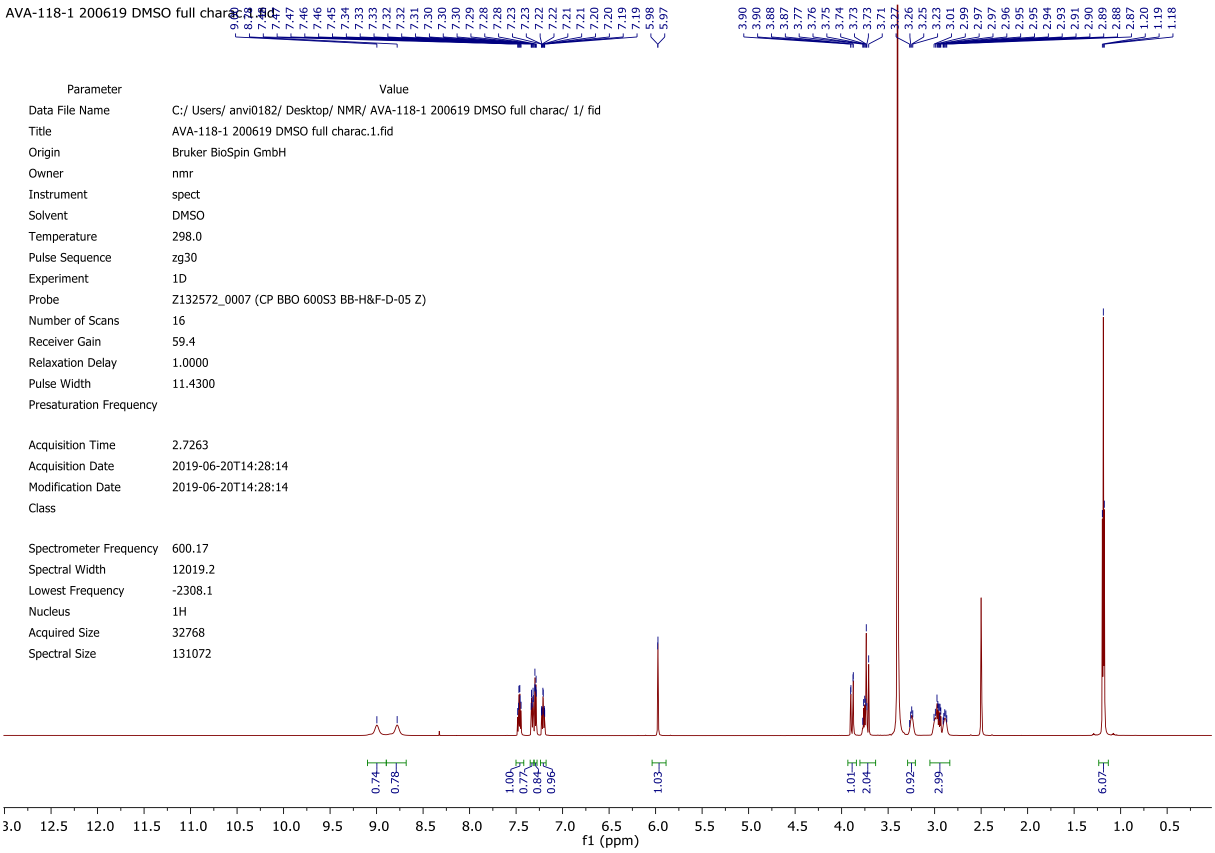

Parameter	Value
Data File Name	C:/ Users/ anvi0182/ Desktop/ NMR/ AVA-118-1 200619 DMSO full charac/ 1/ fid
Title	AVA-118-1 200619 DMSO full charac.1.fid
Origin	Bruker BioSpin GmbH
Owner	nmr
Instrument	spect
Solvent	DMSO
Temperature	298.0
Pulse Sequence	zg30
Experiment	1D
Probe	Z132572_0007 (CP BBO 600S3 BB-H&F-D-05 Z)
Number of Scans	16
Receiver Gain	59.4
Relaxation Delay	1.0000
Pulse Width	11.4300
Presaturation Frequency	
Acquisition Time	2.7263
Acquisition Date	2019-06-20T14:28:14
Modification Date	2019-06-20T14:28:14
Class	
Spectrometer Frequency	600.17
Spectral Width	12019.2
Lowest Frequency	-2308.1
Nucleus	1H
Acquired Size	32768
Spectral Size	131072

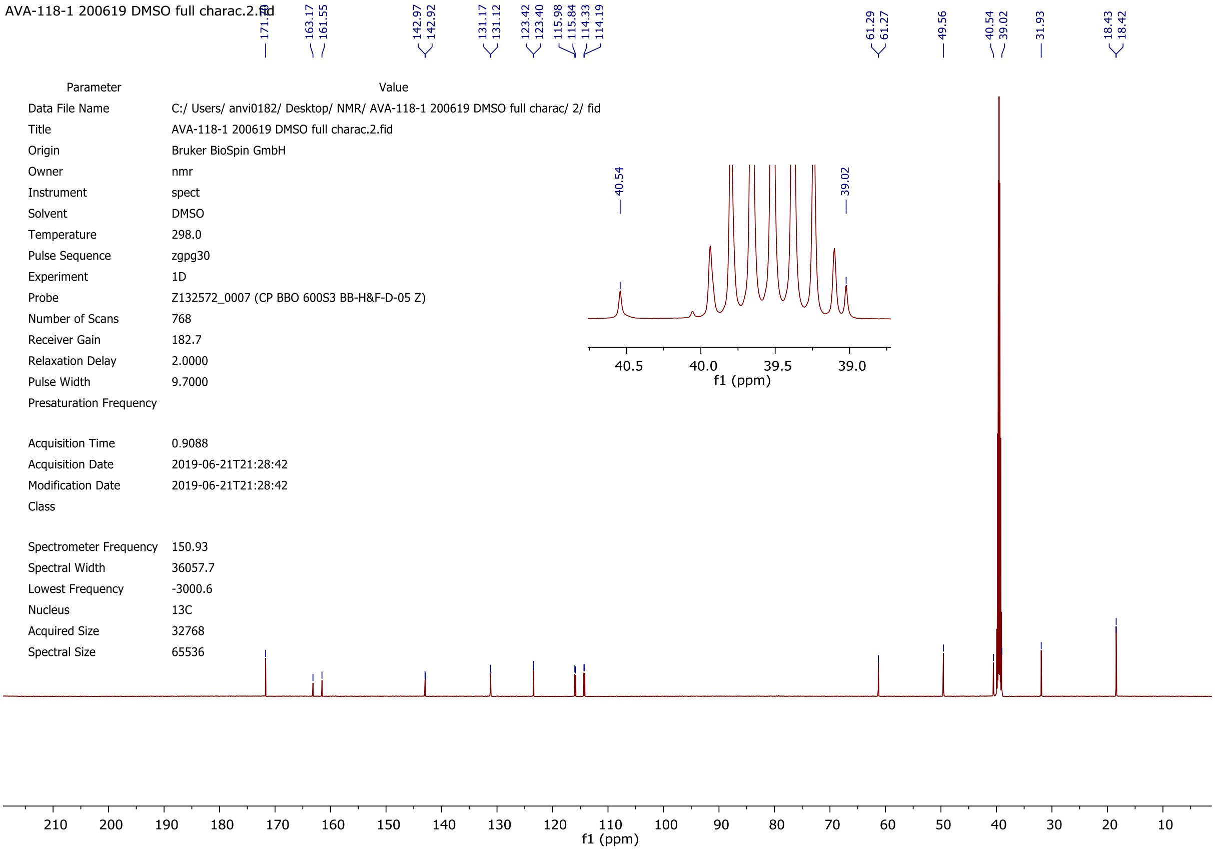

Parameter	Value
Data File Name	C:/ Users/ anvi0182/ Desktop/ NMR/ AVA-118-1 200619 DMSO full charac/ 2/ fid
Title	AVA-118-1 200619 DMSO full charac.2.fid
Origin	Bruker BioSpin GmbH
Owner	nmr
Instrument	spect
Solvent	DMSO
Temperature	298.0
Pulse Sequence	zgpg30
Experiment	1D
Probe	Z132572_0007 (CP BBO 600S3 BB-H&F-D-05 Z)
Number of Scans	768
Receiver Gain	182.7
Relaxation Delay	2.0000
Pulse Width	9.7000
Presaturation Frequency	
Acquisition Time	0.9088
Acquisition Date	2019-06-21T21:28:42
Modification Date	2019-06-21T21:28:42
Class	
Spectrometer Frequency	150.93
Spectral Width	36057.7
Lowest Frequency	-3000.6
Nucleus	13C
Acquired Size	32768
Spectral Size	65536

AL474 19F.1.fid

Parameter	Value
Data File Name	C:/ Users/ anvi0182/ Desktop/ NMR/ AL474 19F/ 1/ fid
Title	AL474 19F.1.fid
Comment	
Origin	Bruker BioSpin GmbH
Owner	nmr
Site	
Instrument	spect
Author	
Solvent	DMSO
Temperature	298.0
Pulse Sequence	zgfhigqn.2
Experiment	1D
Probe	Z116098_0046 (PA BBO 400S1 BBF-H-D-05 Z SP)
Number of Scans	16
Receiver Gain	191.2
Relaxation Delay	1.0000
Pulse Width	15.5000
Presaturation Frequency	
Acquisition Time	0.7340
Acquisition Date	2020-07-08T12:41:21
Modification Date	2020-07-08T12:41:21
Class	
Spectrometer Frequency	376.96
Spectral Width	89285.7
Lowest Frequency	-82242.9
Nucleus	19F
Acquired Size	65536
Spectral Size	131072

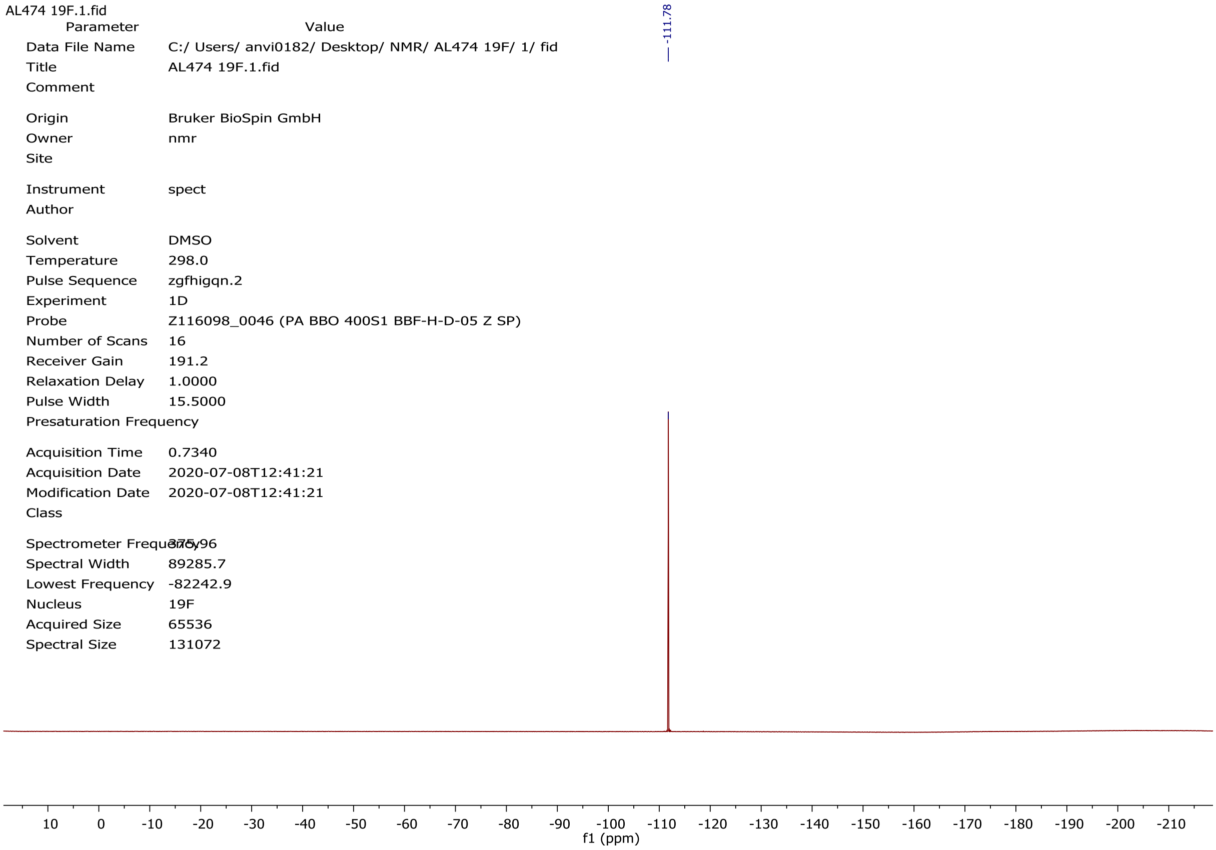

56

AVA-107-1 200619 DMSO full charac.1.fid

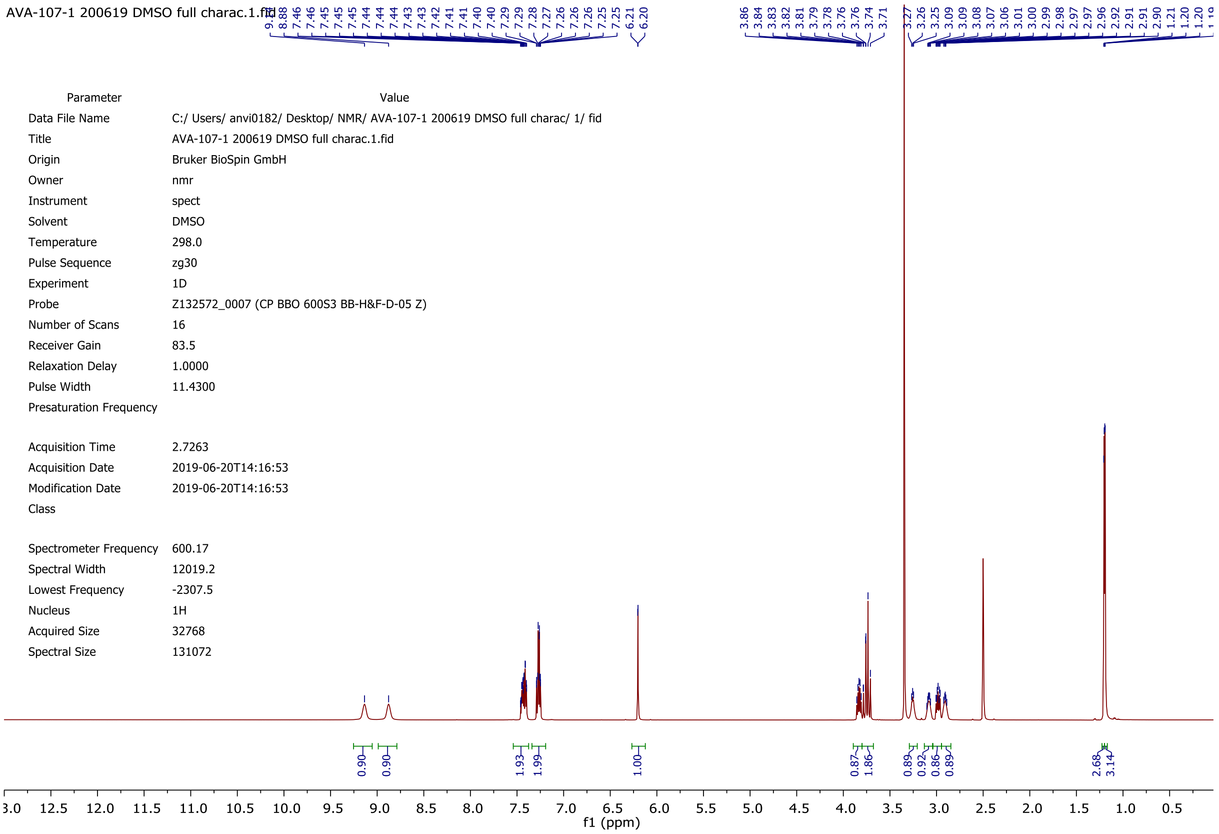

Parameter	Value
Data File Name	C:/ Users/ anvi0182/ Desktop/ NMR/ AVA-107-1 200619 DMSO full charac/ 1/ fid
Title	AVA-107-1 200619 DMSO full charac.1.fid
Origin	Bruker BioSpin GmbH
Owner	nmr
Instrument	spect
Solvent	DMSO
Temperature	298.0
Pulse Sequence	zg30
Experiment	1D
Probe	Z132572_0007 (CP BBO 600S3 BB-H&F-D-05 Z)
Number of Scans	16
Receiver Gain	83.5
Relaxation Delay	1.0000
Pulse Width	11.4300
Presaturation Frequency	
Acquisition Time	2.7263
Acquisition Date	2019-06-20T14:16:53
Modification Date	2019-06-20T14:16:53
Class	
Spectrometer Frequency	600.17
Spectral Width	12019.2
Lowest Frequency	-2307.5
Nucleus	1H
Acquired Size	32768
Spectral Size	131072

AVA-107-1 200619 DMSO full charac.2.fid

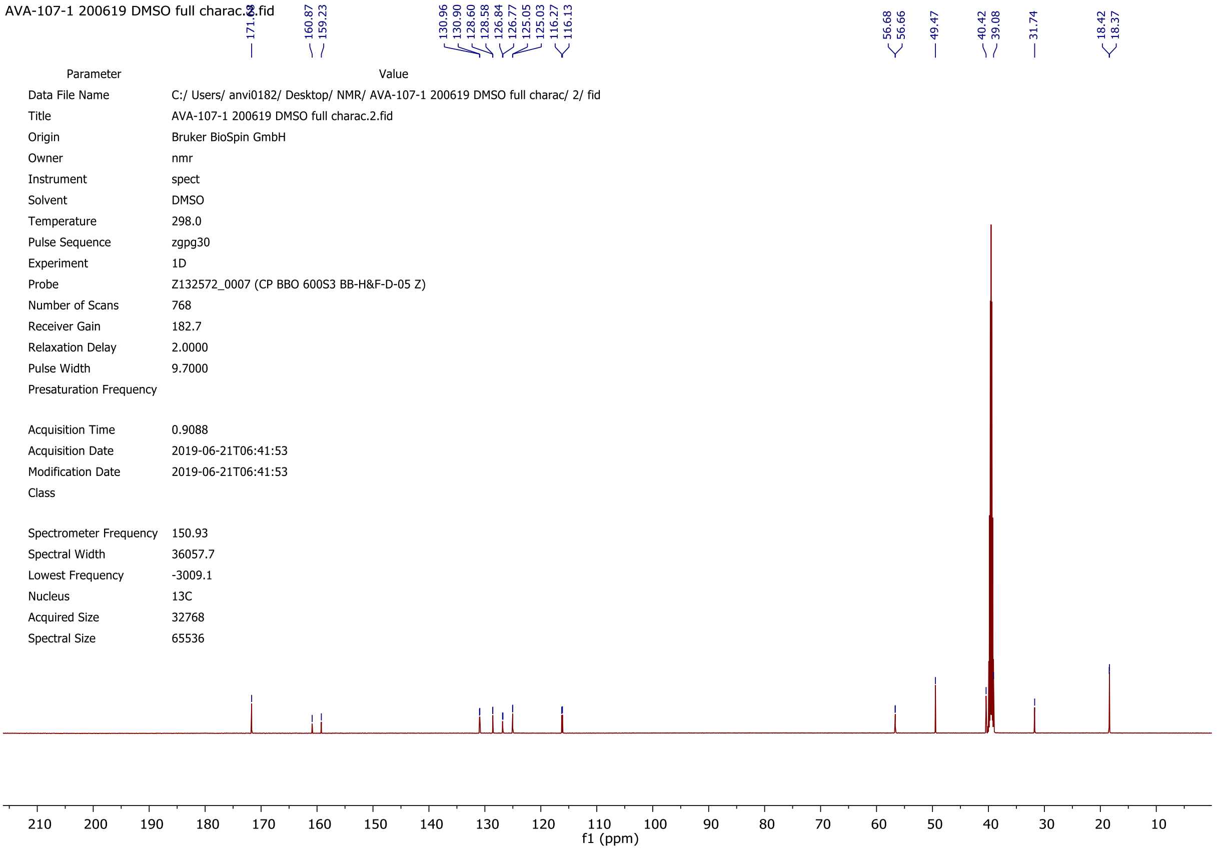

Parameter	Value
Data File Name	C:/ Users/ anvi0182/ Desktop/ NMR/ AVA-107-1 200619 DMSO full charac/ 2/ fid
Title	AVA-107-1 200619 DMSO full charac.2.fid
Origin	Bruker BioSpin GmbH
Owner	nmr
Instrument	spect
Solvent	DMSO
Temperature	298.0
Pulse Sequence	zgpg30
Experiment	1D
Probe	Z132572_0007 (CP BBO 600S3 BB-H&F-D-05 Z)
Number of Scans	768
Receiver Gain	182.7
Relaxation Delay	2.0000
Pulse Width	9.7000
Presaturation Frequency	
Acquisition Time	0.9088
Acquisition Date	2019-06-21T06:41:53
Modification Date	2019-06-21T06:41:53
Class	
Spectrometer Frequency	150.93
Spectral Width	36057.7
Lowest Frequency	-3009.1
Nucleus	13C
Acquired Size	32768
Spectral Size	65536

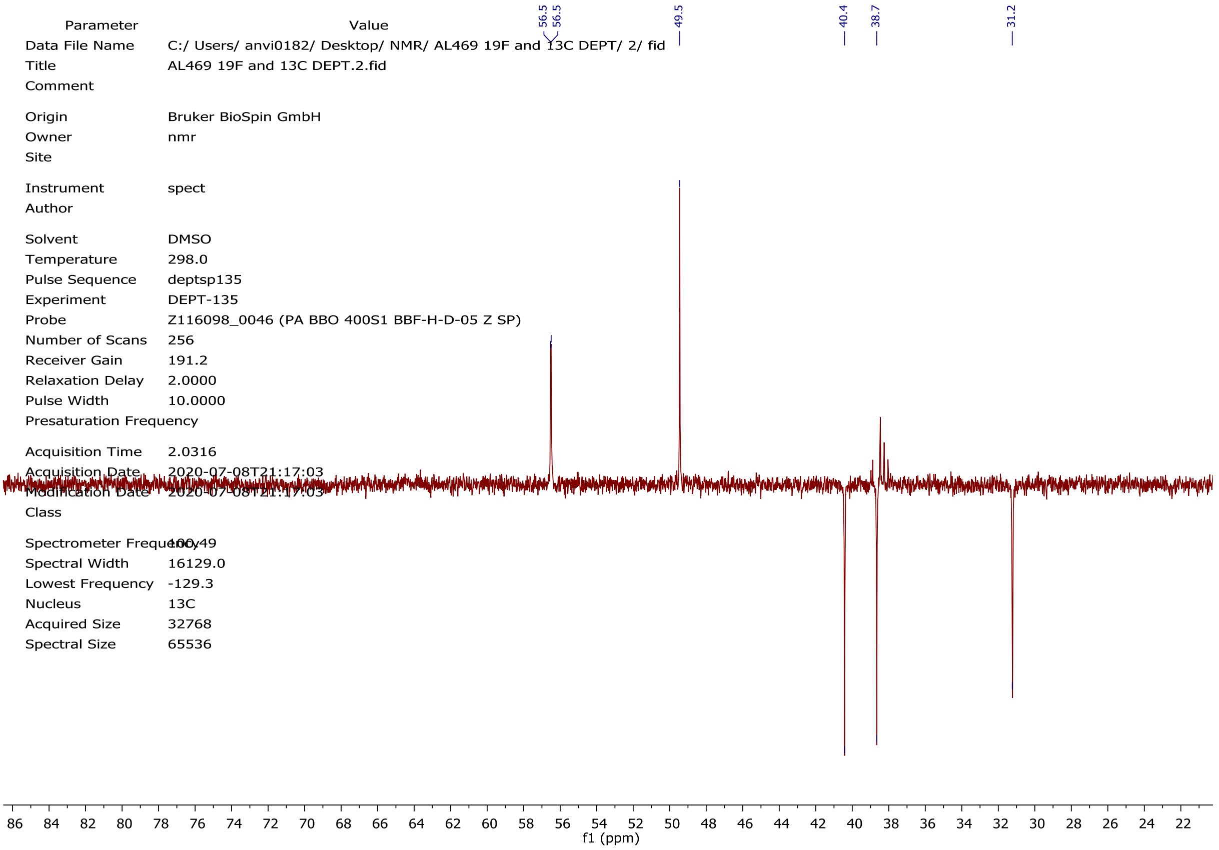

Parameter	Value
Data File Name	C:/ Users/ anvi0182/ Desktop/ NMR/ AL469 19F and 13C DEPT/ 2/ fid
Title	AL469 19F and 13C DEPT.2.fid
Comment	
Origin	Bruker BioSpin GmbH
Owner	nmr
Site	
Instrument	spect
Author	
Solvent	DMSO
Temperature	298.0
Pulse Sequence	deptsp135
Experiment	DEPT-135
Probe	Z116098_0046 (PA BBO 400S1 BBF-H-D-05 Z SP)
Number of Scans	256
Receiver Gain	191.2
Relaxation Delay	2.0000
Pulse Width	10.0000
Presaturation Frequency	
Acquisition Time	2.0316
Acquisition Date	2020-07-08T21:17:03
Modification Date	2020-07-08T21:17:03
Class	
Spectrometer Frequency	100.49
Spectral Width	16129.0
Lowest Frequency	-129.3
Nucleus	13C
Acquired Size	32768
Spectral Size	65536

AL469 19F and 13C DEPT.1.fid

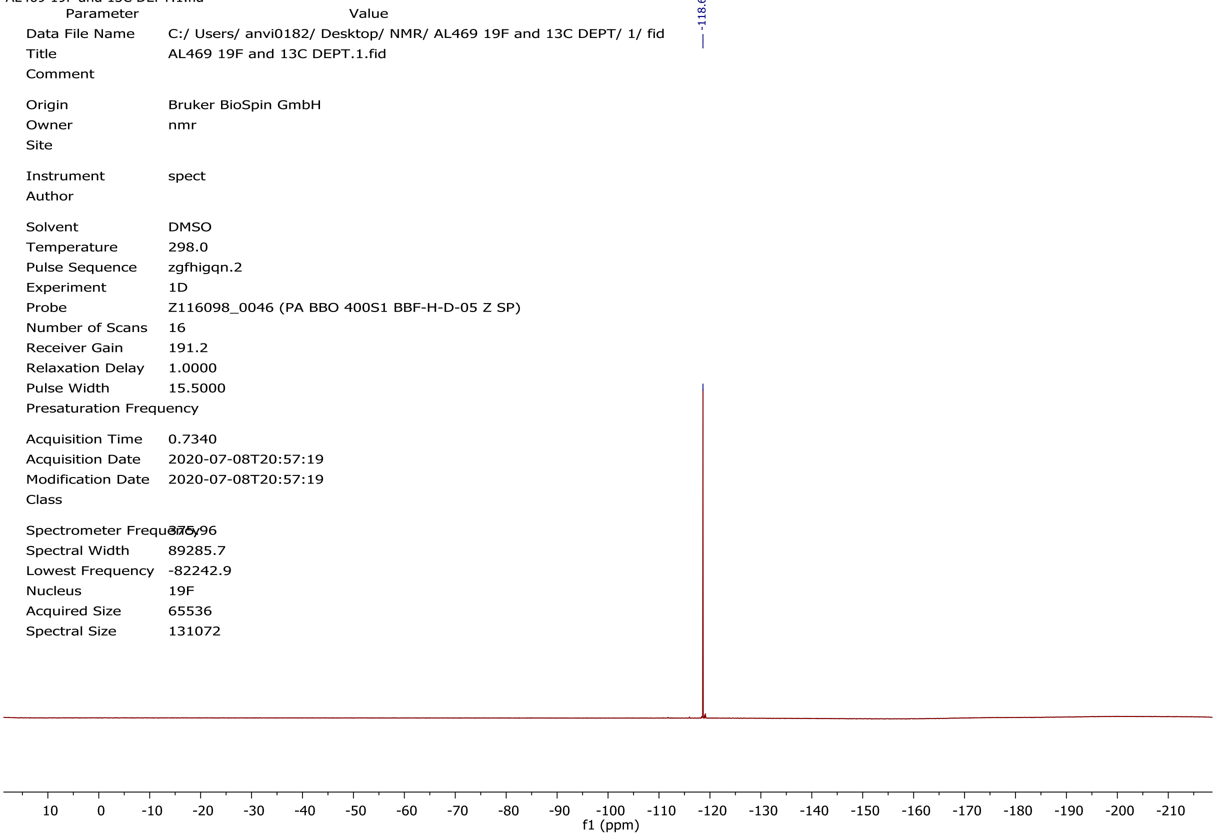

Parameter	Value
Data File Name	C:/ Users/ anvi0182/ Desktop/ NMR/ AL469 19F and 13C DEPT/ 1/ fid
Title	AL469 19F and 13C DEPT.1.fid
Comment	
Origin	Bruker BioSpin GmbH
Owner	nmr
Site	
Instrument	spect
Author	
Solvent	DMSO
Temperature	298.0
Pulse Sequence	zgfhigqn.2
Experiment	1D
Probe	Z116098_0046 (PA BBO 400S1 BBF-H-D-05 Z SP)
Number of Scans	16
Receiver Gain	191.2
Relaxation Delay	1.0000
Pulse Width	15.5000
Presaturation Frequency	
Acquisition Time	0.7340
Acquisition Date	2020-07-08T20:57:19
Modification Date	2020-07-08T20:57:19
Class	
Spectrometer Frequency	375.96
Spectral Width	89285.7
Lowest Frequency	-82242.9
Nucleus	19F
Acquired Size	65536
Spectral Size	131072

57

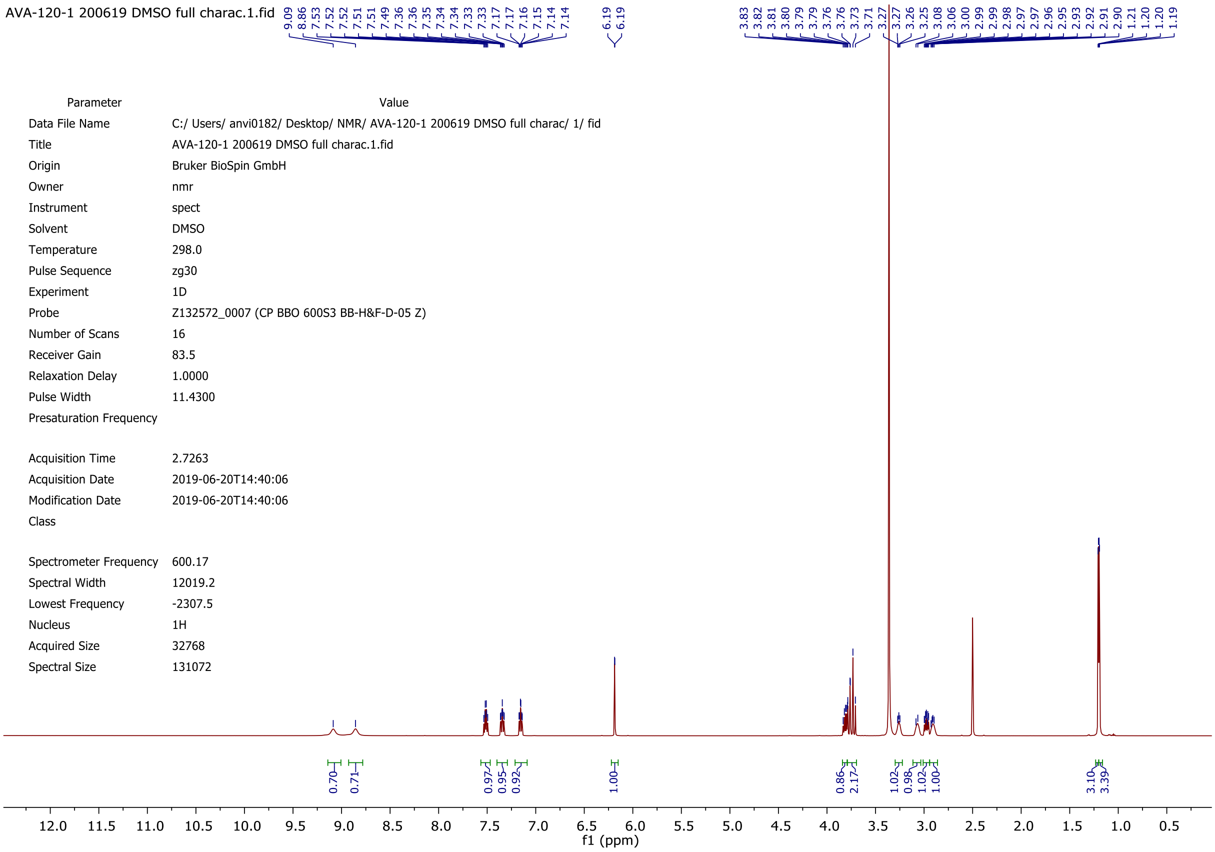

Parameter	Value
Data File Name	C:/ Users/ anvi0182/ Desktop/ NMR/ AVA-120-1 200619 DMSO full charac/ 1/ fid
Title	AVA-120-1 200619 DMSO full charac.1.fid
Origin	Bruker BioSpin GmbH
Owner	nmr
Instrument	spect
Solvent	DMSO
Temperature	298.0
Pulse Sequence	zg30
Experiment	1D
Probe	Z132572_0007 (CP BBO 600S3 BB-H&F-D-05 Z)
Number of Scans	16
Receiver Gain	83.5
Relaxation Delay	1.0000
Pulse Width	11.4300
Presaturation Frequency	
Acquisition Time	2.7263
Acquisition Date	2019-06-20T14:40:06
Modification Date	2019-06-20T14:40:06
Class	
Spectrometer Frequency	600.17
Spectral Width	12019.2
Lowest Frequency	-2307.5
Nucleus	1H
Acquired Size	32768
Spectral Size	131072

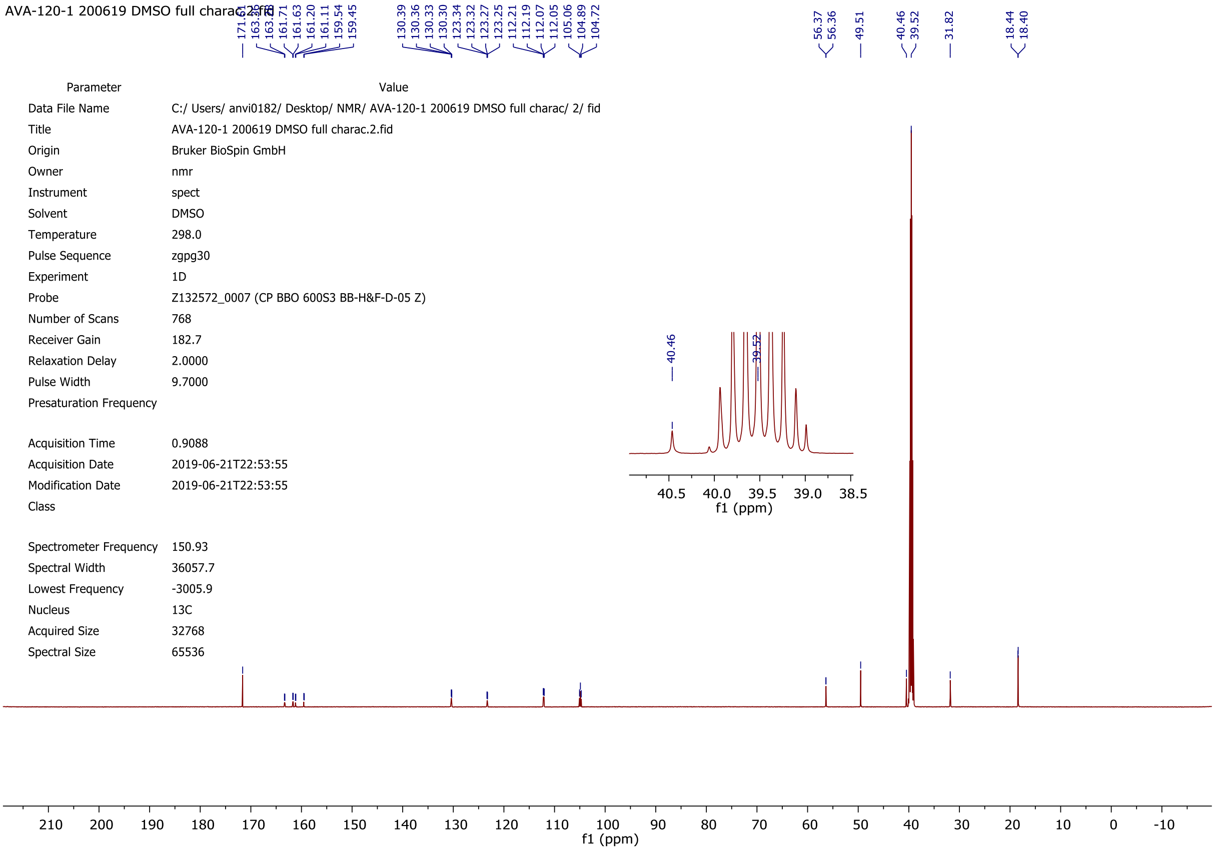

Parameter	Value
Data File Name	C:/ Users/ anvi0182/ Desktop/ NMR/ AVA-120-1 200619 DMSO full charac/ 2/ fid
Title	AVA-120-1 200619 DMSO full charac.2.fid
Origin	Bruker BioSpin GmbH
Owner	nmr
Instrument	spect
Solvent	DMSO
Temperature	298.0
Pulse Sequence	zgpg30
Experiment	1D
Probe	Z132572_0007 (CP BBO 600S3 BB-H&F-D-05 Z)
Number of Scans	768
Receiver Gain	182.7
Relaxation Delay	2.0000
Pulse Width	9.7000
Presaturation Frequency	
Acquisition Time	0.9088
Acquisition Date	2019-06-21T22:53:55
Modification Date	2019-06-21T22:53:55
Class	
Spectrometer Frequency	150.93
Spectral Width	36057.7
Lowest Frequency	-3005.9
Nucleus	13C
Acquired Size	32768
Spectral Size	65536

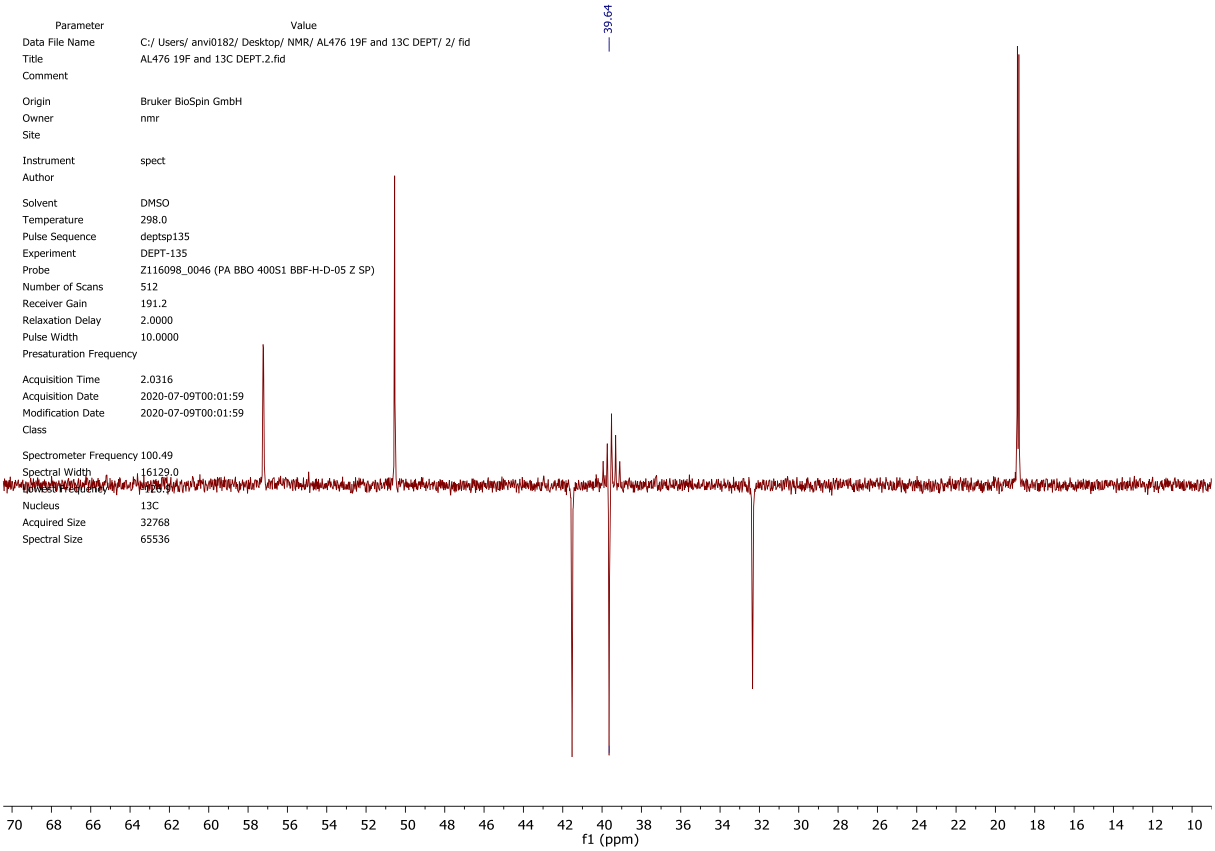

AL476 19F and 13C DEPT.1.fid

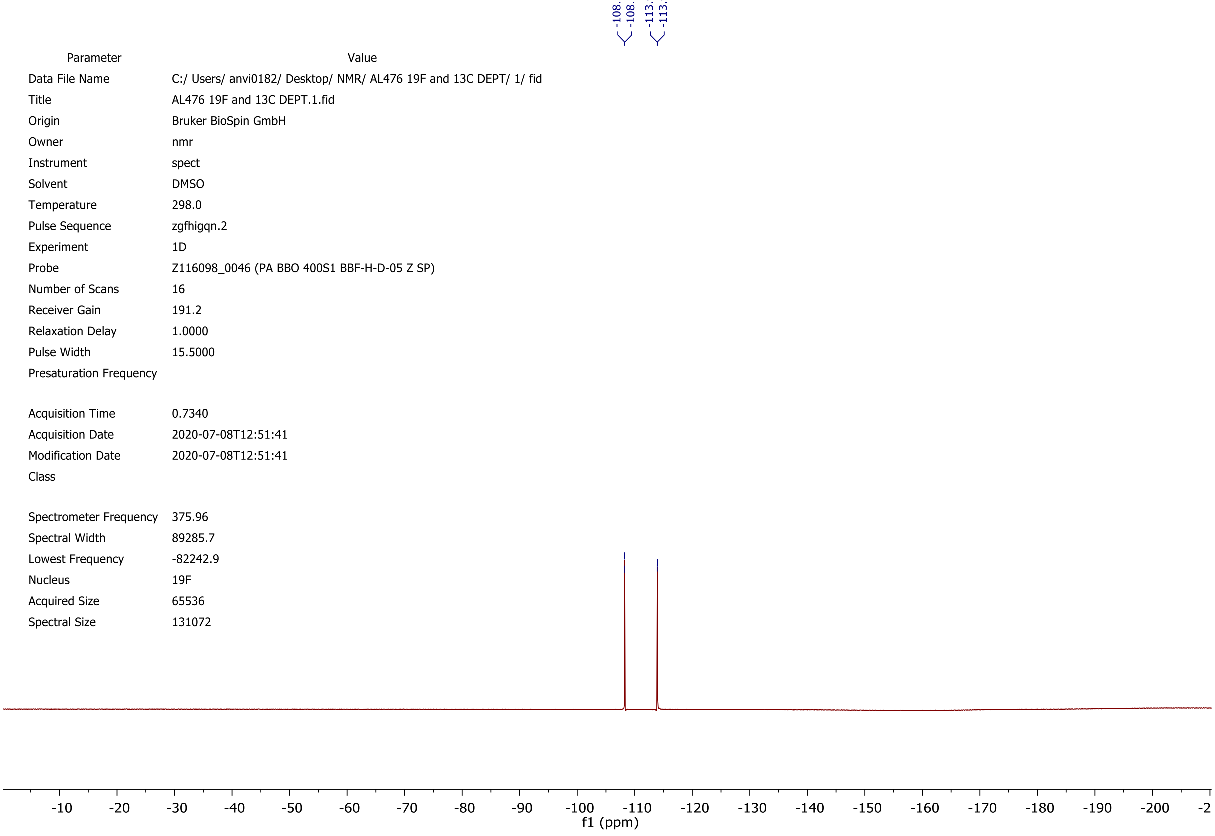

58

AVA-119-1 200619 DMSO full charac.1.fid

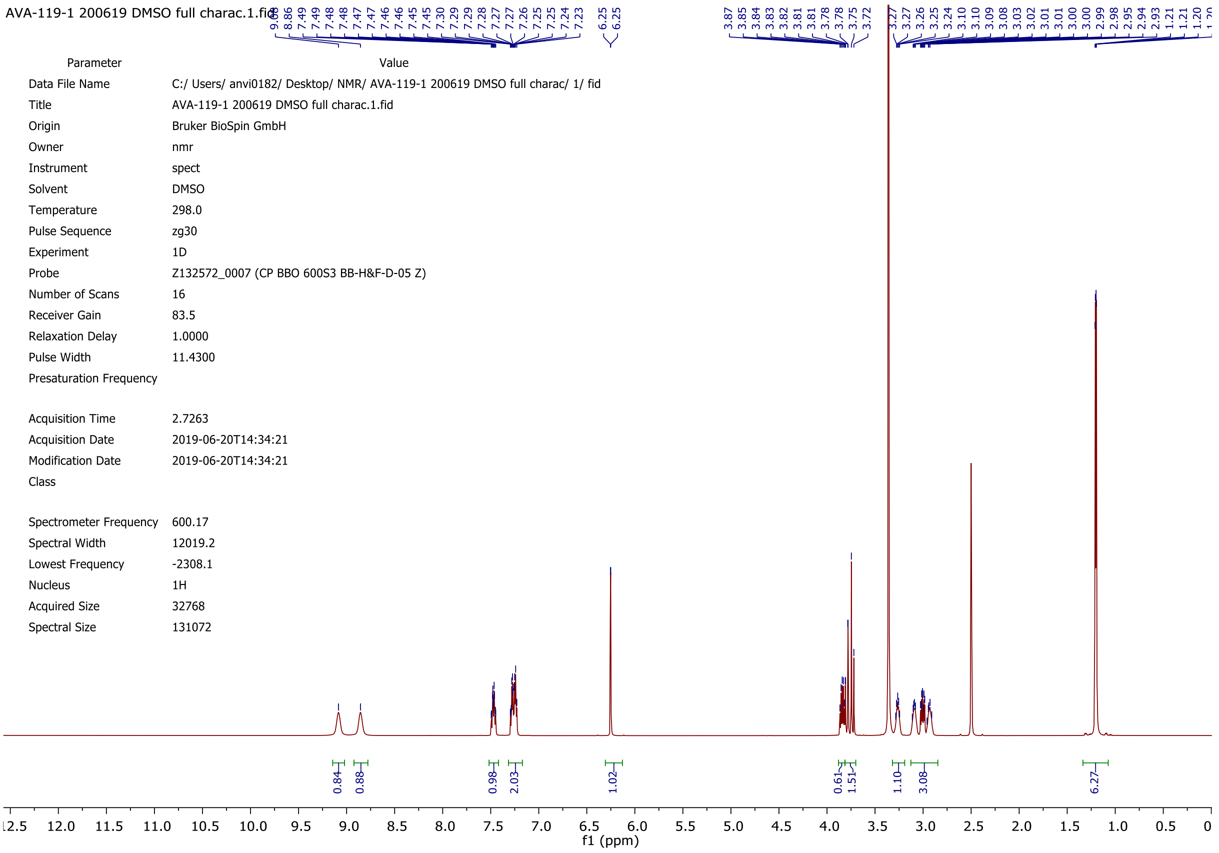

Parameter	Value
Data File Name	C:/ Users/ anvi0182/ Desktop/ NMR/ AVA-119-1 200619 DMSO full charac/ 1/ fid
Title	AVA-119-1 200619 DMSO full charac.1.fid
Origin	Bruker BioSpin GmbH
Owner	nmr
Instrument	spect
Solvent	DMSO
Temperature	298.0
Pulse Sequence	zg30
Experiment	1D
Probe	Z132572_0007 (CP BBO 600S3 BB-H&F-D-05 Z)
Number of Scans	16
Receiver Gain	83.5
Relaxation Delay	1.0000
Pulse Width	11.4300
Presaturation Frequency	
Acquisition Time	2.7263
Acquisition Date	2019-06-20T14:34:21
Modification Date	2019-06-20T14:34:21
Class	
Spectrometer Frequency	600.17
Spectral Width	12019.2
Lowest Frequency	-2308.1
Nucleus	1H
Acquired Size	32768
Spectral Size	131072

AVA-119-1 200619 DMSO full charac.2.fid

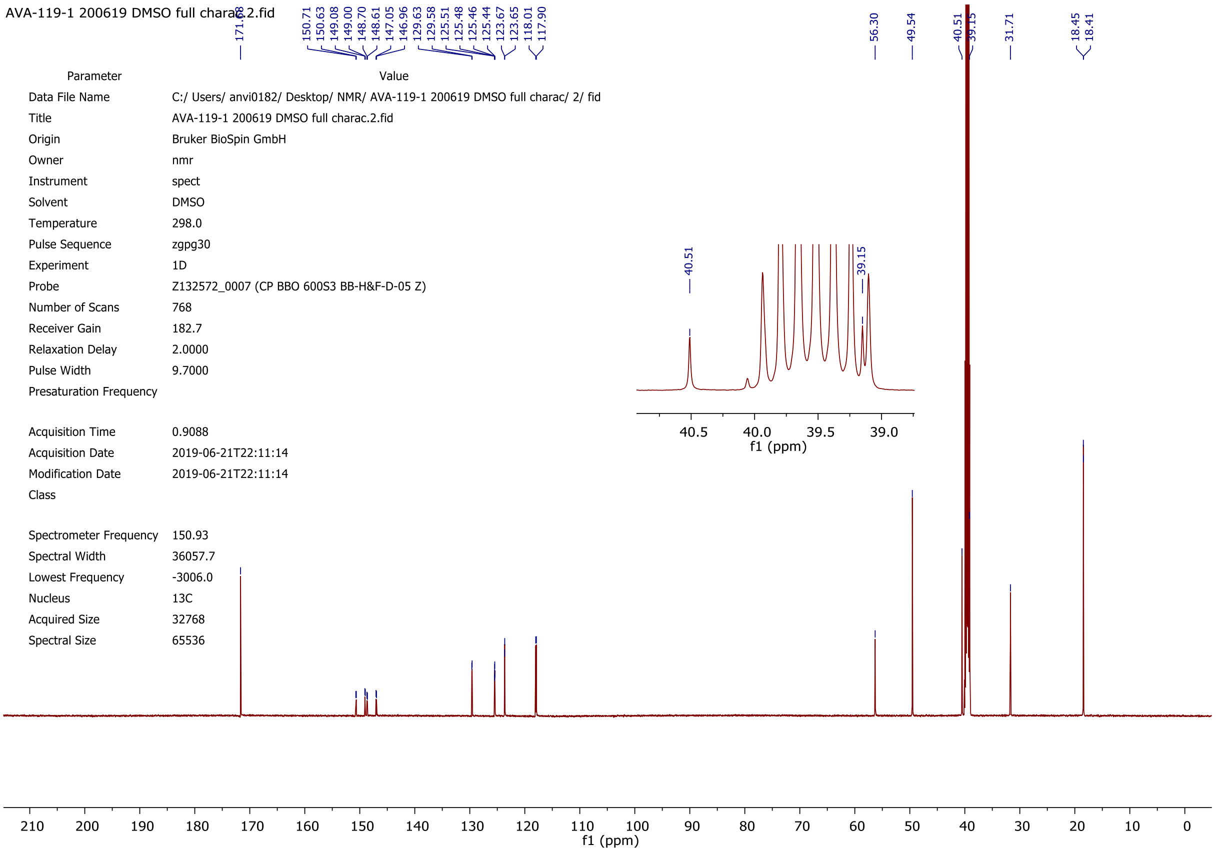

Parameter	Value
Data File Name	C:/ Users/ anvi0182/ Desktop/ NMR/ AVA-119-1 200619 DMSO full charac/ 2/ fid
Title	AVA-119-1 200619 DMSO full charac.2.fid
Origin	Bruker BioSpin GmbH
Owner	nmr
Instrument	spect
Solvent	DMSO
Temperature	298.0
Pulse Sequence	zgpg30
Experiment	1D
Probe	Z132572_0007 (CP BBO 600S3 BB-H&F-D-05 Z)
Number of Scans	768
Receiver Gain	182.7
Relaxation Delay	2.0000
Pulse Width	9.7000
Presaturation Frequency	
Acquisition Time	0.9088
Acquisition Date	2019-06-21T22:11:14
Modification Date	2019-06-21T22:11:14
Class	
Spectrometer Frequency	150.93
Spectral Width	36057.7
Lowest Frequency	-3006.0
Nucleus	13C
Acquired Size	32768
Spectral Size	65536

AL475 19F.1.fid

Parameter	Value
Data File Name	C:/ Users/ anvi0182/ Desktop/ NMR/ AL475 19F/ 1/ fid
Title	AL475 19F.1.fid
Comment	
Origin	Bruker BioSpin GmbH
Owner	nmr
Site	
Instrument	spect
Author	
Solvent	DMSO
Temperature	298.0
Pulse Sequence	zgfhigqn.2
Experiment	1D
Probe	Z116098_0046 (PA BBO 400S1 BBF-H-D-05 Z SP)
Number of Scans	16
Receiver Gain	191.2
Relaxation Delay	1.0000
Pulse Width	15.5000
Presaturation Frequency	
Acquisition Time	0.7340
Acquisition Date	2020-07-08T12:46:29
Modification Date	2020-07-08T12:46:29
Class	
Spectrometer Frequency	375.96
Spectral Width	89285.7
Lowest Frequency	-82242.9
Nucleus	19F
Acquired Size	65536
Spectral Size	131072

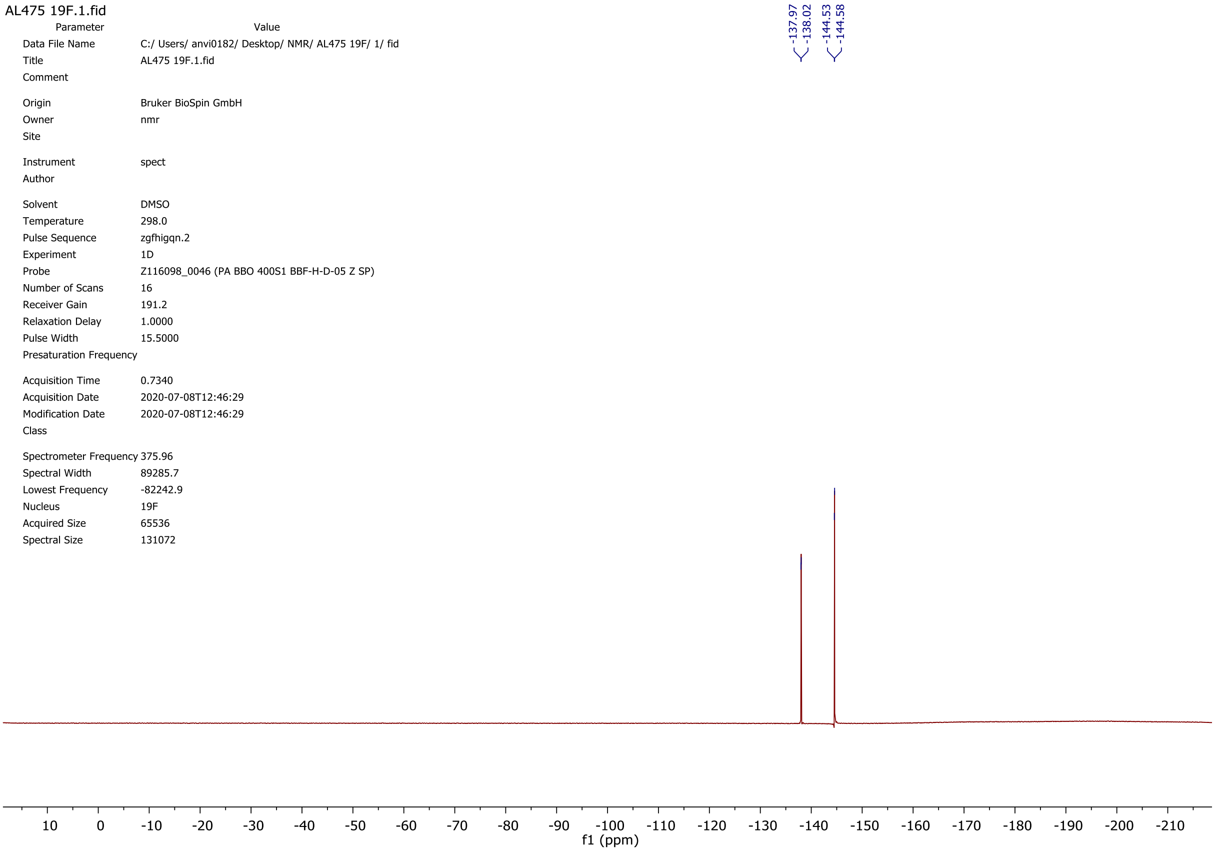

Analytical HPLC of the compounds tested against the mutant G122S-*Ag*AChE1, *Aa*AChE1, including the compounds selected for enzyme kinetics and in vivo studies.

18

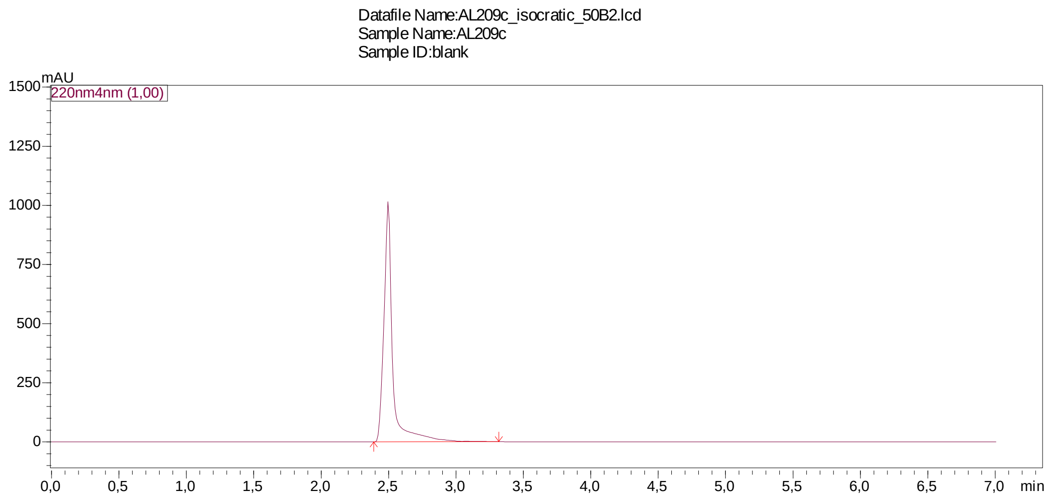

Peak#	Ret. Time	Area	Height	Conc.	Name	Area%
1	2,497	4585848	1014872	0,000		100,000
Total		4585848	1014872	0,000		100,000

4

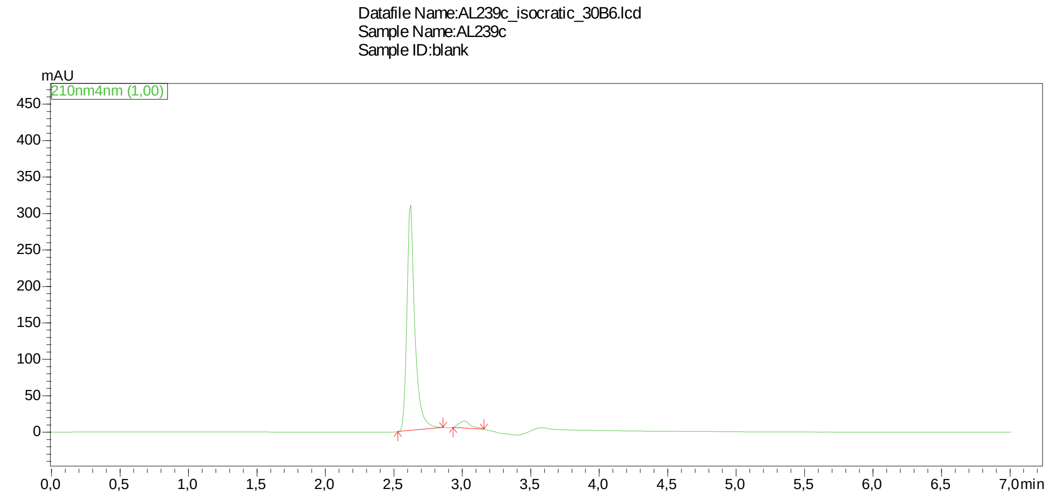

Peak#	Ret. Time	Area	Height	Conc.	Name	Area%
1	2,620	1246723	309053	0,000		95,667
2	3,017	56462	9886	0,000		4,333
Total		1303185	318938	0,000		100,000

20

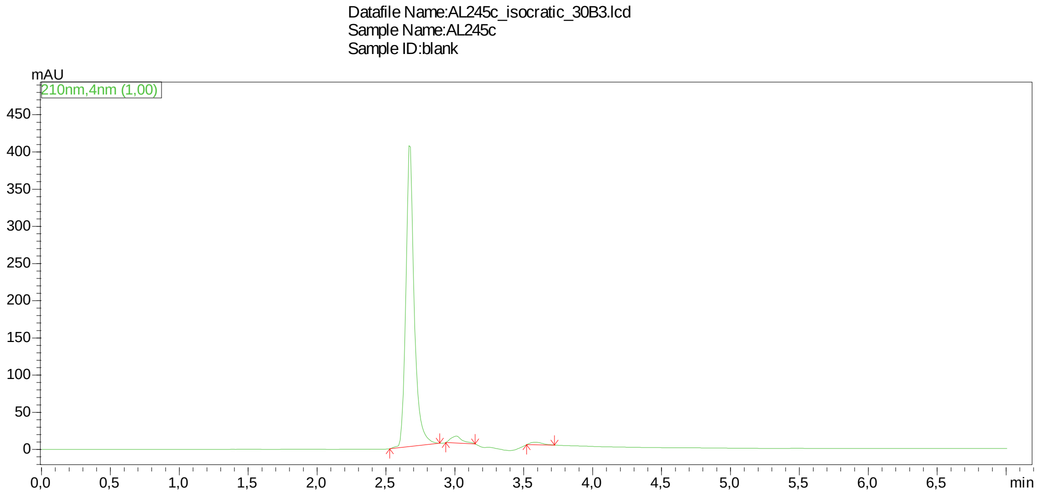

Peak#	Ret. Time		Area	Height	Conc.	Name	Area%
1	2,672	1714931		404271	0,000		95,813
2	3,009	53767	9422	0,000		3,004	
3	3,587	21183	3483	0,000		1,183	
Total		1789882		417176	0,000		100,000

23

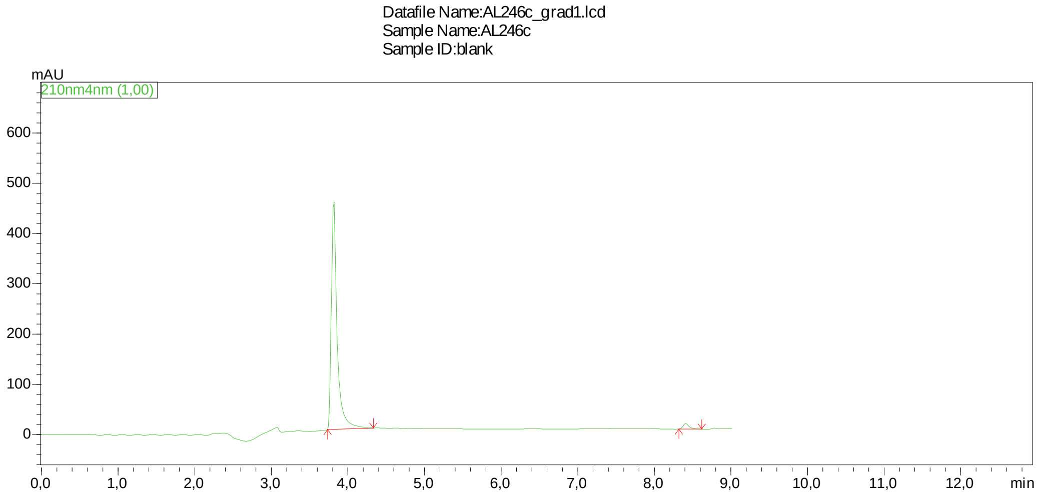

Peak#	Ret. Time		Area	Height	Conc.	Name	Area%
1	3,815	2380335		452347	0,000		97,493
2	8,409	61205	10965	0,000		2,507	
Total		2441540		463312	0,000		100,000

21

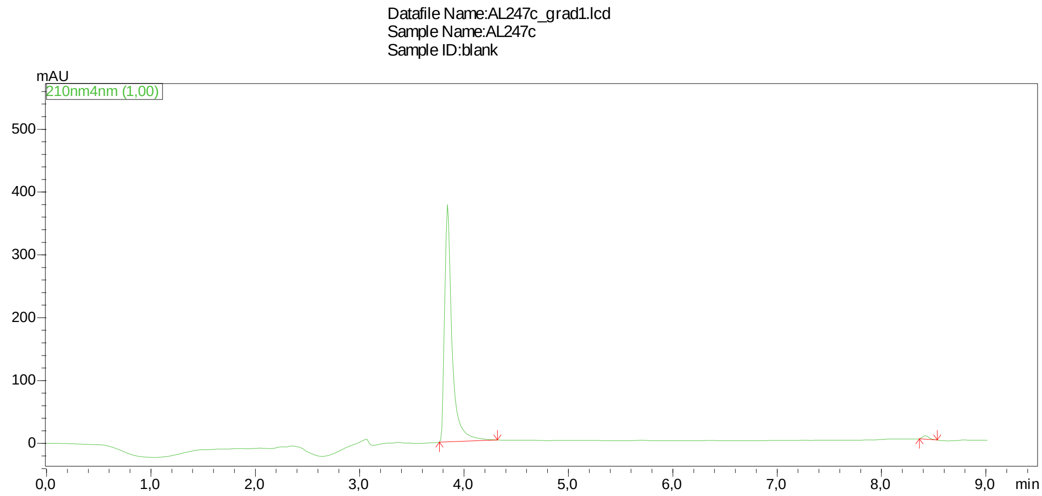

Peak#	Ret. Time		Area	Height	Conc.	Name	Area%
1	3,842	1875389		377493	0,000		98,791
2	8,420	22960	5330	0,000		1,209	
Total		1898349		382823	0,000		100,000

19

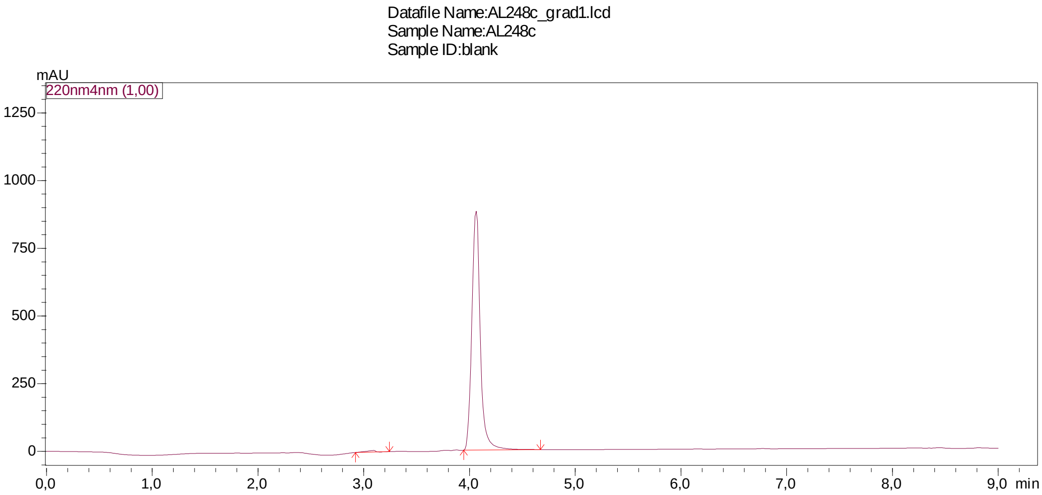

Peak#	Ret. Time		Area	Height	Conc.	Name	Area%
1	3,091	33345	5955	0,000		0,672	
2	4,062	4930898		882028	0,000		99,328
Total		4964243		887983	0,000		100,000

30

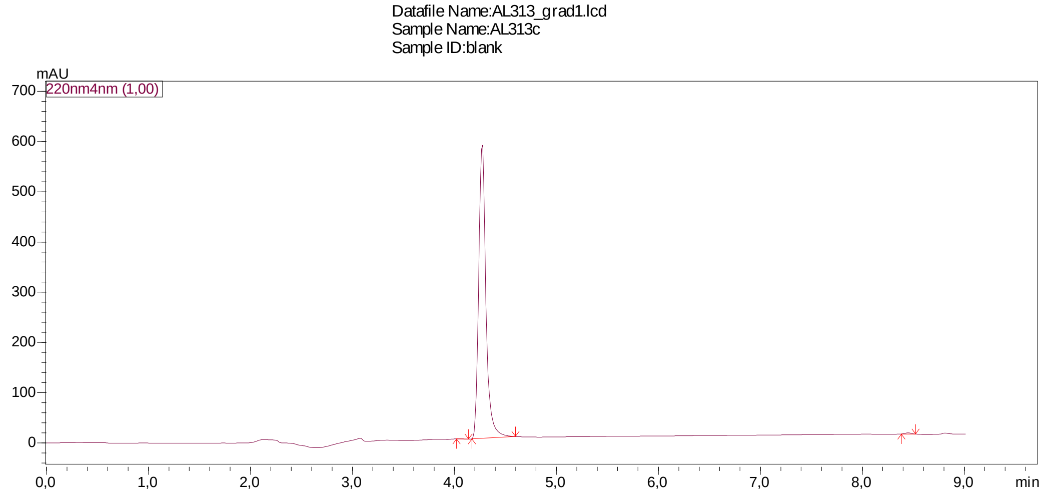

Peak#	Ret. Time		Area	Height	Conc.	Name	Area%	
1	4,067	761	352	0,000		0,026		
2	4,273	2902404		583556	0,000		99,648	
3	8,451	9494	2365	0,000		0,326		
Total		2912658		586273	0,000		100,000	

36

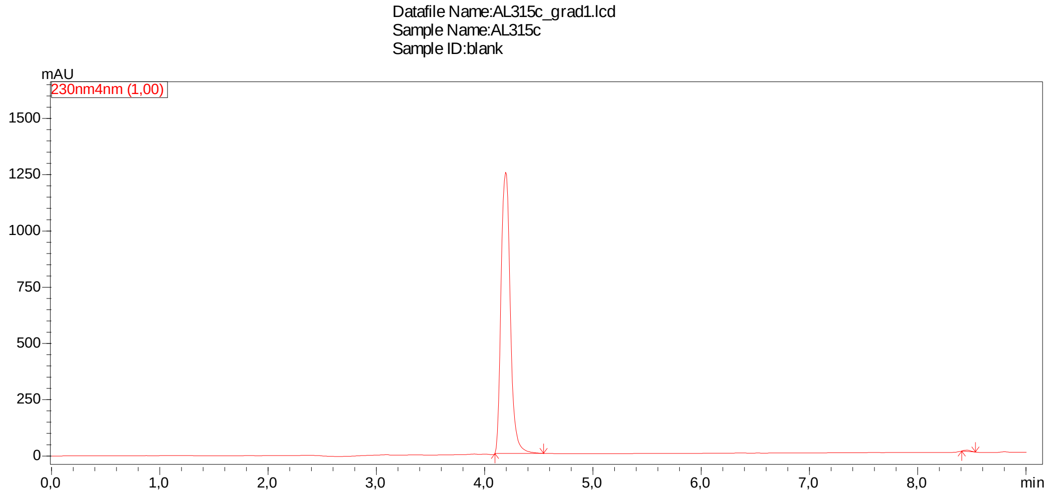

Peak#	Ret. Time		Area	Height	Conc.	Name	Area%	
1	4,195	7420738		1249822		0,000		99,697
2	8,448	22556	5489	0,000		0,303		
Total		7443294		1255311		0,000		100,000

24

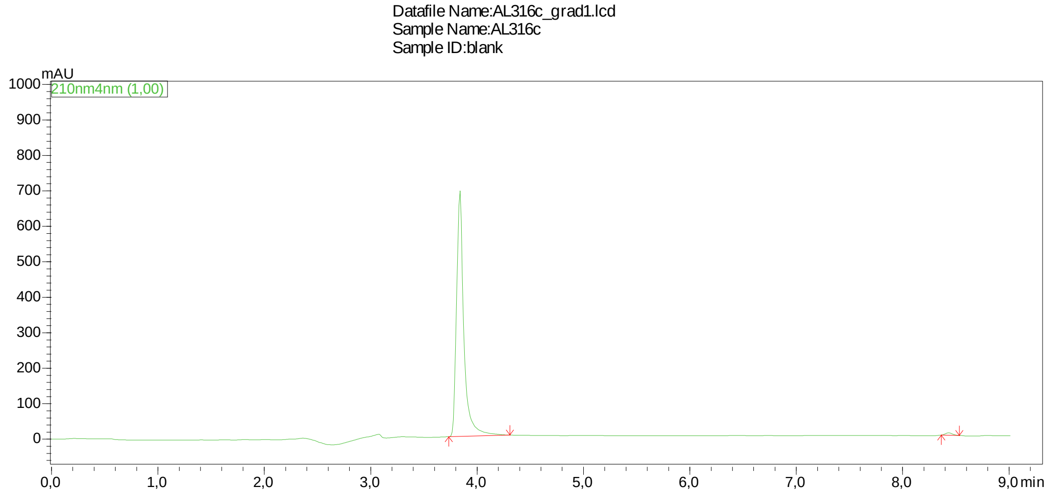

Peak#	Ret. Time		Area	Height	Conc.	Name	Area%	
1	3,838	3304190		692321	0,000		99,090	
2	8,432	30340	6763	0,000		0,910		
Total		3334531		699084	0,000		100,000	

50

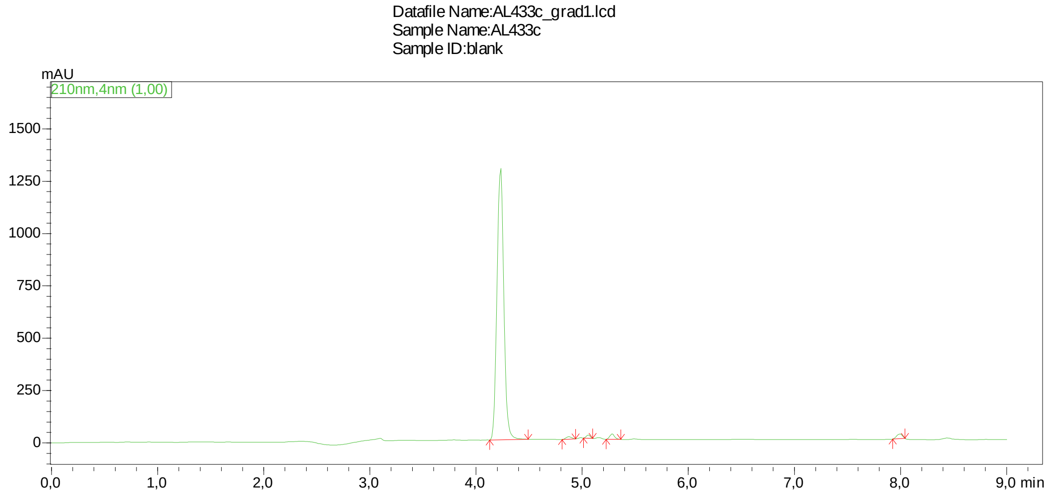

Peak#	Ret. Time		Area	Height	Conc.	Name	Area%	
1	4,232	5828116		1296049		0,000		95,882
2	4,875	44163	12035	0,000		0,727		
3	5,058	39986	15848	0,000		0,658		
4	5,282	81343	25774	0,000		1,338		
5	7,994	84811	21800	0,000		1,395		

Total 6078420 1371506 0,000 100,000

41

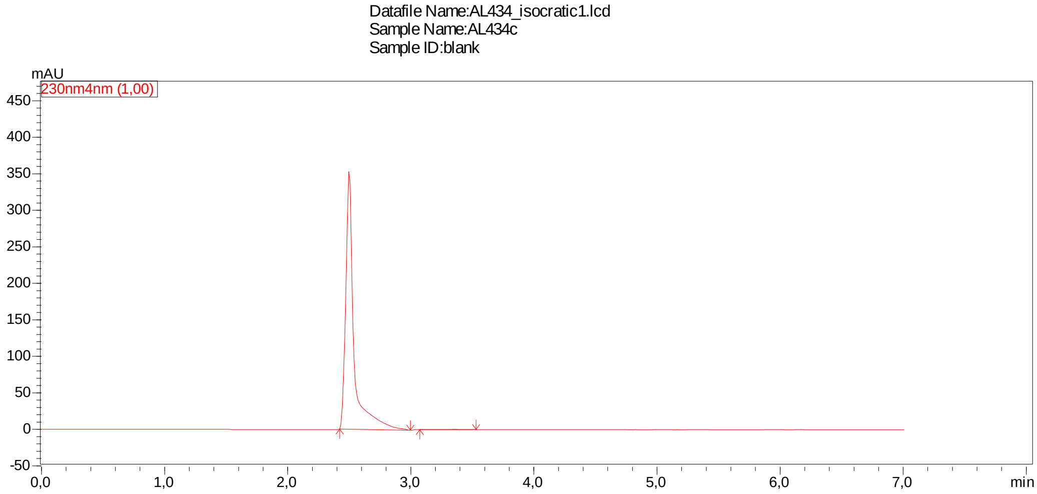

Peak#	Ret. Time		Area	Height	Conc.	Name	Area%
1	2,499	1626640		352594	0,000		99,841
2	3,353	2585	267	0,000		0,159	
Total		1629225		352861	0,000		100,000

42

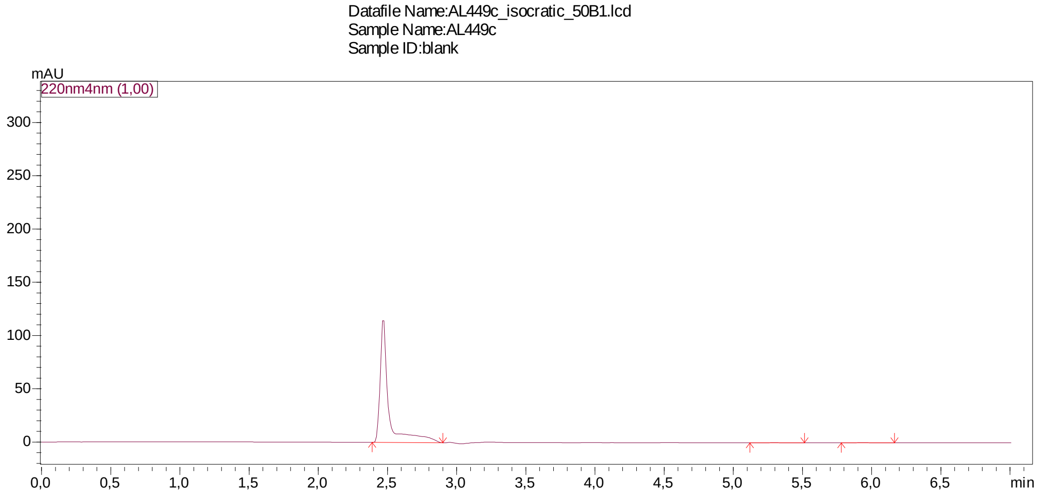

2,469	503416	114364	0,000		99,351
5,291	1621	158	0,000		0,320
5,927	1667	179	0,000		0,329

506704 114701 0,000 100,000

51

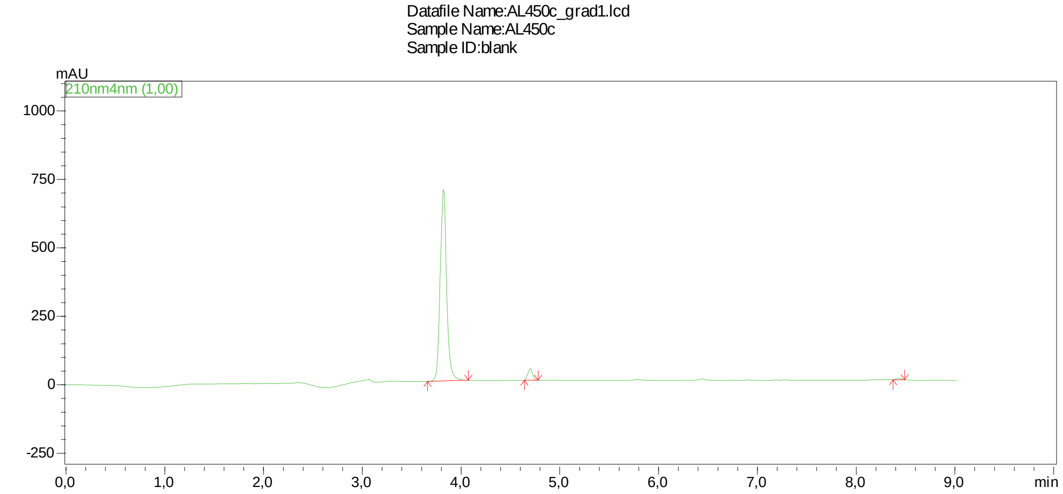

Peak#	Ret. Time	Area	Height	Conc.	Name	Area%
1	3,823	3188143	699841	0,000		95,137
2	4,701	143836 42336	0,000		4,292	
3	8,430	19140 5068	0,000		0,571	
Total		3351119	747246	0,000		100,000

26

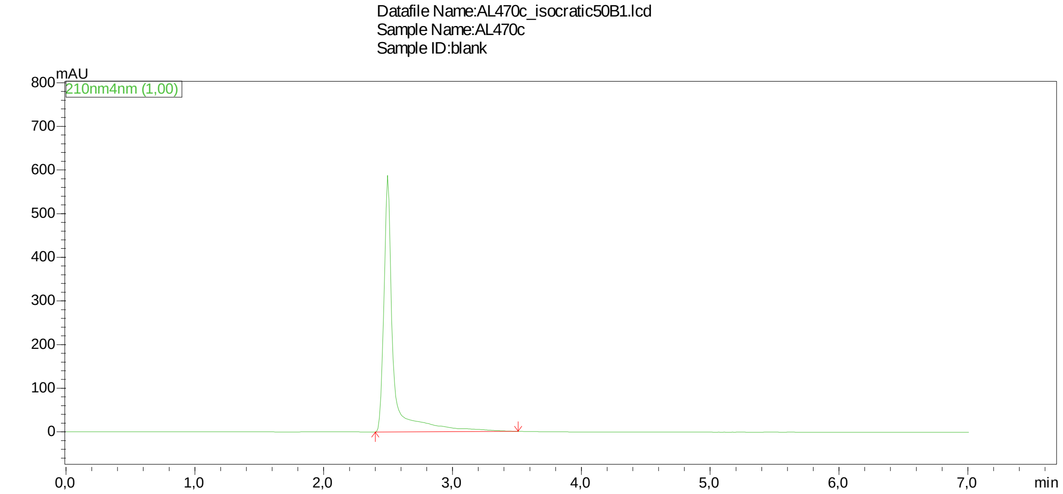

Peak#	Ret. Time	Area	Height	Conc.	Name	Area%
1	2,497	2974885	587637	0,000		100,000
Total		2974885	587637	0,000		100,000

33

Datafile Name:AL472c_isocratic_50B3.lcd
Sample Name:AL472c
Sample ID:blank

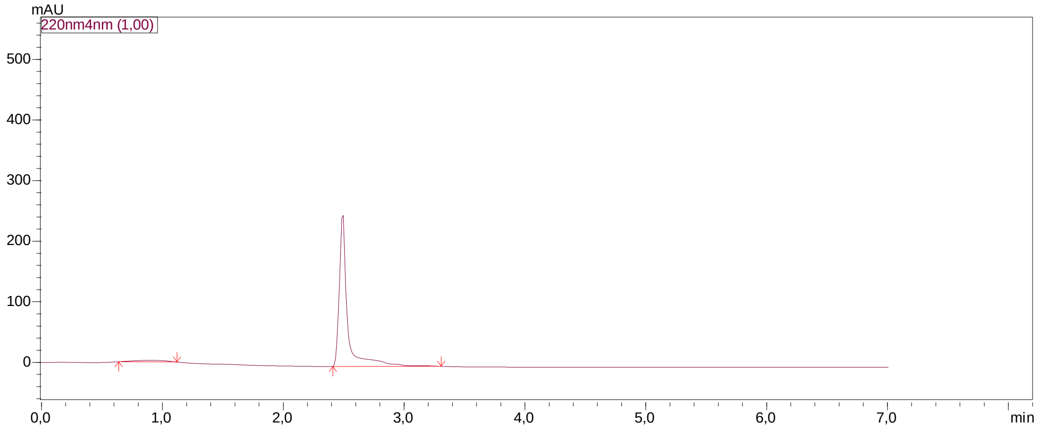

Peak#	Ret. Time		Area	Height	Conc.	Name	Area%
1	0,909	47828	2680	0,000		3,836	
2	2,491	1199053		249231	0,000		96,164
Total		1246881		251910	0,000		100,000

31

Datafile Name:AL473c_isocratic_50B1.lcd
Sample Name:AL473c
Sample ID:blank

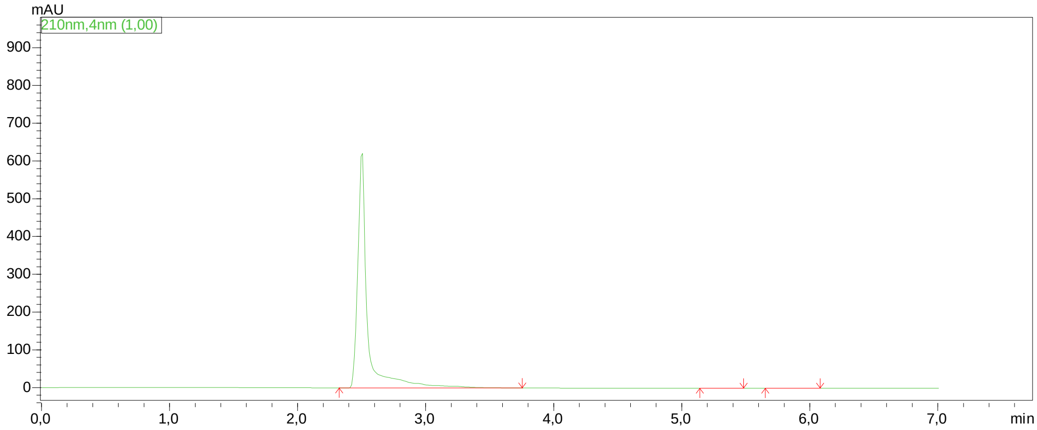

Peak#	Ret. Time		Area	Height	Conc.	Name	Area%
1	2,502	3255593		621106	0,000		99,823
2	5,261	1522	150	0,000		0,047	
3	5,816	4243	366	0,000		0,130	
Total		3261358		621623	0,000		100,000

40 (isocratic)

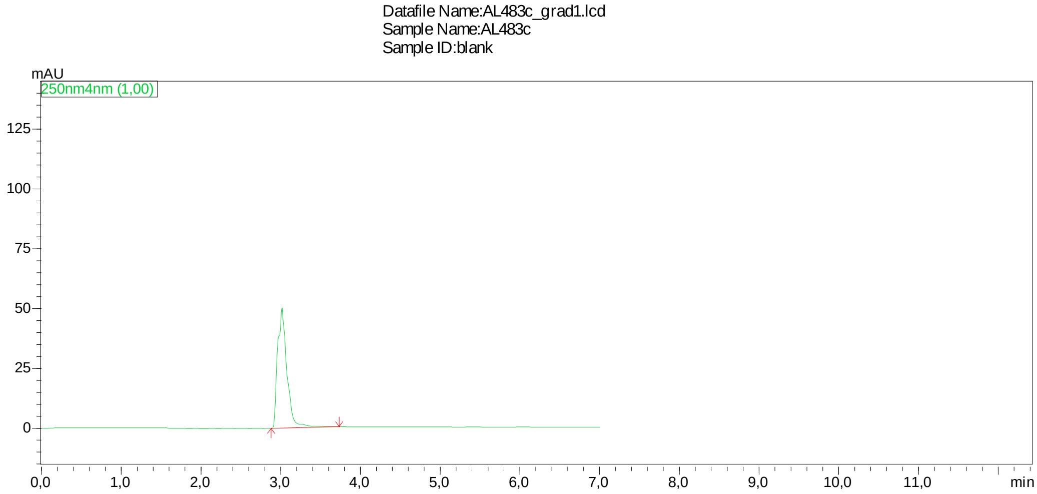

Peak#	Ret. Time	Area	Height	Conc.	Name	Area%
1	3,015	410008	50182	0,000		100,000
Total		410008	50182	0,000		100,000

34

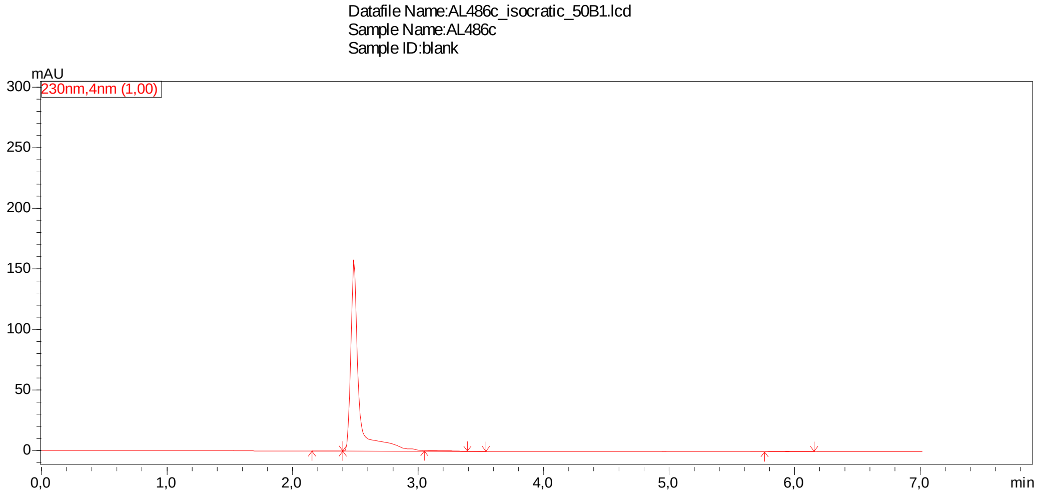

Peak#	Ret. Time	Area	Height	Conc.	Name	Area%
1	2,289	1200	132	0,000		0,165
2	2,488	720036	158024	0,000		99,150
3	3,123	2697	238	0,000		0,371
4	5,934	2277	212	0,000		0,314
Total		726209	158605	0,000		100,000

46

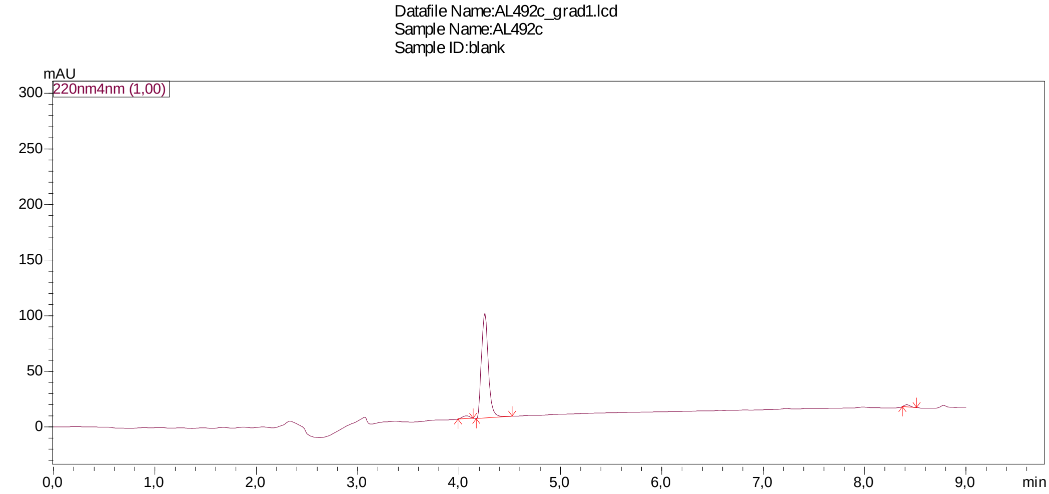

Peak#	Ret. Time		Area	Height	Conc.	Name	Area%
1	4,078	12134	2417	0,000		2,628	
2	4,254	442913	94380	0,000		95,913	
3	8,416	6741	1990	0,000		1,460	
Total		461788	98787	0,000		100,000	

32

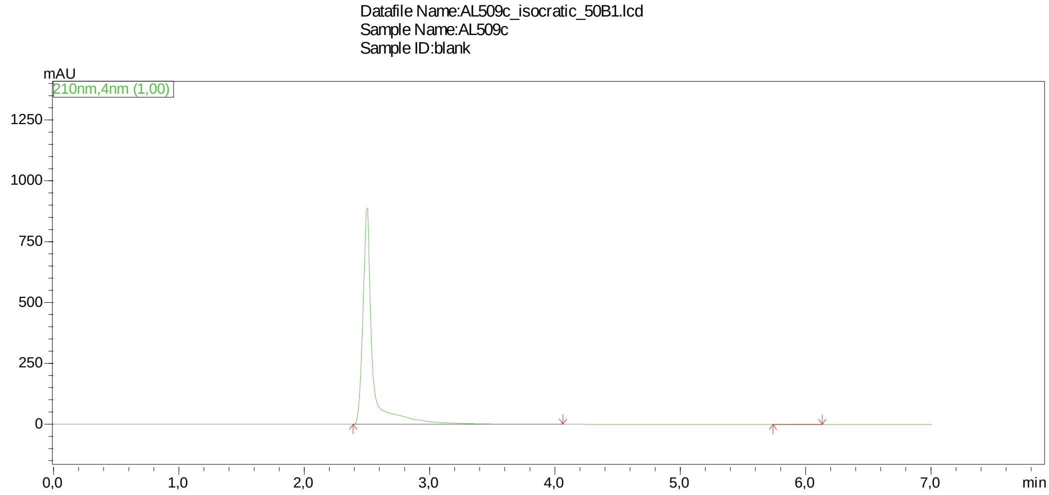

Peak#	Ret. Time		Area	Height	Conc.	Name	Area%
1	2,502	4713528		888444	0,000		99,959
2	5,926	1932	201	0,000		0,041	
Total		4715460		888645	0,000		100,000

22

Datafile Name:AL510c_isocratic_50B1.lcd
Sample Name:AL510c
Sample ID:blank

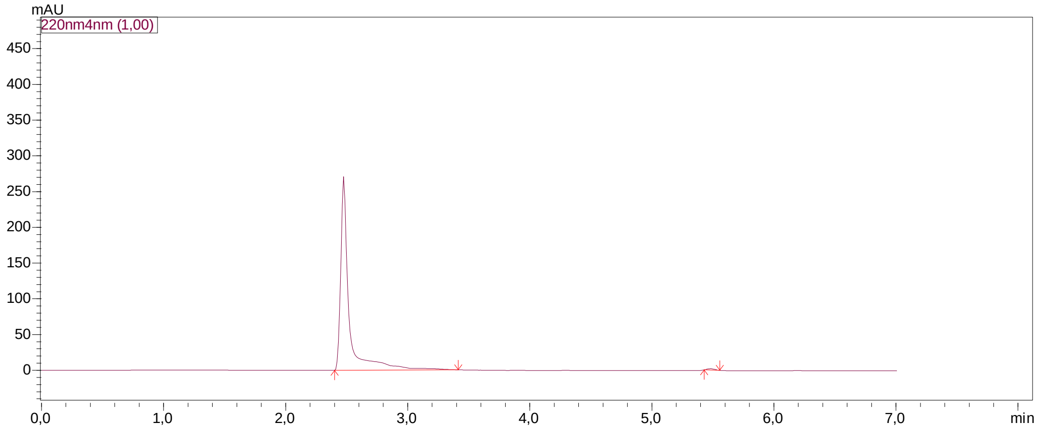

Peak#	Ret. Time		Area	Height	Conc.	Name	Area%
1	2,475	1296319		270733	0,000		99,406
2	5,480	7745	1860	0,000		0,594	
Total		1304064		272593	0,000		100,000
